# Integrated Analyses Identify Immune-Related Signature Associated with Qingyihuaji Formula for Treatment of Pancreatic Ductal Adenocarcinoma Using Network Pharmacology and Weighted Gene Co-Expression Network

**DOI:** 10.1155/2020/7503605

**Published:** 2020-05-20

**Authors:** Xiang Qian, Zhuo Chen, Sha Sha Chen, Lu Ming Liu, Ai Qin Zhang

**Affiliations:** ^1^Institute of Cancer and Basic Medicine (ICBM), Chinese Academy of Sciences, Hangzhou, China; ^2^Cancer Hospital of the University of Chinese Academy of Sciences, Hangzhou, China; ^3^Zhejiang Cancer Hospital, Hangzhou, China; ^4^Department of Traditional Chinese Medicine, Taizhou Cancer Hospital, Zhejiang, China; ^5^Department of Integrative Oncology, Fudan University Shanghai Cancer Center, China

## Abstract

The study aimed to clarify the potential immune-related targets and mechanisms of Qingyihuaji Formula (QYHJ) against pancreatic cancer (PC) through network pharmacology and weighted gene co-expression network analysis (WGCNA). Active ingredients of herbs in QYHJ were identified by the TCMSP database. Then, the putative targets of active ingredients were predicted with SwissTargetPrediction and the STITCH databases. The expression profiles of GSE32676 were downloaded from the GEO database. WGCNA was used to identify the co-expression modules. Besides, the putative targets, immune-related targets, and the critical module genes were mapped with the specific disease to select the overlapped genes (OGEs). Functional enrichment analysis of putative targets and OGEs was conducted. The overall survival (OS) analysis of OGEs was investigated using the Kaplan-Meier plotter. The relative expression and methylation levels of OGEs were detected in UALCAN, human protein atlas (HPA), Oncomine, DiseaseMeth version 2.0 and, MEXPRESS database, respectively. Gene set enrichment analysis (GSEA) was conducted to elucidate the key pathways of highly-expressed OGEs further. OS analyses found that 12 up-regulated OGEs, including CDK1, PLD1, MET, F2RL1, XDH, NEK2, TOP2A, NQO1, CCND1, PTK6, CTSE, and ERBB2 that could be utilized as potential diagnostic indicators for PC. Further, methylation analyses suggested that the abnormal up-regulation of these OGEs probably resulted from hypomethylation, and GSEA revealed the genes markedly related to cell cycle and proliferation of PC. This study identified CDK1, PLD1, MET, F2RL1, XDH, NEK2, TOP2A, NQO1, CCND1, PTK6, CTSE, and ERBB2 might be used as reliable immune-related biomarkers for prognosis of PC, which may be essential immunotherapies targets of QYHJ.

## 1. Introduction

Pancreatic cancer (PC) is the most common cause of cancer mortality globally, which causes an estimated 227,000 deaths per year [1] and more than 52% cases with a 5-year survival rate of less than 5% at a distant stage [2, 3]. Pancreatic ductal adenocarcinoma (PDAC) is one of the most common histological types of the exocrine pancreas and accounts for 95% of all PC patients [4], which had been characterized by local invasion and even distant metastasis at the initial diagnosis because its clinical presentation is lack of specific biomarkers. In the past few decades, though the several diagnostic tools have emerged. Surgical resection is known as the only potentially curative therapy in patients with PDAC, but only less than 20% of the patients are eligible for tumor resection [5]. Despite receiving curative resection, the cancer relapse can lead to metastatic disease that directly results in an ominous prognosis. Additionally, the pathogenesis of PDAC at the molecular level remains unsubstantial. Currently, part of the tumor markers has been strictly identified to suggest the prognosis of patients with PDAC [3, 6]. Consequently, the promising therapeutic avenues and the novel immune-related biomarkers with increased specificity for early diagnosis are urgently needed.

Traditional Chinese medicine (TCM) is one of the historic medical systems, which has been utilized in China for 3000 years [7]. Accumulating evidence suggested that TCM contributes to treating multifarious diseases, including malignancies [8, 9]. The novel TCM formula Qingyihuaji (QYHJ) is produced by professor Luming Liu, which composed of Banzhilian (*Herba Scutellariae Barbatae,* HSB), Baihuasheshecao (*HerbaHedyotdis,* HBHY), Tiannanxing (*Rhizoma Arisaematis erubescentis,* RAE), Jiaogulan (*Herba seu Radix Gynostemmatis pentaphylli,* HSRGP), and Doukou (*Fructus Amomi Rotundus,* FAR), which was widely used for the treatment of PC during the past decades [10–12]. Besides, our previous clinical studies suggested that QYHJ treatment combined with first-line clinical western medicine improves the survival of PC patients with liver metastases [11, 13]. Thus, QYHJ might be an innovative therapeutic avenue for PC; nevertheless, its pharmacological action has not been fully clarified.

Increasing literature supports a vital role in the immune system in PDAC initiation and development. Chinese herbal formulae are considered as multitarget and multicomponent characteristics that utilizing its active components to regulate the immune and body systems [14]. Therefore, a new perspective is required to explore and explain the mechanism of QYHJ systematically and comprehensively. Recently, big data bioinformatics of molecular targets has gained more attention. The public databases, including The Cancer Genome Atlas (TCGA) and Gene Expression Omnibus (GEO), favoring researchers to conduct data mining for the identification of novel genomic targets for therapeutic intervention of PDAC. Moreover, many studies have proven that network pharmacology is a practical approach for drug target prediction from the “multiple targets” perspective to investigate the macro-regulation of TCM [15]. Simultaneously, WGCNA, a systems biology method, is proverbially used in cancers [16]. More importantly, WGCNA, a novel systems biology-based avenue, is widely used in high-throughput microarray since it can identify the specific co-expression modules that associated with the clinical traits for screening out the biomarkers to improve the diagnosis and therapy of cancers in clinical practice [17].

In the current study, network pharmacology was used to identify the special compounds of herbs in QYHJ. The putative targets of these compounds were acquired from the publicly available platforms. Using the GEO database, WGCNA was conducted on the PDAC gene expression file to identify gene co-expression modules that related to the pathological stage and explore the potential hub genes. Then, we used integrated bioinformatics methods to investigate the functional and pathway enrichment of putative targets and the overlapped hub genes. Besides, MEXPRESS and DiseaseMeth 2.0 were utilized to assess the methylation status of those hub genes, while Gene Set Enrichment Analysis (GSEA) was employed to explore potential biological functions. This study aimed to uncover the potential candidate biomarkers and targets of QYHJ for PC treatment, which also supplies a precious chance for a preliminary exploration of the pathogenesis in PDAC.

## 2. Materials and Methods

### 2.1. Collection of Chemical Ingredients in QYHJ

Compound information of herbs in QYHJ was acquired from Traditional Chinese Medicine Systems Pharmacology Database and Analysis Platform [18] (TCMSP, http://tcmspw.com/tcmsp.php), which provides a chemical pharmacokinetic property based on absorption, distribution, metabolism, and excretion (ADME) parameters. In the TCMSP server, the active ingredients of each herbal medicine in QYHJ were mainly filtered by integrating the pharmacokinetic properties comprising oral bioavailability (OB) ⩾30%, drug-likeness (DL) ⩾0.18 [19], as well as half-life (HL)⩾4 h. OB prescreening is employed to estimate an orally administered dose of unchanged drug that reaches the systemic circulation in TCM remedy, which reveals the convergence of the ADME process. DL is a qualitative concept used in drug design to assess whether a compound is chemically suitable for the drug. The ‘drug-like' level of the compounds is 0.18, which is used as a selection criterion for the ‘drug-like' compounds in the traditional Chinese herbs. Drug HL (t1/2), which defined as “the time taken for the amount of compound in the body to fall by half”, which is the most central property as it dictates the timescale of treatment. In this study, the active ingredients were selected for further analysis when they met both of these criteria. Next, the 2D structure of the active ingredients was collected from the PubChem database (https://pubchem.ncbi.nlm.nih.gov/); Draw them with ChemBioDraw Ultra 14.0 software and save as an “sdf” file format. Then, the 2D structure was converted to the simplified molecular-input entry specification (SMILES) file using Open Babel GUI software.

### 2.2. Targets Prediction of QYHJ

The SwissTargetPrediction (http://www.swisstargetprediction.ch/), an online tool, allows the user to assess the most probable macromolecular targets of a small molecule-based on a combination of 2D and 3D chemical similarity [20]. Similarly, STITCH (http://stitch.embl.de/), a free public web-server, has been used extensively in TCM to investigate the molecular mechanism of the potential effective components according to text mining and molecular docking methods [21]. The SMILES information of the active ingredients was imported into the SwissTargetPrediction and the STITCH databases, respectively. The probability value of each potential target listed in SwissTargetPrediction database was used to investigate the accuracy of the current predictions, whose probability value ≥0.1 was identified in this study; additionally, the potential target proteins with confidence score ≥ 7 in the STITCH databases were collected as putative targets of QYHJ [22]. Generally, we obtained 675 putative targets when integrating redundant data.

### 2.3. Identification of PDAC and Immune-Related Genes

The target genes related to PDAC were collected from the GeneCards database (https://www.genecards.org/), which is an integrative database that provides user-friendly information on all annotated and predicted human genes by automatically integrates gene-centric data from ~150 web sources [23]. The keyword “Pancreatic ductal adenocarcinoma” and “Immune” were used in the GeneCards database to search for PDAC-/Immune-related targets, respectively, and finally obtained a total of 3,208 and 15,877 genes related to PDAC and Immune, respectively, from the database (Table 1S and Table 2S).

### 2.4. PDAC Microarray Data Collection and Data Processing

GEO is a public functional genomics data repository, in which the gene expression profile was selected for the current study. We used the keyword “Pancreatic ductal adenocarcinoma” OR “PDAC” AND “Homo sapiens” (Organisms) AND “Expression profiling by array”(Filter), and then corresponding datasets were screened with the following inclusion criteria including pancreas tissue and non-malignant pancreas samples used as controls. The gene expression data of GSE32676 was downloaded from the GEO database. The platform of dataset GSE32676 is the GPL570 (HG-U133_Plus_2) Affymetrix Human Genome U133 Plus 2.0 Array, which includes twenty-five human pancreatic tumor and seven non-malignant pancreas samples, and was used to perform WGCNA for identifying hub model genes in this study. Furthermore, the GEO2R is an interactive web tool based on the GEOquery and limma R packages from the Bioconductor project. To identify DEGs, |log_2_FC| > 1 and *P* < 0.05 were set as cutoff criteria.

### 2.5. Co-Expression Network Construction

The expression data of all genes in the GSE32676 was used to identify the significant gene modules using the WGCNA package in R [24]. A weighted adjacency matrix was constructed using a power function a_mn_ = |c_mn_|^*β*^ (c_mn_ = Pearson's correlation between gene m and gene n; a_mn_ = adjacency between gene m and gene n). An appropriate *β* value was selected to increase the similarity matrix and construct a scale-free co-expression network. Next, the adjacency matrix was transformed into a topological overlap matrix (TOM). The dynamic tree cut algorithm was applied to detect gene modules. Here, we selected soft-thresholding power as 8 (scale-free R^2^ = 0.85), and minimal module size as 30 to identify hub modules. To investigate the connections between gene modules and clinical properties of PDAC, the relationships between the module eigengenes and clinical features were calculated. Gene significance (GS) and module membership (MM) were defined by the correlation coefficient of each module eigengene and each trait, respectively. In general, modules with a higher Pearson's correlation coefficient have more clinical significance. Among genes of active ingredients, GeneCards, and significant module, the overlapped genes (OGEs) were analyzed with Venn diagrams by Venny 2.1 (http://bioinfogp.cnb.csic.es/tools/venny/).

### 2.6. Visualization of Gene Expression Patterns and Chromosome Locations

“RCircos” (R package) was used to reveal the expression patterns and chromosomal locations of the 18 OGEs from Venn diagrams analysis.

### 2.7. Functional Enrichment Analysis and Protein-Protein Interaction (PPI) Network

Gene ontology (GO) and KEGG enrichment analysis of active ingredients related putative targets and the OGEs, respectively, were performed using the Metascape database (http://metascape.org/), which is a gene annotation and analysis resource [25]. *P-value*< 0.05 was set as the cut-off criteria to identify the outstanding GO terms and KEGG pathways and visualized by bubble diagram or “GOplot” (R package). To estimate the interactive associations among the OGEs, meanwhile, the PPI network of 18 shared genes was established by using the STRING (http://string-db.org), which is a database of known and predicted protein-protein interactions [26].

### 2.8. Survival Analysis of OGEs

Kaplan Meier plotter (http://kmplot.com/) is an online tool for interactively investigating survival correlations, which is capable of assessing the effect of 54 k genes on survival in 21 cancer types [27]. To assess the clinical significance of OGEs, the PDAC patients were divided into high and low expression groups. The overall survival (OS) of the two groups was assessed by Kaplan-Meier plots and log-rank P-value, log-rank *P-value< 0.05* was the cut-off criterion, and the number-at-risk is visualized below the curves.

### 2.9. Exploration of the mRNA, Protein and Methylation Levels of OGEs

To further confirm the mRNA and protein level of OGEs in PDAC, we examined the relative mRNA expression of there genes in the UALCAN and Oncomine database. In addition, the protein expression and distribution of OGEs were investigated in PC tissues and compared normal tissues in The Human Protein Atlas (HPA, version: 18.1) database (https://www.proteinatlas.org/), which aim to map all the human proteins in cells, tissues, and organs [28]. UALCAN (http://ualcan.path.uab.edu) is a comprehensive, user-friendly, and interactive web resource to allow users to identify biomarkers in silico validation of potential genes of interest [29]. Oncomine 4.5 (https://www.oncomine.org/), a cancer microarray database and online data-mining platform, contributes to discovering novel genes associated with the progression of tumors [30]. In this study, we estimated the mRNA relative expression of OGEs in the UALCAN and Oncomine database, and P < 0.05 and a fold change of 2 were considered as statistically significant.

Furthermore, the human disease methylation database, DiseaseMeth version 2.0 (http://bio-bigdata.hrbmu.edu.cn/diseasemeth/), a web-based resource focused on the aberrant methylomes of human diseases [31], was utilized to compare methylation levels of OGEs between the pancreatic adenocarcinoma and paraneoplastic control tissues. Additionally, we also investigated the relationship between OGEs expression levels and their DNA methylation status using MEXPRESS (http://mexpress.be) [32], an online tool contains the latest TCGA data, clinical data and so on.

### 2.10. GSEA

GSEA, a computational method derives its power by centering on gene sets, that is, groups of genes that share similar biological characteristics, which performs biological information from a new perspective [33]. In data set GSE32676, samples of PDAC were separated into high expression groups and low expression groups based on the median expression of the overlapped up-regulated-genes, respectively. JAVA GSEA 3.0 (http://software.broadinstitute.org/gsea/index.jsp) was used in the present study to conduct GSEA. The biologically defined gene sets “c2.cp.kegg.v6.2.symbols.gmt”, was used as the reference gene.

### 2.11. Statistical Analysis

Overall survival curves were calculated by the Kaplan-Meier method and analyzed by the log-rank test. The WGCNA was carried out with “WGCNA.” The R 3.5.1 (64-bit) was used in this study. A *P < 0.05* was considered statistically significant.

## 3. Results

### 3.1. Screening of the Active Ingredients in QYHJ

**Figure 1:**
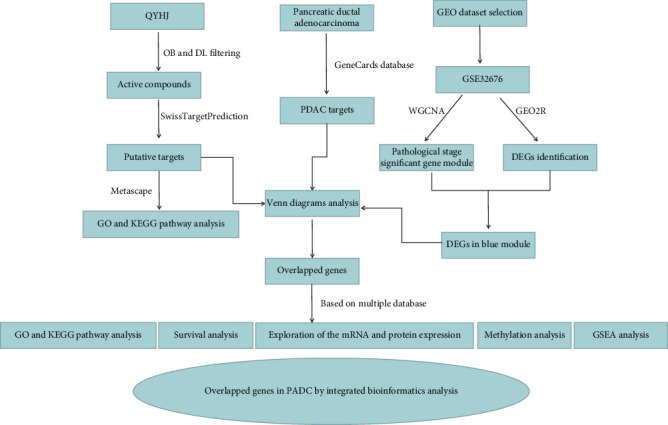
The flow diagram of network pharmacology and prognostic signatures analysis of QYHJ related targets. ADME properties of QYHJ were firstly assessed, and then potential therapeutic targets of QYHJ against PDAC were identified through identifying OGEs with WGCNA. Next, the GO and KEGG pathways enrichment analysis, prognostic value, expression levels, and methylation of OGEs were evaluated with multiple databases.

In the current study, we have conducted a multi-dimensional analysis to identify potential biomarkers of PDAC and targets of QYHJ by integrated bioinformatics methods in PDAC. The flow chart of our analysis is displayed in Figure 1. First, 123, 94, 37, 202, and 71 compounds of five medicinal herbs, RAE, HSB, HBHY, HSRGP, and FAR in QYHJ, respectively, were retrieved from TCMSP. Based on the screening criteria, there are 6 active components in RAE, 29 active components in HSB, 7 active components in HBHY, 15 active components in HSRGP, and 10 active components in FAR, respectively. The specific ADME parameters and the SMILES information of aforementioned the 67 selected active ingredients are shown in Table 3S.

### 3.2. Putative Targets Prediction in QYHJ

Putative targets were predicted by a combination of SwissTargetPrediction and STITCH servers. Consequently, We identified 220 candidate targets in RAE, 342 candidate targets in HSB, 309 candidate targets in HBHY, 302 candidate targets in HSRGP, and 446 candidate targets in FAR, respectively. Next, a total of 675 putative targets were collected from 67 compounds after eliminating the redundancy, which was objects of the study for subsequent analysis and visualized as the ingredients-targets network by Cytoscape (Figure S1).

### 3.3. Functional Enrichment Analysis of Putative Targets

**Figure 2:**
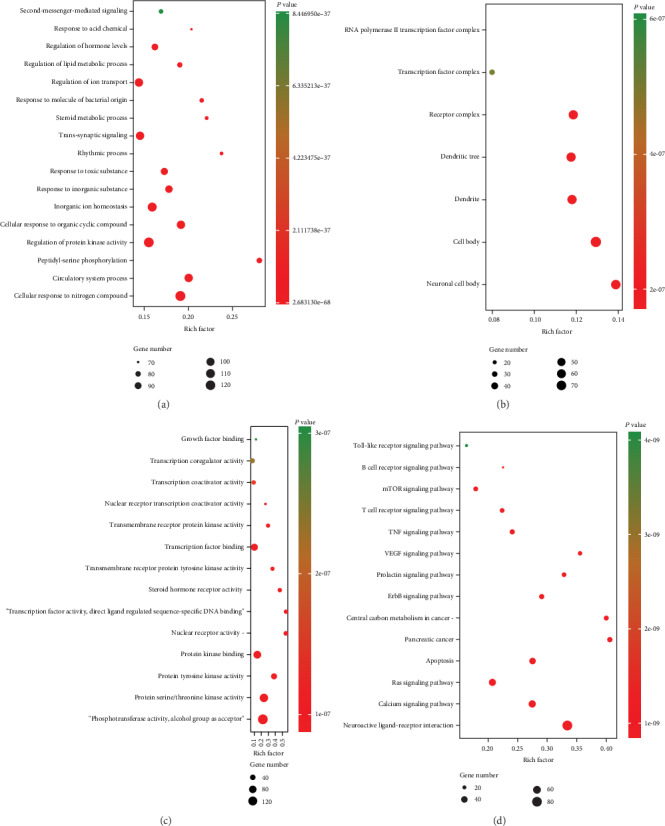
Functional annotation of active ingredients related to putative targets. (a) Enriched biological processes. (b) Enriched cellular components. (c) Enriched molecular functions. (d) Enriched KEGG pathways.

To explore the 675 putative targets of QYHJ, GO terms and KEGG pathway enrichment analysis was performed by Metascape. The GO enrichment analysis consists of biological process (BP), cellular component (CC), and molecular function (MF). GO functional biology processes include cellular response to nitrogen compound, circulatory system process, regulation of protein kinase activity, and so on (Figure 2(a)). According to the results in Figure 2(b), the putative targets were most significantly enriched in the neuronal cell body, dendrite, receptor complex of GO cellular component. Phosphotransferase activity-alcohol group as acceptor, protein serine/threonine kinase activity, and transcription factor binding of molecular function (Figure 2(c)). Additionally, KEGG pathways of the putative targets were analyzed and shown in Figure 2(d), including Neuroactive ligand-receptor interaction, Calcium signaling pathway, Apoptosis, Pancreatic cancer and Central carbon metabolism in cancer, etc. These signaling pathways involved with signal transduction, metabolism, and angiogenesis. In general, these biological processes and signaling pathways, which probably related to the beneficial effects of QYHJ against PC.

### 3.4. Detecting Dataset Quality of GSE32676 and Clinical Data

**Figure 3:**
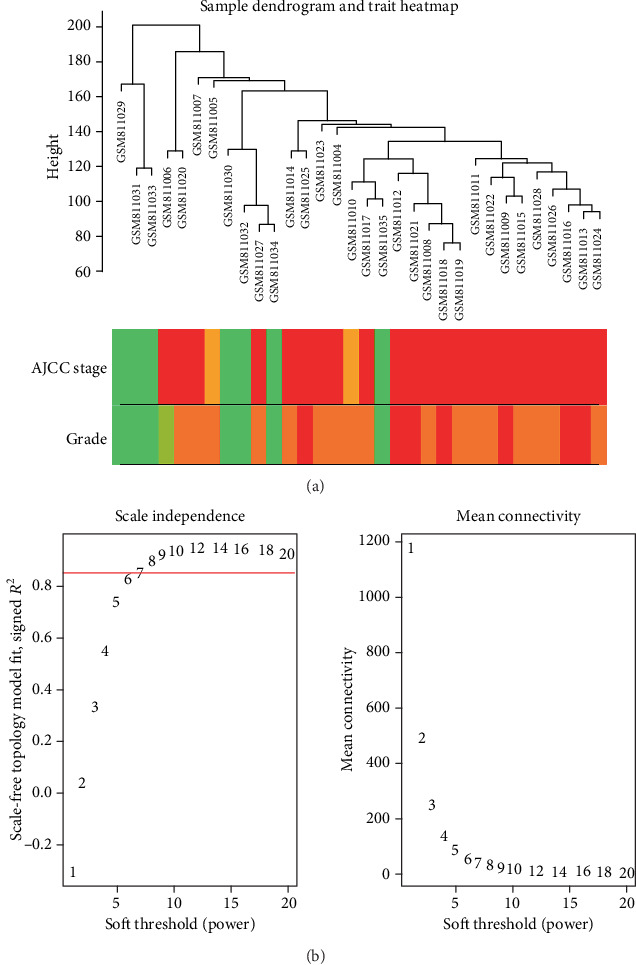
Clustering dendrogram and determination of soft-thresholding power in the WGCNA. (a) Clustering dendrogram of 32 samples. (b) Analysis of the scale-free fitting indices for various soft-thresholding powers (*β*), and mean connectivity analysis of various soft-thresholding powers.

WGCNA was conducted on all genes of 32 samples in GSE32676 (Figure 3). After checking the quality by WGCNA R package, there were no samplers removed in the sample clustering (Figure 3(a)), and we could find 2 types of pathological tumor stage and histological grade of patients with PDAC. Besides, to ensure a scale-free network, a soft threshold power of *β* = 8 (scale-free R^2^ = 0.85) was selected (Figure 3(b)).

### 3.5. Identification of the Key Module

**Figure 4:**
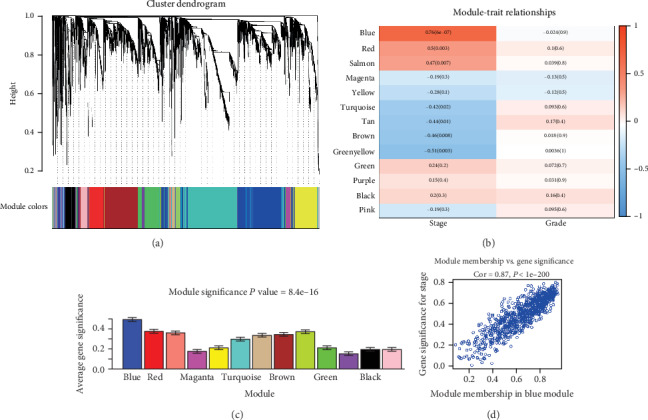
Identification of relevant modules of pancreatic cancer clinical traits. (a) Clustering dendrograms of genes based on dissimilarity topological overlap and module colours. (b) Heatmap of the correlation between module eigengenes and clinical traits of pancreatic cancer. (c) Distribution of average gene significance in the modules connected with the clinicopathological stages of pancreatic cancer. (d) Scatter plot of module eigengenes in the blue module.

After merging similar clusters, we eventually identified 13 modules that contained similar gene patterns and non-clustering genes shown in gray (Figure 4(a)). From the heat map of module–trait relationships, we found that the blue module was the most highly correlated with the clinical-pathological stage (r = 0.76, P = 6e-7) by using Pearson's correlation analysis, and contained a total of 928 genes, shown in Figure 4(b). Also, modules with a greater MS were significantly connected with the development of the disease.  Figure 4(c) displays the significance level of 13 co-expression modules associated with the pathological stage. The MS of the blue module was significantly higher than any other module. Moreover, scatterplots of GS for stage vs. MM in the blue module was plotted (Figure 4(d)). The *P-value* less than 1e-200 indicated that they were highly correlated. Hence, the blue module was chosen for subsequent analyses.

### 3.6. Identification and PPI Network Construction of OGEs

**Figure 5:**
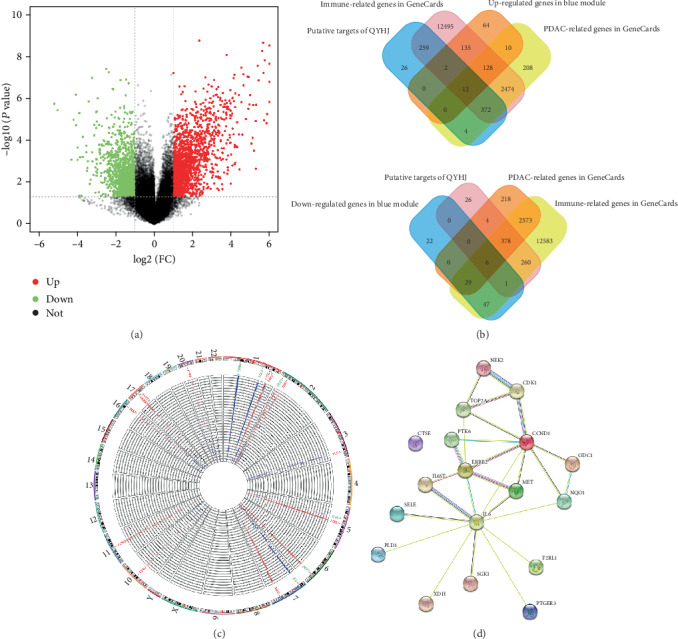
Identification of OGEs and construction of the PPI network. (a) The volcano plot of differentially expressed genes between cancer and normal samples. The *x*-axis shows the gene expression difference by a logtransformed fold change while the *y*-axis shows significance by–log10 transformed *P* value value. The red dot represents the up-regulated gene, while the blue dot represents the down-regulated gene. (b) Identification of immunity-related genes of PDAC with Venn diagram analysis. (c) Circular visualization of expression patterns, and chromosomal positions of OGEs. The 32 samples microarray datasets in GSE32676 are represented in the inner circular heatmaps. Red represents up-regulation, and blue represents down-regulation. Chromosomes were located in the outer circle, lines connected with a specific gene and its corresponding chromosomal locations. The 12 up-regulated and 6 down-regulated genes shown in red and green, respectively. (d) PPI network of OGEs.

We used the GEO2R tool to identify DEGs between 25 PDAC tissue samples and 7 control samples from the GEO database, and screened out 1151 up-regulated and 851 down-regulated DEGs, as shown in the volcano plot in Figure 5(a). In addition, we used the Venn diagram web-tool to cross the putative, PDAC, immune, and blue module targets and found 18 overlapped DEGs (Figure 5(b)), including 12 up-regulated OGEs and 6 down-regulated OGEs. As shown in Figure 5(c), the 18 OGEs were also visualized the expression patterns of the 32 sample datasets included in the present study, as well as its chromosomal locations. The up-regulated genes CDK1, PLD1, MET, F2RL1, XDH, NEK2, TOP2A, NQO1, CCND1, PTK6, CTSE, ERBB2 were located in chromosomes 1, 2, 3, 5, 7, 10, 11, 16, 17, and 20. The down-regulated genes PTGER3, SGK1, ODC1, SELE, IL6ST, and IL6 were distributed in chromosomes 1, 2, 5, 6, and 7. Protein interactions among OGEs were predicted with STRING tools. As a result, the 18 immunity-related targets of PDAC were mostly connected, suggesting that QYHJ has a regulating effect on gene network at a whole molecular level in PC (Figure 5(d)).

### 3.7. Functional Enrichment Analysis of OGEs

**Figure 6:**
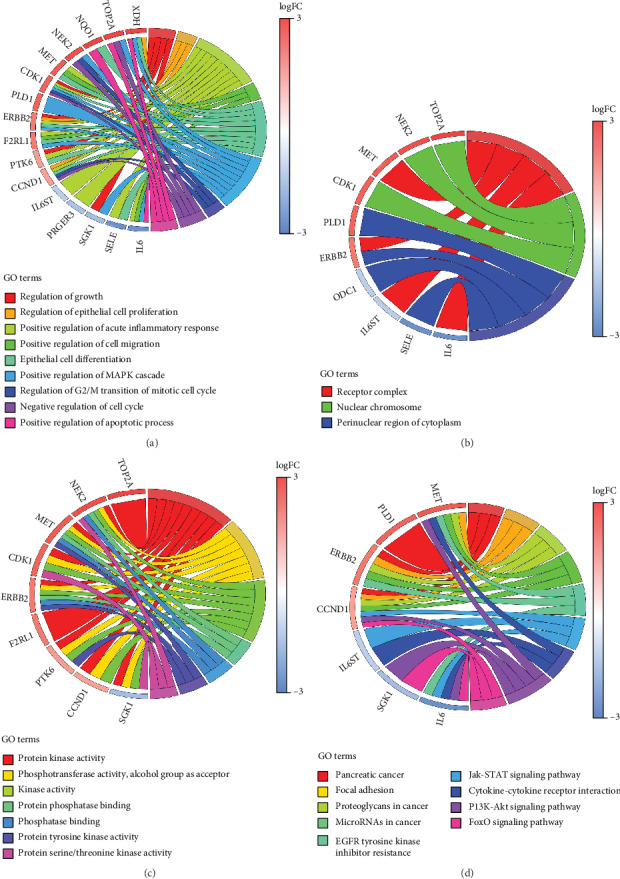
Functional enrichment analysis of 18 overlapped DEGs. (a) Chord plot of biological process. (b) Chord plot of cellular component (c) Chord plot of molecular function. (d) Chord plot of KEGG pathways. DEGs:differentially expressed genes.

To further gain a more in-depth comprehension of the biological behaviors of the OGEs, functional enrichment analysis was performed once more. The GO analysis revealed that the OGEs were significantly related to several BP, including regulation of growth, regulation of epithelial cell proliferation and differentiation, positive regulation of cell migration and apoptotic process, negative regulation of cell cycle (Figure 6(a)). CC analysis showed that the OGEs were mainly enriched in the receptor complex, nuclear chromosome, and perinuclear region of cytoplasm (Figure 6(b)). MF analysis suggested that the OGEs were significantly associated with protein kinase activity, protein phosphatase binding, etc. (Figure 6(c)). The results suggested that the OGEs were mostly involved in pancreatic cancer, focal adhesion, PI3K-Akt signaling pathway, and so on (Figure 6(d)).

### 3.8. Survival Analysis of OGEs in Patients with PDAC

**Figure 7:**
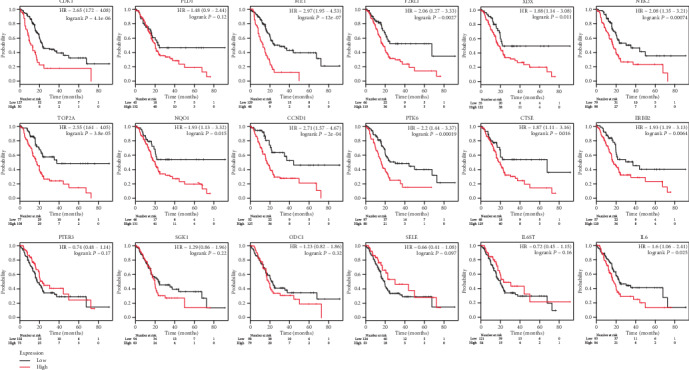
The prognostic value of 18 overlapped DEGs in PDAC patients. The overall survival rate of CDK1 (a), PLD1 (b), MET (c), F2RL1 (d), XDH (e), NEK2 (f), TOP2A (g), NQO1 (h), CCND1 (i), PTK6 (j), CTSE (k), ERBB2 (l), PTER3 (m), SGK1 (n), ODC1 (o), SELE (p), IL6ST (q), and IL (r).

To further clarify the prognostic values of OGEs, the OS of PDAC patients was analyzed by the Kaplan-Meier plotter. As suggested in Figures 7(a)-7(r), we found that the high mRNA expression of CDK1, PLD1, MET, F2RL1, XDH, NEK2, TOP2A, NQO1, CCND1, PTK6, CTSE, ERBB2, and IL6 was associated with poor overall survival rate, whlie the other genes had no statistical influence on patients' OS. Additionally, the poor prognosis gene could be complicatedly connected to multiple active ingredients of various herbs (Table 4S) based on the ingredients-targets network, which indicated that these ingredients might synergistically help to the pharmacological action of the QYHJ for the treatment of PDAC. Collectively, these findings concluded that CDK1, PLD1, MET, F2RL1, XDH, NEK2, TOP2A, NQO1, CCND1, PTK6, CTSE, ERBB2 is closely related to overall survival, which might be vital biomarkers for the prognosis of PDAC.

### 3.9. Validation of up-Regulated Overlapping Genes Expression Levels in Numerous Databases

**Figure 8:**
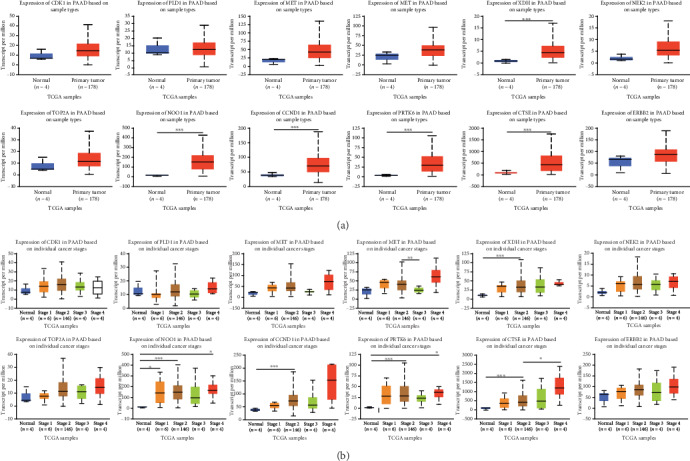
The mRNA expression levels of 12 overlapped DEGs in PDAC patients. (a) CDK1, PLD1, MET, F2RL1, XDH, NEK2, TOP2A, NQO1, CCND1, PTK6, CTSE, ERBB2 expression differences between PDAC and normal tissues. (b) Expression of CDK1, PLD1, MET, F2RL1, XDH, NEK2, TOP2A, NQO1, CCND1, PTK6, CTSE, ERBB2 in PDAC tissues with different T stages.

**Figure 9:**
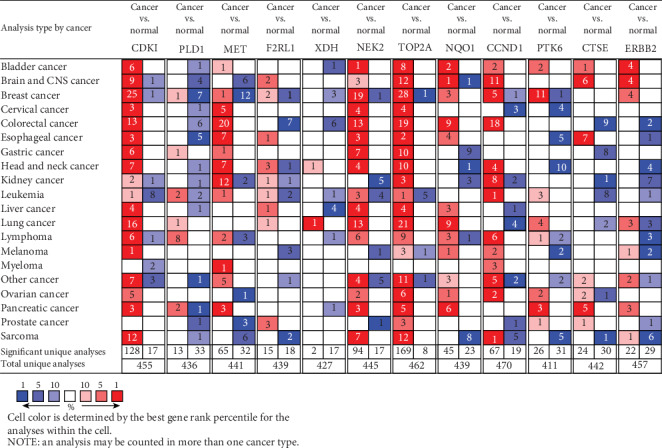
An overview of mRNA levels of 12 overlapped DEGs in 20 different types of cancers based on Oncomine. The numbers in colored rectangle represent the quantities of datasets with statistically significant. P-value: 0.05, fold change: 1.5, gene rank: 10%, and data type: mRNA was set as the threshold.

**Figure 10:**
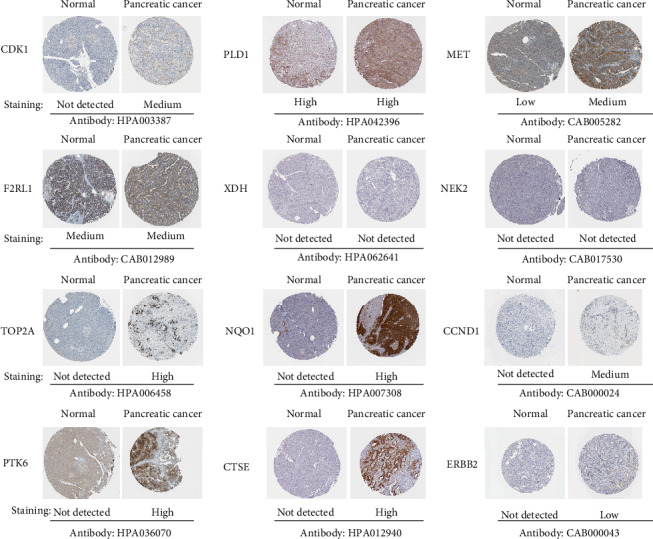
Representative immunohistochemistry images of 12 overlapped DEGs in PDAC tissues and normal pancreatic tissue based on Human Protein Atlas. CDK1, TOP2A, NQO1, CCND1, PTK6, CTSE, and ERBB2 proteins were not expressed in control pancreatic tissues; however, their low, medium, and high expressions were detected in PDAC tissues. Low protein expressions of MET was observed in normal pancreatic tissues, whereas there was a medium expression of MET in pancreatic cancer tissues. Significantly, the high, medium expression of PLD1 and F2RL1, respectively, were found in both tissues. Also, we found that XDH and NEK2 were not expressed in both cancer and normal tissues.

According to the results of survival analysis, we further confirmed the transcription level of up-regulated overlapping genes between primary tumor tissues and normal tissues in multiple databases in UALCAN and Oncomine. It was noted that all of them were significantly up-regulated in part, in PDAC samples compared with normal controls in the TCGA dataset with sample types (Figure 8(a)), and individual cancer stages (Figure 8(b)). Furthermore, an overview of up-regulated overlapping genes in all sorts of tumors showed that up-regulated overlapping genes, except XDH, were observably overexpressed in pancreatic cancer (Figure 9). After studying the mRNA expression patterns of up-regulated overlapping genes in PDAC, the protein level was also investigated through the HPA database. As was shown in Figure 10, the protein expression of up-regulated overlapping genes essentially in agreement with transcriptional expression, with most genes have a medium or high protein expression in pancreatic cancer tissues. Taken together, our results showed that these up-regulated overlapping genes might play a central role in the onset and development of PDAC.

### 3.10. Relationship between Expression of up-Regulated Overlapping Genes and Methylation

**Figure 11:**
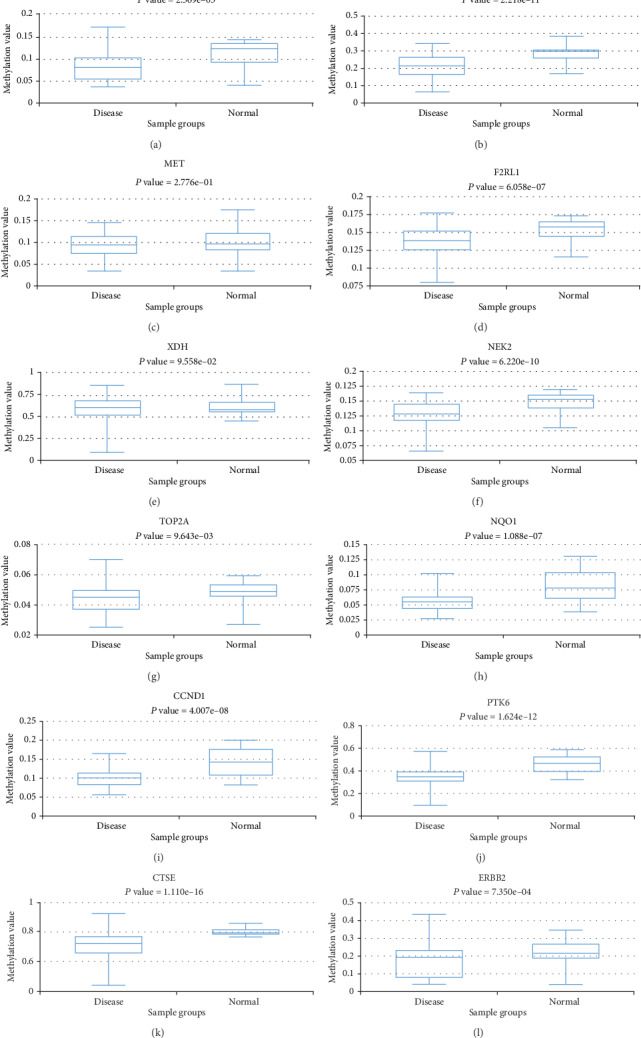
Methylation analyses of 12 overlapped DEGs. The methylation levels of (a) CDK1, (b) PLD1, (c) MET, (d) F2RL1, (e) XDH, (f) NEK2, (g) TOP2A, (h) NQO1, (i) CCND1, (j) PTK6, (k) CTSE, and (l) ERBB2 PDAC and corresponding normal tissues were explored using DiseaseMeth 2.0.

We investigated the relationship between the methylation status and the expression of up-regulated overlapping genes to clarify underlying mechanisms of aberrant elevation in PDAC tissues. The result from DiseaseMeth version 2.0 showed that the CDK1, PLD1, F2RL1, NEK2, TOP2A, NQO1, CCND1, PTK6, CTSE, and ERBB2 mean methylation levels were all obstinately high in disease state compared with normal tissues. Meanwhile, the mean methylation levels of MET and XDH showed no statistical difference (Figure 11). Then, as we observed from MEXPRESS analysis, multiple methylation sites in the DNA sequences of up-regulated overlapping genes that were negatively related to itself expression. (Figure S2).

### 3.11. Up-Regulated Overlapping Genes GSEA Analysis

**Figure 12:**
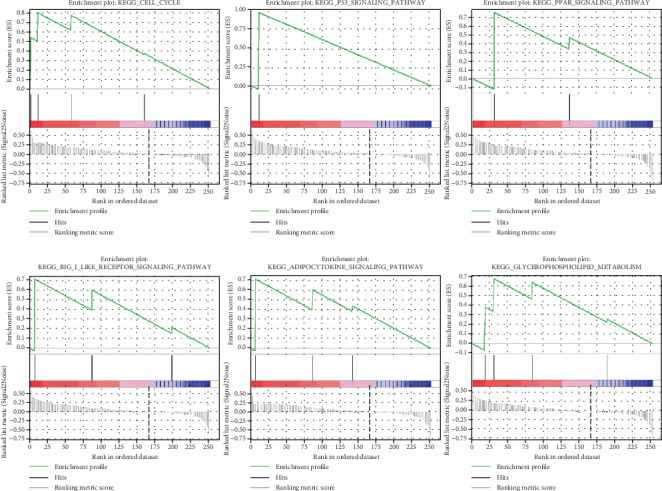
Gene set enrichment analysis (GSEA) using GSE32676. The six representative common KEGG pathway sets enriched in pancreatic cancer samples with a high expression of 12 overlapped DEGs.

To further elucidate the potential mechanisms of up-regulated overlapping genes in PC, GSEA was performed to explore prominent KEGG pathways between the highly-expressed and lowly-expressed groups. As shown in Figure 12, GSEA analysis suggests a high expression of overlapping genes is enriched in “cell cycle,” “P53 signaling pathway”, “PPAR signaling pathway,” “RIG I like receptor signaling pathway,” adipocytokine signaling pathway,” and “glycerophospholipid metabolism.”

## 4. Discussion

Pancreatic ductal adenocarcinoma (PDAC) is considered as a lethal disease, characterized by highly invasive and chemo-resistant. Chemotherapeutics combined with Chinese herbal formulations not only significantly reduce the cancer recurrence rate, alleviate the side effects caused by chemotherapy drugs, and contribute to improving patient survival of postoperative cancer patients [34, 35]. Although we found that QYHJ significantly inhibited the proliferation, migration and promoted apoptosis of CFPAC-1 cells in vitro and effectively reversed gemcitabine resistance by regulating the lncRNA AB209630/miR-373/EphB2-NANOG signals [12], underlying molecular mechanisms and biomarkers are not fully elucidated. In this study, network pharmacology methods were used to identify bioactive ingredients in QYHJ and biological functions regulated by these ingredients based on their OB, DL, and HL. We employed the TCMSP database to acquire 527 potential ingredients present in QYHJ. After screening, 67 ingredients were selected to possess suitable OB and DL properties. To elucidate the potential biological molecular mechanism of QYHJ, and 675 putative targets were collected. Using the KEGG pathway enrichment analysis, we obtained various types of signaling pathways, including pancreatic cancer, apoptosis, and VEGF signaling pathways. These pathways may participate in the initiation and progression of PDAC.

With great advances that have been made in microarray and sequencing technology, massive information on genomics and proteomics has emerged. Additionally, the high-throughput platforms and new methods provide a novel strategy for medical oncology. WGCNA is a systems biology method that is used to correlate the modules to clinical traits by a soft-threshold algorithm. In the current study, we selected the GSE32676 dataset that is only containing tumor and adjacent normal samples from the GEO database and identified significant modules through WGCNA. Finally, thirteen modules were obtained from the WGCNA analysis. Among them, the blue module was most related to the tumor stage. After taking an intersection, we eventually obtained 18 immune-related targets with high functional significance of QYHJ were chosen as OGEs in the significant module. Chromosome mapping of the 18 OGEs shown chromosomes 1 contained most genes. Gross M et.al. demonstrated that the loci on chromosomes 1q32.1 map to NR5A2 was susceptibility loci for pancreatic cancer [36], which is has been identified as a susceptibility gene of pancreatic cancer [37]. These results suggest chromosome 1 plays a vital role in influencing the pathogenesis of PDAC.

The result of GO and KEGG analysis proved that the OGEs mainly associated with regulation of cell proliferation, differentiation, migration, apoptotic process, negative regulation of cell cycle and found that these OGEs are mainly concentrated upon pancreatic cancer, focal adhesion, PI3K-Akt signaling pathway, which suggests a potential mechanism for PDAC proliferation and metastasis. Here, we also performed a survival analysis to screen up-regulated OGEs with a significant difference. A total of 12 genes (CDK1, PLD1, MET, F2RL1, XDH, NEK2, TOP2A, NQO1, CCND1, PTK6, CTSE, ERBB2) were highly expressed and associated with the depressing prognosis. Further, we determined that CDK1, PLD1, MET, F2RL1, XDH, NEK2, TOP2A, NQO1, CCND1, PTK6, CTSE, ERBB2 were not only significantly upregulated in PDAC tissues but also correlated positively as well with TNM stage, suggesting that these genes may be potential immune-related biomarkers for prognosis prediction for PDAC patients.

Many studies have confirmed that cell cycle dysregulation plays a vital role in types of cancers. The cell cycle-related protein CDK1 belongs to the cyclin-dependent kinases (CDKs) family and promotes the development of cells from the G2 phase to the M phase [38]. To date, accumulating evidence has elucidated that CDK1 is significantly overexpressed and associated with poor outcome in gastric and lung cancers. Consistent with published data, a study by Piao J et.al. [39] found that the expression of CDK1 significantly increased in PDAC tissues and correlated with tumor size, histological grade, and poor outcomes. Fortunately, we also discovered a similar result, and CDK1 interacts with multiple ingredients for QYHJ. Hence, QYHJ might treat PDAN by regulating the expression of CDK1. PLD1, a central enzyme, was regulated lipid metabolism by catalyzing the hydrolysis of phosphatidylcholine. Several studies have indicated that PLD1 contributed to the invasion, metastasis, and angiogenesis of various human tumors [40]. Consistently, Hu and colleagues showed that PLD1 was increased in PDAC and directly associated with poor prognosis and vascular invasion [41]. Similarly, we also found MET, the receptor of hepatocyte growth factor, was highly-expressed and closely related to short survival time, consistent with the study by Zhou J et.al [3]. Most importantly, Lux A et.al [42]. Further elucidated that MET-positive patients showed a markedly unsatisfactory survival time, and it can be served as a negative prognostic factor. F2RL1 encoded the PAR2. The PAR2 is a G protein-coupled receptors, which highly expressed in the pancreas and contributed to accelerating tumor growth in pancreatic cancer [43]. Besides, Shi K et.al [44]. found that the orthotopically growing primary tumors were restricted by knocking down F2RL1. NEK2 was proved to be overexpressed in PDAC tissues and significantly related to histological differentiation, lymph node metastasis, and tumor stage [45]. High expression of TOP2A was significantly associated with tumor metastasis and shorter survival in pancreatic cancer patients, and knockdown of TOP2A inhibited proliferation and migration in Panc-1 and CaPan-1 cell lines [46]. It was showed that NQO1 overexpression may be identified as an independent prognostic biomarker in PDAC, and connected with the tumor-node-metastasis (TNM) stage [47]. Moreover, recent studies have revealed that CCND1 was up-regulated in pancreatic cancer tissues and validated as a direct binding target of miR-720/miR-584 to inhibit cell proliferation and invasion [48, 49]. Besides, silencing PTK6 significantly attenuated cellular migration and invasion in pancreatic cancer [50]. And CTSE may be a novel marker for a definitive diagnosis of PDAC with a higher detection frequency [51]. Meanwhile, oncogenic ERBB2 combined with KRAS mutations synergistically improve the progression of PDAC [52]. As for the XDH gene, it was not reported to participate in the development of PDAC. Further study should be needed. Our findings once again suggested that these genes considered to be good for the diagnosis of PDAC.

Further, we also found that CDK1, PLD1, F2RL1, NEK2, TOP2A, NQO1, CCND1, PTK6, CTSE, and ERBB2 were hypomethylated in PDAC samples compared with homologous normal samples, which could be the potential mechanism for the adamant up-regulation of these 10 genes in PDAC. To further investigate the up-regulated overlapping gene's biological events, we performed the GSEA for each gene. The results of GSEA suggested that the Cell cycle, P53, and RIG I like receptor signaling pathways were associated with the high-expression samples of these genes, indicating the genes have an important role in PDAC progress.

## 5. Conclusions

The present study assessed underlying immune-related targets of QYHJ by combining WGCNA and bioinformatics, and we identified multiple strongly up-regulated genes (CDK1, PLD1, MET, F2RL1, XDH, NEK2, TOP2A, NQO1, CCND1, PTK6, CTSE, ERBB2) associated with the poor prognosis of PDAC due to its DNA hypomethylation level. However, further studies need to be done in PDAC cells to assess their clinical value as biomarkers and therapeutic targets accurately.

## Data Availability

The datasets analyzed during the current study available from the corresponding author on reasonable request.

## Supplementary Materials

Supplementary Materials Table 1S. The PDAC genes in the GeneCards database Supplementary file 2: Table 2S. The immune-related targets in the GeneCards database Supplementary file 3: Table 3S. The detailed ADME parameters and the SMILES information of the aforementioned 67 selected compounds. Supplementary file 4: Figure S1. The potential active ingredients-target network of QYHJ. Supplementary file 5: Table 4S. The association between active ingredients and up-regulated overlapping genes. Supplementary file 6: Figure S2. Association of Methylation sites with the expression of 12 overlapped DEGs.
