# Supplementary figures and images for: Integrated Analyses Identify Immune-Related Signature Associated with Qingyihuaji Formula for Treatment of Pancreatic Ductal Adenocarcinoma Using Network Pharmacology and Weighted Gene Co-Expression Network
Source: J Immunol Res. 2020 May 20;2020:7503605. doi: 10.1155/2020/7503605 (PMC7256764; doi:10.1155/2020/7503605)

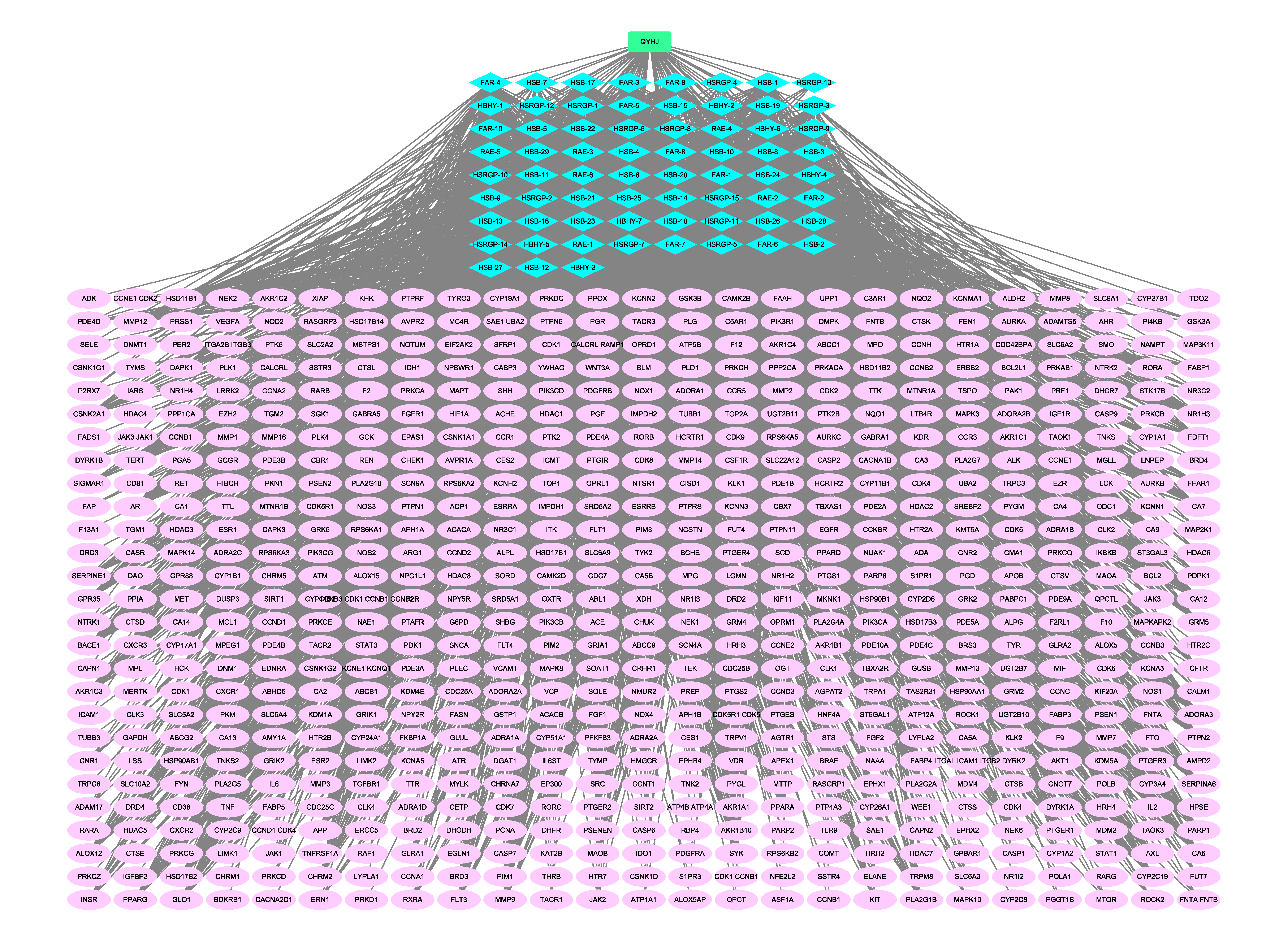

Supplement: Supplementary Materials — Table 1S. The PDAC genes in the GeneCards database Supplementary file 2: Table 2S. The immune-related targets in the GeneCards database Supplementary file 3: Table 3S. The detailed ADME parameters and the SMILES information of the aforementioned 67 selected compounds. Supplementary file 4: Figure S1. The potential active ingredients-target network of QYHJ. Supplementary file 5: Table 4S. The association between active ingredients and up-regulated overlapping genes. Supplementary file 6: Figure S2. Association of Methylation sites with the expression of 12 overlapped DEGs. [file 7503605.f1.zip › Supplementary Figure S1.tif]

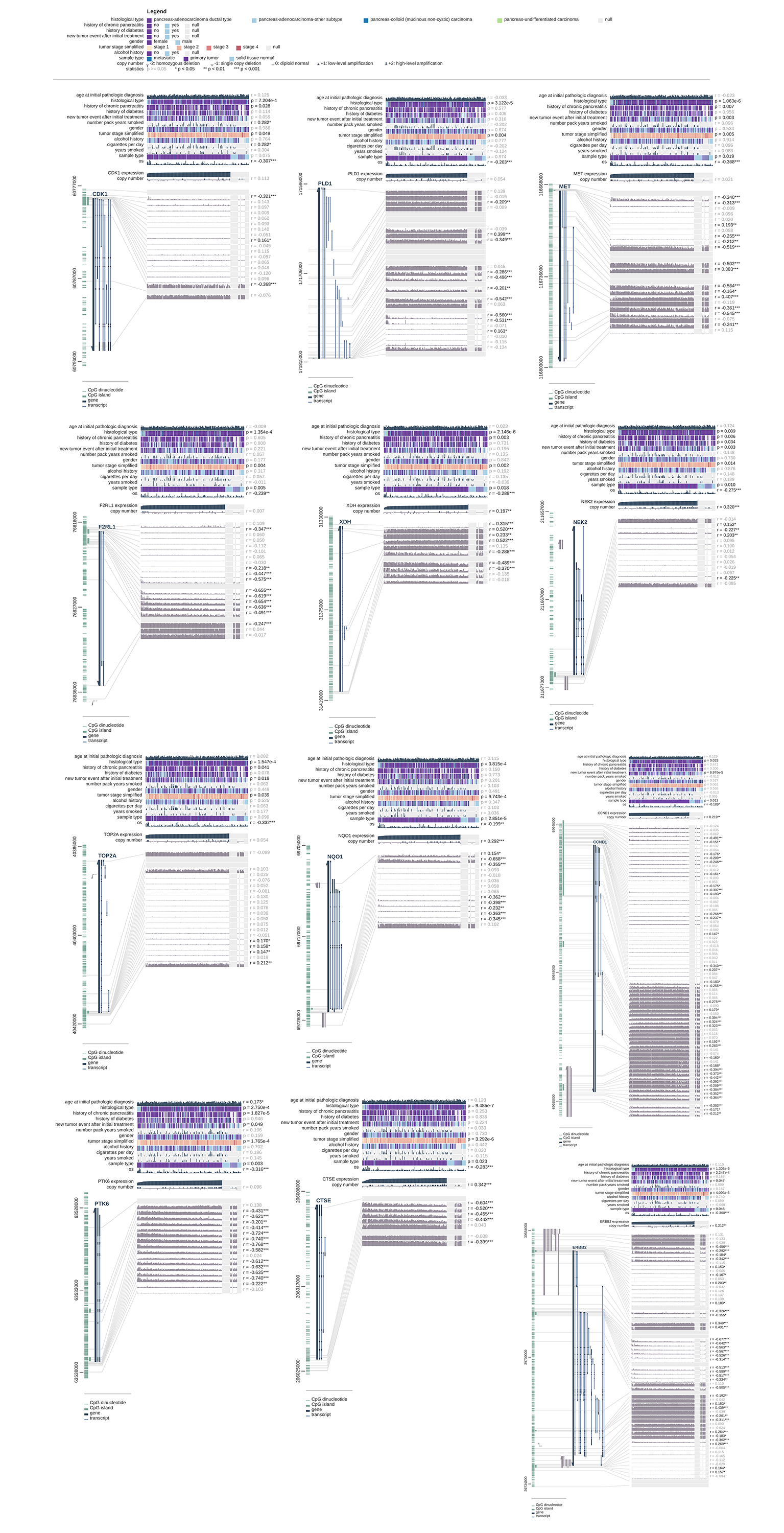

Supplement: Supplementary Materials — Table 1S. The PDAC genes in the GeneCards database Supplementary file 2: Table 2S. The immune-related targets in the GeneCards database Supplementary file 3: Table 3S. The detailed ADME parameters and the SMILES information of the aforementioned 67 selected compounds. Supplementary file 4: Figure S1. The potential active ingredients-target network of QYHJ. Supplementary file 5: Table 4S. The association between active ingredients and up-regulated overlapping genes. Supplementary file 6: Figure S2. Association of Methylation sites with the expression of 12 overlapped DEGs. [file 7503605.f1.zip › Supplementary Figure S2.tif]
