# Supplementary material for: Integrated Analyses Identify Immune-Related Signature Associated with Qingyihuaji Formula for Treatment of Pancreatic Ductal Adenocarcinoma Using Network Pharmacology and Weighted Gene Co-Expression Network
Source: J Immunol Res. 2020 May 20;2020:7503605. doi: 10.1155/2020/7503605 (PMC7256764; doi:10.1155/2020/7503605)
Supplement: Supplementary Materials — Table 1S. The PDAC genes in the GeneCards database Supplementary file 2: Table 2S. The immune-related targets in the GeneCards database Supplementary file 3: Table 3S. The detailed ADME parameters and the SMILES information of the aforementioned 67 selected compounds. Supplementary file 4: Figure S1. The potential active ingredients-target network of QYHJ. Supplementary file 5: Table 4S. The association between active ingredients and up-regulated overlapping genes. Supplementary file 6: Figure S2. Association of Methylation sites with the expression of 12 overlapped DEGs. [file 7503605.f1.zip › Supplementary file Table 1S.doc]

**Supplementary Table 1 The PDAC genes in the GeneCards database**

| Gene Symbol | Description |
| --- | --- |
| TP53 | Tumor Protein P53 |
| CDKN2A | Cyclin Dependent Kinase Inhibitor 2A |
| KRAS | KRAS Proto-Oncogene, GTPase |
| EGFR | Epidermal Growth Factor Receptor |
| ERBB2 | Erb-B2 Receptor Tyrosine Kinase 2 |
| SMAD4 | SMAD Family Member 4 |
| BRCA2 | BRCA2 DNA Repair Associated |
| BRCA1 | BRCA1 DNA Repair Associated |
| CTNNB1 | Catenin Beta 1 |
| CDH1 | Cadherin 1 |
| PIK3CA | Phosphatidylinositol-4,5-Bisphosphate 3-Kinase Catalytic Subunit Alpha |
| HRAS | HRas Proto-Oncogene, GTPase |
| PTEN | Phosphatase And Tensin Homolog |
| SPINK1 | Serine Peptidase Inhibitor Kazal Type 1 |
| CCND1 | Cyclin D1 |
| CFTR | CF Transmembrane Conductance Regulator |
| PRSS1 | Serine Protease 1 |
| AKT1 | AKT Serine/Threonine Kinase 1 |
| INS | Insulin |
| TGFB1 | Transforming Growth Factor Beta 1 |
| IL6 | Interleukin 6 |
| MLH1 | MutL Homolog 1 |
| CDKN1A | Cyclin Dependent Kinase Inhibitor 1A |
| PALLD | Palladin, Cytoskeletal Associated Protein |
| STAT3 | Signal Transducer And Activator Of Transcription 3 |
| BRAF | B-Raf Proto-Oncogene, Serine/Threonine Kinase |
| PDX1 | Pancreatic And Duodenal Homeobox 1 |
| CDKN1B | Cyclin Dependent Kinase Inhibitor 1B |
| MET | MET Proto-Oncogene, Receptor Tyrosine Kinase |
| MIR21 | MicroRNA 21 |
| TGFBR2 | Transforming Growth Factor Beta Receptor 2 |
| STK11 | Serine/Threonine Kinase 11 |
| ESR1 | Estrogen Receptor 1 |
| BAX | BCL2 Associated X, Apoptosis Regulator |
| TERT | Telomerase Reverse Transcriptase |
| MSH2 | MutS Homolog 2 |
| VEGFA | Vascular Endothelial Growth Factor A |
| MTOR | Mechanistic Target Of Rapamycin Kinase |
| MUC1 | Mucin 1, Cell Surface Associated |
| RB1 | RB Transcriptional Corepressor 1 |
| FAS | Fas Cell Surface Death Receptor |
| ATM | ATM Serine/Threonine Kinase |
| SRC | SRC Proto-Oncogene, Non-Receptor Tyrosine Kinase |
| PALB2 | Partner And Localizer Of BRCA2 |
| KRT7 | Keratin 7 |
| FGFR2 | Fibroblast Growth Factor Receptor 2 |
| EPCAM | Epithelial Cell Adhesion Molecule |
| RET | Ret Proto-Oncogene |
| FGFR1 | Fibroblast Growth Factor Receptor 1 |
| APC | APC Regulator Of WNT Signaling Pathway |
| CDK4 | Cyclin Dependent Kinase 4 |
| MEN1 | Menin 1 |
| CAV1 | Caveolin 1 |
| CASP8 | Caspase 8 |
| AKT2 | AKT Serine/Threonine Kinase 2 |
| MIR96 | MicroRNA 96 |
| PPARG | Peroxisome Proliferator Activated Receptor Gamma |
| FHIT | Fragile Histidine Triad Diadenosine Triphosphatase |
| GNAS | GNAS Complex Locus |
| H19 | H19 Imprinted Maternally Expressed Transcript |
| MIR125A | MicroRNA 125a |
| MIR145 | MicroRNA 145 |
| MIR483 | MicroRNA 483 |
| RAF1 | Raf-1 Proto-Oncogene, Serine/Threonine Kinase |
| MAP2K1 | Mitogen-Activated Protein Kinase Kinase 1 |
| AURKA | Aurora Kinase A |
| PTGS2 | Prostaglandin-Endoperoxide Synthase 2 |
| MIR17 | MicroRNA 17 |
| KRT20 | Keratin 20 |
| EGF | Epidermal Growth Factor |
| MST1R | Macrophage Stimulating 1 Receptor |
| MIR34A | MicroRNA 34a |
| MIR143 | MicroRNA 143 |
| FASLG | Fas Ligand |
| CEL | Carboxyl Ester Lipase |
| BCL2 | BCL2 Apoptosis Regulator |
| MIR10B | MicroRNA 10b |
| MIR200A | MicroRNA 200a |
| MIR200C | MicroRNA 200c |
| MIR155 | MicroRNA 155 |
| MMP9 | Matrix Metallopeptidase 9 |
| FGFR4 | Fibroblast Growth Factor Receptor 4 |
| CPA1 | Carboxypeptidase A1 |
| IL10 | Interleukin 10 |
| MIR141 | MicroRNA 141 |
| CHGA | Chromogranin A |
| MDM2 | MDM2 Proto-Oncogene |
| MIR148A | MicroRNA 148a |
| RAD51 | RAD51 Recombinase |
| IGF2 | Insulin Like Growth Factor 2 |
| MAP2K2 | Mitogen-Activated Protein Kinase Kinase 2 |
| CEACAM5 | CEA Cell Adhesion Molecule 5 |
| HIF1A | Hypoxia Inducible Factor 1 Subunit Alpha |
| MIR205 | MicroRNA 205 |
| MIR221 | MicroRNA 221 |
| MYC | MYC Proto-Oncogene, BHLH Transcription Factor |
| MIR126 | MicroRNA 126 |
| MMP2 | Matrix Metallopeptidase 2 |
| CDX2 | Caudal Type Homeobox 2 |
| KIT | KIT Proto-Oncogene, Receptor Tyrosine Kinase |
| MUC5AC | Mucin 5AC, Oligomeric Mucus/Gel-Forming |
| SST | Somatostatin |
| MIR210 | MicroRNA 210 |
| MIR203A | MicroRNA 203a |
| MIR182 | MicroRNA 182 |
| KRT19 | Keratin 19 |
| MIR222 | MicroRNA 222 |
| MIR128-2 | MicroRNA 128-2 |
| MIR27A | MicroRNA 27a |
| EP300 | E1A Binding Protein P300 |
| MIR146A | MicroRNA 146a |
| SMAD7 | SMAD Family Member 7 |
| MSH6 | MutS Homolog 6 |
| PGR | Progesterone Receptor |
| TERC | Telomerase RNA Component |
| CD44 | CD44 Molecule (Indian Blood Group) |
| MUC4 | Mucin 4, Cell Surface Associated |
| MIR375 | MicroRNA 375 |
| BIRC5 | Baculoviral IAP Repeat Containing 5 |
| MIR15A | MicroRNA 15a |
| CXCL8 | C-X-C Motif Chemokine Ligand 8 |
| MIR195 | MicroRNA 195 |
| SCT | Secretin |
| CTNNA1 | Catenin Alpha 1 |
| MAPK1 | Mitogen-Activated Protein Kinase 1 |
| MIR223 | MicroRNA 223 |
| CASP3 | Caspase 3 |
| GAST | Gastrin |
| MIR20A | MicroRNA 20a |
| HNF1B | HNF1 Homeobox B |
| MIR25 | MicroRNA 25 |
| TNF | Tumor Necrosis Factor |
| BCL2L1 | BCL2 Like 1 |
| MIRLET7D | MicroRNA Let-7d |
| MIRLET7A1 | MicroRNA Let-7a-1 |
| CD274 | CD274 Molecule |
| HMGA1 | High Mobility Group AT-Hook 1 |
| CCK | Cholecystokinin |
| IGF1R | Insulin Like Growth Factor 1 Receptor |
| TGFA | Transforming Growth Factor Alpha |
| MIR214 | MicroRNA 214 |
| MIR100 | MicroRNA 100 |
| MKI67 | Marker Of Proliferation Ki-67 |
| MIR150 | MicroRNA 150 |
| MIR23B | MicroRNA 23b |
| FGF2 | Fibroblast Growth Factor 2 |
| MUC2 | Mucin 2, Oligomeric Mucus/Gel-Forming |
| MIR10A | MicroRNA 10a |
| BUB1 | BUB1 Mitotic Checkpoint Serine/Threonine Kinase |
| MSLN | Mesothelin |
| MIR16-1 | MicroRNA 16-1 |
| MIR26A1 | MicroRNA 26a-1 |
| NOTCH1 | Notch Receptor 1 |
| ERBB3 | Erb-B2 Receptor Tyrosine Kinase 3 |
| VEGFC | Vascular Endothelial Growth Factor C |
| WT1 | WT1 Transcription Factor |
| MUC6 | Mucin 6, Oligomeric Mucus/Gel-Forming |
| SLC2A1 | Solute Carrier Family 2 Member 1 |
| SERPINB5 | Serpin Family B Member 5 |
| CHEK2 | Checkpoint Kinase 2 |
| TYMP | Thymidine Phosphorylase |
| MIR199B | MicroRNA 199b |
| PLAU | Plasminogen Activator, Urokinase |
| MIR107 | MicroRNA 107 |
| CXCR4 | C-X-C Motif Chemokine Receptor 4 |
| KRT8 | Keratin 8 |
| MIR127 | MicroRNA 127 |
| SYP | Synaptophysin |
| MIR106A | MicroRNA 106a |
| KDR | Kinase Insert Domain Receptor |
| CXCL12 | C-X-C Motif Chemokine Ligand 12 |
| HGF | Hepatocyte Growth Factor |
| MIR34B | MicroRNA 34b |
| TYMS | Thymidylate Synthetase |
| MMP7 | Matrix Metallopeptidase 7 |
| NF1 | Neurofibromin 1 |
| MALAT1 | Metastasis Associated Lung Adenocarcinoma Transcript 1 |
| INSR | Insulin Receptor |
| VIM | Vimentin |
| MIR15B | MicroRNA 15b |
| MAPK3 | Mitogen-Activated Protein Kinase 3 |
| CDKN2B | Cyclin Dependent Kinase Inhibitor 2B |
| TNFSF10 | TNF Superfamily Member 10 |
| FOS | Fos Proto-Oncogene, AP-1 Transcription Factor Subunit |
| NRAS | NRAS Proto-Oncogene, GTPase |
| SP1 | Sp1 Transcription Factor |
| MIR34C | MicroRNA 34c |
| PROM1 | Prominin 1 |
| TSC2 | TSC Complex Subunit 2 |
| REG3A | Regenerating Family Member 3 Alpha |
| FGFR3 | Fibroblast Growth Factor Receptor 3 |
| CDKN1C | Cyclin Dependent Kinase Inhibitor 1C |
| SMAD3 | SMAD Family Member 3 |
| TFF1 | Trefoil Factor 1 |
| PLAUR | Plasminogen Activator, Urokinase Receptor |
| MAPK8 | Mitogen-Activated Protein Kinase 8 |
| CCNA2 | Cyclin A2 |
| CEACAM3 | CEA Cell Adhesion Molecule 3 |
| MIR183 | MicroRNA 183 |
| GLI1 | GLI Family Zinc Finger 1 |
| LGALS3 | Galectin 3 |
| RHOA | Ras Homolog Family Member A |
| PTK2 | Protein Tyrosine Kinase 2 |
| S100A4 | S100 Calcium Binding Protein A4 |
| JUN | Jun Proto-Oncogene, AP-1 Transcription Factor Subunit |
| SOD2 | Superoxide Dismutase 2 |
| IGF1 | Insulin Like Growth Factor 1 |
| NFKB1 | Nuclear Factor Kappa B Subunit 1 |
| REG1A | Regenerating Family Member 1 Alpha |
| GRP | Gastrin Releasing Peptide |
| CTSB | Cathepsin B |
| IL1B | Interleukin 1 Beta |
| SNAI1 | Snail Family Transcriptional Repressor 1 |
| CDH2 | Cadherin 2 |
| MIR376A1 | MicroRNA 376a-1 |
| PDGFRB | Platelet Derived Growth Factor Receptor Beta |
| PMS2 | PMS1 Homolog 2, Mismatch Repair System Component |
| NKX2-1 | NK2 Homeobox 1 |
| SMAD2 | SMAD Family Member 2 |
| THBS1 | Thrombospondin 1 |
| VIP | Vasoactive Intestinal Peptide |
| ALK | ALK Receptor Tyrosine Kinase |
| MIR31 | MicroRNA 31 |
| RABL3 | RAB, Member Of RAS Oncogene Family Like 3 |
| PARP1 | Poly(ADP-Ribose) Polymerase 1 |
| TCF7L2 | Transcription Factor 7 Like 2 |
| RAC1 | Rac Family Small GTPase 1 |
| NQO1 | NAD(P)H Quinone Dehydrogenase 1 |
| CD24 | CD24 Molecule |
| CASP9 | Caspase 9 |
| CEACAM6 | CEA Cell Adhesion Molecule 6 |
| FSCN1 | Fascin Actin-Bundling Protein 1 |
| EZR | Ezrin |
| MMP14 | Matrix Metallopeptidase 14 |
| BMP6 | Bone Morphogenetic Protein 6 |
| CCKBR | Cholecystokinin B Receptor |
| MIR199A1 | MicroRNA 199a-1 |
| PTHLH | Parathyroid Hormone Like Hormone |
| E2F1 | E2F Transcription Factor 1 |
| MUC16 | Mucin 16, Cell Surface Associated |
| ANXA5 | Annexin A5 |
| CDK2 | Cyclin Dependent Kinase 2 |
| NTRK1 | Neurotrophic Receptor Tyrosine Kinase 1 |
| SHH | Sonic Hedgehog Signaling Molecule |
| TWIST1 | Twist Family BHLH Transcription Factor 1 |
| ITGB1 | Integrin Subunit Beta 1 |
| MIR29C | MicroRNA 29c |
| PIK3CG | Phosphatidylinositol-4,5-Bisphosphate 3-Kinase Catalytic Subunit Gamma |
| ACVR1B | Activin A Receptor Type 1B |
| STAT1 | Signal Transducer And Activator Of Transcription 1 |
| ZEB1 | Zinc Finger E-Box Binding Homeobox 1 |
| SPARC | Secreted Protein Acidic And Cysteine Rich |
| DNMT1 | DNA Methyltransferase 1 |
| HSP90AA1 | Heat Shock Protein 90 Alpha Family Class A Member 1 |
| HSPB1 | Heat Shock Protein Family B (Small) Member 1 |
| HOTAIR | HOX Transcript Antisense RNA |
| IRS1 | Insulin Receptor Substrate 1 |
| SNAI2 | Snail Family Transcriptional Repressor 2 |
| BAP1 | BRCA1 Associated Protein 1 |
| ABCG2 | ATP Binding Cassette Subfamily G Member 2 (Junior Blood Group) |
| TIMP2 | TIMP Metallopeptidase Inhibitor 2 |
| TGFBR1 | Transforming Growth Factor Beta Receptor 1 |
| DPYD | Dihydropyrimidine Dehydrogenase |
| FLT4 | Fms Related Receptor Tyrosine Kinase 4 |
| SSTR2 | Somatostatin Receptor 2 |
| AREG | Amphiregulin |
| CASR | Calcium Sensing Receptor |
| XIAP | X-Linked Inhibitor Of Apoptosis |
| ENO1 | Enolase 1 |
| RASSF1 | Ras Association Domain Family Member 1 |
| ABCB1 | ATP Binding Cassette Subfamily B Member 1 |
| GSTP1 | Glutathione S-Transferase Pi 1 |
| TLR4 | Toll Like Receptor 4 |
| NTS | Neurotensin |
| SLC29A1 | Solute Carrier Family 29 Member 1 (Augustine Blood Group) |
| PNLIP | Pancreatic Lipase |
| HDAC1 | Histone Deacetylase 1 |
| MIR196A1 | MicroRNA 196a-1 |
| RELA | RELA Proto-Oncogene, NF-KB Subunit |
| PTF1A | Pancreas Associated Transcription Factor 1a |
| GATA6 | GATA Binding Protein 6 |
| NRP1 | Neuropilin 1 |
| TNFRSF10B | TNF Receptor Superfamily Member 10b |
| IGF2BP3 | Insulin Like Growth Factor 2 MRNA Binding Protein 3 |
| HBEGF | Heparin Binding EGF Like Growth Factor |
| BAK1 | BCL2 Antagonist/Killer 1 |
| BSG | Basigin (Ok Blood Group) |
| CTNND1 | Catenin Delta 1 |
| PSCA | Prostate Stem Cell Antigen |
| KLF4 | Kruppel Like Factor 4 |
| MIR204 | MicroRNA 204 |
| BMPR1A | Bone Morphogenetic Protein Receptor Type 1A |
| GAPDH | Glyceraldehyde-3-Phosphate Dehydrogenase |
| CYCS | Cytochrome C, Somatic |
| PLK1 | Polo Like Kinase 1 |
| ESR2 | Estrogen Receptor 2 |
| MCL1 | MCL1 Apoptosis Regulator, BCL2 Family Member |
| VHL | Von Hippel-Lindau Tumor Suppressor |
| PDGFRA | Platelet Derived Growth Factor Receptor Alpha |
| IDH1 | Isocitrate Dehydrogenase (NADP(+)) 1 |
| KCNQ1OT1 | KCNQ1 Opposite Strand/Antisense Transcript 1 |
| TNFRSF10A | TNF Receptor Superfamily Member 10a |
| ANXA2 | Annexin A2 |
| LOX | Lysyl Oxidase |
| HNF4A | Hepatocyte Nuclear Factor 4 Alpha |
| SPP1 | Secreted Phosphoprotein 1 |
| EZH2 | Enhancer Of Zeste 2 Polycomb Repressive Complex 2 Subunit |
| CLDN4 | Claudin 4 |
| CFLAR | CASP8 And FADD Like Apoptosis Regulator |
| CCN2 | Cellular Communication Network Factor 2 |
| TIMP1 | TIMP Metallopeptidase Inhibitor 1 |
| CTRC | Chymotrypsin C |
| GDF15 | Growth Differentiation Factor 15 |
| GADD45A | Growth Arrest And DNA Damage Inducible Alpha |
| POSTN | Periostin |
| CDK6 | Cyclin Dependent Kinase 6 |
| KRT18 | Keratin 18 |
| AR | Androgen Receptor |
| TGFB2 | Transforming Growth Factor Beta 2 |
| PKM | Pyruvate Kinase M1/2 |
| BMP2 | Bone Morphogenetic Protein 2 |
| MIR93 | MicroRNA 93 |
| PVT1 | Pvt1 Oncogene |
| CDC42 | Cell Division Cycle 42 |
| CXCR2 | C-X-C Motif Chemokine Receptor 2 |
| MIR30A | MicroRNA 30a |
| XRCC3 | X-Ray Repair Cross Complementing 3 |
| DCK | Deoxycytidine Kinase |
| MIR342 | MicroRNA 342 |
| ALB | Albumin |
| CCND2 | Cyclin D2 |
| NFKBIA | NFKB Inhibitor Alpha |
| RPS6KB1 | Ribosomal Protein S6 Kinase B1 |
| CASP7 | Caspase 7 |
| MGMT | O-6-Methylguanine-DNA Methyltransferase |
| BCL2L11 | BCL2 Like 11 |
| CD82 | CD82 Molecule |
| CCKAR | Cholecystokinin A Receptor |
| CA2 | Carbonic Anhydrase 2 |
| FGF1 | Fibroblast Growth Factor 1 |
| PIK3R1 | Phosphoinositide-3-Kinase Regulatory Subunit 1 |
| MEG3 | Maternally Expressed 3 |
| GGT1 | Gamma-Glutamyltransferase 1 |
| ENO2 | Enolase 2 |
| UCA1 | Urothelial Cancer Associated 1 |
| MIR125B1 | MicroRNA 125b-1 |
| TP63 | Tumor Protein P63 |
| CHEK1 | Checkpoint Kinase 1 |
| LCN2 | Lipocalin 2 |
| CEACAM7 | CEA Cell Adhesion Molecule 7 |
| SERPINA3 | Serpin Family A Member 3 |
| ERCC6 | ERCC Excision Repair 6, Chromatin Remodeling Factor |
| MIR142 | MicroRNA 142 |
| MIR30E | MicroRNA 30e |
| MIR99A | MicroRNA 99a |
| BID | BH3 Interacting Domain Death Agonist |
| LEP | Leptin |
| MAP2K4 | Mitogen-Activated Protein Kinase Kinase 4 |
| AXIN2 | Axin 2 |
| HDAC9 | Histone Deacetylase 9 |
| AFAP1-AS1 | AFAP1 Antisense RNA 1 |
| GSTM1 | Glutathione S-Transferase Mu 1 |
| S100A2 | S100 Calcium Binding Protein A2 |
| NES | Nestin |
| DNMT3A | DNA Methyltransferase 3 Alpha |
| MIR18A | MicroRNA 18a |
| TUG1 | Taurine Up-Regulated 1 |
| JAK1 | Janus Kinase 1 |
| AKT3 | AKT Serine/Threonine Kinase 3 |
| RRM2 | Ribonucleotide Reductase Regulatory Subunit M2 |
| FOXM1 | Forkhead Box M1 |
| RRM1 | Ribonucleotide Reductase Catalytic Subunit M1 |
| FLT1 | Fms Related Receptor Tyrosine Kinase 1 |
| ALOX5 | Arachidonate 5-Lipoxygenase |
| NDRG1 | N-Myc Downstream Regulated 1 |
| CYP17A1 | Cytochrome P450 Family 17 Subfamily A Member 1 |
| CSF3 | Colony Stimulating Factor 3 |
| PTCH1 | Patched 1 |
| MIR146B | MicroRNA 146b |
| HOTTIP | HOXA Distal Transcript Antisense RNA |
| NME1 | NME/NM23 Nucleoside Diphosphate Kinase 1 |
| NTSR1 | Neurotensin Receptor 1 |
| MMP1 | Matrix Metallopeptidase 1 |
| ILK | Integrin Linked Kinase |
| JAG1 | Jagged Canonical Notch Ligand 1 |
| HPSE | Heparanase |
| TSC1 | TSC Complex Subunit 1 |
| CCNB1 | Cyclin B1 |
| GAS5 | Growth Arrest Specific 5 |
| LINC-ROR | Long Intergenic Non-Protein Coding RNA, Regulator Of Reprogramming |
| ABCC1 | ATP Binding Cassette Subfamily C Member 1 |
| BAD | BCL2 Associated Agonist Of Cell Death |
| PHB | Prohibitin |
| NFE2L2 | Nuclear Factor, Erythroid 2 Like 2 |
| FN1 | Fibronectin 1 |
| PRKD1 | Protein Kinase D1 |
| IGFBP3 | Insulin Like Growth Factor Binding Protein 3 |
| TGM2 | Transglutaminase 2 |
| MAPK14 | Mitogen-Activated Protein Kinase 14 |
| MIR373 | MicroRNA 373 |
| GCG | Glucagon |
| ERBB4 | Erb-B2 Receptor Tyrosine Kinase 4 |
| RARB | Retinoic Acid Receptor Beta |
| MME | Membrane Metalloendopeptidase |
| CCR6 | C-C Motif Chemokine Receptor 6 |
| SPRY4-IT1 | SPRY4 Intronic Transcript 1 |
| E2F3 | E2F Transcription Factor 3 |
| PPY | Pancreatic Polypeptide |
| CTAG1B | Cancer/Testis Antigen 1B |
| F2RL1 | F2R Like Trypsin Receptor 1 |
| TIMP3 | TIMP Metallopeptidase Inhibitor 3 |
| FOXP3 | Forkhead Box P3 |
| CHUK | Component Of Inhibitor Of Nuclear Factor Kappa B Kinase Complex |
| EIF4EBP1 | Eukaryotic Translation Initiation Factor 4E Binding Protein 1 |
| NEAT1 | Nuclear Paraspeckle Assembly Transcript 1 |
| PENK | Proenkephalin |
| MSH3 | MutS Homolog 3 |
| IL2 | Interleukin 2 |
| KRT14 | Keratin 14 |
| PCNA | Proliferating Cell Nuclear Antigen |
| S100A6 | S100 Calcium Binding Protein A6 |
| MIR497 | MicroRNA 497 |
| XRCC1 | X-Ray Repair Cross Complementing 1 |
| NOS2 | Nitric Oxide Synthase 2 |
| CYP1A1 | Cytochrome P450 Family 1 Subfamily A Member 1 |
| SLC22A18 | Solute Carrier Family 22 Member 18 |
| ALDH1A1 | Aldehyde Dehydrogenase 1 Family Member A1 |
| GRPR | Gastrin Releasing Peptide Receptor |
| MRE11 | MRE11 Homolog, Double Strand Break Repair Nuclease |
| CDKN2B-AS1 | CDKN2B Antisense RNA 1 |
| HSPA5 | Heat Shock Protein Family A (Hsp70) Member 5 |
| SOX2 | SRY-Box Transcription Factor 2 |
| PRKAR1A | Protein Kinase CAMP-Dependent Type I Regulatory Subunit Alpha |
| PRSS2 | Serine Protease 2 |
| AXL | AXL Receptor Tyrosine Kinase |
| MAPK10 | Mitogen-Activated Protein Kinase 10 |
| MIR199A2 | MicroRNA 199a-2 |
| MIR191 | MicroRNA 191 |
| S100PBP | S100P Binding Protein |
| ELANE | Elastase, Neutrophil Expressed |
| MIR197 | MicroRNA 197 |
| CCAT1 | Colon Cancer Associated Transcript 1 |
| DKC1 | Dyskerin Pseudouridine Synthase 1 |
| PKD1 | Polycystin 1, Transient Receptor Potential Channel Interacting |
| ANXA1 | Annexin A1 |
| MIR23A | MicroRNA 23a |
| HNF1A | HNF1 Homeobox A |
| POU5F1 | POU Class 5 Homeobox 1 |
| PIK3CB | Phosphatidylinositol-4,5-Bisphosphate 3-Kinase Catalytic Subunit Beta |
| MIR29B2 | MicroRNA 29b-2 |
| BMI1 | BMI1 Proto-Oncogene, Polycomb Ring Finger |
| NBN | Nibrin |
| MIRLET7I | MicroRNA Let-7i |
| KLK3 | Kallikrein Related Peptidase 3 |
| SLC35F6 | Solute Carrier Family 35 Member F6 |
| MIR139 | MicroRNA 139 |
| JAK2 | Janus Kinase 2 |
| MIR181B1 | MicroRNA 181b-1 |
| MIR198 | MicroRNA 198 |
| TNFRSF6B | TNF Receptor Superfamily Member 6b |
| LINC00673 | Long Intergenic Non-Protein Coding RNA 673 |
| EIF2AK3 | Eukaryotic Translation Initiation Factor 2 Alpha Kinase 3 |
| CCND3 | Cyclin D3 |
| MMP3 | Matrix Metallopeptidase 3 |
| SNHG15 | Small Nucleolar RNA Host Gene 15 |
| CCL2 | C-C Motif Chemokine Ligand 2 |
| CASC2 | Cancer Susceptibility 2 |
| EPHA2 | EPH Receptor A2 |
| MIR200B | MicroRNA 200b |
| MIR330 | MicroRNA 330 |
| CTLA4 | Cytotoxic T-Lymphocyte Associated Protein 4 |
| MTHFR | Methylenetetrahydrofolate Reductase |
| MIR29A | MicroRNA 29a |
| BNIP3 | BCL2 Interacting Protein 3 |
| MIR148B | MicroRNA 148b |
| ETV4 | ETS Variant Transcription Factor 4 |
| SMO | Smoothened, Frizzled Class Receptor |
| MIR181A1 | MicroRNA 181a-1 |
| GDNF | Glial Cell Derived Neurotrophic Factor |
| IKBKB | Inhibitor Of Nuclear Factor Kappa B Kinase Subunit Beta |
| GSN | Gelsolin |
| HFE | Homeostatic Iron Regulator |
| BUB1B | BUB1 Mitotic Checkpoint Serine/Threonine Kinase B |
| ABCB4 | ATP Binding Cassette Subfamily B Member 4 |
| MIR212 | MicroRNA 212 |
| E2F2 | E2F Transcription Factor 2 |
| RAD51D | RAD51 Paralog D |
| CYP19A1 | Cytochrome P450 Family 19 Subfamily A Member 1 |
| MIF | Macrophage Migration Inhibitory Factor |
| PLA2G2A | Phospholipase A2 Group IIA |
| COPS5 | COP9 Signalosome Subunit 5 |
| TP73 | Tumor Protein P73 |
| SERPINE1 | Serpin Family E Member 1 |
| CAT | Catalase |
| CRP | C-Reactive Protein |
| PDCD1 | Programmed Cell Death 1 |
| PIK3R3 | Phosphoinositide-3-Kinase Regulatory Subunit 3 |
| XIST | X Inactive Specific Transcript |
| PLG | Plasminogen |
| HK2 | Hexokinase 2 |
| POLE | DNA Polymerase Epsilon, Catalytic Subunit |
| CLDN7 | Claudin 7 |
| CYTOR | Cytoskeleton Regulator RNA |
| OGG1 | 8-Oxoguanine DNA Glycosylase |
| RAD51C | RAD51 Paralog C |
| MSR1 | Macrophage Scavenger Receptor 1 |
| GH1 | Growth Hormone 1 |
| KLF10 | Kruppel Like Factor 10 |
| CTSD | Cathepsin D |
| IL32 | Interleukin 32 |
| PDPN | Podoplanin |
| IL24 | Interleukin 24 |
| CALCA | Calcitonin Related Polypeptide Alpha |
| SIRT1 | Sirtuin 1 |
| CREB1 | CAMP Responsive Element Binding Protein 1 |
| YAP1 | Yes Associated Protein 1 |
| CRNDE | Colorectal Neoplasia Differentially Expressed |
| OLFM4 | Olfactomedin 4 |
| HNF1A-AS1 | HNF1A Antisense RNA 1 |
| FOXO3 | Forkhead Box O3 |
| NUPR1 | Nuclear Protein 1, Transcriptional Regulator |
| CDC73 | Cell Division Cycle 73 |
| U2AF1 | U2 Small Nuclear RNA Auxiliary Factor 1 |
| TOP2A | DNA Topoisomerase II Alpha |
| MIR24-1 | MicroRNA 24-1 |
| PIP | Prolactin Induced Protein |
| IFI27 | Interferon Alpha Inducible Protein 27 |
| EIF4E | Eukaryotic Translation Initiation Factor 4E |
| STMN1 | Stathmin 1 |
| RALA | RAS Like Proto-Oncogene A |
| SETD2 | SET Domain Containing 2, Histone Lysine Methyltransferase |
| MIR32 | MicroRNA 32 |
| MIR345 | MicroRNA 345 |
| MSMB | Microseminoprotein Beta |
| SOX9 | SRY-Box Transcription Factor 9 |
| ODC1 | Ornithine Decarboxylase 1 |
| KLF6 | Kruppel Like Factor 6 |
| MIRLET7F1 | MicroRNA Let-7f-1 |
| VEGFD | Vascular Endothelial Growth Factor D |
| MIR301A | MicroRNA 301a |
| SPINT2 | Serine Peptidase Inhibitor, Kunitz Type 2 |
| GHRH | Growth Hormone Releasing Hormone |
| SULF1 | Sulfatase 1 |
| FAM3C | FAM3 Metabolism Regulating Signaling Molecule C |
| CDKN2C | Cyclin Dependent Kinase Inhibitor 2C |
| MIR24-2 | MicroRNA 24-2 |
| KRT5 | Keratin 5 |
| FASN | Fatty Acid Synthase |
| ITGA6 | Integrin Subunit Alpha 6 |
| FANCC | FA Complementation Group C |
| GSK3B | Glycogen Synthase Kinase 3 Beta |
| LNCRNA-ATB | Long Noncoding RNA Activated By TGF-Beta |
| DAPK1 | Death Associated Protein Kinase 1 |
| IRAIN | IGF1R Antisense Imprinted Non-Protein Coding RNA |
| GHET1 | Gastric Carcinoma Proliferation Enhancing Transcript 1 |
| BECN1 | Beclin 1 |
| ABCC8 | ATP Binding Cassette Subfamily C Member 8 |
| FGF7 | Fibroblast Growth Factor 7 |
| TNFRSF1A | TNF Receptor Superfamily Member 1A |
| GFRA1 | GDNF Family Receptor Alpha 1 |
| MIR181D | MicroRNA 181d |
| SKP2 | S-Phase Kinase Associated Protein 2 |
| H2AC18 | H2A Clustered Histone 18 |
| NRG1 | Neuregulin 1 |
| NORAD | Non-Coding RNA Activated By DNA Damage |
| MIR202 | MicroRNA 202 |
| ERG | ETS Transcription Factor ERG |
| RHOB | Ras Homolog Family Member B |
| IFNG | Interferon Gamma |
| CLCA4 | Chloride Channel Accessory 4 |
| BARD1 | BRCA1 Associated RING Domain 1 |
| MIR193B | MicroRNA 193b |
| RNF43 | Ring Finger Protein 43 |
| CEP290 | Centrosomal Protein 290 |
| NOTCH3 | Notch Receptor 3 |
| MIR196B | MicroRNA 196b |
| MIR181C | MicroRNA 181c |
| CSF2 | Colony Stimulating Factor 2 |
| DNMT3B | DNA Methyltransferase 3 Beta |
| LINC00261 | Long Intergenic Non-Protein Coding RNA 261 |
| CA9 | Carbonic Anhydrase 9 |
| PRKCA | Protein Kinase C Alpha |
| XRCC2 | X-Ray Repair Cross Complementing 2 |
| MIR424 | MicroRNA 424 |
| NR1H4 | Nuclear Receptor Subfamily 1 Group H Member 4 |
| EPHA3 | EPH Receptor A3 |
| FANCG | FA Complementation Group G |
| ABCC2 | ATP Binding Cassette Subfamily C Member 2 |
| PYY | Peptide YY |
| CDKN3 | Cyclin Dependent Kinase Inhibitor 3 |
| RNASE1 | Ribonuclease A Family Member 1, Pancreatic |
| HSPA4 | Heat Shock Protein Family A (Hsp70) Member 4 |
| FOXA1 | Forkhead Box A1 |
| RALB | RAS Like Proto-Oncogene B |
| NEUROD1 | Neuronal Differentiation 1 |
| VDR | Vitamin D Receptor |
| GCK | Glucokinase |
| ERCC1 | ERCC Excision Repair 1, Endonuclease Non-Catalytic Subunit |
| AMACR | Alpha-Methylacyl-CoA Racemase |
| BMP4 | Bone Morphogenetic Protein 4 |
| RNASEL | Ribonuclease L |
| NAT2 | N-Acetyltransferase 2 |
| WT1-AS | WT1 Antisense RNA |
| GIPC1 | GIPC PDZ Domain Containing Family Member 1 |
| AFP | Alpha Fetoprotein |
| IGF2R | Insulin Like Growth Factor 2 Receptor |
| NEK8 | NIMA Related Kinase 8 |
| HULC | Hepatocellular Carcinoma Up-Regulated Long Non-Coding RNA |
| OGFR | Opioid Growth Factor Receptor |
| B3GALT5 | Beta-1,3-Galactosyltransferase 5 |
| CCDC26 | CCDC26 Long Non-Coding RNA |
| MIRLET7G | MicroRNA Let-7g |
| ETS1 | ETS Proto-Oncogene 1, Transcription Factor |
| EPAS1 | Endothelial PAS Domain Protein 1 |
| GACAT2 | Gastric Cancer Associated Transcript 2 |
| MIRLET7B | MicroRNA Let-7b |
| SOCS3 | Suppressor Of Cytokine Signaling 3 |
| MIR206 | MicroRNA 206 |
| TEK | TEK Receptor Tyrosine Kinase |
| MIR217 | MicroRNA 217 |
| RAD54L | RAD54 Like |
| MIR181A2 | MicroRNA 181a-2 |
| TUSC3 | Tumor Suppressor Candidate 3 |
| HOXB2 | Homeobox B2 |
| GRB2 | Growth Factor Receptor Bound Protein 2 |
| NOP14 | NOP14 Nucleolar Protein |
| CCNG1 | Cyclin G1 |
| S100P | S100 Calcium Binding Protein P |
| MIR92A1 | MicroRNA 92a-1 |
| NGF | Nerve Growth Factor |
| AGR2 | Anterior Gradient 2, Protein Disulphide Isomerase Family Member |
| IGFBP2 | Insulin Like Growth Factor Binding Protein 2 |
| CCNE1 | Cyclin E1 |
| WWOX | WW Domain Containing Oxidoreductase |
| CLPS | Colipase |
| MUTYH | MutY DNA Glycosylase |
| INHBA | Inhibin Subunit Beta A |
| RB1CC1 | RB1 Inducible Coiled-Coil 1 |
| LGALS1 | Galectin 1 |
| ATP8B1 | ATPase Phospholipid Transporting 8B1 |
| LINC01772 | Long Intergenic Non-Protein Coding RNA 1772 |
| GPC3 | Glypican 3 |
| ACP3 | Acid Phosphatase 3 |
| FBXW7 | F-Box And WD Repeat Domain Containing 7 |
| MIR92A2 | MicroRNA 92a-2 |
| FOXO1 | Forkhead Box O1 |
| MIR224 | MicroRNA 224 |
| HLA-A | Major Histocompatibility Complex, Class I, A |
| PAK1 | P21 (RAC1) Activated Kinase 1 |
| RHOC | Ras Homolog Family Member C |
| CHKA | Choline Kinase Alpha |
| NTRK3 | Neurotrophic Receptor Tyrosine Kinase 3 |
| DLEU2 | Deleted In Lymphocytic Leukemia 2 |
| CCL5 | C-C Motif Chemokine Ligand 5 |
| KDM4C | Lysine Demethylase 4C |
| KCNJ11 | Potassium Inwardly Rectifying Channel Subfamily J Member 11 |
| CSF1 | Colony Stimulating Factor 1 |
| NPY | Neuropeptide Y |
| MPO | Myeloperoxidase |
| CSF1R | Colony Stimulating Factor 1 Receptor |
| RUNX3 | RUNX Family Transcription Factor 3 |
| BDNF | Brain Derived Neurotrophic Factor |
| DDR2 | Discoidin Domain Receptor Tyrosine Kinase 2 |
| INHA | Inhibin Subunit Alpha |
| MMP11 | Matrix Metallopeptidase 11 |
| STAT5B | Signal Transducer And Activator Of Transcription 5B |
| MIR486-1 | MicroRNA 486-1 |
| GHRL | Ghrelin And Obestatin Prepropeptide |
| ELAVL1 | ELAV Like RNA Binding Protein 1 |
| TFAP2A | Transcription Factor AP-2 Alpha |
| ELAC2 | ElaC Ribonuclease Z 2 |
| MIR335 | MicroRNA 335 |
| APOE | Apolipoprotein E |
| IRS2 | Insulin Receptor Substrate 2 |
| RXRA | Retinoid X Receptor Alpha |
| CDC25A | Cell Division Cycle 25A |
| ADIPOQ | Adiponectin, C1Q And Collagen Domain Containing |
| MIR140 | MicroRNA 140 |
| SERPINA1 | Serpin Family A Member 1 |
| ABCB11 | ATP Binding Cassette Subfamily B Member 11 |
| MIR31HG | MIR31 Host Gene |
| CD36 | CD36 Molecule |
| CDK1 | Cyclin Dependent Kinase 1 |
| NR1H2 | Nuclear Receptor Subfamily 1 Group H Member 2 |
| NOTCH2 | Notch Receptor 2 |
| KIAA0087 | KIAA0087 LncRNA |
| ARAF | A-Raf Proto-Oncogene, Serine/Threonine Kinase |
| ACTC1 | Actin Alpha Cardiac Muscle 1 |
| ENSG00000284669 |  |
| CYP1B1 | Cytochrome P450 Family 1 Subfamily B Member 1 |
| SCTR | Secretin Receptor |
| MIR103A2 | MicroRNA 103a-2 |
| IL2RA | Interleukin 2 Receptor Subunit Alpha |
| PRKCB | Protein Kinase C Beta |
| MIR451A | MicroRNA 451a |
| ENSG00000261220 |  |
| HMGB1 | High Mobility Group Box 1 |
| MIR9-1 | MicroRNA 9-1 |
| PAX6 | Paired Box 6 |
| MIR181B2 | MicroRNA 181b-2 |
| IL4 | Interleukin 4 |
| MIR106B | MicroRNA 106b |
| ZBTB20-AS4 | ZBTB20 Antisense RNA 4 |
| ENSG00000244953 |  |
| LINC02041 | Long Intergenic Non-Protein Coding RNA 2041 |
| ENSG00000261211 |  |
| DDX6P1 | DEAD-Box Helicase 6 Pseudogene 1 |
| ENSG00000229962 |  |
| NUTF2P3 | Nuclear Transport Factor 2 Pseudogene 3 |
| LOC105377769 | Uncharacterized LOC105377769 |
| MIR429 | MicroRNA 429 |
| ETV1 | ETS Variant Transcription Factor 1 |
| PMS1 | PMS1 Homolog 1, Mismatch Repair System Component |
| ERCC2 | ERCC Excision Repair 2, TFIIH Core Complex Helicase Subunit |
| CDH3 | Cadherin 3 |
| ICAM1 | Intercellular Adhesion Molecule 1 |
| IL18 | Interleukin 18 |
| HES1 | Hes Family BHLH Transcription Factor 1 |
| PTPRC | Protein Tyrosine Phosphatase Receptor Type C |
| TXN | Thioredoxin |
| IL3 | Interleukin 3 |
| JUP | Junction Plakoglobin |
| EPHB2 | EPH Receptor B2 |
| SAT1 | Spermidine/Spermine N1-Acetyltransferase 1 |
| IFNA2 | Interferon Alpha 2 |
| ACE | Angiotensin I Converting Enzyme |
| CYP2E1 | Cytochrome P450 Family 2 Subfamily E Member 1 |
| SLC10A2 | Solute Carrier Family 10 Member 2 |
| DPP4 | Dipeptidyl Peptidase 4 |
| CXCL1 | C-X-C Motif Chemokine Ligand 1 |
| HMGCR | 3-Hydroxy-3-Methylglutaryl-CoA Reductase |
| KEAP1 | Kelch Like ECH Associated Protein 1 |
| AHR | Aryl Hydrocarbon Receptor |
| WDR19 | WD Repeat Domain 19 |
| APEX1 | Apurinic/Apyrimidinic Endodeoxyribonuclease 1 |
| PPM1D | Protein Phosphatase, Mg2+/Mn2+ Dependent 1D |
| NCAM1 | Neural Cell Adhesion Molecule 1 |
| MIR130B | MicroRNA 130b |
| ANGPT2 | Angiopoietin 2 |
| ENG | Endoglin |
| MIR192 | MicroRNA 192 |
| STAT5A | Signal Transducer And Activator Of Transcription 5A |
| MIR216A | MicroRNA 216a |
| FAP | Fibroblast Activation Protein Alpha |
| CCN1 | Cellular Communication Network Factor 1 |
| TLR2 | Toll Like Receptor 2 |
| MIR615 | MicroRNA 615 |
| CNC2 | Carney Complex Type 2, Multiple Neoplasia And Lentiginosis |
| CYP3A4 | Cytochrome P450 Family 3 Subfamily A Member 4 |
| PDPK1 | 3-Phosphoinositide Dependent Protein Kinase 1 |
| CYP2D6 | Cytochrome P450 Family 2 Subfamily D Member 6 |
| THBD | Thrombomodulin |
| PDCD4 | Programmed Cell Death 4 |
| PKHD1 | PKHD1 Ciliary IPT Domain Containing Fibrocystin/Polyductin |
| SMARCA4 | SWI/SNF Related, Matrix Associated, Actin Dependent Regulator Of Chromatin, Subfamily A, Member 4 |
| S100A8 | S100 Calcium Binding Protein A8 |
| CYP1A2 | Cytochrome P450 Family 1 Subfamily A Member 2 |
| ITGAV | Integrin Subunit Alpha V |
| MTA1 | Metastasis Associated 1 |
| SHC1 | SHC Adaptor Protein 1 |
| CLU | Clusterin |
| IDO1 | Indoleamine 2,3-Dioxygenase 1 |
| SSTR5 | Somatostatin Receptor 5 |
| PXN | Paxillin |
| MMP13 | Matrix Metallopeptidase 13 |
| ZG16B | Zymogen Granule Protein 16B |
| VWF | Von Willebrand Factor |
| LEPR | Leptin Receptor |
| CCR7 | C-C Motif Chemokine Receptor 7 |
| PTK2B | Protein Tyrosine Kinase 2 Beta |
| CALB2 | Calbindin 2 |
| PECAM1 | Platelet And Endothelial Cell Adhesion Molecule 1 |
| MMP26 | Matrix Metallopeptidase 26 |
| IL17A | Interleukin 17A |
| RELB | RELB Proto-Oncogene, NF-KB Subunit |
| STC1 | Stanniocalcin 1 |
| DHFR | Dihydrofolate Reductase |
| CD40 | CD40 Molecule |
| CALR | Calreticulin |
| MAP3K1 | Mitogen-Activated Protein Kinase Kinase Kinase 1 |
| FGF10 | Fibroblast Growth Factor 10 |
| CD34 | CD34 Molecule |
| GATA3 | GATA Binding Protein 3 |
| HMOX1 | Heme Oxygenase 1 |
| CREBBP | CREB Binding Protein |
| IAPP | Islet Amyloid Polypeptide |
| CRTC1 | CREB Regulated Transcription Coactivator 1 |
| SFN | Stratifin |
| TIMP4 | TIMP Metallopeptidase Inhibitor 4 |
| HMGA2 | High Mobility Group AT-Hook 2 |
| NTRK2 | Neurotrophic Receptor Tyrosine Kinase 2 |
| ADRB2 | Adrenoceptor Beta 2 |
| GJA1 | Gap Junction Protein Alpha 1 |
| FOXA2 | Forkhead Box A2 |
| MUC5B | Mucin 5B, Oligomeric Mucus/Gel-Forming |
| PEBP1 | Phosphatidylethanolamine Binding Protein 1 |
| REG4 | Regenerating Family Member 4 |
| SLC9A3R1 | SLC9A3 Regulator 1 |
| HP | Haptoglobin |
| PLAT | Plasminogen Activator, Tissue Type |
| NAT1 | N-Acetyltransferase 1 |
| WNT5A | Wnt Family Member 5A |
| MIR149 | MicroRNA 149 |
| TMPRSS15 | Transmembrane Serine Protease 15 |
| KISS1 | KiSS-1 Metastasis Suppressor |
| COMT | Catechol-O-Methyltransferase |
| MIR494 | MicroRNA 494 |
| NOTCH4 | Notch Receptor 4 |
| ALOX12 | Arachidonate 12-Lipoxygenase, 12S Type |
| IER3 | Immediate Early Response 3 |
| B2M | Beta-2-Microglobulin |
| NANOG | Nanog Homeobox |
| AURKB | Aurora Kinase B |
| CELA3B | Chymotrypsin Like Elastase 3B |
| ECT2 | Epithelial Cell Transforming 2 |
| PTPN11 | Protein Tyrosine Phosphatase Non-Receptor Type 11 |
| MVP | Major Vault Protein |
| PLCG1 | Phospholipase C Gamma 1 |
| SLPI | Secretory Leukocyte Peptidase Inhibitor |
| TKT | Transketolase |
| SREBF1 | Sterol Regulatory Element Binding Transcription Factor 1 |
| CD40LG | CD40 Ligand |
| IFNA1 | Interferon Alpha 1 |
| CD4 | CD4 Molecule |
| NCOA3 | Nuclear Receptor Coactivator 3 |
| FOLH1 | Folate Hydrolase 1 |
| DDR1 | Discoidin Domain Receptor Tyrosine Kinase 1 |
| EDNRA | Endothelin Receptor Type A |
| MIR409 | MicroRNA 409 |
| SLC2A2 | Solute Carrier Family 2 Member 2 |
| MIR98 | MicroRNA 98 |
| AMY2A | Amylase Alpha 2A |
| ABCC3 | ATP Binding Cassette Subfamily C Member 3 |
| MIR339 | MicroRNA 339 |
| YBX1 | Y-Box Binding Protein 1 |
| ROS1 | ROS Proto-Oncogene 1, Receptor Tyrosine Kinase |
| PTPN3 | Protein Tyrosine Phosphatase Non-Receptor Type 3 |
| ADA | Adenosine Deaminase |
| MIR331 | MicroRNA 331 |
| RECK | Reversion Inducing Cysteine Rich Protein With Kazal Motifs |
| NR3C1 | Nuclear Receptor Subfamily 3 Group C Member 1 |
| GPER1 | G Protein-Coupled Estrogen Receptor 1 |
| DUSP1 | Dual Specificity Phosphatase 1 |
| IGFBP1 | Insulin Like Growth Factor Binding Protein 1 |
| TCTN1 | Tectonic Family Member 1 |
| TGFB3 | Transforming Growth Factor Beta 3 |
| BRIP1 | BRCA1 Interacting Protein C-Terminal Helicase 1 |
| MYH11 | Myosin Heavy Chain 11 |
| BRD4 | Bromodomain Containing 4 |
| S100B | S100 Calcium Binding Protein B |
| LINC00511 | Long Intergenic Non-Protein Coding RNA 511 |
| F3 | Coagulation Factor III, Tissue Factor |
| RAD50 | RAD50 Double Strand Break Repair Protein |
| TTN | Titin |
| PTPRG | Protein Tyrosine Phosphatase Receptor Type G |
| EBAG9 | Estrogen Receptor Binding Site Associated Antigen 9 |
| PDGFA | Platelet Derived Growth Factor Subunit A |
| DDIT3 | DNA Damage Inducible Transcript 3 |
| MCM2 | Minichromosome Maintenance Complex Component 2 |
| RARA | Retinoic Acid Receptor Alpha |
| TGIF1 | TGFB Induced Factor Homeobox 1 |
| PSMD4 | Proteasome 26S Subunit, Non-ATPase 4 |
| FADD | Fas Associated Via Death Domain |
| GPT | Glutamic--Pyruvic Transaminase |
| KRT17 | Keratin 17 |
| ITGA3 | Integrin Subunit Alpha 3 |
| ONECUT1 | One Cut Homeobox 1 |
| HOXB13 | Homeobox B13 |
| GSTT1 | Glutathione S-Transferase Theta 1 |
| IL1A | Interleukin 1 Alpha |
| WWTR1 | WW Domain Containing Transcription Regulator 1 |
| MIRLET7A3 | MicroRNA Let-7a-3 |
| ARID1A | AT-Rich Interaction Domain 1A |
| FGF19 | Fibroblast Growth Factor 19 |
| ELN | Elastin |
| DKK1 | Dickkopf WNT Signaling Pathway Inhibitor 1 |
| FLNB | Filamin B |
| ABLIM2 | Actin Binding LIM Protein Family Member 2 |
| VCAM1 | Vascular Cell Adhesion Molecule 1 |
| ITGB4 | Integrin Subunit Beta 4 |
| ROCK1 | Rho Associated Coiled-Coil Containing Protein Kinase 1 |
| HSPD1 | Heat Shock Protein Family D (Hsp60) Member 1 |
| TCF4 | Transcription Factor 4 |
| ST14 | Suppression Of Tumorigenicity 14 |
| GUCY2C | Guanylate Cyclase 2C |
| MIRLET7C | MicroRNA Let-7c |
| DMBT1 | Deleted In Malignant Brain Tumors 1 |
| TOP1 | DNA Topoisomerase I |
| SRGAP1 | SLIT-ROBO Rho GTPase Activating Protein 1 |
| HSPA1A | Heat Shock Protein Family A (Hsp70) Member 1A |
| SSTR1 | Somatostatin Receptor 1 |
| MIR124-3 | MicroRNA 124-3 |
| PAX8 | Paired Box 8 |
| NAPSA | Napsin A Aspartic Peptidase |
| HDAC2 | Histone Deacetylase 2 |
| CDH13 | Cadherin 13 |
| CHGB | Chromogranin B |
| CISH | Cytokine Inducible SH2 Containing Protein |
| IL13 | Interleukin 13 |
| ANPEP | Alanyl Aminopeptidase, Membrane |
| SSTR3 | Somatostatin Receptor 3 |
| TUBB | Tubulin Beta Class I |
| CASP2 | Caspase 2 |
| UHRF1 | Ubiquitin Like With PHD And Ring Finger Domains 1 |
| LAMC2 | Laminin Subunit Gamma 2 |
| LEF1 | Lymphoid Enhancer Binding Factor 1 |
| MBL2 | Mannose Binding Lectin 2 |
| SNCG | Synuclein Gamma |
| PLA2G1B | Phospholipase A2 Group IB |
| KITLG | KIT Ligand |
| SOD1 | Superoxide Dismutase 1 |
| MAML2 | Mastermind Like Transcriptional Coactivator 2 |
| MTDH | Metadherin |
| AFAP1 | Actin Filament Associated Protein 1 |
| ATR | ATR Serine/Threonine Kinase |
| STX1A | Syntaxin 1A |
| PDGFB | Platelet Derived Growth Factor Subunit B |
| AGER | Advanced Glycosylation End-Product Specific Receptor |
| GHR | Growth Hormone Receptor |
| MIR124-1 | MicroRNA 124-1 |
| PRL | Prolactin |
| TMEM238L | Transmembrane Protein 238 Like |
| NR1I2 | Nuclear Receptor Subfamily 1 Group I Member 2 |
| CLDN18 | Claudin 18 |
| MYCN | MYCN Proto-Oncogene, BHLH Transcription Factor |
| MXI1 | MAX Interactor 1, Dimerization Protein |
| CTSL | Cathepsin L |
| CXCL10 | C-X-C Motif Chemokine Ligand 10 |
| FOSL1 | FOS Like 1, AP-1 Transcription Factor Subunit |
| BIRC3 | Baculoviral IAP Repeat Containing 3 |
| ANG | Angiogenin |
| SOCS1 | Suppressor Of Cytokine Signaling 1 |
| PRKN | Parkin RBR E3 Ubiquitin Protein Ligase |
| SDHD | Succinate Dehydrogenase Complex Subunit D |
| NF2 | Neurofibromin 2 |
| LTF | Lactotransferrin |
| DIABLO | Diablo IAP-Binding Mitochondrial Protein |
| SFTPD | Surfactant Protein D |
| VEGFB | Vascular Endothelial Growth Factor B |
| MIR125B2 | MicroRNA 125b-2 |
| PNLIPRP1 | Pancreatic Lipase Related Protein 1 |
| RARS1 | Arginyl-TRNA Synthetase 1 |
| BCL10 | BCL10 Immune Signaling Adaptor |
| NGFR | Nerve Growth Factor Receptor |
| TUSC7 | Tumor Suppressor Candidate 7 |
| SLC7A5 | Solute Carrier Family 7 Member 5 |
| VTN | Vitronectin |
| PTGS1 | Prostaglandin-Endoperoxide Synthase 1 |
| MIR151A | MicroRNA 151a |
| CLDN3 | Claudin 3 |
| MUC17 | Mucin 17, Cell Surface Associated |
| SLC16A1 | Solute Carrier Family 16 Member 1 |
| RUNX2 | RUNX Family Transcription Factor 2 |
| WNT1 | Wnt Family Member 1 |
| IL6R | Interleukin 6 Receptor |
| NOS3 | Nitric Oxide Synthase 3 |
| ROBO1 | Roundabout Guidance Receptor 1 |
| LDHA | Lactate Dehydrogenase A |
| EPOR | Erythropoietin Receptor |
| KLK10 | Kallikrein Related Peptidase 10 |
| HSPA8 | Heat Shock Protein Family A (Hsp70) Member 8 |
| ZEB2 | Zinc Finger E-Box Binding Homeobox 2 |
| S100A9 | S100 Calcium Binding Protein A9 |
| XBP1 | X-Box Binding Protein 1 |
| BIRC2 | Baculoviral IAP Repeat Containing 2 |
| ALCAM | Activated Leukocyte Cell Adhesion Molecule |
| TG | Thyroglobulin |
| SELE | Selectin E |
| CXCR1 | C-X-C Motif Chemokine Receptor 1 |
| UCHL1 | Ubiquitin C-Terminal Hydrolase L1 |
| DCC | DCC Netrin 1 Receptor |
| CEACAM1 | CEA Cell Adhesion Molecule 1 |
| SLC25A13 | Solute Carrier Family 25 Member 13 |
| NQO2 | N-Ribosyldihydronicotinamide:Quinone Reductase 2 |
| EPO | Erythropoietin |
| CDC25B | Cell Division Cycle 25B |
| CSK | C-Terminal Src Kinase |
| TLR9 | Toll Like Receptor 9 |
| DCLK1 | Doublecortin Like Kinase 1 |
| IGFBP5 | Insulin Like Growth Factor Binding Protein 5 |
| LINC00491 | Long Intergenic Non-Protein Coding RNA 491 |
| LMNA | Lamin A/C |
| SDC1 | Syndecan 1 |
| CCNE2 | Cyclin E2 |
| SOX2-OT | SOX2 Overlapping Transcript |
| TDGF1 | Teratocarcinoma-Derived Growth Factor 1 |
| FENDRR | FOXF1 Adjacent Non-Coding Developmental Regulatory RNA |
| GLP1R | Glucagon Like Peptide 1 Receptor |
| EDN1 | Endothelin 1 |
| ACCS | 1-Aminocyclopropane-1-Carboxylate Synthase Homolog (Inactive) |
| MAP3K14 | Mitogen-Activated Protein Kinase Kinase Kinase 14 |
| EREG | Epiregulin |
| INSM1 | INSM Transcriptional Repressor 1 |
| IGF2-AS | IGF2 Antisense RNA |
| TFF2 | Trefoil Factor 2 |
| ABCA3 | ATP Binding Cassette Subfamily A Member 3 |
| FEZF1-AS1 | FEZF1 Antisense RNA 1 |
| LINC00339 | Long Intergenic Non-Protein Coding RNA 339 |
| PTRH2 | Peptidyl-TRNA Hydrolase 2 |
| CCAT2 | Colon Cancer Associated Transcript 2 |
| CDA | Cytidine Deaminase |
| PRKAA2 | Protein Kinase AMP-Activated Catalytic Subunit Alpha 2 |
| TFAP2B | Transcription Factor AP-2 Beta |
| GLI3 | GLI Family Zinc Finger 3 |
| LOC389641 | Uncharacterized LOC389641 |
| CTTN | Cortactin |
| VCP | Valosin Containing Protein |
| CADM1 | Cell Adhesion Molecule 1 |
| DICER1 | Dicer 1, Ribonuclease III |
| GATA4 | GATA Binding Protein 4 |
| FGF8 | Fibroblast Growth Factor 8 |
| TNFRSF10D | TNF Receptor Superfamily Member 10d |
| PRSS3 | Serine Protease 3 |
| SULF2 | Sulfatase 2 |
| TNFRSF11B | TNF Receptor Superfamily Member 11b |
| ROR2 | Receptor Tyrosine Kinase Like Orphan Receptor 2 |
| MIR193A | MicroRNA 193a |
| DKK3 | Dickkopf WNT Signaling Pathway Inhibitor 3 |
| NEDD9 | Neural Precursor Cell Expressed, Developmentally Down-Regulated 9 |
| MIR138-1 | MicroRNA 138-1 |
| TF | Transferrin |
| TP53BP1 | Tumor Protein P53 Binding Protein 1 |
| PIM1 | Pim-1 Proto-Oncogene, Serine/Threonine Kinase |
| SFRP1 | Secreted Frizzled Related Protein 1 |
| SOS1 | SOS Ras/Rac Guanine Nucleotide Exchange Factor 1 |
| BAG1 | BAG Cochaperone 1 |
| PANDAR | Promoter Of CDKN1A Antisense DNA Damage Activated RNA |
| GNRH1 | Gonadotropin Releasing Hormone 1 |
| F2R | Coagulation Factor II Thrombin Receptor |
| ID1 | Inhibitor Of DNA Binding 1, HLH Protein |
| MAP2K7 | Mitogen-Activated Protein Kinase Kinase 7 |
| CLPTM1L | CLPTM1 Like |
| SELP | Selectin P |
| PIGR | Polymeric Immunoglobulin Receptor |
| HOXB9 | Homeobox B9 |
| NCOA1 | Nuclear Receptor Coactivator 1 |
| AQP3 | Aquaporin 3 (Gill Blood Group) |
| POLK | DNA Polymerase Kappa |
| PTPA | Protein Phosphatase 2 Phosphatase Activator |
| H2AX | H2A.X Variant Histone |
| GAD2 | Glutamate Decarboxylase 2 |
| EPHB4 | EPH Receptor B4 |
| PKD2 | Polycystin 2, Transient Receptor Potential Cation Channel |
| ESRRA | Estrogen Related Receptor Alpha |
| CDC25C | Cell Division Cycle 25C |
| MST1 | Macrophage Stimulating 1 |
| KDM1A | Lysine Demethylase 1A |
| CLDN1 | Claudin 1 |
| CDC20 | Cell Division Cycle 20 |
| IRF1 | Interferon Regulatory Factor 1 |
| CX3CR1 | C-X3-C Motif Chemokine Receptor 1 |
| APAF1 | Apoptotic Peptidase Activating Factor 1 |
| SDHB | Succinate Dehydrogenase Complex Iron Sulfur Subunit B |
| MIR503 | MicroRNA 503 |
| SHBG | Sex Hormone Binding Globulin |
| MIR101-1 | MicroRNA 101-1 |
| RAB11B | RAB11B, Member RAS Oncogene Family |
| EEF1A1 | Eukaryotic Translation Elongation Factor 1 Alpha 1 |
| DLK1 | Delta Like Non-Canonical Notch Ligand 1 |
| BTC | Betacellulin |
| MDK | Midkine |
| LYVE1 | Lymphatic Vessel Endothelial Hyaluronan Receptor 1 |
| MYLK | Myosin Light Chain Kinase |
| MAPK8IP1 | Mitogen-Activated Protein Kinase 8 Interacting Protein 1 |
| DUSP6 | Dual Specificity Phosphatase 6 |
| CTSE | Cathepsin E |
| TTR | Transthyretin |
| ROR1 | Receptor Tyrosine Kinase Like Orphan Receptor 1 |
| NUTM1 | NUT Midline Carcinoma Family Member 1 |
| CEP55 | Centrosomal Protein 55 |
| SLC39A1 | Solute Carrier Family 39 Member 1 |
| CXADR | CXADR Ig-Like Cell Adhesion Molecule |
| AQP1 | Aquaporin 1 (Colton Blood Group) |
| CDH17 | Cadherin 17 |
| ACACB | Acetyl-CoA Carboxylase Beta |
| TNC | Tenascin C |
| FABP4 | Fatty Acid Binding Protein 4 |
| PIK3R2 | Phosphoinositide-3-Kinase Regulatory Subunit 2 |
| ADAM9 | ADAM Metallopeptidase Domain 9 |
| PRKAA1 | Protein Kinase AMP-Activated Catalytic Subunit Alpha 1 |
| HSP90B1 | Heat Shock Protein 90 Beta Family Member 1 |
| BGLAP | Bone Gamma-Carboxyglutamate Protein |
| FST | Follistatin |
| GRN | Granulin Precursor |
| RAD52 | RAD52 Homolog, DNA Repair Protein |
| PARK7 | Parkinsonism Associated Deglycase |
| SOX4 | SRY-Box Transcription Factor 4 |
| CTHRC1 | Collagen Triple Helix Repeat Containing 1 |
| NR3C2 | Nuclear Receptor Subfamily 3 Group C Member 2 |
| HDAC6 | Histone Deacetylase 6 |
| MUC3A | Mucin 3A, Cell Surface Associated |
| MUC13 | Mucin 13, Cell Surface Associated |
| CDX1 | Caudal Type Homeobox 1 |
| PSMB5 | Proteasome 20S Subunit Beta 5 |
| CD59 | CD59 Molecule (CD59 Blood Group) |
| SPINT1 | Serine Peptidase Inhibitor, Kunitz Type 1 |
| HNRNPA2B1 | Heterogeneous Nuclear Ribonucleoprotein A2/B1 |
| L1CAM | L1 Cell Adhesion Molecule |
| UMPS | Uridine Monophosphate Synthetase |
| TNFRSF1B | TNF Receptor Superfamily Member 1B |
| FOXC1 | Forkhead Box C1 |
| ANGPT1 | Angiopoietin 1 |
| CYP27B1 | Cytochrome P450 Family 27 Subfamily B Member 1 |
| REG1B | Regenerating Family Member 1 Beta |
| ABCB5 | ATP Binding Cassette Subfamily B Member 5 |
| COL11A1 | Collagen Type XI Alpha 1 Chain |
| ADM | Adrenomedullin |
| MIR501 | MicroRNA 501 |
| TRPM8 | Transient Receptor Potential Cation Channel Subfamily M Member 8 |
| ASS1 | Argininosuccinate Synthase 1 |
| CASP1 | Caspase 1 |
| BMP7 | Bone Morphogenetic Protein 7 |
| PAX2 | Paired Box 2 |
| DIRC3 | Disrupted In Renal Carcinoma 3 |
| TFF3 | Trefoil Factor 3 |
| MIR27B | MicroRNA 27b |
| GUCA2A | Guanylate Cyclase Activator 2A |
| ACTN4 | Actinin Alpha 4 |
| WRN | WRN RecQ Like Helicase |
| ADCY10 | Adenylate Cyclase 10 |
| GPC1 | Glypican 1 |
| EGR1 | Early Growth Response 1 |
| ITGA2 | Integrin Subunit Alpha 2 |
| PSG2 | Pregnancy Specific Beta-1-Glycoprotein 2 |
| MMP10 | Matrix Metallopeptidase 10 |
| ALDOA | Aldolase, Fructose-Bisphosphate A |
| PRKCD | Protein Kinase C Delta |
| PPP3CA | Protein Phosphatase 3 Catalytic Subunit Alpha |
| TK1 | Thymidine Kinase 1 |
| ADAM19 | ADAM Metallopeptidase Domain 19 |
| PGF | Placental Growth Factor |
| CD55 | CD55 Molecule (Cromer Blood Group) |
| RAB8A | RAB8A, Member RAS Oncogene Family |
| PROK1 | Prokineticin 1 |
| MAPK9 | Mitogen-Activated Protein Kinase 9 |
| ROCK2 | Rho Associated Coiled-Coil Containing Protein Kinase 2 |
| EDNRB | Endothelin Receptor Type B |
| ADAM11 | ADAM Metallopeptidase Domain 11 |
| AGTR1 | Angiotensin II Receptor Type 1 |
| NOX4 | NADPH Oxidase 4 |
| ABCA12 | ATP Binding Cassette Subfamily A Member 12 |
| BCAR1 | BCAR1 Scaffold Protein, Cas Family Member |
| FGF4 | Fibroblast Growth Factor 4 |
| IL11 | Interleukin 11 |
| TH | Tyrosine Hydroxylase |
| JAK3 | Janus Kinase 3 |
| SAA1 | Serum Amyloid A1 |
| BRDT | Bromodomain Testis Associated |
| ATF4 | Activating Transcription Factor 4 |
| ACP1 | Acid Phosphatase 1 |
| GLUD1 | Glutamate Dehydrogenase 1 |
| SF3B1 | Splicing Factor 3b Subunit 1 |
| TFPI2 | Tissue Factor Pathway Inhibitor 2 |
| PRLR | Prolactin Receptor |
| TNFRSF10C | TNF Receptor Superfamily Member 10c |
| GIP | Gastric Inhibitory Polypeptide |
| BMPR2 | Bone Morphogenetic Protein Receptor Type 2 |
| ABCD2 | ATP Binding Cassette Subfamily D Member 2 |
| ACOT9 | Acyl-CoA Thioesterase 9 |
| SYDE1 | Synapse Defective Rho GTPase Homolog 1 |
| F2 | Coagulation Factor II, Thrombin |
| FBLN1 | Fibulin 1 |
| TUBB3 | Tubulin Beta 3 Class III |
| SNHG1 | Small Nucleolar RNA Host Gene 1 |
| DES | Desmin |
| AGT | Angiotensinogen |
| KLK6 | Kallikrein Related Peptidase 6 |
| MIAT | Myocardial Infarction Associated Transcript |
| HLA-B | Major Histocompatibility Complex, Class I, B |
| EPHA4 | EPH Receptor A4 |
| PSMB8 | Proteasome 20S Subunit Beta 8 |
| CX3CL1 | C-X3-C Motif Chemokine Ligand 1 |
| SDHC | Succinate Dehydrogenase Complex Subunit C |
| LINC01133 | Long Intergenic Non-Protein Coding RNA 1133 |
| PRKCZ | Protein Kinase C Zeta |
| NOD2 | Nucleotide Binding Oligomerization Domain Containing 2 |
| DANCR | Differentiation Antagonizing Non-Protein Coding RNA |
| XRCC6 | X-Ray Repair Cross Complementing 6 |
| SLC1A5 | Solute Carrier Family 1 Member 5 |
| LOXL2 | Lysyl Oxidase Like 2 |
| KLF5 | Kruppel Like Factor 5 |
| LIFR | LIF Receptor Subunit Alpha |
| S100A1 | S100 Calcium Binding Protein A1 |
| TLR7 | Toll Like Receptor 7 |
| PRSS8 | Serine Protease 8 |
| DLL4 | Delta Like Canonical Notch Ligand 4 |
| DNM2 | Dynamin 2 |
| HSPG2 | Heparan Sulfate Proteoglycan 2 |
| HLA-G | Major Histocompatibility Complex, Class I, G |
| VMP1 | Vacuole Membrane Protein 1 |
| FEZF1 | FEZ Family Zinc Finger 1 |
| ADAM12 | ADAM Metallopeptidase Domain 12 |
| MIR122 | MicroRNA 122 |
| GRB7 | Growth Factor Receptor Bound Protein 7 |
| VTCN1 | V-Set Domain Containing T Cell Activation Inhibitor 1 |
| GUSB | Glucuronidase Beta |
| SLC17A5 | Solute Carrier Family 17 Member 5 |
| NEK1 | NIMA Related Kinase 1 |
| CIP2A | Cellular Inhibitor Of PP2A |
| HOXA-AS2 | HOXA Cluster Antisense RNA 2 |
| SLC9A1 | Solute Carrier Family 9 Member A1 |
| MCM7 | Minichromosome Maintenance Complex Component 7 |
| TMPRSS2 | Transmembrane Serine Protease 2 |
| CYP2A6 | Cytochrome P450 Family 2 Subfamily A Member 6 |
| SPHK1 | Sphingosine Kinase 1 |
| NAMPT | Nicotinamide Phosphoribosyltransferase |
| NOG | Noggin |
| IL12A | Interleukin 12A |
| EPHA7 | EPH Receptor A7 |
| PSEN2 | Presenilin 2 |
| LAMA5 | Laminin Subunit Alpha 5 |
| TJP1 | Tight Junction Protein 1 |
| HOTAIRM1 | HOXA Transcript Antisense RNA, Myeloid-Specific 1 |
| ELK1 | ETS Transcription Factor ELK1 |
| IQGAP1 | IQ Motif Containing GTPase Activating Protein 1 |
| ENAH | ENAH Actin Regulator |
| CEMIP | Cell Migration Inducing Hyaluronidase 1 |
| STIM1 | Stromal Interaction Molecule 1 |
| SATB2 | SATB Homeobox 2 |
| NCOR2 | Nuclear Receptor Corepressor 2 |
| SMARCB1 | SWI/SNF Related, Matrix Associated, Actin Dependent Regulator Of Chromatin, Subfamily B, Member 1 |
| PTN | Pleiotrophin |
| KRT13 | Keratin 13 |
| TAP1 | Transporter 1, ATP Binding Cassette Subfamily B Member |
| MIR196A2 | MicroRNA 196a-2 |
| LINC00472 | Long Intergenic Non-Protein Coding RNA 472 |
| DAXX | Death Domain Associated Protein |
| IBSP | Integrin Binding Sialoprotein |
| TNK2 | Tyrosine Kinase Non Receptor 2 |
| FGF3 | Fibroblast Growth Factor 3 |
| MIR135A1 | MicroRNA 135a-1 |
| CTCF | CCCTC-Binding Factor |
| AKR1B10 | Aldo-Keto Reductase Family 1 Member B10 |
| TRPM7 | Transient Receptor Potential Cation Channel Subfamily M Member 7 |
| PML | Promyelocytic Leukemia |
| SNHG16 | Small Nucleolar RNA Host Gene 16 |
| GFAP | Glial Fibrillary Acidic Protein |
| CD80 | CD80 Molecule |
| P4HB | Prolyl 4-Hydroxylase Subunit Beta |
| TFRC | Transferrin Receptor |
| C9orf139 | Chromosome 9 Open Reading Frame 139 |
| ANXA10 | Annexin A10 |
| PRODH | Proline Dehydrogenase 1 |
| RACK1 | Receptor For Activated C Kinase 1 |
| MIR22 | MicroRNA 22 |
| PEG10 | Paternally Expressed 10 |
| AKR1C3 | Aldo-Keto Reductase Family 1 Member C3 |
| PTK6 | Protein Tyrosine Kinase 6 |
| MIR30B | MicroRNA 30b |
| ITGA5 | Integrin Subunit Alpha 5 |
| SATB1 | SATB Homeobox 1 |
| TJP2 | Tight Junction Protein 2 |
| TNFSF11 | TNF Superfamily Member 11 |
| KLK7 | Kallikrein Related Peptidase 7 |
| FOLR1 | Folate Receptor Alpha |
| EPHA5 | EPH Receptor A5 |
| STK4 | Serine/Threonine Kinase 4 |
| SEL1L | SEL1L Adaptor Subunit Of ERAD E3 Ubiquitin Ligase |
| ABL1 | ABL Proto-Oncogene 1, Non-Receptor Tyrosine Kinase |
| HOXB7 | Homeobox B7 |
| AHSG | Alpha 2-HS Glycoprotein |
| ZFHX3 | Zinc Finger Homeobox 3 |
| GLI2 | GLI Family Zinc Finger 2 |
| SERPINB2 | Serpin Family B Member 2 |
| PPP3CB | Protein Phosphatase 3 Catalytic Subunit Beta |
| MIR600HG | MIR600 Host Gene |
| ENSG00000250899 |  |
| RPL13AP23 | Ribosomal Protein L13a Pseudogene 23 |
| KMT2C | Lysine Methyltransferase 2C |
| GPRC5A | G Protein-Coupled Receptor Class C Group 5 Member A |
| NPPA | Natriuretic Peptide A |
| UBC | Ubiquitin C |
| NR5A2 | Nuclear Receptor Subfamily 5 Group A Member 2 |
| CSPP1 | Centrosome And Spindle Pole Associated Protein 1 |
| TACSTD2 | Tumor Associated Calcium Signal Transducer 2 |
| ABO | ABO, Alpha 1-3-N-Acetylgalactosaminyltransferase And Alpha 1-3-Galactosyltransferase |
| ACTG1 | Actin Gamma 1 |
| PSMD5 | Proteasome 26S Subunit, Non-ATPase 5 |
| MSN | Moesin |
| SUFU | SUFU Negative Regulator Of Hedgehog Signaling |
| EIF2AK2 | Eukaryotic Translation Initiation Factor 2 Alpha Kinase 2 |
| NRP2 | Neuropilin 2 |
| CAMP | Cathelicidin Antimicrobial Peptide |
| STC2 | Stanniocalcin 2 |
| IKBKE | Inhibitor Of Nuclear Factor Kappa B Kinase Subunit Epsilon |
| MIR186 | MicroRNA 186 |
| GAL | Galanin And GMAP Prepropeptide |
| FOXP1 | Forkhead Box P1 |
| CPS1 | Carbamoyl-Phosphate Synthase 1 |
| PI3 | Peptidase Inhibitor 3 |
| S100A7 | S100 Calcium Binding Protein A7 |
| IGFBP7 | Insulin Like Growth Factor Binding Protein 7 |
| CLDN2 | Claudin 2 |
| TRIM28 | Tripartite Motif Containing 28 |
| MICA | MHC Class I Polypeptide-Related Sequence A |
| MAGEA4 | MAGE Family Member A4 |
| CDC6 | Cell Division Cycle 6 |
| CD86 | CD86 Molecule |
| BIRC7 | Baculoviral IAP Repeat Containing 7 |
| COL18A1 | Collagen Type XVIII Alpha 1 Chain |
| HPGD | 15-Hydroxyprostaglandin Dehydrogenase |
| RPL15 | Ribosomal Protein L15 |
| TPX2 | TPX2 Microtubule Nucleation Factor |
| DRAIC | Downregulated RNA In Cancer, Inhibitor Of Cell Invasion And Migration |
| FGF5 | Fibroblast Growth Factor 5 |
| LTA | Lymphotoxin Alpha |
| ALPP | Alkaline Phosphatase, Placental |
| TULP3 | TUB Like Protein 3 |
| CA4 | Carbonic Anhydrase 4 |
| HDGF | Heparin Binding Growth Factor |
| CD9 | CD9 Molecule |
| IL15 | Interleukin 15 |
| GAD1 | Glutamate Decarboxylase 1 |
| CLCA1 | Chloride Channel Accessory 1 |
| AMBP | Alpha-1-Microglobulin/Bikunin Precursor |
| POMC | Proopiomelanocortin |
| EGLN3 | Egl-9 Family Hypoxia Inducible Factor 3 |
| MIR320A | MicroRNA 320a |
| STUB1 | STIP1 Homology And U-Box Containing Protein 1 |
| PROX1 | Prospero Homeobox 1 |
| ITGB3 | Integrin Subunit Beta 3 |
| DNAJC21 | DnaJ Heat Shock Protein Family (Hsp40) Member C21 |
| PIM3 | Pim-3 Proto-Oncogene, Serine/Threonine Kinase |
| RAN | RAN, Member RAS Oncogene Family |
| ADCYAP1 | Adenylate Cyclase Activating Polypeptide 1 |
| P2RY2 | Purinergic Receptor P2Y2 |
| NR2F2 | Nuclear Receptor Subfamily 2 Group F Member 2 |
| PTTG1 | PTTG1 Regulator Of Sister Chromatid Separation, Securin |
| MCAM | Melanoma Cell Adhesion Molecule |
| EMX2 | Empty Spiracles Homeobox 2 |
| TLR5 | Toll Like Receptor 5 |
| TAGLN2 | Transgelin 2 |
| IL4R | Interleukin 4 Receptor |
| MIR33A | MicroRNA 33a |
| USP9X | Ubiquitin Specific Peptidase 9 X-Linked |
| PTPRH | Protein Tyrosine Phosphatase Receptor Type H |
| LGALS4 | Galectin 4 |
| EDN2 | Endothelin 2 |
| ATF3 | Activating Transcription Factor 3 |
| BBC3 | BCL2 Binding Component 3 |
| MIR29B1 | MicroRNA 29b-1 |
| SPOP | Speckle Type BTB/POZ Protein |
| CEBPB | CCAAT Enhancer Binding Protein Beta |
| CD151 | CD151 Molecule (Raph Blood Group) |
| IHH | Indian Hedgehog Signaling Molecule |
| BAG3 | BAG Cochaperone 3 |
| CYP24A1 | Cytochrome P450 Family 24 Subfamily A Member 1 |
| PTGER4 | Prostaglandin E Receptor 4 |
| ARTN | Artemin |
| MIR296 | MicroRNA 296 |
| NDUFS3 | NADH:Ubiquinone Oxidoreductase Core Subunit S3 |
| PSMC4 | Proteasome 26S Subunit, ATPase 4 |
| MAD2L1 | Mitotic Arrest Deficient 2 Like 1 |
| HMMR | Hyaluronan Mediated Motility Receptor |
| HHEX | Hematopoietically Expressed Homeobox |
| MIR449A | MicroRNA 449a |
| WNT3A | Wnt Family Member 3A |
| S100A10 | S100 Calcium Binding Protein A10 |
| DAB2 | DAB Adaptor Protein 2 |
| NCL | Nucleolin |
| MIR132 | MicroRNA 132 |
| TLR3 | Toll Like Receptor 3 |
| MTAP | Methylthioadenosine Phosphorylase |
| MIR7-1 | MicroRNA 7-1 |
| TGFBI | Transforming Growth Factor Beta Induced |
| ARSH | Arylsulfatase Family Member H |
| FH | Fumarate Hydratase |
| PNPLA2 | Patatin Like Phospholipase Domain Containing 2 |
| SNHG7 | Small Nucleolar RNA Host Gene 7 |
| C3 | Complement C3 |
| CFL1 | Cofilin 1 |
| CELA3A | Chymotrypsin Like Elastase 3A |
| PODXL | Podocalyxin Like |
| SULT1A1 | Sulfotransferase Family 1A Member 1 |
| MIR377 | MicroRNA 377 |
| VDAC1 | Voltage Dependent Anion Channel 1 |
| CP | Ceruloplasmin |
| RREB1 | Ras Responsive Element Binding Protein 1 |
| RHEB | Ras Homolog, MTORC1 Binding |
| NCOA2 | Nuclear Receptor Coactivator 2 |
| KLK1 | Kallikrein 1 |
| WEE1 | WEE1 G2 Checkpoint Kinase |
| PPP1R1B | Protein Phosphatase 1 Regulatory Inhibitor Subunit 1B |
| CHI3L1 | Chitinase 3 Like 1 |
| PC | Pyruvate Carboxylase |
| IGF2BP2 | Insulin Like Growth Factor 2 MRNA Binding Protein 2 |
| NTF3 | Neurotrophin 3 |
| ABCC4 | ATP Binding Cassette Subfamily C Member 4 |
| LSM1 | LSM1 Homolog, MRNA Degradation Associated |
| CCL18 | C-C Motif Chemokine Ligand 18 |
| MYB | MYB Proto-Oncogene, Transcription Factor |
| NPM1 | Nucleophosmin 1 |
| PBX1 | PBX Homeobox 1 |
| CGB3 | Chorionic Gonadotropin Subunit Beta 3 |
| RHO | Rhodopsin |
| TDG | Thymine DNA Glycosylase |
| TXNIP | Thioredoxin Interacting Protein |
| MSI1 | Musashi RNA Binding Protein 1 |
| MARK4 | Microtubule Affinity Regulating Kinase 4 |
| FBP1 | Fructose-Bisphosphatase 1 |
| TIMELESS | Timeless Circadian Regulator |
| COX5A | Cytochrome C Oxidase Subunit 5A |
| RARG | Retinoic Acid Receptor Gamma |
| PRDX3 | Peroxiredoxin 3 |
| GAS6 | Growth Arrest Specific 6 |
| KLF9 | Kruppel Like Factor 9 |
| JUND | JunD Proto-Oncogene, AP-1 Transcription Factor Subunit |
| LASP1 | LIM And SH3 Protein 1 |
| MIR184 | MicroRNA 184 |
| AGTR2 | Angiotensin II Receptor Type 2 |
| KAT2B | Lysine Acetyltransferase 2B |
| PLXNB1 | Plexin B1 |
| CD46 | CD46 Molecule |
| CXCR3 | C-X-C Motif Chemokine Receptor 3 |
| PPARA | Peroxisome Proliferator Activated Receptor Alpha |
| PTGES | Prostaglandin E Synthase |
| CSN1S1 | Casein Alpha S1 |
| TRAF6 | TNF Receptor Associated Factor 6 |
| GHRHR | Growth Hormone Releasing Hormone Receptor |
| CD276 | CD276 Molecule |
| KL | Klotho |
| HPN | Hepsin |
| AKR1A1 | Aldo-Keto Reductase Family 1 Member A1 |
| P2RX7 | Purinergic Receptor P2X 7 |
| G6PD | Glucose-6-Phosphate Dehydrogenase |
| CD14 | CD14 Molecule |
| GPX1 | Glutathione Peroxidase 1 |
| MIR219A1 | MicroRNA 219a-1 |
| WIF1 | WNT Inhibitory Factor 1 |
| PIN1 | Peptidylprolyl Cis/Trans Isomerase, NIMA-Interacting 1 |
| IGFBP4 | Insulin Like Growth Factor Binding Protein 4 |
| LPXN | Leupaxin |
| SLC16A4 | Solute Carrier Family 16 Member 4 |
| PAWR | Pro-Apoptotic WT1 Regulator |
| TP73-AS1 | TP73 Antisense RNA 1 |
| IL13RA2 | Interleukin 13 Receptor Subunit Alpha 2 |
| THBS2 | Thrombospondin 2 |
| LRP1 | LDL Receptor Related Protein 1 |
| SPRY2 | Sprouty RTK Signaling Antagonist 2 |
| PGK1 | Phosphoglycerate Kinase 1 |
| ULK1 | Unc-51 Like Autophagy Activating Kinase 1 |
| DEFB1 | Defensin Beta 1 |
| KRT4 | Keratin 4 |
| TCF7 | Transcription Factor 7 |
| SQSTM1 | Sequestosome 1 |
| TTK | TTK Protein Kinase |
| MARK3 | Microtubule Affinity Regulating Kinase 3 |
| ING1 | Inhibitor Of Growth Family Member 1 |
| DCN | Decorin |
| CCL20 | C-C Motif Chemokine Ligand 20 |
| MIR26B | MicroRNA 26b |
| LGALS3BP | Galectin 3 Binding Protein |
| NEU1 | Neuraminidase 1 |
| BRMS1 | BRMS1 Transcriptional Repressor And Anoikis Regulator |
| ATRX | ATRX Chromatin Remodeler |
| RPSA | Ribosomal Protein SA |
| BPI | Bactericidal Permeability Increasing Protein |
| ID2 | Inhibitor Of DNA Binding 2 |
| MARK1 | Microtubule Affinity Regulating Kinase 1 |
| CSNK2A1 | Casein Kinase 2 Alpha 1 |
| MIR218-1 | MicroRNA 218-1 |
| RNF2 | Ring Finger Protein 2 |
| ALDH9A1 | Aldehyde Dehydrogenase 9 Family Member A1 |
| PDHX | Pyruvate Dehydrogenase Complex Component X |
| FUT2 | Fucosyltransferase 2 |
| HLA-C | Major Histocompatibility Complex, Class I, C |
| DIRAS3 | DIRAS Family GTPase 3 |
| TMPRSS4 | Transmembrane Serine Protease 4 |
| KCNMA1 | Potassium Calcium-Activated Channel Subfamily M Alpha 1 |
| PIK3C2A | Phosphatidylinositol-4-Phosphate 3-Kinase Catalytic Subunit Type 2 Alpha |
| ANP32A | Acidic Nuclear Phosphoprotein 32 Family Member A |
| RBP1 | Retinol Binding Protein 1 |
| MAP4K4 | Mitogen-Activated Protein Kinase Kinase Kinase Kinase 4 |
| PLIN1 | Perilipin 1 |
| CCL4 | C-C Motif Chemokine Ligand 4 |
| SND1 | Staphylococcal Nuclease And Tudor Domain Containing 1 |
| PSMA6 | Proteasome 20S Subunit Alpha 6 |
| TFAM | Transcription Factor A, Mitochondrial |
| GNA11 | G Protein Subunit Alpha 11 |
| PDIA3 | Protein Disulfide Isomerase Family A Member 3 |
| MAP1LC3B | Microtubule Associated Protein 1 Light Chain 3 Beta |
| FUT3 | Fucosyltransferase 3 (Lewis Blood Group) |
| TCHP | Trichoplein Keratin Filament Binding |
| DEFB4A | Defensin Beta 4A |
| PRKCI | Protein Kinase C Iota |
| IL33 | Interleukin 33 |
| WNT2 | Wnt Family Member 2 |
| MIR124-2 | MicroRNA 124-2 |
| BLM | BLM RecQ Like Helicase |
| PAF1 | PAF1 Homolog, Paf1/RNA Polymerase II Complex Component |
| HSPH1 | Heat Shock Protein Family H (Hsp110) Member 1 |
| XAGE1B | X Antigen Family Member 1B |
| IL22 | Interleukin 22 |
| TNFSF13B | TNF Superfamily Member 13b |
| KCNH2 | Potassium Voltage-Gated Channel Subfamily H Member 2 |
| TRPM2 | Transient Receptor Potential Cation Channel Subfamily M Member 2 |
| AXIN1 | Axin 1 |
| HIC1 | HIC ZBTB Transcriptional Repressor 1 |
| HSP90AB1 | Heat Shock Protein 90 Alpha Family Class B Member 1 |
| CUX1 | Cut Like Homeobox 1 |
| NMB | Neuromedin B |
| NTN1 | Netrin 1 |
| PLAGL1 | PLAG1 Like Zinc Finger 1 |
| SLC9A3 | Solute Carrier Family 9 Member A3 |
| ANXA4 | Annexin A4 |
| TCF3 | Transcription Factor 3 |
| CTRL | Chymotrypsin Like |
| AQP5 | Aquaporin 5 |
| ISG15 | ISG15 Ubiquitin Like Modifier |
| PRDM2 | PR/SET Domain 2 |
| MICB | MHC Class I Polypeptide-Related Sequence B |
| GPC5 | Glypican 5 |
| MIR361 | MicroRNA 361 |
| ADAM17 | ADAM Metallopeptidase Domain 17 |
| HNRNPK | Heterogeneous Nuclear Ribonucleoprotein K |
| TGFBR3 | Transforming Growth Factor Beta Receptor 3 |
| MIA2 | MIA SH3 Domain ER Export Factor 2 |
| KCNJ1 | Potassium Inwardly Rectifying Channel Subfamily J Member 1 |
| LGR5 | Leucine Rich Repeat Containing G Protein-Coupled Receptor 5 |
| PLIN2 | Perilipin 2 |
| CLIC1 | Chloride Intracellular Channel 1 |
| SERPINF1 | Serpin Family F Member 1 |
| CCNB2 | Cyclin B2 |
| LAMB1 | Laminin Subunit Beta 1 |
| TMPRSS11A | Transmembrane Serine Protease 11A |
| ITGB6 | Integrin Subunit Beta 6 |
| SIRT3 | Sirtuin 3 |
| HDAC3 | Histone Deacetylase 3 |
| RIPK1 | Receptor Interacting Serine/Threonine Kinase 1 |
| PDCD1LG2 | Programmed Cell Death 1 Ligand 2 |
| WASF2 | WASP Family Member 2 |
| ACE2 | Angiotensin I Converting Enzyme 2 |
| XAGE1A | X Antigen Family Member 1A |
| FURIN | Furin, Paired Basic Amino Acid Cleaving Enzyme |
| HSD17B1 | Hydroxysteroid 17-Beta Dehydrogenase 1 |
| HSPA9 | Heat Shock Protein Family A (Hsp70) Member 9 |
| ADGRE5 | Adhesion G Protein-Coupled Receptor E5 |
| EPHA1 | EPH Receptor A1 |
| TRAF2 | TNF Receptor Associated Factor 2 |
| WNT10B | Wnt Family Member 10B |
| RHOD | Ras Homolog Family Member D |
| APOA1 | Apolipoprotein A1 |
| SOD3 | Superoxide Dismutase 3 |
| HHLA2 | HERV-H LTR-Associating 2 |
| BHLHE40 | Basic Helix-Loop-Helix Family Member E40 |
| S100A11 | S100 Calcium Binding Protein A11 |
| NODAL | Nodal Growth Differentiation Factor |
| ST6GAL1 | ST6 Beta-Galactoside Alpha-2,6-Sialyltransferase 1 |
| PRKD2 | Protein Kinase D2 |
| MAP3K8 | Mitogen-Activated Protein Kinase Kinase Kinase 8 |
| PRKACA | Protein Kinase CAMP-Activated Catalytic Subunit Alpha |
| MAGEA1 | MAGE Family Member A1 |
| UBE2C | Ubiquitin Conjugating Enzyme E2 C |
| LOC111099027 | Transmembrane Protease Serine 2 Breakpoint Cluster Recombination Region |
| LOC111099028 | ERG, ETS Transcription Factor Breakpoint Cluster Recombination Region |
| SLC3A2 | Solute Carrier Family 3 Member 2 |
| MIR506 | MicroRNA 506 |
| CD99 | CD99 Molecule (Xg Blood Group) |
| PRDX1 | Peroxiredoxin 1 |
| LIF | LIF Interleukin 6 Family Cytokine |
| SULT2A1 | Sulfotransferase Family 2A Member 1 |
| CBS | Cystathionine Beta-Synthase |
| AMY1A | Amylase Alpha 1A |
| TFAP2C | Transcription Factor AP-2 Gamma |
| ADAMTSL1 | ADAMTS Like 1 |
| TACC1 | Transforming Acidic Coiled-Coil Containing Protein 1 |
| CCR5 | C-C Motif Chemokine Receptor 5 (Gene/Pseudogene) |
| CCL3 | C-C Motif Chemokine Ligand 3 |
| LIN28B | Lin-28 Homolog B |
| TIAM1 | TIAM Rac1 Associated GEF 1 |
| SELL | Selectin L |
| COL5A1 | Collagen Type V Alpha 1 Chain |
| TIE1 | Tyrosine Kinase With Immunoglobulin Like And EGF Like Domains 1 |
| ASPN | Asporin |
| EPB41L3 | Erythrocyte Membrane Protein Band 4.1 Like 3 |
| EMP2 | Epithelial Membrane Protein 2 |
| MIR708 | MicroRNA 708 |
| CASP6 | Caspase 6 |
| SMAD6 | SMAD Family Member 6 |
| RDX | Radixin |
| RBX1 | Ring-Box 1 |
| UGT1A1 | UDP Glucuronosyltransferase Family 1 Member A1 |
| PPIA | Peptidylprolyl Isomerase A |
| PTGER3 | Prostaglandin E Receptor 3 |
| FUT8 | Fucosyltransferase 8 |
| CTSK | Cathepsin K |
| TM4SF1 | Transmembrane 4 L Six Family Member 1 |
| PITX2 | Paired Like Homeodomain 2 |
| SEMA3A | Semaphorin 3A |
| GNRHR | Gonadotropin Releasing Hormone Receptor |
| PON1 | Paraoxonase 1 |
| APP | Amyloid Beta Precursor Protein |
| RBPJ | Recombination Signal Binding Protein For Immunoglobulin Kappa J Region |
| MMP8 | Matrix Metallopeptidase 8 |
| NOX1 | NADPH Oxidase 1 |
| CDH5 | Cadherin 5 |
| VASH2 | Vasohibin 2 |
| SIX1 | SIX Homeobox 1 |
| CBL | Cbl Proto-Oncogene |
| KLK11 | Kallikrein Related Peptidase 11 |
| MAP3K5 | Mitogen-Activated Protein Kinase Kinase Kinase 5 |
| FABP3 | Fatty Acid Binding Protein 3 |
| ARNTL | Aryl Hydrocarbon Receptor Nuclear Translocator Like |
| LRP6 | LDL Receptor Related Protein 6 |
| MIR337 | MicroRNA 337 |
| NCOR1 | Nuclear Receptor Corepressor 1 |
| PRKDC | Protein Kinase, DNA-Activated, Catalytic Subunit |
| MUC7 | Mucin 7, Secreted |
| CTNNBIP1 | Catenin Beta Interacting Protein 1 |
| H4-16 | H4 Histone 16 |
| GOT1 | Glutamic-Oxaloacetic Transaminase 1 |
| RAPGEF3 | Rap Guanine Nucleotide Exchange Factor 3 |
| DYRK1B | Dual Specificity Tyrosine Phosphorylation Regulated Kinase 1B |
| ACTA2 | Actin Alpha 2, Smooth Muscle |
| KLK4 | Kallikrein Related Peptidase 4 |
| LAMB3 | Laminin Subunit Beta 3 |
| DSG3 | Desmoglein 3 |
| PSMD11 | Proteasome 26S Subunit, Non-ATPase 11 |
| SYK | Spleen Associated Tyrosine Kinase |
| PAEP | Progestagen Associated Endometrial Protein |
| VIPR1 | Vasoactive Intestinal Peptide Receptor 1 |
| FLT3 | Fms Related Receptor Tyrosine Kinase 3 |
| NUMB | NUMB Endocytic Adaptor Protein |
| MAP3K7 | Mitogen-Activated Protein Kinase Kinase Kinase 7 |
| PEA15 | Proliferation And Apoptosis Adaptor Protein 15 |
| STK11IP | Serine/Threonine Kinase 11 Interacting Protein |
| SOX18 | SRY-Box Transcription Factor 18 |
| LPAR1 | Lysophosphatidic Acid Receptor 1 |
| ERCC4 | ERCC Excision Repair 4, Endonuclease Catalytic Subunit |
| INPPL1 | Inositol Polyphosphate Phosphatase Like 1 |
| CBARP | CACN Subunit Beta Associated Regulatory Protein |
| ID3 | Inhibitor Of DNA Binding 3, HLH Protein |
| PSMD2 | Proteasome 26S Subunit, Non-ATPase 2 |
| MB | Myoglobin |
| DACH1 | Dachshund Family Transcription Factor 1 |
| FAM3B | FAM3 Metabolism Regulating Signaling Molecule B |
| LLGL2 | LLGL Scribble Cell Polarity Complex Component 2 |
| MTA2 | Metastasis Associated 1 Family Member 2 |
| CCR9 | C-C Motif Chemokine Receptor 9 |
| ERP29 | Endoplasmic Reticulum Protein 29 |
| IL7 | Interleukin 7 |
| PDIA2 | Protein Disulfide Isomerase Family A Member 2 |
| GZMB | Granzyme B |
| CBLB | Cbl Proto-Oncogene B |
| USP22 | Ubiquitin Specific Peptidase 22 |
| NR4A1 | Nuclear Receptor Subfamily 4 Group A Member 1 |
| EGOT | Eosinophil Granule Ontogeny Transcript |
| GJB2 | Gap Junction Protein Beta 2 |
| RPS27A | Ribosomal Protein S27a |
| NPTX2 | Neuronal Pentraxin 2 |
| PF4 | Platelet Factor 4 |
| MDC1 | Mediator Of DNA Damage Checkpoint 1 |
| EPS8 | Epidermal Growth Factor Receptor Pathway Substrate 8 |
| CD63 | CD63 Molecule |
| ELF3 | E74 Like ETS Transcription Factor 3 |
| SLC5A8 | Solute Carrier Family 5 Member 8 |
| ETS2 | ETS Proto-Oncogene 2, Transcription Factor |
| PRKG2 | Protein Kinase CGMP-Dependent 2 |
| MUCL1 | Mucin Like 1 |
| LOC110006317 | Serine/Threonine Kinase 11 Intron 1 Alu-Mediated Recombination Region |
| LOC110006318 | Serine/Threonine Kinase 11 Intron 3 Alu-Mediated Recombination Region |
| FABP6 | Fatty Acid Binding Protein 6 |
| BTRC | Beta-Transducin Repeat Containing E3 Ubiquitin Protein Ligase |
| TFE3 | Transcription Factor Binding To IGHM Enhancer 3 |
| SH3GLB1 | SH3 Domain Containing GRB2 Like, Endophilin B1 |
| PTGER2 | Prostaglandin E Receptor 2 |
| SLC28A3 | Solute Carrier Family 28 Member 3 |
| EIF2S1 | Eukaryotic Translation Initiation Factor 2 Subunit Alpha |
| VAV3 | Vav Guanine Nucleotide Exchange Factor 3 |
| CCL22 | C-C Motif Chemokine Ligand 22 |
| CRABP2 | Cellular Retinoic Acid Binding Protein 2 |
| CA12 | Carbonic Anhydrase 12 |
| CSTA | Cystatin A |
| VCL | Vinculin |
| CRISP3 | Cysteine Rich Secretory Protein 3 |
| CHRNA7 | Cholinergic Receptor Nicotinic Alpha 7 Subunit |
| ANO1 | Anoctamin 1 |
| ATG5 | Autophagy Related 5 |
| PLCB1 | Phospholipase C Beta 1 |
| PHLPP1 | PH Domain And Leucine Rich Repeat Protein Phosphatase 1 |
| CD68 | CD68 Molecule |
| HSPE1 | Heat Shock Protein Family E (Hsp10) Member 1 |
| CYP2A13 | Cytochrome P450 Family 2 Subfamily A Member 13 |
| CLDN5 | Claudin 5 |
| PSMC6 | Proteasome 26S Subunit, ATPase 6 |
| GJB1 | Gap Junction Protein Beta 1 |
| PELP1 | Proline, Glutamate And Leucine Rich Protein 1 |
| LAPTM4B | Lysosomal Protein Transmembrane 4 Beta |
| RPRM | Reprimo, TP53 Dependent G2 Arrest Mediator Homolog |
| NNMT | Nicotinamide N-Methyltransferase |
| STEAP3 | STEAP3 Metalloreductase |
| SSBP3 | Single Stranded DNA Binding Protein 3 |
| TSPAN32 | Tetraspanin 32 |
| TRIM21 | Tripartite Motif Containing 21 |
| PPP2R1A | Protein Phosphatase 2 Scaffold Subunit Aalpha |
| PLD1 | Phospholipase D1 |
| KRT1 | Keratin 1 |
| GSR | Glutathione-Disulfide Reductase |
| MIR133B | MicroRNA 133b |
| BCL9L | BCL9 Like |
| GOLM1 | Golgi Membrane Protein 1 |
| NACC1 | Nucleus Accumbens Associated 1 |
| SLCO1B3 | Solute Carrier Organic Anion Transporter Family Member 1B3 |
| NEK9 | NIMA Related Kinase 9 |
| SEC23B | SEC23 Homolog B, COPII Coat Complex Component |
| TPBG | Trophoblast Glycoprotein |
| CDH11 | Cadherin 11 |
| F5 | Coagulation Factor V |
| DLC1 | DLC1 Rho GTPase Activating Protein |
| MECP2 | Methyl-CpG Binding Protein 2 |
| RCN1 | Reticulocalbin 1 |
| PFN1 | Profilin 1 |
| APOD | Apolipoprotein D |
| GALNT3 | Polypeptide N-Acetylgalactosaminyltransferase 3 |
| FZD5 | Frizzled Class Receptor 5 |
| GPX3 | Glutathione Peroxidase 3 |
| FXYD5 | FXYD Domain Containing Ion Transport Regulator 5 |
| IDH2 | Isocitrate Dehydrogenase (NADP(+)) 2 |
| DSG2 | Desmoglein 2 |
| BRSK2 | BR Serine/Threonine Kinase 2 |
| HTATIP2 | HIV-1 Tat Interactive Protein 2 |
| KNG1 | Kininogen 1 |
| LPO | Lactoperoxidase |
| ADAM8 | ADAM Metallopeptidase Domain 8 |
| ZFP36 | ZFP36 Ring Finger Protein |
| PRDX4 | Peroxiredoxin 4 |
| CDK5 | Cyclin Dependent Kinase 5 |
| JAG2 | Jagged Canonical Notch Ligand 2 |
| LUM | Lumican |
| HDC | Histidine Decarboxylase |
| STEAP4 | STEAP4 Metalloreductase |
| MIR133A1 | MicroRNA 133a-1 |
| CCL28 | C-C Motif Chemokine Ligand 28 |
| KIF14 | Kinesin Family Member 14 |
| FABP2 | Fatty Acid Binding Protein 2 |
| ILF2 | Interleukin Enhancer Binding Factor 2 |
| ENPP2 | Ectonucleotide Pyrophosphatase/Phosphodiesterase 2 |
| PSMA1 | Proteasome 20S Subunit Alpha 1 |
| ADIPOR1 | Adiponectin Receptor 1 |
| TMEFF2 | Transmembrane Protein With EGF Like And Two Follistatin Like Domains 2 |
| NDRG2 | NDRG Family Member 2 |
| CCN4 | Cellular Communication Network Factor 4 |
| PYGM | Glycogen Phosphorylase, Muscle Associated |
| TUBA1B | Tubulin Alpha 1b |
| SLC39A6 | Solute Carrier Family 39 Member 6 |
| GRM1 | Glutamate Metabotropic Receptor 1 |
| ACKR2 | Atypical Chemokine Receptor 2 |
| NSD1 | Nuclear Receptor Binding SET Domain Protein 1 |
| PAK4 | P21 (RAC1) Activated Kinase 4 |
| CYP3A5 | Cytochrome P450 Family 3 Subfamily A Member 5 |
| FXYD3 | FXYD Domain Containing Ion Transport Regulator 3 |
| MIR675 | MicroRNA 675 |
| MTRR | 5-Methyltetrahydrofolate-Homocysteine Methyltransferase Reductase |
| MMP19 | Matrix Metallopeptidase 19 |
| MIR455 | MicroRNA 455 |
| MIR1290 | MicroRNA 1290 |
| APPL1 | Adaptor Protein, Phosphotyrosine Interacting With PH Domain And Leucine Zipper 1 |
| DEK | DEK Proto-Oncogene |
| MIR152 | MicroRNA 152 |
| PSMB9 | Proteasome 20S Subunit Beta 9 |
| HDAC4 | Histone Deacetylase 4 |
| LIMS1 | LIM Zinc Finger Domain Containing 1 |
| TAGLN | Transgelin |
| ID4 | Inhibitor Of DNA Binding 4, HLH Protein |
| LIMK1 | LIM Domain Kinase 1 |
| DAB2IP | DAB2 Interacting Protein |
| RYR1 | Ryanodine Receptor 1 |
| MIR448 | MicroRNA 448 |
| HAVCR2 | Hepatitis A Virus Cellular Receptor 2 |
| SERPINC1 | Serpin Family C Member 1 |
| TNS4 | Tensin 4 |
| PRNP | Prion Protein |
| GPI | Glucose-6-Phosphate Isomerase |
| NMBR | Neuromedin B Receptor |
| EPHA8 | EPH Receptor A8 |
| MTSS1 | MTSS I-BAR Domain Containing 1 |
| RBP4 | Retinol Binding Protein 4 |
| NLRP3 | NLR Family Pyrin Domain Containing 3 |
| MYD88 | MYD88 Innate Immune Signal Transduction Adaptor |
| FOXC2 | Forkhead Box C2 |
| GPX4 | Glutathione Peroxidase 4 |
| NSD3 | Nuclear Receptor Binding SET Domain Protein 3 |
| MKKS | McKusick-Kaufman Syndrome |
| SLC39A3 | Solute Carrier Family 39 Member 3 |
| PNP | Purine Nucleoside Phosphorylase |
| DUSP19 | Dual Specificity Phosphatase 19 |
| SKIL | SKI Like Proto-Oncogene |
| KISS1R | KISS1 Receptor |
| CRK | CRK Proto-Oncogene, Adaptor Protein |
| RUNX1 | RUNX Family Transcription Factor 1 |
| ATP6AP2 | ATPase H+ Transporting Accessory Protein 2 |
| MIR326 | MicroRNA 326 |
| AMBRA1 | Autophagy And Beclin 1 Regulator 1 |
| THY1 | Thy-1 Cell Surface Antigen |
| IKBKG | Inhibitor Of Nuclear Factor Kappa B Kinase Regulatory Subunit Gamma |
| ADIPOR2 | Adiponectin Receptor 2 |
| SOX10 | SRY-Box Transcription Factor 10 |
| LPAR2 | Lysophosphatidic Acid Receptor 2 |
| EHMT2 | Euchromatic Histone Lysine Methyltransferase 2 |
| RNY1 | RNA, Ro60-Associated Y1 |
| SLC12A2 | Solute Carrier Family 12 Member 2 |
| ACVR1 | Activin A Receptor Type 1 |
| AKR7A2 | Aldo-Keto Reductase Family 7 Member A2 |
| PSAT1 | Phosphoserine Aminotransferase 1 |
| CST3 | Cystatin C |
| ACVRL1 | Activin A Receptor Like Type 1 |
| KLK2 | Kallikrein Related Peptidase 2 |
| PLEC | Plectin |
| KCNJ3 | Potassium Inwardly Rectifying Channel Subfamily J Member 3 |
| ARFGEF3 | ARFGEF Family Member 3 |
| YES1 | YES Proto-Oncogene 1, Src Family Tyrosine Kinase |
| SLC28A1 | Solute Carrier Family 28 Member 1 |
| NCOA4 | Nuclear Receptor Coactivator 4 |
| LAMP1 | Lysosomal Associated Membrane Protein 1 |
| CXCL16 | C-X-C Motif Chemokine Ligand 16 |
| EPHX2 | Epoxide Hydrolase 2 |
| TP53TG1 | TP53 Target 1 |
| MIR454 | MicroRNA 454 |
| HHIP | Hedgehog Interacting Protein |
| EGLN1 | Egl-9 Family Hypoxia Inducible Factor 1 |
| CYLD | CYLD Lysine 63 Deubiquitinase |
| LINC00958 | Long Intergenic Non-Protein Coding RNA 958 |
| TCF7L1 | Transcription Factor 7 Like 1 |
| PHGDH | Phosphoglycerate Dehydrogenase |
| RECQL | RecQ Like Helicase |
| LINC01194 | Long Intergenic Non-Protein Coding RNA 1194 |
| FZD1 | Frizzled Class Receptor 1 |
| MMP20 | Matrix Metallopeptidase 20 |
| CRTC3 | CREB Regulated Transcription Coactivator 3 |
| EBI3 | Epstein-Barr Virus Induced 3 |
| SAGE1 | Sarcoma Antigen 1 |
| QSOX1 | Quiescin Sulfhydryl Oxidase 1 |
| RAB27B | RAB27B, Member RAS Oncogene Family |
| FKBP5 | FKBP Prolyl Isomerase 5 |
| ARHGEF15 | Rho Guanine Nucleotide Exchange Factor 15 |
| IFIT3 | Interferon Induced Protein With Tetratricopeptide Repeats 3 |
| XDH | Xanthine Dehydrogenase |
| ACTB | Actin Beta |
| HSPB2 | Heat Shock Protein Family B (Small) Member 2 |
| YWHAQ | Tyrosine 3-Monooxygenase/Tryptophan 5-Monooxygenase Activation Protein Theta |
| MNX1-AS1 | MNX1 Antisense RNA 1 (Head To Head) |
| GABBR1 | Gamma-Aminobutyric Acid Type B Receptor Subunit 1 |
| ATF1 | Activating Transcription Factor 1 |
| WNT7A | Wnt Family Member 7A |
| HSPA2 | Heat Shock Protein Family A (Hsp70) Member 2 |
| CXCR6 | C-X-C Motif Chemokine Receptor 6 |
| TLR8 | Toll Like Receptor 8 |
| PRKCE | Protein Kinase C Epsilon |
| MIR328 | MicroRNA 328 |
| WNK2 | WNK Lysine Deficient Protein Kinase 2 |
| LTB4R | Leukotriene B4 Receptor |
| EIF5A | Eukaryotic Translation Initiation Factor 5A |
| LMO4 | LIM Domain Only 4 |
| BRS3 | Bombesin Receptor Subtype 3 |
| EFEMP1 | EGF Containing Fibulin Extracellular Matrix Protein 1 |
| MUC20 | Mucin 20, Cell Surface Associated |
| FZD7 | Frizzled Class Receptor 7 |
| WRAP53 | WD Repeat Containing Antisense To TP53 |
| SLC19A1 | Solute Carrier Family 19 Member 1 |
| SEMA3C | Semaphorin 3C |
| MIR135A2 | MicroRNA 135a-2 |
| CAPN2 | Calpain 2 |
| HSF1 | Heat Shock Transcription Factor 1 |
| MIR135B | MicroRNA 135b |
| FABP5 | Fatty Acid Binding Protein 5 |
| ANXA6 | Annexin A6 |
| BMP1 | Bone Morphogenetic Protein 1 |
| REN | Renin |
| LGALS9 | Galectin 9 |
| SLC4A4 | Solute Carrier Family 4 Member 4 |
| AZGP1 | Alpha-2-Glycoprotein 1, Zinc-Binding |
| SULT1A3 | Sulfotransferase Family 1A Member 3 |
| FEN1 | Flap Structure-Specific Endonuclease 1 |
| SLIT2 | Slit Guidance Ligand 2 |
| TYK2 | Tyrosine Kinase 2 |
| WNT3 | Wnt Family Member 3 |
| RASSF2 | Ras Association Domain Family Member 2 |
| ADAM10 | ADAM Metallopeptidase Domain 10 |
| MMP15 | Matrix Metallopeptidase 15 |
| CBX7 | Chromobox 7 |
| SKI | SKI Proto-Oncogene |
| MSX2 | Msh Homeobox 2 |
| MUCL3 | Mucin Like 3 |
| KLRK1 | Killer Cell Lectin Like Receptor K1 |
| SYCN | Syncollin |
| TACR1 | Tachykinin Receptor 1 |
| LINC01234 | Long Intergenic Non-Protein Coding RNA 1234 |
| VCAN | Versican |
| TFEB | Transcription Factor EB |
| FAM215A | Family With Sequence Similarity 215 Member A |
| E2F4 | E2F Transcription Factor 4 |
| POLB | DNA Polymerase Beta |
| SLC4A2 | Solute Carrier Family 4 Member 2 |
| PIWIL1 | Piwi Like RNA-Mediated Gene Silencing 1 |
| SREBF2 | Sterol Regulatory Element Binding Transcription Factor 2 |
| CFB | Complement Factor B |
| CHKB | Choline Kinase Beta |
| PTBP1 | Polypyrimidine Tract Binding Protein 1 |
| TRIM29 | Tripartite Motif Containing 29 |
| HYAL1 | Hyaluronidase 1 |
| MYOF | Myoferlin |
| SLC5A5 | Solute Carrier Family 5 Member 5 |
| PNMA1 | PNMA Family Member 1 |
| RHOG | Ras Homolog Family Member G |
| ICAM2 | Intercellular Adhesion Molecule 2 |
| UPK3A | Uroplakin 3A |
| EFNA1 | Ephrin A1 |
| PLAC1 | Placenta Enriched 1 |
| BCL3 | BCL3 Transcription Coactivator |
| MIR608 | MicroRNA 608 |
| PCDH10 | Protocadherin 10 |
| PRDX2 | Peroxiredoxin 2 |
| ACVR2A | Activin A Receptor Type 2A |
| EIF3A | Eukaryotic Translation Initiation Factor 3 Subunit A |
| EWSR1 | EWS RNA Binding Protein 1 |
| CUL1 | Cullin 1 |
| IFNB1 | Interferon Beta 1 |
| RAP1A | RAP1A, Member Of RAS Oncogene Family |
| ARG1 | Arginase 1 |
| FLNA | Filamin A |
| ABCA1 | ATP Binding Cassette Subfamily A Member 1 |
| SLC39A4 | Solute Carrier Family 39 Member 4 |
| SPTBN1 | Spectrin Beta, Non-Erythrocytic 1 |
| ARRB1 | Arrestin Beta 1 |
| PCNT | Pericentrin |
| DESI2 | Desumoylating Isopeptidase 2 |
| CLIC4 | Chloride Intracellular Channel 4 |
| SLC26A3 | Solute Carrier Family 26 Member 3 |
| MT3 | Metallothionein 3 |
| MIR194-1 | MicroRNA 194-1 |
| ACVR1C | Activin A Receptor Type 1C |
| TNFAIP3 | TNF Alpha Induced Protein 3 |
| GSK3A | Glycogen Synthase Kinase 3 Alpha |
| DUOX2 | Dual Oxidase 2 |
| CDK8 | Cyclin Dependent Kinase 8 |
| SALL4 | Spalt Like Transcription Factor 4 |
| ITGB2 | Integrin Subunit Beta 2 |
| MIR940 | MicroRNA 940 |
| DEFA1 | Defensin Alpha 1 |
| CEBPA | CCAAT Enhancer Binding Protein Alpha |
| COL14A1 | Collagen Type XIV Alpha 1 Chain |
| F11R | F11 Receptor |
| UPF1 | UPF1 RNA Helicase And ATPase |
| ITPR3 | Inositol 1,4,5-Trisphosphate Receptor Type 3 |
| ALX4 | ALX Homeobox 4 |
| NIN | Ninein |
| MIR194-2 | MicroRNA 194-2 |
| DPYSL3 | Dihydropyrimidinase Like 3 |
| SCGB3A1 | Secretoglobin Family 3A Member 1 |
| OSM | Oncostatin M |
| RPS6 | Ribosomal Protein S6 |
| CES2 | Carboxylesterase 2 |
| LMTK3 | Lemur Tyrosine Kinase 3 |
| SDF4 | Stromal Cell Derived Factor 4 |
| CCN5 | Cellular Communication Network Factor 5 |
| GUCA2B | Guanylate Cyclase Activator 2B |
| SLC2A3 | Solute Carrier Family 2 Member 3 |
| RTN4 | Reticulon 4 |
| NEK2 | NIMA Related Kinase 2 |
| ZFP36L2 | ZFP36 Ring Finger Protein Like 2 |
| CIB1 | Calcium And Integrin Binding 1 |
| ANXA3 | Annexin A3 |
| SPARCL1 | SPARC Like 1 |
| LTBP1 | Latent Transforming Growth Factor Beta Binding Protein 1 |
| DYRK1A | Dual Specificity Tyrosine Phosphorylation Regulated Kinase 1A |
| ARID1B | AT-Rich Interaction Domain 1B |
| ELOC | Elongin C |
| BHLHA15 | Basic Helix-Loop-Helix Family Member A15 |
| CDO1 | Cysteine Dioxygenase Type 1 |
| IL2RB | Interleukin 2 Receptor Subunit Beta |
| MAPKAPK2 | MAPK Activated Protein Kinase 2 |
| FOXP2 | Forkhead Box P2 |
| CAV2 | Caveolin 2 |
| GLRX | Glutaredoxin |
| PPARGC1B | PPARG Coactivator 1 Beta |
| ZBTB33 | Zinc Finger And BTB Domain Containing 33 |
| OCIAD1 | OCIA Domain Containing 1 |
| SFRP5 | Secreted Frizzled Related Protein 5 |
| CCL21 | C-C Motif Chemokine Ligand 21 |
| KIF20A | Kinesin Family Member 20A |
| PTH1R | Parathyroid Hormone 1 Receptor |
| PER1 | Period Circadian Regulator 1 |
| LMX1A | LIM Homeobox Transcription Factor 1 Alpha |
| TUBG1 | Tubulin Gamma 1 |
| TTF2 | Transcription Termination Factor 2 |
| SETDB1 | SET Domain Bifurcated Histone Lysine Methyltransferase 1 |
| RAD54B | RAD54 Homolog B |
| TPO | Thyroid Peroxidase |
| AKAP12 | A-Kinase Anchoring Protein 12 |
| PYCARD | PYD And CARD Domain Containing |
| UBE2I | Ubiquitin Conjugating Enzyme E2 I |
| IRAK4 | Interleukin 1 Receptor Associated Kinase 4 |
| RAB27A | RAB27A, Member RAS Oncogene Family |
| STX2 | Syntaxin 2 |
| ABCC5 | ATP Binding Cassette Subfamily C Member 5 |
| PROS1 | Protein S |
| KLF2 | Kruppel Like Factor 2 |
| ATF2 | Activating Transcription Factor 2 |
| MAP2K5 | Mitogen-Activated Protein Kinase Kinase 5 |
| CSNK2A2 | Casein Kinase 2 Alpha 2 |
| CA1 | Carbonic Anhydrase 1 |
| NOS1 | Nitric Oxide Synthase 1 |
| LPCAT1 | Lysophosphatidylcholine Acyltransferase 1 |
| PRMT5 | Protein Arginine Methyltransferase 5 |
| JMJD6 | Jumonji Domain Containing 6, Arginine Demethylase And Lysine Hydroxylase |
| MT2A | Metallothionein 2A |
| TRPV6 | Transient Receptor Potential Cation Channel Subfamily V Member 6 |
| CALM1 | Calmodulin 1 |
| GRK6 | G Protein-Coupled Receptor Kinase 6 |
| CTAG2 | Cancer/Testis Antigen 2 |
| DDT | D-Dopachrome Tautomerase |
| PDGFD | Platelet Derived Growth Factor D |
| MIR374A | MicroRNA 374a |
| APOA2 | Apolipoprotein A2 |
| HSPA1B | Heat Shock Protein Family A (Hsp70) Member 1B |
| DDX58 | DExD/H-Box Helicase 58 |
| KDM5B | Lysine Demethylase 5B |
| SLC26A9 | Solute Carrier Family 26 Member 9 |
| A1BG | Alpha-1-B Glycoprotein |
| MT-ND4 | Mitochondrially Encoded NADH:Ubiquinone Oxidoreductase Core Subunit 4 |
| NEDD4 | NEDD4 E3 Ubiquitin Protein Ligase |
| LALBA | Lactalbumin Alpha |
| NFATC1 | Nuclear Factor Of Activated T Cells 1 |
| CD79A | CD79a Molecule |
| PCBP2 | Poly(RC) Binding Protein 2 |
| SLC6A14 | Solute Carrier Family 6 Member 14 |
| IQSEC1 | IQ Motif And Sec7 Domain ArfGEF 1 |
| GMNN | Geminin DNA Replication Inhibitor |
| PAK2 | P21 (RAC1) Activated Kinase 2 |
| DEAF1 | DEAF1 Transcription Factor |
| RPA1 | Replication Protein A1 |
| PCA3 | Prostate Cancer Associated 3 |
| IL22RA1 | Interleukin 22 Receptor Subunit Alpha 1 |
| KPNA2 | Karyopherin Subunit Alpha 2 |
| MDM4 | MDM4 Regulator Of P53 |
| GATA5 | GATA Binding Protein 5 |
| TMIGD2 | Transmembrane And Immunoglobulin Domain Containing 2 |
| SOX17 | SRY-Box Transcription Factor 17 |
| RGN | Regucalcin |
| IRF2 | Interferon Regulatory Factor 2 |
| CYP11A1 | Cytochrome P450 Family 11 Subfamily A Member 1 |
| MCPH1 | Microcephalin 1 |
| ARHGAP35 | Rho GTPase Activating Protein 35 |
| B3GAT1 | Beta-1,3-Glucuronyltransferase 1 |
| MGLL | Monoglyceride Lipase |
| LFNG | LFNG O-Fucosylpeptide 3-Beta-N-Acetylglucosaminyltransferase |
| HMGB2 | High Mobility Group Box 2 |
| CXCL9 | C-X-C Motif Chemokine Ligand 9 |
| XRCC4 | X-Ray Repair Cross Complementing 4 |
| PHLDA1 | Pleckstrin Homology Like Domain Family A Member 1 |
| CD69 | CD69 Molecule |
| CD164 | CD164 Molecule |
| HIF1AN | Hypoxia Inducible Factor 1 Subunit Alpha Inhibitor |
| LAMA3 | Laminin Subunit Alpha 3 |
| KMT2D | Lysine Methyltransferase 2D |
| ELK3 | ETS Transcription Factor ELK3 |
| GNA12 | G Protein Subunit Alpha 12 |
| LINC00628 | Long Intergenic Non-Protein Coding RNA 628 |
| STEAP1 | STEAP Family Member 1 |
| PPP4C | Protein Phosphatase 4 Catalytic Subunit |
| ING4 | Inhibitor Of Growth Family Member 4 |
| MAGEC2 | MAGE Family Member C2 |
| EEF2 | Eukaryotic Translation Elongation Factor 2 |
| BBS9 | Bardet-Biedl Syndrome 9 |
| TAP2 | Transporter 2, ATP Binding Cassette Subfamily B Member |
| KRT10 | Keratin 10 |
| PRDX5 | Peroxiredoxin 5 |
| BACH1 | BTB Domain And CNC Homolog 1 |
| METTL3 | Methyltransferase Like 3 |
| PPP2CA | Protein Phosphatase 2 Catalytic Subunit Alpha |
| ARHGEF2 | Rho/Rac Guanine Nucleotide Exchange Factor 2 |
| TMPO | Thymopoietin |
| ENTPD1 | Ectonucleoside Triphosphate Diphosphohydrolase 1 |
| TNKS2 | Tankyrase 2 |
| RAB5A | RAB5A, Member RAS Oncogene Family |
| AGO2 | Argonaute RISC Catalytic Component 2 |
| TPD52 | Tumor Protein D52 |
| LRG1 | Leucine Rich Alpha-2-Glycoprotein 1 |
| CXCL2 | C-X-C Motif Chemokine Ligand 2 |
| EMD | Emerin |
| RASSF6 | Ras Association Domain Family Member 6 |
| MRGBP | MRG Domain Binding Protein |
| PPARGC1A | PPARG Coactivator 1 Alpha |
| EIF6 | Eukaryotic Translation Initiation Factor 6 |
| FRAT1 | FRAT Regulator Of WNT Signaling Pathway 1 |
| LAMP2 | Lysosomal Associated Membrane Protein 2 |
| PLCB3 | Phospholipase C Beta 3 |
| AFAP1L2 | Actin Filament Associated Protein 1 Like 2 |
| PEBP4 | Phosphatidylethanolamine Binding Protein 4 |
| MORC4 | MORC Family CW-Type Zinc Finger 4 |
| BCHE | Butyrylcholinesterase |
| PFKP | Phosphofructokinase, Platelet |
| MARCKS | Myristoylated Alanine Rich Protein Kinase C Substrate |
| SNW1 | SNW Domain Containing 1 |
| HOXA1 | Homeobox A1 |
| TYRP1 | Tyrosinase Related Protein 1 |
| FANCD2 | FA Complementation Group D2 |
| AOC1 | Amine Oxidase Copper Containing 1 |
| NTF4 | Neurotrophin 4 |
| CCDC88A | Coiled-Coil Domain Containing 88A |
| ACHE | Acetylcholinesterase (Cartwright Blood Group) |
| CDC42BPB | CDC42 Binding Protein Kinase Beta |
| FABP1 | Fatty Acid Binding Protein 1 |
| PLAC8 | Placenta Associated 8 |
| EDIL3 | EGF Like Repeats And Discoidin Domains 3 |
| EIF2A | Eukaryotic Translation Initiation Factor 2A |
| BMPR1B | Bone Morphogenetic Protein Receptor Type 1B |
| CARD16 | Caspase Recruitment Domain Family Member 16 |
| OS9 | OS9 Endoplasmic Reticulum Lectin |
| FERMT3 | Fermitin Family Member 3 |
| PTPRJ | Protein Tyrosine Phosphatase Receptor Type J |
| IL12B | Interleukin 12B |
| ISYNA1 | Inositol-3-Phosphate Synthase 1 |
| VAV2 | Vav Guanine Nucleotide Exchange Factor 2 |
| MAP1LC3A | Microtubule Associated Protein 1 Light Chain 3 Alpha |
| PSMB4 | Proteasome 20S Subunit Beta 4 |
| AHCY | Adenosylhomocysteinase |
| PLAG1 | PLAG1 Zinc Finger |
| DROSHA | Drosha Ribonuclease III |
| MIR874 | MicroRNA 874 |
| IRS4 | Insulin Receptor Substrate 4 |
| MIR138-2 | MicroRNA 138-2 |
| RXRB | Retinoid X Receptor Beta |
| FTO | FTO Alpha-Ketoglutarate Dependent Dioxygenase |
| USP8 | Ubiquitin Specific Peptidase 8 |
| BCL6 | BCL6 Transcription Repressor |
| AMFR | Autocrine Motility Factor Receptor |
| ORAI1 | ORAI Calcium Release-Activated Calcium Modulator 1 |
| HTRA3 | HtrA Serine Peptidase 3 |
| GRK3 | G Protein-Coupled Receptor Kinase 3 |
| CYP4F2 | Cytochrome P450 Family 4 Subfamily F Member 2 |
| MOAP1 | Modulator Of Apoptosis 1 |
| POU1F1 | POU Class 1 Homeobox 1 |
| TNFSF13 | TNF Superfamily Member 13 |
| CRKL | CRK Like Proto-Oncogene, Adaptor Protein |
| ALDH1B1 | Aldehyde Dehydrogenase 1 Family Member B1 |
| SERBP1 | SERPINE1 MRNA Binding Protein 1 |
| PPBP | Pro-Platelet Basic Protein |
| PEAK1 | Pseudopodium Enriched Atypical Kinase 1 |
| CHD7 | Chromodomain Helicase DNA Binding Protein 7 |
| KRT16 | Keratin 16 |
| ESRP1 | Epithelial Splicing Regulatory Protein 1 |
| UCHL5 | Ubiquitin C-Terminal Hydrolase L5 |
| GABRB3 | Gamma-Aminobutyric Acid Type A Receptor Subunit Beta3 |
| LTBP4 | Latent Transforming Growth Factor Beta Binding Protein 4 |
| CANT1 | Calcium Activated Nucleotidase 1 |
| MIR637 | MicroRNA 637 |
| CXCL13 | C-X-C Motif Chemokine Ligand 13 |
| NME2 | NME/NM23 Nucleoside Diphosphate Kinase 2 |
| MLXIPL | MLX Interacting Protein Like |
| CRYAB | Crystallin Alpha B |
| PLEK | Pleckstrin |
| SRPRB | SRP Receptor Subunit Beta |
| CYP2C19 | Cytochrome P450 Family 2 Subfamily C Member 19 |
| URI1 | URI1 Prefoldin Like Chaperone |
| SPOCK1 | SPARC (Osteonectin), Cwcv And Kazal Like Domains Proteoglycan 1 |
| TAC1 | Tachykinin Precursor 1 |
| NGRN | Neugrin, Neurite Outgrowth Associated |
| GCNT1 | Glucosaminyl (N-Acetyl) Transferase 1 |
| NRG2 | Neuregulin 2 |
| ATP5F1B | ATP Synthase F1 Subunit Beta |
| CKAP4 | Cytoskeleton Associated Protein 4 |
| SPAM1 | Sperm Adhesion Molecule 1 |
| LXN | Latexin |
| ABCC11 | ATP Binding Cassette Subfamily C Member 11 |
| ITGAL | Integrin Subunit Alpha L |
| LPAR3 | Lysophosphatidic Acid Receptor 3 |
| NPPB | Natriuretic Peptide B |
| DEPTOR | DEP Domain Containing MTOR Interacting Protein |
| GNA13 | G Protein Subunit Alpha 13 |
| CTNNA2 | Catenin Alpha 2 |
| LINC00963 | Long Intergenic Non-Protein Coding RNA 963 |
| ITGB5 | Integrin Subunit Beta 5 |
| RAD23B | RAD23 Homolog B, Nucleotide Excision Repair Protein |
| LHCGR | Luteinizing Hormone/Choriogonadotropin Receptor |
| MIR367 | MicroRNA 367 |
| RBBP8 | RB Binding Protein 8, Endonuclease |
| AGK | Acylglycerol Kinase |
| MYBL2 | MYB Proto-Oncogene Like 2 |
| FERMT2 | Fermitin Family Member 2 |
| BMX | BMX Non-Receptor Tyrosine Kinase |
| ITGA1 | Integrin Subunit Alpha 1 |
| FANCA | FA Complementation Group A |
| HSD3B2 | Hydroxy-Delta-5-Steroid Dehydrogenase, 3 Beta- And Steroid Delta-Isomerase 2 |
| NFKB2 | Nuclear Factor Kappa B Subunit 2 |
| CNN1 | Calponin 1 |
| PNKD | PNKD Metallo-Beta-Lactamase Domain Containing |
| MIR153-1 | MicroRNA 153-1 |
| MIR153-2 | MicroRNA 153-2 |
| CLDN6 | Claudin 6 |
| SFRP4 | Secreted Frizzled Related Protein 4 |
| LAMTOR5 | Late Endosomal/Lysosomal Adaptor, MAPK And MTOR Activator 5 |
| PKN1 | Protein Kinase N1 |
| ATP2C1 | ATPase Secretory Pathway Ca2+ Transporting 1 |
| GANAB | Glucosidase II Alpha Subunit |
| CNTLN | Centlein |
| DPEP1 | Dipeptidase 1 |
| PMEPA1 | Prostate Transmembrane Protein, Androgen Induced 1 |
| COL1A2 | Collagen Type I Alpha 2 Chain |
| CDH10 | Cadherin 10 |
| MT1E | Metallothionein 1E |
| SDC2 | Syndecan 2 |
| LOC110806263 | TERT 5' Regulatory Region |
| PER2 | Period Circadian Regulator 2 |
| IVL | Involucrin |
| BDH1 | 3-Hydroxybutyrate Dehydrogenase 1 |
| CCNT2 | Cyclin T2 |
| MYH9 | Myosin Heavy Chain 9 |
| TMSB10 | Thymosin Beta 10 |
| SRSF6 | Serine And Arginine Rich Splicing Factor 6 |
| GYS1 | Glycogen Synthase 1 |
| UGT2B17 | UDP Glucuronosyltransferase Family 2 Member B17 |
| CACYBP | Calcyclin Binding Protein |
| PPP1R13L | Protein Phosphatase 1 Regulatory Subunit 13 Like |
| RAC3 | Rac Family Small GTPase 3 |
| GOLPH3 | Golgi Phosphoprotein 3 |
| GPNMB | Glycoprotein Nmb |
| MIR17HG | MiR-17-92a-1 Cluster Host Gene |
| CXCL11 | C-X-C Motif Chemokine Ligand 11 |
| MGAT4A | Alpha-1,3-Mannosyl-Glycoprotein 4-Beta-N-Acetylglucosaminyltransferase A |
| SIRT6 | Sirtuin 6 |
| RALBP1 | RalA Binding Protein 1 |
| THRB | Thyroid Hormone Receptor Beta |
| HOXC8 | Homeobox C8 |
| TOPBP1 | DNA Topoisomerase II Binding Protein 1 |
| CRH | Corticotropin Releasing Hormone |
| USP7 | Ubiquitin Specific Peptidase 7 |
| ALKBH3 | AlkB Homolog 3, Alpha-Ketoglutaratedependent Dioxygenase |
| CLTC | Clathrin Heavy Chain |
| TRIP10 | Thyroid Hormone Receptor Interactor 10 |
| GFRA3 | GDNF Family Receptor Alpha 3 |
| PBK | PDZ Binding Kinase |
| UBD | Ubiquitin D |
| HIPK2 | Homeodomain Interacting Protein Kinase 2 |
| PIAS3 | Protein Inhibitor Of Activated STAT 3 |
| H4C1 | H4 Clustered Histone 1 |
| SOS2 | SOS Ras/Rho Guanine Nucleotide Exchange Factor 2 |
| ARHGDIA | Rho GDP Dissociation Inhibitor Alpha |
| TNFAIP8 | TNF Alpha Induced Protein 8 |
| DTNBP1 | Dystrobrevin Binding Protein 1 |
| EDN3 | Endothelin 3 |
| ACKR1 | Atypical Chemokine Receptor 1 (Duffy Blood Group) |
| FOXJ1 | Forkhead Box J1 |
| KCNN4 | Potassium Calcium-Activated Channel Subfamily N Member 4 |
| PRKG1 | Protein Kinase CGMP-Dependent 1 |
| MATK | Megakaryocyte-Associated Tyrosine Kinase |
| EAF2 | ELL Associated Factor 2 |
| PTPRU | Protein Tyrosine Phosphatase Receptor Type U |
| EMB | Embigin |
| PSMD9 | Proteasome 26S Subunit, Non-ATPase 9 |
| TPT1 | Tumor Protein, Translationally-Controlled 1 |
| KPNB1 | Karyopherin Subunit Beta 1 |
| MIR370 | MicroRNA 370 |
| MAP4K1 | Mitogen-Activated Protein Kinase Kinase Kinase Kinase 1 |
| NT5E | 5'-Nucleotidase Ecto |
| DCTD | DCMP Deaminase |
| EGLN2 | Egl-9 Family Hypoxia Inducible Factor 2 |
| RING1 | Ring Finger Protein 1 |
| EYA4 | EYA Transcriptional Coactivator And Phosphatase 4 |
| COMMD7 | COMM Domain Containing 7 |
| OXT | Oxytocin/Neurophysin I Prepropeptide |
| SMAD1 | SMAD Family Member 1 |
| LRAT | Lecithin Retinol Acyltransferase |
| BTG1 | BTG Anti-Proliferation Factor 1 |
| LTBP2 | Latent Transforming Growth Factor Beta Binding Protein 2 |
| PDIA6 | Protein Disulfide Isomerase Family A Member 6 |
| RPN2 | Ribophorin II |
| CSDE1 | Cold Shock Domain Containing E1 |
| VSIR | V-Set Immunoregulatory Receptor |
| TRIM33 | Tripartite Motif Containing 33 |
| KRT81 | Keratin 81 |
| HBP1 | HMG-Box Transcription Factor 1 |
| RUVBL1 | RuvB Like AAA ATPase 1 |
| FOXQ1 | Forkhead Box Q1 |
| GP5 | Glycoprotein V Platelet |
| CD1A | CD1a Molecule |
| PTGER1 | Prostaglandin E Receptor 1 |
| NXPH1 | Neurexophilin 1 |
| NXPH2 | Neurexophilin 2 |
| LBX1 | Ladybird Homeobox 1 |
| ARF6 | ADP Ribosylation Factor 6 |
| NKX2-8 | NK2 Homeobox 8 |
| CCNH | Cyclin H |
| CYP2C9 | Cytochrome P450 Family 2 Subfamily C Member 9 |
| NRTN | Neurturin |
| KLF8 | Kruppel Like Factor 8 |
| RHOH | Ras Homolog Family Member H |
| RBBP5 | RB Binding Protein 5, Histone Lysine Methyltransferase Complex Subunit |
| TRADD | TNFRSF1A Associated Via Death Domain |
| COL3A1 | Collagen Type III Alpha 1 Chain |
| MAPT | Microtubule Associated Protein Tau |
| UBL4A | Ubiquitin Like 4A |
| ADRB1 | Adrenoceptor Beta 1 |
| CCR2 | C-C Motif Chemokine Receptor 2 |
| ESYT2 | Extended Synaptotagmin 2 |
| ECD | Ecdysoneless Cell Cycle Regulator |
| EXOC1 | Exocyst Complex Component 1 |
| ALDH1A2 | Aldehyde Dehydrogenase 1 Family Member A2 |
| CTBP1 | C-Terminal Binding Protein 1 |
| PGAM1 | Phosphoglycerate Mutase 1 |
| MSI2 | Musashi RNA Binding Protein 2 |
| SPEN | Spen Family Transcriptional Repressor |
| YWHAE | Tyrosine 3-Monooxygenase/Tryptophan 5-Monooxygenase Activation Protein Epsilon |
| CD81 | CD81 Molecule |
| NOP2 | NOP2 Nucleolar Protein |
| MIR539 | MicroRNA 539 |
| PTPRF | Protein Tyrosine Phosphatase Receptor Type F |
| HDAC5 | Histone Deacetylase 5 |
| TOX3 | TOX High Mobility Group Box Family Member 3 |
| BMP3 | Bone Morphogenetic Protein 3 |
| MIR323A | MicroRNA 323a |
| AP2B1 | Adaptor Related Protein Complex 2 Subunit Beta 1 |
| APLP2 | Amyloid Beta Precursor Like Protein 2 |
| ZFX | Zinc Finger Protein X-Linked |
| NUCKS1 | Nuclear Casein Kinase And Cyclin Dependent Kinase Substrate 1 |
| CYBA | Cytochrome B-245 Alpha Chain |
| PPP2R2A | Protein Phosphatase 2 Regulatory Subunit Balpha |
| RBBP4 | RB Binding Protein 4, Chromatin Remodeling Factor |
| CIITA | Class II Major Histocompatibility Complex Transactivator |
| CPA4 | Carboxypeptidase A4 |
| ONECUT3 | One Cut Homeobox 3 |
| DIXDC1 | DIX Domain Containing 1 |
| GLIPR1 | GLI Pathogenesis Related 1 |
| IRAK1 | Interleukin 1 Receptor Associated Kinase 1 |
| AICDA | Activation Induced Cytidine Deaminase |
| TFPI | Tissue Factor Pathway Inhibitor |
| ESRRG | Estrogen Related Receptor Gamma |
| FUT6 | Fucosyltransferase 6 |
| AQP4 | Aquaporin 4 |
| MAZ | MYC Associated Zinc Finger Protein |
| PTP4A3 | Protein Tyrosine Phosphatase 4A3 |
| GABRP | Gamma-Aminobutyric Acid Type A Receptor Subunit Pi |
| EIF4A1 | Eukaryotic Translation Initiation Factor 4A1 |
| DNM1L | Dynamin 1 Like |
| KAT5 | Lysine Acetyltransferase 5 |
| NET1 | Neuroepithelial Cell Transforming 1 |
| SHC2 | SHC Adaptor Protein 2 |
| LHX2 | LIM Homeobox 2 |
| SNORA23 | Small Nucleolar RNA, H/ACA Box 23 |
| ITIH5 | Inter-Alpha-Trypsin Inhibitor Heavy Chain 5 |
| KLK13 | Kallikrein Related Peptidase 13 |
| VAV1 | Vav Guanine Nucleotide Exchange Factor 1 |
| CAST | Calpastatin |
| STK24 | Serine/Threonine Kinase 24 |
| F10 | Coagulation Factor X |
| PRRC2A | Proline Rich Coiled-Coil 2A |
| BLVRB | Biliverdin Reductase B |
| STIP1 | Stress Induced Phosphoprotein 1 |
| PRKD3 | Protein Kinase D3 |
| OGT | O-Linked N-Acetylglucosamine (GlcNAc) Transferase |
| COL1A1 | Collagen Type I Alpha 1 Chain |
| IDO2 | Indoleamine 2,3-Dioxygenase 2 |
| ERCC3 | ERCC Excision Repair 3, TFIIH Core Complex Helicase Subunit |
| MXD1 | MAX Dimerization Protein 1 |
| MIR99B | MicroRNA 99b |
| PPM1A | Protein Phosphatase, Mg2+/Mn2+ Dependent 1A |
| GFI1 | Growth Factor Independent 1 Transcriptional Repressor |
| TBX4 | T-Box Transcription Factor 4 |
| SLC24A2 | Solute Carrier Family 24 Member 2 |
| CST7 | Cystatin F |
| ZNF451 | Zinc Finger Protein 451 |
| CCDC68 | Coiled-Coil Domain Containing 68 |
| SERP1 | Stress Associated Endoplasmic Reticulum Protein 1 |
| MIR664A | MicroRNA 664a |
| MIR545 | MicroRNA 545 |
| LINC00226 | Long Intergenic Non-Protein Coding RNA 226 |
| MIR4295 | MicroRNA 4295 |
| MIR1294 | MicroRNA 1294 |
| MIR1253 | MicroRNA 1253 |
| F11-AS1 | F11 Antisense RNA 1 |
| ST2 | Suppression Of Tumorigenicity 2 |
| SMARCC1 | SWI/SNF Related, Matrix Associated, Actin Dependent Regulator Of Chromatin Subfamily C Member 1 |
| LDHB | Lactate Dehydrogenase B |
| SDC3 | Syndecan 3 |
| DCD | Dermcidin |
| HSD3B1 | Hydroxy-Delta-5-Steroid Dehydrogenase, 3 Beta- And Steroid Delta-Isomerase 1 |
| FOXL1 | Forkhead Box L1 |
| GFPT1 | Glutamine--Fructose-6-Phosphate Transaminase 1 |
| LBP | Lipopolysaccharide Binding Protein |
| MEST | Mesoderm Specific Transcript |
| IL6ST | Interleukin 6 Signal Transducer |
| SRP72 | Signal Recognition Particle 72 |
| RPL29 | Ribosomal Protein L29 |
| PTX3 | Pentraxin 3 |
| SIK1 | Salt Inducible Kinase 1 |
| MIRLET7A2 | MicroRNA Let-7a-2 |
| MAX | MYC Associated Factor X |
| KIF15 | Kinesin Family Member 15 |
| CCT2 | Chaperonin Containing TCP1 Subunit 2 |
| HPR | Haptoglobin-Related Protein |
| ECM1 | Extracellular Matrix Protein 1 |
| ALPG | Alkaline Phosphatase, Germ Cell |
| JPH4 | Junctophilin 4 |
| PRRX1 | Paired Related Homeobox 1 |
| KRT15 | Keratin 15 |
| PBRM1 | Polybromo 1 |
| MTNR1A | Melatonin Receptor 1A |
| MVD | Mevalonate Diphosphate Decarboxylase |
| RNLS | Renalase, FAD Dependent Amine Oxidase |
| MADD | MAP Kinase Activating Death Domain |
| STIL | STIL Centriolar Assembly Protein |
| FUBP1 | Far Upstream Element Binding Protein 1 |
| MECOM | MDS1 And EVI1 Complex Locus |
| TRPS1 | Transcriptional Repressor GATA Binding 1 |
| CRYAA | Crystallin Alpha A |
| PLD2 | Phospholipase D2 |
| ACTN1 | Actinin Alpha 1 |
| PLA2G10 | Phospholipase A2 Group X |
| SELENOP | Selenoprotein P |
| MGAT5 | Alpha-1,6-Mannosylglycoprotein 6-Beta-N-Acetylglucosaminyltransferase |
| PAM | Peptidylglycine Alpha-Amidating Monooxygenase |
| PFKM | Phosphofructokinase, Muscle |
| SIN3A | SIN3 Transcription Regulator Family Member A |
| YWHAZ | Tyrosine 3-Monooxygenase/Tryptophan 5-Monooxygenase Activation Protein Zeta |
| PTGR2 | Prostaglandin Reductase 2 |
| DVL2 | Dishevelled Segment Polarity Protein 2 |
| PPM1H | Protein Phosphatase, Mg2+/Mn2+ Dependent 1H |
| SOX11 | SRY-Box Transcription Factor 11 |
| MPL | MPL Proto-Oncogene, Thrombopoietin Receptor |
| GALNT6 | Polypeptide N-Acetylgalactosaminyltransferase 6 |
| CNR2 | Cannabinoid Receptor 2 |
| KLK14 | Kallikrein Related Peptidase 14 |
| SLC31A1 | Solute Carrier Family 31 Member 1 |
| COMP | Cartilage Oligomeric Matrix Protein |
| PAX5 | Paired Box 5 |
| MARVELD2 | MARVEL Domain Containing 2 |
| SYT7 | Synaptotagmin 7 |
| KCNA5 | Potassium Voltage-Gated Channel Subfamily A Member 5 |
| KHDRBS1 | KH RNA Binding Domain Containing, Signal Transduction Associated 1 |
| TUBA4A | Tubulin Alpha 4a |
| CACNA1C | Calcium Voltage-Gated Channel Subunit Alpha1 C |
| RHOBTB2 | Rho Related BTB Domain Containing 2 |
| ASPM | Abnormal Spindle Microtubule Assembly |
| RTRAF | RNA Transcription, Translation And Transport Factor |
| SPAG1 | Sperm Associated Antigen 1 |
| TMSB4X | Thymosin Beta 4 X-Linked |
| ANLN | Anillin Actin Binding Protein |
| ARL4C | ADP Ribosylation Factor Like GTPase 4C |
| DEFA1B | Defensin Alpha 1B |
| RGS22 | Regulator Of G Protein Signaling 22 |
| SRRT | Serrate, RNA Effector Molecule |
| PLPP5 | Phospholipid Phosphatase 5 |
| ESRRB | Estrogen Related Receptor Beta |
| EGFL7 | EGF Like Domain Multiple 7 |
| GNAQ | G Protein Subunit Alpha Q |
| CXCL3 | C-X-C Motif Chemokine Ligand 3 |
| DMKN | Dermokine |
| DUSP4 | Dual Specificity Phosphatase 4 |
| DAG1 | Dystroglycan 1 |
| IL23A | Interleukin 23 Subunit Alpha |
| GAS1 | Growth Arrest Specific 1 |
| DDX3X | DEAD-Box Helicase 3 X-Linked |
| PSIP1 | PC4 And SFRS1 Interacting Protein 1 |
| CCAR1 | Cell Division Cycle And Apoptosis Regulator 1 |
| FES | FES Proto-Oncogene, Tyrosine Kinase |
| CLCA2 | Chloride Channel Accessory 2 |
| PI16 | Peptidase Inhibitor 16 |
| TPD52L2 | TPD52 Like 2 |
| LSP1 | Lymphocyte Specific Protein 1 |
| DMTN | Dematin Actin Binding Protein |
| CSTB | Cystatin B |
| EEF2K | Eukaryotic Elongation Factor 2 Kinase |
| TOLLIP | Toll Interacting Protein |
| USF1 | Upstream Transcription Factor 1 |
| HBA1 | Hemoglobin Subunit Alpha 1 |
| BHLHE41 | Basic Helix-Loop-Helix Family Member E41 |
| CARM1 | Coactivator Associated Arginine Methyltransferase 1 |
| CXCR5 | C-X-C Motif Chemokine Receptor 5 |
| SPHK2 | Sphingosine Kinase 2 |
| SPN | Sialophorin |
| ARID4B | AT-Rich Interaction Domain 4B |
| ALOX15 | Arachidonate 15-Lipoxygenase |
| PRPF19 | Pre-MRNA Processing Factor 19 |
| C16orf74 | Chromosome 16 Open Reading Frame 74 |
| HOXA9 | Homeobox A9 |
| TBX3 | T-Box Transcription Factor 3 |
| PRAG1 | PEAK1 Related, Kinase-Activating Pseudokinase 1 |
| MAP2K3 | Mitogen-Activated Protein Kinase Kinase 3 |
| GLO1 | Glyoxalase I |
| TAT | Tyrosine Aminotransferase |
| KDM6A | Lysine Demethylase 6A |
| CREB3L4 | CAMP Responsive Element Binding Protein 3 Like 4 |
| UPP1 | Uridine Phosphorylase 1 |
| MIR504 | MicroRNA 504 |
| PLXNA3 | Plexin A3 |
| FAAH | Fatty Acid Amide Hydrolase |
| STK33 | Serine/Threonine Kinase 33 |
| RAB23 | RAB23, Member RAS Oncogene Family |
| HNRNPF | Heterogeneous Nuclear Ribonucleoprotein F |
| RGS6 | Regulator Of G Protein Signaling 6 |
| SOAT1 | Sterol O-Acyltransferase 1 |
| PLA2G2D | Phospholipase A2 Group IID |
| KRBOX4 | KRAB Box Domain Containing 4 |
| TRIM37 | Tripartite Motif Containing 37 |
| PIK3R4 | Phosphoinositide-3-Kinase Regulatory Subunit 4 |
| FGF6 | Fibroblast Growth Factor 6 |
| SPTAN1 | Spectrin Alpha, Non-Erythrocytic 1 |
| RBBP6 | RB Binding Protein 6, Ubiquitin Ligase |
| HGFAC | HGF Activator |
| CGA | Glycoprotein Hormones, Alpha Polypeptide |
| CKS1B | CDC28 Protein Kinase Regulatory Subunit 1B |
| GLUL | Glutamate-Ammonia Ligase |
| NID1 | Nidogen 1 |
| ADAMTS18 | ADAM Metallopeptidase With Thrombospondin Type 1 Motif 18 |
| C1QBP | Complement C1q Binding Protein |
| TUBB2A | Tubulin Beta 2A Class IIa |
| OCLN | Occludin |
| LLGL1 | LLGL Scribble Cell Polarity Complex Component 1 |
| RASD1 | Ras Related Dexamethasone Induced 1 |
| KDM3A | Lysine Demethylase 3A |
| PTS | 6-Pyruvoyltetrahydropterin Synthase |
| TBL1X | Transducin Beta Like 1 X-Linked |
| STARD3 | StAR Related Lipid Transfer Domain Containing 3 |
| PDE3B | Phosphodiesterase 3B |
| CIZ1 | CDKN1A Interacting Zinc Finger Protein 1 |
| LOC106721785 | BRCA2 Promoter/Silencer Region |
| PDLIM5 | PDZ And LIM Domain 5 |
| HTR1B | 5-Hydroxytryptamine Receptor 1B |
| AGRN | Agrin |
| NBL1 | NBL1, DAN Family BMP Antagonist |
| NMT1 | N-Myristoyltransferase 1 |
| STX3 | Syntaxin 3 |
| TGFB1I1 | Transforming Growth Factor Beta 1 Induced Transcript 1 |
| E2F5 | E2F Transcription Factor 5 |
| CST6 | Cystatin E/M |
| KLK15 | Kallikrein Related Peptidase 15 |
| CSF3R | Colony Stimulating Factor 3 Receptor |
| HOXD13 | Homeobox D13 |
| TDGF1P3 | Teratocarcinoma-Derived Growth Factor 1 Pseudogene 3 |
| GPC6 | Glypican 6 |
| BMP10 | Bone Morphogenetic Protein 10 |
| STOML2 | Stomatin Like 2 |
| CREB3L1 | CAMP Responsive Element Binding Protein 3 Like 1 |
| SUZ12P1 | SUZ12 Pseudogene 1 |
| AQP8 | Aquaporin 8 |
| GPRC6A | G Protein-Coupled Receptor Class C Group 6 Member A |
| COL17A1 | Collagen Type XVII Alpha 1 Chain |
| RAD23A | RAD23 Homolog A, Nucleotide Excision Repair Protein |
| HOXA5 | Homeobox A5 |
| ACVR2B | Activin A Receptor Type 2B |
| GPR68 | G Protein-Coupled Receptor 68 |
| TNFRSF14 | TNF Receptor Superfamily Member 14 |
| PTMA | Prothymosin Alpha |
| SIRPA | Signal Regulatory Protein Alpha |
| WDR5 | WD Repeat Domain 5 |
| MAML3 | Mastermind Like Transcriptional Coactivator 3 |
| UPK2 | Uroplakin 2 |
| RAP1GAP | RAP1 GTPase Activating Protein |
| CEACAM4 | CEA Cell Adhesion Molecule 4 |
| IFITM3 | Interferon Induced Transmembrane Protein 3 |
| RPLP2 | Ribosomal Protein Lateral Stalk Subunit P2 |
| BRD2 | Bromodomain Containing 2 |
| CCL1 | C-C Motif Chemokine Ligand 1 |
| PPA1 | Inorganic Pyrophosphatase 1 |
| CSPG4 | Chondroitin Sulfate Proteoglycan 4 |
| ACTR2 | Actin Related Protein 2 |
| TST | Thiosulfate Sulfurtransferase |
| REL | REL Proto-Oncogene, NF-KB Subunit |
| PDZD2 | PDZ Domain Containing 2 |
| DLG1 | Discs Large MAGUK Scaffold Protein 1 |
| NR2C2 | Nuclear Receptor Subfamily 2 Group C Member 2 |
| SIRT7 | Sirtuin 7 |
| PARPBP | PARP1 Binding Protein |
| NMU | Neuromedin U |
| NR1I3 | Nuclear Receptor Subfamily 1 Group I Member 3 |
| VASH1 | Vasohibin 1 |
| ANXA13 | Annexin A13 |
| RPS6KA3 | Ribosomal Protein S6 Kinase A3 |
| CGB5 | Chorionic Gonadotropin Subunit Beta 5 |
| CLN3 | CLN3 Lysosomal/Endosomal Transmembrane Protein, Battenin |
| FABP12 | Fatty Acid Binding Protein 12 |
| SEZ6L2 | Seizure Related 6 Homolog Like 2 |
| INTS6 | Integrator Complex Subunit 6 |
| USF2 | Upstream Transcription Factor 2, C-Fos Interacting |
| APLN | Apelin |
| MIR9-2 | MicroRNA 9-2 |
| SEMA4D | Semaphorin 4D |
| MIR491 | MicroRNA 491 |
| MIR301B | MicroRNA 301b |
| TNFSF12 | TNF Superfamily Member 12 |
| FBN2 | Fibrillin 2 |
| GRK2 | G Protein-Coupled Receptor Kinase 2 |
| SCYL1 | SCY1 Like Pseudokinase 1 |
| GSTA1 | Glutathione S-Transferase Alpha 1 |
| MT1F | Metallothionein 1F |
| MED1 | Mediator Complex Subunit 1 |
| PPIG | Peptidylprolyl Isomerase G |
| TBC1D3 | TBC1 Domain Family Member 3 |
| KCNK5 | Potassium Two Pore Domain Channel Subfamily K Member 5 |
| MIR136 | MicroRNA 136 |
| RAB11A | RAB11A, Member RAS Oncogene Family |
| FAT1 | FAT Atypical Cadherin 1 |
| LOC110386951 | CYP19A1 Promoter II/1.3 |
| PTP4A2 | Protein Tyrosine Phosphatase 4A2 |
| RAD21 | RAD21 Cohesin Complex Component |
| SENP3 | SUMO Specific Peptidase 3 |
| SEPSECS | Sep (O-Phosphoserine) TRNA:Sec (Selenocysteine) TRNA Synthase |
| CERK | Ceramide Kinase |
| SLC40A1 | Solute Carrier Family 40 Member 1 |
| LTB4R2 | Leukotriene B4 Receptor 2 |
| TRAF4 | TNF Receptor Associated Factor 4 |
| CAP1 | Cyclase Associated Actin Cytoskeleton Regulatory Protein 1 |
| DSG1 | Desmoglein 1 |
| USP16 | Ubiquitin Specific Peptidase 16 |
| BANP | BTG3 Associated Nuclear Protein |
| UBB | Ubiquitin B |
| SRPK1 | SRSF Protein Kinase 1 |
| H3C1 | H3 Clustered Histone 1 |
| RPA2 | Replication Protein A2 |
| SIN3B | SIN3 Transcription Regulator Family Member B |
| AURKC | Aurora Kinase C |
| EFS | Embryonal Fyn-Associated Substrate |
| PRPF8 | Pre-MRNA Processing Factor 8 |
| POU5F1B | POU Class 5 Homeobox 1B |
| ANK1 | Ankyrin 1 |
| ETV7 | ETS Variant Transcription Factor 7 |
| H2BC21 | H2B Clustered Histone 21 |
| RNF14 | Ring Finger Protein 14 |
| CTBP1-AS | CTBP1 Antisense RNA |
| ENSG00000261777 |  |
| CAPZB | Capping Actin Protein Of Muscle Z-Line Subunit Beta |
| ADGRE2 | Adhesion G Protein-Coupled Receptor E2 |
| WASF3 | WASP Family Member 3 |
| DKK4 | Dickkopf WNT Signaling Pathway Inhibitor 4 |
| PON2 | Paraoxonase 2 |
| TLE1 | TLE Family Member 1, Transcriptional Corepressor |
| INSIG2 | Insulin Induced Gene 2 |
| SCAP | SREBF Chaperone |
| CXCL14 | C-X-C Motif Chemokine Ligand 14 |
| PRDX6 | Peroxiredoxin 6 |
| EXTL3 | Exostosin Like Glycosyltransferase 3 |
| THAP9-AS1 | THAP9 Antisense RNA 1 |
| SMURF2 | SMAD Specific E3 Ubiquitin Protein Ligase 2 |
| GSTO1 | Glutathione S-Transferase Omega 1 |
| IGSF8 | Immunoglobulin Superfamily Member 8 |
| FHL2 | Four And A Half LIM Domains 2 |
| CCT3 | Chaperonin Containing TCP1 Subunit 3 |
| RBBP9 | RB Binding Protein 9, Serine Hydrolase |
| ABCC9 | ATP Binding Cassette Subfamily C Member 9 |
| CUEDC2 | CUE Domain Containing 2 |
| ME2 | Malic Enzyme 2 |
| CNDP2 | Carnosine Dipeptidase 2 |
| CHST15 | Carbohydrate Sulfotransferase 15 |
| ICMT | Isoprenylcysteine Carboxyl Methyltransferase |
| CYB5A | Cytochrome B5 Type A |
| C1GALT1C1 | C1GALT1 Specific Chaperone 1 |
| GAB1 | GRB2 Associated Binding Protein 1 |
| YY1 | YY1 Transcription Factor |
| PCAT14 | Prostate Cancer Associated Transcript 14 |
| EHF | ETS Homologous Factor |
| MIR129-2 | MicroRNA 129-2 |
| CD200 | CD200 Molecule |
| ST8SIA1 | ST8 Alpha-N-Acetyl-Neuraminide Alpha-2,8-Sialyltransferase 1 |
| CD47 | CD47 Molecule |
| PMP22 | Peripheral Myelin Protein 22 |
| CD163 | CD163 Molecule |
| VASP | Vasodilator Stimulated Phosphoprotein |
| ICA1 | Islet Cell Autoantigen 1 |
| ST13 | ST13 Hsp70 Interacting Protein |
| STOM | Stomatin |
| RNPEP | Arginyl Aminopeptidase |
| IGFBP6 | Insulin Like Growth Factor Binding Protein 6 |
| TMEM26 | Transmembrane Protein 26 |
| TUBA1A | Tubulin Alpha 1a |
| M6PR | Mannose-6-Phosphate Receptor, Cation Dependent |
| DNTT | DNA Nucleotidylexotransferase |
| EPRS1 | Glutamyl-Prolyl-TRNA Synthetase 1 |
| MIR302A | MicroRNA 302a |
| KSR2 | Kinase Suppressor Of Ras 2 |
| WWC1 | WW And C2 Domain Containing 1 |
| CFI | Complement Factor I |
| EIF3F | Eukaryotic Translation Initiation Factor 3 Subunit F |
| NEDD8 | NEDD8 Ubiquitin Like Modifier |
| TMOD4 | Tropomodulin 4 |
| DNAJA1 | DnaJ Heat Shock Protein Family (Hsp40) Member A1 |
| AP2A1 | Adaptor Related Protein Complex 2 Subunit Alpha 1 |
| DSC3 | Desmocollin 3 |
| GPR161 | G Protein-Coupled Receptor 161 |
| CXCL17 | C-X-C Motif Chemokine Ligand 17 |
| MT1X | Metallothionein 1X |
| MGP | Matrix Gla Protein |
| SLC15A1 | Solute Carrier Family 15 Member 1 |
| CEP170 | Centrosomal Protein 170 |
| WNT7B | Wnt Family Member 7B |
| EIF2S2 | Eukaryotic Translation Initiation Factor 2 Subunit Beta |
| PRPSAP2 | Phosphoribosyl Pyrophosphate Synthetase Associated Protein 2 |
| KDM6B | Lysine Demethylase 6B |
| NAE1 | NEDD8 Activating Enzyme E1 Subunit 1 |
| SCGB2A1 | Secretoglobin Family 2A Member 1 |
| CRHR1 | Corticotropin Releasing Hormone Receptor 1 |
| ARG2 | Arginase 2 |
| NECTIN4 | Nectin Cell Adhesion Molecule 4 |
| SKP1 | S-Phase Kinase Associated Protein 1 |
| FERMT1 | Fermitin Family Member 1 |
| ARHGEF4 | Rho Guanine Nucleotide Exchange Factor 4 |
| PRKACG | Protein Kinase CAMP-Activated Catalytic Subunit Gamma |
| NUDT1 | Nudix Hydrolase 1 |
| AMPH | Amphiphysin |
| TARBP2 | TARBP2 Subunit Of RISC Loading Complex |
| ANXA8L1 | Annexin A8 Like 1 |
| CGB7 | Chorionic Gonadotropin Subunit Beta 7 |
| IRF3 | Interferon Regulatory Factor 3 |
| SLC22A3 | Solute Carrier Family 22 Member 3 |
| MAP4K5 | Mitogen-Activated Protein Kinase Kinase Kinase Kinase 5 |
| CPEB4 | Cytoplasmic Polyadenylation Element Binding Protein 4 |
| FOLR2 | Folate Receptor Beta |
| SLCO2B1 | Solute Carrier Organic Anion Transporter Family Member 2B1 |
| ADARB2-AS1 | ADARB2 Antisense RNA 1 |
| NR0B1 | Nuclear Receptor Subfamily 0 Group B Member 1 |
| ATP6V1E1 | ATPase H+ Transporting V1 Subunit E1 |
| FNTA | Farnesyltransferase, CAAX Box, Alpha |
| CTSC | Cathepsin C |
| CSMD1 | CUB And Sushi Multiple Domains 1 |
| SMG1 | SMG1 Nonsense Mediated MRNA Decay Associated PI3K Related Kinase |
| SENP1 | SUMO Specific Peptidase 1 |
| PGRMC1 | Progesterone Receptor Membrane Component 1 |
| PPIB | Peptidylprolyl Isomerase B |
| MIR603 | MicroRNA 603 |
| PLXNC1 | Plexin C1 |
| DPP6 | Dipeptidyl Peptidase Like 6 |
| GLS | Glutaminase |
| MAPK12 | Mitogen-Activated Protein Kinase 12 |
| TWIST2 | Twist Family BHLH Transcription Factor 2 |
| CD101 | CD101 Molecule |
| SUMO1 | Small Ubiquitin Like Modifier 1 |
| PTGIS | Prostaglandin I2 Synthase |
| RIOK3 | RIO Kinase 3 |
| MT1A | Metallothionein 1A |
| RGL2 | Ral Guanine Nucleotide Dissociation Stimulator Like 2 |
| CTSS | Cathepsin S |
| HEY2 | Hes Related Family BHLH Transcription Factor With YRPW Motif 2 |
| SOCS6 | Suppressor Of Cytokine Signaling 6 |
| HCLS1 | Hematopoietic Cell-Specific Lyn Substrate 1 |
| SF3B4 | Splicing Factor 3b Subunit 4 |
| SEM1 | SEM1 26S Proteasome Complex Subunit |
| TPM4 | Tropomyosin 4 |
| BLZF1 | Basic Leucine Zipper Nuclear Factor 1 |
| GGH | Gamma-Glutamyl Hydrolase |
| SLC4A1 | Solute Carrier Family 4 Member 1 (Diego Blood Group) |
| EHMT1 | Euchromatic Histone Lysine Methyltransferase 1 |
| LTBR | Lymphotoxin Beta Receptor |
| NISCH | Nischarin |
| LINC01197 | Long Intergenic Non-Protein Coding RNA 1197 |
| CD28 | CD28 Molecule |
| PROKR1 | Prokineticin Receptor 1 |
| KIF3B | Kinesin Family Member 3B |
| SH3BP4 | SH3 Domain Binding Protein 4 |
| SAFB | Scaffold Attachment Factor B |
| LOC645468 | Insulin Like Growth Factor 2 MRNA Binding Protein 3 Pseudogene |
| SLC25A5 | Solute Carrier Family 25 Member 5 |
| MYO1D | Myosin ID |
| PCLAF | PCNA Clamp Associated Factor |
| ORM1 | Orosomucoid 1 |
| COL10A1 | Collagen Type X Alpha 1 Chain |
| BCAR3 | BCAR3 Adaptor Protein, NSP Family Member |
| MSL1 | MSL Complex Subunit 1 |
| PRKCQ | Protein Kinase C Theta |
| LMO2 | LIM Domain Only 2 |
| MIR363 | MicroRNA 363 |
| BUB3 | BUB3 Mitotic Checkpoint Protein |
| TES | Testin LIM Domain Protein |
| KRT9 | Keratin 9 |
| HTRA1 | HtrA Serine Peptidase 1 |
| TP53INP1 | Tumor Protein P53 Inducible Nuclear Protein 1 |
| NAF1 | Nuclear Assembly Factor 1 Ribonucleoprotein |
| EPC1 | Enhancer Of Polycomb Homolog 1 |
| CD58 | CD58 Molecule |
| TTLL4 | Tubulin Tyrosine Ligase Like 4 |
| REST | RE1 Silencing Transcription Factor |
| MZB1 | Marginal Zone B And B1 Cell Specific Protein |
| TGM1 | Transglutaminase 1 |
| MIR1285-2 | MicroRNA 1285-2 |
| MIR1285-1 | MicroRNA 1285-1 |
| KCNK2 | Potassium Two Pore Domain Channel Subfamily K Member 2 |
| ERAP1 | Endoplasmic Reticulum Aminopeptidase 1 |
| UGP2 | UDP-Glucose Pyrophosphorylase 2 |
| FSTL3 | Follistatin Like 3 |
| CELSR3 | Cadherin EGF LAG Seven-Pass G-Type Receptor 3 |
| NMI | N-Myc And STAT Interactor |
| NUMBL | NUMB Like Endocytic Adaptor Protein |
| MKNK2 | MAPK Interacting Serine/Threonine Kinase 2 |
| MIR371A | MicroRNA 371a |
| EIF4G3 | Eukaryotic Translation Initiation Factor 4 Gamma 3 |
| KDM2B | Lysine Demethylase 2B |
| SHMT1 | Serine Hydroxymethyltransferase 1 |
| NCAPG | Non-SMC Condensin I Complex Subunit G |
| CSNK1E | Casein Kinase 1 Epsilon |
| GFRA2 | GDNF Family Receptor Alpha 2 |
| DSTN | Destrin, Actin Depolymerizing Factor |
| LOXL1 | Lysyl Oxidase Like 1 |
| SIM2 | SIM BHLH Transcription Factor 2 |
| SNHG14 | Small Nucleolar RNA Host Gene 14 |
| UQCRB | Ubiquinol-Cytochrome C Reductase Binding Protein |
| SKAP2 | Src Kinase Associated Phosphoprotein 2 |
| TMEM92 | Transmembrane Protein 92 |
| HOXB3 | Homeobox B3 |
| THSD7B | Thrombospondin Type 1 Domain Containing 7B |
| NKD2 | NKD Inhibitor Of WNT Signaling Pathway 2 |
| TNFRSF4 | TNF Receptor Superfamily Member 4 |
| DAPK1-IT1 | DAPK1 Intronic Transcript 1 |
| SMARCE1 | SWI/SNF Related, Matrix Associated, Actin Dependent Regulator Of Chromatin, Subfamily E, Member 1 |
| CCL8 | C-C Motif Chemokine Ligand 8 |
| RIF1 | Replication Timing Regulatory Factor 1 |
| IMPDH2 | Inosine Monophosphate Dehydrogenase 2 |
| HAT1 | Histone Acetyltransferase 1 |
| LMAN1 | Lectin, Mannose Binding 1 |
| RORC | RAR Related Orphan Receptor C |
| PSME3 | Proteasome Activator Subunit 3 |
| CSE1L | Chromosome Segregation 1 Like |
| HOXA13 | Homeobox A13 |
| NPPC | Natriuretic Peptide C |
| SDHA | Succinate Dehydrogenase Complex Flavoprotein Subunit A |
| TXNL1 | Thioredoxin Like 1 |
| PLK3 | Polo Like Kinase 3 |
| ZBED3 | Zinc Finger BED-Type Containing 3 |
| IRF7 | Interferon Regulatory Factor 7 |
| ZIC2 | Zic Family Member 2 |
| ATP5F1A | ATP Synthase F1 Subunit Alpha |
| NACA | Nascent Polypeptide Associated Complex Subunit Alpha |
| BTF3 | Basic Transcription Factor 3 |
| ARHGDIG | Rho GDP Dissociation Inhibitor Gamma |
| EI24 | EI24 Autophagy Associated Transmembrane Protein |
| PFDN5 | Prefoldin Subunit 5 |
| SCARB1 | Scavenger Receptor Class B Member 1 |
| SOCS2 | Suppressor Of Cytokine Signaling 2 |
| SON | SON DNA Binding Protein |
| PCBP3 | Poly(RC) Binding Protein 3 |
| MIR891B | MicroRNA 891b |
| DHX9 | DExH-Box Helicase 9 |
| LAMB2 | Laminin Subunit Beta 2 |
| RCC1 | Regulator Of Chromosome Condensation 1 |
| PLSCR1 | Phospholipid Scramblase 1 |
| CLOCK | Clock Circadian Regulator |
| PRDM1 | PR/SET Domain 1 |
| SLC28A2 | Solute Carrier Family 28 Member 2 |
| ITGA4 | Integrin Subunit Alpha 4 |
| PPP1CB | Protein Phosphatase 1 Catalytic Subunit Beta |
| UIMC1 | Ubiquitin Interaction Motif Containing 1 |
| HYOU1 | Hypoxia Up-Regulated 1 |
| FUCA1 | Alpha-L-Fucosidase 1 |
| DST | Dystonin |
| TCTE3 | T-Complex-Associated-Testis-Expressed 3 |
| NPBWR1 | Neuropeptides B And W Receptor 1 |
| LTK | Leukocyte Receptor Tyrosine Kinase |
| RAB38 | RAB38, Member RAS Oncogene Family |
| BOP1 | BOP1 Ribosomal Biogenesis Factor |
| TM4SF18 | Transmembrane 4 L Six Family Member 18 |
| RRAS | RAS Related |
| CACNA1H | Calcium Voltage-Gated Channel Subunit Alpha1 H |
| CARD10 | Caspase Recruitment Domain Family Member 10 |
| TCP1 | T-Complex 1 |
| MIR365A | MicroRNA 365a |
| FMR1 | FMRP Translational Regulator 1 |
| CCAR2 | Cell Cycle And Apoptosis Regulator 2 |
| PABPC1 | Poly(A) Binding Protein Cytoplasmic 1 |
| HTR1D | 5-Hydroxytryptamine Receptor 1D |
| PROK2 | Prokineticin 2 |
| PROKR2 | Prokineticin Receptor 2 |
| NOB1 | NIN1 (RPN12) Binding Protein 1 Homolog |
| PDE2A | Phosphodiesterase 2A |
| TRIP6 | Thyroid Hormone Receptor Interactor 6 |
| COL6A1 | Collagen Type VI Alpha 1 Chain |
| CLEC7A | C-Type Lectin Domain Containing 7A |
| NKD1 | NKD Inhibitor Of WNT Signaling Pathway 1 |
| CDK11A | Cyclin Dependent Kinase 11A |
| LIMD1 | LIM Domains Containing 1 |
| HNRNPD | Heterogeneous Nuclear Ribonucleoprotein D |
| WIPF1 | WAS/WASL Interacting Protein Family Member 1 |
| BIN3 | Bridging Integrator 3 |
| BIRC6 | Baculoviral IAP Repeat Containing 6 |
| PHF10 | PHD Finger Protein 10 |
| RANGAP1 | Ran GTPase Activating Protein 1 |
| S1PR1 | Sphingosine-1-Phosphate Receptor 1 |
| SMYD3 | SET And MYND Domain Containing 3 |
| DDX5 | DEAD-Box Helicase 5 |
| MAP3K10 | Mitogen-Activated Protein Kinase Kinase Kinase 10 |
| LAS1L | LAS1 Like Ribosome Biogenesis Factor |
| MTUS1 | Microtubule Associated Scaffold Protein 1 |
| CYP2B6 | Cytochrome P450 Family 2 Subfamily B Member 6 |
| EIF3I | Eukaryotic Translation Initiation Factor 3 Subunit I |
| ASH2L | ASH2 Like, Histone Lysine Methyltransferase Complex Subunit |
| AAMP | Angio Associated Migratory Cell Protein |
| LOC111188163 | MSLN Promoter Region |
| GLIS3-AS1 | GLIS3 Antisense RNA 1 |
| MYOD1 | Myogenic Differentiation 1 |
| G3BP1 | G3BP Stress Granule Assembly Factor 1 |
| PROZ | Protein Z, Vitamin K Dependent Plasma Glycoprotein |
| ADORA3 | Adenosine A3 Receptor |
| NFKBIB | NFKB Inhibitor Beta |
| LAMA2 | Laminin Subunit Alpha 2 |
| PAFAH1B2 | Platelet Activating Factor Acetylhydrolase 1b Catalytic Subunit 2 |
| MDH1 | Malate Dehydrogenase 1 |
| NT5C1A | 5'-Nucleotidase, Cytosolic IA |
| NOSTRIN | Nitric Oxide Synthase Trafficking |
| IPO8 | Importin 8 |
| COP1 | COP1 E3 Ubiquitin Ligase |
| MIR379 | MicroRNA 379 |
| GNAI2 | G Protein Subunit Alpha I2 |
| CEP78 | Centrosomal Protein 78 |
| TRIM24 | Tripartite Motif Containing 24 |
| PSMD10 | Proteasome 26S Subunit, Non-ATPase 10 |
| FAM91A1 | Family With Sequence Similarity 91 Member A1 |
| ITCH | Itchy E3 Ubiquitin Protein Ligase |
| MAPK7 | Mitogen-Activated Protein Kinase 7 |
| AHCYL1 | Adenosylhomocysteinase Like 1 |
| NCR2 | Natural Cytotoxicity Triggering Receptor 2 |
| FBLN5 | Fibulin 5 |
| GRHL2 | Grainyhead Like Transcription Factor 2 |
| MIR489 | MicroRNA 489 |
| ASAH2 | N-Acylsphingosine Amidohydrolase 2 |
| LSR | Lipolysis Stimulated Lipoprotein Receptor |
| MRVI1 | Murine Retrovirus Integration Site 1 Homolog |
| ANGPTL1 | Angiopoietin Like 1 |
| BICD1 | BICD Cargo Adaptor 1 |
| RNF149 | Ring Finger Protein 149 |
| NDFIP2 | Nedd4 Family Interacting Protein 2 |
| RNF5P1 | Ring Finger Protein 5 Pseudogene 1 |
| LOC101929141 | Coiled-Coil Domain-Containing Protein 144A-Like |
| HEXIM1 | HEXIM P-TEFb Complex Subunit 1 |
| DLG5 | Discs Large MAGUK Scaffold Protein 5 |
| TOM1L1 | Target Of Myb1 Like 1 Membrane Trafficking Protein |
| PRKCH | Protein Kinase C Eta |
| ADH1A | Alcohol Dehydrogenase 1A (Class I), Alpha Polypeptide |
| WNK1 | WNK Lysine Deficient Protein Kinase 1 |
| POGZ | Pogo Transposable Element Derived With ZNF Domain |
| FCHSD2 | FCH And Double SH3 Domains 2 |
| C9orf78 | Chromosome 9 Open Reading Frame 78 |
| TRMO | TRNA Methyltransferase O |
| KAT2A | Lysine Acetyltransferase 2A |
| TBP | TATA-Box Binding Protein |
| CAB39 | Calcium Binding Protein 39 |
| PKP2 | Plakophilin 2 |
| HBB | Hemoglobin Subunit Beta |
| INSL3 | Insulin Like 3 |
| RORA | RAR Related Orphan Receptor A |
| MX1 | MX Dynamin Like GTPase 1 |
| PARVA | Parvin Alpha |
| MIR216B | MicroRNA 216b |
| LYPD3 | LY6/PLAUR Domain Containing 3 |
| ATP1A1 | ATPase Na+/K+ Transporting Subunit Alpha 1 |
| DNMBP | Dynamin Binding Protein |
| PLEKHA1 | Pleckstrin Homology Domain Containing A1 |
| ARIH1 | Ariadne RBR E3 Ubiquitin Protein Ligase 1 |
| H3C14 | H3 Clustered Histone 14 |
| APOH | Apolipoprotein H |
| TNXB | Tenascin XB |
| IPO4 | Importin 4 |
| CPA5 | Carboxypeptidase A5 |
| MAP3K2 | Mitogen-Activated Protein Kinase Kinase Kinase 2 |
| KRT6B | Keratin 6B |
| DGCR8 | DGCR8 Microprocessor Complex Subunit |
| FKBP4 | FKBP Prolyl Isomerase 4 |
| ENDOG | Endonuclease G |
| RICTOR | RPTOR Independent Companion Of MTOR Complex 2 |
| ATL1 | Atlastin GTPase 1 |
| RSPO1 | R-Spondin 1 |
| USP2 | Ubiquitin Specific Peptidase 2 |
| NADK | NAD Kinase |
| PBXIP1 | PBX Homeobox Interacting Protein 1 |
| RNF13 | Ring Finger Protein 13 |
| NOP53 | NOP53 Ribosome Biogenesis Factor |
| SLC26A2 | Solute Carrier Family 26 Member 2 |
| DSC2 | Desmocollin 2 |
| SLK | STE20 Like Kinase |
| DDIT4 | DNA Damage Inducible Transcript 4 |
| IRX4 | Iroquois Homeobox 4 |
| LTBP3 | Latent Transforming Growth Factor Beta Binding Protein 3 |
| MIR655 | MicroRNA 655 |
| IL13RA1 | Interleukin 13 Receptor Subunit Alpha 1 |
| GCH1 | GTP Cyclohydrolase 1 |
| PDGFC | Platelet Derived Growth Factor C |
| HEPH | Hephaestin |
| EHD1 | EH Domain Containing 1 |
| MBD2 | Methyl-CpG Binding Domain Protein 2 |
| SRPK2 | SRSF Protein Kinase 2 |
| USP5 | Ubiquitin Specific Peptidase 5 |
| ITSN2 | Intersectin 2 |
| PRR14 | Proline Rich 14 |
| DDX1 | DEAD-Box Helicase 1 |
| TRAPPC10 | Trafficking Protein Particle Complex 10 |
| CYRIB | CYFIP Related Rac1 Interactor B |
| SLC26A4 | Solute Carrier Family 26 Member 4 |
| DNAJB6 | DnaJ Heat Shock Protein Family (Hsp40) Member B6 |
| ADAM15 | ADAM Metallopeptidase Domain 15 |
| TNFRSF12A | TNF Receptor Superfamily Member 12A |
| AFDN | Afadin, Adherens Junction Formation Factor |
| F8 | Coagulation Factor VIII |
| MIR1246 | MicroRNA 1246 |
| PLAAT4 | Phospholipase A And Acyltransferase 4 |
| STK3 | Serine/Threonine Kinase 3 |
| EPS8L2 | EPS8 Like 2 |
| S100A16 | S100 Calcium Binding Protein A16 |
| EEF1D | Eukaryotic Translation Elongation Factor 1 Delta |
| FOLH1B | Folate Hydrolase 1B |
| MEGF6 | Multiple EGF Like Domains 6 |
| KRT3 | Keratin 3 |
| SORT1 | Sortilin 1 |
| LOXL3 | Lysyl Oxidase Like 3 |
| SERPINA10 | Serpin Family A Member 10 |
| TRPC3 | Transient Receptor Potential Cation Channel Subfamily C Member 3 |
| CD2AP | CD2 Associated Protein |
| ARMC3 | Armadillo Repeat Containing 3 |
| UBBP1 | Ubiquitin B Pseudogene 1 |
| STARP1 | Steroidogenic Acute Regulatory Protein Pseudogene 1 |
| RPL23AP27 | Ribosomal Protein L23a Pseudogene 27 |
| CAPN1 | Calpain 1 |
| NUCB1 | Nucleobindin 1 |
| NUP88 | Nucleoporin 88 |
| ASAP2 | ArfGAP With SH3 Domain, Ankyrin Repeat And PH Domain 2 |
| APPL2 | Adaptor Protein, Phosphotyrosine Interacting With PH Domain And Leucine Zipper 2 |
| MARCHF7 | Membrane Associated Ring-CH-Type Finger 7 |
| HTRA2 | HtrA Serine Peptidase 2 |
| PARD6A | Par-6 Family Cell Polarity Regulator Alpha |
| PPP5C | Protein Phosphatase 5 Catalytic Subunit |
| NRCAM | Neuronal Cell Adhesion Molecule |
| ARHGAP17 | Rho GTPase Activating Protein 17 |
| CHMP3 | Charged Multivesicular Body Protein 3 |
| CRCP | CGRP Receptor Component |
| CHST14 | Carbohydrate Sulfotransferase 14 |
| ITM2B | Integral Membrane Protein 2B |
| SIAH1 | Siah E3 Ubiquitin Protein Ligase 1 |
| NMUR1 | Neuromedin U Receptor 1 |
| LAMA1 | Laminin Subunit Alpha 1 |
| EFNB1 | Ephrin B1 |
| NRG3 | Neuregulin 3 |
| NRG4 | Neuregulin 4 |
| SNCA | Synuclein Alpha |
| NEO1 | Neogenin 1 |
| PPM1B | Protein Phosphatase, Mg2+/Mn2+ Dependent 1B |
| ITSN1 | Intersectin 1 |
| NFU1 | NFU1 Iron-Sulfur Cluster Scaffold |
| GAPDHS | Glyceraldehyde-3-Phosphate Dehydrogenase, Spermatogenic |
| CCNO | Cyclin O |
| UBE4B | Ubiquitination Factor E4B |
| PIK3AP1 | Phosphoinositide-3-Kinase Adaptor Protein 1 |
| GAPVD1 | GTPase Activating Protein And VPS9 Domains 1 |
| STAC | SH3 And Cysteine Rich Domain |
| SF3A3 | Splicing Factor 3a Subunit 3 |
| USP38 | Ubiquitin Specific Peptidase 38 |
| TCP11 | T-Complex 11 |
| NCKIPSD | NCK Interacting Protein With SH3 Domain |
| MPHOSPH8 | M-Phase Phosphoprotein 8 |
| MED21 | Mediator Complex Subunit 21 |
| WBP11 | WW Domain Binding Protein 11 |
| TTC1 | Tetratricopeptide Repeat Domain 1 |
| SH3BGR | SH3 Domain Binding Glutamate Rich Protein |
| DNAJA4 | DnaJ Heat Shock Protein Family (Hsp40) Member A4 |
| BZW1 | Basic Leucine Zipper And W2 Domains 1 |
| VCPIP1 | Valosin Containing Protein Interacting Protein 1 |
| HOXC11 | Homeobox C11 |
| DCUN1D4 | Defective In Cullin Neddylation 1 Domain Containing 4 |
| GKAP1 | G Kinase Anchoring Protein 1 |
| ZBTB37 | Zinc Finger And BTB Domain Containing 37 |
| NCKAP5 | NCK Associated Protein 5 |
| CFAP36 | Cilia And Flagella Associated Protein 36 |
| C2orf73 | Chromosome 2 Open Reading Frame 73 |
| BOD1L1 | Biorientation Of Chromosomes In Cell Division 1 Like 1 |
| BDKRB1 | Bradykinin Receptor B1 |
| CD109 | CD109 Molecule |
| IFI16 | Interferon Gamma Inducible Protein 16 |
| RFXANK | Regulatory Factor X Associated Ankyrin Containing Protein |
| PLAA | Phospholipase A2 Activating Protein |
| MAN2A1 | Mannosidase Alpha Class 2A Member 1 |
| HMOX2 | Heme Oxygenase 2 |
| PRSS27 | Serine Protease 27 |
| ST3GAL1 | ST3 Beta-Galactoside Alpha-2,3-Sialyltransferase 1 |
| STK38L | Serine/Threonine Kinase 38 Like |
| PRPF4 | Pre-MRNA Processing Factor 4 |
| SERPINB4 | Serpin Family B Member 4 |
| MYO1C | Myosin IC |
| EPX | Eosinophil Peroxidase |
| DCUN1D1 | Defective In Cullin Neddylation 1 Domain Containing 1 |
| PDE6D | Phosphodiesterase 6D |
| RAP1B | RAP1B, Member Of RAS Oncogene Family |
| ARF1 | ADP Ribosylation Factor 1 |
| DDX17 | DEAD-Box Helicase 17 |
| CBR1 | Carbonyl Reductase 1 |
| DUT | Deoxyuridine Triphosphatase |
| AEBP1 | AE Binding Protein 1 |
| INPP4B | Inositol Polyphosphate-4-Phosphatase Type II B |
| CA6 | Carbonic Anhydrase 6 |
| TCF12 | Transcription Factor 12 |
| SYT5 | Synaptotagmin 5 |
| YWHAG | Tyrosine 3-Monooxygenase/Tryptophan 5-Monooxygenase Activation Protein Gamma |
| SUPT6H | SPT6 Homolog, Histone Chaperone And Transcription Elongation Factor |
| ZYX | Zyxin |
| ADGRG2 | Adhesion G Protein-Coupled Receptor G2 |
| PGD | Phosphogluconate Dehydrogenase |
| TCIRG1 | T Cell Immune Regulator 1, ATPase H+ Transporting V0 Subunit A3 |
| NINL | Ninein Like |
| CTSH | Cathepsin H |
| TBXT | T-Box Transcription Factor T |
| DACT2 | Dishevelled Binding Antagonist Of Beta Catenin 2 |
| ANP32C | Acidic Nuclear Phosphoprotein 32 Family Member C |
| URGCP | Upregulator Of Cell Proliferation |
| C8orf17 | Chromosome 8 Open Reading Frame 17 |
| FCGRT | Fc Fragment Of IgG Receptor And Transporter |
| DLGAP1 | DLG Associated Protein 1 |
| DNAJC2 | DnaJ Heat Shock Protein Family (Hsp40) Member C2 |
| HNRNPC | Heterogeneous Nuclear Ribonucleoprotein C |
| IGKC | Immunoglobulin Kappa Constant |
| CDK11B | Cyclin Dependent Kinase 11B |
| KRT6A | Keratin 6A |
| AIF1 | Allograft Inflammatory Factor 1 |
| BTG2 | BTG Anti-Proliferation Factor 2 |
| SYNM | Synemin |
| UBE3A | Ubiquitin Protein Ligase E3A |
| PPL | Periplakin |
| DMP1 | Dentin Matrix Acidic Phosphoprotein 1 |
| PABPC4 | Poly(A) Binding Protein Cytoplasmic 4 |
| AHNAK2 | AHNAK Nucleoprotein 2 |
| MIR100HG | Mir-100-Let-7a-2-Mir-125b-1 Cluster Host Gene |
| PYGO2 | Pygopus Family PHD Finger 2 |
| AMHR2 | Anti-Mullerian Hormone Receptor Type 2 |
| MIB1 | Mindbomb E3 Ubiquitin Protein Ligase 1 |
| GATD3A | Glutamine Amidotransferase Like Class 1 Domain Containing 3A |
| ERVK-6 | Endogenous Retrovirus Group K Member 6, Envelope |
| FBLIM1 | Filamin Binding LIM Protein 1 |
| TMEM240 | Transmembrane Protein 240 |
| SCO2 | Synthesis Of Cytochrome C Oxidase 2 |
| PCID2 | PCI Domain Containing 2 |
| TXN2 | Thioredoxin 2 |
| MPZL2 | Myelin Protein Zero Like 2 |
| HNRNPU | Heterogeneous Nuclear Ribonucleoprotein U |
| SYNCRIP | Synaptotagmin Binding Cytoplasmic RNA Interacting Protein |
| HNRNPA3 | Heterogeneous Nuclear Ribonucleoprotein A3 |
| PRSS21 | Serine Protease 21 |
| EFEMP2 | EGF Containing Fibulin Extracellular Matrix Protein 2 |
| BTK | Bruton Tyrosine Kinase |
| PSD4 | Pleckstrin And Sec7 Domain Containing 4 |
| CCNK | Cyclin K |
| TINAGL1 | Tubulointerstitial Nephritis Antigen Like 1 |
| LGALS7B | Galectin 7B |
| CAPNS1 | Calpain Small Subunit 1 |
| CHD3 | Chromodomain Helicase DNA Binding Protein 3 |
| WNT10A | Wnt Family Member 10A |
| POLR3K | RNA Polymerase III Subunit K |
| NAA10 | N-Alpha-Acetyltransferase 10, NatA Catalytic Subunit |
| CCT5 | Chaperonin Containing TCP1 Subunit 5 |
| PROM2 | Prominin 2 |
| THRA | Thyroid Hormone Receptor Alpha |
| USP21 | Ubiquitin Specific Peptidase 21 |
| VANGL1 | VANGL Planar Cell Polarity Protein 1 |
| SEPTIN5 | Septin 5 |
| KRT78 | Keratin 78 |
| CCNDBP1 | Cyclin D1 Binding Protein 1 |
| SHARPIN | SHANK Associated RH Domain Interactor |
| CACUL1 | CDK2 Associated Cullin Domain 1 |
| ATG9A | Autophagy Related 9A |
| ATG2B | Autophagy Related 2B |
| KRT79 | Keratin 79 |
| CYP4B1 | Cytochrome P450 Family 4 Subfamily B Member 1 |
| MYO1B | Myosin IB |
| RBM39 | RNA Binding Motif Protein 39 |
| ATG9B | Autophagy Related 9B |
| PREX2 | Phosphatidylinositol-3,4,5-Trisphosphate Dependent Rac Exchange Factor 2 |
| CBFA2T3 | CBFA2/RUNX1 Partner Transcriptional Co-Repressor 3 |
| ITIH3 | Inter-Alpha-Trypsin Inhibitor Heavy Chain 3 |
| CD7 | CD7 Molecule |
| CCT7 | Chaperonin Containing TCP1 Subunit 7 |
| GLB1 | Galactosidase Beta 1 |
| STAU1 | Staufen Double-Stranded RNA Binding Protein 1 |
| KCNK9 | Potassium Two Pore Domain Channel Subfamily K Member 9 |
| HAND2 | Heart And Neural Crest Derivatives Expressed 2 |
| NTNG2 | Netrin G2 |
| TNFAIP8L2 | TNF Alpha Induced Protein 8 Like 2 |
| ADAMTS7P3 | ADAMTS7 Pseudogene 3 |
| POFUT1 | Protein O-Fucosyltransferase 1 |
| CD1D | CD1d Molecule |
| IMMT | Inner Membrane Mitochondrial Protein |
| CD2 | CD2 Molecule |
| CTPS1 | CTP Synthase 1 |
| NDUFS6 | NADH:Ubiquinone Oxidoreductase Subunit S6 |
| ARPC1B | Actin Related Protein 2/3 Complex Subunit 1B |
| VDAC3 | Voltage Dependent Anion Channel 3 |
| USP11 | Ubiquitin Specific Peptidase 11 |
| HNRNPH3 | Heterogeneous Nuclear Ribonucleoprotein H3 |
| FBXW7-AS1 | FBXW7 Antisense RNA 1 |
| PCGF2 | Polycomb Group Ring Finger 2 |
| CCHCR1 | Coiled-Coil Alpha-Helical Rod Protein 1 |
| GNB2 | G Protein Subunit Beta 2 |
| HLA-DRA | Major Histocompatibility Complex, Class II, DR Alpha |
| C1GALT1 | Core 1 Synthase, Glycoprotein-N-Acetylgalactosamine 3-Beta-Galactosyltransferase 1 |
| LYZ | Lysozyme |
| GRIN2B | Glutamate Ionotropic Receptor NMDA Type Subunit 2B |
| GSTK1 | Glutathione S-Transferase Kappa 1 |
| CCL23 | C-C Motif Chemokine Ligand 23 |
| IL21R | Interleukin 21 Receptor |
| GLS2 | Glutaminase 2 |
| ST6GALNAC2 | ST6 N-Acetylgalactosaminide Alpha-2,6-Sialyltransferase 2 |
| STMN3 | Stathmin 3 |
| EVPL | Envoplakin |
| B4GALT1 | Beta-1,4-Galactosyltransferase 1 |
| PTGDS | Prostaglandin D2 Synthase |
| B4GALT3 | Beta-1,4-Galactosyltransferase 3 |
| SLC12A1 | Solute Carrier Family 12 Member 1 |
| SLC15A2 | Solute Carrier Family 15 Member 2 |
| HSPA6 | Heat Shock Protein Family A (Hsp70) Member 6 |
| HNRNPA0 | Heterogeneous Nuclear Ribonucleoprotein A0 |
| OGA | O-GlcNAcase |
| MYO10 | Myosin X |
| TRPC6 | Transient Receptor Potential Cation Channel Subfamily C Member 6 |
| EBF1 | EBF Transcription Factor 1 |
| ARHGAP15 | Rho GTPase Activating Protein 15 |
| EBF3 | EBF Transcription Factor 3 |
| RRAGD | Ras Related GTP Binding D |
| MARCKSL1 | MARCKS Like 1 |
| MCF2 | MCF.2 Cell Line Derived Transforming Sequence |
