# Supplementary material for: Integrated Analyses Identify Immune-Related Signature Associated with Qingyihuaji Formula for Treatment of Pancreatic Ductal Adenocarcinoma Using Network Pharmacology and Weighted Gene Co-Expression Network
Source: J Immunol Res. 2020 May 20;2020:7503605. doi: 10.1155/2020/7503605 (PMC7256764; doi:10.1155/2020/7503605)
Supplement: Supplementary Materials — Table 1S. The PDAC genes in the GeneCards database Supplementary file 2: Table 2S. The immune-related targets in the GeneCards database Supplementary file 3: Table 3S. The detailed ADME parameters and the SMILES information of the aforementioned 67 selected compounds. Supplementary file 4: Figure S1. The potential active ingredients-target network of QYHJ. Supplementary file 5: Table 4S. The association between active ingredients and up-regulated overlapping genes. Supplementary file 6: Figure S2. Association of Methylation sites with the expression of 12 overlapped DEGs. [file 7503605.f1.zip › Supplementary file Table 2S.doc]

**Supplementary Table 2 The immune-related genes in the GeneCards database**

| Gene Symbol | Description |
| --- | --- |
| IL10 | Interleukin 10 |
| IFNG | Interferon Gamma |
| RAG1 | Recombination Activating 1 |
| IL6 | Interleukin 6 |
| JAK3 | Janus Kinase 3 |
| CD40LG | CD40 Ligand |
| TLR2 | Toll Like Receptor 2 |
| IL2RA | Interleukin 2 Receptor Subunit Alpha |
| IL7R | Interleukin 7 Receptor |
| TLR4 | Toll Like Receptor 4 |
| CTLA4 | Cytotoxic T-Lymphocyte Associated Protein 4 |
| HLA-DRB1 | Major Histocompatibility Complex, Class II, DR Beta 1 |
| FOXP3 | Forkhead Box P3 |
| TNF | Tumor Necrosis Factor |
| CD19 | CD19 Molecule |
| RAG2 | Recombination Activating 2 |
| ZAP70 | Zeta Chain Of T Cell Receptor Associated Protein Kinase 70 |
| CD79A | CD79a Molecule |
| CD40 | CD40 Molecule |
| STAT1 | Signal Transducer And Activator Of Transcription 1 |
| TLR3 | Toll Like Receptor 3 |
| TNFRSF13B | TNF Receptor Superfamily Member 13B |
| STAT3 | Signal Transducer And Activator Of Transcription 3 |
| FASLG | Fas Ligand |
| FAS | Fas Cell Surface Death Receptor |
| HLA-B | Major Histocompatibility Complex, Class I, B |
| CD4 | CD4 Molecule |
| IL2 | Interleukin 2 |
| HLA-A | Major Histocompatibility Complex, Class I, A |
| NOD2 | Nucleotide Binding Oligomerization Domain Containing 2 |
| PTPRC | Protein Tyrosine Phosphatase Receptor Type C |
| ADA | Adenosine Deaminase |
| IL2RG | Interleukin 2 Receptor Subunit Gamma |
| HLA-DQB1 | Major Histocompatibility Complex, Class II, DQ Beta 1 |
| CCL2 | C-C Motif Chemokine Ligand 2 |
| MYD88 | MYD88 Innate Immune Signal Transduction Adaptor |
| MBL2 | Mannose Binding Lectin 2 |
| IL4 | Interleukin 4 |
| STIM1 | Stromal Interaction Molecule 1 |
| IL2RB | Interleukin 2 Receptor Subunit Beta |
| ACP5 | Acid Phosphatase 5, Tartrate Resistant |
| NFKB1 | Nuclear Factor Kappa B Subunit 1 |
| C3 | Complement C3 |
| ICOS | Inducible T Cell Costimulator |
| FCGR2B | Fc Fragment Of IgG Receptor IIb |
| HLA-DQA1 | Major Histocompatibility Complex, Class II, DQ Alpha 1 |
| TNFRSF13C | TNF Receptor Superfamily Member 13C |
| PRF1 | Perforin 1 |
| PLCG2 | Phospholipase C Gamma 2 |
| SH2D1A | SH2 Domain Containing 1A |
| B2M | Beta-2-Microglobulin |
| IL7 | Interleukin 7 |
| IL12B | Interleukin 12B |
| PTPN22 | Protein Tyrosine Phosphatase Non-Receptor Type 22 |
| IL12RB1 | Interleukin 12 Receptor Subunit Beta 1 |
| TLR5 | Toll Like Receptor 5 |
| CCR5 | C-C Motif Chemokine Receptor 5 (Gene/Pseudogene) |
| FCGR2A | Fc Fragment Of IgG Receptor IIa |
| BTK | Bruton Tyrosine Kinase |
| TNFRSF1A | TNF Receptor Superfamily Member 1A |
| PDCD1 | Programmed Cell Death 1 |
| HAVCR2 | Hepatitis A Virus Cellular Receptor 2 |
| CIITA | Class II Major Histocompatibility Complex Transactivator |
| CCL3 | C-C Motif Chemokine Ligand 3 |
| TLR9 | Toll Like Receptor 9 |
| CR2 | Complement C3d Receptor 2 |
| IFIH1 | Interferon Induced With Helicase C Domain 1 |
| ORAI1 | ORAI Calcium Release-Activated Calcium Modulator 1 |
| DDX58 | DExD/H-Box Helicase 58 |
| IKBKG | Inhibitor Of Nuclear Factor Kappa B Kinase Regulatory Subunit Gamma |
| TGFB1 | Transforming Growth Factor Beta 1 |
| IL1B | Interleukin 1 Beta |
| TP53 | Tumor Protein P53 |
| ITGB2 | Integrin Subunit Beta 2 |
| MPO | Myeloperoxidase |
| CXCL8 | C-X-C Motif Chemokine Ligand 8 |
| ITGAM | Integrin Subunit Alpha M |
| CD8A | CD8a Molecule |
| IL13 | Interleukin 13 |
| AIRE | Autoimmune Regulator |
| IFNGR1 | Interferon Gamma Receptor 1 |
| NFKB2 | Nuclear Factor Kappa B Subunit 2 |
| CD274 | CD274 Molecule |
| CCR6 | C-C Motif Chemokine Receptor 6 |
| HLA-DPB1 | Major Histocompatibility Complex, Class II, DP Beta 1 |
| IL17A | Interleukin 17A |
| CD28 | CD28 Molecule |
| CXCL12 | C-X-C Motif Chemokine Ligand 12 |
| DOCK8 | Dedicator Of Cytokinesis 8 |
| STAT4 | Signal Transducer And Activator Of Transcription 4 |
| DCLRE1C | DNA Cross-Link Repair 1C |
| FCGR3B | Fc Fragment Of IgG Receptor IIIb |
| PNP | Purine Nucleoside Phosphorylase |
| HLA-C | Major Histocompatibility Complex, Class I, C |
| RIPK1 | Receptor Interacting Serine/Threonine Kinase 1 |
| PRTN3 | Proteinase 3 |
| CD247 | CD247 Molecule |
| IL18 | Interleukin 18 |
| IL5 | Interleukin 5 |
| IL4R | Interleukin 4 Receptor |
| C4B | Complement C4B (Chido Blood Group) |
| IRF5 | Interferon Regulatory Factor 5 |
| CARD11 | Caspase Recruitment Domain Family Member 11 |
| IRAK4 | Interleukin 1 Receptor Associated Kinase 4 |
| C4A | Complement C4A (Rodgers Blood Group) |
| GATA3 | GATA Binding Protein 3 |
| WAS | WASP Actin Nucleation Promoting Factor |
| HLA-G | Major Histocompatibility Complex, Class I, G |
| LRBA | LPS Responsive Beige-Like Anchor Protein |
| ITK | IL2 Inducible T Cell Kinase |
| CD86 | CD86 Molecule |
| ICAM1 | Intercellular Adhesion Molecule 1 |
| TREX1 | Three Prime Repair Exonuclease 1 |
| INS | Insulin |
| CD70 | CD70 Molecule |
| NLRP1 | NLR Family Pyrin Domain Containing 1 |
| NFKBIA | NFKB Inhibitor Alpha |
| TNFAIP3 | TNF Alpha Induced Protein 3 |
| IKBKB | Inhibitor Of Nuclear Factor Kappa B Kinase Subunit Beta |
| IL17F | Interleukin 17F |
| IL23R | Interleukin 23 Receptor |
| CCR1 | C-C Motif Chemokine Receptor 1 |
| TNFRSF4 | TNF Receptor Superfamily Member 4 |
| TYK2 | Tyrosine Kinase 2 |
| CASP8 | Caspase 8 |
| XIAP | X-Linked Inhibitor Of Apoptosis |
| CCL11 | C-C Motif Chemokine Ligand 11 |
| CD3E | CD3e Molecule |
| SPP1 | Secreted Phosphoprotein 1 |
| CCL5 | C-C Motif Chemokine Ligand 5 |
| CD209 | CD209 Molecule |
| CFP | Complement Factor Properdin |
| UNC13D | Unc-13 Homolog D |
| RAC2 | Rac Family Small GTPase 2 |
| BCL10 | BCL10 Immune Signaling Adaptor |
| IFNA1 | Interferon Alpha 1 |
| IL10RA | Interleukin 10 Receptor Subunit Alpha |
| JAK2 | Janus Kinase 2 |
| NHEJ1 | Non-Homologous End Joining Factor 1 |
| CFH | Complement Factor H |
| IL15 | Interleukin 15 |
| TLR7 | Toll Like Receptor 7 |
| RAB27A | RAB27A, Member RAS Oncogene Family |
| CD3D | CD3d Molecule |
| STING1 | Stimulator Of Interferon Response CGAMP Interactor 1 |
| LIG4 | DNA Ligase 4 |
| ICOSLG | Inducible T Cell Costimulator Ligand |
| CD81 | CD81 Molecule |
| HMGB1 | High Mobility Group Box 1 |
| TNFSF13B | TNF Superfamily Member 13b |
| MIF | Macrophage Migration Inhibitory Factor |
| C1QA | Complement C1q A Chain |
| TNFRSF1B | TNF Receptor Superfamily Member 1B |
| CSF2 | Colony Stimulating Factor 2 |
| ATM | ATM Serine/Threonine Kinase |
| CRP | C-Reactive Protein |
| CXCL10 | C-X-C Motif Chemokine Ligand 10 |
| IL21 | Interleukin 21 |
| CALR | Calreticulin |
| PRKCD | Protein Kinase C Delta |
| CLEC7A | C-Type Lectin Domain Containing 7A |
| HLA-DPA1 | Major Histocompatibility Complex, Class II, DP Alpha 1 |
| STAT5B | Signal Transducer And Activator Of Transcription 5B |
| IRF3 | Interferon Regulatory Factor 3 |
| C5 | Complement C5 |
| MIR155 | MicroRNA 155 |
| IRGM | Immunity Related GTPase M |
| TLR1 | Toll Like Receptor 1 |
| ALB | Albumin |
| CD80 | CD80 Molecule |
| MEFV | MEFV Innate Immuity Regulator, Pyrin |
| CD27 | CD27 Molecule |
| PIK3CD | Phosphatidylinositol-4,5-Bisphosphate 3-Kinase Catalytic Subunit Delta |
| PIK3R1 | Phosphoinositide-3-Kinase Regulatory Subunit 1 |
| STX11 | Syntaxin 11 |
| IL1RN | Interleukin 1 Receptor Antagonist |
| CARD9 | Caspase Recruitment Domain Family Member 9 |
| SYK | Spleen Associated Tyrosine Kinase |
| FCGR3A | Fc Fragment Of IgG Receptor IIIa |
| GUSB | Glucuronidase Beta |
| ITGAL | Integrin Subunit Alpha L |
| PTPN11 | Protein Tyrosine Phosphatase Non-Receptor Type 11 |
| IRF1 | Interferon Regulatory Factor 1 |
| BTNL2 | Butyrophilin Like 2 |
| IFNB1 | Interferon Beta 1 |
| CCND1 | Cyclin D1 |
| CFI | Complement Factor I |
| IRAK1 | Interleukin 1 Receptor Associated Kinase 1 |
| IL12A | Interleukin 12A |
| IDO1 | Indoleamine 2,3-Dioxygenase 1 |
| CX3CR1 | C-X3-C Motif Chemokine Receptor 1 |
| TLR8 | Toll Like Receptor 8 |
| MIR21 | MicroRNA 21 |
| AK2 | Adenylate Kinase 2 |
| C1S | Complement C1s |
| TLR6 | Toll Like Receptor 6 |
| CD59 | CD59 Molecule (CD59 Blood Group) |
| IGHE | Immunoglobulin Heavy Constant Epsilon |
| STXBP2 | Syntaxin Binding Protein 2 |
| MALT1 | MALT1 Paracaspase |
| FCGR2C | Fc Fragment Of IgG Receptor IIc (Gene/Pseudogene) |
| NLRP3 | NLR Family Pyrin Domain Containing 3 |
| IRF7 | Interferon Regulatory Factor 7 |
| ITGB3 | Integrin Subunit Beta 3 |
| MIR146A | MicroRNA 146a |
| LCK | LCK Proto-Oncogene, Src Family Tyrosine Kinase |
| IL1A | Interleukin 1 Alpha |
| CD14 | CD14 Molecule |
| SLC11A1 | Solute Carrier Family 11 Member 1 |
| HRAS | HRas Proto-Oncogene, GTPase |
| MS4A1 | Membrane Spanning 4-Domains A1 |
| AKT1 | AKT Serine/Threonine Kinase 1 |
| HLA-DRA | Major Histocompatibility Complex, Class II, DR Alpha |
| NRAS | NRAS Proto-Oncogene, GTPase |
| PSMB9 | Proteasome 20S Subunit Beta 9 |
| LAT | Linker For Activation Of T Cells |
| C1QC | Complement C1q C Chain |
| ELANE | Elastase, Neutrophil Expressed |
| DDX41 | DEAD-Box Helicase 41 |
| KLRK1 | Killer Cell Lectin Like Receptor K1 |
| IGHM | Immunoglobulin Heavy Constant Mu |
| TNFSF12 | TNF Superfamily Member 12 |
| CYBB | Cytochrome B-245 Beta Chain |
| NBN | Nibrin |
| WIPF1 | WAS/WASL Interacting Protein Family Member 1 |
| NCF2 | Neutrophil Cytosolic Factor 2 |
| VCAM1 | Vascular Cell Adhesion Molecule 1 |
| MSN | Moesin |
| PIK3CA | Phosphatidylinositol-4,5-Bisphosphate 3-Kinase Catalytic Subunit Alpha |
| IRF4 | Interferon Regulatory Factor 4 |
| CASP10 | Caspase 10 |
| C1QB | Complement C1q B Chain |
| TCIRG1 | T Cell Immune Regulator 1, ATPase H+ Transporting V0 Subunit A3 |
| CXCR4 | C-X-C Motif Chemokine Receptor 4 |
| RFXAP | Regulatory Factor X Associated Protein |
| CORO1A | Coronin 1A |
| EGFR | Epidermal Growth Factor Receptor |
| KRAS | KRAS Proto-Oncogene, GTPase |
| IL1R1 | Interleukin 1 Receptor Type 1 |
| TBK1 | TANK Binding Kinase 1 |
| RASGRP1 | RAS Guanyl Releasing Protein 1 |
| LTA | Lymphotoxin Alpha |
| TAP1 | Transporter 1, ATP Binding Cassette Subfamily B Member |
| PADI4 | Peptidyl Arginine Deiminase 4 |
| IL23A | Interleukin 23 Subunit Alpha |
| PTEN | Phosphatase And Tensin Homolog |
| MAGT1 | Magnesium Transporter 1 |
| CD244 | CD244 Molecule |
| DNMT3B | DNA Methyltransferase 3 Beta |
| KIT | KIT Proto-Oncogene, Receptor Tyrosine Kinase |
| RFXANK | Regulatory Factor X Associated Ankyrin Containing Protein |
| MPL | MPL Proto-Oncogene, Thrombopoietin Receptor |
| IFNGR2 | Interferon Gamma Receptor 2 |
| CR1 | Complement C3b/C4b Receptor 1 (Knops Blood Group) |
| GZMB | Granzyme B |
| NEU1 | Neuraminidase 1 |
| MBP | Myelin Basic Protein |
| ARPC1B | Actin Related Protein 2/3 Complex Subunit 1B |
| CGAS | Cyclic GMP-AMP Synthase |
| REL | REL Proto-Oncogene, NF-KB Subunit |
| BRAF | B-Raf Proto-Oncogene, Serine/Threonine Kinase |
| RFX5 | Regulatory Factor X5 |
| BRCA1 | BRCA1 DNA Repair Associated |
| ADAR | Adenosine Deaminase RNA Specific |
| SPINK5 | Serine Peptidase Inhibitor Kazal Type 5 |
| CD3G | CD3g Molecule |
| TRAF6 | TNF Receptor Associated Factor 6 |
| LYST | Lysosomal Trafficking Regulator |
| PSTPIP1 | Proline-Serine-Threonine Phosphatase Interacting Protein 1 |
| TBX21 | T-Box Transcription Factor 21 |
| RELA | RELA Proto-Oncogene, NF-KB Subunit |
| CSF3 | Colony Stimulating Factor 3 |
| IFNAR2 | Interferon Alpha And Beta Receptor Subunit 2 |
| CCL4 | C-C Motif Chemokine Ligand 4 |
| CSF1 | Colony Stimulating Factor 1 |
| IL17RA | Interleukin 17 Receptor A |
| FLG | Filaggrin |
| SAMHD1 | SAM And HD Domain Containing Deoxynucleoside Triphosphate Triphosphohydrolase 1 |
| C2 | Complement C2 |
| VWF | Von Willebrand Factor |
| TAP2 | Transporter 2, ATP Binding Cassette Subfamily B Member |
| ISG15 | ISG15 Ubiquitin Like Modifier |
| RMRP | RNA Component Of Mitochondrial RNA Processing Endoribonuclease |
| CD69 | CD69 Molecule |
| FCGR1A | Fc Fragment Of IgG Receptor Ia |
| TNFSF4 | TNF Superfamily Member 4 |
| MAVS | Mitochondrial Antiviral Signaling Protein |
| DNASE1 | Deoxyribonuclease 1 |
| CD46 | CD46 Molecule |
| BCL6 | BCL6 Transcription Repressor |
| NLRC4 | NLR Family CARD Domain Containing 4 |
| MAPK1 | Mitogen-Activated Protein Kinase 1 |
| BCL2 | BCL2 Apoptosis Regulator |
| MTOR | Mechanistic Target Of Rapamycin Kinase |
| TLR10 | Toll Like Receptor 10 |
| LTF | Lactotransferrin |
| UNG | Uracil DNA Glycosylase |
| TNFSF11 | TNF Superfamily Member 11 |
| KIR3DL1 | Killer Cell Immunoglobulin Like Receptor, Three Ig Domains And Long Cytoplasmic Tail 1 |
| DKC1 | Dyskerin Pseudouridine Synthase 1 |
| MYC | MYC Proto-Oncogene, BHLH Transcription Factor |
| AICDA | Activation Induced Cytidine Deaminase |
| IRF8 | Interferon Regulatory Factor 8 |
| CAMP | Cathelicidin Antimicrobial Peptide |
| IL9 | Interleukin 9 |
| SFTPD | Surfactant Protein D |
| FOXN1 | Forkhead Box N1 |
| PRKDC | Protein Kinase, DNA-Activated, Catalytic Subunit |
| SELL | Selectin L |
| CCR7 | C-C Motif Chemokine Receptor 7 |
| MECP2 | Methyl-CpG Binding Protein 2 |
| NOS2 | Nitric Oxide Synthase 2 |
| BRCA2 | BRCA2 DNA Repair Associated |
| LEP | Leptin |
| SLAMF1 | Signaling Lymphocytic Activation Molecule Family Member 1 |
| MAPK14 | Mitogen-Activated Protein Kinase 14 |
| CXCR3 | C-X-C Motif Chemokine Receptor 3 |
| IL18R1 | Interleukin 18 Receptor 1 |
| CHUK | Component Of Inhibitor Of Nuclear Factor Kappa B Kinase Complex |
| TRIM21 | Tripartite Motif Containing 21 |
| CDH1 | Cadherin 1 |
| SBDS | SBDS Ribosome Maturation Factor |
| MPZ | Myelin Protein Zero |
| CD55 | CD55 Molecule (Cromer Blood Group) |
| ADA2 | Adenosine Deaminase 2 |
| CYBA | Cytochrome B-245 Alpha Chain |
| ZNF627 | Zinc Finger Protein 627 |
| HBB | Hemoglobin Subunit Beta |
| RELB | RELB Proto-Oncogene, NF-KB Subunit |
| SH2B3 | SH2B Adaptor Protein 3 |
| TICAM1 | Toll Like Receptor Adaptor Molecule 1 |
| CFB | Complement Factor B |
| PGM3 | Phosphoglucomutase 3 |
| HLA-E | Major Histocompatibility Complex, Class I, E |
| LYN | LYN Proto-Oncogene, Src Family Tyrosine Kinase |
| JUN | Jun Proto-Oncogene, AP-1 Transcription Factor Subunit |
| ITGA2B | Integrin Subunit Alpha 2b |
| IL16 | Interleukin 16 |
| IL10RB | Interleukin 10 Receptor Subunit Beta |
| TTC7A | Tetratricopeptide Repeat Domain 7A |
| HP | Haptoglobin |
| EPX | Eosinophil Peroxidase |
| MYH7 | Myosin Heavy Chain 7 |
| SRC | SRC Proto-Oncogene, Non-Receptor Tyrosine Kinase |
| VEGFA | Vascular Endothelial Growth Factor A |
| TRAF3 | TNF Receptor Associated Factor 3 |
| MIR17 | MicroRNA 17 |
| FOS | Fos Proto-Oncogene, AP-1 Transcription Factor Subunit |
| S100A9 | S100 Calcium Binding Protein A9 |
| CD79B | CD79b Molecule |
| CXCL9 | C-X-C Motif Chemokine Ligand 9 |
| TRAF3IP2 | TRAF3 Interacting Protein 2 |
| HBA1 | Hemoglobin Subunit Alpha 1 |
| TYROBP | Transmembrane Immune Signaling Adaptor TYROBP |
| C5AR1 | Complement C5a Receptor 1 |
| HSPD1 | Heat Shock Protein Family D (Hsp60) Member 1 |
| TIRAP | TIR Domain Containing Adaptor Protein |
| JAK1 | Janus Kinase 1 |
| SMAD3 | SMAD Family Member 3 |
| MIR150 | MicroRNA 150 |
| SLAMF6 | SLAM Family Member 6 |
| BLNK | B Cell Linker |
| TGFB2 | Transforming Growth Factor Beta 2 |
| IL3 | Interleukin 3 |
| RIPK2 | Receptor Interacting Serine/Threonine Kinase 2 |
| SMARCAL1 | SWI/SNF Related, Matrix Associated, Actin Dependent Regulator Of Chromatin, Subfamily A Like 1 |
| FLT4 | Fms Related Receptor Tyrosine Kinase 4 |
| FCN3 | Ficolin 3 |
| NR3C1 | Nuclear Receptor Subfamily 3 Group C Member 1 |
| IL33 | Interleukin 33 |
| ITGA4 | Integrin Subunit Alpha 4 |
| SOCS3 | Suppressor Of Cytokine Signaling 3 |
| XRCC4 | X-Ray Repair Cross Complementing 4 |
| CD1D | CD1d Molecule |
| ALOX5 | Arachidonate 5-Lipoxygenase |
| PSMB8 | Proteasome 20S Subunit Beta 8 |
| LEPR | Leptin Receptor |
| VDR | Vitamin D Receptor |
| CASP1 | Caspase 1 |
| CD38 | CD38 Molecule |
| CXCR1 | C-X-C Motif Chemokine Receptor 1 |
| NOD1 | Nucleotide Binding Oligomerization Domain Containing 1 |
| MYO5A | Myosin VA |
| DNASE1L3 | Deoxyribonuclease 1 Like 3 |
| BLK | BLK Proto-Oncogene, Src Family Tyrosine Kinase |
| RAC1 | Rac Family Small GTPase 1 |
| H2AC18 | H2A Clustered Histone 18 |
| PIK3CG | Phosphatidylinositol-4,5-Bisphosphate 3-Kinase Catalytic Subunit Gamma |
| POU2AF1 | POU Class 2 Homeobox Associating Factor 1 |
| XRCC5 | X-Ray Repair Cross Complementing 5 |
| TAPBP | TAP Binding Protein |
| FYN | FYN Proto-Oncogene, Src Family Tyrosine Kinase |
| TNFSF15 | TNF Superfamily Member 15 |
| HBA2 | Hemoglobin Subunit Alpha 2 |
| APOE | Apolipoprotein E |
| ADAM17 | ADAM Metallopeptidase Domain 17 |
| NCF4 | Neutrophil Cytosolic Factor 4 |
| MMP2 | Matrix Metallopeptidase 2 |
| TRIM5 | Tripartite Motif Containing 5 |
| S100A8 | S100 Calcium Binding Protein A8 |
| TFRC | Transferrin Receptor |
| IL6R | Interleukin 6 Receptor |
| CDK4 | Cyclin Dependent Kinase 4 |
| FGFR3 | Fibroblast Growth Factor Receptor 3 |
| PTPN2 | Protein Tyrosine Phosphatase Non-Receptor Type 2 |
| MMP9 | Matrix Metallopeptidase 9 |
| ERCC6 | ERCC Excision Repair 6, Chromatin Remodeling Factor |
| IFNA2 | Interferon Alpha 2 |
| TRAC | T Cell Receptor Alpha Constant |
| NCF1 | Neutrophil Cytosolic Factor 1 |
| PPARG | Peroxisome Proliferator Activated Receptor Gamma |
| CXCL1 | C-X-C Motif Chemokine Ligand 1 |
| CLEC4M | C-Type Lectin Domain Family 4 Member M |
| ITGAX | Integrin Subunit Alpha X |
| IFI16 | Interferon Gamma Inducible Protein 16 |
| STAT5A | Signal Transducer And Activator Of Transcription 5A |
| ITCH | Itchy E3 Ubiquitin Protein Ligase |
| UNC93B1 | Unc-93 Homolog B1, TLR Signaling Regulator |
| DEFB4A | Defensin Beta 4A |
| THPO | Thrombopoietin |
| MX1 | MX Dynamin Like GTPase 1 |
| TG | Thyroglobulin |
| CCR2 | C-C Motif Chemokine Receptor 2 |
| CTPS1 | CTP Synthase 1 |
| TNFRSF17 | TNF Receptor Superfamily Member 17 |
| C1R | Complement C1r |
| STAT6 | Signal Transducer And Activator Of Transcription 6 |
| FGFR1 | Fibroblast Growth Factor Receptor 1 |
| SELE | Selectin E |
| SLPI | Secretory Leukocyte Peptidase Inhibitor |
| MOG | Myelin Oligodendrocyte Glycoprotein |
| CHD7 | Chromodomain Helicase DNA Binding Protein 7 |
| TNFRSF18 | TNF Receptor Superfamily Member 18 |
| LCN2 | Lipocalin 2 |
| PMP22 | Peripheral Myelin Protein 22 |
| CCR3 | C-C Motif Chemokine Receptor 3 |
| SELP | Selectin P |
| MASP2 | Mannan Binding Lectin Serine Peptidase 2 |
| CD44 | CD44 Molecule (Indian Blood Group) |
| FADD | Fas Associated Via Death Domain |
| TERT | Telomerase Reverse Transcriptase |
| TPMT | Thiopurine S-Methyltransferase |
| CFTR | CF Transmembrane Conductance Regulator |
| TREM2 | Triggering Receptor Expressed On Myeloid Cells 2 |
| IL22 | Interleukin 22 |
| PRL | Prolactin |
| PRPS1 | Phosphoribosyl Pyrophosphate Synthetase 1 |
| TNFRSF8 | TNF Receptor Superfamily Member 8 |
| MRE11 | MRE11 Homolog, Double Strand Break Repair Nuclease |
| FLT3 | Fms Related Receptor Tyrosine Kinase 3 |
| IGH | Immunoglobulin Heavy Locus |
| CTSG | Cathepsin G |
| HSP90AA1 | Heat Shock Protein 90 Alpha Family Class A Member 1 |
| BLM | BLM RecQ Like Helicase |
| APOH | Apolipoprotein H |
| ETS1 | ETS Proto-Oncogene 1, Transcription Factor |
| IL17RC | Interleukin 17 Receptor C |
| SOCS1 | Suppressor Of Cytokine Signaling 1 |
| MAPK8 | Mitogen-Activated Protein Kinase 8 |
| GBA | Glucosylceramidase Beta |
| ENG | Endoglin |
| FCN2 | Ficolin 2 |
| MICA | MHC Class I Polypeptide-Related Sequence A |
| PSMB4 | Proteasome 20S Subunit Beta 4 |
| POMC | Proopiomelanocortin |
| FCGRT | Fc Fragment Of IgG Receptor And Transporter |
| CCL20 | C-C Motif Chemokine Ligand 20 |
| HSPG2 | Heparan Sulfate Proteoglycan 2 |
| UBE2L3 | Ubiquitin Conjugating Enzyme E2 L3 |
| HLA-DMA | Major Histocompatibility Complex, Class II, DM Alpha |
| ITGB1 | Integrin Subunit Beta 1 |
| HLA-DRB5 | Major Histocompatibility Complex, Class II, DR Beta 5 |
| TNIP1 | TNFAIP3 Interacting Protein 1 |
| CDKN1A | Cyclin Dependent Kinase Inhibitor 1A |
| SERPINA3 | Serpin Family A Member 3 |
| CASP3 | Caspase 3 |
| TNFSF13 | TNF Superfamily Member 13 |
| INPP5D | Inositol Polyphosphate-5-Phosphatase D |
| LAG3 | Lymphocyte Activating 3 |
| IGHG1 | Immunoglobulin Heavy Constant Gamma 1 (G1m Marker) |
| BIRC3 | Baculoviral IAP Repeat Containing 3 |
| LBR | Lamin B Receptor |
| RNASE3 | Ribonuclease A Family Member 3 |
| ITGA2 | Integrin Subunit Alpha 2 |
| CD1A | CD1a Molecule |
| MMP1 | Matrix Metallopeptidase 1 |
| ERAP1 | Endoplasmic Reticulum Aminopeptidase 1 |
| TNFRSF14 | TNF Receptor Superfamily Member 14 |
| PTX3 | Pentraxin 3 |
| RAP1A | RAP1A, Member Of RAS Oncogene Family |
| GATA2 | GATA Binding Protein 2 |
| SLAMF7 | SLAM Family Member 7 |
| OCLN | Occludin |
| TPO | Thyroid Peroxidase |
| MUSK | Muscle Associated Receptor Tyrosine Kinase |
| RAF1 | Raf-1 Proto-Oncogene, Serine/Threonine Kinase |
| TMPO | Thymopoietin |
| TNFSF10 | TNF Superfamily Member 10 |
| IGF1 | Insulin Like Growth Factor 1 |
| CCR4 | C-C Motif Chemokine Receptor 4 |
| RPL5 | Ribosomal Protein L5 |
| MYO9B | Myosin IXB |
| IGKC | Immunoglobulin Kappa Constant |
| IL36RN | Interleukin 36 Receptor Antagonist |
| MIR34A | MicroRNA 34a |
| C7 | Complement C7 |
| TSLP | Thymic Stromal Lymphopoietin |
| CTNNB1 | Catenin Beta 1 |
| FCER2 | Fc Fragment Of IgE Receptor II |
| TBX1 | T-Box Transcription Factor 1 |
| PIEZO1 | Piezo Type Mechanosensitive Ion Channel Component 1 |
| NOTCH1 | Notch Receptor 1 |
| PTGS2 | Prostaglandin-Endoperoxide Synthase 2 |
| XRCC6 | X-Ray Repair Cross Complementing 6 |
| CLEC12A | C-Type Lectin Domain Family 12 Member A |
| NUP107 | Nucleoporin 107 |
| ICAM2 | Intercellular Adhesion Molecule 2 |
| ALK | ALK Receptor Tyrosine Kinase |
| CASR | Calcium Sensing Receptor |
| LY96 | Lymphocyte Antigen 96 |
| CBL | Cbl Proto-Oncogene |
| SERPINA1 | Serpin Family A Member 1 |
| OTULIN | OTU Deubiquitinase With Linear Linkage Specificity |
| CD34 | CD34 Molecule |
| S100A12 | S100 Calcium Binding Protein A12 |
| C8B | Complement C8 Beta Chain |
| KMT2D | Lysine Methyltransferase 2D |
| MAPK3 | Mitogen-Activated Protein Kinase 3 |
| P2RX7 | Purinergic Receptor P2X 7 |
| CD22 | CD22 Molecule |
| MUC1 | Mucin 1, Cell Surface Associated |
| DPP4 | Dipeptidyl Peptidase 4 |
| VEGFC | Vascular Endothelial Growth Factor C |
| CXCL13 | C-X-C Motif Chemokine Ligand 13 |
| OAS1 | 2'-5'-Oligoadenylate Synthetase 1 |
| CSF3R | Colony Stimulating Factor 3 Receptor |
| TEC | Tec Protein Tyrosine Kinase |
| MS4A2 | Membrane Spanning 4-Domains A2 |
| TNFRSF11A | TNF Receptor Superfamily Member 11a |
| COMT | Catechol-O-Methyltransferase |
| IFNL3 | Interferon Lambda 3 |
| VAV1 | Vav Guanine Nucleotide Exchange Factor 1 |
| MICB | MHC Class I Polypeptide-Related Sequence B |
| MIR140 | MicroRNA 140 |
| CLU | Clusterin |
| TYR | Tyrosinase |
| LILRB1 | Leukocyte Immunoglobulin Like Receptor B1 |
| EP300 | E1A Binding Protein P300 |
| CDC42 | Cell Division Cycle 42 |
| HLA-DRB3 | Major Histocompatibility Complex, Class II, DR Beta 3 |
| AIM2 | Absent In Melanoma 2 |
| HLA-DQA2 | Major Histocompatibility Complex, Class II, DQ Alpha 2 |
| ICAM3 | Intercellular Adhesion Molecule 3 |
| KLRD1 | Killer Cell Lectin Like Receptor D1 |
| CTSC | Cathepsin C |
| EIF2AK2 | Eukaryotic Translation Initiation Factor 2 Alpha Kinase 2 |
| NLRP12 | NLR Family Pyrin Domain Containing 12 |
| ABL1 | ABL Proto-Oncogene 1, Non-Receptor Tyrosine Kinase |
| BCL11B | BAF Chromatin Remodeling Complex Subunit BCL11B |
| SKIV2L | Ski2 Like RNA Helicase |
| PRDM1 | PR/SET Domain 1 |
| HCK | HCK Proto-Oncogene, Src Family Tyrosine Kinase |
| EPG5 | Ectopic P-Granules Autophagy Protein 5 Homolog |
| IFNAR1 | Interferon Alpha And Beta Receptor Subunit 1 |
| CD5 | CD5 Molecule |
| PML | Promyelocytic Leukemia |
| BMP6 | Bone Morphogenetic Protein 6 |
| KMT2A | Lysine Methyltransferase 2A |
| TIMP1 | TIMP Metallopeptidase Inhibitor 1 |
| FCRL3 | Fc Receptor Like 3 |
| CCL17 | C-C Motif Chemokine Ligand 17 |
| HLA-DRB4 | Major Histocompatibility Complex, Class II, DR Beta 4 |
| BCL2L1 | BCL2 Like 1 |
| GPI | Glucose-6-Phosphate Isomerase |
| LGALS3 | Galectin 3 |
| MMEL1 | Membrane Metalloendopeptidase Like 1 |
| CLEC4E | C-Type Lectin Domain Family 4 Member E |
| ANXA1 | Annexin A1 |
| CAT | Catalase |
| CD96 | CD96 Molecule |
| APOBEC3G | Apolipoprotein B MRNA Editing Enzyme Catalytic Subunit 3G |
| CHEK2 | Checkpoint Kinase 2 |
| RORC | RAR Related Orphan Receptor C |
| TSHR | Thyroid Stimulating Hormone Receptor |
| IL27 | Interleukin 27 |
| LIF | LIF Interleukin 6 Family Cytokine |
| NUP133 | Nucleoporin 133 |
| CBLIF | Cobalamin Binding Intrinsic Factor |
| EPHB4 | EPH Receptor B4 |
| ARG1 | Arginase 1 |
| IL12RB2 | Interleukin 12 Receptor Subunit Beta 2 |
| C8A | Complement C8 Alpha Chain |
| ABCB1 | ATP Binding Cassette Subfamily B Member 1 |
| KITLG | KIT Ligand |
| CCL21 | C-C Motif Chemokine Ligand 21 |
| GRB2 | Growth Factor Receptor Bound Protein 2 |
| TTC37 | Tetratricopeptide Repeat Domain 37 |
| HELLS | Helicase, Lymphoid Specific |
| NTRK1 | Neurotrophic Receptor Tyrosine Kinase 1 |
| BCR | BCR Activator Of RhoGEF And GTPase |
| GH1 | Growth Hormone 1 |
| F2 | Coagulation Factor II, Thrombin |
| DSG1 | Desmoglein 1 |
| CCL22 | C-C Motif Chemokine Ligand 22 |
| IL21R | Interleukin 21 Receptor |
| RHD | Rh Blood Group D Antigen |
| GATA1 | GATA Binding Protein 1 |
| CYBC1 | Cytochrome B-245 Chaperone 1 |
| NGF | Nerve Growth Factor |
| LIFR | LIF Receptor Subunit Alpha |
| KIAA0319L | KIAA0319 Like |
| HLA-DOA | Major Histocompatibility Complex, Class II, DO Alpha |
| JAGN1 | Jagunal Homolog 1 |
| LGALS1 | Galectin 1 |
| GP1BA | Glycoprotein Ib Platelet Subunit Alpha |
| COL2A1 | Collagen Type II Alpha 1 Chain |
| RNASEH2B | Ribonuclease H2 Subunit B |
| BIRC2 | Baculoviral IAP Repeat Containing 2 |
| KLRC1 | Killer Cell Lectin Like Receptor C1 |
| TRB | T Cell Receptor Beta Locus |
| MT-CO1 | Mitochondrially Encoded Cytochrome C Oxidase I |
| CCL19 | C-C Motif Chemokine Ligand 19 |
| F3 | Coagulation Factor III, Tissue Factor |
| ACKR2 | Atypical Chemokine Receptor 2 |
| NCAM1 | Neural Cell Adhesion Molecule 1 |
| IGLL1 | Immunoglobulin Lambda Like Polypeptide 1 |
| IL11 | Interleukin 11 |
| MASP1 | Mannan Binding Lectin Serine Peptidase 1 |
| ACE | Angiotensin I Converting Enzyme |
| MIR221 | MicroRNA 221 |
| EBI3 | Epstein-Barr Virus Induced 3 |
| SP1 | Sp1 Transcription Factor |
| ATG5 | Autophagy Related 5 |
| UMPS | Uridine Monophosphate Synthetase |
| PPIA | Peptidylprolyl Isomerase A |
| S100B | S100 Calcium Binding Protein B |
| CSF1R | Colony Stimulating Factor 1 Receptor |
| TPI1 | Triosephosphate Isomerase 1 |
| CXCR2 | C-X-C Motif Chemokine Receptor 2 |
| C9 | Complement C9 |
| CD84 | CD84 Molecule |
| CACNA1C | Calcium Voltage-Gated Channel Subunit Alpha1 C |
| NFATC2 | Nuclear Factor Of Activated T Cells 2 |
| AP3B1 | Adaptor Related Protein Complex 3 Subunit Beta 1 |
| CYLD | CYLD Lysine 63 Deubiquitinase |
| CXCR5 | C-X-C Motif Chemokine Receptor 5 |
| BAX | BCL2 Associated X, Apoptosis Regulator |
| LY9 | Lymphocyte Antigen 9 |
| IL6ST | Interleukin 6 Signal Transducer |
| G6PD | Glucose-6-Phosphate Dehydrogenase |
| HIRA | Histone Cell Cycle Regulator |
| TCF3 | Transcription Factor 3 |
| TF | Transferrin |
| LBP | Lipopolysaccharide Binding Protein |
| AGER | Advanced Glycosylation End-Product Specific Receptor |
| CD276 | CD276 Molecule |
| IL25 | Interleukin 25 |
| RNASEH2A | Ribonuclease H2 Subunit A |
| NOS3 | Nitric Oxide Synthase 3 |
| RNASEL | Ribonuclease L |
| SLC22A4 | Solute Carrier Family 22 Member 4 |
| AGRN | Agrin |
| BST2 | Bone Marrow Stromal Cell Antigen 2 |
| CD58 | CD58 Molecule |
| DHX58 | DExH-Box Helicase 58 |
| RNPC3 | RNA Binding Region (RNP1, RRM) Containing 3 |
| LACC1 | Laccase Domain Containing 1 |
| PMM2 | Phosphomannomutase 2 |
| HLA-DMB | Major Histocompatibility Complex, Class II, DM Beta |
| GP1BB | Glycoprotein Ib Platelet Subunit Beta |
| CSK | C-Terminal Src Kinase |
| IDH1 | Isocitrate Dehydrogenase (NADP(+)) 1 |
| PRKAR1A | Protein Kinase CAMP-Dependent Type I Regulatory Subunit Alpha |
| F2RL1 | F2R Like Trypsin Receptor 1 |
| MRAP | Melanocortin 2 Receptor Accessory Protein |
| FOXC2 | Forkhead Box C2 |
| AKT2 | AKT Serine/Threonine Kinase 2 |
| BTLA | B And T Lymphocyte Associated |
| MET | MET Proto-Oncogene, Receptor Tyrosine Kinase |
| BACH2 | BTB Domain And CNC Homolog 2 |
| MVK | Mevalonate Kinase |
| MIR16-1 | MicroRNA 16-1 |
| CDKN2A | Cyclin Dependent Kinase Inhibitor 2A |
| DNMT1 | DNA Methyltransferase 1 |
| PF4 | Platelet Factor 4 |
| IKZF1 | IKAROS Family Zinc Finger 1 |
| IL18RAP | Interleukin 18 Receptor Accessory Protein |
| TNFRSF10A | TNF Receptor Superfamily Member 10a |
| FLT3LG | Fms Related Receptor Tyrosine Kinase 3 Ligand |
| CCR9 | C-C Motif Chemokine Receptor 9 |
| ERBB2 | Erb-B2 Receptor Tyrosine Kinase 2 |
| GDNF | Glial Cell Derived Neurotrophic Factor |
| PDGFRA | Platelet Derived Growth Factor Receptor Alpha |
| HSPA4 | Heat Shock Protein Family A (Hsp70) Member 4 |
| EGF | Epidermal Growth Factor |
| MIR197 | MicroRNA 197 |
| PSEN1 | Presenilin 1 |
| IL1RAPL2 | Interleukin 1 Receptor Accessory Protein Like 2 |
| ITIH4 | Inter-Alpha-Trypsin Inhibitor Heavy Chain 4 |
| CXCL11 | C-X-C Motif Chemokine Ligand 11 |
| KRT1 | Keratin 1 |
| NSD2 | Nuclear Receptor Binding SET Domain Protein 2 |
| CREBBP | CREB Binding Protein |
| CDKN1B | Cyclin Dependent Kinase Inhibitor 1B |
| RBPJ | Recombination Signal Binding Protein For Immunoglobulin Kappa J Region |
| INAVA | Innate Immunity Activator |
| FN1 | Fibronectin 1 |
| UFD1 | Ubiquitin Recognition Factor In ER Associated Degradation 1 |
| PIGA | Phosphatidylinositol Glycan Anchor Biosynthesis Class A |
| DEFB1 | Defensin Beta 1 |
| MAP3K7 | Mitogen-Activated Protein Kinase Kinase Kinase 7 |
| ERVW-1 | Endogenous Retrovirus Group W Member 1, Envelope |
| CD180 | CD180 Molecule |
| MT-TF | Mitochondrially Encoded TRNA-Phe (UUU/C) |
| MT-TH | Mitochondrially Encoded TRNA-His (CAU/C) |
| SLC7A7 | Solute Carrier Family 7 Member 7 |
| MAN2B1 | Mannosidase Alpha Class 2B Member 1 |
| THSD1 | Thrombospondin Type 1 Domain Containing 1 |
| MIR200B | MicroRNA 200b |
| CD36 | CD36 Molecule |
| CCL18 | C-C Motif Chemokine Ligand 18 |
| HFE | Homeostatic Iron Regulator |
| MIR142 | MicroRNA 142 |
| HLA-DOB | Major Histocompatibility Complex, Class II, DO Beta |
| PYCARD | PYD And CARD Domain Containing |
| AP1S3 | Adaptor Related Protein Complex 1 Subunit Sigma 3 |
| SPN | Sialophorin |
| HIF1A | Hypoxia Inducible Factor 1 Subunit Alpha |
| LAMB2 | Laminin Subunit Beta 2 |
| SLC30A2 | Solute Carrier Family 30 Member 2 |
| USP18 | Ubiquitin Specific Peptidase 18 |
| CXCL2 | C-X-C Motif Chemokine Ligand 2 |
| KRT16 | Keratin 16 |
| IL32 | Interleukin 32 |
| TINF2 | TERF1 Interacting Nuclear Factor 2 |
| OSM | Oncostatin M |
| MSH2 | MutS Homolog 2 |
| RTEL1 | Regulator Of Telomere Elongation Helicase 1 |
| CLEC6A | C-Type Lectin Domain Containing 6A |
| TOP2A | DNA Topoisomerase II Alpha |
| MIR19A | MicroRNA 19a |
| CTSK | Cathepsin K |
| RNASEH2C | Ribonuclease H2 Subunit C |
| POLR3A | RNA Polymerase III Subunit A |
| MIR144 | MicroRNA 144 |
| CX3CL1 | C-X3-C Motif Chemokine Ligand 1 |
| FGA | Fibrinogen Alpha Chain |
| SMARCA4 | SWI/SNF Related, Matrix Associated, Actin Dependent Regulator Of Chromatin, Subfamily A, Member 4 |
| F8 | Coagulation Factor VIII |
| FANCA | FA Complementation Group A |
| TET2 | Tet Methylcytosine Dioxygenase 2 |
| CA2 | Carbonic Anhydrase 2 |
| MIR23B | MicroRNA 23b |
| MDM2 | MDM2 Proto-Oncogene |
| VIP | Vasoactive Intestinal Peptide |
| CLEC4D | C-Type Lectin Domain Family 4 Member D |
| IL37 | Interleukin 37 |
| KRT8 | Keratin 8 |
| FERMT3 | Fermitin Family Member 3 |
| GZMA | Granzyme A |
| RPS19 | Ribosomal Protein S19 |
| CD83 | CD83 Molecule |
| MIR20A | MicroRNA 20a |
| CD163 | CD163 Molecule |
| PTK2B | Protein Tyrosine Kinase 2 Beta |
| IRGC | Immunity Related GTPase Cinema |
| SLC29A3 | Solute Carrier Family 29 Member 3 |
| COPA | COPI Coat Complex Subunit Alpha |
| TTR | Transthyretin |
| MIR99A | MicroRNA 99a |
| TOLLIP | Toll Interacting Protein |
| GJA1 | Gap Junction Protein Alpha 1 |
| NLRC5 | NLR Family CARD Domain Containing 5 |
| TSC2 | TSC Complex Subunit 2 |
| NEB | Nebulin |
| PLP1 | Proteolipid Protein 1 |
| CYP27B1 | Cytochrome P450 Family 27 Subfamily B Member 1 |
| IL19 | Interleukin 19 |
| ENPP1 | Ectonucleotide Pyrophosphatase/Phosphodiesterase 1 |
| RO60 | Ro60, Y RNA Binding Protein |
| ANXA5 | Annexin A5 |
| C6 | Complement C6 |
| APRT | Adenine Phosphoribosyltransferase |
| CREB1 | CAMP Responsive Element Binding Protein 1 |
| SLC17A5 | Solute Carrier Family 17 Member 5 |
| TGM2 | Transglutaminase 2 |
| TERC | Telomerase RNA Component |
| GNLY | Granulysin |
| FGF2 | Fibroblast Growth Factor 2 |
| CP | Ceruloplasmin |
| RET | Ret Proto-Oncogene |
| STAT2 | Signal Transducer And Activator Of Transcription 2 |
| LILRB2 | Leukocyte Immunoglobulin Like Receptor B2 |
| GNAS | GNAS Complex Locus |
| TNFRSF25 | TNF Receptor Superfamily Member 25 |
| RB1 | RB Transcriptional Corepressor 1 |
| CCL3L1 | C-C Motif Chemokine Ligand 3 Like 1 |
| BANK1 | B Cell Scaffold Protein With Ankyrin Repeats 1 |
| PRDM16 | PR/SET Domain 16 |
| PLCG1 | Phospholipase C Gamma 1 |
| HAMP | Hepcidin Antimicrobial Peptide |
| DEFA5 | Defensin Alpha 5 |
| CD9 | CD9 Molecule |
| CLEC16A | C-Type Lectin Domain Containing 16A |
| CCRL2 | C-C Motif Chemokine Receptor Like 2 |
| SIGLEC10 | Sialic Acid Binding Ig Like Lectin 10 |
| CCL7 | C-C Motif Chemokine Ligand 7 |
| ESR1 | Estrogen Receptor 1 |
| KIR2DL3 | Killer Cell Immunoglobulin Like Receptor, Two Ig Domains And Long Cytoplasmic Tail 3 |
| UNC119 | Unc-119 Lipid Binding Chaperone |
| CRH | Corticotropin Releasing Hormone |
| F5 | Coagulation Factor V |
| CNR2 | Cannabinoid Receptor 2 |
| TRA | T Cell Receptor Alpha Locus |
| PECAM1 | Platelet And Endothelial Cell Adhesion Molecule 1 |
| FCN1 | Ficolin 1 |
| EDA | Ectodysplasin A |
| SERPINE1 | Serpin Family E Member 1 |
| KCNJ11 | Potassium Inwardly Rectifying Channel Subfamily J Member 11 |
| IRF2BP2 | Interferon Regulatory Factor 2 Binding Protein 2 |
| GAD2 | Glutamate Decarboxylase 2 |
| TEK | TEK Receptor Tyrosine Kinase |
| IKBKE | Inhibitor Of Nuclear Factor Kappa B Kinase Subunit Epsilon |
| HMOX1 | Heme Oxygenase 1 |
| GPT | Glutamic--Pyruvic Transaminase |
| MAPK10 | Mitogen-Activated Protein Kinase 10 |
| CCL24 | C-C Motif Chemokine Ligand 24 |
| MIR125A | MicroRNA 125a |
| ENO1 | Enolase 1 |
| CHAT | Choline O-Acetyltransferase |
| C4B_2 | Complement Component 4B (Chido Blood Group), Copy 2 |
| CAMK4 | Calcium/Calmodulin Dependent Protein Kinase IV |
| PDGFRB | Platelet Derived Growth Factor Receptor Beta |
| MAP2K1 | Mitogen-Activated Protein Kinase Kinase 1 |
| CTSB | Cathepsin B |
| FLT1 | Fms Related Receptor Tyrosine Kinase 1 |
| ADIPOQ | Adiponectin, C1Q And Collagen Domain Containing |
| SLC46A1 | Solute Carrier Family 46 Member 1 |
| CFD | Complement Factor D |
| SQSTM1 | Sequestosome 1 |
| KRT14 | Keratin 14 |
| SIGLEC5 | Sialic Acid Binding Ig Like Lectin 5 |
| PARN | Poly(A)-Specific Ribonuclease |
| TXK | TXK Tyrosine Kinase |
| SETD2 | SET Domain Containing 2, Histone Lysine Methyltransferase |
| MADCAM1 | Mucosal Vascular Addressin Cell Adhesion Molecule 1 |
| PRG2 | Proteoglycan 2, Pro Eosinophil Major Basic Protein |
| ADAMTS13 | ADAM Metallopeptidase With Thrombospondin Type 1 Motif 13 |
| ARVCF | ARVCF Delta Catenin Family Member |
| CASP9 | Caspase 9 |
| TNFRSF11B | TNF Receptor Superfamily Member 11b |
| TRIM22 | Tripartite Motif Containing 22 |
| NFKBIL1 | NFKB Inhibitor Like 1 |
| ERCC1 | ERCC Excision Repair 1, Endonuclease Non-Catalytic Subunit |
| RAB7A | RAB7A, Member RAS Oncogene Family |
| CCBE1 | Collagen And Calcium Binding EGF Domains 1 |
| CFHR5 | Complement Factor H Related 5 |
| MERTK | MER Proto-Oncogene, Tyrosine Kinase |
| NPM1 | Nucleophosmin 1 |
| SST | Somatostatin |
| MME | Membrane Metalloendopeptidase |
| KIR2DL1 | Killer Cell Immunoglobulin Like Receptor, Two Ig Domains And Long Cytoplasmic Tail 1 |
| MC2R | Melanocortin 2 Receptor |
| APOA1 | Apolipoprotein A1 |
| ACD | ACD Shelterin Complex Subunit And Telomerase Recruitment Factor |
| IL18BP | Interleukin 18 Binding Protein |
| LRRC8A | Leucine Rich Repeat Containing 8 VRAC Subunit A |
| PGLYRP1 | Peptidoglycan Recognition Protein 1 |
| KRT18 | Keratin 18 |
| IL31 | Interleukin 31 |
| SLC35A2 | Solute Carrier Family 35 Member A2 |
| NF1 | Neurofibromin 1 |
| XDH | Xanthine Dehydrogenase |
| MMP3 | Matrix Metallopeptidase 3 |
| DGKE | Diacylglycerol Kinase Epsilon |
| SLC25A1 | Solute Carrier Family 25 Member 1 |
| RBCK1 | RANBP2-Type And C3HC4-Type Zinc Finger Containing 1 |
| LCP2 | Lymphocyte Cytosolic Protein 2 |
| APOBEC3F | Apolipoprotein B MRNA Editing Enzyme Catalytic Subunit 3F |
| CD2 | CD2 Molecule |
| FOXJ1 | Forkhead Box J1 |
| CRKL | CRK Like Proto-Oncogene, Adaptor Protein |
| THBD | Thrombomodulin |
| CCL27 | C-C Motif Chemokine Ligand 27 |
| CDSN | Corneodesmosin |
| DGCR2 | DiGeorge Syndrome Critical Region Gene 2 |
| PMEL | Premelanosome Protein |
| HLA-F | Major Histocompatibility Complex, Class I, F |
| SOS1 | SOS Ras/Rac Guanine Nucleotide Exchange Factor 1 |
| GFI1 | Growth Factor Independent 1 Transcriptional Repressor |
| MIR223 | MicroRNA 223 |
| GZMM | Granzyme M |
| IFI27 | Interferon Alpha Inducible Protein 27 |
| CD24 | CD24 Molecule |
| CYP21A2 | Cytochrome P450 Family 21 Subfamily A Member 2 |
| KDR | Kinase Insert Domain Receptor |
| BPI | Bactericidal Permeability Increasing Protein |
| ITGAE | Integrin Subunit Alpha E |
| CCR8 | C-C Motif Chemokine Receptor 8 |
| CCL26 | C-C Motif Chemokine Ligand 26 |
| IRGQ | Immunity Related GTPase Q |
| EDAR | Ectodysplasin A Receptor |
| WT1 | WT1 Transcription Factor |
| SFTPA1 | Surfactant Protein A1 |
| POT1 | Protection Of Telomeres 1 |
| CD7 | CD7 Molecule |
| IL1RL1 | Interleukin 1 Receptor Like 1 |
| ACTB | Actin Beta |
| RREB1 | Ras Responsive Element Binding Protein 1 |
| FCER1A | Fc Fragment Of IgE Receptor Ia |
| COL17A1 | Collagen Type XVII Alpha 1 Chain |
| TNFRSF9 | TNF Receptor Superfamily Member 9 |
| TGFBR2 | Transforming Growth Factor Beta Receptor 2 |
| CAV1 | Caveolin 1 |
| PRKCQ | Protein Kinase C Theta |
| MST1 | Macrophage Stimulating 1 |
| SNRPN | Small Nuclear Ribonucleoprotein Polypeptide N |
| SEC24C | SEC24 Homolog C, COPII Coat Complex Component |
| NFE2L2 | Nuclear Factor, Erythroid 2 Like 2 |
| PVR | PVR Cell Adhesion Molecule |
| ADRB2 | Adrenoceptor Beta 2 |
| CEACAM3 | CEA Cell Adhesion Molecule 3 |
| MIR145 | MicroRNA 145 |
| KLRC2 | Killer Cell Lectin Like Receptor C2 |
| MEN1 | Menin 1 |
| NR1H4 | Nuclear Receptor Subfamily 1 Group H Member 4 |
| MAP2K2 | Mitogen-Activated Protein Kinase Kinase 2 |
| IL36G | Interleukin 36 Gamma |
| PIK3CB | Phosphatidylinositol-4,5-Bisphosphate 3-Kinase Catalytic Subunit Beta |
| ATP6V0A2 | ATPase H+ Transporting V0 Subunit A2 |
| CD160 | CD160 Molecule |
| SERPING1 | Serpin Family G Member 1 |
| CXorf21 | Chromosome X Open Reading Frame 21 |
| HCST | Hematopoietic Cell Signal Transducer |
| CFLAR | CASP8 And FADD Like Apoptosis Regulator |
| CHIT1 | Chitinase 1 |
| RAD51 | RAD51 Recombinase |
| IFIT3 | Interferon Induced Protein With Tetratricopeptide Repeats 3 |
| LILRB3 | Leukocyte Immunoglobulin Like Receptor B3 |
| CD68 | CD68 Molecule |
| DNAL1 | Dynein Axonemal Light Chain 1 |
| CCDC39 | Coiled-Coil Domain Containing 39 |
| IL27RA | Interleukin 27 Receptor Subunit Alpha |
| FANCD2 | FA Complementation Group D2 |
| CD1C | CD1c Molecule |
| SLC39A4 | Solute Carrier Family 39 Member 4 |
| CLEC4C | C-Type Lectin Domain Family 4 Member C |
| SSB | Small RNA Binding Exonuclease Protection Factor La |
| HAVCR1 | Hepatitis A Virus Cellular Receptor 1 |
| IL17RE | Interleukin 17 Receptor E |
| HSPA5 | Heat Shock Protein Family A (Hsp70) Member 5 |
| SP110 | SP110 Nuclear Body Protein |
| ALPL | Alkaline Phosphatase, Biomineralization Associated |
| FHL2 | Four And A Half LIM Domains 2 |
| PRKCE | Protein Kinase C Epsilon |
| EPO | Erythropoietin |
| LAIR1 | Leukocyte Associated Immunoglobulin Like Receptor 1 |
| IDH2 | Isocitrate Dehydrogenase (NADP(+)) 2 |
| RSAD2 | Radical S-Adenosyl Methionine Domain Containing 2 |
| DOCK2 | Dedicator Of Cytokinesis 2 |
| MCL1 | MCL1 Apoptosis Regulator, BCL2 Family Member |
| SMC1A | Structural Maintenance Of Chromosomes 1A |
| IGHV4-38-2 | Immunoglobulin Heavy Variable 4-38-2 |
| TREM1 | Triggering Receptor Expressed On Myeloid Cells 1 |
| LILRB4 | Leukocyte Immunoglobulin Like Receptor B4 |
| GGT1 | Gamma-Glutamyltransferase 1 |
| ELN | Elastin |
| TCF4 | Transcription Factor 4 |
| TPP2 | Tripeptidyl Peptidase 2 |
| CCL13 | C-C Motif Chemokine Ligand 13 |
| AHR | Aryl Hydrocarbon Receptor |
| CFHR1 | Complement Factor H Related 1 |
| PCCA | Propionyl-CoA Carboxylase Subunit Alpha |
| MIR132 | MicroRNA 132 |
| CFHR3 | Complement Factor H Related 3 |
| MIR184 | MicroRNA 184 |
| IRF2 | Interferon Regulatory Factor 2 |
| DEFB103B | Defensin Beta 103B |
| CD33 | CD33 Molecule |
| MAP3K14 | Mitogen-Activated Protein Kinase Kinase Kinase 14 |
| LYZ | Lysozyme |
| PI3 | Peptidase Inhibitor 3 |
| ERCC2 | ERCC Excision Repair 2, TFIIH Core Complex Helicase Subunit |
| CFHR2 | Complement Factor H Related 2 |
| CFHR4 | Complement Factor H Related 4 |
| CD48 | CD48 Molecule |
| PRDX1 | Peroxiredoxin 1 |
| TTN | Titin |
| PTH | Parathyroid Hormone |
| TMC6 | Transmembrane Channel Like 6 |
| CXCL5 | C-X-C Motif Chemokine Ligand 5 |
| RETN | Resistin |
| STUB1 | STIP1 Homology And U-Box Containing Protein 1 |
| MIR134 | MicroRNA 134 |
| CHRNE | Cholinergic Receptor Nicotinic Epsilon Subunit |
| ALG12 | ALG12 Alpha-1,6-Mannosyltransferase |
| CD1B | CD1b Molecule |
| ZBTB24 | Zinc Finger And BTB Domain Containing 24 |
| RBP4 | Retinol Binding Protein 4 |
| IGF2R | Insulin Like Growth Factor 2 Receptor |
| CCN2 | Cellular Communication Network Factor 2 |
| CEACAM1 | CEA Cell Adhesion Molecule 1 |
| CHD1 | Chromodomain Helicase DNA Binding Protein 1 |
| CEBPB | CCAAT Enhancer Binding Protein Beta |
| S100A7 | S100 Calcium Binding Protein A7 |
| APOB | Apolipoprotein B |
| APP | Amyloid Beta Precursor Protein |
| NKX2-5 | NK2 Homeobox 5 |
| SOD1 | Superoxide Dismutase 1 |
| CSN1S1 | Casein Alpha S1 |
| PARP1 | Poly(ADP-Ribose) Polymerase 1 |
| KRT17 | Keratin 17 |
| INSR | Insulin Receptor |
| PTGDR2 | Prostaglandin D2 Receptor 2 |
| TAT | Tyrosine Aminotransferase |
| RPSA | Ribosomal Protein SA |
| RPS14 | Ribosomal Protein S14 |
| CIB1 | Calcium And Integrin Binding 1 |
| BDNF | Brain Derived Neurotrophic Factor |
| PXK | PX Domain Containing Serine/Threonine Kinase Like |
| CENPB | Centromere Protein B |
| GYPC | Glycophorin C (Gerbich Blood Group) |
| APTX | Aprataxin |
| DEAF1 | DEAF1 Transcription Factor |
| PRKG1 | Protein Kinase CGMP-Dependent 1 |
| FGF8 | Fibroblast Growth Factor 8 |
| ASAH1 | N-Acylsphingosine Amidohydrolase 1 |
| TKT | Transketolase |
| SELPLG | Selectin P Ligand |
| GJC2 | Gap Junction Protein Gamma 2 |
| NFATC1 | Nuclear Factor Of Activated T Cells 1 |
| MUC5B | Mucin 5B, Oligomeric Mucus/Gel-Forming |
| RIPK3 | Receptor Interacting Serine/Threonine Kinase 3 |
| AQP4 | Aquaporin 4 |
| IGF1R | Insulin Like Growth Factor 1 Receptor |
| KLHDC8B | Kelch Domain Containing 8B |
| AGPAT2 | 1-Acylglycerol-3-Phosphate O-Acyltransferase 2 |
| C3AR1 | Complement C3a Receptor 1 |
| ANXA2 | Annexin A2 |
| LTB | Lymphotoxin Beta |
| UBB | Ubiquitin B |
| ENTPD1 | Ectonucleoside Triphosphate Diphosphohydrolase 1 |
| HGF | Hepatocyte Growth Factor |
| VTCN1 | V-Set Domain Containing T Cell Activation Inhibitor 1 |
| TNPO3 | Transportin 3 |
| RPL11 | Ribosomal Protein L11 |
| ALG1 | ALG1 Chitobiosyldiphosphodolichol Beta-Mannosyltransferase |
| TGFB3 | Transforming Growth Factor Beta 3 |
| BUB1B | BUB1 Mitotic Checkpoint Serine/Threonine Kinase B |
| CLEC4A | C-Type Lectin Domain Family 4 Member A |
| PTPN6 | Protein Tyrosine Phosphatase Non-Receptor Type 6 |
| CD226 | CD226 Molecule |
| CHI3L1 | Chitinase 3 Like 1 |
| OTC | Ornithine Carbamoyltransferase |
| HK1 | Hexokinase 1 |
| CSF2RA | Colony Stimulating Factor 2 Receptor Subunit Alpha |
| APC | APC Regulator Of WNT Signaling Pathway |
| IL36A | Interleukin 36 Alpha |
| ALOX15 | Arachidonate 15-Lipoxygenase |
| KIR2DS2 | Killer Cell Immunoglobulin Like Receptor, Two Ig Domains And Short Cytoplasmic Tail 2 |
| DMBT1 | Deleted In Malignant Brain Tumors 1 |
| KIR2DS1 | Killer Cell Immunoglobulin Like Receptor, Two Ig Domains And Short Cytoplasmic Tail 1 |
| FLII | FLII Actin Remodeling Protein |
| DGCR8 | DGCR8 Microprocessor Complex Subunit |
| SRF | Serum Response Factor |
| TAC1 | Tachykinin Precursor 1 |
| GIMAP5 | GTPase, IMAP Family Member 5 |
| CD207 | CD207 Molecule |
| CALCA | Calcitonin Related Polypeptide Alpha |
| VTN | Vitronectin |
| CISH | Cytokine Inducible SH2 Containing Protein |
| POLG | DNA Polymerase Gamma, Catalytic Subunit |
| HYDIN | HYDIN Axonemal Central Pair Apparatus Protein |
| GBP5 | Guanylate Binding Protein 5 |
| NCR1 | Natural Cytotoxicity Triggering Receptor 1 |
| CDK9 | Cyclin Dependent Kinase 9 |
| SDHB | Succinate Dehydrogenase Complex Iron Sulfur Subunit B |
| RBP3 | Retinol Binding Protein 3 |
| IFIT1 | Interferon Induced Protein With Tetratricopeptide Repeats 1 |
| MUC5AC | Mucin 5AC, Oligomeric Mucus/Gel-Forming |
| BCL3 | BCL3 Transcription Coactivator |
| PMS2 | PMS1 Homolog 2, Mismatch Repair System Component |
| INPPL1 | Inositol Polyphosphate Phosphatase Like 1 |
| TFE3 | Transcription Factor Binding To IGHM Enhancer 3 |
| PRODH | Proline Dehydrogenase 1 |
| RPS27A | Ribosomal Protein S27a |
| IFITM3 | Interferon Induced Transmembrane Protein 3 |
| ITGB4 | Integrin Subunit Beta 4 |
| SERPINB1 | Serpin Family B Member 1 |
| CEBPE | CCAAT Enhancer Binding Protein Epsilon |
| MIR30E | MicroRNA 30e |
| KRT10 | Keratin 10 |
| MANBA | Mannosidase Beta |
| FANCC | FA Complementation Group C |
| SMAD4 | SMAD Family Member 4 |
| GHR | Growth Hormone Receptor |
| RPL35A | Ribosomal Protein L35a |
| ITGAV | Integrin Subunit Alpha V |
| IFNL1 | Interferon Lambda 1 |
| FCAR | Fc Fragment Of IgA Receptor |
| JAZF1 | JAZF Zinc Finger 1 |
| TIMM8A | Translocase Of Inner Mitochondrial Membrane 8A |
| MIR326 | MicroRNA 326 |
| FGF7 | Fibroblast Growth Factor 7 |
| CBLB | Cbl Proto-Oncogene B |
| PRSS2 | Serine Protease 2 |
| IL15RA | Interleukin 15 Receptor Subunit Alpha |
| POLE | DNA Polymerase Epsilon, Catalytic Subunit |
| RPS24 | Ribosomal Protein S24 |
| BIRC5 | Baculoviral IAP Repeat Containing 5 |
| TJP1 | Tight Junction Protein 1 |
| MSH6 | MutS Homolog 6 |
| ATR | ATR Serine/Threonine Kinase |
| USB1 | U6 SnRNA Biogenesis Phosphodiesterase 1 |
| CD300LF | CD300 Molecule Like Family Member F |
| SLEB3 | Systemic Lupus Erythematosus Susceptibility 3 |
| SLEB4 | Systemic Lupus Erythematosus, Susceptibility To, 4 |
| SLEB15 | Systemic Lupus Erthematosus, Susceptibility To, 15 |
| SLEB5 | Systemic Lupus Erythematosus, Susceptibility To, 5 |
| SLEB7 | Systemic Lupus Erythematosus, Susceptibility To, 7 |
| SLEB8 | Systemic Lupus Erythematosus, Susceptibility To, 8 |
| SLEB12 | Systemic Lupus Erythematosus, Susceptibility To, 12 |
| SLEB13 | Systemic Lupus Erythematosus, Susceptibility To, 13 |
| SLEB14 | Systemic Lupus Erythematosus, Susceptiblity To, 14 |
| MLH1 | MutL Homolog 1 |
| TUSC3 | Tumor Suppressor Candidate 3 |
| NSD1 | Nuclear Receptor Binding SET Domain Protein 1 |
| TGFBR1 | Transforming Growth Factor Beta Receptor 1 |
| SLC35C1 | Solute Carrier Family 35 Member C1 |
| SEMA3E | Semaphorin 3E |
| ACP1 | Acid Phosphatase 1 |
| NLRC3 | NLR Family CARD Domain Containing 3 |
| KLRG1 | Killer Cell Lectin Like Receptor G1 |
| PLA2G2A | Phospholipase A2 Group IIA |
| LPP | LIM Domain Containing Preferred Translocation Partner In Lipoma |
| PIK3R2 | Phosphoinositide-3-Kinase Regulatory Subunit 2 |
| AKT3 | AKT Serine/Threonine Kinase 3 |
| IRF9 | Interferon Regulatory Factor 9 |
| SAG | S-Antigen Visual Arrestin |
| CCR10 | C-C Motif Chemokine Receptor 10 |
| LILRA2 | Leukocyte Immunoglobulin Like Receptor A2 |
| PEPD | Peptidase D |
| MAP3K8 | Mitogen-Activated Protein Kinase Kinase Kinase 8 |
| SP100 | SP100 Nuclear Antigen |
| ANPEP | Alanyl Aminopeptidase, Membrane |
| SERPINC1 | Serpin Family C Member 1 |
| CSTA | Cystatin A |
| CTSA | Cathepsin A |
| FHL1 | Four And A Half LIM Domains 1 |
| SOS2 | SOS Ras/Rho Guanine Nucleotide Exchange Factor 2 |
| JMJD1C | Jumonji Domain Containing 1C |
| TBX2 | T-Box Transcription Factor 2 |
| MIR15A | MicroRNA 15a |
| CCL8 | C-C Motif Chemokine Ligand 8 |
| PAX5 | Paired Box 5 |
| MIR30A | MicroRNA 30a |
| CYCS | Cytochrome C, Somatic |
| STK4 | Serine/Threonine Kinase 4 |
| GPR35 | G Protein-Coupled Receptor 35 |
| PPARA | Peroxisome Proliferator Activated Receptor Alpha |
| CD74 | CD74 Molecule |
| C5AR2 | Complement Component 5a Receptor 2 |
| IRAK3 | Interleukin 1 Receptor Associated Kinase 3 |
| ADORA2A | Adenosine A2a Receptor |
| CD200 | CD200 Molecule |
| GSN | Gelsolin |
| FTH1 | Ferritin Heavy Chain 1 |
| IL26 | Interleukin 26 |
| TPRKB | TP53RK Binding Protein |
| ISG20 | Interferon Stimulated Exonuclease Gene 20 |
| FMR1 | FMRP Translational Regulator 1 |
| NPHS1 | NPHS1 Adhesion Molecule, Nephrin |
| ATN1 | Atrophin 1 |
| ITGA6 | Integrin Subunit Alpha 6 |
| LTBR | Lymphotoxin Beta Receptor |
| SERPINB3 | Serpin Family B Member 3 |
| STAR | Steroidogenic Acute Regulatory Protein |
| COG7 | Component Of Oligomeric Golgi Complex 7 |
| IL20 | Interleukin 20 |
| SCGB1A1 | Secretoglobin Family 1A Member 1 |
| TRIM25 | Tripartite Motif Containing 25 |
| PCNA | Proliferating Cell Nuclear Antigen |
| CHRM3 | Cholinergic Receptor Muscarinic 3 |
| RBM8A | RNA Binding Motif Protein 8A |
| EIF2AK3 | Eukaryotic Translation Initiation Factor 2 Alpha Kinase 3 |
| SIGIRR | Single Ig And TIR Domain Containing |
| PDCD1LG2 | Programmed Cell Death 1 Ligand 2 |
| KRT5 | Keratin 5 |
| TBXAS1 | Thromboxane A Synthase 1 |
| ACTN4 | Actinin Alpha 4 |
| PLEC | Plectin |
| GSK3B | Glycogen Synthase Kinase 3 Beta |
| F9 | Coagulation Factor IX |
| HSP90B1 | Heat Shock Protein 90 Beta Family Member 1 |
| GPC3 | Glypican 3 |
| SHH | Sonic Hedgehog Signaling Molecule |
| ERBB3 | Erb-B2 Receptor Tyrosine Kinase 3 |
| ESS2 | Ess-2 Splicing Factor Homolog |
| TNFSF8 | TNF Superfamily Member 8 |
| ACOD1 | Aconitate Decarboxylase 1 |
| TGM1 | Transglutaminase 1 |
| ADAMTS3 | ADAM Metallopeptidase With Thrombospondin Type 1 Motif 3 |
| CCL1 | C-C Motif Chemokine Ligand 1 |
| PIGR | Polymeric Immunoglobulin Receptor |
| FLNA | Filamin A |
| PSIP1 | PC4 And SFRS1 Interacting Protein 1 |
| KDM6A | Lysine Demethylase 6A |
| DHFR | Dihydrofolate Reductase |
| LPL | Lipoprotein Lipase |
| TRAF1 | TNF Receptor Associated Factor 1 |
| FAT4 | FAT Atypical Cadherin 4 |
| CAVIN1 | Caveolae Associated Protein 1 |
| MYH3 | Myosin Heavy Chain 3 |
| NAGLU | N-Acetyl-Alpha-Glucosaminidase |
| DNAAF5 | Dynein Axonemal Assembly Factor 5 |
| EDNRB | Endothelin Receptor Type B |
| CD52 | CD52 Molecule |
| LGALS9 | Galectin 9 |
| ULBP1 | UL16 Binding Protein 1 |
| RUNX1 | RUNX Family Transcription Factor 1 |
| FGFR2 | Fibroblast Growth Factor Receptor 2 |
| RPS6KB1 | Ribosomal Protein S6 Kinase B1 |
| NOP10 | NOP10 Ribonucleoprotein |
| TRIM29 | Tripartite Motif Containing 29 |
| DMD | Dystrophin |
| NLRP10 | NLR Family Pyrin Domain Containing 10 |
| PLAUR | Plasminogen Activator, Urokinase Receptor |
| CCND3 | Cyclin D3 |
| SEC61A1 | SEC61 Translocon Subunit Alpha 1 |
| APOL1 | Apolipoprotein L1 |
| LAMP2 | Lysosomal Associated Membrane Protein 2 |
| PCCB | Propionyl-CoA Carboxylase Subunit Beta |
| DDX3X | DEAD-Box Helicase 3 X-Linked |
| IL5RA | Interleukin 5 Receptor Subunit Alpha |
| RAPSN | Receptor Associated Protein Of The Synapse |
| ITPR3 | Inositol 1,4,5-Trisphosphate Receptor Type 3 |
| GLUL | Glutamate-Ammonia Ligase |
| MMP12 | Matrix Metallopeptidase 12 |
| NBEAL2 | Neurobeachin Like 2 |
| GAA | Glucosidase Alpha, Acid |
| PLG | Plasminogen |
| ADNP | Activity Dependent Neuroprotector Homeobox |
| GNRH1 | Gonadotropin Releasing Hormone 1 |
| PKP1 | Plakophilin 1 |
| NCR3 | Natural Cytotoxicity Triggering Receptor 3 |
| FGB | Fibrinogen Beta Chain |
| SPAG1 | Sperm Associated Antigen 1 |
| LAMA3 | Laminin Subunit Alpha 3 |
| CXCL16 | C-X-C Motif Chemokine Ligand 16 |
| WRAP53 | WD Repeat Containing Antisense To TP53 |
| SPTA1 | Spectrin Alpha, Erythrocytic 1 |
| HPRT1 | Hypoxanthine Phosphoribosyltransferase 1 |
| FGG | Fibrinogen Gamma Chain |
| CASP4 | Caspase 4 |
| GBE1 | 1,4-Alpha-Glucan Branching Enzyme 1 |
| CTSE | Cathepsin E |
| LAMA2 | Laminin Subunit Alpha 2 |
| ATP7A | ATPase Copper Transporting Alpha |
| PGLYRP3 | Peptidoglycan Recognition Protein 3 |
| MYH9 | Myosin Heavy Chain 9 |
| TRIP13 | Thyroid Hormone Receptor Interactor 13 |
| EZR | Ezrin |
| ABCB11 | ATP Binding Cassette Subfamily B Member 11 |
| PPP3CA | Protein Phosphatase 3 Catalytic Subunit Alpha |
| RUNX2 | RUNX Family Transcription Factor 2 |
| RYR1 | Ryanodine Receptor 1 |
| ANGPT1 | Angiopoietin 1 |
| OPRM1 | Opioid Receptor Mu 1 |
| ERCC5 | ERCC Excision Repair 5, Endonuclease |
| MAPK9 | Mitogen-Activated Protein Kinase 9 |
| CYP3A4 | Cytochrome P450 Family 3 Subfamily A Member 4 |
| MT-ND1 | Mitochondrially Encoded NADH:Ubiquinone Oxidoreductase Core Subunit 1 |
| TSC1 | TSC Complex Subunit 1 |
| S1PR1 | Sphingosine-1-Phosphate Receptor 1 |
| LETM1 | Leucine Zipper And EF-Hand Containing Transmembrane Protein 1 |
| U2AF1 | U2 Small Nuclear RNA Auxiliary Factor 1 |
| DLAT | Dihydrolipoamide S-Acetyltransferase |
| DSG3 | Desmoglein 3 |
| DNAI1 | Dynein Axonemal Intermediate Chain 1 |
| PROP1 | PROP Paired-Like Homeobox 1 |
| PRSS3 | Serine Protease 3 |
| ETV6 | ETS Variant Transcription Factor 6 |
| CDCA7 | Cell Division Cycle Associated 7 |
| ATG16L1 | Autophagy Related 16 Like 1 |
| KIR2DS5 | Killer Cell Immunoglobulin Like Receptor, Two Ig Domains And Short Cytoplasmic Tail 5 |
| SMPD1 | Sphingomyelin Phosphodiesterase 1 |
| SHC1 | SHC Adaptor Protein 1 |
| RAP1B | RAP1B, Member Of RAS Oncogene Family |
| TRPS1 | Transcriptional Repressor GATA Binding 1 |
| BGLAP | Bone Gamma-Carboxyglutamate Protein |
| ACTR2 | Actin Related Protein 2 |
| TMC8 | Transmembrane Channel Like 8 |
| NOTCH2 | Notch Receptor 2 |
| RANBP2 | RAN Binding Protein 2 |
| CLDN1 | Claudin 1 |
| BMP4 | Bone Morphogenetic Protein 4 |
| CD47 | CD47 Molecule |
| KIR3DS1 | Killer Cell Immunoglobulin Like Receptor, Three Ig Domains And Short Cytoplasmic Tail 1 |
| CST3 | Cystatin C |
| UMOD | Uromodulin |
| HLA-DQB2 | Major Histocompatibility Complex, Class II, DQ Beta 2 |
| PON1 | Paraoxonase 1 |
| IL17D | Interleukin 17D |
| VIM | Vimentin |
| CTSD | Cathepsin D |
| IL22RA1 | Interleukin 22 Receptor Subunit Alpha 1 |
| BCL2L11 | BCL2 Like 11 |
| NHP2 | NHP2 Ribonucleoprotein |
| TARBP2 | TARBP2 Subunit Of RISC Loading Complex |
| MAF | MAF BZIP Transcription Factor |
| MT-ND4 | Mitochondrially Encoded NADH:Ubiquinone Oxidoreductase Core Subunit 4 |
| CXCR6 | C-X-C Motif Chemokine Receptor 6 |
| NR1H3 | Nuclear Receptor Subfamily 1 Group H Member 3 |
| GAD1 | Glutamate Decarboxylase 1 |
| PTPRN | Protein Tyrosine Phosphatase Receptor Type N |
| HNRNPA2B1 | Heterogeneous Nuclear Ribonucleoprotein A2/B1 |
| COL1A1 | Collagen Type I Alpha 1 Chain |
| FSCN1 | Fascin Actin-Bundling Protein 1 |
| TIA1 | TIA1 Cytotoxic Granule Associated RNA Binding Protein |
| COL4A3 | Collagen Type IV Alpha 3 Chain |
| FCRL4 | Fc Receptor Like 4 |
| NCR2 | Natural Cytotoxicity Triggering Receptor 2 |
| PPBP | Pro-Platelet Basic Protein |
| GFAP | Glial Fibrillary Acidic Protein |
| GAPDH | Glyceraldehyde-3-Phosphate Dehydrogenase |
| CTAG1B | Cancer/Testis Antigen 1B |
| LTA4H | Leukotriene A4 Hydrolase |
| ZNF341 | Zinc Finger Protein 341 |
| PAK2 | P21 (RAC1) Activated Kinase 2 |
| TRAIP | TRAF Interacting Protein |
| MT-ND6 | Mitochondrially Encoded NADH:Ubiquinone Oxidoreductase Core Subunit 6 |
| GALE | UDP-Galactose-4-Epimerase |
| AGK | Acylglycerol Kinase |
| ULBP3 | UL16 Binding Protein 3 |
| PDGFB | Platelet Derived Growth Factor Subunit B |
| STX1A | Syntaxin 1A |
| WASF1 | WASP Family Member 1 |
| AR | Androgen Receptor |
| CDKN2B | Cyclin Dependent Kinase Inhibitor 2B |
| DNAH11 | Dynein Axonemal Heavy Chain 11 |
| DNAH5 | Dynein Axonemal Heavy Chain 5 |
| DRC1 | Dynein Regulatory Complex Subunit 1 |
| MYLK | Myosin Light Chain Kinase |
| ACTR3 | Actin Related Protein 3 |
| TRAF2 | TNF Receptor Associated Factor 2 |
| CELIAC13 | Celiac Disease, Susceptibility To, 13 |
| CD6 | CD6 Molecule |
| APCS | Amyloid P Component, Serum |
| GAS1 | Growth Arrest Specific 1 |
| TBCE | Tubulin Folding Cofactor E |
| ZEB1 | Zinc Finger E-Box Binding Homeobox 1 |
| GP9 | Glycoprotein IX Platelet |
| EXTL3 | Exostosin Like Glycosyltransferase 3 |
| KIR2DL4 | Killer Cell Immunoglobulin Like Receptor, Two Ig Domains And Long Cytoplasmic Tail 4 |
| CHGA | Chromogranin A |
| IFITM1 | Interferon Induced Transmembrane Protein 1 |
| SFTPA2 | Surfactant Protein A2 |
| CCNT1 | Cyclin T1 |
| SPTAN1 | Spectrin Alpha, Non-Erythrocytic 1 |
| IRF6 | Interferon Regulatory Factor 6 |
| CTTN | Cortactin |
| C1QBP | Complement C1q Binding Protein |
| MIR126 | MicroRNA 126 |
| SETX | Senataxin |
| LMNA | Lamin A/C |
| MIR181A2 | MicroRNA 181a-2 |
| MIR181A1 | MicroRNA 181a-1 |
| IDUA | Alpha-L-Iduronidase |
| EDARADD | EDAR Associated Death Domain |
| SNAP25 | Synaptosome Associated Protein 25 |
| MUC7 | Mucin 7, Secreted |
| ATRX | ATRX Chromatin Remodeler |
| DCTN4 | Dynactin Subunit 4 |
| TGIF1 | TGFB Induced Factor Homeobox 1 |
| RARA | Retinoic Acid Receptor Alpha |
| TRIP11 | Thyroid Hormone Receptor Interactor 11 |
| F13A1 | Coagulation Factor XIII A Chain |
| IKZF3 | IKAROS Family Zinc Finger 3 |
| GAST | Gastrin |
| SIRT1 | Sirtuin 1 |
| SDC1 | Syndecan 1 |
| DCT | Dopachrome Tautomerase |
| KIR2DL2 | Killer Cell Immunoglobulin Like Receptor, Two Ig Domains And Long Cytoplasmic Tail 2 |
| PGM1 | Phosphoglucomutase 1 |
| ATF2 | Activating Transcription Factor 2 |
| NLRX1 | NLR Family Member X1 |
| RNU4ATAC | RNA, U4atac Small Nuclear (U12-Dependent Splicing) |
| NCK1 | NCK Adaptor Protein 1 |
| LGALS3BP | Galectin 3 Binding Protein |
| NPC2 | NPC Intracellular Cholesterol Transporter 2 |
| DGCR6 | DiGeorge Syndrome Critical Region Gene 6 |
| SPIB | Spi-B Transcription Factor |
| MT-TW | Mitochondrially Encoded TRNA-Trp (UGA/G) |
| TH | Tyrosine Hydroxylase |
| PGLYRP4 | Peptidoglycan Recognition Protein 4 |
| GRIN2A | Glutamate Ionotropic Receptor NMDA Type Subunit 2A |
| IL24 | Interleukin 24 |
| PRKCB | Protein Kinase C Beta |
| CXCL6 | C-X-C Motif Chemokine Ligand 6 |
| MUC2 | Mucin 2, Oligomeric Mucus/Gel-Forming |
| GHRL | Ghrelin And Obestatin Prepropeptide |
| VPS45 | Vacuolar Protein Sorting 45 Homolog |
| SNRNP70 | Small Nuclear Ribonucleoprotein U1 Subunit 70 |
| LALBA | Lactalbumin Alpha |
| EZH2 | Enhancer Of Zeste 2 Polycomb Repressive Complex 2 Subunit |
| PTGS1 | Prostaglandin-Endoperoxide Synthase 1 |
| HGSNAT | Heparan-Alpha-Glucosaminide N-Acetyltransferase |
| ERAP2 | Endoplasmic Reticulum Aminopeptidase 2 |
| CCK | Cholecystokinin |
| DNAAF2 | Dynein Axonemal Assembly Factor 2 |
| HES7 | Hes Family BHLH Transcription Factor 7 |
| FCER1G | Fc Fragment Of IgE Receptor Ig |
| LMO2 | LIM Domain Only 2 |
| COG6 | Component Of Oligomeric Golgi Complex 6 |
| LAMP1 | Lysosomal Associated Membrane Protein 1 |
| RNASE7 | Ribonuclease A Family Member 7 |
| NEAT1 | Nuclear Paraspeckle Assembly Transcript 1 |
| CLCN7 | Chloride Voltage-Gated Channel 7 |
| CRYAB | Crystallin Alpha B |
| KAT5 | Lysine Acetyltransferase 5 |
| CD63 | CD63 Molecule |
| AGTR1 | Angiotensin II Receptor Type 1 |
| KIR2DS4 | Killer Cell Immunoglobulin Like Receptor, Two Ig Domains And Short Cytoplasmic Tail 4 |
| BCKDHA | Branched Chain Keto Acid Dehydrogenase E1 Subunit Alpha |
| TOR1A | Torsin Family 1 Member A |
| SMARCB1 | SWI/SNF Related, Matrix Associated, Actin Dependent Regulator Of Chromatin, Subfamily B, Member 1 |
| ZBTB16 | Zinc Finger And BTB Domain Containing 16 |
| OAS3 | 2'-5'-Oligoadenylate Synthetase 3 |
| CNTF | Ciliary Neurotrophic Factor |
| FBXL4 | F-Box And Leucine Rich Repeat Protein 4 |
| WRN | WRN RecQ Like Helicase |
| NPY | Neuropeptide Y |
| APOBEC3A | Apolipoprotein B MRNA Editing Enzyme Catalytic Subunit 3A |
| SNCA | Synuclein Alpha |
| COLEC11 | Collectin Subfamily Member 11 |
| PIM1 | Pim-1 Proto-Oncogene, Serine/Threonine Kinase |
| SAA1 | Serum Amyloid A1 |
| BPIFA1 | BPI Fold Containing Family A Member 1 |
| EOMES | Eomesodermin |
| FIP1L1 | Factor Interacting With PAPOLA And CPSF1 |
| FFAR2 | Free Fatty Acid Receptor 2 |
| G6PC3 | Glucose-6-Phosphatase Catalytic Subunit 3 |
| IGF2 | Insulin Like Growth Factor 2 |
| ITGA3 | Integrin Subunit Alpha 3 |
| ALDOA | Aldolase, Fructose-Bisphosphate A |
| HBG2 | Hemoglobin Subunit Gamma 2 |
| CLDN2 | Claudin 2 |
| CABIN1 | Calcineurin Binding Protein 1 |
| TNFSF14 | TNF Superfamily Member 14 |
| NR1H2 | Nuclear Receptor Subfamily 1 Group H Member 2 |
| TAB2 | TGF-Beta Activated Kinase 1 (MAP3K7) Binding Protein 2 |
| CNR1 | Cannabinoid Receptor 1 |
| DST | Dystonin |
| TSG101 | Tumor Susceptibility 101 |
| CLEC5A | C-Type Lectin Domain Containing 5A |
| ARRB1 | Arrestin Beta 1 |
| SNRPD1 | Small Nuclear Ribonucleoprotein D1 Polypeptide |
| ABCA1 | ATP Binding Cassette Subfamily A Member 1 |
| PLA2G7 | Phospholipase A2 Group VII |
| KLRC4 | Killer Cell Lectin Like Receptor C4 |
| RNF125 | Ring Finger Protein 125 |
| MMP8 | Matrix Metallopeptidase 8 |
| GNB1 | G Protein Subunit Beta 1 |
| NECTIN1 | Nectin Cell Adhesion Molecule 1 |
| BSCL2 | BSCL2 Lipid Droplet Biogenesis Associated, Seipin |
| COLQ | Collagen Like Tail Subunit Of Asymmetric Acetylcholinesterase |
| ALG13 | ALG13 UDP-N-Acetylglucosaminyltransferase Subunit |
| FBXW11 | F-Box And WD Repeat Domain Containing 11 |
| IGHV3-23 | Immunoglobulin Heavy Variable 3-23 |
| IL1R2 | Interleukin 1 Receptor Type 2 |
| SEMA4A | Semaphorin 4A |
| COL5A1 | Collagen Type V Alpha 1 Chain |
| RNF135 | Ring Finger Protein 135 |
| ACTA1 | Actin Alpha 1, Skeletal Muscle |
| RSPH9 | Radial Spoke Head Component 9 |
| BUB1 | BUB1 Mitotic Checkpoint Serine/Threonine Kinase |
| SOX2 | SRY-Box Transcription Factor 2 |
| JUNB | JunB Proto-Oncogene, AP-1 Transcription Factor Subunit |
| NECTIN2 | Nectin Cell Adhesion Molecule 2 |
| AOC1 | Amine Oxidase Copper Containing 1 |
| GPR15 | G Protein-Coupled Receptor 15 |
| TNFAIP6 | TNF Alpha Induced Protein 6 |
| CELIAC2 | Celiac Disease 2 |
| CELIAC5 | Celiac Disease, Susceptibility To, 5 |
| CELIAC10 | Celiac Disease, Susceptibility To, 10 |
| CELIAC11 | Celiac Disease, Susceptibility To, 11 |
| CELIAC12 | Celiac Disease, Susceptibility To, 12 |
| CELIAC6 | Celiac Disease, Susceptibility To, 6 |
| CELIAC7 | Celiac Disease, Susceptibility To, 7 |
| CELIAC8 | Celiac Disease, Susceptibility To, 8 |
| CELIAC9 | Celiac Disease, Susceptibility To, 9 |
| FBN1 | Fibrillin 1 |
| CLEC1A | C-Type Lectin Domain Family 1 Member A |
| MAN2A1 | Mannosidase Alpha Class 2A Member 1 |
| GNAQ | G Protein Subunit Alpha Q |
| ERVK-6 | Endogenous Retrovirus Group K Member 6, Envelope |
| RSPH1 | Radial Spoke Head Component 1 |
| RSPH4A | Radial Spoke Head Component 4A |
| DNAAF1 | Dynein Axonemal Assembly Factor 1 |
| HDAC1 | Histone Deacetylase 1 |
| TNFRSF10B | TNF Receptor Superfamily Member 10b |
| TP63 | Tumor Protein P63 |
| SOD2 | Superoxide Dismutase 2 |
| PSMB5 | Proteasome 20S Subunit Beta 5 |
| TNFAIP8L2 | TNF Alpha Induced Protein 8 Like 2 |
| IFNG-AS1 | IFNG Antisense RNA 1 |
| HDAC9 | Histone Deacetylase 9 |
| GP6 | Glycoprotein VI Platelet |
| FURIN | Furin, Paired Basic Amino Acid Cleaving Enzyme |
| IGES | Immunoglobulin E Concentration, Serum |
| RAD50 | RAD50 Double Strand Break Repair Protein |
| FGR | FGR Proto-Oncogene, Src Family Tyrosine Kinase |
| PDE4A | Phosphodiesterase 4A |
| LAMTOR2 | Late Endosomal/Lysosomal Adaptor, MAPK And MTOR Activator 2 |
| DOK7 | Docking Protein 7 |
| SPI1 | Spi-1 Proto-Oncogene |
| COL3A1 | Collagen Type III Alpha 1 Chain |
| TCIM | Transcriptional And Immune Response Regulator |
| FANCI | FA Complementation Group I |
| GHRH | Growth Hormone Releasing Hormone |
| DNAI2 | Dynein Axonemal Intermediate Chain 2 |
| CCDC40 | Coiled-Coil Domain Containing 40 |
| CCDC103 | Coiled-Coil Domain Containing 103 |
| DNAAF3 | Dynein Axonemal Assembly Factor 3 |
| PDX1 | Pancreatic And Duodenal Homeobox 1 |
| SKP2 | S-Phase Kinase Associated Protein 2 |
| RNF168 | Ring Finger Protein 168 |
| SLC25A13 | Solute Carrier Family 25 Member 13 |
| PSMA7 | Proteasome 20S Subunit Alpha 7 |
| TCN2 | Transcobalamin 2 |
| PIK3R5 | Phosphoinositide-3-Kinase Regulatory Subunit 5 |
| SMARCA2 | SWI/SNF Related, Matrix Associated, Actin Dependent Regulator Of Chromatin, Subfamily A, Member 2 |
| ACVRL1 | Activin A Receptor Like Type 1 |
| PRKACA | Protein Kinase CAMP-Activated Catalytic Subunit Alpha |
| MAPK11 | Mitogen-Activated Protein Kinase 11 |
| DUOX2 | Dual Oxidase 2 |
| MARCO | Macrophage Receptor With Collagenous Structure |
| LIMK1 | LIM Domain Kinase 1 |
| TRIM14 | Tripartite Motif Containing 14 |
| CD8B | CD8b Molecule |
| EDN1 | Endothelin 1 |
| KARS1 | Lysyl-TRNA Synthetase 1 |
| ATP7B | ATPase Copper Transporting Beta |
| FUT2 | Fucosyltransferase 2 |
| BAD | BCL2 Associated Agonist Of Cell Death |
| COL1A2 | Collagen Type I Alpha 2 Chain |
| SH2D2A | SH2 Domain Containing 2A |
| TANK | TRAF Family Member Associated NFKB Activator |
| CST7 | Cystatin F |
| SPINK1 | Serine Peptidase Inhibitor Kazal Type 1 |
| MYH6 | Myosin Heavy Chain 6 |
| MRC1 | Mannose Receptor C-Type 1 |
| LILRA4 | Leukocyte Immunoglobulin Like Receptor A4 |
| PSAP | Prosaposin |
| SIGLEC1 | Sialic Acid Binding Ig Like Lectin 1 |
| ALOX12 | Arachidonate 12-Lipoxygenase, 12S Type |
| OCRL | OCRL Inositol Polyphosphate-5-Phosphatase |
| DES | Desmin |
| CCNO | Cyclin O |
| RASGRP3 | RAS Guanyl Releasing Protein 3 |
| RAI1 | Retinoic Acid Induced 1 |
| PNPLA1 | Patatin Like Phospholipase Domain Containing 1 |
| MT-ND5 | Mitochondrially Encoded NADH:Ubiquinone Oxidoreductase Core Subunit 5 |
| RTN4 | Reticulon 4 |
| FOXO3 | Forkhead Box O3 |
| AP2B1 | Adaptor Related Protein Complex 2 Subunit Beta 1 |
| TAGAP | T Cell Activation RhoGTPase Activating Protein |
| BRD4 | Bromodomain Containing 4 |
| MTHFR | Methylenetetrahydrofolate Reductase |
| RARRES2 | Retinoic Acid Receptor Responder 2 |
| PTGER4 | Prostaglandin E Receptor 4 |
| CPT2 | Carnitine Palmitoyltransferase 2 |
| TIMP2 | TIMP Metallopeptidase Inhibitor 2 |
| CCND2 | Cyclin D2 |
| MITF | Melanocyte Inducing Transcription Factor |
| SEC23B | SEC23 Homolog B, COPII Coat Complex Component |
| MECOM | MDS1 And EVI1 Complex Locus |
| FTCD | Formimidoyltransferase Cyclodeaminase |
| CARMIL2 | Capping Protein Regulator And Myosin 1 Linker 2 |
| SLC2A1 | Solute Carrier Family 2 Member 1 |
| DSP | Desmoplakin |
| DNTT | DNA Nucleotidylexotransferase |
| TLN1 | Talin 1 |
| ACADVL | Acyl-CoA Dehydrogenase Very Long Chain |
| GALC | Galactosylceramidase |
| DLL1 | Delta Like Canonical Notch Ligand 1 |
| SIGLEC7 | Sialic Acid Binding Ig Like Lectin 7 |
| OPTN | Optineurin |
| CDK2 | Cyclin Dependent Kinase 2 |
| CACNA1B | Calcium Voltage-Gated Channel Subunit Alpha1 B |
| ATP6AP1 | ATPase H+ Transporting Accessory Protein 1 |
| IL9R | Interleukin 9 Receptor |
| H19 | H19 Imprinted Maternally Expressed Transcript |
| CEBPD | CCAAT Enhancer Binding Protein Delta |
| CLEC10A | C-Type Lectin Domain Containing 10A |
| DHX9 | DExH-Box Helicase 9 |
| MYCN | MYCN Proto-Oncogene, BHLH Transcription Factor |
| TIFA | TRAF Interacting Protein With Forkhead Associated Domain |
| SLC15A4 | Solute Carrier Family 15 Member 4 |
| XBP1 | X-Box Binding Protein 1 |
| KNG1 | Kininogen 1 |
| SOX18 | SRY-Box Transcription Factor 18 |
| CD200R1 | CD200 Receptor 1 |
| SFTPC | Surfactant Protein C |
| SRD5A3 | Steroid 5 Alpha-Reductase 3 |
| MT-TL1 | Mitochondrially Encoded TRNA-Leu (UUA/G) 1 |
| WASF2 | WASP Family Member 2 |
| H2AX | H2A.X Variant Histone |
| BTN3A2 | Butyrophilin Subfamily 3 Member A2 |
| CEACAM5 | CEA Cell Adhesion Molecule 5 |
| ABCB4 | ATP Binding Cassette Subfamily B Member 4 |
| USP14 | Ubiquitin Specific Peptidase 14 |
| MIR15B | MicroRNA 15b |
| MIR423 | MicroRNA 423 |
| APPL1 | Adaptor Protein, Phosphotyrosine Interacting With PH Domain And Leucine Zipper 1 |
| HAX1 | HCLS1 Associated Protein X-1 |
| MALAT1 | Metastasis Associated Lung Adenocarcinoma Transcript 1 |
| TRAV24 | T Cell Receptor Alpha Variable 24 |
| TXNRD2 | Thioredoxin Reductase 2 |
| CYP2D6 | Cytochrome P450 Family 2 Subfamily D Member 6 |
| PTK2 | Protein Tyrosine Kinase 2 |
| EPRS1 | Glutamyl-Prolyl-TRNA Synthetase 1 |
| ZMYND11 | Zinc Finger MYND-Type Containing 11 |
| HPS1 | HPS1 Biogenesis Of Lysosomal Organelles Complex 3 Subunit 1 |
| ADAM10 | ADAM Metallopeptidase Domain 10 |
| DNMT3A | DNA Methyltransferase 3 Alpha |
| HCRT | Hypocretin Neuropeptide Precursor |
| EGR1 | Early Growth Response 1 |
| IL12A-AS1 | IL12A Antisense RNA 1 |
| TAL1 | TAL BHLH Transcription Factor 1, Erythroid Differentiation Factor |
| FANCM | FA Complementation Group M |
| SEMA7A | Semaphorin 7A (John Milton Hagen Blood Group) |
| EBAG9 | Estrogen Receptor Binding Site Associated Antigen 9 |
| GCK | Glucokinase |
| KLK3 | Kallikrein Related Peptidase 3 |
| RHOA | Ras Homolog Family Member A |
| LIG1 | DNA Ligase 1 |
| MPIG6B | Megakaryocyte And Platelet Inhibitory Receptor G6b |
| SETBP1 | SET Binding Protein 1 |
| UROS | Uroporphyrinogen III Synthase |
| FPR1 | Formyl Peptide Receptor 1 |
| IRS1 | Insulin Receptor Substrate 1 |
| MSR1 | Macrophage Scavenger Receptor 1 |
| CARD14 | Caspase Recruitment Domain Family Member 14 |
| RHOH | Ras Homolog Family Member H |
| BPIFB1 | BPI Fold Containing Family B Member 1 |
| PRKCZ | Protein Kinase C Zeta |
| NRTN | Neurturin |
| H2BC21 | H2B Clustered Histone 21 |
| SDHA | Succinate Dehydrogenase Complex Flavoprotein Subunit A |
| IL17C | Interleukin 17C |
| CANDF1 | Candidiasis, Familial 1 |
| PKLR | Pyruvate Kinase L/R |
| ARSB | Arylsulfatase B |
| DPM2 | Dolichyl-Phosphate Mannosyltransferase Subunit 2, Regulatory |
| MAP2K7 | Mitogen-Activated Protein Kinase Kinase 7 |
| GAS8 | Growth Arrest Specific 8 |
| MSH5 | MutS Homolog 5 |
| MIR331 | MicroRNA 331 |
| AIS2 | Autoimmune Susceptibility 2 |
| AIS3 | Autoimmune Susceptibility 3 (Vitiligo Specific) |
| AIS4 | Autoimmune Disease, Susceptibility To, 4 |
| GLB1 | Galactosidase Beta 1 |
| MAP2K4 | Mitogen-Activated Protein Kinase Kinase 4 |
| CANX | Calnexin |
| PIK3C2A | Phosphatidylinositol-4-Phosphate 3-Kinase Catalytic Subunit Type 2 Alpha |
| TRBV11-1 | T Cell Receptor Beta Variable 11-1 |
| TRBV11-3 | T Cell Receptor Beta Variable 11-3 |
| PLEK | Pleckstrin |
| LAMC2 | Laminin Subunit Gamma 2 |
| SEPSECS | Sep (O-Phosphoserine) TRNA:Sec (Selenocysteine) TRNA Synthase |
| GALNS | Galactosamine (N-Acetyl)-6-Sulfatase |
| ITPR1 | Inositol 1,4,5-Trisphosphate Receptor Type 1 |
| TGFA | Transforming Growth Factor Alpha |
| FPR2 | Formyl Peptide Receptor 2 |
| RECQL4 | RecQ Like Helicase 4 |
| COG4 | Component Of Oligomeric Golgi Complex 4 |
| ZMYND10 | Zinc Finger MYND-Type Containing 10 |
| UBASH3A | Ubiquitin Associated And SH3 Domain Containing A |
| ZC3H12A | Zinc Finger CCCH-Type Containing 12A |
| CEACAM6 | CEA Cell Adhesion Molecule 6 |
| ELP1 | Elongator Complex Protein 1 |
| MOGS | Mannosyl-Oligosaccharide Glucosidase |
| SYT2 | Synaptotagmin 2 |
| FCGR1B | Fc Fragment Of IgG Receptor Ib |
| VSIG4 | V-Set And Immunoglobulin Domain Containing 4 |
| SDHD | Succinate Dehydrogenase Complex Subunit D |
| BUB3 | BUB3 Mitotic Checkpoint Protein |
| RPA1 | Replication Protein A1 |
| CEP290 | Centrosomal Protein 290 |
| DUSP1 | Dual Specificity Phosphatase 1 |
| ALG6 | ALG6 Alpha-1,3-Glucosyltransferase |
| UBE2N | Ubiquitin Conjugating Enzyme E2 N |
| ATXN2 | Ataxin 2 |
| PTPRJ | Protein Tyrosine Phosphatase Receptor Type J |
| RHCE | Rh Blood Group CcEe Antigens |
| IL34 | Interleukin 34 |
| AZU1 | Azurocidin 1 |
| SCN5A | Sodium Voltage-Gated Channel Alpha Subunit 5 |
| GCG | Glucagon |
| DBT | Dihydrolipoamide Branched Chain Transacylase E2 |
| IRS2 | Insulin Receptor Substrate 2 |
| MMP14 | Matrix Metallopeptidase 14 |
| TCL1A | T Cell Leukemia/Lymphoma 1A |
| SLC35A1 | Solute Carrier Family 35 Member A1 |
| MMUT | Methylmalonyl-CoA Mutase |
| PLAU | Plasminogen Activator, Urokinase |
| ARMC4 | Armadillo Repeat Containing 4 |
| CCDC151 | Coiled-Coil Domain Containing 151 |
| DNAAF4 | Dynein Axonemal Assembly Factor 4 |
| RNASE2 | Ribonuclease A Family Member 2 |
| CD72 | CD72 Molecule |
| GAS6 | Growth Arrest Specific 6 |
| TALDO1 | Transaldolase 1 |
| WDR4 | WD Repeat Domain 4 |
| PHEX | Phosphate Regulating Endopeptidase Homolog X-Linked |
| MIR222 | MicroRNA 222 |
| ABCG2 | ATP Binding Cassette Subfamily G Member 2 (Junior Blood Group) |
| ALDH2 | Aldehyde Dehydrogenase 2 Family Member |
| CDK1 | Cyclin Dependent Kinase 1 |
| RGS1 | Regulator Of G Protein Signaling 1 |
| POLR3B | RNA Polymerase III Subunit B |
| SOX11 | SRY-Box Transcription Factor 11 |
| MC1R | Melanocortin 1 Receptor |
| NFKBIB | NFKB Inhibitor Beta |
| TIMD4 | T Cell Immunoglobulin And Mucin Domain Containing 4 |
| CDK6 | Cyclin Dependent Kinase 6 |
| SF3B1 | Splicing Factor 3b Subunit 1 |
| PTPN3 | Protein Tyrosine Phosphatase Non-Receptor Type 3 |
| JAM3 | Junctional Adhesion Molecule 3 |
| MEF2C | Myocyte Enhancer Factor 2C |
| TAZ | Tafazzin |
| TRIM68 | Tripartite Motif Containing 68 |
| ISL1 | ISL LIM Homeobox 1 |
| FERMT1 | Fermitin Family Member 1 |
| HSPA1A | Heat Shock Protein Family A (Hsp70) Member 1A |
| CSF2RB | Colony Stimulating Factor 2 Receptor Subunit Beta |
| OCA2 | OCA2 Melanosomal Transmembrane Protein |
| TGM5 | Transglutaminase 5 |
| NUP43 | Nucleoporin 43 |
| HRG | Histidine Rich Glycoprotein |
| CD300LB | CD300 Molecule Like Family Member B |
| MAG | Myelin Associated Glycoprotein |
| KPNA2 | Karyopherin Subunit Alpha 2 |
| ACTC1 | Actin Alpha Cardiac Muscle 1 |
| MIR192 | MicroRNA 192 |
| MIRLET7E | MicroRNA Let-7e |
| FOXP2 | Forkhead Box P2 |
| IL31RA | Interleukin 31 Receptor A |
| CYP7B1 | Cytochrome P450 Family 7 Subfamily B Member 1 |
| MAGEA3 | MAGE Family Member A3 |
| ENPP3 | Ectonucleotide Pyrophosphatase/Phosphodiesterase 3 |
| EMP2 | Epithelial Membrane Protein 2 |
| SH3KBP1 | SH3 Domain Containing Kinase Binding Protein 1 |
| MIR203A | MicroRNA 203a |
| PDE4D | Phosphodiesterase 4D |
| ABCC4 | ATP Binding Cassette Subfamily C Member 4 |
| DEFA1 | Defensin Alpha 1 |
| TFR2 | Transferrin Receptor 2 |
| RPL7 | Ribosomal Protein L7 |
| MAPK13 | Mitogen-Activated Protein Kinase 13 |
| NPC1 | NPC Intracellular Cholesterol Transporter 1 |
| ABCC8 | ATP Binding Cassette Subfamily C Member 8 |
| SARM1 | Sterile Alpha And TIR Motif Containing 1 |
| CLDN11 | Claudin 11 |
| CD177 | CD177 Molecule |
| DSC1 | Desmocollin 1 |
| ADGRE5 | Adhesion G Protein-Coupled Receptor E5 |
| TP53BP1 | Tumor Protein P53 Binding Protein 1 |
| FABP5 | Fatty Acid Binding Protein 5 |
| MIR106B | MicroRNA 106b |
| KLRB1 | Killer Cell Lectin Like Receptor B1 |
| TRBV11-2 | T Cell Receptor Beta Variable 11-2 |
| NUP85 | Nucleoporin 85 |
| WASL | WASP Like Actin Nucleation Promoting Factor |
| TACR1 | Tachykinin Receptor 1 |
| VCL | Vinculin |
| ARF1 | ADP Ribosylation Factor 1 |
| SCARB2 | Scavenger Receptor Class B Member 2 |
| ACTN2 | Actinin Alpha 2 |
| CEP57 | Centrosomal Protein 57 |
| MT-CO3 | Mitochondrially Encoded Cytochrome C Oxidase III |
| TNFSF9 | TNF Superfamily Member 9 |
| NCSTN | Nicastrin |
| H3C14 | H3 Clustered Histone 14 |
| LCAT | Lecithin-Cholesterol Acyltransferase |
| VPREB1 | V-Set Pre-B Cell Surrogate Light Chain 1 |
| LRRC56 | Leucine Rich Repeat Containing 56 |
| BMP2 | Bone Morphogenetic Protein 2 |
| CDC25C | Cell Division Cycle 25C |
| NSMCE3 | NSE3 Homolog, SMC5-SMC6 Complex Component |
| LPIN2 | Lipin 2 |
| PCGF2 | Polycomb Group Ring Finger 2 |
| DSG2 | Desmoglein 2 |
| PSMD12 | Proteasome 26S Subunit, Non-ATPase 12 |
| FOXO1 | Forkhead Box O1 |
| ARG2 | Arginase 2 |
| SIAE | Sialic Acid Acetylesterase |
| KPNA1 | Karyopherin Subunit Alpha 1 |
| PIGN | Phosphatidylinositol Glycan Anchor Biosynthesis Class N |
| VPS13B | Vacuolar Protein Sorting 13 Homolog B |
| TRIM13 | Tripartite Motif Containing 13 |
| PDPK1 | 3-Phosphoinositide Dependent Protein Kinase 1 |
| ARHGEF1 | Rho Guanine Nucleotide Exchange Factor 1 |
| CNTNAP2 | Contactin Associated Protein 2 |
| CD1E | CD1e Molecule |
| CMA1 | Chymase 1 |
| MIR9-1 | MicroRNA 9-1 |
| GSTP1 | Glutathione S-Transferase Pi 1 |
| NKX2-1 | NK2 Homeobox 1 |
| MIR92A1 | MicroRNA 92a-1 |
| PSMC4 | Proteasome 26S Subunit, ATPase 4 |
| CD300A | CD300a Molecule |
| HPSE | Heparanase |
| UBC | Ubiquitin C |
| DYRK1A | Dual Specificity Tyrosine Phosphorylation Regulated Kinase 1A |
| ANTXR2 | ANTXR Cell Adhesion Molecule 2 |
| FGF23 | Fibroblast Growth Factor 23 |
| IL20RA | Interleukin 20 Receptor Subunit Alpha |
| KCNN4 | Potassium Calcium-Activated Channel Subfamily N Member 4 |
| GNS | Glucosamine (N-Acetyl)-6-Sulfatase |
| NFKBIE | NFKB Inhibitor Epsilon |
| CSNK2A1 | Casein Kinase 2 Alpha 1 |
| HNMT | Histamine N-Methyltransferase |
| IFNA21 | Interferon Alpha 21 |
| SLC30A10 | Solute Carrier Family 30 Member 10 |
| IGHV3-21 | Immunoglobulin Heavy Variable 3-21 |
| NUP214 | Nucleoporin 214 |
| MTHFD1 | Methylenetetrahydrofolate Dehydrogenase, Cyclohydrolase And Formyltetrahydrofolate Synthetase 1 |
| PRKCA | Protein Kinase C Alpha |
| CLEC1B | C-Type Lectin Domain Family 1 Member B |
| MT-CYB | Mitochondrially Encoded Cytochrome B |
| TRAT1 | T Cell Receptor Associated Transmembrane Adaptor 1 |
| STX3 | Syntaxin 3 |
| VAMP1 | Vesicle Associated Membrane Protein 1 |
| NRXN1 | Neurexin 1 |
| AP2S1 | Adaptor Related Protein Complex 2 Subunit Sigma 1 |
| CDH3 | Cadherin 3 |
| CISD2 | CDGSH Iron Sulfur Domain 2 |
| BATF | Basic Leucine Zipper ATF-Like Transcription Factor |
| SPTLC2 | Serine Palmitoyltransferase Long Chain Base Subunit 2 |
| ATG7 | Autophagy Related 7 |
| XK | X-Linked Kx Blood Group |
| XPO1 | Exportin 1 |
| CCL25 | C-C Motif Chemokine Ligand 25 |
| TRIM8 | Tripartite Motif Containing 8 |
| LRRK2 | Leucine Rich Repeat Kinase 2 |
| IL13RA2 | Interleukin 13 Receptor Subunit Alpha 2 |
| MSMB | Microseminoprotein Beta |
| LMNB2 | Lamin B2 |
| MIR196A1 | MicroRNA 196a-1 |
| IGAN2 | IgA Nephropathy, Susceptibility To, 2 |
| APC2 | APC Regulator Of WNT Signaling Pathway 2 |
| CTBP1 | C-Terminal Binding Protein 1 |
| PPL | Periplakin |
| ACTA2 | Actin Alpha 2, Smooth Muscle |
| AOC3 | Amine Oxidase Copper Containing 3 |
| EHMT1 | Euchromatic Histone Lysine Methyltransferase 1 |
| PHGDH | Phosphoglycerate Dehydrogenase |
| PGR | Progesterone Receptor |
| GATA4 | GATA Binding Protein 4 |
| TICAM2 | Toll Like Receptor Adaptor Molecule 2 |
| ASXL1 | ASXL Transcriptional Regulator 1 |
| HMGCR | 3-Hydroxy-3-Methylglutaryl-CoA Reductase |
| MIR199A1 | MicroRNA 199a-1 |
| TCOF1 | Treacle Ribosome Biogenesis Factor 1 |
| NUP210 | Nucleoporin 210 |
| CHRNA1 | Cholinergic Receptor Nicotinic Alpha 1 Subunit |
| MIR24-1 | MicroRNA 24-1 |
| MR1 | Major Histocompatibility Complex, Class I-Related |
| TRPM1 | Transient Receptor Potential Cation Channel Subfamily M Member 1 |
| CTSL | Cathepsin L |
| MIR93 | MicroRNA 93 |
| MIR25 | MicroRNA 25 |
| GFPT1 | Glutamine--Fructose-6-Phosphate Transaminase 1 |
| PIK3R3 | Phosphoinositide-3-Kinase Regulatory Subunit 3 |
| GNE | Glucosamine (UDP-N-Acetyl)-2-Epimerase/N-Acetylmannosamine Kinase |
| GFI1B | Growth Factor Independent 1B Transcriptional Repressor |
| BST1 | Bone Marrow Stromal Cell Antigen 1 |
| AP3D1 | Adaptor Related Protein Complex 3 Subunit Delta 1 |
| DHODH | Dihydroorotate Dehydrogenase (Quinone) |
| TRIM56 | Tripartite Motif Containing 56 |
| PGF | Placental Growth Factor |
| GSS | Glutathione Synthetase |
| OFD1 | OFD1 Centriole And Centriolar Satellite Protein |
| SPTB | Spectrin Beta, Erythrocytic |
| NGFR | Nerve Growth Factor Receptor |
| PRSS1 | Serine Protease 1 |
| RFX1 | Regulatory Factor X1 |
| GRIN2B | Glutamate Ionotropic Receptor NMDA Type Subunit 2B |
| GAS5 | Growth Arrest Specific 5 |
| SCNN1A | Sodium Channel Epithelial 1 Subunit Alpha |
| KLK7 | Kallikrein Related Peptidase 7 |
| MIR22 | MicroRNA 22 |
| TPM2 | Tropomyosin 2 |
| TNFSF18 | TNF Superfamily Member 18 |
| CDH23 | Cadherin Related 23 |
| NLRP6 | NLR Family Pyrin Domain Containing 6 |
| HRH1 | Histamine Receptor H1 |
| TNFRSF12A | TNF Receptor Superfamily Member 12A |
| DCK | Deoxycytidine Kinase |
| F11R | F11 Receptor |
| RSPH3 | Radial Spoke Head 3 |
| NR4A1 | Nuclear Receptor Subfamily 4 Group A Member 1 |
| AHCY | Adenosylhomocysteinase |
| FGF10 | Fibroblast Growth Factor 10 |
| COG1 | Component Of Oligomeric Golgi Complex 1 |
| EFL1 | Elongation Factor Like GTPase 1 |
| PIAS1 | Protein Inhibitor Of Activated STAT 1 |
| MYOM2 | Myomesin 2 |
| MAP3K1 | Mitogen-Activated Protein Kinase Kinase Kinase 1 |
| KIR3DL2 | Killer Cell Immunoglobulin Like Receptor, Three Ig Domains And Long Cytoplasmic Tail 2 |
| MIR210 | MicroRNA 210 |
| NEFL | Neurofilament Light |
| LHCGR | Luteinizing Hormone/Choriogonadotropin Receptor |
| IFNL4 | Interferon Lambda 4 (Gene/Pseudogene) |
| IL17RB | Interleukin 17 Receptor B |
| NPSR1 | Neuropeptide S Receptor 1 |
| RAD54B | RAD54 Homolog B |
| IFNL2 | Interferon Lambda 2 |
| DCAF1 | DDB1 And CUL4 Associated Factor 1 |
| TBX6 | T-Box Transcription Factor 6 |
| AIP | Aryl Hydrocarbon Receptor Interacting Protein |
| LMX1B | LIM Homeobox Transcription Factor 1 Beta |
| PSENEN | Presenilin Enhancer, Gamma-Secretase Subunit |
| SNRPE | Small Nuclear Ribonucleoprotein Polypeptide E |
| HMGCL | 3-Hydroxy-3-Methylglutaryl-CoA Lyase |
| FANCG | FA Complementation Group G |
| SLC19A1 | Solute Carrier Family 19 Member 1 |
| SMAD7 | SMAD Family Member 7 |
| DDOST | Dolichyl-Diphosphooligosaccharide--Protein Glycosyltransferase Non-Catalytic Subunit |
| NOS1 | Nitric Oxide Synthase 1 |
| GPNMB | Glycoprotein Nmb |
| NFATC4 | Nuclear Factor Of Activated T Cells 4 |
| CSN3 | Casein Kappa |
| EPCAM | Epithelial Cell Adhesion Molecule |
| PTGER2 | Prostaglandin E Receptor 2 |
| DCD | Dermcidin |
| KCNAB2 | Potassium Voltage-Gated Channel Subfamily A Regulatory Beta Subunit 2 |
| IFNLR1 | Interferon Lambda Receptor 1 |
| ELK1 | ETS Transcription Factor ELK1 |
| MIR381 | MicroRNA 381 |
| IFI6 | Interferon Alpha Inducible Protein 6 |
| CDKN2C | Cyclin Dependent Kinase Inhibitor 2C |
| SLC3A1 | Solute Carrier Family 3 Member 1 |
| MIR122 | MicroRNA 122 |
| AGO2 | Argonaute RISC Catalytic Component 2 |
| TYRP1 | Tyrosinase Related Protein 1 |
| SCN4A | Sodium Voltage-Gated Channel Alpha Subunit 4 |
| RPGR | Retinitis Pigmentosa GTPase Regulator |
| NME8 | NME/NM23 Family Member 8 |
| LRRC6 | Leucine Rich Repeat Containing 6 |
| CCDC114 | Coiled-Coil Domain Containing 114 |
| CCDC65 | Coiled-Coil Domain Containing 65 |
| SH2D1B | SH2 Domain Containing 1B |
| IFNA13 | Interferon Alpha 13 |
| ALPP | Alkaline Phosphatase, Placental |
| TREML1 | Triggering Receptor Expressed On Myeloid Cells Like 1 |
| IGHG2 | Immunoglobulin Heavy Constant Gamma 2 (G2m Marker) |
| IFNA5 | Interferon Alpha 5 |
| IFNA6 | Interferon Alpha 6 |
| IFNA8 | Interferon Alpha 8 |
| MPDU1 | Mannose-P-Dolichol Utilization Defect 1 |
| ACKR1 | Atypical Chemokine Receptor 1 (Duffy Blood Group) |
| PMP2 | Peripheral Myelin Protein 2 |
| HADHA | Hydroxyacyl-CoA Dehydrogenase Trifunctional Multienzyme Complex Subunit Alpha |
| AXL | AXL Receptor Tyrosine Kinase |
| SPHK1 | Sphingosine Kinase 1 |
| TERF1 | Telomeric Repeat Binding Factor 1 |
| APOBEC3H | Apolipoprotein B MRNA Editing Enzyme Catalytic Subunit 3H |
| PYY | Peptide YY |
| LY86 | Lymphocyte Antigen 86 |
| PAH | Phenylalanine Hydroxylase |
| PAK1 | P21 (RAC1) Activated Kinase 1 |
| C4BPB | Complement Component 4 Binding Protein Beta |
| IL1RAP | Interleukin 1 Receptor Accessory Protein |
| NLRP9 | NLR Family Pyrin Domain Containing 9 |
| RNF213 | Ring Finger Protein 213 |
| HBEGF | Heparin Binding EGF Like Growth Factor |
| CRLF1 | Cytokine Receptor Like Factor 1 |
| GNA11 | G Protein Subunit Alpha 11 |
| APAF1 | Apoptotic Peptidase Activating Factor 1 |
| EXO1 | Exonuclease 1 |
| VAV3 | Vav Guanine Nucleotide Exchange Factor 3 |
| NNT | Nicotinamide Nucleotide Transhydrogenase |
| WFS1 | Wolframin ER Transmembrane Glycoprotein |
| COL11A2 | Collagen Type XI Alpha 2 Chain |
| CRIPT | CXXC Repeat Containing Interactor Of PDZ3 Domain |
| OAS2 | 2'-5'-Oligoadenylate Synthetase 2 |
| LZTR1 | Leucine Zipper Like Transcription Regulator 1 |
| IL3RA | Interleukin 3 Receptor Subunit Alpha |
| YDJC | YdjC Chitooligosaccharide Deacetylase Homolog |
| COL5A2 | Collagen Type V Alpha 2 Chain |
| VHL | Von Hippel-Lindau Tumor Suppressor |
| SNRPD3 | Small Nuclear Ribonucleoprotein D3 Polypeptide |
| IFNA14 | Interferon Alpha 14 |
| FGFR4 | Fibroblast Growth Factor Receptor 4 |
| JUP | Junction Plakoglobin |
| CRHR1 | Corticotropin Releasing Hormone Receptor 1 |
| DEFA6 | Defensin Alpha 6 |
| ALYREF | Aly/REF Export Factor |
| MIR29B1 | MicroRNA 29b-1 |
| MLANA | Melan-A |
| HRH4 | Histamine Receptor H4 |
| MMP13 | Matrix Metallopeptidase 13 |
| NUP205 | Nucleoporin 205 |
| CYP4F22 | Cytochrome P450 Family 4 Subfamily F Member 22 |
| RIPPLY2 | Ripply Transcriptional Repressor 2 |
| NELFA | Negative Elongation Factor Complex Member A |
| NPHS2 | NPHS2 Stomatin Family Member, Podocin |
| SLAMF8 | SLAM Family Member 8 |
| PSMD4 | Proteasome 26S Subunit, Non-ATPase 4 |
| STK36 | Serine/Threonine Kinase 36 |
| GAS2L2 | Growth Arrest Specific 2 Like 2 |
| MCIDAS | Multiciliate Differentiation And DNA Synthesis Associated Cell Cycle Protein |
| ARAF | A-Raf Proto-Oncogene, Serine/Threonine Kinase |
| SERPINB4 | Serpin Family B Member 4 |
| SIRPA | Signal Regulatory Protein Alpha |
| NFATC3 | Nuclear Factor Of Activated T Cells 3 |
| MIR29A | MicroRNA 29a |
| HLCS | Holocarboxylase Synthetase |
| MAOA | Monoamine Oxidase A |
| MNX1 | Motor Neuron And Pancreas Homeobox 1 |
| AMACR | Alpha-Methylacyl-CoA Racemase |
| SIN3A | SIN3 Transcription Regulator Family Member A |
| ADCY7 | Adenylate Cyclase 7 |
| AQP5 | Aquaporin 5 |
| DRD2 | Dopamine Receptor D2 |
| CHEK1 | Checkpoint Kinase 1 |
| SCN9A | Sodium Voltage-Gated Channel Alpha Subunit 9 |
| ALPK1 | Alpha Kinase 1 |
| GDF15 | Growth Differentiation Factor 15 |
| CYP11A1 | Cytochrome P450 Family 11 Subfamily A Member 1 |
| DAB2IP | DAB2 Interacting Protein |
| SAMD9L | Sterile Alpha Motif Domain Containing 9 Like |
| SOCS2 | Suppressor Of Cytokine Signaling 2 |
| FUCA1 | Alpha-L-Fucosidase 1 |
| SMARCD2 | SWI/SNF Related, Matrix Associated, Actin Dependent Regulator Of Chromatin, Subfamily D, Member 2 |
| GINS1 | GINS Complex Subunit 1 |
| CYP7A1 | Cytochrome P450 Family 7 Subfamily A Member 1 |
| HYOU1 | Hypoxia Up-Regulated 1 |
| TREML2 | Triggering Receptor Expressed On Myeloid Cells Like 2 |
| CPLX1 | Complexin 1 |
| FGFRL1 | Fibroblast Growth Factor Receptor Like 1 |
| SPATA5 | Spermatogenesis Associated 5 |
| RPLP0 | Ribosomal Protein Lateral Stalk Subunit P0 |
| PIBF1 | Progesterone Immunomodulatory Binding Factor 1 |
| SRSF2 | Serine And Arginine Rich Splicing Factor 2 |
| HSD3B2 | Hydroxy-Delta-5-Steroid Dehydrogenase, 3 Beta- And Steroid Delta-Isomerase 2 |
| CAMK2G | Calcium/Calmodulin Dependent Protein Kinase II Gamma |
| CTCF | CCCTC-Binding Factor |
| MSX1 | Msh Homeobox 1 |
| ARSA | Arylsulfatase A |
| PSMD14 | Proteasome 26S Subunit, Non-ATPase 14 |
| PROS1 | Protein S |
| IGHG4 | Immunoglobulin Heavy Constant Gamma 4 (G4m Marker) |
| PTCH1 | Patched 1 |
| LFNG | LFNG O-Fucosylpeptide 3-Beta-N-Acetylglucosaminyltransferase |
| MPV17 | Mitochondrial Inner Membrane Protein MPV17 |
| DLL3 | Delta Like Canonical Notch Ligand 3 |
| WDR73 | WD Repeat Domain 73 |
| MESP2 | Mesoderm Posterior BHLH Transcription Factor 2 |
| MT-TS1 | Mitochondrially Encoded TRNA-Ser (UCN) 1 |
| MT-TS2 | Mitochondrially Encoded TRNA-Ser (AGU/C) 2 |
| MT-TQ | Mitochondrially Encoded TRNA-Gln (CAA/G) |
| PLAT | Plasminogen Activator, Tissue Type |
| CBS | Cystathionine Beta-Synthase |
| RAET1E | Retinoic Acid Early Transcript 1E |
| IL1RL2 | Interleukin 1 Receptor Like 2 |
| PRKN | Parkin RBR E3 Ubiquitin Protein Ligase |
| RERE | Arginine-Glutamic Acid Dipeptide Repeats |
| NUP160 | Nucleoporin 160 |
| ALOX12B | Arachidonate 12-Lipoxygenase, 12R Type |
| IVL | Involucrin |
| DDC | Dopa Decarboxylase |
| KLK6 | Kallikrein Related Peptidase 6 |
| MIR32 | MicroRNA 32 |
| CANDN1 | Candidiasis, Nail 1 (With ICAM1 Deficiency) |
| GNPTAB | N-Acetylglucosamine-1-Phosphate Transferase Subunits Alpha And Beta |
| SLC5A7 | Solute Carrier Family 5 Member 7 |
| RAD54L | RAD54 Like |
| PSMA3 | Proteasome 20S Subunit Alpha 3 |
| MYB | MYB Proto-Oncogene, Transcription Factor |
| LRP5 | LDL Receptor Related Protein 5 |
| SLX4 | SLX4 Structure-Specific Endonuclease Subunit |
| AREG | Amphiregulin |
| NUP37 | Nucleoporin 37 |
| WASF5P | WASP Family Member 5, Pseudogene |
| MMACHC | Metabolism Of Cobalamin Associated C |
| NUP93 | Nucleoporin 93 |
| CYSLTR1 | Cysteinyl Leukotriene Receptor 1 |
| MST1R | Macrophage Stimulating 1 Receptor |
| CXADR | CXADR Ig-Like Cell Adhesion Molecule |
| PARP9 | Poly(ADP-Ribose) Polymerase Family Member 9 |
| NRP1 | Neuropilin 1 |
| PTHLH | Parathyroid Hormone Like Hormone |
| HACE1 | HECT Domain And Ankyrin Repeat Containing E3 Ubiquitin Protein Ligase 1 |
| PSMD5 | Proteasome 26S Subunit, Non-ATPase 5 |
| CNPY3 | Canopy FGF Signaling Regulator 3 |
| PRG4 | Proteoglycan 4 |
| NUMA1 | Nuclear Mitotic Apparatus Protein 1 |
| PSMB11 | Proteasome Subunit Beta 11 |
| RHOD | Ras Homolog Family Member D |
| SERAC1 | Serine Active Site Containing 1 |
| BHLHE40 | Basic Helix-Loop-Helix Family Member E40 |
| IGKV3D-11 | Immunoglobulin Kappa Variable 3D-11 |
| AFP | Alpha Fetoprotein |
| ARHGEF6 | Rac/Cdc42 Guanine Nucleotide Exchange Factor 6 |
| MIR375 | MicroRNA 375 |
| APOBEC1 | Apolipoprotein B MRNA Editing Enzyme Catalytic Subunit 1 |
| F10 | Coagulation Factor X |
| MEX3C | Mex-3 RNA Binding Family Member C |
| PHB | Prohibitin |
| ZBP1 | Z-DNA Binding Protein 1 |
| POMP | Proteasome Maturation Protein |
| OSTM1 | Osteoclastogenesis Associated Transmembrane Protein 1 |
| NEDD4 | NEDD4 E3 Ubiquitin Protein Ligase |
| IFIT2 | Interferon Induced Protein With Tetratricopeptide Repeats 2 |
| KIR2DS3 | Killer Cell Immunoglobulin Like Receptor, Two Ig Domains And Short Cytoplasmic Tail 3 |
| CBFB | Core-Binding Factor Subunit Beta |
| CNBP | CCHC-Type Zinc Finger Nucleic Acid Binding Protein |
| ADCY10 | Adenylate Cyclase 10 |
| PSORS5 | Psoriasis Susceptibility 5 |
| PSORS4 | Psoriasis Susceptibility 4 |
| PSORS6 | Psoriasis Susceptibility 6 |
| PSORS9 | Psoriasis Susceptibility 9 |
| PSORS10 | Psoriasis Susceptibility 10 |
| PSORS3 | Psoriasis Susceptibility 3 |
| PSORS7 | Psoriasis Susceptibility 7 |
| PSORS8 | Psoriasis Susceptibility 8 |
| PSORS11 | Psoriasis Susceptibility 11 |
| MS3 | Multiple Sclerosis, Susceptibility To, 3 |
| AGL | Amylo-Alpha-1, 6-Glucosidase, 4-Alpha-Glucanotransferase |
| RNF31 | Ring Finger Protein 31 |
| HMGB2 | High Mobility Group Box 2 |
| HSPB1 | Heat Shock Protein Family B (Small) Member 1 |
| SDCCAG8 | Serologically Defined Colon Cancer Antigen 8 |
| TBC1D23 | TBC1 Domain Family Member 23 |
| PKM | Pyruvate Kinase M1/2 |
| SGSH | N-Sulfoglucosamine Sulfohydrolase |
| MAPK12 | Mitogen-Activated Protein Kinase 12 |
| NOX1 | NADPH Oxidase 1 |
| KDM4C | Lysine Demethylase 4C |
| SULT1A3 | Sulfotransferase Family 1A Member 3 |
| TXN | Thioredoxin |
| TWIST1 | Twist Family BHLH Transcription Factor 1 |
| INHA | Inhibin Subunit Alpha |
| PLVAP | Plasmalemma Vesicle Associated Protein |
| SSRP1 | Structure Specific Recognition Protein 1 |
| MT-CO2 | Mitochondrially Encoded Cytochrome C Oxidase II |
| CD109 | CD109 Molecule |
| TERF2 | Telomeric Repeat Binding Factor 2 |
| MIR27A | MicroRNA 27a |
| JUND | JunD Proto-Oncogene, AP-1 Transcription Factor Subunit |
| IL1F10 | Interleukin 1 Family Member 10 |
| CDR2 | Cerebellar Degeneration Related Protein 2 |
| BTN3A1 | Butyrophilin Subfamily 3 Member A1 |
| H4-16 | H4 Histone 16 |
| ANKRD17 | Ankyrin Repeat Domain 17 |
| SNX10 | Sorting Nexin 10 |
| BCL2A1 | BCL2 Related Protein A1 |
| GBP1 | Guanylate Binding Protein 1 |
| PRKD1 | Protein Kinase D1 |
| GC | GC Vitamin D Binding Protein |
| SNRPB | Small Nuclear Ribonucleoprotein Polypeptides B And B1 |
| SP140 | SP140 Nuclear Body Protein |
| CCDC115 | Coiled-Coil Domain Containing 115 |
| SLC26A2 | Solute Carrier Family 26 Member 2 |
| EPHB2 | EPH Receptor B2 |
| NSUN2 | NOP2/Sun RNA Methyltransferase 2 |
| SELENOK | Selenoprotein K |
| FTL | Ferritin Light Chain |
| ERCC4 | ERCC Excision Repair 4, Endonuclease Catalytic Subunit |
| UPF1 | UPF1 RNA Helicase And ATPase |
| TMEM70 | Transmembrane Protein 70 |
| CORT | Cortistatin |
| EEF1A1 | Eukaryotic Translation Elongation Factor 1 Alpha 1 |
| SUOX | Sulfite Oxidase |
| ST14 | Suppression Of Tumorigenicity 14 |
| TRBV12-3 | T Cell Receptor Beta Variable 12-3 |
| OTUD7B | OTU Deubiquitinase 7B |
| MIR195 | MicroRNA 195 |
| TRAFD1 | TRAF-Type Zinc Finger Domain Containing 1 |
| BAIAP2 | BAR/IMD Domain Containing Adaptor Protein 2 |
| PIN1 | Peptidylprolyl Cis/Trans Isomerase, NIMA-Interacting 1 |
| LACTB | Lactamase Beta |
| IGHG3 | Immunoglobulin Heavy Constant Gamma 3 (G3m Marker) |
| ATP4A | ATPase H+/K+ Transporting Subunit Alpha |
| KLLN | Killin, P53 Regulated DNA Replication Inhibitor |
| MIR342 | MicroRNA 342 |
| ERCC3 | ERCC Excision Repair 3, TFIIH Core Complex Helicase Subunit |
| IFITM2 | Interferon Induced Transmembrane Protein 2 |
| CYP2C19 | Cytochrome P450 Family 2 Subfamily C Member 19 |
| CDC25A | Cell Division Cycle 25A |
| UTP4 | UTP4 Small Subunit Processome Component |
| GIMAP6 | GTPase, IMAP Family Member 6 |
| SPTLC1 | Serine Palmitoyltransferase Long Chain Base Subunit 1 |
| TGM3 | Transglutaminase 3 |
| GZMH | Granzyme H |
| PSMB10 | Proteasome 20S Subunit Beta 10 |
| C4BPA | Complement Component 4 Binding Protein Alpha |
| IGLC1 | Immunoglobulin Lambda Constant 1 |
| IGKV4-1 | Immunoglobulin Kappa Variable 4-1 |
| IGKV5-2 | Immunoglobulin Kappa Variable 5-2 |
| PIK3R4 | Phosphoinositide-3-Kinase Regulatory Subunit 4 |
| CCNB2 | Cyclin B2 |
| COL10A1 | Collagen Type X Alpha 1 Chain |
| HPS6 | HPS6 Biogenesis Of Lysosomal Organelles Complex 2 Subunit 3 |
| GSR | Glutathione-Disulfide Reductase |
| RORA | RAR Related Orphan Receptor A |
| SGPL1 | Sphingosine-1-Phosphate Lyase 1 |
| UBAC2 | UBA Domain Containing 2 |
| CTC1 | CST Telomere Replication Complex Component 1 |
| ASS1 | Argininosuccinate Synthase 1 |
| PILRA | Paired Immunoglobin Like Type 2 Receptor Alpha |
| VCP | Valosin Containing Protein |
| TRBC1 | T Cell Receptor Beta Constant 1 |
| CLCNKB | Chloride Voltage-Gated Channel Kb |
| OSCAR | Osteoclast Associated Ig-Like Receptor |
| WASF3 | WASP Family Member 3 |
| NCKIPSD | NCK Interacting Protein With SH3 Domain |
| AGA | Aspartylglucosaminidase |
| CLDN19 | Claudin 19 |
| CAPN3 | Calpain 3 |
| DISC1 | DISC1 Scaffold Protein |
| SOX9 | SRY-Box Transcription Factor 9 |
| PLA2G6 | Phospholipase A2 Group VI |
| DNAAF6 | Dynein Axonemal Assembly Factor 6 |
| ITPR2 | Inositol 1,4,5-Trisphosphate Receptor Type 2 |
| C8G | Complement C8 Gamma Chain |
| HPSE2 | Heparanase 2 (Inactive) |
| MUC16 | Mucin 16, Cell Surface Associated |
| CD93 | CD93 Molecule |
| IRAK2 | Interleukin 1 Receptor Associated Kinase 2 |
| TECPR2 | Tectonin Beta-Propeller Repeat Containing 2 |
| A2ML1 | Alpha-2-Macroglobulin Like 1 |
| MIR143 | MicroRNA 143 |
| HRH2 | Histamine Receptor H2 |
| ALMS1 | ALMS1 Centrosome And Basal Body Associated Protein |
| LMOD1 | Leiomodin 1 |
| BMP7 | Bone Morphogenetic Protein 7 |
| FGF4 | Fibroblast Growth Factor 4 |
| RAET1G | Retinoic Acid Early Transcript 1G |
| DMP1 | Dentin Matrix Acidic Phosphoprotein 1 |
| MGP | Matrix Gla Protein |
| ORMDL3 | ORMDL Sphingolipid Biosynthesis Regulator 3 |
| ARHGEF2 | Rho/Rac Guanine Nucleotide Exchange Factor 2 |
| PGLYRP2 | Peptidoglycan Recognition Protein 2 |
| NTF3 | Neurotrophin 3 |
| PPP3CB | Protein Phosphatase 3 Catalytic Subunit Beta |
| FBXL19 | F-Box And Leucine Rich Repeat Protein 19 |
| COG8 | Component Of Oligomeric Golgi Complex 8 |
| CRTAM | Cytotoxic And Regulatory T Cell Molecule |
| MIR424 | MicroRNA 424 |
| BTN3A3 | Butyrophilin Subfamily 3 Member A3 |
| HLA-S | Major Histocompatibility Complex, Class I, S (Pseudogene) |
| SALL4 | Spalt Like Transcription Factor 4 |
| GRHL3 | Grainyhead Like Transcription Factor 3 |
| UBE4A | Ubiquitination Factor E4A |
| RASA2 | RAS P21 Protein Activator 2 |
| LILRA1 | Leukocyte Immunoglobulin Like Receptor A1 |
| PREP | Prolyl Endopeptidase |
| DIP2C | Disco Interacting Protein 2 Homolog C |
| PLEKHM1 | Pleckstrin Homology And RUN Domain Containing M1 |
| DEFA3 | Defensin Alpha 3 |
| LILRB5 | Leukocyte Immunoglobulin Like Receptor B5 |
| MMAB | Metabolism Of Cobalamin Associated B |
| MGMT | O-6-Methylguanine-DNA Methyltransferase |
| FMN1 | Formin 1 |
| HNF1A | HNF1 Homeobox A |
| PLA2G10 | Phospholipase A2 Group X |
| ADORA3 | Adenosine A3 Receptor |
| FAF1 | Fas Associated Factor 1 |
| CALM1 | Calmodulin 1 |
| GJB2 | Gap Junction Protein Beta 2 |
| DUOX1 | Dual Oxidase 1 |
| NTRK2 | Neurotrophic Receptor Tyrosine Kinase 2 |
| EPSTI1 | Epithelial Stromal Interaction 1 |
| ECE1 | Endothelin Converting Enzyme 1 |
| CPLANE1 | Ciliogenesis And Planar Polarity Effector 1 |
| DAXX | Death Domain Associated Protein |
| GATA6 | GATA Binding Protein 6 |
| USP8 | Ubiquitin Specific Peptidase 8 |
| RAD17 | RAD17 Checkpoint Clamp Loader Component |
| RBM14 | RNA Binding Motif Protein 14 |
| REG3A | Regenerating Family Member 3 Alpha |
| HYAL1 | Hyaluronidase 1 |
| DNAH1 | Dynein Axonemal Heavy Chain 1 |
| DNAJB13 | DnaJ Heat Shock Protein Family (Hsp40) Member B13 |
| TTC25 | Tetratricopeptide Repeat Domain 25 |
| CFAP298 | Cilia And Flagella Associated Protein 298 |
| CFAP300 | Cilia And Flagella Associated Protein 300 |
| FST | Follistatin |
| SIGLEC8 | Sialic Acid Binding Ig Like Lectin 8 |
| SNX9 | Sorting Nexin 9 |
| TRIM63 | Tripartite Motif Containing 63 |
| BTRC | Beta-Transducin Repeat Containing E3 Ubiquitin Protein Ligase |
| IFI44L | Interferon Induced Protein 44 Like |
| PRPS2 | Phosphoribosyl Pyrophosphate Synthetase 2 |
| BECN1 | Beclin 1 |
| CLPB | Caseinolytic Mitochondrial Matrix Peptidase Chaperone Subunit B |
| WASF4P | WASP Family Member 4, Pseudogene |
| CAST | Calpastatin |
| RARB | Retinoic Acid Receptor Beta |
| ADGRE3 | Adhesion G Protein-Coupled Receptor E3 |
| MSMO1 | Methylsterol Monooxygenase 1 |
| LOC102723407 | Immunoglobulin Heavy Variable 4-38-2-Like |
| TOP1 | DNA Topoisomerase I |
| UBE3B | Ubiquitin Protein Ligase E3B |
| HJV | Hemojuvelin BMP Co-Receptor |
| PRKD2 | Protein Kinase D2 |
| IGAN1 | IgA Nephropathy |
| GIMAP4 | GTPase, IMAP Family Member 4 |
| FLNB | Filamin B |
| UBE3A | Ubiquitin Protein Ligase E3A |
| NT5E | 5'-Nucleotidase Ecto |
| MAPT | Microtubule Associated Protein Tau |
| PVT1 | Pvt1 Oncogene |
| IVD | Isovaleryl-CoA Dehydrogenase |
| PAX4 | Paired Box 4 |
| BTD | Biotinidase |
| PROCR | Protein C Receptor |
| TNIP2 | TNFAIP3 Interacting Protein 2 |
| TYRO3 | TYRO3 Protein Tyrosine Kinase |
| KL | Klotho |
| APBB1IP | Amyloid Beta Precursor Protein Binding Family B Member 1 Interacting Protein |
| DYNLL1 | Dynein Light Chain LC8-Type 1 |
| OSMR | Oncostatin M Receptor |
| LMBRD1 | LMBR1 Domain Containing 1 |
| CENPF | Centromere Protein F |
| CD2AP | CD2 Associated Protein |
| MBTPS2 | Membrane Bound Transcription Factor Peptidase, Site 2 |
| LAMB3 | Laminin Subunit Beta 3 |
| PRDM10 | PR/SET Domain 10 |
| MIR151A | MicroRNA 151a |
| MCCC2 | Methylcrotonoyl-CoA Carboxylase 2 |
| WDR34 | WD Repeat Domain 34 |
| IL17B | Interleukin 17B |
| FOXP1 | Forkhead Box P1 |
| REN | Renin |
| CETP | Cholesteryl Ester Transfer Protein |
| THBS1 | Thrombospondin 1 |
| EIF4E | Eukaryotic Translation Initiation Factor 4E |
| CARD8 | Caspase Recruitment Domain Family Member 8 |
| DDX39B | DExD-Box Helicase 39B |
| NDP | Norrin Cystine Knot Growth Factor NDP |
| LAGE3 | L Antigen Family Member 3 |
| SEMA4D | Semaphorin 4D |
| FGF3 | Fibroblast Growth Factor 3 |
| HLA-H | Major Histocompatibility Complex, Class I, H (Pseudogene) |
| POLR2L | RNA Polymerase II Subunit L |
| TNFRSF21 | TNF Receptor Superfamily Member 21 |
| SIGLEC9 | Sialic Acid Binding Ig Like Lectin 9 |
| GRIA3 | Glutamate Ionotropic Receptor AMPA Type Subunit 3 |
| PCNT | Pericentrin |
| LGALS8 | Galectin 8 |
| SUPT5H | SPT5 Homolog, DSIF Elongation Factor Subunit |
| PAX6 | Paired Box 6 |
| HGD | Homogentisate 1,2-Dioxygenase |
| NONO | Non-POU Domain Containing Octamer Binding |
| MCPH1 | Microcephalin 1 |
| GPR182 | G Protein-Coupled Receptor 182 |
| SEPTIN9 | Septin 9 |
| MLKL | Mixed Lineage Kinase Domain Like Pseudokinase |
| LDLR | Low Density Lipoprotein Receptor |
| COG5 | Component Of Oligomeric Golgi Complex 5 |
| OASL | 2'-5'-Oligoadenylate Synthetase Like |
| ATOD3 | Dermatitis, Atopic, 3 |
| HEXB | Hexosaminidase Subunit Beta |
| DUSP10 | Dual Specificity Phosphatase 10 |
| LILRA5 | Leukocyte Immunoglobulin Like Receptor A5 |
| DICER1 | Dicer 1, Ribonuclease III |
| PTPN1 | Protein Tyrosine Phosphatase Non-Receptor Type 1 |
| MATR3 | Matrin 3 |
| GPR183 | G Protein-Coupled Receptor 183 |
| IGLC6 | Immunoglobulin Lambda Constant 6 (Gene/Pseudogene) |
| CCNA2 | Cyclin A2 |
| RPA2 | Replication Protein A2 |
| ALPI | Alkaline Phosphatase, Intestinal |
| BCHE | Butyrylcholinesterase |
| MYH11 | Myosin Heavy Chain 11 |
| NR1I2 | Nuclear Receptor Subfamily 1 Group I Member 2 |
| EIF4G1 | Eukaryotic Translation Initiation Factor 4 Gamma 1 |
| MLPH | Melanophilin |
| TSPO | Translocator Protein |
| TCN1 | Transcobalamin 1 |
| DIABLO | Diablo IAP-Binding Mitochondrial Protein |
| RPGRIP1L | RPGRIP1 Like |
| GADD45A | Growth Arrest And DNA Damage Inducible Alpha |
| SMAD2 | SMAD Family Member 2 |
| NAIP | NLR Family Apoptosis Inhibitory Protein |
| SCT | Secretin |
| FCAMR | Fc Fragment Of IgA And IgM Receptor |
| CCL28 | C-C Motif Chemokine Ligand 28 |
| ECM1 | Extracellular Matrix Protein 1 |
| SHARPIN | SHANK Associated RH Domain Interactor |
| SERPINH1 | Serpin Family H Member 1 |
| BAK1 | BCL2 Antagonist/Killer 1 |
| SFPQ | Splicing Factor Proline And Glutamine Rich |
| NDN | Necdin, MAGE Family Member |
| ASGR2 | Asialoglycoprotein Receptor 2 |
| DSG4 | Desmoglein 4 |
| PICALM | Phosphatidylinositol Binding Clathrin Assembly Protein |
| WDR26 | WD Repeat Domain 26 |
| WNT2B | Wnt Family Member 2B |
| NR4A2 | Nuclear Receptor Subfamily 4 Group A Member 2 |
| CMTM6 | CKLF Like MARVEL Transmembrane Domain Containing 6 |
| SEMA3A | Semaphorin 3A |
| ABCC6 | ATP Binding Cassette Subfamily C Member 6 |
| OSGEP | O-Sialoglycoprotein Endopeptidase |
| TP53RK | TP53 Regulating Kinase |
| IQSEC2 | IQ Motif And Sec7 Domain ArfGEF 2 |
| PHF11 | PHD Finger Protein 11 |
| IGHA1 | Immunoglobulin Heavy Constant Alpha 1 |
| POLR1C | RNA Polymerase I And III Subunit C |
| HTR2A | 5-Hydroxytryptamine Receptor 2A |
| POLR3F | RNA Polymerase III Subunit F |
| APLNR | Apelin Receptor |
| MIR198 | MicroRNA 198 |
| POU2F2 | POU Class 2 Homeobox 2 |
| TXLNA | Taxilin Alpha |
| MIR185 | MicroRNA 185 |
| LEF1 | Lymphoid Enhancer Binding Factor 1 |
| MDC1 | Mediator Of DNA Damage Checkpoint 1 |
| CRLF2 | Cytokine Receptor Like Factor 2 |
| FUT8 | Fucosyltransferase 8 |
| CDC73 | Cell Division Cycle 73 |
| HVCN1 | Hydrogen Voltage Gated Channel 1 |
| MIR146B | MicroRNA 146b |
| COQ2 | Coenzyme Q2, Polyprenyltransferase |
| MT-ATP6 | Mitochondrially Encoded ATP Synthase Membrane Subunit 6 |
| SLC39A8 | Solute Carrier Family 39 Member 8 |
| BRIP1 | BRCA1 Interacting Protein C-Terminal Helicase 1 |
| ALG2 | ALG2 Alpha-1,3/1,6-Mannosyltransferase |
| MIR518B | MicroRNA 518b |
| ATOD1 | Dermatitis, Atopic |
| ATOD5 | Dermatitis, Atopic, 5 |
| ATOD6 | Dermatitis, Atopic, 6 |
| SS3 | Sarcoidosis, Susceptibility To, 3 |
| MS2 | Multiple Sclerosis, Susceptibility To, 2 |
| MS4 | Multiple Sclerosis, Susceptiblity To, 4 |
| ATOD7 | Dermatitis, Atopic, Susceptibility To, 7 |
| ATOD8 | Dermatitis, Atopic, 8 |
| ATOD9 | Dermatitis, Atopic, 9 |
| ITLN1 | Intelectin 1 |
| CLC | Charcot-Leyden Crystal Galectin |
| HDC | Histidine Decarboxylase |
| KIR2DL5A | Killer Cell Immunoglobulin Like Receptor, Two Ig Domains And Long Cytoplasmic Tail 5A |
| PRLR | Prolactin Receptor |
| MIR346 | MicroRNA 346 |
| HNF1B | HNF1 Homeobox B |
| CNP | 2',3'-Cyclic Nucleotide 3' Phosphodiesterase |
| ARHGAP45 | Rho GTPase Activating Protein 45 |
| SLC3A2 | Solute Carrier Family 3 Member 2 |
| PRRC2A | Proline Rich Coiled-Coil 2A |
| B3GNT2 | UDP-GlcNAc:BetaGal Beta-1,3-N-Acetylglucosaminyltransferase 2 |
| ALOX15B | Arachidonate 15-Lipoxygenase Type B |
| PSMA5 | Proteasome 20S Subunit Alpha 5 |
| BCS1L | BCS1 Homolog, Ubiquinol-Cytochrome C Reductase Complex Chaperone |
| FBXO32 | F-Box Protein 32 |
| FBL | Fibrillarin |
| USP7 | Ubiquitin Specific Peptidase 7 |
| KIF11 | Kinesin Family Member 11 |
| SLC30A8 | Solute Carrier Family 30 Member 8 |
| HCP5 | HLA Complex P5 |
| BMX | BMX Non-Receptor Tyrosine Kinase |
| BBS1 | Bardet-Biedl Syndrome 1 |
| DNM2 | Dynamin 2 |
| MUC3A | Mucin 3A, Cell Surface Associated |
| GRK3 | G Protein-Coupled Receptor Kinase 3 |
| MIRLET7D | MicroRNA Let-7d |
| DHPS | Deoxyhypusine Synthase |
| ELF4 | E74 Like ETS Transcription Factor 4 |
| LRP1 | LDL Receptor Related Protein 1 |
| SCNN1B | Sodium Channel Epithelial 1 Subunit Beta |
| STT3B | STT3 Oligosaccharyltransferase Complex Catalytic Subunit B |
| CALM3 | Calmodulin 3 |
| CC2D2A | Coiled-Coil And C2 Domain Containing 2A |
| SNAP29 | Synaptosome Associated Protein 29 |
| ARHGAP31 | Rho GTPase Activating Protein 31 |
| ARID1A | AT-Rich Interaction Domain 1A |
| MIR320A | MicroRNA 320a |
| RBBP4 | RB Binding Protein 4, Chromatin Remodeling Factor |
| GAL | Galanin And GMAP Prepropeptide |
| SH3GL1 | SH3 Domain Containing GRB2 Like 1, Endophilin A2 |
| PTAFR | Platelet Activating Factor Receptor |
| MIR23A | MicroRNA 23a |
| IFNA4 | Interferon Alpha 4 |
| IFNA7 | Interferon Alpha 7 |
| ODC1 | Ornithine Decarboxylase 1 |
| DPM1 | Dolichyl-Phosphate Mannosyltransferase Subunit 1, Catalytic |
| ABCC1 | ATP Binding Cassette Subfamily C Member 1 |
| DDR2 | Discoidin Domain Receptor Tyrosine Kinase 2 |
| ADAM33 | ADAM Metallopeptidase Domain 33 |
| MAFB | MAF BZIP Transcription Factor B |
| BAZ1B | Bromodomain Adjacent To Zinc Finger Domain 1B |
| FAH | Fumarylacetoacetate Hydrolase |
| ULBP2 | UL16 Binding Protein 2 |
| SRP54 | Signal Recognition Particle 54 |
| CSNK2B | Casein Kinase 2 Beta |
| PELI3 | Pellino E3 Ubiquitin Protein Ligase Family Member 3 |
| ITGB7 | Integrin Subunit Beta 7 |
| PRDX5 | Peroxiredoxin 5 |
| MIR186 | MicroRNA 186 |
| SLC7A5 | Solute Carrier Family 7 Member 5 |
| CREM | CAMP Responsive Element Modulator |
| NEK9 | NIMA Related Kinase 9 |
| MIR30D | MicroRNA 30d |
| GRP | Gastrin Releasing Peptide |
| BRD2 | Bromodomain Containing 2 |
| HSPA8 | Heat Shock Protein Family A (Hsp70) Member 8 |
| RXRB | Retinoid X Receptor Beta |
| SLC9A3 | Solute Carrier Family 9 Member A3 |
| GMNN | Geminin DNA Replication Inhibitor |
| CHIA | Chitinase Acidic |
| ATP8B1 | ATPase Phospholipid Transporting 8B1 |
| OLR1 | Oxidized Low Density Lipoprotein Receptor 1 |
| MX2 | MX Dynamin Like GTPase 2 |
| PTGDS | Prostaglandin D2 Synthase |
| REG3G | Regenerating Family Member 3 Gamma |
| LRP4 | LDL Receptor Related Protein 4 |
| HNRNPA1 | Heterogeneous Nuclear Ribonucleoprotein A1 |
| MLXIPL | MLX Interacting Protein Like |
| SARS2 | Seryl-TRNA Synthetase 2, Mitochondrial |
| ATP12A | ATPase H+/K+ Transporting Non-Gastric Alpha2 Subunit |
| ALLC | Allantoicase |
| NUDT10 | Nudix Hydrolase 10 |
| CFL1 | Cofilin 1 |
| IFNA17 | Interferon Alpha 17 |
| COL6A1 | Collagen Type VI Alpha 1 Chain |
| UBE2K | Ubiquitin Conjugating Enzyme E2 K |
| RASGRP2 | RAS Guanyl Releasing Protein 2 |
| TONSL | Tonsoku Like, DNA Repair Protein |
| ZCCHC3 | Zinc Finger CCHC-Type Containing 3 |
| RNF182 | Ring Finger Protein 182 |
| MPI | Mannose Phosphate Isomerase |
| XPC | XPC Complex Subunit, DNA Damage Recognition And Repair Factor |
| CALM2 | Calmodulin 2 |
| HTR3A | 5-Hydroxytryptamine Receptor 3A |
| IFNA10 | Interferon Alpha 10 |
| LAIR2 | Leukocyte Associated Immunoglobulin Like Receptor 2 |
| IFNA16 | Interferon Alpha 16 |
| SEC22B | SEC22 Homolog B, Vesicle Trafficking Protein (Gene/Pseudogene) |
| TMEM67 | Transmembrane Protein 67 |
| IGKV2D-29 | Immunoglobulin Kappa Variable 2D-29 |
| VSIR | V-Set Immunoregulatory Receptor |
| LAP3 | Leucine Aminopeptidase 3 |
| PNPLA2 | Patatin Like Phospholipase Domain Containing 2 |
| TBCD | Tubulin Folding Cofactor D |
| TBC1D24 | TBC1 Domain Family Member 24 |
| TFAP2A | Transcription Factor AP-2 Alpha |
| PTH1R | Parathyroid Hormone 1 Receptor |
| TRIP10 | Thyroid Hormone Receptor Interactor 10 |
| MAP3K5 | Mitogen-Activated Protein Kinase Kinase Kinase 5 |
| BCKDHB | Branched Chain Keto Acid Dehydrogenase E1 Subunit Beta |
| WDR35 | WD Repeat Domain 35 |
| CYP2E1 | Cytochrome P450 Family 2 Subfamily E Member 1 |
| DEFA4 | Defensin Alpha 4 |
| ALOXE3 | Arachidonate Lipoxygenase 3 |
| NIPAL4 | NIPA Like Domain Containing 4 |
| STX16 | Syntaxin 16 |
| IARS2 | Isoleucyl-TRNA Synthetase 2, Mitochondrial |
| HAPLN1 | Hyaluronan And Proteoglycan Link Protein 1 |
| EPPIN | Epididymal Peptidase Inhibitor |
| KLK4 | Kallikrein Related Peptidase 4 |
| IGHV4-34 | Immunoglobulin Heavy Variable 4-34 |
| DLG5 | Discs Large MAGUK Scaffold Protein 5 |
| BTNL8 | Butyrophilin Like 8 |
| PKD2 | Polycystin 2, Transient Receptor Potential Cation Channel |
| MIR486-1 | MicroRNA 486-1 |
| CEBPA | CCAAT Enhancer Binding Protein Alpha |
| MMAA | Metabolism Of Cobalamin Associated A |
| TRG | T Cell Receptor Gamma Locus |
| ACVR2B | Activin A Receptor Type 2B |
| KIF5B | Kinesin Family Member 5B |
| RARS1 | Arginyl-TRNA Synthetase 1 |
| FAM111A | Family With Sequence Similarity 111 Member A |
| COG2 | Component Of Oligomeric Golgi Complex 2 |
| UBE2A | Ubiquitin Conjugating Enzyme E2 A |
| GPER1 | G Protein-Coupled Estrogen Receptor 1 |
| PSEN2 | Presenilin 2 |
| PACS1 | Phosphofurin Acidic Cluster Sorting Protein 1 |
| ARL3 | ADP Ribosylation Factor Like GTPase 3 |
| ARFGAP1 | ADP Ribosylation Factor GTPase Activating Protein 1 |
| TNNI3 | Troponin I3, Cardiac Type |
| S100A7A | S100 Calcium Binding Protein A7A |
| GLI1 | GLI Family Zinc Finger 1 |
| GAS2 | Growth Arrest Specific 2 |
| YWHAZ | Tyrosine 3-Monooxygenase/Tryptophan 5-Monooxygenase Activation Protein Zeta |
| SLC18A3 | Solute Carrier Family 18 Member A3 |
| DLL4 | Delta Like Canonical Notch Ligand 4 |
| CMKLR1 | Chemerin Chemokine-Like Receptor 1 |
| ERBB4 | Erb-B2 Receptor Tyrosine Kinase 4 |
| FAS-AS1 | FAS Antisense RNA 1 |
| HERC5 | HECT And RLD Domain Containing E3 Ubiquitin Protein Ligase 5 |
| ROCK1 | Rho Associated Coiled-Coil Containing Protein Kinase 1 |
| CASP7 | Caspase 7 |
| BLOC1S6 | Biogenesis Of Lysosomal Organelles Complex 1 Subunit 6 |
| NTF4 | Neurotrophin 4 |
| GAB2 | GRB2 Associated Binding Protein 2 |
| AMBP | Alpha-1-Microglobulin/Bikunin Precursor |
| IGFBP2 | Insulin Like Growth Factor Binding Protein 2 |
| FLG2 | Filaggrin Family Member 2 |
| HDAC4 | Histone Deacetylase 4 |
| IGHV3-30 | Immunoglobulin Heavy Variable 3-30 |
| CYP1A1 | Cytochrome P450 Family 1 Subfamily A Member 1 |
| OTUD5 | OTU Deubiquitinase 5 |
| CDKN3 | Cyclin Dependent Kinase Inhibitor 3 |
| NQO1 | NAD(P)H Quinone Dehydrogenase 1 |
| NAMPT | Nicotinamide Phosphoribosyltransferase |
| SNAP23 | Synaptosome Associated Protein 23 |
| TRPV1 | Transient Receptor Potential Cation Channel Subfamily V Member 1 |
| SNRPB2 | Small Nuclear Ribonucleoprotein Polypeptide B2 |
| ANKRD55 | Ankyrin Repeat Domain 55 |
| LCE3C | Late Cornified Envelope 3C |
| LCE3B | Late Cornified Envelope 3B |
| RBBP7 | RB Binding Protein 7, Chromatin Remodeling Factor |
| XPA | XPA, DNA Damage Recognition And Repair Factor |
| NFAT5 | Nuclear Factor Of Activated T Cells 5 |
| IGHV1-46 | Immunoglobulin Heavy Variable 1-46 |
| BCOR | BCL6 Corepressor |
| TFF3 | Trefoil Factor 3 |
| PADI2 | Peptidyl Arginine Deiminase 2 |
| SLC6A4 | Solute Carrier Family 6 Member 4 |
| MBL3P | Mannose-Binding Lectin Family Member 3, Pseudogene |
| CD300E | CD300e Molecule |
| NAGS | N-Acetylglutamate Synthase |
| MIR296 | MicroRNA 296 |
| MIRLET7B | MicroRNA Let-7b |
| GTF2H5 | General Transcription Factor IIH Subunit 5 |
| ASNS | Asparagine Synthetase (Glutamine-Hydrolyzing) |
| ARPC5 | Actin Related Protein 2/3 Complex Subunit 5 |
| FGF1 | Fibroblast Growth Factor 1 |
| TAB3 | TGF-Beta Activated Kinase 1 (MAP3K7) Binding Protein 3 |
| COMP | Cartilage Oligomeric Matrix Protein |
| ARHGAP26 | Rho GTPase Activating Protein 26 |
| LDB3 | LIM Domain Binding 3 |
| ACP3 | Acid Phosphatase 3 |
| BMI1 | BMI1 Proto-Oncogene, Polycomb Ring Finger |
| RPS17 | Ribosomal Protein S17 |
| APOC2 | Apolipoprotein C2 |
| G6PC | Glucose-6-Phosphatase Catalytic Subunit |
| MUS81 | MUS81 Structure-Specific Endonuclease Subunit |
| NANOG | Nanog Homeobox |
| MYL2 | Myosin Light Chain 2 |
| VIPR1 | Vasoactive Intestinal Peptide Receptor 1 |
| POLH | DNA Polymerase Eta |
| CTNND1 | Catenin Delta 1 |
| RAPGEF3 | Rap Guanine Nucleotide Exchange Factor 3 |
| MEG3 | Maternally Expressed 3 |
| DDB1 | Damage Specific DNA Binding Protein 1 |
| IGHA2 | Immunoglobulin Heavy Constant Alpha 2 (A2m Marker) |
| NODAL | Nodal Growth Differentiation Factor |
| VAMP3 | Vesicle Associated Membrane Protein 3 |
| KIF15 | Kinesin Family Member 15 |
| LYAR | Ly1 Antibody Reactive |
| UBE2V1 | Ubiquitin Conjugating Enzyme E2 V1 |
| APLN | Apelin |
| VASP | Vasodilator Stimulated Phosphoprotein |
| CCDC47 | Coiled-Coil Domain Containing 47 |
| RPS29 | Ribosomal Protein S29 |
| SKI | SKI Proto-Oncogene |
| KRT19 | Keratin 19 |
| HLA-DPB2 | Major Histocompatibility Complex, Class II, DP Beta 2 (Pseudogene) |
| IBD5 | Inflammatory Bowel Disease 5 |
| CTRL | Chymotrypsin Like |
| SAA4 | Serum Amyloid A4, Constitutive |
| DUSP5 | Dual Specificity Phosphatase 5 |
| IL17RD | Interleukin 17 Receptor D |
| NUP98 | Nucleoporin 98 And 96 Precursor |
| IGK | Immunoglobulin Kappa Locus |
| TRIM32 | Tripartite Motif Containing 32 |
| RPP14 | Ribonuclease P/MRP Subunit P14 |
| RPP21 | Ribonuclease P/MRP Subunit P21 |
| RPP40 | Ribonuclease P/MRP Subunit P40 |
| FAM221B | Family With Sequence Similarity 221 Member B |
| MIR324 | MicroRNA 324 |
| RPS26 | Ribosomal Protein S26 |
| STEAP3 | STEAP3 Metalloreductase |
| PSMA1 | Proteasome 20S Subunit Alpha 1 |
| POU2F1 | POU Class 2 Homeobox 1 |
| FSTL1 | Follistatin Like 1 |
| HBG1 | Hemoglobin Subunit Gamma 1 |
| STX4 | Syntaxin 4 |
| RETREG1 | Reticulophagy Regulator 1 |
| SELENON | Selenoprotein N |
| ADK | Adenosine Kinase |
| MYH10 | Myosin Heavy Chain 10 |
| NTS | Neurotensin |
| LTB4R | Leukotriene B4 Receptor |
| IVNS1ABP | Influenza Virus NS1A Binding Protein |
| ANGPT2 | Angiopoietin 2 |
| PLAAT4 | Phospholipase A And Acyltransferase 4 |
| KLRC4-KLRK1 | KLRC4-KLRK1 Readthrough |
| F2R | Coagulation Factor II Thrombin Receptor |
| TPT1 | Tumor Protein, Translationally-Controlled 1 |
| CFAP410 | Cilia And Flagella Associated Protein 410 |
| ZFAT | Zinc Finger And AT-Hook Domain Containing |
| DGUOK | Deoxyguanosine Kinase |
| DLG1 | Discs Large MAGUK Scaffold Protein 1 |
| SEMA3C | Semaphorin 3C |
| NEK8 | NIMA Related Kinase 8 |
| MKS1 | MKS Transition Zone Complex Subunit 1 |
| B9D2 | B9 Domain Containing 2 |
| NPHP3 | Nephrocystin 3 |
| AOAH | Acyloxyacyl Hydrolase |
| FBLN5 | Fibulin 5 |
| EFEMP2 | EGF Containing Fibulin Extracellular Matrix Protein 2 |
| OPLAH | 5-Oxoprolinase, ATP-Hydrolysing |
| GORAB | Golgin, RAB6 Interacting |
| WDR19 | WD Repeat Domain 19 |
| CTSS | Cathepsin S |
| MIR200C | MicroRNA 200c |
| LIPA | Lipase A, Lysosomal Acid Type |
| PSME3 | Proteasome Activator Subunit 3 |
| FKTN | Fukutin |
| TXNIP | Thioredoxin Interacting Protein |
| UBR4 | Ubiquitin Protein Ligase E3 Component N-Recognin 4 |
| KRT7 | Keratin 7 |
| ENO2 | Enolase 2 |
| AMPH | Amphiphysin |
| VEGFD | Vascular Endothelial Growth Factor D |
| HSPA9 | Heat Shock Protein Family A (Hsp70) Member 9 |
| EIF4EBP1 | Eukaryotic Translation Initiation Factor 4E Binding Protein 1 |
| IGFBP1 | Insulin Like Growth Factor Binding Protein 1 |
| IFI30 | IFI30 Lysosomal Thiol Reductase |
| IFT122 | Intraflagellar Transport 122 |
| FRAS1 | Fraser Extracellular Matrix Complex Subunit 1 |
| CYP17A1 | Cytochrome P450 Family 17 Subfamily A Member 1 |
| TRNT1 | TRNA Nucleotidyl Transferase 1 |
| RNF113A | Ring Finger Protein 113A |
| KIAA1109 | KIAA1109 |
| CASK | Calcium/Calmodulin Dependent Serine Protein Kinase |
| DOLK | Dolichol Kinase |
| TUBB | Tubulin Beta Class I |
| MARCHF8 | Membrane Associated Ring-CH-Type Finger 8 |
| HOTAIR | HOX Transcript Antisense RNA |
| ITPA | Inosine Triphosphatase |
| BLOC1S1 | Biogenesis Of Lysosomal Organelles Complex 1 Subunit 1 |
| RPP38 | Ribonuclease P/MRP Subunit P38 |
| POP5 | POP5 Homolog, Ribonuclease P/MRP Subunit |
| RPP25 | Ribonuclease P And MRP Subunit P25 |
| MIRLET7G | MicroRNA Let-7g |
| IGKV2-29 | Immunoglobulin Kappa Variable 2-29 (Gene/Pseudogene) |
| NPAT | Nuclear Protein, Coactivator Of Histone Transcription |
| IQGAP1 | IQ Motif Containing GTPase Activating Protein 1 |
| JAG1 | Jagged Canonical Notch Ligand 1 |
| PSME2 | Proteasome Activator Subunit 2 |
| PTMA | Prothymosin Alpha |
| ADAD1 | Adenosine Deaminase Domain Containing 1 |
| MIR200A | MicroRNA 200a |
| CYP19A1 | Cytochrome P450 Family 19 Subfamily A Member 1 |
| GABRD | Gamma-Aminobutyric Acid Type A Receptor Subunit Delta |
| CEP126 | Centrosomal Protein 126 |
| IL13RA1 | Interleukin 13 Receptor Subunit Alpha 1 |
| MCOLN2 | Mucolipin 2 |
| WNT4 | Wnt Family Member 4 |
| NIPBL | NIPBL Cohesin Loading Factor |
| MTCP1 | Mature T Cell Proliferation 1 |
| MIR662 | MicroRNA 662 |
| WHCR | Wolf-Hirschhorn Syndrome Chromosome Region |
| MYOD1 | Myogenic Differentiation 1 |
| MYOG | Myogenin |
| PSPC1 | Paraspeckle Component 1 |
| POLR3C | RNA Polymerase III Subunit C |
| POLR3D | RNA Polymerase III Subunit D |
| SYTL3 | Synaptotagmin Like 3 |
| SLAMF9 | SLAM Family Member 9 |
| HDAC6 | Histone Deacetylase 6 |
| RFC2 | Replication Factor C Subunit 2 |
| KLF1 | Kruppel Like Factor 1 |
| ZIC3 | Zic Family Member 3 |
| EYA4 | EYA Transcriptional Coactivator And Phosphatase 4 |
| SCNN1G | Sodium Channel Epithelial 1 Subunit Gamma |
| COL6A2 | Collagen Type VI Alpha 2 Chain |
| IFT172 | Intraflagellar Transport 172 |
| ANO5 | Anoctamin 5 |
| FGF19 | Fibroblast Growth Factor 19 |
| PIP5K1C | Phosphatidylinositol-4-Phosphate 5-Kinase Type 1 Gamma |
| F11 | Coagulation Factor XI |
| FYB1 | FYN Binding Protein 1 |
| COL18A1 | Collagen Type XVIII Alpha 1 Chain |
| APEX1 | Apurinic/Apyrimidinic Endodeoxyribonuclease 1 |
| HDAC2 | Histone Deacetylase 2 |
| GLI3 | GLI Family Zinc Finger 3 |
| CLECL1 | C-Type Lectin Like 1 |
| MAEA | Macrophage Erythroblast Attacher |
| PLA2G1B | Phospholipase A2 Group IB |
| SRP72 | Signal Recognition Particle 72 |
| COL13A1 | Collagen Type XIII Alpha 1 Chain |
| MYSM1 | Myb Like, SWIRM And MPN Domains 1 |
| TREML4 | Triggering Receptor Expressed On Myeloid Cells Like 4 |
| TRPM2 | Transient Receptor Potential Cation Channel Subfamily M Member 2 |
| ACHE | Acetylcholinesterase (Cartwright Blood Group) |
| CDH5 | Cadherin 5 |
| TAB1 | TGF-Beta Activated Kinase 1 (MAP3K7) Binding Protein 1 |
| MAP2K3 | Mitogen-Activated Protein Kinase Kinase 3 |
| TRAF5 | TNF Receptor Associated Factor 5 |
| ADSL | Adenylosuccinate Lyase |
| DAAM2 | Dishevelled Associated Activator Of Morphogenesis 2 |
| MIR205 | MicroRNA 205 |
| MIR494 | MicroRNA 494 |
| G3BP1 | G3BP Stress Granule Assembly Factor 1 |
| KRT9 | Keratin 9 |
| POLR3G | RNA Polymerase III Subunit G |
| HSP90AB1 | Heat Shock Protein 90 Alpha Family Class B Member 1 |
| HHLA2 | HERV-H LTR-Associating 2 |
| PANK2 | Pantothenate Kinase 2 |
| HM13 | Histocompatibility Minor 13 |
| VAMP7 | Vesicle Associated Membrane Protein 7 |
| COL4A4 | Collagen Type IV Alpha 4 Chain |
| PPP3R1 | Protein Phosphatase 3 Regulatory Subunit B, Alpha |
| OPRD1 | Opioid Receptor Delta 1 |
| MPLKIP | M-Phase Specific PLK1 Interacting Protein |
| CGA | Glycoprotein Hormones, Alpha Polypeptide |
| SCHIP1 | Schwannomin Interacting Protein 1 |
| ADAMTS2 | ADAM Metallopeptidase With Thrombospondin Type 1 Motif 2 |
| GLIS3 | GLIS Family Zinc Finger 3 |
| GOLM1 | Golgi Membrane Protein 1 |
| SIDT1 | SID1 Transmembrane Family Member 1 |
| KHDRBS1 | KH RNA Binding Domain Containing, Signal Transduction Associated 1 |
| MRPL28 | Mitochondrial Ribosomal Protein L28 |
| BAG3 | BAG Cochaperone 3 |
| PPP1CC | Protein Phosphatase 1 Catalytic Subunit Gamma |
| GTF2I | General Transcription Factor IIi |
| CCDC22 | Coiled-Coil Domain Containing 22 |
| SLC5A11 | Solute Carrier Family 5 Member 11 |
| FAM167A | Family With Sequence Similarity 167 Member A |
| HEXIM1 | HEXIM P-TEFb Complex Subunit 1 |
| PRKCG | Protein Kinase C Gamma |
| PPP6C | Protein Phosphatase 6 Catalytic Subunit |
| AGFG1 | ArfGAP With FG Repeats 1 |
| IGKV1-33 | Immunoglobulin Kappa Variable 1-33 |
| PLA2G4A | Phospholipase A2 Group IVA |
| CCT5 | Chaperonin Containing TCP1 Subunit 5 |
| IGL | Immunoglobulin Lambda Locus |
| PQBP1 | Polyglutamine Binding Protein 1 |
| PKHD1 | PKHD1 Ciliary IPT Domain Containing Fibrocystin/Polyductin |
| LTK | Leukocyte Receptor Tyrosine Kinase |
| HTN3 | Histatin 3 |
| TRAV27 | T Cell Receptor Alpha Variable 27 |
| RAN | RAN, Member RAS Oncogene Family |
| TRIM28 | Tripartite Motif Containing 28 |
| MB | Myoglobin |
| CD300LD | CD300 Molecule Like Family Member D |
| DEFB124 | Defensin Beta 124 |
| WASHC1 | WASH Complex Subunit 1 |
| AQP3 | Aquaporin 3 (Gill Blood Group) |
| APOBEC3B | Apolipoprotein B MRNA Editing Enzyme Catalytic Subunit 3B |
| SLC37A4 | Solute Carrier Family 37 Member 4 |
| CCNB1 | Cyclin B1 |
| CD300C | CD300c Molecule |
| PIP | Prolactin Induced Protein |
| ALDH3A2 | Aldehyde Dehydrogenase 3 Family Member A2 |
| SLC4A1 | Solute Carrier Family 4 Member 1 (Diego Blood Group) |
| CLEC2B | C-Type Lectin Domain Family 2 Member B |
| TRBC2 | T Cell Receptor Beta Constant 2 |
| GLRA1 | Glycine Receptor Alpha 1 |
| SLC2A10 | Solute Carrier Family 2 Member 10 |
| NSMCE2 | NSE2 (MMS21) Homolog, SMC5-SMC6 Complex SUMO Ligase |
| MYO9A | Myosin IXA |
| RTTN | Rotatin |
| ITPKC | Inositol-Trisphosphate 3-Kinase C |
| MIR99B | MicroRNA 99b |
| PVALB | Parvalbumin |
| PDIA3 | Protein Disulfide Isomerase Family A Member 3 |
| CACNA1H | Calcium Voltage-Gated Channel Subunit Alpha1 H |
| GMPPB | GDP-Mannose Pyrophosphorylase B |
| MIR127 | MicroRNA 127 |
| MIR345 | MicroRNA 345 |
| MIR500A | MicroRNA 500a |
| IL21-AS1 | IL21 Antisense RNA 1 |
| ENSG00000266919 |  |
| WG | Wegener Granulomatosis |
| H6PD | Hexose-6-Phosphate Dehydrogenase/Glucose 1-Dehydrogenase |
| MCM4 | Minichromosome Maintenance Complex Component 4 |
| GALT | Galactose-1-Phosphate Uridylyltransferase |
| TRIM26 | Tripartite Motif Containing 26 |
| SIK3 | SIK Family Kinase 3 |
| TRBV19 | T Cell Receptor Beta Variable 19 |
| GP5 | Glycoprotein V Platelet |
| ANKRD26 | Ankyrin Repeat Domain 26 |
| IGHV3-49 | Immunoglobulin Heavy Variable 3-49 |
| SPTBN2 | Spectrin Beta, Non-Erythrocytic 2 |
| ORM1 | Orosomucoid 1 |
| ATRN | Attractin |
| DHCR7 | 7-Dehydrocholesterol Reductase |
| L2HGDH | L-2-Hydroxyglutarate Dehydrogenase |
| PFKM | Phosphofructokinase, Muscle |
| TRIM38 | Tripartite Motif Containing 38 |
| WNT10B | Wnt Family Member 10B |
| CD151 | CD151 Molecule (Raph Blood Group) |
| KDSR | 3-Ketodihydrosphingosine Reductase |
| TOP3A | DNA Topoisomerase III Alpha |
| GPR101 | G Protein-Coupled Receptor 101 |
| ADAT3 | Adenosine Deaminase TRNA Specific 3 |
| LECT2 | Leukocyte Cell Derived Chemotaxin 2 |
| CARTPT | CART Prepropeptide |
| PRNP | Prion Protein |
| AAAS | Aladin WD Repeat Nucleoporin |
| TPM3 | Tropomyosin 3 |
| CRNN | Cornulin |
| PXN | Paxillin |
| RAET1L | Retinoic Acid Early Transcript 1L |
| RPGRIP1 | RPGR Interacting Protein 1 |
| AIF1 | Allograft Inflammatory Factor 1 |
| MIR615 | MicroRNA 615 |
| INSL3 | Insulin Like 3 |
| FGL2 | Fibrinogen Like 2 |
| FUT4 | Fucosyltransferase 4 |
| PAG1 | Phosphoprotein Membrane Anchor With Glycosphingolipid Microdomains 1 |
| CASP2 | Caspase 2 |
| PLA2G2D | Phospholipase A2 Group IID |
| ROCK2 | Rho Associated Coiled-Coil Containing Protein Kinase 2 |
| EIF2S1 | Eukaryotic Translation Initiation Factor 2 Subunit Alpha |
| ABCD1 | ATP Binding Cassette Subfamily D Member 1 |
| POLR3E | RNA Polymerase III Subunit E |
| IFNK | Interferon Kappa |
| MATK | Megakaryocyte-Associated Tyrosine Kinase |
| ELMO1 | Engulfment And Cell Motility 1 |
| PAK3 | P21 (RAC1) Activated Kinase 3 |
| NTRK3 | Neurotrophic Receptor Tyrosine Kinase 3 |
| CPOX | Coproporphyrinogen Oxidase |
| ALG9 | ALG9 Alpha-1,2-Mannosyltransferase |
| DKK1 | Dickkopf WNT Signaling Pathway Inhibitor 1 |
| GCH1 | GTP Cyclohydrolase 1 |
| PSMB1 | Proteasome 20S Subunit Beta 1 |
| TBL2 | Transducin Beta Like 2 |
| ANK1 | Ankyrin 1 |
| ZMPSTE24 | Zinc Metallopeptidase STE24 |
| ZC3HAV1 | Zinc Finger CCCH-Type Containing, Antiviral 1 |
| SYTL2 | Synaptotagmin Like 2 |
| UHRF1BP1 | UHRF1 Binding Protein 1 |
| PHRF1 | PHD And Ring Finger Domains 1 |
| MAS1L | MAS1 Proto-Oncogene Like, G Protein-Coupled Receptor |
| SYS1 | SYS1 Golgi Trafficking Protein |
| MIR608 | MicroRNA 608 |
| MIR130B | MicroRNA 130b |
| MIR637 | MicroRNA 637 |
| MIR601 | MicroRNA 601 |
| MIR663A | MicroRNA 663a |
| PNPP1 | PNP Pseudogene 1 |
| ROR2 | Receptor Tyrosine Kinase Like Orphan Receptor 2 |
| IFNE | Interferon Epsilon |
| RPS6 | Ribosomal Protein S6 |
| MTUS1 | Microtubule Associated Scaffold Protein 1 |
| SDHC | Succinate Dehydrogenase Complex Subunit C |
| DHX36 | DEAH-Box Helicase 36 |
| ADARB1 | Adenosine Deaminase RNA Specific B1 |
| COL7A1 | Collagen Type VII Alpha 1 Chain |
| NFAM1 | NFAT Activating Protein With ITAM Motif 1 |
| PEBP1 | Phosphatidylethanolamine Binding Protein 1 |
| PUS10 | Pseudouridine Synthase 10 |
| LILRA3 | Leukocyte Immunoglobulin Like Receptor A3 |
| TK2 | Thymidine Kinase 2 |
| XCL1 | X-C Motif Chemokine Ligand 1 |
| POLR3K | RNA Polymerase III Subunit K |
| POLR3H | RNA Polymerase III Subunit H |
| IGSF6 | Immunoglobulin Superfamily Member 6 |
| TBKBP1 | TBK1 Binding Protein 1 |
| RIOK3 | RIO Kinase 3 |
| ELOC | Elongin C |
| FAAH | Fatty Acid Amide Hydrolase |
| KMT2E | Lysine Methyltransferase 2E |
| RASA1 | RAS P21 Protein Activator 1 |
| VAMP8 | Vesicle Associated Membrane Protein 8 |
| MT-TK | Mitochondrially Encoded TRNA-Lys (AAA/G) |
| EDNRA | Endothelin Receptor Type A |
| CYP3A5 | Cytochrome P450 Family 3 Subfamily A Member 5 |
| SMARCA1 | SWI/SNF Related, Matrix Associated, Actin Dependent Regulator Of Chromatin, Subfamily A, Member 1 |
| TOPBP1 | DNA Topoisomerase II Binding Protein 1 |
| PLD2 | Phospholipase D2 |
| MMP7 | Matrix Metallopeptidase 7 |
| VSTM1 | V-Set And Transmembrane Domain Containing 1 |
| NUP188 | Nucleoporin 188 |
| PLN | Phospholamban |
| IDS | Iduronate 2-Sulfatase |
| DECR1 | 2,4-Dienoyl-CoA Reductase 1 |
| CACNA1A | Calcium Voltage-Gated Channel Subunit Alpha1 A |
| FGL1 | Fibrinogen Like 1 |
| ACADM | Acyl-CoA Dehydrogenase Medium Chain |
| ATP6V1A | ATPase H+ Transporting V1 Subunit A |
| MYO1E | Myosin IE |
| HSD11B1 | Hydroxysteroid 11-Beta Dehydrogenase 1 |
| MYF5 | Myogenic Factor 5 |
| DDX1 | DEAD-Box Helicase 1 |
| HERC2 | HECT And RLD Domain Containing E3 Ubiquitin Protein Ligase 2 |
| CEACAM8 | CEA Cell Adhesion Molecule 8 |
| SMC3 | Structural Maintenance Of Chromosomes 3 |
| REV3L | REV3 Like, DNA Directed Polymerase Zeta Catalytic Subunit |
| FANCL | FA Complementation Group L |
| UBE2T | Ubiquitin Conjugating Enzyme E2 T |
| EXOSC9 | Exosome Component 9 |
| PTPRN2 | Protein Tyrosine Phosphatase Receptor Type N2 |
| GSTM1 | Glutathione S-Transferase Mu 1 |
| DDX60L | DExD/H-Box 60 Like |
| SLCO1B1 | Solute Carrier Organic Anion Transporter Family Member 1B1 |
| PIM2 | Pim-2 Proto-Oncogene, Serine/Threonine Kinase |
| HLTF | Helicase Like Transcription Factor |
| CXCL14 | C-X-C Motif Chemokine Ligand 14 |
| HTR1A | 5-Hydroxytryptamine Receptor 1A |
| ADAMTSL1 | ADAMTS Like 1 |
| RNF34 | Ring Finger Protein 34 |
| GPHN | Gephyrin |
| NFIX | Nuclear Factor I X |
| ROR1 | Receptor Tyrosine Kinase Like Orphan Receptor 1 |
| DEFB118 | Defensin Beta 118 |
| DEFB127 | Defensin Beta 127 |
| ATF3 | Activating Transcription Factor 3 |
| LAMA5 | Laminin Subunit Alpha 5 |
| CSHL1 | Chorionic Somatomammotropin Hormone Like 1 |
| IGLL5 | Immunoglobulin Lambda Like Polypeptide 5 |
| ADORA1 | Adenosine A1 Receptor |
| YES1 | YES Proto-Oncogene 1, Src Family Tyrosine Kinase |
| CES1 | Carboxylesterase 1 |
| SPPL2B | Signal Peptide Peptidase Like 2B |
| EIF2AK4 | Eukaryotic Translation Initiation Factor 2 Alpha Kinase 4 |
| CYFIP2 | Cytoplasmic FMR1 Interacting Protein 2 |
| SOX10 | SRY-Box Transcription Factor 10 |
| TRAV8-4 | T Cell Receptor Alpha Variable 8-4 |
| TRAV19 | T Cell Receptor Alpha Variable 19 |
| NELFCD | Negative Elongation Factor Complex Member C/D |
| COX4I2 | Cytochrome C Oxidase Subunit 4I2 |
| IFTAP | Intraflagellar Transport Associated Protein |
| THY1 | Thy-1 Cell Surface Antigen |
| PNPLA5 | Patatin Like Phospholipase Domain Containing 5 |
| WHAMM | WASP Homolog Associated With Actin, Golgi Membranes And Microtubules |
| MIR433 | MicroRNA 433 |
| MIR484 | MicroRNA 484 |
| MIR208A | MicroRNA 208a |
| MIR769 | MicroRNA 769 |
| MIR638 | MicroRNA 638 |
| MIR654 | MicroRNA 654 |
| MIR92B | MicroRNA 92b |
| MIR642A | MicroRNA 642a |
| MIR657 | MicroRNA 657 |
| MIR612 | MicroRNA 612 |
| MIR611 | MicroRNA 611 |
| MIR629 | MicroRNA 629 |
| MIR557 | MicroRNA 557 |
| MIR575 | MicroRNA 575 |
| MIR583 | MicroRNA 583 |
| MIR325 | MicroRNA 325 |
| MIR622 | MicroRNA 622 |
| MIR658 | MicroRNA 658 |
| MIR596 | MicroRNA 596 |
| MIR600 | MicroRNA 600 |
| MIR602 | MicroRNA 602 |
| SLEH1 | Systemic Lupus Erythematosus With Hemolytic Anemia 1 |
| SLEN1 | Systemic Lupus Erythematosus With Nephritis 1 |
| SLEN2 | Systemic Lupus Erythematosus With Nephritis 2 |
| SLEN3 | Systemic Lupus Erythematosus With Nephritis 3 |
| CERS3 | Ceramide Synthase 3 |
| P2RX5 | Purinergic Receptor P2X 5 |
| MYF6 | Myogenic Factor 6 |
| IGHV3-33 | Immunoglobulin Heavy Variable 3-33 |
| HTN1 | Histatin 1 |
| ATP6V1B2 | ATPase H+ Transporting V1 Subunit B2 |
| GPR65 | G Protein-Coupled Receptor 65 |
| TGM6 | Transglutaminase 6 |
| SON | SON DNA Binding Protein |
| UBASH3B | Ubiquitin Associated And SH3 Domain Containing B |
| LAMP3 | Lysosomal Associated Membrane Protein 3 |
| PSMC6 | Proteasome 26S Subunit, ATPase 6 |
| SLC25A22 | Solute Carrier Family 25 Member 22 |
| KIAA0586 | KIAA0586 |
| CRK | CRK Proto-Oncogene, Adaptor Protein |
| NOX5 | NADPH Oxidase 5 |
| GNB3 | G Protein Subunit Beta 3 |
| ESR2 | Estrogen Receptor 2 |
| HMMR | Hyaluronan Mediated Motility Receptor |
| IGHV1-69 | Immunoglobulin Heavy Variable 1-69 |
| IGHV1-2 | Immunoglobulin Heavy Variable 1-2 |
| IGHV3-13 | Immunoglobulin Heavy Variable 3-13 |
| IGHV4-59 | Immunoglobulin Heavy Variable 4-59 |
| IGHV2-70 | Immunoglobulin Heavy Variable 2-70 |
| CYSLTR2 | Cysteinyl Leukotriene Receptor 2 |
| PLCD1 | Phospholipase C Delta 1 |
| TRPM7 | Transient Receptor Potential Cation Channel Subfamily M Member 7 |
| PSME1 | Proteasome Activator Subunit 1 |
| DEFB119 | Defensin Beta 119 |
| PES1 | Pescadillo Ribosomal Biogenesis Factor 1 |
| COL6A3 | Collagen Type VI Alpha 3 Chain |
| CDC20 | Cell Division Cycle 20 |
| BAG6 | BAG Cochaperone 6 |
| TNNT2 | Troponin T2, Cardiac Type |
| DYNC2LI1 | Dynein Cytoplasmic 2 Light Intermediate Chain 1 |
| FOLH1 | Folate Hydrolase 1 |
| UNC13B | Unc-13 Homolog B |
| SCP2 | Sterol Carrier Protein 2 |
| LAMA4 | Laminin Subunit Alpha 4 |
| ARPC4 | Actin Related Protein 2/3 Complex Subunit 4 |
| SLA2 | Src Like Adaptor 2 |
| YY1 | YY1 Transcription Factor |
| SLC28A1 | Solute Carrier Family 28 Member 1 |
| TBL1XR1 | TBL1X Receptor 1 |
| SCO2 | Synthesis Of Cytochrome C Oxidase 2 |
| IGHV3-48 | Immunoglobulin Heavy Variable 3-48 |
| IGHV3-7 | Immunoglobulin Heavy Variable 3-7 |
| IGHV3-11 | Immunoglobulin Heavy Variable 3-11 (Gene/Pseudogene) |
| IGHV3-53 | Immunoglobulin Heavy Variable 3-53 |
| IGHV4-39 | Immunoglobulin Heavy Variable 4-39 |
| IGHV2-5 | Immunoglobulin Heavy Variable 2-5 |
| PCBP2 | Poly(RC) Binding Protein 2 |
| KDM1A | Lysine Demethylase 1A |
| LORICRIN | Loricrin Cornified Envelope Precursor Protein |
| ATRIP | ATR Interacting Protein |
| PSMA6 | Proteasome 20S Subunit Alpha 6 |
| EWSR1 | EWS RNA Binding Protein 1 |
| RPS7 | Ribosomal Protein S7 |
| TRBV7-9 | T Cell Receptor Beta Variable 7-9 |
| ABHD5 | Abhydrolase Domain Containing 5 |
| MLF1 | Myeloid Leukemia Factor 1 |
| CLN3 | CLN3 Lysosomal/Endosomal Transmembrane Protein, Battenin |
| SUFU | SUFU Negative Regulator Of Hedgehog Signaling |
| PITX2 | Paired Like Homeodomain 2 |
| OPRK1 | Opioid Receptor Kappa 1 |
| DPYD | Dihydropyrimidine Dehydrogenase |
| ALCAM | Activated Leukocyte Cell Adhesion Molecule |
| PLXND1 | Plexin D1 |
| RNF114 | Ring Finger Protein 114 |
| DEFB126 | Defensin Beta 126 |
| HPGD | 15-Hydroxyprostaglandin Dehydrogenase |
| RN7SK | RNA Component Of 7SK Nuclear Ribonucleoprotein |
| ID1 | Inhibitor Of DNA Binding 1, HLH Protein |
| TRAV29DV5 | T Cell Receptor Alpha Variable 29/Delta Variable 5 (Gene/Pseudogene) |
| DEK | DEK Proto-Oncogene |
| DPEP1 | Dipeptidase 1 |
| TRIM31 | Tripartite Motif Containing 31 |
| CSN2 | Casein Beta |
| ARFRP1 | ADP Ribosylation Factor Related Protein 1 |
| MMP19 | Matrix Metallopeptidase 19 |
| GIMAP2 | GTPase, IMAP Family Member 2 |
| TNXB | Tenascin XB |
| RHBDF2 | Rhomboid 5 Homolog 2 |
| FBXL7 | F-Box And Leucine Rich Repeat Protein 7 |
| LAT2 | Linker For Activation Of T Cells Family Member 2 |
| IDO2 | Indoleamine 2,3-Dioxygenase 2 |
| IL22RA2 | Interleukin 22 Receptor Subunit Alpha 2 |
| WNT5A | Wnt Family Member 5A |
| IGHD | Immunoglobulin Heavy Constant Delta |
| HNRNPK | Heterogeneous Nuclear Ribonucleoprotein K |
| CKS1B | CDC28 Protein Kinase Regulatory Subunit 1B |
| GFRA1 | GDNF Family Receptor Alpha 1 |
| PSMB3 | Proteasome 20S Subunit Beta 3 |
| TPH1 | Tryptophan Hydroxylase 1 |
| SNRPC | Small Nuclear Ribonucleoprotein Polypeptide C |
| PRKD3 | Protein Kinase D3 |
| DGKZ | Diacylglycerol Kinase Zeta |
| RAB6A | RAB6A, Member RAS Oncogene Family |
| CUL4B | Cullin 4B |
| AK1 | Adenylate Kinase 1 |
| ORM2 | Orosomucoid 2 |
| RBX1 | Ring-Box 1 |
| TCTN2 | Tectonic Family Member 2 |
| B9D1 | B9 Domain Containing 1 |
| TMEM216 | Transmembrane Protein 216 |
| TFEB | Transcription Factor EB |
| OAT | Ornithine Aminotransferase |
| AHSG | Alpha 2-HS Glycoprotein |
| SHANK3 | SH3 And Multiple Ankyrin Repeat Domains 3 |
| CYP1A2 | Cytochrome P450 Family 1 Subfamily A Member 2 |
| RPS10 | Ribosomal Protein S10 |
| HSPE1 | Heat Shock Protein Family E (Hsp10) Member 1 |
| CLEC4G | C-Type Lectin Domain Family 4 Member G |
| SMG7 | SMG7 Nonsense Mediated MRNA Decay Factor |
| DPYSL5 | Dihydropyrimidinase Like 5 |
| FCMR | Fc Fragment Of IgM Receptor |
| SMARCE1 | SWI/SNF Related, Matrix Associated, Actin Dependent Regulator Of Chromatin, Subfamily E, Member 1 |
| LOC108684022 | FRAXA Repeat Instability Region |
| SERPINB9 | Serpin Family B Member 9 |
| MYBPC3 | Myosin Binding Protein C3 |
| CHIC2 | Cysteine Rich Hydrophobic Domain 2 |
| EOGT | EGF Domain Specific O-Linked N-Acetylglucosamine Transferase |
| SLC12A3 | Solute Carrier Family 12 Member 3 |
| SLC4A2 | Solute Carrier Family 4 Member 2 |
| TNFRSF6B | TNF Receptor Superfamily Member 6b |
| XRCC1 | X-Ray Repair Cross Complementing 1 |
| ABCC2 | ATP Binding Cassette Subfamily C Member 2 |
| IGKV2-40 | Immunoglobulin Kappa Variable 2-40 |
| MID1 | Midline 1 |
| RDX | Radixin |
| CTRC | Chymotrypsin C |
| NABP1 | Nucleic Acid Binding Protein 1 |
| GIMAP8 | GTPase, IMAP Family Member 8 |
| GULP1 | GULP PTB Domain Containing Engulfment Adaptor 1 |
| YTHDF2 | YTH N6-Methyladenosine RNA Binding Protein 2 |
| CD101 | CD101 Molecule |
| TRAV9-2 | T Cell Receptor Alpha Variable 9-2 |
| TRAV18 | T Cell Receptor Alpha Variable 18 |
| TRAV40 | T Cell Receptor Alpha Variable 40 |
| TRAV14DV4 | T Cell Receptor Alpha Variable 14/Delta Variable 4 |
| TRAV2 | T Cell Receptor Alpha Variable 2 |
| TRAV38-1 | T Cell Receptor Alpha Variable 38-1 |
| TRAV38-2DV8 | T Cell Receptor Alpha Variable 38-2/Delta Variable 8 |
| TRAV9-1 | T Cell Receptor Alpha Variable 9-1 |
| PEX5 | Peroxisomal Biogenesis Factor 5 |
| GRIN1 | Glutamate Ionotropic Receptor NMDA Type Subunit 1 |
| SLC7A11 | Solute Carrier Family 7 Member 11 |
| CRBN | Cereblon |
| AHCTF1 | AT-Hook Containing Transcription Factor 1 |
| COL4A5 | Collagen Type IV Alpha 5 Chain |
| EPB42 | Erythrocyte Membrane Protein Band 4.2 |
| OGDH | Oxoglutarate Dehydrogenase |
| TMEM189 | Transmembrane Protein 189 |
| IGHV3-9 | Immunoglobulin Heavy Variable 3-9 |
| MAGEC1 | MAGE Family Member C1 |
| IGHMBP2 | Immunoglobulin Mu DNA Binding Protein 2 |
| RAB38 | RAB38, Member RAS Oncogene Family |
| MYO1G | Myosin IG |
| LCT | Lactase |
| NRON | Non-Coding Repressor Of NFAT |
| ATG12 | Autophagy Related 12 |
| CLSPN | Claspin |
| STOML1 | Stomatin Like 1 |
| ANKRD49 | Ankyrin Repeat Domain 49 |
| MEX3D | Mex-3 RNA Binding Family Member D |
| PAGR1 | PAXIP1 Associated Glutamate Rich Protein 1 |
| WASH3P | WASP Family Homolog 3, Pseudogene |
| MIR483 | MicroRNA 483 |
| WASH6P | WASP Family Homolog 6, Pseudogene |
| CPA1 | Carboxypeptidase A1 |
| FDPS | Farnesyl Diphosphate Synthase |
| DBNL | Drebrin Like |
| NRDE2 | NRDE-2, Necessary For RNA Interference, Domain Containing |
| B4GALT1 | Beta-1,4-Galactosyltransferase 1 |
| RC3H1 | Ring Finger And CCCH-Type Domains 1 |
| LEPQTL1 | Leptin, Serum Levels Of |
| LGR4 | Leucine Rich Repeat Containing G Protein-Coupled Receptor 4 |
| PSMC1 | Proteasome 26S Subunit, ATPase 1 |
| POLD1 | DNA Polymerase Delta 1, Catalytic Subunit |
| SLC22A5 | Solute Carrier Family 22 Member 5 |
| DCDC2 | Doublecortin Domain Containing 2 |
| B3GAT1 | Beta-1,3-Glucuronyltransferase 1 |
| MIR31 | MicroRNA 31 |
| PSMA4 | Proteasome 20S Subunit Alpha 4 |
| PPP3CC | Protein Phosphatase 3 Catalytic Subunit Gamma |
| MAP3K3 | Mitogen-Activated Protein Kinase Kinase Kinase 3 |
| NARS1 | Asparaginyl-TRNA Synthetase 1 |
| UBE2D1 | Ubiquitin Conjugating Enzyme E2 D1 |
| FABP2 | Fatty Acid Binding Protein 2 |
| PMAIP1 | Phorbol-12-Myristate-13-Acetate-Induced Protein 1 |
| PCSK1 | Proprotein Convertase Subtilisin/Kexin Type 1 |
| TJP2 | Tight Junction Protein 2 |
| AGT | Angiotensinogen |
| FTSJ3 | FtsJ RNA 2'-O-Methyltransferase 3 |
| GTF2E2 | General Transcription Factor IIE Subunit 2 |
| PACRG | Parkin Coregulated |
| E2F1 | E2F Transcription Factor 1 |
| IGKV3-20 | Immunoglobulin Kappa Variable 3-20 |
| UBE2D3 | Ubiquitin Conjugating Enzyme E2 D3 |
| IFN1@ | Interferon, Type 1, Cluster |
| ARRB2 | Arrestin Beta 2 |
| KIF21B | Kinesin Family Member 21B |
| TACR3 | Tachykinin Receptor 3 |
| APOA2 | Apolipoprotein A2 |
| RAD51C | RAD51 Paralog C |
| FANCE | FA Complementation Group E |
| GRN | Granulin Precursor |
| BSG | Basigin (Ok Blood Group) |
| FECH | Ferrochelatase |
| ARID1B | AT-Rich Interaction Domain 1B |
| THOC6 | THO Complex 6 |
| TUFM | Tu Translation Elongation Factor, Mitochondrial |
| MIR214 | MicroRNA 214 |
| HTRA1 | HtrA Serine Peptidase 1 |
| TFF2 | Trefoil Factor 2 |
| FOXE1 | Forkhead Box E1 |
| ICAM5 | Intercellular Adhesion Molecule 5 |
| KYNU | Kynureninase |
| ENPP2 | Ectonucleotide Pyrophosphatase/Phosphodiesterase 2 |
| NAT2 | N-Acetyltransferase 2 |
| TRAV12-2 | T Cell Receptor Alpha Variable 12-2 |
| CD99 | CD99 Molecule (Xg Blood Group) |
| NPPB | Natriuretic Peptide B |
| PSMC3 | Proteasome 26S Subunit, ATPase 3 |
| CUL1 | Cullin 1 |
| GBP2 | Guanylate Binding Protein 2 |
| DRD1 | Dopamine Receptor D1 |
| ZEB2 | Zinc Finger E-Box Binding Homeobox 2 |
| GLRB | Glycine Receptor Beta |
| NGLY1 | N-Glycanase 1 |
| TUBB4B | Tubulin Beta 4B Class IVb |
| RPS27 | Ribosomal Protein S27 |
| SIGLEC15 | Sialic Acid Binding Ig Like Lectin 15 |
| RPL15 | Ribosomal Protein L15 |
| GLI2 | GLI Family Zinc Finger 2 |
| TKFC | Triokinase And FMN Cyclase |
| SPARC | Secreted Protein Acidic And Cysteine Rich |
| CDH15 | Cadherin 15 |
| PSMD3 | Proteasome 26S Subunit, Non-ATPase 3 |
| CRCP | CGRP Receptor Component |
| MGAM | Maltase-Glucoamylase |
| CADM1 | Cell Adhesion Molecule 1 |
| FBXO38 | F-Box Protein 38 |
| CRISP3 | Cysteine Rich Secretory Protein 3 |
| SNRPA | Small Nuclear Ribonucleoprotein Polypeptide A |
| JAM2 | Junctional Adhesion Molecule 2 |
| TRAV22 | T Cell Receptor Alpha Variable 22 |
| KIF3A | Kinesin Family Member 3A |
| RXRA | Retinoid X Receptor Alpha |
| GTF2IRD1 | GTF2I Repeat Domain Containing 1 |
| DEFB104A | Defensin Beta 104A |
| LIG3 | DNA Ligase 3 |
| PSMC5 | Proteasome 26S Subunit, ATPase 5 |
| MYOC | Myocilin |
| PSMD1 | Proteasome 26S Subunit, Non-ATPase 1 |
| MTAP | Methylthioadenosine Phosphorylase |
| SLC7A9 | Solute Carrier Family 7 Member 9 |
| GJB3 | Gap Junction Protein Beta 3 |
| GJB6 | Gap Junction Protein Beta 6 |
| FLVCR1 | FLVCR Heme Transporter 1 |
| MAP2K6 | Mitogen-Activated Protein Kinase Kinase 6 |
| IL36B | Interleukin 36 Beta |
| APOBEC3C | Apolipoprotein B MRNA Editing Enzyme Catalytic Subunit 3C |
| SECTM1 | Secreted And Transmembrane 1 |
| AP2M1 | Adaptor Related Protein Complex 2 Subunit Mu 1 |
| IFT20 | Intraflagellar Transport 20 |
| TRAV21 | T Cell Receptor Alpha Variable 21 |
| NOTCH3 | Notch Receptor 3 |
| NOG | Noggin |
| TPM1 | Tropomyosin 1 |
| ICAM4 | Intercellular Adhesion Molecule 4 (Landsteiner-Wiener Blood Group) |
| MUC4 | Mucin 4, Cell Surface Associated |
| LYNX1 | Ly6/Neurotoxin 1 |
| IGKV1-5 | Immunoglobulin Kappa Variable 1-5 |
| IGKV2D-28 | Immunoglobulin Kappa Variable 2D-28 |
| AZI2 | 5-Azacytidine Induced 2 |
| ECSIT | ECSIT Signaling Integrator |
| DUSP6 | Dual Specificity Phosphatase 6 |
| PSMB2 | Proteasome 20S Subunit Beta 2 |
| DEFB103A | Defensin Beta 103A |
| AKAP9 | A-Kinase Anchoring Protein 9 |
| MIR17HG | MiR-17-92a-1 Cluster Host Gene |
| HR | HR Lysine Demethylase And Nuclear Receptor Corepressor |
| IGFBP3 | Insulin Like Growth Factor Binding Protein 3 |
| DUSP2 | Dual Specificity Phosphatase 2 |
| RPS15 | Ribosomal Protein S15 |
| DNAH14 | Dynein Axonemal Heavy Chain 14 |
| TNC | Tenascin C |
| RAB11A | RAB11A, Member RAS Oncogene Family |
| PSMB6 | Proteasome 20S Subunit Beta 6 |
| SYVN1 | Synoviolin 1 |
| PSMB7 | Proteasome 20S Subunit Beta 7 |
| PSMD7 | Proteasome 26S Subunit, Non-ATPase 7 |
| PSMD13 | Proteasome 26S Subunit, Non-ATPase 13 |
| PSMC2 | Proteasome 26S Subunit, ATPase 2 |
| CLDN16 | Claudin 16 |
| IGLV6-57 | Immunoglobulin Lambda Variable 6-57 |
| IGLV3-21 | Immunoglobulin Lambda Variable 3-21 |
| IGLV3-25 | Immunoglobulin Lambda Variable 3-25 |
| IGLC3 | Immunoglobulin Lambda Constant 3 (Kern-Oz+ Marker) |
| IGLV1-40 | Immunoglobulin Lambda Variable 1-40 |
| IGLV1-44 | Immunoglobulin Lambda Variable 1-44 |
| IGLV1-51 | Immunoglobulin Lambda Variable 1-51 |
| IGLV2-14 | Immunoglobulin Lambda Variable 2-14 |
| IGLV4-3 | Immunoglobulin Lambda Variable 4-3 |
| IGLV7-43 | Immunoglobulin Lambda Variable 7-43 |
| IGLV3-19 | Immunoglobulin Lambda Variable 3-19 |
| IGKV1D-16 | Immunoglobulin Kappa Variable 1D-16 |
| IGLV2-11 | Immunoglobulin Lambda Variable 2-11 |
| IGKV1-17 | Immunoglobulin Kappa Variable 1-17 |
| IGKV1D-13 | Immunoglobulin Kappa Variable 1D-13 |
| IGKV2-24 | Immunoglobulin Kappa Variable 2-24 |
| IGLV3-27 | Immunoglobulin Lambda Variable 3-27 |
| IGLV8-61 | Immunoglobulin Lambda Variable 8-61 |
| IGLV1-47 | Immunoglobulin Lambda Variable 1-47 |
| IGLV2-18 | Immunoglobulin Lambda Variable 2-18 |
| IGLV2-8 | Immunoglobulin Lambda Variable 2-8 |
| IGLV3-10 | Immunoglobulin Lambda Variable 3-10 |
| IGKV3-11 | Immunoglobulin Kappa Variable 3-11 |
| IGKV1-27 | Immunoglobulin Kappa Variable 1-27 |
| IGKV1D-8 | Immunoglobulin Kappa Variable 1D-8 |
| IGKV2-30 | Immunoglobulin Kappa Variable 2-30 |
| IGLV3-9 | Immunoglobulin Lambda Variable 3-9 (Gene/Pseudogene) |
| IGLV7-46 | Immunoglobulin Lambda Variable 7-46 (Gene/Pseudogene) |
| IGKV3D-15 | Immunoglobulin Kappa Variable 3D-15 (Gene/Pseudogene) |
| IGLV1-36 | Immunoglobulin Lambda Variable 1-36 |
| IGLV3-12 | Immunoglobulin Lambda Variable 3-12 |
| IGLV3-16 | Immunoglobulin Lambda Variable 3-16 |
| IGKV3-15 | Immunoglobulin Kappa Variable 3-15 |
| IGKV1D-33 | Immunoglobulin Kappa Variable 1D-33 |
| IGKV1-12 | Immunoglobulin Kappa Variable 1-12 |
| IGKV1-16 | Immunoglobulin Kappa Variable 1-16 |
| IGKV1-39 | Immunoglobulin Kappa Variable 1-39 (Gene/Pseudogene) |
| IGKV1-9 | Immunoglobulin Kappa Variable 1-9 |
| IGKV1D-17 | Immunoglobulin Kappa Variable 1D-17 |
| IGKV1D-43 | Immunoglobulin Kappa Variable 1D-43 |
| IGLV5-52 | Immunoglobulin Lambda Variable 5-52 |
| IGLV10-54 | Immunoglobulin Lambda Variable 10-54 |
| IGLV2-23 | Immunoglobulin Lambda Variable 2-23 |
| IGLV3-1 | Immunoglobulin Lambda Variable 3-1 |
| IGKV1D-39 | Immunoglobulin Kappa Variable 1D-39 |
| IGKV1-6 | Immunoglobulin Kappa Variable 1-6 |
| IGKV1-8 | Immunoglobulin Kappa Variable 1-8 |
| IGKV1D-12 | Immunoglobulin Kappa Variable 1D-12 |
| IGKV2-28 | Immunoglobulin Kappa Variable 2-28 |
| IGKV2D-26 | Immunoglobulin Kappa Variable 2D-26 |
| IGKV2D-30 | Immunoglobulin Kappa Variable 2D-30 |
| IGLV4-60 | Immunoglobulin Lambda Variable 4-60 |
| IGLV4-69 | Immunoglobulin Lambda Variable 4-69 |
| IGLV5-37 | Immunoglobulin Lambda Variable 5-37 |
| IGKV3D-20 | Immunoglobulin Kappa Variable 3D-20 |
| IGKV3D-7 | Immunoglobulin Kappa Variable 3D-7 |
| IGKV6-21 | Immunoglobulin Kappa Variable 6-21 (Non-Functional) |
| IGKV6D-21 | Immunoglobulin Kappa Variable 6D-21 (Non-Functional) |
| IGLV3-22 | Immunoglobulin Lambda Variable 3-22 (Gene/Pseudogene) |
| IGKV2D-40 | Immunoglobulin Kappa Variable 2D-40 |
| IGLV5-45 | Immunoglobulin Lambda Variable 5-45 |
| IGLV9-49 | Immunoglobulin Lambda Variable 9-49 |
| IAPP | Islet Amyloid Polypeptide |
| VNN1 | Vanin 1 |
| SMARCC2 | SWI/SNF Related, Matrix Associated, Actin Dependent Regulator Of Chromatin Subfamily C Member 2 |
| FMO3 | Flavin Containing Dimethylaniline Monoxygenase 3 |
| PALB2 | Partner And Localizer Of BRCA2 |
| FANCF | FA Complementation Group F |
| FANCB | FA Complementation Group B |
| TRBV6-5 | T Cell Receptor Beta Variable 6-5 |
| PSMD2 | Proteasome 26S Subunit, Non-ATPase 2 |
| LNPEP | Leucyl And Cystinyl Aminopeptidase |
| CLCA4 | Chloride Channel Accessory 4 |
| ACAN | Aggrecan |
| TRAV8-6 | T Cell Receptor Alpha Variable 8-6 |
| TRAJ3 | T Cell Receptor Alpha Joining 3 |
| HSPA2 | Heat Shock Protein Family A (Hsp70) Member 2 |
| TCF7L2 | Transcription Factor 7 Like 2 |
| MT-ND2 | Mitochondrially Encoded NADH:Ubiquinone Oxidoreductase Core Subunit 2 |
| NFASC | Neurofascin |
| STRA6 | Signaling Receptor And Transporter Of Retinol STRA6 |
| DYSF | Dysferlin |
| TRBJ1-1 | T Cell Receptor Beta Joining 1-1 |
| SLC25A24 | Solute Carrier Family 25 Member 24 |
| TBX19 | T-Box Transcription Factor 19 |
| ORC6 | Origin Recognition Complex Subunit 6 |
| CRELD1 | Cysteine Rich With EGF Like Domains 1 |
| MKRN3 | Makorin Ring Finger Protein 3 |
| IER3IP1 | Immediate Early Response 3 Interacting Protein 1 |
| TRAPPC11 | Trafficking Protein Particle Complex 11 |
| KDF1 | Keratinocyte Differentiation Factor 1 |
| ZNRD2 | Zinc Ribbon Domain Containing 2 |
| HSPA6 | Heat Shock Protein Family A (Hsp70) Member 6 |
| MID2 | Midline 2 |
| PSMD6 | Proteasome 26S Subunit, Non-ATPase 6 |
| THEM4 | Thioesterase Superfamily Member 4 |
| DHX8 | DEAH-Box Helicase 8 |
| TRAV12-3 | T Cell Receptor Alpha Variable 12-3 |
| TRAV17 | T Cell Receptor Alpha Variable 17 |
| NLRP2 | NLR Family Pyrin Domain Containing 2 |
| EPOR | Erythropoietin Receptor |
| ACTG1 | Actin Gamma 1 |
| LMO7 | LIM Domain 7 |
| RYR2 | Ryanodine Receptor 2 |
| CAV3 | Caveolin 3 |
| NCOA2 | Nuclear Receptor Coactivator 2 |
| SLC7A2 | Solute Carrier Family 7 Member 2 |
| ALPG | Alkaline Phosphatase, Germ Cell |
| SYNPO | Synaptopodin |
| NEXN | Nexilin F-Actin Binding Protein |
| PSMD11 | Proteasome 26S Subunit, Non-ATPase 11 |
| RAD21 | RAD21 Cohesin Complex Component |
| IGKV@ | Immunoglobulin Kappa Variable Cluster |
| IGLC2 | Immunoglobulin Lambda Constant 2 |
| IGHV1-24 | Immunoglobulin Heavy Variable 1-24 |
| AGBL5 | ATP/GTP Binding Protein Like 5 |
| PCYT1A | Phosphate Cytidylyltransferase 1, Choline, Alpha |
| GJB4 | Gap Junction Protein Beta 4 |
| VPS33B | VPS33B Late Endosome And Lysosome Associated |
| LRIG2 | Leucine Rich Repeats And Immunoglobulin Like Domains 2 |
| KTN1 | Kinectin 1 |
| CLEC11A | C-Type Lectin Domain Containing 11A |
| GBX2 | Gastrulation Brain Homeobox 2 |
| IFITM5 | Interferon Induced Transmembrane Protein 5 |
| TRAV20 | T Cell Receptor Alpha Variable 20 |
| TRAV13-2 | T Cell Receptor Alpha Variable 13-2 |
| TRAV13-1 | T Cell Receptor Alpha Variable 13-1 |
| TRAV26-1 | T Cell Receptor Alpha Variable 26-1 |
| TRAV39 | T Cell Receptor Alpha Variable 39 |
| TRAV8-3 | T Cell Receptor Alpha Variable 8-3 |
| TRAV10 | T Cell Receptor Alpha Variable 10 |
| TRAV16 | T Cell Receptor Alpha Variable 16 |
| TRAV23DV6 | T Cell Receptor Alpha Variable 23/Delta Variable 6 |
| TRAV25 | T Cell Receptor Alpha Variable 25 |
| TRAV26-2 | T Cell Receptor Alpha Variable 26-2 |
| TRAV6 | T Cell Receptor Alpha Variable 6 |
| TRAV8-2 | T Cell Receptor Alpha Variable 8-2 |
| TRAV1-2 | T Cell Receptor Alpha Variable 1-2 |
| TRAV3 | T Cell Receptor Alpha Variable 3 (Gene/Pseudogene) |
| TRAV30 | T Cell Receptor Alpha Variable 30 |
| TRAV4 | T Cell Receptor Alpha Variable 4 |
| TRAV41 | T Cell Receptor Alpha Variable 41 |
| TRAV8-1 | T Cell Receptor Alpha Variable 8-1 |
| TRAV1-1 | T Cell Receptor Alpha Variable 1-1 |
| TRAV12-1 | T Cell Receptor Alpha Variable 12-1 |
| TRAV34 | T Cell Receptor Alpha Variable 34 |
| TRAV36DV7 | T Cell Receptor Alpha Variable 36/Delta Variable 7 |
| TRAV5 | T Cell Receptor Alpha Variable 5 |
| TRAV7 | T Cell Receptor Alpha Variable 7 |
| TRAJ42 | T Cell Receptor Alpha Joining 42 |
| ASL | Argininosuccinate Lyase |
| STEAP4 | STEAP4 Metalloreductase |
| HIVEP2 | HIVEP Zinc Finger 2 |
| TRAPPC10 | Trafficking Protein Particle Complex 10 |
| KLHL2 | Kelch Like Family Member 2 |
| IL4I1 | Interleukin 4 Induced 1 |
| PIGM | Phosphatidylinositol Glycan Anchor Biosynthesis Class M |
| PSMA2 | Proteasome 20S Subunit Alpha 2 |
| CYP24A1 | Cytochrome P450 Family 24 Subfamily A Member 1 |
| MYRIP | Myosin VIIA And Rab Interacting Protein |
| LIN54 | Lin-54 DREAM MuvB Core Complex Component |
| UCA1 | Urothelial Cancer Associated 1 |
| LINC01554 | Long Intergenic Non-Protein Coding RNA 1554 |
| LINC01193 | Long Intergenic Non-Protein Coding RNA 1193 |
| SLC27A4 | Solute Carrier Family 27 Member 4 |
| ANKRD1 | Ankyrin Repeat Domain 1 |
| TRBV3-1 | T Cell Receptor Beta Variable 3-1 |
| FFAR3 | Free Fatty Acid Receptor 3 |
| CAPZA2 | Capping Actin Protein Of Muscle Z-Line Subunit Alpha 2 |
| CTSH | Cathepsin H |
| LPA | Lipoprotein(A) |
| CSPG4 | Chondroitin Sulfate Proteoglycan 4 |
| HTRA2 | HtrA Serine Peptidase 2 |
| KRT15 | Keratin 15 |
| LRRC2 | Leucine Rich Repeat Containing 2 |
| COPB1 | COPI Coat Complex Subunit Beta 1 |
| LST1 | Leukocyte Specific Transcript 1 |
| DDB2 | Damage Specific DNA Binding Protein 2 |
| TRBV16 | T Cell Receptor Beta Variable 16 (Gene/Pseudogene) |
| CUL5 | Cullin 5 |
| SOX4 | SRY-Box Transcription Factor 4 |
| TLK2 | Tousled Like Kinase 2 |
| CTNNA1 | Catenin Alpha 1 |
| CLDN5 | Claudin 5 |
| IFIT5 | Interferon Induced Protein With Tetratricopeptide Repeats 5 |
| EDA2R | Ectodysplasin A2 Receptor |
| CLEC2D | C-Type Lectin Domain Family 2 Member D |
| IGHV4-31 | Immunoglobulin Heavy Variable 4-31 |
| IGHJ1 | Immunoglobulin Heavy Joining 1 |
| IGHV2-70D | Immunoglobulin Heavy Variable 2-70D |
| IGHV1-69-2 | Immunoglobulin Heavy Variable 1-69-2 |
| IGHV1-69D | Immunoglobulin Heavy Variable 1-69D |
| IGHV3-64D | Immunoglobulin Heavy Variable 3-64D |
| GPR84 | G Protein-Coupled Receptor 84 |
| PLD3 | Phospholipase D Family Member 3 |
| CSPP1 | Centrosome And Spindle Pole Associated Protein 1 |
| SMTN | Smoothelin |
| TRBV18 | T Cell Receptor Beta Variable 18 |
| NMT2 | N-Myristoyltransferase 2 |
| ADARB2 | Adenosine Deaminase RNA Specific B2 (Inactive) |
| DNAJC3 | DnaJ Heat Shock Protein Family (Hsp40) Member C3 |
| ATF6 | Activating Transcription Factor 6 |
| ATP5PO | ATP Synthase Peripheral Stalk Subunit OSCP |
| MED29 | Mediator Complex Subunit 29 |
| MIRLET7C | MicroRNA Let-7c |
| BTN1A1 | Butyrophilin Subfamily 1 Member A1 |
| PELI1 | Pellino E3 Ubiquitin Protein Ligase 1 |
| HUWE1 | HECT, UBA And WWE Domain Containing E3 Ubiquitin Protein Ligase 1 |
| MDK | Midkine |
| INHBA | Inhibin Subunit Beta A |
| ADAM8 | ADAM Metallopeptidase Domain 8 |
| ARNTL | Aryl Hydrocarbon Receptor Nuclear Translocator Like |
| CCNE1 | Cyclin E1 |
| DUSP4 | Dual Specificity Phosphatase 4 |
| POSTN | Periostin |
| TRIM4 | Tripartite Motif Containing 4 |
| TRBD1 | T Cell Receptor Beta Diversity 1 |
| TRBJ1-2 | T Cell Receptor Beta Joining 1-2 |
| LYVE1 | Lymphatic Vessel Endothelial Hyaluronan Receptor 1 |
| TIAM1 | TIAM Rac1 Associated GEF 1 |
| SRCAP | Snf2 Related CREBBP Activator Protein |
| PLXNC1 | Plexin C1 |
| DBN1 | Drebrin 1 |
| PTPRO | Protein Tyrosine Phosphatase Receptor Type O |
| IGHV1-18 | Immunoglobulin Heavy Variable 1-18 |
| IGHV1-3 | Immunoglobulin Heavy Variable 1-3 |
| IGHV3-15 | Immunoglobulin Heavy Variable 3-15 |
| IGHV3-20 | Immunoglobulin Heavy Variable 3-20 |
| IGHV3-66 | Immunoglobulin Heavy Variable 3-66 |
| IGHV3-74 | Immunoglobulin Heavy Variable 3-74 |
| IGHV4-28 | Immunoglobulin Heavy Variable 4-28 |
| IGHV5-51 | Immunoglobulin Heavy Variable 5-51 |
| IGHV3-43 | Immunoglobulin Heavy Variable 3-43 |
| IGHV3-72 | Immunoglobulin Heavy Variable 3-72 |
| IGHV3-73 | Immunoglobulin Heavy Variable 3-73 |
| IGHV4-4 | Immunoglobulin Heavy Variable 4-4 |
| IGHV4-61 | Immunoglobulin Heavy Variable 4-61 |
| IGHV1-58 | Immunoglobulin Heavy Variable 1-58 |
| IGHV2-26 | Immunoglobulin Heavy Variable 2-26 |
| IGHV3-64 | Immunoglobulin Heavy Variable 3-64 |
| IGHV1-45 | Immunoglobulin Heavy Variable 1-45 |
| IGHV6-1 | Immunoglobulin Heavy Variable 6-1 |
| IGHD1-1 | Immunoglobulin Heavy Diversity 1-1 |
| IGHV5-10-1 | Immunoglobulin Heavy Variable 5-10-1 |
| IGHV7-4-1 | Immunoglobulin Heavy Variable 7-4-1 |
| PC | Pyruvate Carboxylase |
| CLCA1 | Chloride Channel Accessory 1 |
| FKBP5 | FKBP Prolyl Isomerase 5 |
| PRKAR1B | Protein Kinase CAMP-Dependent Type I Regulatory Subunit Beta |
| TAX1BP1 | Tax1 Binding Protein 1 |
| RASGRP4 | RAS Guanyl Releasing Protein 4 |
| LINC01194 | Long Intergenic Non-Protein Coding RNA 1194 |
| NCR3LG1 | Natural Killer Cell Cytotoxicity Receptor 3 Ligand 1 |
| BID | BH3 Interacting Domain Death Agonist |
| RNH1 | Ribonuclease/Angiogenin Inhibitor 1 |
| PLA2R1 | Phospholipase A2 Receptor 1 |
| LTBP3 | Latent Transforming Growth Factor Beta Binding Protein 3 |
| TRBV20-1 | T Cell Receptor Beta Variable 20-1 |
| TRBV4-1 | T Cell Receptor Beta Variable 4-1 |
| TRBV4-2 | T Cell Receptor Beta Variable 4-2 |
| TRBV12-4 | T Cell Receptor Beta Variable 12-4 |
| HMGB3 | High Mobility Group Box 3 |
| TCF7 | Transcription Factor 7 |
| NRG1 | Neuregulin 1 |
| OOEP | Oocyte Expressed Protein |
| GET1 | Guided Entry Of Tail-Anchored Proteins Factor 1 |
| MIR106A | MicroRNA 106a |
| PRINS | Psoriasis Associated Non-Protein Coding RNA Induced By Stress |
| PDXK | Pyridoxal Kinase |
| PFKL | Phosphofructokinase, Liver Type |
| UBR2 | Ubiquitin Protein Ligase E3 Component N-Recognin 2 |
| PLOD1 | Procollagen-Lysine,2-Oxoglutarate 5-Dioxygenase 1 |
| AHI1 | Abelson Helper Integration Site 1 |
| CCDC86 | Coiled-Coil Domain Containing 86 |
| PPP2R1B | Protein Phosphatase 2 Scaffold Subunit Abeta |
| NOC2L | NOC2 Like Nucleolar Associated Transcriptional Repressor |
| TRBV5-4 | T Cell Receptor Beta Variable 5-4 |
| TRBV28 | T Cell Receptor Beta Variable 28 |
| TRBV27 | T Cell Receptor Beta Variable 27 |
| TRBV30 | T Cell Receptor Beta Variable 30 (Gene/Pseudogene) |
| TRBV2 | T Cell Receptor Beta Variable 2 |
| TRBV25-1 | T Cell Receptor Beta Variable 25-1 |
| TRBV5-1 | T Cell Receptor Beta Variable 5-1 |
| TRBV6-1 | T Cell Receptor Beta Variable 6-1 |
| TRBV5-6 | T Cell Receptor Beta Variable 5-6 |
| TRBV9 | T Cell Receptor Beta Variable 9 |
| TRBV24-1 | T Cell Receptor Beta Variable 24-1 |
| TRBV29-1 | T Cell Receptor Beta Variable 29-1 |
| TRBV6-6 | T Cell Receptor Beta Variable 6-6 |
| TRBV6-8 | T Cell Receptor Beta Variable 6-8 |
| TRBV7-2 | T Cell Receptor Beta Variable 7-2 |
| TRBJ2-1 | T Cell Receptor Beta Joining 2-1 |
| TRBJ2-7 | T Cell Receptor Beta Joining 2-7 |
| TRBV10-1 | T Cell Receptor Beta Variable 10-1(Gene/Pseudogene) |
| TRBV10-2 | T Cell Receptor Beta Variable 10-2 |
| TRBV5-5 | T Cell Receptor Beta Variable 5-5 |
| TRBV6-4 | T Cell Receptor Beta Variable 6-4 |
| TRBV7-4 | T Cell Receptor Beta Variable 7-4 (Gene/Pseudogene) |
| TRBV7-6 | T Cell Receptor Beta Variable 7-6 |
| TRBV7-7 | T Cell Receptor Beta Variable 7-7 |
| TRBJ1-3 | T Cell Receptor Beta Joining 1-3 |
| TRBJ1-4 | T Cell Receptor Beta Joining 1-4 |
| TRBJ1-5 | T Cell Receptor Beta Joining 1-5 |
| TRBJ1-6 | T Cell Receptor Beta Joining 1-6 |
| TRBV10-3 | T Cell Receptor Beta Variable 10-3 |
| TRBV13 | T Cell Receptor Beta Variable 13 |
| TRBV14 | T Cell Receptor Beta Variable 14 |
| TRBV12-5 | T Cell Receptor Beta Variable 12-5 |
| ABCD3 | ATP Binding Cassette Subfamily D Member 3 |
| IGKJ1 | Immunoglobulin Kappa Joining 1 |
| ACTG2 | Actin Gamma 2, Smooth Muscle |
| TUBB4A | Tubulin Beta 4A Class IVa |
| DIS3 | DIS3 Homolog, Exosome Endoribonuclease And 3'-5' Exoribonuclease |
| NR3C2 | Nuclear Receptor Subfamily 3 Group C Member 2 |
| KCNH1 | Potassium Voltage-Gated Channel Subfamily H Member 1 |
| ATL1 | Atlastin GTPase 1 |
| CDC45 | Cell Division Cycle 45 |
| HSPA1B | Heat Shock Protein Family A (Hsp70) Member 1B |
| NLRP5 | NLR Family Pyrin Domain Containing 5 |
| EVPL | Envoplakin |
| NME1 | NME/NM23 Nucleoside Diphosphate Kinase 1 |
| PPP1R12A | Protein Phosphatase 1 Regulatory Subunit 12A |
| AP1S1 | Adaptor Related Protein Complex 1 Subunit Sigma 1 |
| LY75 | Lymphocyte Antigen 75 |
| PMVK | Phosphomevalonate Kinase |
| GOT2 | Glutamic-Oxaloacetic Transaminase 2 |
| AACS | Acetoacetyl-CoA Synthetase |
| VPS11 | VPS11 Core Subunit Of CORVET And HOPS Complexes |
| TMEM165 | Transmembrane Protein 165 |
| TOR1B | Torsin Family 1 Member B |
| TMEM199 | Transmembrane Protein 199 |
| LAMP5 | Lysosomal Associated Membrane Protein Family Member 5 |
| MT-RNR2 | Mitochondrially Encoded 16S RRNA |
| MT-TE | Mitochondrially Encoded TRNA-Glu (GAA/G) |
| IBD2 | Inflammatory Bowel Disease 2 |
| IBD3 | Inflammatory Bowel Disease 3 |
| IBD6 | Inflammatory Bowel Disease 6 |
| APPL2 | Adaptor Protein, Phosphotyrosine Interacting With PH Domain And Leucine Zipper 2 |
| GSK3A | Glycogen Synthase Kinase 3 Alpha |
| MUL1 | Mitochondrial E3 Ubiquitin Protein Ligase 1 |
| DEF6 | DEF6 Guanine Nucleotide Exchange Factor |
| RCAN1 | Regulator Of Calcineurin 1 |
| PILRB | Paired Immunoglobin Like Type 2 Receptor Beta |
| PIANP | PILR Alpha Associated Neural Protein |
| SKP1 | S-Phase Kinase Associated Protein 1 |
| POLA1 | DNA Polymerase Alpha 1, Catalytic Subunit |
| PDGFRL | Platelet Derived Growth Factor Receptor Like |
| GPIHBP1 | Glycosylphosphatidylinositol Anchored High Density Lipoprotein Binding Protein 1 |
| SPHK2 | Sphingosine Kinase 2 |
| KPNA3 | Karyopherin Subunit Alpha 3 |
| SIGLEC12 | Sialic Acid Binding Ig Like Lectin 12 (Gene/Pseudogene) |
| SIGLEC11 | Sialic Acid Binding Ig Like Lectin 11 |
| PTS | 6-Pyruvoyltetrahydropterin Synthase |
| ACKR4 | Atypical Chemokine Receptor 4 |
| LRP2 | LDL Receptor Related Protein 2 |
| CLDN7 | Claudin 7 |
| MLN | Motilin |
| RBL1 | RB Transcriptional Corepressor Like 1 |
| PLD1 | Phospholipase D1 |
| DSN1 | DSN1 Component Of MIS12 Kinetochore Complex |
| ASB14 | Ankyrin Repeat And SOCS Box Containing 14 |
| ATP2C1 | ATPase Secretory Pathway Ca2+ Transporting 1 |
| GUCY2C | Guanylate Cyclase 2C |
| EYS | Eyes Shut Homolog |
| EREG | Epiregulin |
| RNASET2 | Ribonuclease T2 |
| TRIM62 | Tripartite Motif Containing 62 |
| FZD4 | Frizzled Class Receptor 4 |
| HNF4A | Hepatocyte Nuclear Factor 4 Alpha |
| EPHX1 | Epoxide Hydrolase 1 |
| SSR4 | Signal Sequence Receptor Subunit 4 |
| IGLJ1 | Immunoglobulin Lambda Joining 1 |
| IGKV1-13 | Immunoglobulin Kappa Variable 1-13 (Gene/Pseudogene) |
| IGLV5-39 | Immunoglobulin Lambda Variable 5-39 |
| MIR141 | MicroRNA 141 |
| MAP2 | Microtubule Associated Protein 2 |
| NR2F2 | Nuclear Receptor Subfamily 2 Group F Member 2 |
| CRHR2 | Corticotropin Releasing Hormone Receptor 2 |
| FADS1 | Fatty Acid Desaturase 1 |
| EPHA3 | EPH Receptor A3 |
| FES | FES Proto-Oncogene, Tyrosine Kinase |
| SMURF2 | SMAD Specific E3 Ubiquitin Protein Ligase 2 |
| AXIN1 | Axin 1 |
| PLPP5 | Phospholipid Phosphatase 5 |
| PTPN4 | Protein Tyrosine Phosphatase Non-Receptor Type 4 |
| AQP1 | Aquaporin 1 (Colton Blood Group) |
| CCL23 | C-C Motif Chemokine Ligand 23 |
| CLDN6 | Claudin 6 |
| ELAVL4 | ELAV Like RNA Binding Protein 4 |
| TMEM79 | Transmembrane Protein 79 |
| DNAH8 | Dynein Axonemal Heavy Chain 8 |
| DCN | Decorin |
| MAPKAPK2 | MAPK Activated Protein Kinase 2 |
| DEPTOR | DEP Domain Containing MTOR Interacting Protein |
| FGF5 | Fibroblast Growth Factor 5 |
| ASGR1 | Asialoglycoprotein Receptor 1 |
| F13B | Coagulation Factor XIII B Chain |
| DDR1 | Discoidin Domain Receptor Tyrosine Kinase 1 |
| ADAMTS4 | ADAM Metallopeptidase With Thrombospondin Type 1 Motif 4 |
| UCHL1 | Ubiquitin C-Terminal Hydrolase L1 |
| TRDV2 | T Cell Receptor Delta Variable 2 |
| MYO5B | Myosin VB |
| GSTT1 | Glutathione S-Transferase Theta 1 |
| PMCH | Pro-Melanin Concentrating Hormone |
| STT3A | STT3 Oligosaccharyltransferase Complex Catalytic Subunit A |
| RPS28 | Ribosomal Protein S28 |
| HPR | Haptoglobin-Related Protein |
| ACTN1 | Actinin Alpha 1 |
| NCOR1 | Nuclear Receptor Corepressor 1 |
| MOBP | Myelin Associated Oligodendrocyte Basic Protein |
| GNB2 | G Protein Subunit Beta 2 |
| ABCG8 | ATP Binding Cassette Subfamily G Member 8 |
| UBA52 | Ubiquitin A-52 Residue Ribosomal Protein Fusion Product 1 |
| MKI67 | Marker Of Proliferation Ki-67 |
| TRDV1 | T Cell Receptor Delta Variable 1 |
| TRDV3 | T Cell Receptor Delta Variable 3 |
| CCL16 | C-C Motif Chemokine Ligand 16 |
| PCM1 | Pericentriolar Material 1 |
| CXCL3 | C-X-C Motif Chemokine Ligand 3 |
| PRDX3 | Peroxiredoxin 3 |
| PPP2R5D | Protein Phosphatase 2 Regulatory Subunit B'Delta |
| UGT1A1 | UDP Glucuronosyltransferase Family 1 Member A1 |
| SMN1 | Survival Of Motor Neuron 1, Telomeric |
| VIPAS39 | VPS33B Interacting Protein, Apical-Basolateral Polarity Regulator, Spe-39 Homolog |
| ABCA7 | ATP Binding Cassette Subfamily A Member 7 |
| POLR1D | RNA Polymerase I And III Subunit D |
| GRAP2 | GRB2 Related Adaptor Protein 2 |
| DARS2 | Aspartyl-TRNA Synthetase 2, Mitochondrial |
| GJD2 | Gap Junction Protein Delta 2 |
| ELAC2 | ElaC Ribonuclease Z 2 |
| ARFGAP3 | ADP Ribosylation Factor GTPase Activating Protein 3 |
| CTDSPL | CTD Small Phosphatase Like |
| UNC45B | Unc-45 Myosin Chaperone B |
| NAPB | NSF Attachment Protein Beta |
| CELA3B | Chymotrypsin Like Elastase 3B |
| IGDCC3 | Immunoglobulin Superfamily DCC Subclass Member 3 |
| PNPLA4 | Patatin Like Phospholipase Domain Containing 4 |
| CELA1 | Chymotrypsin Like Elastase 1 |
| TATDN1 | TatD DNase Domain Containing 1 |
| VILL | Villin Like |
| TMEM120A | Transmembrane Protein 120A |
| SOHLH1 | Spermatogenesis And Oogenesis Specific Basic Helix-Loop-Helix 1 |
| ARSL | Arylsulfatase L |
| SOHLH2 | Spermatogenesis And Oogenesis Specific Basic Helix-Loop-Helix 2 |
| PLPP7 | Phospholipid Phosphatase 7 (Inactive) |
| MIR491 | MicroRNA 491 |
| H3-2 | H3.2 Histone (Putative) |
| MT-TT | Mitochondrially Encoded TRNA-Thr (ACN) |
| HT | Hashimoto Thyroiditis |
| IBD4 | Inflammatory Bowel Disease 4 |
| IBD7 | Inflammatory Bowel Disease 7 |
| IBD8 | Inflammatory Bowel Disease 8 |
| IBD9 | Inflammatory Bowel Disease 9 |
| IBD15 | Inflammatory Bowel Disease-15 |
| IBD18 | Inflammatory Bowel Disease-18 |
| LPRS | Leprosy, Susceptibility To 1 |
| IBD11 | Inflammatory Bowel Disease 11 |
| IBD12 | Inflammatory Bowel Disease 12 |
| IBD16 | Inflammatory Bowel Disease-16 |
| IBD20 | Inflammatory Bowel Disease-20 |
| IBD21 | Inflammatory Bowel Disease-21 |
| IBD22 | Inflammatory Bowel Disease-22 |
| IBD23 | Inflammatory Bowel Disease-23 |
| IBD24 | Inflammatory Bowel Disease-24 |
| IBD25 | Inflammatory Bowel Disease-25 |
| IBD26 | Inflammatory Bowel Disease-26 |
| IBD27 | Inflammatory Bowel Disease-27 |
| INDX | Immunoneurologic Syndrome X-Linked, Of Wood, Black, And Norbury |
| ATPLS | Antiphospholipid Syndrome, Familial |
| EOE1 | Esophagitis, Eosinophilic |
| EOE2 | Esophagitis, Eosinophilic, 2 |
| AA1 | Alopecia Areata 1 |
| BCL11A | BAF Chromatin Remodeling Complex Subunit BCL11A |
| TYMP | Thymidine Phosphorylase |
| ITGAD | Integrin Subunit Alpha D |
| XYLT1 | Xylosyltransferase 1 |
| DPF2 | Double PHD Fingers 2 |
| IMMT | Inner Membrane Mitochondrial Protein |
| NBAS | NBAS Subunit Of NRZ Tethering Complex |
| VAV2 | Vav Guanine Nucleotide Exchange Factor 2 |
| RAB11FIP1 | RAB11 Family Interacting Protein 1 |
| SLC10A1 | Solute Carrier Family 10 Member 1 |
| DROSHA | Drosha Ribonuclease III |
| SCD | Stearoyl-CoA Desaturase |
| STMN1 | Stathmin 1 |
| GSDMD | Gasdermin D |
| VANGL1 | VANGL Planar Cell Polarity Protein 1 |
| RPL26 | Ribosomal Protein L26 |
| ERCC6L2 | ERCC Excision Repair 6 Like 2 |
| TAL2 | TAL BHLH Transcription Factor 2 |
| ELOVL4 | ELOVL Fatty Acid Elongase 4 |
| PNKP | Polynucleotide Kinase 3'-Phosphatase |
| COX5A | Cytochrome C Oxidase Subunit 5A |
| ADCYAP1 | Adenylate Cyclase Activating Polypeptide 1 |
| FGF9 | Fibroblast Growth Factor 9 |
| SERPINA7 | Serpin Family A Member 7 |
| ATAD1 | ATPase Family AAA Domain Containing 1 |
| IGSF3 | Immunoglobulin Superfamily Member 3 |
| SAMD9 | Sterile Alpha Motif Domain Containing 9 |
| PSMD9 | Proteasome 26S Subunit, Non-ATPase 9 |
| CBX5 | Chromobox 5 |
| DLX3 | Distal-Less Homeobox 3 |
| CNTN2 | Contactin 2 |
| OTUB1 | OTU Deubiquitinase, Ubiquitin Aldehyde Binding 1 |
| SLC2A4 | Solute Carrier Family 2 Member 4 |
| DYNC1H1 | Dynein Cytoplasmic 1 Heavy Chain 1 |
| RAB27B | RAB27B, Member RAS Oncogene Family |
| SNRNP200 | Small Nuclear Ribonucleoprotein U5 Subunit 200 |
| SLC30A1 | Solute Carrier Family 30 Member 1 |
| XPO5 | Exportin 5 |
| DNAH9 | Dynein Axonemal Heavy Chain 9 |
| BRWD1 | Bromodomain And WD Repeat Domain Containing 1 |
| JCHAIN | Joining Chain Of Multimeric IgA And IgM |
| RAB8A | RAB8A, Member RAS Oncogene Family |
| RAB5A | RAB5A, Member RAS Oncogene Family |
| LY6E | Lymphocyte Antigen 6 Family Member E |
| FLNC | Filamin C |
| KEL | Kell Metallo-Endopeptidase (Kell Blood Group) |
| BNC2 | Basonuclin 2 |
| PSCA | Prostate Stem Cell Antigen |
| CSH1 | Chorionic Somatomammotropin Hormone 1 |
| TDGF1 | Teratocarcinoma-Derived Growth Factor 1 |
| MIR183 | MicroRNA 183 |
| ITGA1 | Integrin Subunit Alpha 1 |
| CLPS | Colipase |
| MTRNR2L8 | MT-RNR2 Like 8 |
| PAX3 | Paired Box 3 |
| TRAV35 | T Cell Receptor Alpha Variable 35 |
| DIAPH1 | Diaphanous Related Formin 1 |
| PLCE1 | Phospholipase C Epsilon 1 |
| CLTCL1 | Clathrin Heavy Chain Like 1 |
| CTAG1A | Cancer/Testis Antigen 1A |
| ZIC2 | Zic Family Member 2 |
| BMPR1A | Bone Morphogenetic Protein Receptor Type 1A |
| PEA15 | Proliferation And Apoptosis Adaptor Protein 15 |
| IGLP1 | Immune Response To Synthetic Polypeptides 1 |
| AK5 | Adenylate Kinase 5 |
| XRCC2 | X-Ray Repair Cross Complementing 2 |
| TLX1 | T Cell Leukemia Homeobox 1 |
| OXA1L | OXA1L Mitochondrial Inner Membrane Protein |
| CHRNG | Cholinergic Receptor Nicotinic Gamma Subunit |
| RFT1 | RFT1 Homolog |
| SUN2 | Sad1 And UNC84 Domain Containing 2 |
| GGT2 | Gamma-Glutamyltransferase 2 |
| TRGV3 | T Cell Receptor Gamma Variable 3 |
| SERPINB7 | Serpin Family B Member 7 |
| PRDX4 | Peroxiredoxin 4 |
| KLK2 | Kallikrein Related Peptidase 2 |
| CALCR | Calcitonin Receptor |
| MFHAS1 | Malignant Fibrous Histiocytoma Amplified Sequence 1 |
| LNX1 | Ligand Of Numb-Protein X 1 |
| HAS1 | Hyaluronan Synthase 1 |
| AKR1A1 | Aldo-Keto Reductase Family 1 Member A1 |
| MPEG1 | Macrophage Expressed 1 |
| IGLC7 | Immunoglobulin Lambda Constant 7 |
| SERPINA6 | Serpin Family A Member 6 |
| AFAP1 | Actin Filament Associated Protein 1 |
| H2AC21 | H2A Clustered Histone 21 |
| UBN1 | Ubinuclein 1 |
| UBA7 | Ubiquitin Like Modifier Activating Enzyme 7 |
| SPON2 | Spondin 2 |
| EPB41 | Erythrocyte Membrane Protein Band 4.1 |
| ABHD16A | Abhydrolase Domain Containing 16A |
| PANX1 | Pannexin 1 |
| TDP2 | Tyrosyl-DNA Phosphodiesterase 2 |
| RANBP1 | RAN Binding Protein 1 |
| BCL2L2 | BCL2 Like 2 |
| TCERG1 | Transcription Elongation Regulator 1 |
| HMGA2 | High Mobility Group AT-Hook 2 |
| PDPN | Podoplanin |
| FGF17 | Fibroblast Growth Factor 17 |
| CLDN9 | Claudin 9 |
| ARPC2 | Actin Related Protein 2/3 Complex Subunit 2 |
| COCH | Cochlin |
| RNF6 | Ring Finger Protein 6 |
| AKAP12 | A-Kinase Anchoring Protein 12 |
| ILF2 | Interleukin Enhancer Binding Factor 2 |
| RPS6KA3 | Ribosomal Protein S6 Kinase A3 |
| TRIP4 | Thyroid Hormone Receptor Interactor 4 |
| RAPGEF1 | Rap Guanine Nucleotide Exchange Factor 1 |
| TFG | Trafficking From ER To Golgi Regulator |
| DSC2 | Desmocollin 2 |
| GHITM | Growth Hormone Inducible Transmembrane Protein |
| NPEPPS | Aminopeptidase Puromycin Sensitive |
| APOA4 | Apolipoprotein A4 |
| ADPRM | ADP-Ribose/CDP-Alcohol Diphosphatase, Manganese Dependent |
| SUGT1 | SGT1 Homolog, MIS12 Kinetochore Complex Assembly Cochaperone |
| SCIMP | SLP Adaptor And CSK Interacting Membrane Protein |
| DNAJA2 | DnaJ Heat Shock Protein Family (Hsp40) Member A2 |
| ARPC3 | Actin Related Protein 2/3 Complex Subunit 3 |
| TDO2 | Tryptophan 2,3-Dioxygenase |
| PRMT5 | Protein Arginine Methyltransferase 5 |
| AGR2 | Anterior Gradient 2, Protein Disulphide Isomerase Family Member |
| ABCD4 | ATP Binding Cassette Subfamily D Member 4 |
| MLLT10 | MLLT10 Histone Lysine Methyltransferase DOT1L Cofactor |
| PIGL | Phosphatidylinositol Glycan Anchor Biosynthesis Class L |
| TSR2 | TSR2 Ribosome Maturation Factor |
| CORIN | Corin, Serine Peptidase |
| STOX1 | Storkhead Box 1 |
| C1GALT1C1 | C1GALT1 Specific Chaperone 1 |
| FZR1 | Fizzy And Cell Division Cycle 20 Related 1 |
| CASP14 | Caspase 14 |
| IGHV7-81 | Immunoglobulin Heavy Variable 7-81 (Non-Functional) |
| IGHV3-38 | Immunoglobulin Heavy Variable 3-38 (Non-Functional) |
| IGHV3-16 | Immunoglobulin Heavy Variable 3-16 (Non-Functional) |
| IGKV2D-24 | Immunoglobulin Kappa Variable 2D-24 (Non-Functional) |
| IGHV3-35 | Immunoglobulin Heavy Variable 3-35 (Non-Functional) |
| COL4A1 | Collagen Type IV Alpha 1 Chain |
| LEMD3 | LEM Domain Containing 3 |
| HOXA13 | Homeobox A13 |
| PON2 | Paraoxonase 2 |
| SHBG | Sex Hormone Binding Globulin |
| LGI1 | Leucine Rich Glioma Inactivated 1 |
| GCM2 | Glial Cells Missing Transcription Factor 2 |
| SIL1 | SIL1 Nucleotide Exchange Factor |
| PIR | Pirin |
| PAM16 | Presequence Translocase Associated Motor 16 |
| KCNRG | Potassium Channel Regulator |
| PPIP5K1 | Diphosphoinositol Pentakisphosphate Kinase 1 |
| ARMS2 | Age-Related Maculopathy Susceptibility 2 |
| MIR10A | MicroRNA 10a |
| RN7SL1 | RNA Component Of Signal Recognition Particle 7SL1 |
| ZFAT-AS1 | ZFAT Antisense RNA 1 |
| TRBJ2-3 | T Cell Receptor Beta Joining 2-3 |
| TRBV4-3 | T Cell Receptor Beta Variable 4-3 |
| TRBV6-3 | T Cell Receptor Beta Variable 6-3 |
| POU1F1 | POU Class 1 Homeobox 1 |
| TNRC6C | Trinucleotide Repeat Containing Adaptor 6C |
| RAB23 | RAB23, Member RAS Oncogene Family |
| CAP2 | Cyclase Associated Actin Cytoskeleton Regulatory Protein 2 |
| FOXH1 | Forkhead Box H1 |
| EXOSC10 | Exosome Component 10 |
| BAZ1A | Bromodomain Adjacent To Zinc Finger Domain 1A |
| AMPD1 | Adenosine Monophosphate Deaminase 1 |
| CGB5 | Chorionic Gonadotropin Subunit Beta 5 |
| DNAJC6 | DnaJ Heat Shock Protein Family (Hsp40) Member C6 |
| TPSAB1 | Tryptase Alpha/Beta 1 |
| TRBJ2-2 | T Cell Receptor Beta Joining 2-2 |
| TRBJ2-4 | T Cell Receptor Beta Joining 2-4 |
| TRBJ2-5 | T Cell Receptor Beta Joining 2-5 |
| TRBJ2-6 | T Cell Receptor Beta Joining 2-6 |
| TRBV6-2 | T Cell Receptor Beta Variable 6-2 (Gene/Pseudogene) |
| TRBV6-9 | T Cell Receptor Beta Variable 6-9 |
| TRBV7-8 | T Cell Receptor Beta Variable 7-8 |
| TRBV5-8 | T Cell Receptor Beta Variable 5-8 |
| GNB1L | G Protein Subunit Beta 1 Like |
| IREB2 | Iron Responsive Element Binding Protein 2 |
| MAP3K4 | Mitogen-Activated Protein Kinase Kinase Kinase 4 |
| TIMP3 | TIMP Metallopeptidase Inhibitor 3 |
| PDLIM2 | PDZ And LIM Domain 2 |
| REST | RE1 Silencing Transcription Factor |
| KPNB1 | Karyopherin Subunit Beta 1 |
| PSMD10 | Proteasome 26S Subunit, Non-ATPase 10 |
| PRDX6 | Peroxiredoxin 6 |
| LSM2 | LSM2 Homolog, U6 Small Nuclear RNA And MRNA Degradation Associated |
| FLI1 | Fli-1 Proto-Oncogene, ETS Transcription Factor |
| NBR1 | NBR1 Autophagy Cargo Receptor |
| NLRP4 | NLR Family Pyrin Domain Containing 4 |
| STK11 | Serine/Threonine Kinase 11 |
| ARL11 | ADP Ribosylation Factor Like GTPase 11 |
| ZNF622 | Zinc Finger Protein 622 |
| SLC26A4 | Solute Carrier Family 26 Member 4 |
| ATF4 | Activating Transcription Factor 4 |
| RTN4R | Reticulon 4 Receptor |
| CEP152 | Centrosomal Protein 152 |
| PLK1 | Polo Like Kinase 1 |
| SYP | Synaptophysin |
| SCRIB | Scribble Planar Cell Polarity Protein |
| GLA | Galactosidase Alpha |
| BDKRB2 | Bradykinin Receptor B2 |
| GBP3 | Guanylate Binding Protein 3 |
| CCL3L3 | C-C Motif Chemokine Ligand 3 Like 3 |
| LCP1 | Lymphocyte Cytosolic Protein 1 |
| GREM1 | Gremlin 1, DAN Family BMP Antagonist |
| GLIPR1 | GLI Pathogenesis Related 1 |
| RIT1 | Ras Like Without CAAX 1 |
| PIK3C3 | Phosphatidylinositol 3-Kinase Catalytic Subunit Type 3 |
| PGRMC1 | Progesterone Receptor Membrane Component 1 |
| EFNA5 | Ephrin A5 |
| ITGA7 | Integrin Subunit Alpha 7 |
| KCTD1 | Potassium Channel Tetramerization Domain Containing 1 |
| SEMA3D | Semaphorin 3D |
| IGHV3-43D | Immunoglobulin Heavy Variable 3-43D |
| IGHV4-30-4 | Immunoglobulin Heavy Variable 4-30-4 |
| IGHV3-30-3 | Immunoglobulin Heavy Variable 3-30-3 |
| IGHV3-30-5 | Immunoglobulin Heavy Variable 3-30-5 |
| HERC1 | HECT And RLD Domain Containing E3 Ubiquitin Protein Ligase Family Member 1 |
| UBE2W | Ubiquitin Conjugating Enzyme E2 W |
| PLA2G2F | Phospholipase A2 Group IIF |
| PAX2 | Paired Box 2 |
| KIF1B | Kinesin Family Member 1B |
| LIN28B | Lin-28 Homolog B |
| DEFA1B | Defensin Alpha 1B |
| PPP1CA | Protein Phosphatase 1 Catalytic Subunit Alpha |
| HEPHL1 | Hephaestin Like 1 |
| SUCO | SUN Domain Containing Ossification Factor |
| SLC22A8 | Solute Carrier Family 22 Member 8 |
| ACTL6A | Actin Like 6A |
| FBXO9 | F-Box Protein 9 |
| BAP1 | BRCA1 Associated Protein 1 |
| CDAN1 | Codanin 1 |
| CFC1 | Cripto, FRL-1, Cryptic Family 1 |
| C15orf41 | Chromosome 15 Open Reading Frame 41 |
| KAT2B | Lysine Acetyltransferase 2B |
| HCFC1 | Host Cell Factor C1 |
| MEIS2 | Meis Homeobox 2 |
| UFL1 | UFM1 Specific Ligase 1 |
| EIF5A | Eukaryotic Translation Initiation Factor 5A |
| NSD3 | Nuclear Receptor Binding SET Domain Protein 3 |
| TRGV9 | T Cell Receptor Gamma Variable 9 |
| FCRL5 | Fc Receptor Like 5 |
| WWOX | WW Domain Containing Oxidoreductase |
| PFN1 | Profilin 1 |
| GPR55 | G Protein-Coupled Receptor 55 |
| DTX3L | Deltex E3 Ubiquitin Ligase 3L |
| CHD4 | Chromodomain Helicase DNA Binding Protein 4 |
| PAWR | Pro-Apoptotic WT1 Regulator |
| LTC4S | Leukotriene C4 Synthase |
| IGKV3-7 | Immunoglobulin Kappa Variable 3-7 (Non-Functional) |
| IGLV5-48 | Immunoglobulin Lambda Variable 5-48 (Non-Functional) |
| IGLV1-50 | Immunoglobulin Lambda Variable 1-50 (Non-Functional) |
| IGKV1-37 | Immunoglobulin Kappa Variable 1-37 (Non-Functional) |
| IGKV1D-42 | Immunoglobulin Kappa Variable 1D-42 (Non-Functional) |
| IGKV6D-41 | Immunoglobulin Kappa Variable 6D-41 (Non-Functional) |
| IGLV11-55 | Immunoglobulin Lambda Variable 11-55 (Non-Functional) |
| IGHV1-8 | Immunoglobulin Heavy Variable 1-8 |
| IGLV3-32 | Immunoglobulin Lambda Variable 3-32 (Non-Functional) |
| IGLV2-33 | Immunoglobulin Lambda Variable 2-33 (Non-Functional) |
| IGKV1D-37 | Immunoglobulin Kappa Variable 1D-37 (Non-Functional) |
| WNT3 | Wnt Family Member 3 |
| DGAT1 | Diacylglycerol O-Acyltransferase 1 |
| ATP6V0E2 | ATPase H+ Transporting V0 Subunit E2 |
| PSMD8 | Proteasome 26S Subunit, Non-ATPase 8 |
| PHYH | Phytanoyl-CoA 2-Hydroxylase |
| PEX7 | Peroxisomal Biogenesis Factor 7 |
| ERCC8 | ERCC Excision Repair 8, CSA Ubiquitin Ligase Complex Subunit |
| RANBP9 | RAN Binding Protein 9 |
| TMPRSS2 | Transmembrane Serine Protease 2 |
| MAML1 | Mastermind Like Transcriptional Coactivator 1 |
| NKX2-3 | NK2 Homeobox 3 |
| EDN3 | Endothelin 3 |
| SLCO2A1 | Solute Carrier Organic Anion Transporter Family Member 2A1 |
| NAA10 | N-Alpha-Acetyltransferase 10, NatA Catalytic Subunit |
| TCTN3 | Tectonic Family Member 3 |
| RAB3GAP2 | RAB3 GTPase Activating Non-Catalytic Protein Subunit 2 |
| KANSL1 | KAT8 Regulatory NSL Complex Subunit 1 |
| ATL3 | Atlastin GTPase 3 |
| SRY | Sex Determining Region Y |
| INF2 | Inverted Formin, FH2 And WH2 Domain Containing |
| CD53 | CD53 Molecule |
| NT5C | 5', 3'-Nucleotidase, Cytosolic |
| SLC41A1 | Solute Carrier Family 41 Member 1 |
| RAB3IL1 | RAB3A Interacting Protein Like 1 |
| SLC41A2 | Solute Carrier Family 41 Member 2 |
| ARFGAP2 | ADP Ribosylation Factor GTPase Activating Protein 2 |
| MTUS2 | Microtubule Associated Scaffold Protein 2 |
| KRTCAP2 | Keratinocyte Associated Protein 2 |
| TMEM185B | Transmembrane Protein 185B |
| MEG8 | Maternally Expressed 8, Small Nucleolar RNA Host Gene |
| MIR18B | MicroRNA 18b |
| LINC00426 | Long Intergenic Non-Protein Coding RNA 426 |
| CXCR2P1 | C-X-C Motif Chemokine Receptor 2 Pseudogene 1 |
| LINC02384 | Long Intergenic Non-Protein Coding RNA 2384 |
| CCR5AS | CCR5 Antisense RNA |
| ENSG00000240350 |  |
| NDUFA9 | NADH:Ubiquinone Oxidoreductase Subunit A9 |
| RPS3A | Ribosomal Protein S3A |
| FGFR1OP | FGFR1 Oncogene Partner |
| C1D | C1D Nuclear Receptor Corepressor |
| TIMM13 | Translocase Of Inner Mitochondrial Membrane 13 |
| RHO | Rhodopsin |
| TRGV5 | T Cell Receptor Gamma Variable 5 |
| NUTM1 | NUT Midline Carcinoma Family Member 1 |
| GLS | Glutaminase |
| MAN2C1 | Mannosidase Alpha Class 2C Member 1 |
| IL11RA | Interleukin 11 Receptor Subunit Alpha |
| TP73 | Tumor Protein P73 |
| KRT20 | Keratin 20 |
| PSORS1C1 | Psoriasis Susceptibility 1 Candidate 1 |
| FGF22 | Fibroblast Growth Factor 22 |
| CAPZA1 | Capping Actin Protein Of Muscle Z-Line Subunit Alpha 1 |
| MIR30B | MicroRNA 30b |
| SDCBP | Syndecan Binding Protein |
| TRGV2 | T Cell Receptor Gamma Variable 2 |
| TRGV4 | T Cell Receptor Gamma Variable 4 |
| TRGV8 | T Cell Receptor Gamma Variable 8 |
| GH2 | Growth Hormone 2 |
| SKAP2 | Src Kinase Associated Phosphoprotein 2 |
| STX7 | Syntaxin 7 |
| MOV10 | Mov10 RISC Complex RNA Helicase |
| TARDBP | TAR DNA Binding Protein |
| PLA2G4C | Phospholipase A2 Group IVC |
| PRKAA1 | Protein Kinase AMP-Activated Catalytic Subunit Alpha 1 |
| TRAJ18 | T Cell Receptor Alpha Joining 18 |
| GGH | Gamma-Glutamyl Hydrolase |
| CCNG1 | Cyclin G1 |
| SLC15A3 | Solute Carrier Family 15 Member 3 |
| CMTM4 | CKLF Like MARVEL Transmembrane Domain Containing 4 |
| SSTR2 | Somatostatin Receptor 2 |
| MUC6 | Mucin 6, Oligomeric Mucus/Gel-Forming |
| STS | Steroid Sulfatase |
| PI4KA | Phosphatidylinositol 4-Kinase Alpha |
| MNDA | Myeloid Cell Nuclear Differentiation Antigen |
| TBXT | T-Box Transcription Factor T |
| PPP2R1A | Protein Phosphatase 2 Scaffold Subunit Aalpha |
| KIFAP3 | Kinesin Associated Protein 3 |
| CASZ1 | Castor Zinc Finger 1 |
| FOSL2 | FOS Like 2, AP-1 Transcription Factor Subunit |
| PRKRA | Protein Activator Of Interferon Induced Protein Kinase EIF2AK2 |
| KCNMA1 | Potassium Calcium-Activated Channel Subfamily M Alpha 1 |
| DNM1L | Dynamin 1 Like |
| KCNH2 | Potassium Voltage-Gated Channel Subfamily H Member 2 |
| PROM1 | Prominin 1 |
| PPP3R2 | Protein Phosphatase 3 Regulatory Subunit B, Beta |
| CYP1B1 | Cytochrome P450 Family 1 Subfamily B Member 1 |
| AHNAK | AHNAK Nucleoprotein |
| CAMK2D | Calcium/Calmodulin Dependent Protein Kinase II Delta |
| CST2 | Cystatin SA |
| MIR100 | MicroRNA 100 |
| DBH | Dopamine Beta-Hydroxylase |
| MAD2L2 | Mitotic Arrest Deficient 2 Like 2 |
| MMP21 | Matrix Metallopeptidase 21 |
| RACGAP1 | Rac GTPase Activating Protein 1 |
| PSPH | Phosphoserine Phosphatase |
| CAPN5 | Calpain 5 |
| EPM2A | EPM2A Glucan Phosphatase, Laforin |
| SH3PXD2B | SH3 And PX Domains 2B |
| SUMO4 | Small Ubiquitin Like Modifier 4 |
| GPC4 | Glypican 4 |
| FBXW7 | F-Box And WD Repeat Domain Containing 7 |
| FAP | Fibroblast Activation Protein Alpha |
| MXI1 | MAX Interactor 1, Dimerization Protein |
| C1GALT1 | Core 1 Synthase, Glycoprotein-N-Acetylgalactosamine 3-Beta-Galactosyltransferase 1 |
| CCL15 | C-C Motif Chemokine Ligand 15 |
| STX17 | Syntaxin 17 |
| MYO18A | Myosin XVIIIA |
| KLRC3 | Killer Cell Lectin Like Receptor C3 |
| CUL3 | Cullin 3 |
| TAS2R38 | Taste 2 Receptor Member 38 |
| BGN | Biglycan |
| LTBP1 | Latent Transforming Growth Factor Beta Binding Protein 1 |
| MAP3K11 | Mitogen-Activated Protein Kinase Kinase Kinase 11 |
| SLC22A6 | Solute Carrier Family 22 Member 6 |
| ELMO2 | Engulfment And Cell Motility 2 |
| EPHB6 | EPH Receptor B6 |
| GDF1 | Growth Differentiation Factor 1 |
| DYNC1LI1 | Dynein Cytoplasmic 1 Light Intermediate Chain 1 |
| PSMF1 | Proteasome Inhibitor Subunit 1 |
| ADGRE1 | Adhesion G Protein-Coupled Receptor E1 |
| ACAT1 | Acetyl-CoA Acetyltransferase 1 |
| RPL18 | Ribosomal Protein L18 |
| DLK1 | Delta Like Non-Canonical Notch Ligand 1 |
| EXT1 | Exostosin Glycosyltransferase 1 |
| SLC25A15 | Solute Carrier Family 25 Member 15 |
| KIF1A | Kinesin Family Member 1A |
| MLX | MAX Dimerization Protein MLX |
| EBP | EBP Cholestenol Delta-Isomerase |
| HSD3B7 | Hydroxy-Delta-5-Steroid Dehydrogenase, 3 Beta- And Steroid Delta-Isomerase 7 |
| CHST14 | Carbohydrate Sulfotransferase 14 |
| ABCA12 | ATP Binding Cassette Subfamily A Member 12 |
| KAT6B | Lysine Acetyltransferase 6B |
| SLC52A3 | Solute Carrier Family 52 Member 3 |
| CLIP2 | CAP-Gly Domain Containing Linker Protein 2 |
| CHAMP1 | Chromosome Alignment Maintaining Phosphoprotein 1 |
| ZNF750 | Zinc Finger Protein 750 |
| LIPN | Lipase Family Member N |
| TGDS | TDP-Glucose 4,6-Dehydratase |
| HACD1 | 3-Hydroxyacyl-CoA Dehydratase 1 |
| MGME1 | Mitochondrial Genome Maintenance Exonuclease 1 |
| WASHC5 | WASH Complex Subunit 5 |
| AK3 | Adenylate Kinase 3 |
| AK4 | Adenylate Kinase 4 |
| CCNT2 | Cyclin T2 |
| POU3F1 | POU Class 3 Homeobox 1 |
| AK9 | Adenylate Kinase 9 |
| NPS | Neuropeptide S |
| TUG1 | Taurine Up-Regulated 1 |
| MIR133B | MicroRNA 133b |
| ZFP36L1 | ZFP36 Ring Finger Protein Like 1 |
| TRD | T Cell Receptor Delta Locus |
| ATP5IF1 | ATP Synthase Inhibitory Factor Subunit 1 |
| TNFRSF10D | TNF Receptor Superfamily Member 10d |
| P2RX4 | Purinergic Receptor P2X 4 |
| PACSIN2 | Protein Kinase C And Casein Kinase Substrate In Neurons 2 |
| CCT8 | Chaperonin Containing TCP1 Subunit 8 |
| SIRPB1 | Signal Regulatory Protein Beta 1 |
| TRIM37 | Tripartite Motif Containing 37 |
| GRHPR | Glyoxylate And Hydroxypyruvate Reductase |
| MAGI2 | Membrane Associated Guanylate Kinase, WW And PDZ Domain Containing 2 |
| ARID2 | AT-Rich Interaction Domain 2 |
| VPS13A | Vacuolar Protein Sorting 13 Homolog A |
| ANKRD11 | Ankyrin Repeat Domain 11 |
| CARM1 | Coactivator Associated Arginine Methyltransferase 1 |
| RPL27 | Ribosomal Protein L27 |
| MYLIP | Myosin Regulatory Light Chain Interacting Protein |
| KSR1 | Kinase Suppressor Of Ras 1 |
| SCARB1 | Scavenger Receptor Class B Member 1 |
| ZC3H12D | Zinc Finger CCCH-Type Containing 12D |
| MYO1C | Myosin IC |
| UBE2D2 | Ubiquitin Conjugating Enzyme E2 D2 |
| DEFB4B | Defensin Beta 4B |
| FRS2 | Fibroblast Growth Factor Receptor Substrate 2 |
| RNF19B | Ring Finger Protein 19B |
| SLURP2 | Secreted LY6/PLAUR Domain Containing 2 |
| UCP2 | Uncoupling Protein 2 |
| ATP6V1H | ATPase H+ Transporting V1 Subunit H |
| VKORC1 | Vitamin K Epoxide Reductase Complex Subunit 1 |
| LPAR6 | Lysophosphatidic Acid Receptor 6 |
| OTX2 | Orthodenticle Homeobox 2 |
| PHOX2B | Paired Like Homeobox 2B |
| LIPH | Lipase H |
| UACA | Uveal Autoantigen With Coiled-Coil Domains And Ankyrin Repeats |
| SPPL2A | Signal Peptide Peptidase Like 2A |
| PSAT1 | Phosphoserine Aminotransferase 1 |
| MCHR1 | Melanin Concentrating Hormone Receptor 1 |
| ECI2 | Enoyl-CoA Delta Isomerase 2 |
| COBL | Cordon-Bleu WH2 Repeat Protein |
| NUDT15 | Nudix Hydrolase 15 |
| TREX2 | Three Prime Repair Exonuclease 2 |
| TLDC2 | TBC/LysM-Associated Domain Containing 2 |
| NCF1C | Neutrophil Cytosolic Factor 1C Pseudogene |
| MIR135A1 | MicroRNA 135a-1 |
| NCF1B | Neutrophil Cytosolic Factor 1B Pseudogene |
| NCF4-AS1 | NCF4 Antisense RNA 1 |
| TRDJ1 | T Cell Receptor Delta Joining 1 |
| TRDD1 | T Cell Receptor Delta Diversity 1 |
| KLKB1 | Kallikrein B1 |
| HSPA1L | Heat Shock Protein Family A (Hsp70) Member 1 Like |
| RPS15A | Ribosomal Protein S15a |
| PACS2 | Phosphofurin Acidic Cluster Sorting Protein 2 |
| FOLR2 | Folate Receptor Beta |
| ZRANB3 | Zinc Finger RANBP2-Type Containing 3 |
| LOC108167311 | Proximal ADA Alu-Mediated Recombination Region |
| LOC108167312 | Distal ADA Alu-Mediated Recombination Region |
| GABARAPL1 | GABA Type A Receptor Associated Protein Like 1 |
| NEU2 | Neuraminidase 2 |
| GTF3A | General Transcription Factor IIIA |
| MCCC1 | Methylcrotonoyl-CoA Carboxylase 1 |
| OLFM4 | Olfactomedin 4 |
| BLZF1 | Basic Leucine Zipper Nuclear Factor 1 |
| OMP | Olfactory Marker Protein |
| SPEF2 | Sperm Flagellar 2 |
| CLDN12 | Claudin 12 |
| CD37 | CD37 Molecule |
| HCFC2 | Host Cell Factor C2 |
| IER3 | Immediate Early Response 3 |
| FASN | Fatty Acid Synthase |
| PSME4 | Proteasome Activator Subunit 4 |
| VMA21 | Vacuolar ATPase Assembly Factor VMA21 |
| MIR26A1 | MicroRNA 26a-1 |
| MIR155HG | MIR155 Host Gene |
| DEFB131A | Defensin Beta 131A |
| LGMN | Legumain |
| GNG10 | G Protein Subunit Gamma 10 |
| COMMD3 | COMM Domain Containing 3 |
| RAB10 | RAB10, Member RAS Oncogene Family |
| PLXNA1 | Plexin A1 |
| RECK | Reversion Inducing Cysteine Rich Protein With Kazal Motifs |
| MRC2 | Mannose Receptor C Type 2 |
| SCN10A | Sodium Voltage-Gated Channel Alpha Subunit 10 |
| SGCG | Sarcoglycan Gamma |
| SCN11A | Sodium Voltage-Gated Channel Alpha Subunit 11 |
| RFWD3 | Ring Finger And WD Repeat Domain 3 |
| DNAJC21 | DnaJ Heat Shock Protein Family (Hsp40) Member C21 |
| BCL6B | BCL6B Transcription Repressor |
| SPRED2 | Sprouty Related EVH1 Domain Containing 2 |
| KRT71 | Keratin 71 |
| AGBL3 | ATP/GTP Binding Protein Like 3 |
| RPL35 | Ribosomal Protein L35 |
| SCGB3A2 | Secretoglobin Family 3A Member 2 |
| SLC15A1 | Solute Carrier Family 15 Member 1 |
| SLC40A1 | Solute Carrier Family 40 Member 1 |
| IL20RB | Interleukin 20 Receptor Subunit Beta |
| CGB3 | Chorionic Gonadotropin Subunit Beta 3 |
| PRKAA2 | Protein Kinase AMP-Activated Catalytic Subunit Alpha 2 |
| NEDD8 | NEDD8 Ubiquitin Like Modifier |
| MED15 | Mediator Complex Subunit 15 |
| PLCB2 | Phospholipase C Beta 2 |
| UBE2V2 | Ubiquitin Conjugating Enzyme E2 V2 |
| LONP1 | Lon Peptidase 1, Mitochondrial |
| SMARCA5 | SWI/SNF Related, Matrix Associated, Actin Dependent Regulator Of Chromatin, Subfamily A, Member 5 |
| COQ8A | Coenzyme Q8A |
| TMSB4X | Thymosin Beta 4 X-Linked |
| UBE2E1 | Ubiquitin Conjugating Enzyme E2 E1 |
| CILP | Cartilage Intermediate Layer Protein |
| NUP153 | Nucleoporin 153 |
| MEF2B | Myocyte Enhancer Factor 2B |
| GRK2 | G Protein-Coupled Receptor Kinase 2 |
| CTSW | Cathepsin W |
| P2RY14 | Purinergic Receptor P2Y14 |
| SLC2A3 | Solute Carrier Family 2 Member 3 |
| TTC4 | Tetratricopeptide Repeat Domain 4 |
| IGLV@ | Immunoglobulin Lambda Variable Cluster |
| SOCS5 | Suppressor Of Cytokine Signaling 5 |
| TRIM27 | Tripartite Motif Containing 27 |
| PYDC1 | Pyrin Domain Containing 1 |
| ALG3 | ALG3 Alpha-1,3- Mannosyltransferase |
| IP6K2 | Inositol Hexakisphosphate Kinase 2 |
| CHRDL1 | Chordin Like 1 |
| ESCO2 | Establishment Of Sister Chromatid Cohesion N-Acetyltransferase 2 |
| PDSS2 | Decaprenyl Diphosphate Synthase Subunit 2 |
| CD300H | CD300H Molecule (Gene/Pseudogene) |
| SPTBN1 | Spectrin Beta, Non-Erythrocytic 1 |
| PROK2 | Prokineticin 2 |
| PAPSS2 | 3'-Phosphoadenosine 5'-Phosphosulfate Synthase 2 |
| SMARCAD1 | SWI/SNF-Related, Matrix-Associated Actin-Dependent Regulator Of Chromatin, Subfamily A, Containing DEAD/H Box 1 |
| CDK10 | Cyclin Dependent Kinase 10 |
| SETD5 | SET Domain Containing 5 |
| SHROOM4 | Shroom Family Member 4 |
| SURF4 | Surfeit 4 |
| ELOB | Elongin B |
| CALCOCO2 | Calcium Binding And Coiled-Coil Domain 2 |
| ITGA5 | Integrin Subunit Alpha 5 |
| CCT2 | Chaperonin Containing TCP1 Subunit 2 |
| GRPR | Gastrin Releasing Peptide Receptor |
| APOBEC3D | Apolipoprotein B MRNA Editing Enzyme Catalytic Subunit 3D |
| MAGEA1 | MAGE Family Member A1 |
| DYNC1I2 | Dynein Cytoplasmic 1 Intermediate Chain 2 |
| DYNLT1 | Dynein Light Chain Tctex-Type 1 |
| LMNB1 | Lamin B1 |
| RPLP2 | Ribosomal Protein Lateral Stalk Subunit P2 |
| LRMP | Lymphoid Restricted Membrane Protein |
| CLOCK | Clock Circadian Regulator |
| RAE1 | Ribonucleic Acid Export 1 |
| TRGC2 | T Cell Receptor Gamma Constant 2 |
| NCOR2 | Nuclear Receptor Corepressor 2 |
| YWHAQ | Tyrosine 3-Monooxygenase/Tryptophan 5-Monooxygenase Activation Protein Theta |
| OGT | O-Linked N-Acetylglucosamine (GlcNAc) Transferase |
| TET1 | Tet Methylcytosine Dioxygenase 1 |
| DCSTAMP | Dendrocyte Expressed Seven Transmembrane Protein |
| ALOX5AP | Arachidonate 5-Lipoxygenase Activating Protein |
| POU5F1 | POU Class 5 Homeobox 1 |
| NDUFA13 | NADH:Ubiquinone Oxidoreductase Subunit A13 |
| SLC26A5 | Solute Carrier Family 26 Member 5 |
| SYTL4 | Synaptotagmin Like 4 |
| PIGB | Phosphatidylinositol Glycan Anchor Biosynthesis Class B |
| PDCL | Phosducin Like |
| AK8 | Adenylate Kinase 8 |
| CATSPER1 | Cation Channel Sperm Associated 1 |
| MXD4 | MAX Dimerization Protein 4 |
| FAM124A | Family With Sequence Similarity 124 Member A |
| RNASEH2B-AS1 | RNASEH2B Antisense RNA 1 |
| C13orf42 | Chromosome 13 Open Reading Frame 42 |
| ATRIP-TREX1 | ATRIP-TREX1 Readthrough |
| LOC106029312 | Williams-Beuren Syndrome Medial Block B Recombination Region |
| PNMA2 | PNMA Family Member 2 |
| TFAM | Transcription Factor A, Mitochondrial |
| GPBAR1 | G Protein-Coupled Bile Acid Receptor 1 |
| GHSR | Growth Hormone Secretagogue Receptor |
| ACAA1 | Acetyl-CoA Acyltransferase 1 |
| SLC7A8 | Solute Carrier Family 7 Member 8 |
| DENND1B | DENN Domain Containing 1B |
| CCL14 | C-C Motif Chemokine Ligand 14 |
| ATP11A | ATPase Phospholipid Transporting 11A |
| IGLP2 | Immune Response To Synthetic Polypeptides 2 |
| DDX60 | DExD/H-Box Helicase 60 |
| EPHA2 | EPH Receptor A2 |
| NR0B1 | Nuclear Receptor Subfamily 0 Group B Member 1 |
| FXN | Frataxin |
| POLK | DNA Polymerase Kappa |
| PAX1 | Paired Box 1 |
| KCNE2 | Potassium Voltage-Gated Channel Subfamily E Regulatory Subunit 2 |
| TBATA | Thymus, Brain And Testes Associated |
| TENT5C | Terminal Nucleotidyltransferase 5C |
| KPNA4 | Karyopherin Subunit Alpha 4 |
| GNA15 | G Protein Subunit Alpha 15 |
| MIRLET7A1 | MicroRNA Let-7a-1 |
| TK1 | Thymidine Kinase 1 |
| ANK2 | Ankyrin 2 |
| KLF6 | Kruppel Like Factor 6 |
| CS | Citrate Synthase |
| HMOX2 | Heme Oxygenase 2 |
| ABI1 | Abl Interactor 1 |
| PEX2 | Peroxisomal Biogenesis Factor 2 |
| FEN1 | Flap Structure-Specific Endonuclease 1 |
| IGSF8 | Immunoglobulin Superfamily Member 8 |
| NFX1 | Nuclear Transcription Factor, X-Box Binding 1 |
| MSTN | Myostatin |
| USH1C | USH1 Protein Network Component Harmonin |
| PRKCI | Protein Kinase C Iota |
| CDH2 | Cadherin 2 |
| BRI3 | Brain Protein I3 |
| BATF2 | Basic Leucine Zipper ATF-Like Transcription Factor 2 |
| SOAT1 | Sterol O-Acyltransferase 1 |
| MDH2 | Malate Dehydrogenase 2 |
| TFF1 | Trefoil Factor 1 |
| PSORS1C3 | Psoriasis Susceptibility 1 Candidate 3 |
| LRRFIP1 | LRR Binding FLII Interacting Protein 1 |
| PPP2CB | Protein Phosphatase 2 Catalytic Subunit Beta |
| BACE1 | Beta-Secretase 1 |
| MIR18A | MicroRNA 18a |
| PLCB3 | Phospholipase C Beta 3 |
| RHOG | Ras Homolog Family Member G |
| GALK1 | Galactokinase 1 |
| CD5L | CD5 Molecule Like |
| MIR485 | MicroRNA 485 |
| MCM2 | Minichromosome Maintenance Complex Component 2 |
| CTSV | Cathepsin V |
| BAZ2A | Bromodomain Adjacent To Zinc Finger Domain 2A |
| DEFB106A | Defensin Beta 106A |
| NES | Nestin |
| FERMT2 | Fermitin Family Member 2 |
| GCNT1 | Glucosaminyl (N-Acetyl) Transferase 1 |
| MARK3 | Microtubule Affinity Regulating Kinase 3 |
| CLTC | Clathrin Heavy Chain |
| GNRHR | Gonadotropin Releasing Hormone Receptor |
| NEDD4L | NEDD4 Like E3 Ubiquitin Protein Ligase |
| TRAPPC1 | Trafficking Protein Particle Complex 1 |
| MLEC | Malectin |
| AUTS2 | Activator Of Transcription And Developmental Regulator AUTS2 |
| SYNGAP1 | Synaptic Ras GTPase Activating Protein 1 |
| LPO | Lactoperoxidase |
| YAP1 | Yes Associated Protein 1 |
| PDGFA | Platelet Derived Growth Factor Subunit A |
| KCNQ1 | Potassium Voltage-Gated Channel Subfamily Q Member 1 |
| ZNF683 | Zinc Finger Protein 683 |
| PRPF19 | Pre-MRNA Processing Factor 19 |
| RACK1 | Receptor For Activated C Kinase 1 |
| TGFBR3 | Transforming Growth Factor Beta Receptor 3 |
| TIGIT | T Cell Immunoreceptor With Ig And ITIM Domains |
| NUP35 | Nucleoporin 35 |
| IFT140 | Intraflagellar Transport 140 |
| ILK | Integrin Linked Kinase |
| DHCR24 | 24-Dehydrocholesterol Reductase |
| MGAT2 | Alpha-1,6-Mannosyl-Glycoprotein 2-Beta-N-Acetylglucosaminyltransferase |
| MAN2B2 | Mannosidase Alpha Class 2B Member 2 |
| MVP | Major Vault Protein |
| CTSZ | Cathepsin Z |
| UQCRFS1 | Ubiquinol-Cytochrome C Reductase, Rieske Iron-Sulfur Polypeptide 1 |
| SERPINI1 | Serpin Family I Member 1 |
| GDF5 | Growth Differentiation Factor 5 |
| MCOLN1 | Mucolipin 1 |
| WRNIP1 | WRN Helicase Interacting Protein 1 |
| SLC39A14 | Solute Carrier Family 39 Member 14 |
| GRHL2 | Grainyhead Like Transcription Factor 2 |
| HPS4 | HPS4 Biogenesis Of Lysosomal Organelles Complex 3 Subunit 2 |
| TAPBPL | TAP Binding Protein Like |
| DDIT3 | DNA Damage Inducible Transcript 3 |
| PDE7A | Phosphodiesterase 7A |
| SFTPB | Surfactant Protein B |
| CSNK1A1 | Casein Kinase 1 Alpha 1 |
| HNRNPH1 | Heterogeneous Nuclear Ribonucleoprotein H1 |
| SERPINB13 | Serpin Family B Member 13 |
| PSORS1C2 | Psoriasis Susceptibility 1 Candidate 2 |
| MACIR | Macrophage Immunometabolism Regulator |
| CDC5L | Cell Division Cycle 5 Like |
| TAC3 | Tachykinin Precursor 3 |
| DEFB106B | Defensin Beta 106B |
| GNAI1 | G Protein Subunit Alpha I1 |
| GNAO1 | G Protein Subunit Alpha O1 |
| SUMO1 | Small Ubiquitin Like Modifier 1 |
| TPD52 | Tumor Protein D52 |
| SLC6A3 | Solute Carrier Family 6 Member 3 |
| EIF3H | Eukaryotic Translation Initiation Factor 3 Subunit H |
| ORAI2 | ORAI Calcium Release-Activated Calcium Modulator 2 |
| ANO6 | Anoctamin 6 |
| ART1 | ADP-Ribosyltransferase 1 |
| SPNS2 | Sphingolipid Transporter 2 |
| DEFB114 | Defensin Beta 114 |
| NDRG1 | N-Myc Downstream Regulated 1 |
| WDR1 | WD Repeat Domain 1 |
| CHRNA7 | Cholinergic Receptor Nicotinic Alpha 7 Subunit |
| CALML5 | Calmodulin Like 5 |
| PIP5K1B | Phosphatidylinositol-4-Phosphate 5-Kinase Type 1 Beta |
| RRAS | RAS Related |
| MFN2 | Mitofusin 2 |
| CCN1 | Cellular Communication Network Factor 1 |
| NUP62 | Nucleoporin 62 |
| NUP88 | Nucleoporin 88 |
| TRADD | TNFRSF1A Associated Via Death Domain |
| ACR | Acrosin |
| PRPF3 | Pre-MRNA Processing Factor 3 |
| TPPP | Tubulin Polymerization Promoting Protein |
| ID4 | Inhibitor Of DNA Binding 4, HLH Protein |
| LAD1 | Ladinin 1 |
| L1CAM | L1 Cell Adhesion Molecule |
| PLCB4 | Phospholipase C Beta 4 |
| CACNA1F | Calcium Voltage-Gated Channel Subunit Alpha1 F |
| ARL2BP | ADP Ribosylation Factor Like GTPase 2 Binding Protein |
| KCNJ2 | Potassium Inwardly Rectifying Channel Subfamily J Member 2 |
| CRYAA | Crystallin Alpha A |
| BRD3 | Bromodomain Containing 3 |
| ZMYM2 | Zinc Finger MYM-Type Containing 2 |
| CCHCR1 | Coiled-Coil Alpha-Helical Rod Protein 1 |
| MIR96 | MicroRNA 96 |
| EEF2 | Eukaryotic Translation Elongation Factor 2 |
| GP2 | Glycoprotein 2 |
| GGT3P | Gamma-Glutamyltransferase 3 Pseudogene |
| YWHAE | Tyrosine 3-Monooxygenase/Tryptophan 5-Monooxygenase Activation Protein Epsilon |
| TRBV17 | T Cell Receptor Beta Variable 17 (Non-Functional) |
| GM2A | GM2 Ganglioside Activator |
| KLK15 | Kallikrein Related Peptidase 15 |
| NDC1 | NDC1 Transmembrane Nucleoporin |
| VAPA | VAMP Associated Protein A |
| IGKJ | Immunoglobulin Kappa Joining Cluster |
| IGLJ | Immunoglobulin Lambda Joining Cluster |
| HECTD3 | HECT Domain E3 Ubiquitin Protein Ligase 3 |
| TRIM6 | Tripartite Motif Containing 6 |
| CCDC28B | Coiled-Coil Domain Containing 28B |
| PIGT | Phosphatidylinositol Glycan Anchor Biosynthesis Class T |
| COQ8B | Coenzyme Q8B |
| CYP11B2 | Cytochrome P450 Family 11 Subfamily B Member 2 |
| PKP3 | Plakophilin 3 |
| TERF2IP | TERF2 Interacting Protein |
| MBD4 | Methyl-CpG Binding Domain 4, DNA Glycosylase |
| TOMM40 | Translocase Of Outer Mitochondrial Membrane 40 |
| FOSL1 | FOS Like 1, AP-1 Transcription Factor Subunit |
| RPL10 | Ribosomal Protein L10 |
| CST6 | Cystatin E/M |
| ETS2 | ETS Proto-Oncogene 2, Transcription Factor |
| DDX17 | DEAD-Box Helicase 17 |
| IDE | Insulin Degrading Enzyme |
| MIR29C | MicroRNA 29c |
| ITGB5 | Integrin Subunit Beta 5 |
| RBBP5 | RB Binding Protein 5, Histone Lysine Methyltransferase Complex Subunit |
| SLC2A2 | Solute Carrier Family 2 Member 2 |
| TRPC6 | Transient Receptor Potential Cation Channel Subfamily C Member 6 |
| ACADSB | Acyl-CoA Dehydrogenase Short/Branched Chain |
| GLP1R | Glucagon Like Peptide 1 Receptor |
| TSHB | Thyroid Stimulating Hormone Subunit Beta |
| PDAP1 | PDGFA Associated Protein 1 |
| SLC1A2 | Solute Carrier Family 1 Member 2 |
| UGP2 | UDP-Glucose Pyrophosphorylase 2 |
| TBX20 | T-Box Transcription Factor 20 |
| MGST2 | Microsomal Glutathione S-Transferase 2 |
| RRAGD | Ras Related GTP Binding D |
| GLDN | Gliomedin |
| DCLK3 | Doublecortin Like Kinase 3 |
| PTER | Phosphotriesterase Related |
| PCDHA2 | Protocadherin Alpha 2 |
| OLAH | Oleoyl-ACP Hydrolase |
| HECTD4 | HECT Domain E3 Ubiquitin Protein Ligase 4 |
| ZFAS1 | ZNFX1 Antisense RNA 1 |
| MIR330 | MicroRNA 330 |
| BMPR2 | Bone Morphogenetic Protein Receptor Type 2 |
| HPS5 | HPS5 Biogenesis Of Lysosomal Organelles Complex 2 Subunit 2 |
| BLOC1S3 | Biogenesis Of Lysosomal Organelles Complex 1 Subunit 3 |
| BAHD1 | Bromo Adjacent Homology Domain Containing 1 |
| AKIRIN2 | Akirin 2 |
| SPA17 | Sperm Autoantigenic Protein 17 |
| LAMTOR1 | Late Endosomal/Lysosomal Adaptor, MAPK And MTOR Activator 1 |
| TIMMDC1 | Translocase Of Inner Mitochondrial Membrane Domain Containing 1 |
| ABCC9 | ATP Binding Cassette Subfamily C Member 9 |
| GATAD1 | GATA Zinc Finger Domain Containing 1 |
| NPPA | Natriuretic Peptide A |
| OPRL1 | Opioid Related Nociceptin Receptor 1 |
| JPH4 | Junctophilin 4 |
| CDC6 | Cell Division Cycle 6 |
| NSF | N-Ethylmaleimide Sensitive Factor, Vesicle Fusing ATPase |
| TRDC | T Cell Receptor Delta Constant |
| MIR182 | MicroRNA 182 |
| AFDN | Afadin, Adherens Junction Formation Factor |
| IMPDH2 | Inosine Monophosphate Dehydrogenase 2 |
| ROM1 | Retinal Outer Segment Membrane Protein 1 |
| ATIC | 5-Aminoimidazole-4-Carboxamide Ribonucleotide Formyltransferase/IMP Cyclohydrolase |
| ZFHX3 | Zinc Finger Homeobox 3 |
| CSGALNACT1 | Chondroitin Sulfate N-Acetylgalactosaminyltransferase 1 |
| RILPL2 | Rab Interacting Lysosomal Protein Like 2 |
| IGAD1 | Immunoglobulin A (IgA) Deficiency Susceptibility 1 |
| NPAS2 | Neuronal PAS Domain Protein 2 |
| TRIL | TLR4 Interactor With Leucine Rich Repeats |
| UTRN | Utrophin |
| ADCY3 | Adenylate Cyclase 3 |
| SEC24B | SEC24 Homolog B, COPII Coat Complex Component |
| UBAC1 | UBA Domain Containing 1 |
| HDAC8 | Histone Deacetylase 8 |
| LARP7 | La Ribonucleoprotein 7, Transcriptional Regulator |
| FER | FER Tyrosine Kinase |
| ID2 | Inhibitor Of DNA Binding 2 |
| SH3BP2 | SH3 Domain Binding Protein 2 |
| YWHAH | Tyrosine 3-Monooxygenase/Tryptophan 5-Monooxygenase Activation Protein Eta |
| GNG12 | G Protein Subunit Gamma 12 |
| MPZL3 | Myelin Protein Zero Like 3 |
| MYH2 | Myosin Heavy Chain 2 |
| HNRNPH2 | Heterogeneous Nuclear Ribonucleoprotein H2 |
| ETV5 | ETS Variant Transcription Factor 5 |
| LAMA1 | Laminin Subunit Alpha 1 |
| CPS1 | Carbamoyl-Phosphate Synthase 1 |
| MZB1 | Marginal Zone B And B1 Cell Specific Protein |
| LOC102723996 | ICOS Ligand |
| SLC12A4 | Solute Carrier Family 12 Member 4 |
| AP2A2 | Adaptor Related Protein Complex 2 Subunit Alpha 2 |
| SPRED1 | Sprouty Related EVH1 Domain Containing 1 |
| MIR98 | MicroRNA 98 |
| SERPINB2 | Serpin Family B Member 2 |
| PRPF31 | Pre-MRNA Processing Factor 31 |
| HARS1 | Histidyl-TRNA Synthetase 1 |
| FZD6 | Frizzled Class Receptor 6 |
| MYPN | Myopalladin |
| EVC2 | EvC Ciliary Complex Subunit 2 |
| EVC | EvC Ciliary Complex Subunit 1 |
| SIX3 | SIX Homeobox 3 |
| C17orf99 | Chromosome 17 Open Reading Frame 99 |
| SPATA2 | Spermatogenesis Associated 2 |
| GALNT3 | Polypeptide N-Acetylgalactosaminyltransferase 3 |
| CUL7 | Cullin 7 |
| CDK13 | Cyclin Dependent Kinase 13 |
| TRIM39 | Tripartite Motif Containing 39 |
| MTR | 5-Methyltetrahydrofolate-Homocysteine Methyltransferase |
| AP1M1 | Adaptor Related Protein Complex 1 Subunit Mu 1 |
| CHD8 | Chromodomain Helicase DNA Binding Protein 8 |
| FAM135A | Family With Sequence Similarity 135 Member A |
| MS | Multiple Sclerosis |
| PGK1 | Phosphoglycerate Kinase 1 |
| GK | Glycerol Kinase |
| CACNA1S | Calcium Voltage-Gated Channel Subunit Alpha1 S |
| THEMIS | Thymocyte Selection Associated |
| CDC34 | Cell Division Cycle 34 |
| ATG3 | Autophagy Related 3 |
| UBTF | Upstream Binding Transcription Factor |
| GGCT | Gamma-Glutamylcyclotransferase |
| HABP2 | Hyaluronan Binding Protein 2 |
| TMPRSS15 | Transmembrane Serine Protease 15 |
| NCOA3 | Nuclear Receptor Coactivator 3 |
| CNOT3 | CCR4-NOT Transcription Complex Subunit 3 |
| MAP7 | Microtubule Associated Protein 7 |
| KCNA3 | Potassium Voltage-Gated Channel Subfamily A Member 3 |
| MCEMP1 | Mast Cell Expressed Membrane Protein 1 |
| ADH1C | Alcohol Dehydrogenase 1C (Class I), Gamma Polypeptide |
| SMN2 | Survival Of Motor Neuron 2, Centromeric |
| REG1A | Regenerating Family Member 1 Alpha |
| KISS1R | KISS1 Receptor |
| ANKH | ANKH Inorganic Pyrophosphate Transport Regulator |
| HOXA3 | Homeobox A3 |
| CTNNA2 | Catenin Alpha 2 |
| PRSS57 | Serine Protease 57 |
| IMPDH1 | Inosine Monophosphate Dehydrogenase 1 |
| TRAF3IP1 | TRAF3 Interacting Protein 1 |
| RBBP8 | RB Binding Protein 8, Endonuclease |
| SCYL1 | SCY1 Like Pseudokinase 1 |
| YWHAG | Tyrosine 3-Monooxygenase/Tryptophan 5-Monooxygenase Activation Protein Gamma |
| CD300LG | CD300 Molecule Like Family Member G |
| PKD1 | Polycystin 1, Transient Receptor Potential Channel Interacting |
| CLDN3 | Claudin 3 |
| MYDGF | Myeloid Derived Growth Factor |
| INPP5E | Inositol Polyphosphate-5-Phosphatase E |
| ATP5MG | ATP Synthase Membrane Subunit G |
| GIMAP7 | GTPase, IMAP Family Member 7 |
| EIF4G2 | Eukaryotic Translation Initiation Factor 4 Gamma 2 |
| MKRN1 | Makorin Ring Finger Protein 1 |
| DNASE1L1 | Deoxyribonuclease 1 Like 1 |
| MUC12 | Mucin 12, Cell Surface Associated |
| LTN1 | Listerin E3 Ubiquitin Protein Ligase 1 |
| TCP1 | T-Complex 1 |
| GJA5 | Gap Junction Protein Alpha 5 |
| TBX5 | T-Box Transcription Factor 5 |
| CA5A | Carbonic Anhydrase 5A |
| KLK8 | Kallikrein Related Peptidase 8 |
| WDFY3 | WD Repeat And FYVE Domain Containing 3 |
| RAB39B | RAB39B, Member RAS Oncogene Family |
| SLC12A8 | Solute Carrier Family 12 Member 8 |
| OTOA | Otoancorin |
| SDR16C5 | Short Chain Dehydrogenase/Reductase Family 16C Member 5 |
| ZNF74 | Zinc Finger Protein 74 |
| KRT74 | Keratin 74 |
| ZNF346 | Zinc Finger Protein 346 |
| CASC3 | CASC3 Exon Junction Complex Subunit |
| TCF15 | Transcription Factor 15 |
| PRSS54 | Serine Protease 54 |
| DGCR6L | DiGeorge Syndrome Critical Region Gene 6 Like |
| NXF5 | Nuclear RNA Export Factor 5 |
| PCDHGB7 | Protocadherin Gamma Subfamily B, 7 |
| C2orf49 | Chromosome 2 Open Reading Frame 49 |
| SPATA46 | Spermatogenesis Associated 46 |
| GGTLC3 | Gamma-Glutamyltransferase Light Chain Family Member 3 |
| PCAT1 | Prostate Cancer Associated Transcript 1 |
| MIR215 | MicroRNA 215 |
| MIR335 | MicroRNA 335 |
| C2-AS1 | C2 Antisense RNA 1 |
| ENSG00000235775 |  |
| TRGC1 | T Cell Receptor Gamma Constant 1 |
| TRGJ1 | T Cell Receptor Gamma Joining 1 |
| BIRC7 | Baculoviral IAP Repeat Containing 7 |
| NR2C2 | Nuclear Receptor Subfamily 2 Group C Member 2 |
| VTI1B | Vesicle Transport Through Interaction With T-SNAREs 1B |
| USP9X | Ubiquitin Specific Peptidase 9 X-Linked |
| PIP5K1A | Phosphatidylinositol-4-Phosphate 5-Kinase Type 1 Alpha |
| RASGRF1 | Ras Protein Specific Guanine Nucleotide Releasing Factor 1 |
| TBP | TATA-Box Binding Protein |
| PCLAF | PCNA Clamp Associated Factor |
| GBP6 | Guanylate Binding Protein Family Member 6 |
| COQ6 | Coenzyme Q6, Monooxygenase |
| IFT43 | Intraflagellar Transport 43 |
| IGF2-AS | IGF2 Antisense RNA |
| ATF1 | Activating Transcription Factor 1 |
| DEFB107B | Defensin Beta 107B |
| DEFB107A | Defensin Beta 107A |
| CDK5 | Cyclin Dependent Kinase 5 |
| NUP155 | Nucleoporin 155 |
| PJA2 | Praja Ring Finger Ubiquitin Ligase 2 |
| DLX4 | Distal-Less Homeobox 4 |
| RUNX3 | RUNX Family Transcription Factor 3 |
| TRH | Thyrotropin Releasing Hormone |
| AURKA | Aurora Kinase A |
| CACNA2D1 | Calcium Voltage-Gated Channel Auxiliary Subunit Alpha2delta 1 |
| PLOD2 | Procollagen-Lysine,2-Oxoglutarate 5-Dioxygenase 2 |
| STC1 | Stanniocalcin 1 |
| GOSR1 | Golgi SNAP Receptor Complex Member 1 |
| COG3 | Component Of Oligomeric Golgi Complex 3 |
| ABI2 | Abl Interactor 2 |
| POR | Cytochrome P450 Oxidoreductase |
| BPGM | Bisphosphoglycerate Mutase |
| CTNS | Cystinosin, Lysosomal Cystine Transporter |
| GPC1 | Glypican 1 |
| TMEM231 | Transmembrane Protein 231 |
| WDPCP | WD Repeat Containing Planar Cell Polarity Effector |
| JDP2 | Jun Dimerization Protein 2 |
| CPA3 | Carboxypeptidase A3 |
| DR1 | Down-Regulator Of Transcription 1 |
| ATP6V0D1 | ATPase H+ Transporting V0 Subunit D1 |
| MYBL2 | MYB Proto-Oncogene Like 2 |
| HBS1L | HBS1 Like Translational GTPase |
| ZFPM1 | Zinc Finger Protein, FOG Family Member 1 |
| BAG1 | BAG Cochaperone 1 |
| PRKAB1 | Protein Kinase AMP-Activated Non-Catalytic Subunit Beta 1 |
| PSG2 | Pregnancy Specific Beta-1-Glycoprotein 2 |
| ADM | Adrenomedullin |
| FAF2 | Fas Associated Factor Family Member 2 |
| FOSB | FosB Proto-Oncogene, AP-1 Transcription Factor Subunit |
| PPP2CA | Protein Phosphatase 2 Catalytic Subunit Alpha |
| BMP5 | Bone Morphogenetic Protein 5 |
| F12 | Coagulation Factor XII |
| PDHX | Pyruvate Dehydrogenase Complex Component X |
| KLK5 | Kallikrein Related Peptidase 5 |
| IGHV4-30-2 | Immunoglobulin Heavy Variable 4-30-2 |
| POLR2G | RNA Polymerase II Subunit G |
| KNTC1 | Kinetochore Associated 1 |
| EXOC1 | Exocyst Complex Component 1 |
| SRPRA | SRP Receptor Subunit Alpha |
| MT-ATP8 | Mitochondrially Encoded ATP Synthase Membrane Subunit 8 |
| SGCD | Sarcoglycan Delta |
| TNNC1 | Troponin C1, Slow Skeletal And Cardiac Type |
| CYP26C1 | Cytochrome P450 Family 26 Subfamily C Member 1 |
| TCAP | Titin-Cap |
| CSRP3 | Cysteine And Glycine Rich Protein 3 |
| PPCS | Phosphopantothenoylcysteine Synthetase |
| DOCK6 | Dedicator Of Cytokinesis 6 |
| NEBL | Nebulette |
| TAF1A | TATA-Box Binding Protein Associated Factor, RNA Polymerase I Subunit A |
| RBM20 | RNA Binding Motif Protein 20 |
| FDX2 | Ferredoxin 2 |
| CDON | Cell Adhesion Associated, Oncogene Regulated |
| FOXD3 | Forkhead Box D3 |
| DISP1 | Dispatched RND Transporter Family Member 1 |
| PPP2R5A | Protein Phosphatase 2 Regulatory Subunit B'Alpha |
| CKLF | Chemokine Like Factor |
| MYL3 | Myosin Light Chain 3 |
| AFF4 | AF4/FMR2 Family Member 4 |
| SATB1 | SATB Homeobox 1 |
| CACTIN | Cactin, Spliceosome C Complex Subunit |
| BAAT | Bile Acid-CoA:Amino Acid N-Acyltransferase |
| ACER3 | Alkaline Ceramidase 3 |
| TRIB3 | Tribbles Pseudokinase 3 |
| RASSF1 | Ras Association Domain Family Member 1 |
| PLA2G3 | Phospholipase A2 Group III |
| PINK1 | PTEN Induced Kinase 1 |
| PPT1 | Palmitoyl-Protein Thioesterase 1 |
| SPDEF | SAM Pointed Domain Containing ETS Transcription Factor |
| BATF3 | Basic Leucine Zipper ATF-Like Transcription Factor 3 |
| CACNA1D | Calcium Voltage-Gated Channel Subunit Alpha1 D |
| MIR20B | MicroRNA 20b |
| EXOC4 | Exocyst Complex Component 4 |
| SREBF1 | Sterol Regulatory Element Binding Transcription Factor 1 |
| NCOA1 | Nuclear Receptor Coactivator 1 |
| AXIN2 | Axin 2 |
| SULT2A1 | Sulfotransferase Family 2A Member 1 |
| PEG10 | Paternally Expressed 10 |
| RAB7B | RAB7B, Member RAS Oncogene Family |
| FKBP8 | FKBP Prolyl Isomerase 8 |
| NCKAP1L | NCK Associated Protein 1 Like |
| CRISPLD2 | Cysteine Rich Secretory Protein LCCL Domain Containing 2 |
| SEM1 | SEM1 26S Proteasome Complex Subunit |
| EIF4A1 | Eukaryotic Translation Initiation Factor 4A1 |
| SMURF1 | SMAD Specific E3 Ubiquitin Protein Ligase 1 |
| GNAI2 | G Protein Subunit Alpha I2 |
| ACKR3 | Atypical Chemokine Receptor 3 |
| AMHR2 | Anti-Mullerian Hormone Receptor Type 2 |
| CYP51A1 | Cytochrome P450 Family 51 Subfamily A Member 1 |
| HAND2 | Heart And Neural Crest Derivatives Expressed 2 |
| GDF11 | Growth Differentiation Factor 11 |
| ANXA9 | Annexin A9 |
| PLEKHM2 | Pleckstrin Homology And RUN Domain Containing M2 |
| GCSAM | Germinal Center Associated Signaling And Motility |
| SULT1C3 | Sulfotransferase Family 1C Member 3 |
| CTTNBP2 | Cortactin Binding Protein 2 |
| TAS2R42 | Taste 2 Receptor Member 42 |
| SEPTIN5 | Septin 5 |
| CYS1 | Cystin 1 |
| BICDL1 | BICD Family Like Cargo Adaptor 1 |
| YBX1 | Y-Box Binding Protein 1 |
| TAF1 | TATA-Box Binding Protein Associated Factor 1 |
| SHPK | Sedoheptulokinase |
| ARNT | Aryl Hydrocarbon Receptor Nuclear Translocator |
| WDR77 | WD Repeat Domain 77 |
| GRIN2D | Glutamate Ionotropic Receptor NMDA Type Subunit 2D |
| SFRP1 | Secreted Frizzled Related Protein 1 |
| POLR2B | RNA Polymerase II Subunit B |
| SIGLEC14 | Sialic Acid Binding Ig Like Lectin 14 |
| EMSY | EMSY Transcriptional Repressor, BRCA2 Interacting |
| INVS | Inversin |
| MMP10 | Matrix Metallopeptidase 10 |
| ATG14 | Autophagy Related 14 |
| LRRC32 | Leucine Rich Repeat Containing 32 |
| PALLD | Palladin, Cytoskeletal Associated Protein |
| ZNF335 | Zinc Finger Protein 335 |
| PTPMT1 | Protein Tyrosine Phosphatase Mitochondrial 1 |
| MCAM | Melanoma Cell Adhesion Molecule |
| IGFBP6 | Insulin Like Growth Factor Binding Protein 6 |
| DUSP3 | Dual Specificity Phosphatase 3 |
| SRA1 | Steroid Receptor RNA Activator 1 |
| PRCP | Prolylcarboxypeptidase |
| RNF14 | Ring Finger Protein 14 |
| S100P | S100 Calcium Binding Protein P |
| KIF3B | Kinesin Family Member 3B |
| CRY2 | Cryptochrome Circadian Regulator 2 |
| RAB1A | RAB1A, Member RAS Oncogene Family |
| DCAF7 | DDB1 And CUL4 Associated Factor 7 |
| EPAS1 | Endothelial PAS Domain Protein 1 |
| DTX4 | Deltex E3 Ubiquitin Ligase 4 |
| LEXM | Lymphocyte Expansion Molecule |
| CASP5 | Caspase 5 |
| ACTR1A | Actin Related Protein 1A |
| MRAS | Muscle RAS Oncogene Homolog |
| DDRGK1 | DDRGK Domain Containing 1 |
| KIF23 | Kinesin Family Member 23 |
| PLA2G2E | Phospholipase A2 Group IIE |
| SIK1 | Salt Inducible Kinase 1 |
| TRIO | Trio Rho Guanine Nucleotide Exchange Factor |
| ZNHIT3 | Zinc Finger HIT-Type Containing 3 |
| KEAP1 | Kelch Like ECH Associated Protein 1 |
| MCM3AP | Minichromosome Maintenance Complex Component 3 Associated Protein |
| TRPV4 | Transient Receptor Potential Cation Channel Subfamily V Member 4 |
| CA1 | Carbonic Anhydrase 1 |
| HOXC13 | Homeobox C13 |
| PDC | Phosducin |
| ADRB1 | Adrenoceptor Beta 1 |
| SRSF6 | Serine And Arginine Rich Splicing Factor 6 |
| SOD3 | Superoxide Dismutase 3 |
| DRD3 | Dopamine Receptor D3 |
| NXF1 | Nuclear RNA Export Factor 1 |
| GRIN2C | Glutamate Ionotropic Receptor NMDA Type Subunit 2C |
| SRSF7 | Serine And Arginine Rich Splicing Factor 7 |
| IRS4 | Insulin Receptor Substrate 4 |
| MIR124-1 | MicroRNA 124-1 |
| CUL2 | Cullin 2 |
| CCNDBP1 | Cyclin D1 Binding Protein 1 |
| PLCB1 | Phospholipase C Beta 1 |
| HMGN1 | High Mobility Group Nucleosome Binding Domain 1 |
| TRAF7 | TNF Receptor Associated Factor 7 |
| LOC111365141 | NOS2 5' Regulatory Region |
| AKR1D1 | Aldo-Keto Reductase Family 1 Member D1 |
| PHKA2 | Phosphorylase Kinase Regulatory Subunit Alpha 2 |
| PHKG2 | Phosphorylase Kinase Catalytic Subunit Gamma 2 |
| ALAS2 | 5'-Aminolevulinate Synthase 2 |
| DIS3L2 | DIS3 Like 3'-5' Exoribonuclease 2 |
| SC5D | Sterol-C5-Desaturase |
| GLRX5 | Glutaredoxin 5 |
| DEFB104B | Defensin Beta 104B |
| DEFB105A | Defensin Beta 105A |
| DEFB105B | Defensin Beta 105B |
| SEC61B | SEC61 Translocon Subunit Beta |
| ADAMTS5 | ADAM Metallopeptidase With Thrombospondin Type 1 Motif 5 |
| CAPN1 | Calpain 1 |
| HHEX | Hematopoietically Expressed Homeobox |
| ATCAY | ATCAY Kinesin Light Chain Interacting Caytaxin |
| GABARAP | GABA Type A Receptor-Associated Protein |
| NUP54 | Nucleoporin 54 |
| TRIM34 | Tripartite Motif Containing 34 |
| ADIPOR1 | Adiponectin Receptor 1 |
| PA2G4 | Proliferation-Associated 2G4 |
| ADRA1A | Adrenoceptor Alpha 1A |
| RING1 | Ring Finger Protein 1 |
| YPEL5 | Yippee Like 5 |
| XAGE1A | X Antigen Family Member 1A |
| MCM5 | Minichromosome Maintenance Complex Component 5 |
| CEP55 | Centrosomal Protein 55 |
| RPS6KA5 | Ribosomal Protein S6 Kinase A5 |
| PPP5C | Protein Phosphatase 5 Catalytic Subunit |
| SOST | Sclerostin |
| DAZAP2 | DAZ Associated Protein 2 |
| MIR422A | MicroRNA 422a |
| GRD2 | Graves Disease, Susceptibility To, 2 |
| MAPKBP1 | Mitogen-Activated Protein Kinase Binding Protein 1 |
| ADGRG5 | Adhesion G Protein-Coupled Receptor G5 |
| HIRIP3 | HIRA Interacting Protein 3 |
| COTL1 | Coactosin Like F-Actin Binding Protein 1 |
| PTMS | Parathymosin |
| ADAMDEC1 | ADAM Like Decysin 1 |
| CACNA1G | Calcium Voltage-Gated Channel Subunit Alpha1 G |
| KCNJ6 | Potassium Inwardly Rectifying Channel Subfamily J Member 6 |
| NDUFS3 | NADH:Ubiquinone Oxidoreductase Core Subunit S3 |
| SHOC2 | SHOC2 Leucine Rich Repeat Scaffold Protein |
| TBX4 | T-Box Transcription Factor 4 |
| NXN | Nucleoredoxin |
| NHLRC1 | NHL Repeat Containing E3 Ubiquitin Protein Ligase 1 |
| MED13L | Mediator Complex Subunit 13L |
| MAB21L1 | Mab-21 Like 1 |
| FAM111B | Family With Sequence Similarity 111 Member B |
| KIAA0556 | KIAA0556 |
| PWAR1 | Prader Willi/Angelman Region RNA 1 |
| ICA1 | Islet Cell Autoantigen 1 |
| AZGP1 | Alpha-2-Glycoprotein 1, Zinc-Binding |
| GNB4 | G Protein Subunit Beta 4 |
| FOXO4 | Forkhead Box O4 |
| APOC3 | Apolipoprotein C3 |
| PASD1 | PAS Domain Containing Repressor 1 |
| TRIM3 | Tripartite Motif Containing 3 |
| RNF216 | Ring Finger Protein 216 |
| BTN2A2 | Butyrophilin Subfamily 2 Member A2 |
| HPX | Hemopexin |
| MAPK7 | Mitogen-Activated Protein Kinase 7 |
| LOC108004536 | IGHD Type 1A-1 Recombination Region |
| LOC105980078 | IGHD Type 1A-2 Recombination Region |
| RNASE6 | Ribonuclease A Family Member K6 |
| MYBPH | Myosin Binding Protein H |
| UBE2B | Ubiquitin Conjugating Enzyme E2 B |
| CLTA | Clathrin Light Chain A |
| BBS4 | Bardet-Biedl Syndrome 4 |
| HNRNPC | Heterogeneous Nuclear Ribonucleoprotein C |
| RASGRF2 | Ras Protein Specific Guanine Nucleotide Releasing Factor 2 |
| SCN1A | Sodium Voltage-Gated Channel Alpha Subunit 1 |
| CEP164 | Centrosomal Protein 164 |
| UBE2E2 | Ubiquitin Conjugating Enzyme E2 E2 |
| FGF21 | Fibroblast Growth Factor 21 |
| MPP1 | Membrane Palmitoylated Protein 1 |
| GALNT14 | Polypeptide N-Acetylgalactosaminyltransferase 14 |
| MIR181B1 | MicroRNA 181b-1 |
| XRCC3 | X-Ray Repair Cross Complementing 3 |
| DDA1 | DET1 And DDB1 Associated 1 |
| PTBP1 | Polypyrimidine Tract Binding Protein 1 |
| DUSP16 | Dual Specificity Phosphatase 16 |
| BCL2L12 | BCL2 Like 12 |
| TARM1 | T Cell-Interacting, Activating Receptor On Myeloid Cells 1 |
| CACNB2 | Calcium Voltage-Gated Channel Auxiliary Subunit Beta 2 |
| RAB11FIP2 | RAB11 Family Interacting Protein 2 |
| MIA2 | MIA SH3 Domain ER Export Factor 2 |
| GNA12 | G Protein Subunit Alpha 12 |
| DCTN2 | Dynactin Subunit 2 |
| PJA1 | Praja Ring Finger Ubiquitin Ligase 1 |
| ARF6 | ADP Ribosylation Factor 6 |
| GMPS | Guanine Monophosphate Synthase |
| RELN | Reelin |
| HOXB13 | Homeobox B13 |
| PHC3 | Polyhomeotic Homolog 3 |
| MRPL40 | Mitochondrial Ribosomal Protein L40 |
| SP8 | Sp8 Transcription Factor |
| UGT1A8 | UDP Glucuronosyltransferase Family 1 Member A8 |
| CCDC54 | Coiled-Coil Domain Containing 54 |
| CFAP221 | Cilia And Flagella Associated Protein 221 |
| MIR107 | MicroRNA 107 |
| MIR574 | MicroRNA 574 |
| RNU6ATAC | RNA, U6atac Small Nuclear (U12-Dependent Splicing) |
| MT-TV | Mitochondrially Encoded TRNA-Val (GUN) |
| SM1 | Schistosoma Mansoni, Susceptibility/Resistance To |
| PPM1G | Protein Phosphatase, Mg2+/Mn2+ Dependent 1G |
| TRMT61B | TRNA Methyltransferase 61B |
| AIMP1 | Aminoacyl TRNA Synthetase Complex Interacting Multifunctional Protein 1 |
| PDE5A | Phosphodiesterase 5A |
| PYGL | Glycogen Phosphorylase L |
| CENPE | Centromere Protein E |
| PRMT1 | Protein Arginine Methyltransferase 1 |
| PCK2 | Phosphoenolpyruvate Carboxykinase 2, Mitochondrial |
| DAG1 | Dystroglycan 1 |
| ABCC3 | ATP Binding Cassette Subfamily C Member 3 |
| PLA2G12B | Phospholipase A2 Group XIIB |
| TRIM59 | Tripartite Motif Containing 59 |
| PYDC2 | Pyrin Domain Containing 2 |
| IFI35 | Interferon Induced Protein 35 |
| ELF1 | E74 Like ETS Transcription Factor 1 |
| ACACA | Acetyl-CoA Carboxylase Alpha |
| PRPF8 | Pre-MRNA Processing Factor 8 |
| MPP2 | Membrane Palmitoylated Protein 2 |
| ELAVL1 | ELAV Like RNA Binding Protein 1 |
| GIMAP1 | GTPase, IMAP Family Member 1 |
| ULK1 | Unc-51 Like Autophagy Activating Kinase 1 |
| UBE2L6 | Ubiquitin Conjugating Enzyme E2 L6 |
| RAPGEF2 | Rap Guanine Nucleotide Exchange Factor 2 |
| WDR83 | WD Repeat Domain 83 |
| ARPC1A | Actin Related Protein 2/3 Complex Subunit 1A |
| EVL | Enah/Vasp-Like |
| RHEB | Ras Homolog, MTORC1 Binding |
| HP1BP3 | Heterochromatin Protein 1 Binding Protein 3 |
| KLRF1 | Killer Cell Lectin Like Receptor F1 |
| DEFB108A | Defensin Beta 108A |
| GCSIR | GPR55 Cis Regulatory Suppressor Of Immune Response RNA |
| SP2 | Sp2 Transcription Factor |
| SLC7A6 | Solute Carrier Family 7 Member 6 |
| ANO1 | Anoctamin 1 |
| FAM3B | FAM3 Metabolism Regulating Signaling Molecule B |
| MMD | Monocyte To Macrophage Differentiation Associated |
| CENPJ | Centromere Protein J |
| RCAN2 | Regulator Of Calcineurin 2 |
| GCA | Grancalcin |
| HMHB1 | Histocompatibility Minor HB-1 |
| GLS2 | Glutaminase 2 |
| RICTOR | RPTOR Independent Companion Of MTOR Complex 2 |
| LILRA6 | Leukocyte Immunoglobulin Like Receptor A6 |
| KCNA5 | Potassium Voltage-Gated Channel Subfamily A Member 5 |
| SPOP | Speckle Type BTB/POZ Protein |
| MARVELD2 | MARVEL Domain Containing 2 |
| TOM1 | Target Of Myb1 Membrane Trafficking Protein |
| CAND1 | Cullin Associated And Neddylation Dissociated 1 |
| MUC13 | Mucin 13, Cell Surface Associated |
| KDM6B | Lysine Demethylase 6B |
| ACTR1B | Actin Related Protein 1B |
| FCRL6 | Fc Receptor Like 6 |
| GNAI3 | G Protein Subunit Alpha I3 |
| AMPD3 | Adenosine Monophosphate Deaminase 3 |
| SLC30A7 | Solute Carrier Family 30 Member 7 |
| C6orf15 | Chromosome 6 Open Reading Frame 15 |
| NFIL3 | Nuclear Factor, Interleukin 3 Regulated |
| PPP2R5C | Protein Phosphatase 2 Regulatory Subunit B'Gamma |
| MIR130A | MicroRNA 130a |
| METRNL | Meteorin Like, Glial Cell Differentiation Regulator |
| TSPAN32 | Tetraspanin 32 |
| PROX1 | Prospero Homeobox 1 |
| RNF8 | Ring Finger Protein 8 |
| CNKSR1 | Connector Enhancer Of Kinase Suppressor Of Ras 1 |
| ARMC8 | Armadillo Repeat Containing 8 |
| GFRA3 | GDNF Family Receptor Alpha 3 |
| CPA4 | Carboxypeptidase A4 |
| SMPX | Small Muscle Protein X-Linked |
| STK10 | Serine/Threonine Kinase 10 |
| SRSF1 | Serine And Arginine Rich Splicing Factor 1 |
| DLG4 | Discs Large MAGUK Scaffold Protein 4 |
| MCM7 | Minichromosome Maintenance Complex Component 7 |
| SLC39A2 | Solute Carrier Family 39 Member 2 |
| NPHP1 | Nephrocystin 1 |
| SOX3 | SRY-Box Transcription Factor 3 |
| LHX4 | LIM Homeobox 4 |
| HESX1 | HESX Homeobox 1 |
| NPHP4 | Nephrocystin 4 |
| CDR1 | Cerebellar Degeneration Related Protein 1 |
| LMAN1 | Lectin, Mannose Binding 1 |
| EVI5 | Ecotropic Viral Integration Site 5 |
| AARS1 | Alanyl-TRNA Synthetase 1 |
| ST6GAL1 | ST6 Beta-Galactoside Alpha-2,6-Sialyltransferase 1 |
| SUMF1 | Sulfatase Modifying Factor 1 |
| ABCG5 | ATP Binding Cassette Subfamily G Member 5 |
| MEGF8 | Multiple EGF Like Domains 8 |
| ST3GAL4 | ST3 Beta-Galactoside Alpha-2,3-Sialyltransferase 4 |
| NOX4 | NADPH Oxidase 4 |
| SLC9A3R1 | SLC9A3 Regulator 1 |
| CEP170 | Centrosomal Protein 170 |
| SUPT16H | SPT16 Homolog, Facilitates Chromatin Remodeling Subunit |
| SLC6A5 | Solute Carrier Family 6 Member 5 |
| SULT2B1 | Sulfotransferase Family 2B Member 1 |
| AGXT | Alanine--Glyoxylate And Serine--Pyruvate Aminotransferase |
| SLC6A19 | Solute Carrier Family 6 Member 19 |
| NDE1 | NudE Neurodevelopment Protein 1 |
| ERF | ETS2 Repressor Factor |
| CIDEC | Cell Death Inducing DFFA Like Effector C |
| XPNPEP3 | X-Prolyl Aminopeptidase 3 |
| BPTF | Bromodomain PHD Finger Transcription Factor |
| VPS33A | VPS33A Core Subunit Of CORVET And HOPS Complexes |
| ATOH7 | Atonal BHLH Transcription Factor 7 |
| MAP3K20 | Mitogen-Activated Protein Kinase Kinase Kinase 20 |
| ARHGAP29 | Rho GTPase Activating Protein 29 |
| NADK2 | NAD Kinase 2, Mitochondrial |
| SDR9C7 | Short Chain Dehydrogenase/Reductase Family 9C Member 7 |
| UROC1 | Urocanate Hydratase 1 |
| PRDM12 | PR/SET Domain 12 |
| P4HTM | Prolyl 4-Hydroxylase, Transmembrane |
| NHLRC2 | NHL Repeat Containing 2 |
| FOXE3 | Forkhead Box E3 |
| MAGEL2 | MAGE Family Member L2 |
| NAXD | NAD(P)HX Dehydratase |
| NPAP1 | Nuclear Pore Associated Protein 1 |
| PWRN1 | Prader-Willi Region Non-Protein Coding RNA 1 |
| IPW | Imprinted In Prader-Willi Syndrome |
| SNORD116-1 | Small Nucleolar RNA, C/D Box 116-1 |
| SNORD115-1 | Small Nucleolar RNA, C/D Box 115-1 |
| MKRN3-AS1 | MKRN3 Antisense RNA 1 |
| TRAF4 | TNF Receptor Associated Factor 4 |
| UBE2M | Ubiquitin Conjugating Enzyme E2 M |
| TTLL12 | Tubulin Tyrosine Ligase Like 12 |
| OTUD4 | OTU Deubiquitinase 4 |
| LEPROT | Leptin Receptor Overlapping Transcript |
| TRIM11 | Tripartite Motif Containing 11 |
| SEMG1 | Semenogelin 1 |
| DEFB110 | Defensin Beta 110 |
| DEFB128 | Defensin Beta 128 |
| SIGLEC16 | Sialic Acid Binding Ig Like Lectin 16 (Gene/Pseudogene) |
| TMPRSS6 | Transmembrane Serine Protease 6 |
| IBSP | Integrin Binding Sialoprotein |
| PTGDR | Prostaglandin D2 Receptor |
| EEA1 | Early Endosome Antigen 1 |
| POLR2E | RNA Polymerase II Subunit E |
| CIP2A | Cellular Inhibitor Of PP2A |
| ANKRD36B | Ankyrin Repeat Domain 36B |
| CDKAL1 | CDK5 Regulatory Subunit Associated Protein 1 Like 1 |
| MT2A | Metallothionein 2A |
| PURA | Purine Rich Element Binding Protein A |
| CNOT7 | CCR4-NOT Transcription Complex Subunit 7 |
| NMU | Neuromedin U |
| KCTD7 | Potassium Channel Tetramerization Domain Containing 7 |
| PPIG | Peptidylprolyl Isomerase G |
| DPP8 | Dipeptidyl Peptidase 8 |
| MAP4 | Microtubule Associated Protein 4 |
| GCLC | Glutamate-Cysteine Ligase Catalytic Subunit |
| PDE3B | Phosphodiesterase 3B |
| UBD | Ubiquitin D |
| PTPRK | Protein Tyrosine Phosphatase Receptor Type K |
| WDR36 | WD Repeat Domain 36 |
| IMPA1 | Inositol Monophosphatase 1 |
| AP3B2 | Adaptor Related Protein Complex 3 Subunit Beta 2 |
| DSC3 | Desmocollin 3 |
| NKX2-6 | NK2 Homeobox 6 |
| SLC4A1AP | Solute Carrier Family 4 Member 1 Adaptor Protein |
| TCL1B | T Cell Leukemia/Lymphoma 1B |
| H3C1 | H3 Clustered Histone 1 |
| GFER | Growth Factor, Augmenter Of Liver Regeneration |
| LTBP4 | Latent Transforming Growth Factor Beta Binding Protein 4 |
| GADD45G | Growth Arrest And DNA Damage Inducible Gamma |
| PRKAR2B | Protein Kinase CAMP-Dependent Type II Regulatory Subunit Beta |
| TRIM41 | Tripartite Motif Containing 41 |
| DVL1 | Dishevelled Segment Polarity Protein 1 |
| MAD1L1 | Mitotic Arrest Deficient 1 Like 1 |
| CNTN1 | Contactin 1 |
| KCNE1 | Potassium Voltage-Gated Channel Subfamily E Regulatory Subunit 1 |
| TSPAN12 | Tetraspanin 12 |
| SNTA1 | Syntrophin Alpha 1 |
| LMO1 | LIM Domain Only 1 |
| SRD5A2 | Steroid 5 Alpha-Reductase 2 |
| MOCOS | Molybdenum Cofactor Sulfurase |
| DNA2 | DNA Replication Helicase/Nuclease 2 |
| FRZB | Frizzled Related Protein |
| BRDT | Bromodomain Testis Associated |
| UCHL5 | Ubiquitin C-Terminal Hydrolase L5 |
| GSC | Goosecoid Homeobox |
| WDR45 | WD Repeat Domain 45 |
| CELA2A | Chymotrypsin Like Elastase 2A |
| BFSP2 | Beaded Filament Structural Protein 2 |
| ZDHHC8 | Zinc Finger DHHC-Type Palmitoyltransferase 8 |
| TIPIN | TIMELESS Interacting Protein |
| SLC38A3 | Solute Carrier Family 38 Member 3 |
| SUPT20H | SPT20 Homolog, SAGA Complex Component |
| POC1A | POC1 Centriolar Protein A |
| FOXL1 | Forkhead Box L1 |
| DNAH7 | Dynein Axonemal Heavy Chain 7 |
| TRIAP1 | TP53 Regulated Inhibitor Of Apoptosis 1 |
| ZNF334 | Zinc Finger Protein 334 |
| GSC2 | Goosecoid Homeobox 2 |
| STH | Saitohin |
| MORN1 | MORN Repeat Containing 1 |
| CEMP1 | Cementum Protein 1 |
| TP73-AS1 | TP73 Antisense RNA 1 |
| DGCR5 | DiGeorge Syndrome Critical Region Gene 5 |
| MIR449A | MicroRNA 449a |
| FBXL19-AS1 | FBXL19 Antisense RNA 1 |
| SNHG28 | Small Nucleolar RNA Host Gene 28 |
| SNHG29 | Small Nucleolar RNA Host Gene 29 |
| MIR584 | MicroRNA 584 |
| MIR1275 | MicroRNA 1275 |
| MIR1306 | MicroRNA 1306 |
| MIR521-1 | MicroRNA 521-1 |
| MIR561 | MicroRNA 561 |
| SCARNA22 | Small Cajal Body-Specific RNA 22 |
| SNORA40 | Small Nucleolar RNA, H/ACA Box 40 |
| ST3GAL6-AS1 | ST3GAL6 Antisense RNA 1 |
| DGCR10 | DiGeorge Syndrome Critical Region Gene 10 |
| FLG-AS1 | FLG Antisense RNA 1 |
| MIR664A | MicroRNA 664a |
| CCAT1 | Colon Cancer Associated Transcript 1 |
| MIR599 | MicroRNA 599 |
| MT-TI | Mitochondrially Encoded TRNA-Ile (AUU/C) |
| MT-TL2 | Mitochondrially Encoded TRNA-Leu (CUN) 2 |
| LAMA5-AS1 | LAMA5 Antisense RNA 1 |
| MT-TN | Mitochondrially Encoded TRNA-Asn (AAU/C) |
| PDIA3P1 | Protein Disulfide Isomerase Family A Member 3 Pseudogene 1 |
| MIR3681 | MicroRNA 3681 |
| MT-TA | Mitochondrially Encoded TRNA-Ala (GCN) |
| MT-TP | Mitochondrially Encoded TRNA-Pro (CCN) |
| CARD11-AS1 | CARD11 Antisense RNA 1 |
| DGCR | DiGeorge Syndrome Chromosome Region |
| PCAP | Predisposing For Prostate Cancer |
| DCR | Down Syndrome Chromosome Region |
| HPC3 | Prostate Cancer, Hereditary, 3 |
| AGMX2 | Agammaglobulinemia, X-Linked 2 (With Growth Hormone Deficiency) |
| ENSG00000278769 |  |
| MYAS1 | Myasthenia Gravis With Thymus Hyperplasia |
| DUP22Q11.2 | Chromosome 22q11.2 Microduplication Syndrome |
| PSC | Cholangitis, Primary Sclerosing |
| CILD4 | Ciliary Dyskinesia, Primary, 4 |
| DEL1P36 | Chromosome 1p36 Deletion Syndrome |
| MTBS1 | Mycobacterium Tuberculosis, Susceptibility To Infection By, 1 |
| DGS2 | DiGeorge Syndrome/Velocardiofacial Syndrome Complex 2 |
| HPC10 | Prostate Cancer, Hereditary, 10 |
| HPC14 | Prostate Cancer, Hereditary, 14 |
| HPC4 | Prostate Cancer, Hereditary, 4 |
| HPC5 | Prostate Cancer, Hereditary, 5 |
| GCCD3 | Glucocorticoid Deficiency 3 |
| NBLST4 | Neuroblastoma, Susceptibility To, 4 |
| NBLST5 | Neuroblastoma, Susceptibility To, 5 |
| NBLST6 | Neuroblastoma, Susceptibility To, 6 |
| NBLST7 | Neuroblastoma, Susceptibility To, 7 |
| RC14S | Ring Chromosome 14 Syndrome |
| PBC2 | Biliary Cirrhosis, Primary, 2 |
| PBC3 | Biliary Cirrhosis, Primary, 3 |
| PBC4 | Biliary Cirrhosis, Primary, 4 |
| PBC5 | Biliary Cirrhosis, Primary, 5 |
| CLLS1 | Leukemia, Chronic Lymphocytic, Susceptibility To, 1 |
| CLLS2 | Disrupted In B-Cell Neoplasia |
| CLLS3 | Leukemia, Chronic Lymphocytic, Susceptibility To, 3 |
| CLLS4 | Leukemia, Chronic Lymphocytic Susceptibility To, 4 |
| CLLS5 | Leukemia, Chronic Lymphocytic Susceptiblity To, 5 |
| CILD8 | Ciliary Dyskinesia, Primary, 8 |
| GRD1 | Graves Disease, Susceptiblity To, 1 |
| HPC15 | Prostate Cancer, Hereditary, 15 |
| HPC6 | Prostate Cancer, Susceptibility To |
| HPC7 | Prostate Cancer, Hereditary, 7 |
| RWS | Ragweed Sensitivity |
| LPRS6 | Leprosy, Susceptiblity To, 6 |
| LOC112806077 | Sharpr-MPRA Regulatory Region 13820 |
| AA2 | Alopecia Areata 2 |
| LOC107303343 | Adenosine Deaminase Intronic Regulatory Elements |
| TST1 | Tuberculin Skin Test Reactivity, Absence Of |
| TST2 | Tuberculin Skin Test Reactivity QTL |
| STIM2 | Stromal Interaction Molecule 2 |
| SLC30A4 | Solute Carrier Family 30 Member 4 |
| C1QTNF6 | C1q And TNF Related 6 |
| ZBTB7A | Zinc Finger And BTB Domain Containing 7A |
| DEFB116 | Defensin Beta 116 |
| DEFB112 | Defensin Beta 112 |
| DEFB113 | Defensin Beta 113 |
| DEFB115 | Defensin Beta 115 |
| DEFB135 | Defensin Beta 135 |
| SKAP1 | Src Kinase Associated Phosphoprotein 1 |
| EMD | Emerin |
| RPS12 | Ribosomal Protein S12 |
| CRACR2A | Calcium Release Activated Channel Regulator 2A |
| KCNJ10 | Potassium Inwardly Rectifying Channel Subfamily J Member 10 |
| TACC3 | Transforming Acidic Coiled-Coil Containing Protein 3 |
| IGF2BP2 | Insulin Like Growth Factor 2 MRNA Binding Protein 2 |
| LAMC1 | Laminin Subunit Gamma 1 |
| UBE2G2 | Ubiquitin Conjugating Enzyme E2 G2 |
| UBE3C | Ubiquitin Protein Ligase E3C |
| RAB14 | RAB14, Member RAS Oncogene Family |
| RLIM | Ring Finger Protein, LIM Domain Interacting |
| SNAI1 | Snail Family Transcriptional Repressor 1 |
| PLSCR1 | Phospholipid Scramblase 1 |
| CHRNB4 | Cholinergic Receptor Nicotinic Beta 4 Subunit |
| GLE1 | GLE1 RNA Export Mediator |
| PNISR | PNN Interacting Serine And Arginine Rich Protein |
| PPP6R3 | Protein Phosphatase 6 Regulatory Subunit 3 |
| UBXN1 | UBX Domain Protein 1 |
| KRI1 | KRI1 Homolog |
| PRKCH | Protein Kinase C Eta |
| CUX1 | Cut Like Homeobox 1 |
| FDFT1 | Farnesyl-Diphosphate Farnesyltransferase 1 |
| PTGER3 | Prostaglandin E Receptor 3 |
| COX10 | Cytochrome C Oxidase Assembly Factor Heme A:Farnesyltransferase COX10 |
| MATN3 | Matrilin 3 |
| TUB | TUB Bipartite Transcription Factor |
| STOM | Stomatin |
| PTPN13 | Protein Tyrosine Phosphatase Non-Receptor Type 13 |
| COPS5 | COP9 Signalosome Subunit 5 |
| SEC13 | SEC13 Homolog, Nuclear Pore And COPII Coat Complex Component |
| VRK3 | VRK Serine/Threonine Kinase 3 |
| CKAP4 | Cytoskeleton Associated Protein 4 |
| COMMD9 | COMM Domain Containing 9 |
| PIGC | Phosphatidylinositol Glycan Anchor Biosynthesis Class C |
| MYOM1 | Myomesin 1 |
| C1orf105 | Chromosome 1 Open Reading Frame 105 |
| TRIM10 | Tripartite Motif Containing 10 |
| CD82 | CD82 Molecule |
| LGALS4 | Galectin 4 |
| NME2 | NME/NM23 Nucleoside Diphosphate Kinase 2 |
| ASB2 | Ankyrin Repeat And SOCS Box Containing 2 |
| IBTK | Inhibitor Of Bruton Tyrosine Kinase |
| CBFA2T2 | CBFA2/RUNX1 Partner Transcriptional Co-Repressor 2 |
| COPS7A | COP9 Signalosome Subunit 7A |
| AGBL2 | ATP/GTP Binding Protein Like 2 |
| LINC00328 | Long Intergenic Non-Protein Coding RNA 328 |
| ATP1A1 | ATPase Na+/K+ Transporting Subunit Alpha 1 |
| COPB2 | COPI Coat Complex Subunit Beta 2 |
| RBM15 | RNA Binding Motif Protein 15 |
| PI16 | Peptidase Inhibitor 16 |
| PPP2R5E | Protein Phosphatase 2 Regulatory Subunit B'Epsilon |
| PTGES | Prostaglandin E Synthase |
| PNPLA3 | Patatin Like Phospholipase Domain Containing 3 |
| PLRG1 | Pleiotropic Regulator 1 |
| INS-IGF2 | INS-IGF2 Readthrough |
| SNHG1 | Small Nucleolar RNA Host Gene 1 |
| CACYBP | Calcyclin Binding Protein |
| SH2B1 | SH2B Adaptor Protein 1 |
| MAN1B1 | Mannosidase Alpha Class 1B Member 1 |
| HOXD13 | Homeobox D13 |
| MANEA | Mannosidase Endo-Alpha |
| EIF3M | Eukaryotic Translation Initiation Factor 3 Subunit M |
| PAEP | Progestagen Associated Endometrial Protein |
| SLCO1C1 | Solute Carrier Organic Anion Transporter Family Member 1C1 |
| SLC27A2 | Solute Carrier Family 27 Member 2 |
| ATP6AP2 | ATPase H+ Transporting Accessory Protein 2 |
| IQGAP2 | IQ Motif Containing GTPase Activating Protein 2 |
| RAB18 | RAB18, Member RAS Oncogene Family |
| MIB2 | Mindbomb E3 Ubiquitin Protein Ligase 2 |
| FBXO6 | F-Box Protein 6 |
| VPS35L | VPS35 Endosomal Protein Sorting Factor Like |
| CAMK2B | Calcium/Calmodulin Dependent Protein Kinase II Beta |
| RDH11 | Retinol Dehydrogenase 11 |
| SEH1L | SEH1 Like Nucleoporin |
| DCTN1 | Dynactin Subunit 1 |
| ASB1 | Ankyrin Repeat And SOCS Box Containing 1 |
| SLC2A5 | Solute Carrier Family 2 Member 5 |
| DNAJC13 | DnaJ Heat Shock Protein Family (Hsp40) Member C13 |
| RASA3 | RAS P21 Protein Activator 3 |
| SPTBN4 | Spectrin Beta, Non-Erythrocytic 4 |
| RASA4 | RAS P21 Protein Activator 4 |
| SPRED3 | Sprouty Related EVH1 Domain Containing 3 |
| RASAL3 | RAS Protein Activator Like 3 |
| BUD23 | BUD23 RRNA Methyltransferase And Ribosome Maturation Factor |
| SLC34A3 | Solute Carrier Family 34 Member 3 |
| PRRT2 | Proline Rich Transmembrane Protein 2 |
| USH2A | Usherin |
| COMMD1 | Copper Metabolism Domain Containing 1 |
| IGAT | Immune Response To Synthetic Polypeptide--IRGAT |
| ISCW | Immune Suppression To Streptococcal Antigen |
| CAMKK2 | Calcium/Calmodulin Dependent Protein Kinase Kinase 2 |
| CFL2 | Cofilin 2 |
| MYL9 | Myosin Light Chain 9 |
| MYL12B | Myosin Light Chain 12B |
| MAPKAPK3 | MAPK Activated Protein Kinase 3 |
| SIPA1 | Signal-Induced Proliferation-Associated 1 |
| DYNLL2 | Dynein Light Chain LC8-Type 2 |
| ATAD3B | ATPase Family AAA Domain Containing 3B |
| MUC19 | Mucin 19, Oligomeric |
| TRPA1 | Transient Receptor Potential Cation Channel Subfamily A Member 1 |
| PLPP6 | Phospholipid Phosphatase 6 |
| CASP6 | Caspase 6 |
| CLDN8 | Claudin 8 |
| SLC19A2 | Solute Carrier Family 19 Member 2 |
| FARP2 | FERM, ARH/RhoGEF And Pleckstrin Domain Protein 2 |
| B4GALNT2 | Beta-1,4-N-Acetyl-Galactosaminyltransferase 2 |
| RBM38 | RNA Binding Motif Protein 38 |
| HSD17B12 | Hydroxysteroid 17-Beta Dehydrogenase 12 |
| CCL4L2 | C-C Motif Chemokine Ligand 4 Like 2 |
| SYNJ1 | Synaptojanin 1 |
| UHRF1 | Ubiquitin Like With PHD And Ring Finger Domains 1 |
| NPL | N-Acetylneuraminate Pyruvate Lyase |
| EDC4 | Enhancer Of MRNA Decapping 4 |
| PAF1 | PAF1 Homolog, Paf1/RNA Polymerase II Complex Component |
| NR5A1 | Nuclear Receptor Subfamily 5 Group A Member 1 |
| PROKR2 | Prokineticin Receptor 2 |
| SEMA6A | Semaphorin 6A |
| EML4 | EMAP Like 4 |
| XAF1 | XIAP Associated Factor 1 |
| CLEC2A | C-Type Lectin Domain Family 2 Member A |
| GRB14 | Growth Factor Receptor Bound Protein 14 |
| CLDN18 | Claudin 18 |
| BARD1 | BRCA1 Associated RING Domain 1 |
| LAX1 | Lymphocyte Transmembrane Adaptor 1 |
| RNF41 | Ring Finger Protein 41 |
| STK11IP | Serine/Threonine Kinase 11 Interacting Protein |
| DOK1 | Docking Protein 1 |
| CA4 | Carbonic Anhydrase 4 |
| MIR649 | MicroRNA 649 |
| LEFTY2 | Left-Right Determination Factor 2 |
| SEMA4B | Semaphorin 4B |
| KIF2C | Kinesin Family Member 2C |
| GFRA2 | GDNF Family Receptor Alpha 2 |
| TRIM45 | Tripartite Motif Containing 45 |
| RNASE8 | Ribonuclease A Family Member 8 |
| AMBRA1 | Autophagy And Beclin 1 Regulator 1 |
| CLDN4 | Claudin 4 |
| NDFIP1 | Nedd4 Family Interacting Protein 1 |
| TMED1 | Transmembrane P24 Trafficking Protein 1 |
| GPR108 | G Protein-Coupled Receptor 108 |
| DLD | Dihydrolipoamide Dehydrogenase |
| GPX3 | Glutathione Peroxidase 3 |
| ABCB7 | ATP Binding Cassette Subfamily B Member 7 |
| ANXA3 | Annexin A3 |
| IKZF4 | IKAROS Family Zinc Finger 4 |
| CYRIB | CYFIP Related Rac1 Interactor B |
| MAX | MYC Associated Factor X |
| ANXA6 | Annexin A6 |
| UCN | Urocortin |
| CAMK2A | Calcium/Calmodulin Dependent Protein Kinase II Alpha |
| CENPC | Centromere Protein C |
| BLMH | Bleomycin Hydrolase |
| DAPP1 | Dual Adaptor Of Phosphotyrosine And 3-Phosphoinositides 1 |
| UBE2E3 | Ubiquitin Conjugating Enzyme E2 E3 |
| YWHAB | Tyrosine 3-Monooxygenase/Tryptophan 5-Monooxygenase Activation Protein Beta |
| FEZF2 | FEZ Family Zinc Finger 2 |
| PDE4B | Phosphodiesterase 4B |
| UBE2H | Ubiquitin Conjugating Enzyme E2 H |
| UBR1 | Ubiquitin Protein Ligase E3 Component N-Recognin 1 |
| CYFIP1 | Cytoplasmic FMR1 Interacting Protein 1 |
| ENAH | ENAH Actin Regulator |
| DPP7 | Dipeptidyl Peptidase 7 |
| LRSAM1 | Leucine Rich Repeat And Sterile Alpha Motif Containing 1 |
| DZIP3 | DAZ Interacting Zinc Finger Protein 3 |
| HECTD2 | HECT Domain E3 Ubiquitin Protein Ligase 2 |
| ZFP36L2 | ZFP36 Ring Finger Protein Like 2 |
| RC3H2 | Ring Finger And CCCH-Type Domains 2 |
| ABCG1 | ATP Binding Cassette Subfamily G Member 1 |
| CDKN2B-AS1 | CDKN2B Antisense RNA 1 |
| MIR149 | MicroRNA 149 |
| LIPC | Lipase C, Hepatic Type |
| HSD3B1 | Hydroxy-Delta-5-Steroid Dehydrogenase, 3 Beta- And Steroid Delta-Isomerase 1 |
| NOP56 | NOP56 Ribonucleoprotein |
| RCN1 | Reticulocalbin 1 |
| GID4 | GID Complex Subunit 4 Homolog |
| TRIM35 | Tripartite Motif Containing 35 |
| RUVBL2 | RuvB Like AAA ATPase 2 |
| C9orf72 | C9orf72-SMCR8 Complex Subunit |
| ANGPTL6 | Angiopoietin Like 6 |
| LHB | Luteinizing Hormone Subunit Beta |
| HMGA1 | High Mobility Group AT-Hook 1 |
| CYP11B1 | Cytochrome P450 Family 11 Subfamily B Member 1 |
| NUP50 | Nucleoporin 50 |
| POM121C | POM121 Transmembrane Nucleoporin C |
| NUP58 | Nucleoporin 58 |
| RBM4 | RNA Binding Motif Protein 4 |
| TPM4 | Tropomyosin 4 |
| PTGES2 | Prostaglandin E Synthase 2 |
| DEFB134 | Defensin Beta 134 |
| DEFB133 | Defensin Beta 133 |
| TSC22D3 | TSC22 Domain Family Member 3 |
| IFT46 | Intraflagellar Transport 46 |
| GDF2 | Growth Differentiation Factor 2 |
| DDX6 | DEAD-Box Helicase 6 |
| P2RX1 | Purinergic Receptor P2X 1 |
| TNFRSF10C | TNF Receptor Superfamily Member 10c |
| ALAD | Aminolevulinate Dehydratase |
| UBE2C | Ubiquitin Conjugating Enzyme E2 C |
| RAB5C | RAB5C, Member RAS Oncogene Family |
| SHC2 | SHC Adaptor Protein 2 |
| KCNJ5 | Potassium Inwardly Rectifying Channel Subfamily J Member 5 |
| EXOSC5 | Exosome Component 5 |
| LCOR | Ligand Dependent Nuclear Receptor Corepressor |
| RIDA | Reactive Intermediate Imine Deaminase A Homolog |
| ERN1 | Endoplasmic Reticulum To Nucleus Signaling 1 |
| NFKBIZ | NFKB Inhibitor Zeta |
| SERPINB5 | Serpin Family B Member 5 |
| MIR152 | MicroRNA 152 |
| LPCAT1 | Lysophosphatidylcholine Acyltransferase 1 |
| CUL4A | Cullin 4A |
| DHX33 | DEAH-Box Helicase 33 |
| TRAP1 | TNF Receptor Associated Protein 1 |
| PHLPP1 | PH Domain And Leucine Rich Repeat Protein Phosphatase 1 |
| KIRREL3 | Kirre Like Nephrin Family Adhesion Molecule 3 |
| CKM | Creatine Kinase, M-Type |
| MSLN | Mesothelin |
| ACVR1 | Activin A Receptor Type 1 |
| AGTR2 | Angiotensin II Receptor Type 2 |
| GPD2 | Glycerol-3-Phosphate Dehydrogenase 2 |
| CRHBP | Corticotropin Releasing Hormone Binding Protein |
| RALA | RAS Like Proto-Oncogene A |
| CORO1C | Coronin 1C |
| PBX1 | PBX Homeobox 1 |
| SAFB2 | Scaffold Attachment Factor B2 |
| DEFB108B | Defensin Beta 108B |
| RUBCN | Rubicon Autophagy Regulator |
| TPR | Translocated Promoter Region, Nuclear Basket Protein |
| SLC5A2 | Solute Carrier Family 5 Member 2 |
| HSD17B11 | Hydroxysteroid 17-Beta Dehydrogenase 11 |
| PTF1A | Pancreas Associated Transcription Factor 1a |
| CHD9 | Chromodomain Helicase DNA Binding Protein 9 |
| LRRK1 | Leucine Rich Repeat Kinase 1 |
| WDR62 | WD Repeat Domain 62 |
| CCDC154 | Coiled-Coil Domain Containing 154 |
| PAXX | PAXX Non-Homologous End Joining Factor |
| DLEU1 | Deleted In Lymphocytic Leukemia 1 |
| HLA-DQB3 | Major Histocompatibility Complex, Class II, DQ Beta 3 |
| TGFBI | Transforming Growth Factor Beta Induced |
| CHKA | Choline Kinase Alpha |
| HSD17B8 | Hydroxysteroid 17-Beta Dehydrogenase 8 |
| TIAM2 | TIAM Rac1 Associated GEF 2 |
| XPO7 | Exportin 7 |
| CNOT11 | CCR4-NOT Transcription Complex Subunit 11 |
| XIST | X Inactive Specific Transcript |
| EHMT2 | Euchromatic Histone Lysine Methyltransferase 2 |
| G6PC2 | Glucose-6-Phosphatase Catalytic Subunit 2 |
| PPARGC1B | PPARG Coactivator 1 Beta |
| ADIPOR2 | Adiponectin Receptor 2 |
| CYP20A1 | Cytochrome P450 Family 20 Subfamily A Member 1 |
| LARS1 | Leucyl-TRNA Synthetase 1 |
| HCLS1 | Hematopoietic Cell-Specific Lyn Substrate 1 |
| KMT2C | Lysine Methyltransferase 2C |
| ORC3 | Origin Recognition Complex Subunit 3 |
| RDH12 | Retinol Dehydrogenase 12 |
| CNTFR | Ciliary Neurotrophic Factor Receptor |
| SH3GL2 | SH3 Domain Containing GRB2 Like 2, Endophilin A1 |
| KIF2A | Kinesin Family Member 2A |
| IST1 | IST1 Factor Associated With ESCRT-III |
| PPP1CB | Protein Phosphatase 1 Catalytic Subunit Beta |
| CSRP2 | Cysteine And Glycine Rich Protein 2 |
| PGAM5 | PGAM Family Member 5, Mitochondrial Serine/Threonine Protein Phosphatase |
| CD164 | CD164 Molecule |
| SCAP | SREBF Chaperone |
| PIP4K2A | Phosphatidylinositol-5-Phosphate 4-Kinase Type 2 Alpha |
| LRP8 | LDL Receptor Related Protein 8 |
| SEC14L1 | SEC14 Like Lipid Binding 1 |
| SCART1 | Scavenger Receptor Family Member Expressed On T Cells 1 |
| DEFB121 | Defensin Beta 121 |
| PIK3AP1 | Phosphoinositide-3-Kinase Adaptor Protein 1 |
| BTC | Betacellulin |
| NRG3 | Neuregulin 3 |
| FGF20 | Fibroblast Growth Factor 20 |
| CLDN23 | Claudin 23 |
| CLDN20 | Claudin 20 |
| AP1B1 | Adaptor Related Protein Complex 1 Subunit Beta 1 |
| AP1G1 | Adaptor Related Protein Complex 1 Subunit Gamma 1 |
| AKR1C3 | Aldo-Keto Reductase Family 1 Member C3 |
| SLC39A7 | Solute Carrier Family 39 Member 7 |
| MFAP4 | Microfibril Associated Protein 4 |
| MAZ | MYC Associated Zinc Finger Protein |
| COPS3 | COP9 Signalosome Subunit 3 |
| PSMG1 | Proteasome Assembly Chaperone 1 |
| GATD3A | Glutamine Amidotransferase Like Class 1 Domain Containing 3A |
| PIAS4 | Protein Inhibitor Of Activated STAT 4 |
| SSTR4 | Somatostatin Receptor 4 |
| SLC39A10 | Solute Carrier Family 39 Member 10 |
| SLC11A2 | Solute Carrier Family 11 Member 2 |
| UTS2 | Urotensin 2 |
| H1-0 | H1.0 Linker Histone |
| MIR199B | MicroRNA 199b |
| PAPPA | Pappalysin 1 |
| CAMK1 | Calcium/Calmodulin Dependent Protein Kinase I |
| EPN1 | Epsin 1 |
| LSP1 | Lymphocyte Specific Protein 1 |
| KIR3DL3 | Killer Cell Immunoglobulin Like Receptor, Three Ig Domains And Long Cytoplasmic Tail 3 |
| HTR1B | 5-Hydroxytryptamine Receptor 1B |
| MTRR | 5-Methyltetrahydrofolate-Homocysteine Methyltransferase Reductase |
| OGG1 | 8-Oxoguanine DNA Glycosylase |
| HTT | Huntingtin |
| HK2 | Hexokinase 2 |
| NEFH | Neurofilament Heavy |
| COX4I1 | Cytochrome C Oxidase Subunit 4I1 |
| TECR | Trans-2,3-Enoyl-CoA Reductase |
| CCNE2 | Cyclin E2 |
| EXOC7 | Exocyst Complex Component 7 |
| DCAF8 | DDB1 And CUL4 Associated Factor 8 |
| MDN1 | Midasin AAA ATPase 1 |
| MKX | Mohawk Homeobox |
| NMRAL1 | NmrA Like Redox Sensor 1 |
| GCN1 | GCN1 Activator Of EIF2AK4 |
| MIRLET7I | MicroRNA Let-7i |
| RHOF | Ras Homolog Family Member F, Filopodia Associated |
| ARTN | Artemin |
| CNKSR2 | Connector Enhancer Of Kinase Suppressor Of Ras 2 |
| CKAP5 | Cytoskeleton Associated Protein 5 |
| FOXA1 | Forkhead Box A1 |
| BMPR1B | Bone Morphogenetic Protein Receptor Type 1B |
| PCBD1 | Pterin-4 Alpha-Carbinolamine Dehydratase 1 |
| ADRA2C | Adrenoceptor Alpha 2C |
| ACADS | Acyl-CoA Dehydrogenase Short Chain |
| HS6ST1 | Heparan Sulfate 6-O-Sulfotransferase 1 |
| PPP1R14A | Protein Phosphatase 1 Regulatory Inhibitor Subunit 14A |
| REG4 | Regenerating Family Member 4 |
| NCOA4 | Nuclear Receptor Coactivator 4 |
| NSMF | NMDA Receptor Synaptonuclear Signaling And Neuronal Migration Factor |
| CHAF1A | Chromatin Assembly Factor 1 Subunit A |
| MYO5C | Myosin VC |
| XPO6 | Exportin 6 |
| SNX14 | Sorting Nexin 14 |
| PRPS1L1 | Phosphoribosyl Pyrophosphate Synthetase 1 Like 1 |
| PRICKLE4 | Prickle Planar Cell Polarity Protein 4 |
| HIC2 | HIC ZBTB Transcriptional Repressor 2 |
| YPEL2 | Yippee Like 2 |
| SPINK9 | Serine Peptidase Inhibitor Kazal Type 9 |
| TDRG1 | Testis Development Related 1 |
| SNHG5 | Small Nucleolar RNA Host Gene 5 |
| MIR339 | MicroRNA 339 |
| PPP2R3C | Protein Phosphatase 2 Regulatory Subunit B''Gamma |
| DEFB125 | Defensin Beta 125 |
| DEFB123 | Defensin Beta 123 |
| DEFB129 | Defensin Beta 129 |
| CLCN5 | Chloride Voltage-Gated Channel 5 |
| ZP3 | Zona Pellucida Glycoprotein 3 |
| MSRB1 | Methionine Sulfoxide Reductase B1 |
| CEACAM20 | CEA Cell Adhesion Molecule 20 |
| SSTR1 | Somatostatin Receptor 1 |
| SPOCK3 | SPARC (Osteonectin), Cwcv And Kazal Like Domains Proteoglycan 3 |
| IPO7 | Importin 7 |
| SAT1 | Spermidine/Spermine N1-Acetyltransferase 1 |
| CCKAR | Cholecystokinin A Receptor |
| THBS4 | Thrombospondin 4 |
| VAMP4 | Vesicle Associated Membrane Protein 4 |
| RECQL | RecQ Like Helicase |
| STX5 | Syntaxin 5 |
| NAPA | NSF Attachment Protein Alpha |
| ILVBL | IlvB Acetolactate Synthase Like |
| SMPD4 | Sphingomyelin Phosphodiesterase 4 |
| DPY30 | Dpy-30 Histone Methyltransferase Complex Regulatory Subunit |
| C1RL | Complement C1r Subcomponent Like |
| ARHGDIA | Rho GDP Dissociation Inhibitor Alpha |
| EPPK1 | Epiplakin 1 |
| PXDN | Peroxidasin |
| ANTXR1 | ANTXR Cell Adhesion Molecule 1 |
| ALDH3A1 | Aldehyde Dehydrogenase 3 Family Member A1 |
| MBD1 | Methyl-CpG Binding Domain Protein 1 |
| TCHH | Trichohyalin |
| MARCHF1 | Membrane Associated Ring-CH-Type Finger 1 |
| PRKACB | Protein Kinase CAMP-Activated Catalytic Subunit Beta |
| HIVEP3 | HIVEP Zinc Finger 3 |
| SLURP1 | Secreted LY6/PLAUR Domain Containing 1 |
| CTF1 | Cardiotrophin 1 |
| FAT1 | FAT Atypical Cadherin 1 |
| SSC4D | Scavenger Receptor Cysteine Rich Family Member With 4 Domains |
| RAP1GAP | RAP1 GTPase Activating Protein |
| BIN2 | Bridging Integrator 2 |
| ARIH2 | Ariadne RBR E3 Ubiquitin Protein Ligase 2 |
| CKB | Creatine Kinase B |
| POLR2A | RNA Polymerase II Subunit A |
| PFKFB2 | 6-Phosphofructo-2-Kinase/Fructose-2,6-Biphosphatase 2 |
| SH3RF1 | SH3 Domain Containing Ring Finger 1 |
| PRDX2 | Peroxiredoxin 2 |
| KCND2 | Potassium Voltage-Gated Channel Subfamily D Member 2 |
| PNKD | PNKD Metallo-Beta-Lactamase Domain Containing |
| EFTUD2 | Elongation Factor Tu GTP Binding Domain Containing 2 |
| SLC22A12 | Solute Carrier Family 22 Member 12 |
| ACAD9 | Acyl-CoA Dehydrogenase Family Member 9 |
| EIF2B3 | Eukaryotic Translation Initiation Factor 2B Subunit Gamma |
| PANK4 | Pantothenate Kinase 4 (Inactive) |
| NT5C3A | 5'-Nucleotidase, Cytosolic IIIA |
| OLIG2 | Oligodendrocyte Transcription Factor 2 |
| ARCN1 | Archain 1 |
| PIGG | Phosphatidylinositol Glycan Anchor Biosynthesis Class G |
| LCLAT1 | Lysocardiolipin Acyltransferase 1 |
| SMC5 | Structural Maintenance Of Chromosomes 5 |
| SPAG17 | Sperm Associated Antigen 17 |
| MRPL45 | Mitochondrial Ribosomal Protein L45 |
| MS4A4A | Membrane Spanning 4-Domains A4A |
| GPR89A | G Protein-Coupled Receptor 89A |
| H2AW | H2A.W Histone |
| VPS26C | VPS26 Endosomal Protein Sorting Factor C |
| ZDHHC6 | Zinc Finger DHHC-Type Palmitoyltransferase 6 |
| RCAN3 | RCAN Family Member 3 |
| GCNT2 | Glucosaminyl (N-Acetyl) Transferase 2 (I Blood Group) |
| SMAD6 | SMAD Family Member 6 |
| KLRA1P | Killer Cell Lectin Like Receptor A1, Pseudogene |
| XCR1 | X-C Motif Chemokine Receptor 1 |
| FBXW4 | F-Box And WD Repeat Domain Containing 4 |
| RARG | Retinoic Acid Receptor Gamma |
| MYLK3 | Myosin Light Chain Kinase 3 |
| APOBEC4 | Apolipoprotein B MRNA Editing Enzyme Catalytic Polypeptide Like 4 |
| BTN2A1 | Butyrophilin Subfamily 2 Member A1 |
| PRG3 | Proteoglycan 3, Pro Eosinophil Major Basic Protein 2 |
| DEFB132 | Defensin Beta 132 |
| GLYCAM1 | Glycosylation Dependent Cell Adhesion Molecule 1 (Pseudogene) |
| ATP2A2 | ATPase Sarcoplasmic/Endoplasmic Reticulum Ca2+ Transporting 2 |
| SELENOP | Selenoprotein P |
| ACE2 | Angiotensin I Converting Enzyme 2 |
| SLC5A6 | Solute Carrier Family 5 Member 6 |
| EGLN2 | Egl-9 Family Hypoxia Inducible Factor 2 |
| VAMP2 | Vesicle Associated Membrane Protein 2 |
| SERPINE2 | Serpin Family E Member 2 |
| GDAP1 | Ganglioside Induced Differentiation Associated Protein 1 |
| PTCHD1 | Patched Domain Containing 1 |
| EPGN | Epithelial Mitogen |
| ADCY8 | Adenylate Cyclase 8 |
| TRIP12 | Thyroid Hormone Receptor Interactor 12 |
| ADAM9 | ADAM Metallopeptidase Domain 9 |
| PNOC | Prepronociceptin |
| PELI2 | Pellino E3 Ubiquitin Protein Ligase Family Member 2 |
| EIF4E2 | Eukaryotic Translation Initiation Factor 4E Family Member 2 |
| KCND3 | Potassium Voltage-Gated Channel Subfamily D Member 3 |
| CEP128 | Centrosomal Protein 128 |
| ANG | Angiogenin |
| ADCY9 | Adenylate Cyclase 9 |
| FBXO7 | F-Box Protein 7 |
| HSD17B1 | Hydroxysteroid 17-Beta Dehydrogenase 1 |
| M6PR | Mannose-6-Phosphate Receptor, Cation Dependent |
| UCP3 | Uncoupling Protein 3 |
| GART | Phosphoribosylglycinamide Formyltransferase, Phosphoribosylglycinamide Synthetase, Phosphoribosylaminoimidazole Synthetase |
| SERPINA12 | Serpin Family A Member 12 |
| ABCF1 | ATP Binding Cassette Subfamily F Member 1 |
| SIM2 | SIM BHLH Transcription Factor 2 |
| OBSL1 | Obscurin Like Cytoskeletal Adaptor 1 |
| PLPPR3 | Phospholipid Phosphatase Related 3 |
| KIFBP | Kinesin Family Binding Protein |
| ARIH1 | Ariadne RBR E3 Ubiquitin Protein Ligase 1 |
| MCM6 | Minichromosome Maintenance Complex Component 6 |
| S100A4 | S100 Calcium Binding Protein A4 |
| CTAG2 | Cancer/Testis Antigen 2 |
| LDHA | Lactate Dehydrogenase A |
| USP15 | Ubiquitin Specific Peptidase 15 |
| APOBEC3A_B | APOBEC3A And APOBEC3B Deletion Hybrid |
| DNM3 | Dynamin 3 |
| FLOT1 | Flotillin 1 |
| UGGT1 | UDP-Glucose Glycoprotein Glucosyltransferase 1 |
| PPP2R5B | Protein Phosphatase 2 Regulatory Subunit B'Beta |
| PHLPP2 | PH Domain And Leucine Rich Repeat Protein Phosphatase 2 |
| PIP4K2C | Phosphatidylinositol-5-Phosphate 4-Kinase Type 2 Gamma |
| CLDN10 | Claudin 10 |
| FGF18 | Fibroblast Growth Factor 18 |
| NRG2 | Neuregulin 2 |
| NRG4 | Neuregulin 4 |
| FGF6 | Fibroblast Growth Factor 6 |
| FGF16 | Fibroblast Growth Factor 16 |
| PSMA8 | Proteasome 20S Subunit Alpha 8 |
| CLDN15 | Claudin 15 |
| CLDN17 | Claudin 17 |
| CLDN22 | Claudin 22 |
| HDAC11 | Histone Deacetylase 11 |
| VIPR2 | Vasoactive Intestinal Peptide Receptor 2 |
| P2RY13 | Purinergic Receptor P2Y13 |
| PDZK1 | PDZ Domain Containing 1 |
| TOM1L2 | Target Of Myb1 Like 2 Membrane Trafficking Protein |
| MMP24 | Matrix Metallopeptidase 24 |
| GPR18 | G Protein-Coupled Receptor 18 |
| SHC3 | SHC Adaptor Protein 3 |
| PRKACG | Protein Kinase CAMP-Activated Catalytic Subunit Gamma |
| ADRA2B | Adrenoceptor Alpha 2B |
| KRT13 | Keratin 13 |
| SLC10A2 | Solute Carrier Family 10 Member 2 |
| KIRREL2 | Kirre Like Nephrin Family Adhesion Molecule 2 |
| UPK1A | Uroplakin 1A |
| ANKLE1 | Ankyrin Repeat And LEM Domain Containing 1 |
| MIR26B | MicroRNA 26b |
| MIR29B2 | MicroRNA 29b-2 |
| MYL6B | Myosin Light Chain 6B |
| PCSK9 | Proprotein Convertase Subtilisin/Kexin Type 9 |
| UBE2J2 | Ubiquitin Conjugating Enzyme E2 J2 |
| FBXO10 | F-Box Protein 10 |
| ASH2L | ASH2 Like, Histone Lysine Methyltransferase Complex Subunit |
| INCENP | Inner Centromere Protein |
| RAB32 | RAB32, Member RAS Oncogene Family |
| CDK11A | Cyclin Dependent Kinase 11A |
| MIR1-1 | MicroRNA 1-1 |
| NUDT6 | Nudix Hydrolase 6 |
| PLS3 | Plastin 3 |
| WSB1 | WD Repeat And SOCS Box Containing 1 |
| NCKAP1 | NCK Associated Protein 1 |
| HNRNPD | Heterogeneous Nuclear Ribonucleoprotein D |
| KIR2DL5B | Killer Cell Immunoglobulin Like Receptor, Two Ig Domains And Long Cytoplasmic Tail 5B |
| TBX3 | T-Box Transcription Factor 3 |
| SLC1A4 | Solute Carrier Family 1 Member 4 |
| SLC1A7 | Solute Carrier Family 1 Member 7 |
| KDM5C | Lysine Demethylase 5C |
| DCTD | DCMP Deaminase |
| FBN2 | Fibrillin 2 |
| SH3BP5 | SH3 Domain Binding Protein 5 |
| SCARF2 | Scavenger Receptor Class F Member 2 |
| POU3F4 | POU Class 3 Homeobox 4 |
| TBX22 | T-Box Transcription Factor 22 |
| KLK14 | Kallikrein Related Peptidase 14 |
| RPH3AL | Rabphilin 3A Like (Without C2 Domains) |
| FRAT1 | FRAT Regulator Of WNT Signaling Pathway 1 |
| AIFM3 | Apoptosis Inducing Factor Mitochondria Associated 3 |
| BFAR | Bifunctional Apoptosis Regulator |
| YBX2 | Y-Box Binding Protein 2 |
| LIPI | Lipase I |
| TBX10 | T-Box Transcription Factor 10 |
| LXN | Latexin |
| GAR1 | GAR1 Ribonucleoprotein |
| ANOS1 | Anosmin 1 |
| TXNDC15 | Thioredoxin Domain Containing 15 |
| WSCD1 | WSC Domain Containing 1 |
| YPEL1 | Yippee Like 1 |
| TSSK2 | Testis Specific Serine Kinase 2 |
| DNAJC28 | DnaJ Heat Shock Protein Family (Hsp40) Member C28 |
| OPALIN | Oligodendrocytic Myelin Paranodal And Inner Loop Protein |
| RFESD | Rieske Fe-S Domain Containing |
| ANKRD50 | Ankyrin Repeat Domain 50 |
| FAM149A | Family With Sequence Similarity 149 Member A |
| RIMBP3B | RIMS Binding Protein 3B |
| DLEU2 | Deleted In Lymphocytic Leukemia 2 |
| DAOA-AS1 | DAOA Antisense RNA 1 |
| MIR28 | MicroRNA 28 |
| MIR659 | MicroRNA 659 |
| DGCR9 | DiGeorge Syndrome Critical Region Gene 9 |
| APELA | Apelin Receptor Early Endogenous Ligand |
| MIR299 | MicroRNA 299 |
| MIR502 | MicroRNA 502 |
| DGCR11 | DiGeorge Syndrome Critical Region Gene 11 |
| CBR3-AS1 | CBR3 Antisense RNA 1 |
| MIR374A | MicroRNA 374a |
| KANSL1-AS1 | KANSL1 Antisense RNA 1 |
| MIR3618 | MicroRNA 3618 |
| FAM230A | Family With Sequence Similarity 230 Member A |
| FAM230B | Family With Sequence Similarity 230 Member B |
| DVL1P1 | Dishevelled Segment Polarity Protein 1 Pseudogene 1 |
| DGCR12 | DiGeorge Syndrome Critical Region Gene 12 |
| LOC102724770 | Protein DGCR6 |
| ENSG00000224836 |  |
| ENSG00000281530 |  |
| LOC116309126 | CRISPRi-Validated Cis-Regulatory Element Chr22.250 |
| LOC108449888 | PATRR22 Recombination Region |
| LOC108449897 | PATRR11 Recombination Region |
| S100A11 | S100 Calcium Binding Protein A11 |
| MEF2A | Myocyte Enhancer Factor 2A |
| PPP1R3A | Protein Phosphatase 1 Regulatory Subunit 3A |
| HMSD | Histocompatibility Minor Serpin Domain Containing |
| NLRP2B | NLR Family Pyrin Domain Containing 2B |
| KIR3DP1 | Killer Cell Immunoglobulin Like Receptor, Three Ig Domains Pseudogene 1 |
| KBTBD7 | Kelch Repeat And BTB Domain Containing 7 |
| MIR7703 | MicroRNA 7703 |
| KCNB1 | Potassium Voltage-Gated Channel Subfamily B Member 1 |
| CA8 | Carbonic Anhydrase 8 |
| NIN | Ninein |
| SPCS1 | Signal Peptidase Complex Subunit 1 |
| DRC3 | Dynein Regulatory Complex Subunit 3 |
| PCGEM1 | PCGEM1 Prostate-Specific Transcript |
| CHKB | Choline Kinase Beta |
| TUBA1B | Tubulin Alpha 1b |
| PTPRU | Protein Tyrosine Phosphatase Receptor Type U |
| SEMA6D | Semaphorin 6D |
| LEMD2 | LEM Domain Nuclear Envelope Protein 2 |
| MMP25 | Matrix Metallopeptidase 25 |
| UBE2J1 | Ubiquitin Conjugating Enzyme E2 J1 |
| DOK3 | Docking Protein 3 |
| RAP1GAP2 | RAP1 GTPase Activating Protein 2 |
| CBR1 | Carbonyl Reductase 1 |
| OPA1 | OPA1 Mitochondrial Dynamin Like GTPase |
| FIS1 | Fission, Mitochondrial 1 |
| EXOC8 | Exocyst Complex Component 8 |
| LUZP1 | Leucine Zipper Protein 1 |
| ARRDC4 | Arrestin Domain Containing 4 |
| PIH1D2 | PIH1 Domain Containing 2 |
| CAVIN2 | Caveolae Associated Protein 2 |
| HBB-LCR | Beta-Globin Locus Control Region |
| PLCL2 | Phospholipase C Like 2 |
| GAB1 | GRB2 Associated Binding Protein 1 |
| SLC29A2 | Solute Carrier Family 29 Member 2 |
| KCNJ8 | Potassium Inwardly Rectifying Channel Subfamily J Member 8 |
| CRLS1 | Cardiolipin Synthase 1 |
| AK7 | Adenylate Kinase 7 |
| ADCK1 | AarF Domain Containing Kinase 1 |
| ALKBH1 | AlkB Homolog 1, Histone H2A Dioxygenase |
| CYB5R3 | Cytochrome B5 Reductase 3 |
| B4GALNT1 | Beta-1,4-N-Acetyl-Galactosaminyltransferase 1 |
| TPSB2 | Tryptase Beta 2 (Gene/Pseudogene) |
| GBP4 | Guanylate Binding Protein 4 |
| E2F2 | E2F Transcription Factor 2 |
| PDCD10 | Programmed Cell Death 10 |
| ADAP1 | ArfGAP With Dual PH Domains 1 |
| ERMAP | Erythroblast Membrane Associated Protein (Scianna Blood Group) |
| CYP2R1 | Cytochrome P450 Family 2 Subfamily R Member 1 |
| AKAP13 | A-Kinase Anchoring Protein 13 |
| USO1 | USO1 Vesicle Transport Factor |
| SAR1B | Secretion Associated Ras Related GTPase 1B |
| XAGE1B | X Antigen Family Member 1B |
| MYLPF | Myosin Light Chain, Phosphorylatable, Fast Skeletal Muscle |
| FAU | FAU Ubiquitin Like And Ribosomal Protein S30 Fusion |
| FUBP1 | Far Upstream Element Binding Protein 1 |
| CCDC124 | Coiled-Coil Domain Containing 124 |
| SMPDL3B | Sphingomyelin Phosphodiesterase Acid Like 3B |
| PENK | Proenkephalin |
| DCST1 | DC-STAMP Domain Containing 1 |
| BTNL9 | Butyrophilin Like 9 |
| JAML | Junction Adhesion Molecule Like |
| HPGDS | Hematopoietic Prostaglandin D Synthase |
| MC3R | Melanocortin 3 Receptor |
| CDC37 | Cell Division Cycle 37 |
| RNF7 | Ring Finger Protein 7 |
| PCSK7 | Proprotein Convertase Subtilisin/Kexin Type 7 |
| RAB35 | RAB35, Member RAS Oncogene Family |
| LSM8 | LSM8 Homolog, U6 Small Nuclear RNA Associated |
| DOCK1 | Dedicator Of Cytokinesis 1 |
| CNN2 | Calponin 2 |
| ZNRF1 | Zinc And Ring Finger 1 |
| PLD4 | Phospholipase D Family Member 4 |
| QSOX1 | Quiescin Sulfhydryl Oxidase 1 |
| TMEM30A | Transmembrane Protein 30A |
| TBC1D10C | TBC1 Domain Family Member 10C |
| FBXO4 | F-Box Protein 4 |
| PLEKHO2 | Pleckstrin Homology Domain Containing O2 |
| FBXL5 | F-Box And Leucine Rich Repeat Protein 5 |
| ADGRG3 | Adhesion G Protein-Coupled Receptor G3 |
| FBXW9 | F-Box And WD Repeat Domain Containing 9 |
| NDUFV2 | NADH:Ubiquinone Oxidoreductase Core Subunit V2 |
| TBCB | Tubulin Folding Cofactor B |
| SOX8 | SRY-Box Transcription Factor 8 |
| MAP6 | Microtubule Associated Protein 6 |
| SYNE1 | Spectrin Repeat Containing Nuclear Envelope Protein 1 |
| PDLIM1 | PDZ And LIM Domain 1 |
| PPAT | Phosphoribosyl Pyrophosphate Amidotransferase |
| CYP27A1 | Cytochrome P450 Family 27 Subfamily A Member 1 |
| FH | Fumarate Hydratase |
| SLC34A1 | Solute Carrier Family 34 Member 1 |
| TULP1 | TUB Like Protein 1 |
| TTPA | Alpha Tocopherol Transfer Protein |
| STIP1 | Stress Induced Phosphoprotein 1 |
| NUDCD1 | NudC Domain Containing 1 |
| PPID | Peptidylprolyl Isomerase D |
| PTPRA | Protein Tyrosine Phosphatase Receptor Type A |
| BRAP | BRCA1 Associated Protein |
| ZBTB1 | Zinc Finger And BTB Domain Containing 1 |
| COLCA1 | Colorectal Cancer Associated 1 |
| GPD1 | Glycerol-3-Phosphate Dehydrogenase 1 |
| RHAG | Rh Associated Glycoprotein |
| TMEM107 | Transmembrane Protein 107 |
| IPO5 | Importin 5 |
| BAMBI | BMP And Activin Membrane Bound Inhibitor |
| SRPK1 | SRSF Protein Kinase 1 |
| ACLY | ATP Citrate Lyase |
| DEFB130A | Defensin Beta 130A |
| GNB5 | G Protein Subunit Beta 5 |
| GNG2 | G Protein Subunit Gamma 2 |
| GNG4 | G Protein Subunit Gamma 4 |
| GNG3 | G Protein Subunit Gamma 3 |
| EIF4A2 | Eukaryotic Translation Initiation Factor 4A2 |
| PPM1B | Protein Phosphatase, Mg2+/Mn2+ Dependent 1B |
| EIF4A3 | Eukaryotic Translation Initiation Factor 4A3 |
| TRIM2 | Tripartite Motif Containing 2 |
| TAC4 | Tachykinin Precursor 4 |
| SSX5 | SSX Family Member 5 |
| RCVRN | Recoverin |
| BMS1 | BMS1 Ribosome Biogenesis Factor |
| PPARGC1A | PPARG Coactivator 1 Alpha |
| FTO | FTO Alpha-Ketoglutarate Dependent Dioxygenase |
| MAGED1 | MAGE Family Member D1 |
| CBX3 | Chromobox 3 |
| NR1I3 | Nuclear Receptor Subfamily 1 Group I Member 3 |
| USP17L2 | Ubiquitin Specific Peptidase 17 Like Family Member 2 |
| PTPA | Protein Phosphatase 2 Phosphatase Activator |
| DMPK | DM1 Protein Kinase |
| BBS2 | Bardet-Biedl Syndrome 2 |
| DYNC2H1 | Dynein Cytoplasmic 2 Heavy Chain 1 |
| ARMC5 | Armadillo Repeat Containing 5 |
| COLEC12 | Collectin Subfamily Member 12 |
| RASAL2 | RAS Protein Activator Like 2 |
| ILF3 | Interleukin Enhancer Binding Factor 3 |
| BIN1 | Bridging Integrator 1 |
| STAU1 | Staufen Double-Stranded RNA Binding Protein 1 |
| HSH2D | Hematopoietic SH2 Domain Containing |
| ENTPD2 | Ectonucleoside Triphosphate Diphosphohydrolase 2 |
| CMAHP | Cytidine Monophospho-N-Acetylneuraminic Acid Hydroxylase, Pseudogene |
| SLC22A2 | Solute Carrier Family 22 Member 2 |
| CDR2L | Cerebellar Degeneration Related Protein 2 Like |
| ERG | ETS Transcription Factor ERG |
| SUV39H1 | Suppressor Of Variegation 3-9 Homolog 1 |
| FBXO11 | F-Box Protein 11 |
| TRPV2 | Transient Receptor Potential Cation Channel Subfamily V Member 2 |
| COLEC10 | Collectin Subfamily Member 10 |
| SRP14 | Signal Recognition Particle 14 |
| AKT1S1 | AKT1 Substrate 1 |
| PPP1R12B | Protein Phosphatase 1 Regulatory Subunit 12B |
| NTAN1 | N-Terminal Asparagine Amidase |
| TACR2 | Tachykinin Receptor 2 |
| UBA3 | Ubiquitin Like Modifier Activating Enzyme 3 |
| KLB | Klotho Beta |
| MIR5193 | MicroRNA 5193 |
| NEDD9 | Neural Precursor Cell Expressed, Developmentally Down-Regulated 9 |
| COPS6 | COP9 Signalosome Subunit 6 |
| HNRNPL | Heterogeneous Nuclear Ribonucleoprotein L |
| POLL | DNA Polymerase Lambda |
| MEPE | Matrix Extracellular Phosphoglycoprotein |
| ETV3 | ETS Variant Transcription Factor 3 |
| TAAR1 | Trace Amine Associated Receptor 1 |
| SLC25A3 | Solute Carrier Family 25 Member 3 |
| CRY1 | Cryptochrome Circadian Regulator 1 |
| ZBTB20 | Zinc Finger And BTB Domain Containing 20 |
| DNASE1L2 | Deoxyribonuclease 1 Like 2 |
| GPX1 | Glutathione Peroxidase 1 |
| S100A1 | S100 Calcium Binding Protein A1 |
| KLC1 | Kinesin Light Chain 1 |
| OSBPL1A | Oxysterol Binding Protein Like 1A |
| NFE2 | Nuclear Factor, Erythroid 2 |
| PF4V1 | Platelet Factor 4 Variant 1 |
| NOP53 | NOP53 Ribosome Biogenesis Factor |
| NRP2 | Neuropilin 2 |
| PLK4 | Polo Like Kinase 4 |
| CUBN | Cubilin |
| GGCX | Gamma-Glutamyl Carboxylase |
| MAK | Male Germ Cell Associated Kinase |
| PEX10 | Peroxisomal Biogenesis Factor 10 |
| ANLN | Anillin Actin Binding Protein |
| FASTKD2 | FAST Kinase Domains 2 |
| IFT80 | Intraflagellar Transport 80 |
| FAM13A | Family With Sequence Similarity 13 Member A |
| TTC21B | Tetratricopeptide Repeat Domain 21B |
| PROK1 | Prokineticin 1 |
| SPON1 | Spondin 1 |
| STK38 | Serine/Threonine Kinase 38 |
| SERPINF2 | Serpin Family F Member 2 |
| ADH1B | Alcohol Dehydrogenase 1B (Class I), Beta Polypeptide |
| IL1RAPL1 | Interleukin 1 Receptor Accessory Protein Like 1 |
| GTF2H4 | General Transcription Factor IIH Subunit 4 |
| ZP1 | Zona Pellucida Glycoprotein 1 |
| CDCP1 | CUB Domain Containing Protein 1 |
| EEF1A2 | Eukaryotic Translation Elongation Factor 1 Alpha 2 |
| GSDMB | Gasdermin B |
| ACSL3 | Acyl-CoA Synthetase Long Chain Family Member 3 |
| RNF115 | Ring Finger Protein 115 |
| PER2 | Period Circadian Regulator 2 |
| ADORA2B | Adenosine A2b Receptor |
| AP2A1 | Adaptor Related Protein Complex 2 Subunit Alpha 1 |
| THRB | Thyroid Hormone Receptor Beta |
| LMLN | Leishmanolysin Like Peptidase |
| MAP3K2 | Mitogen-Activated Protein Kinase Kinase Kinase 2 |
| GLDC | Glycine Decarboxylase |
| CHD5 | Chromodomain Helicase DNA Binding Protein 5 |
| HBD | Hemoglobin Subunit Delta |
| ATOH1 | Atonal BHLH Transcription Factor 1 |
| CHD6 | Chromodomain Helicase DNA Binding Protein 6 |
| CTNNA3 | Catenin Alpha 3 |
| ANXA4 | Annexin A4 |
| CSTB | Cystatin B |
| PLAC8 | Placenta Associated 8 |
| ATP6V0D2 | ATPase H+ Transporting V0 Subunit D2 |
| KLHL21 | Kelch Like Family Member 21 |
| BCL7B | BAF Chromatin Remodeling Complex Subunit BCL7B |
| HINT1 | Histidine Triad Nucleotide Binding Protein 1 |
| PLIN1 | Perilipin 1 |
| GUK1 | Guanylate Kinase 1 |
| TGFB1I1 | Transforming Growth Factor Beta 1 Induced Transcript 1 |
| PLTP | Phospholipid Transfer Protein |
| GNG7 | G Protein Subunit Gamma 7 |
| SMIM3 | Small Integral Membrane Protein 3 |
| SIGLEC6 | Sialic Acid Binding Ig Like Lectin 6 |
| RELT | RELT TNF Receptor |
| TNMD | Tenomodulin |
| DUSP7 | Dual Specificity Phosphatase 7 |
| KSR2 | Kinase Suppressor Of Ras 2 |
| DUSP8 | Dual Specificity Phosphatase 8 |
| FRS3 | Fibroblast Growth Factor Receptor Substrate 3 |
| MIR3614 | MicroRNA 3614 |
| MIR6852 | MicroRNA 6852 |
| MINPP1 | Multiple Inositol-Polyphosphate Phosphatase 1 |
| FOXF1 | Forkhead Box F1 |
| DLEC1 | DLEC1 Cilia And Flagella Associated Protein |
| HYLS1 | HYLS1 Centriolar And Ciliogenesis Associated |
| POU6F2 | POU Class 6 Homeobox 2 |
| MYRF | Myelin Regulatory Factor |
| AKAP8 | A-Kinase Anchoring Protein 8 |
| DDX21 | DExD-Box Helicase 21 |
| ALDH3B1 | Aldehyde Dehydrogenase 3 Family Member B1 |
| GDI2 | GDP Dissociation Inhibitor 2 |
| P4HB | Prolyl 4-Hydroxylase Subunit Beta |
| ERO1A | Endoplasmic Reticulum Oxidoreductase 1 Alpha |
| NDUFS2 | NADH:Ubiquinone Oxidoreductase Core Subunit S2 |
| BMP1 | Bone Morphogenetic Protein 1 |
| RAD23B | RAD23 Homolog B, Nucleotide Excision Repair Protein |
| VDAC1 | Voltage Dependent Anion Channel 1 |
| HTR2C | 5-Hydroxytryptamine Receptor 2C |
| ALDH1A2 | Aldehyde Dehydrogenase 1 Family Member A2 |
| EFNB2 | Ephrin B2 |
| GHRHR | Growth Hormone Releasing Hormone Receptor |
| PFAS | Phosphoribosylformylglycinamidine Synthase |
| MLLT1 | MLLT1 Super Elongation Complex Subunit |
| RALB | RAS Like Proto-Oncogene B |
| MAPKAP1 | MAPK Associated Protein 1 |
| TMCO1 | Transmembrane And Coiled-Coil Domains 1 |
| LDHB | Lactate Dehydrogenase B |
| KCNA2 | Potassium Voltage-Gated Channel Subfamily A Member 2 |
| GLO1 | Glyoxalase I |
| PLAGL1 | PLAG1 Like Zinc Finger 1 |
| GYPA | Glycophorin A (MNS Blood Group) |
| CAMTA1 | Calmodulin Binding Transcription Activator 1 |
| CREB3 | CAMP Responsive Element Binding Protein 3 |
| SLC39A1 | Solute Carrier Family 39 Member 1 |
| TDRD6 | Tudor Domain Containing 6 |
| MIR10B | MicroRNA 10b |
| MIR19B1 | MicroRNA 19b-1 |
| GZMK | Granzyme K |
| MAP1LC3A | Microtubule Associated Protein 1 Light Chain 3 Alpha |
| S1PR5 | Sphingosine-1-Phosphate Receptor 5 |
| PROC | Protein C, Inactivator Of Coagulation Factors Va And VIIIa |
| RPE65 | Retinoid Isomerohydrolase RPE65 |
| KLK11 | Kallikrein Related Peptidase 11 |
| ADGRB1 | Adhesion G Protein-Coupled Receptor B1 |
| NRROS | Negative Regulator Of Reactive Oxygen Species |
| G3BP2 | G3BP Stress Granule Assembly Factor 2 |
| RB1CC1 | RB1 Inducible Coiled-Coil 1 |
| ACTN3 | Actinin Alpha 3 (Gene/Pseudogene) |
| UBA1 | Ubiquitin Like Modifier Activating Enzyme 1 |
| QPCT | Glutaminyl-Peptide Cyclotransferase |
| NID1 | Nidogen 1 |
| PDGFD | Platelet Derived Growth Factor D |
| LEFTY1 | Left-Right Determination Factor 1 |
| PRMT6 | Protein Arginine Methyltransferase 6 |
| TPBG | Trophoblast Glycoprotein |
| TRAM1 | Translocation Associated Membrane Protein 1 |
| WNT1 | Wnt Family Member 1 |
| SLC20A2 | Solute Carrier Family 20 Member 2 |
| SNAI2 | Snail Family Transcriptional Repressor 2 |
| TBC1D4 | TBC1 Domain Family Member 4 |
| E2F3 | E2F Transcription Factor 3 |
| HOXA1 | Homeobox A1 |
| TRIM33 | Tripartite Motif Containing 33 |
| NDUFB8 | NADH:Ubiquinone Oxidoreductase Subunit B8 |
| MCF2L | MCF.2 Cell Line Derived Transforming Sequence Like |
| SPINT1 | Serine Peptidase Inhibitor, Kunitz Type 1 |
| GREM2 | Gremlin 2, DAN Family BMP Antagonist |
| PKNOX1 | PBX/Knotted 1 Homeobox 1 |
| CD248 | CD248 Molecule |
| ARID3B | AT-Rich Interaction Domain 3B |
| GABBR1 | Gamma-Aminobutyric Acid Type B Receptor Subunit 1 |
| UCP1 | Uncoupling Protein 1 |
| PDE8A | Phosphodiesterase 8A |
| ICMT | Isoprenylcysteine Carboxyl Methyltransferase |
| SERPINA4 | Serpin Family A Member 4 |
| NPTX2 | Neuronal Pentraxin 2 |
| NSRP1 | Nuclear Speckle Splicing Regulatory Protein 1 |
| ALKBH5 | AlkB Homolog 5, RNA Demethylase |
| MIR455 | MicroRNA 455 |
| HSF1 | Heat Shock Transcription Factor 1 |
| EHBP1 | EH Domain Binding Protein 1 |
| MIR224 | MicroRNA 224 |
| SDC4 | Syndecan 4 |
| S1PR4 | Sphingosine-1-Phosphate Receptor 4 |
| TINAGL1 | Tubulointerstitial Nephritis Antigen Like 1 |
| PBRM1 | Polybromo 1 |
| VPS4A | Vacuolar Protein Sorting 4 Homolog A |
| RPS27L | Ribosomal Protein S27 Like |
| ING4 | Inhibitor Of Growth Family Member 4 |
| PRRC2B | Proline Rich Coiled-Coil 2B |
| PIAS3 | Protein Inhibitor Of Activated STAT 3 |
| ACAD11 | Acyl-CoA Dehydrogenase Family Member 11 |
| RAVER1 | Ribonucleoprotein, PTB Binding 1 |
| PRKAR2A | Protein Kinase CAMP-Dependent Type II Regulatory Subunit Alpha |
| RALBP1 | RalA Binding Protein 1 |
| TWF1 | Twinfilin Actin Binding Protein 1 |
| HNRNPU | Heterogeneous Nuclear Ribonucleoprotein U |
| RPL39 | Ribosomal Protein L39 |
| NACA | Nascent Polypeptide Associated Complex Subunit Alpha |
| KCNJ1 | Potassium Inwardly Rectifying Channel Subfamily J Member 1 |
| PDE11A | Phosphodiesterase 11A |
| WNT10A | Wnt Family Member 10A |
| PDE6B | Phosphodiesterase 6B |
| CHRNB1 | Cholinergic Receptor Nicotinic Beta 1 Subunit |
| YARS2 | Tyrosyl-TRNA Synthetase 2 |
| CHRND | Cholinergic Receptor Nicotinic Delta Subunit |
| RP2 | RP2 Activator Of ARL3 GTPase |
| ZNF423 | Zinc Finger Protein 423 |
| TACO1 | Translational Activator Of Cytochrome C Oxidase I |
| CRB2 | Crumbs Cell Polarity Complex Component 2 |
| CEP120 | Centrosomal Protein 120 |
| FUZ | Fuzzy Planar Cell Polarity Protein |
| MBD5 | Methyl-CpG Binding Domain Protein 5 |
| IFT27 | Intraflagellar Transport 27 |
| ZNF408 | Zinc Finger Protein 408 |
| POC1B | POC1 Centriolar Protein B |
| COA3 | Cytochrome C Oxidase Assembly Factor 3 |
| DOK2 | Docking Protein 2 |
| ABHD12 | Abhydrolase Domain Containing 12 |
| ADCY1 | Adenylate Cyclase 1 |
| UBE2F | Ubiquitin Conjugating Enzyme E2 F (Putative) |
| POLR2H | RNA Polymerase II Subunit H |
| MS4A3 | Membrane Spanning 4-Domains A3 |
| PTK6 | Protein Tyrosine Kinase 6 |
| GRM5 | Glutamate Metabotropic Receptor 5 |
| ASCL1 | Achaete-Scute Family BHLH Transcription Factor 1 |
| TPP1 | Tripeptidyl Peptidase 1 |
| PDSS1 | Decaprenyl Diphosphate Synthase Subunit 1 |
| RAB1B | RAB1B, Member RAS Oncogene Family |
| NEUROG3 | Neurogenin 3 |
| BCL9 | BCL9 Transcription Coactivator |
| KCNIP2 | Potassium Voltage-Gated Channel Interacting Protein 2 |
| TAF11 | TATA-Box Binding Protein Associated Factor 11 |
| RYBP | RING1 And YY1 Binding Protein |
| NMUR2 | Neuromedin U Receptor 2 |
| ECE2 | Endothelin Converting Enzyme 2 |
| PAPOLG | Poly(A) Polymerase Gamma |
| ELAVL3 | ELAV Like RNA Binding Protein 3 |
| GARS1 | Glycyl-TRNA Synthetase 1 |
| MCTS1 | MCTS1 Re-Initiation And Release Factor |
| TSGA10 | Testis Specific 10 |
| TMEM50B | Transmembrane Protein 50B |
| NKAIN2 | Sodium/Potassium Transporting ATPase Interacting 2 |
| MIR193A | MicroRNA 193a |
| MIR361 | MicroRNA 361 |
| WFDC2 | WAP Four-Disulfide Core Domain 2 |
| SI | Sucrase-Isomaltase |
| ADH4 | Alcohol Dehydrogenase 4 (Class II), Pi Polypeptide |
| ITSN1 | Intersectin 1 |
| POU4F1 | POU Class 4 Homeobox 1 |
| SLBP | Stem-Loop Binding Protein |
| ADGRL1 | Adhesion G Protein-Coupled Receptor L1 |
| NGDN | Neuroguidin |
| TPTE2 | Transmembrane Phosphoinositide 3-Phosphatase And Tensin Homolog 2 |
| PRNCR1 | Prostate Cancer Associated Non-Coding RNA 1 |
| DAB2 | DAB Adaptor Protein 2 |
| MATN1 | Matrilin 1 |
| STAB1 | Stabilin 1 |
| PAX9 | Paired Box 9 |
| SLC2A9 | Solute Carrier Family 2 Member 9 |
| FOXA2 | Forkhead Box A2 |
| CRTC1 | CREB Regulated Transcription Coactivator 1 |
| COL9A1 | Collagen Type IX Alpha 1 Chain |
| CACNB3 | Calcium Voltage-Gated Channel Auxiliary Subunit Beta 3 |
| SCTR | Secretin Receptor |
| PHF8 | PHD Finger Protein 8 |
| PCSK5 | Proprotein Convertase Subtilisin/Kexin Type 5 |
| HOXB9 | Homeobox B9 |
| TRDMT1 | TRNA Aspartic Acid Methyltransferase 1 |
| NEIL1 | Nei Like DNA Glycosylase 1 |
| CBY1 | Chibby Family Member 1, Beta Catenin Antagonist |
| EXOC6 | Exocyst Complex Component 6 |
| ASPM | Abnormal Spindle Microtubule Assembly |
| RBP1 | Retinol Binding Protein 1 |
| NPRL3 | NPR3 Like, GATOR1 Complex Subunit |
| EPC1 | Enhancer Of Polycomb Homolog 1 |
| FBLIM1 | Filamin Binding LIM Protein 1 |
| SOX1 | SRY-Box Transcription Factor 1 |
| GPR174 | G Protein-Coupled Receptor 174 |
| MRAP2 | Melanocortin 2 Receptor Accessory Protein 2 |
| CBSL | Cystathionine Beta-Synthase Like |
| CLPTM1L | CLPTM1 Like |
| CCT3 | Chaperonin Containing TCP1 Subunit 3 |
| CLCF1 | Cardiotrophin Like Cytokine Factor 1 |
| NUP42 | Nucleoporin 42 |
| CGN | Cingulin |
| MARCHF9 | Membrane Associated Ring-CH-Type Finger 9 |
| FFAR1 | Free Fatty Acid Receptor 1 |
| PNMA5 | PNMA Family Member 5 |
| IKZF2 | IKAROS Family Zinc Finger 2 |
| CLEC9A | C-Type Lectin Domain Containing 9A |
| CEACAM7 | CEA Cell Adhesion Molecule 7 |
| ABCC5 | ATP Binding Cassette Subfamily C Member 5 |
| METTL3 | Methyltransferase Like 3 |
| MIR2909 | MicroRNA 2909 |
| A2M | Alpha-2-Macroglobulin |
| MYL12A | Myosin Light Chain 12A |
| GNGT1 | G Protein Subunit Gamma Transducin 1 |
| GNG5 | G Protein Subunit Gamma 5 |
| GNGT2 | G Protein Subunit Gamma Transducin 2 |
| GNG11 | G Protein Subunit Gamma 11 |
| GNG13 | G Protein Subunit Gamma 13 |
| GNG8 | G Protein Subunit Gamma 8 |
| RPS6KA1 | Ribosomal Protein S6 Kinase A1 |
| SEC23A | Sec23 Homolog A, COPII Coat Complex Component |
| DYNC1I1 | Dynein Cytoplasmic 1 Intermediate Chain 1 |
| UBE2O | Ubiquitin Conjugating Enzyme E2 O |
| FBXW5 | F-Box And WD Repeat Domain Containing 5 |
| SERPINB12 | Serpin Family B Member 12 |
| BPIFB2 | BPI Fold Containing Family B Member 2 |
| BPIFB4 | BPI Fold Containing Family B Member 4 |
| TRIM50 | Tripartite Motif Containing 50 |
| FBXL21P | F-Box And Leucine Rich Repeat Protein 21, Pseudogene |
| DEFB109A | Defensin Beta 109A (Pseudogene) |
| TSPAN6 | Tetraspanin 6 |
| GLIPR1L2 | GLIPR1 Like 2 |
| DUSP9 | Dual Specificity Phosphatase 9 |
| ACTR3B | Actin Related Protein 3B |
| RASAL1 | RAS Protein Activator Like 1 |
| PSPN | Persephin |
| LAMTOR3 | Late Endosomal/Lysosomal Adaptor, MAPK And MTOR Activator 3 |
| SPTBN5 | Spectrin Beta, Non-Erythrocytic 5 |
| RASGEF1A | RasGEF Domain Family Member 1A |
| PAQR3 | Progestin And AdipoQ Receptor Family Member 3 |
| GFRA4 | GDNF Family Receptor Alpha 4 |
| MIR718 | MicroRNA 718 |
| MIR5004 | MicroRNA 5004 |
| MIR3652 | MicroRNA 3652 |
| MIR7846 | MicroRNA 7846 |
| MIR6502 | MicroRNA 6502 |
| MIR7855 | MicroRNA 7855 |
| KDM5D | Lysine Demethylase 5D |
| RPL30 | Ribosomal Protein L30 |
| PTGER1 | Prostaglandin E Receptor 1 |
| HSPB8 | Heat Shock Protein Family B (Small) Member 8 |
| ARHGDIB | Rho GDP Dissociation Inhibitor Beta |
| MTDH | Metadherin |
| ASCC2 | Activating Signal Cointegrator 1 Complex Subunit 2 |
| TOP2B | DNA Topoisomerase II Beta |
| SPAG9 | Sperm Associated Antigen 9 |
| FBXL20 | F-Box And Leucine Rich Repeat Protein 20 |
| CANT1 | Calcium Activated Nucleotidase 1 |
| CYP2B6 | Cytochrome P450 Family 2 Subfamily B Member 6 |
| H3C2 | H3 Clustered Histone 2 |
| KHSRP | KH-Type Splicing Regulatory Protein |
| PDIA6 | Protein Disulfide Isomerase Family A Member 6 |
| CPT1A | Carnitine Palmitoyltransferase 1A |
| PPP2R2B | Protein Phosphatase 2 Regulatory Subunit Bbeta |
| JMJD6 | Jumonji Domain Containing 6, Arginine Demethylase And Lysine Hydroxylase |
| STOML2 | Stomatin Like 2 |
| CPSF6 | Cleavage And Polyadenylation Specific Factor 6 |
| SLC5A1 | Solute Carrier Family 5 Member 1 |
| HCN4 | Hyperpolarization Activated Cyclic Nucleotide Gated Potassium Channel 4 |
| TPH2 | Tryptophan Hydroxylase 2 |
| TXN2 | Thioredoxin 2 |
| DPAGT1 | Dolichyl-Phosphate N-Acetylglucosaminephosphotransferase 1 |
| CASQ2 | Calsequestrin 2 |
| CYP26A1 | Cytochrome P450 Family 26 Subfamily A Member 1 |
| NEUROD1 | Neuronal Differentiation 1 |
| ALG8 | ALG8 Alpha-1,3-Glucosyltransferase |
| CHD3 | Chromodomain Helicase DNA Binding Protein 3 |
| B3GALNT1 | Beta-1,3-N-Acetylgalactosaminyltransferase 1 (Globoside Blood Group) |
| VPS37A | VPS37A Subunit Of ESCRT-I |
| DPM3 | Dolichyl-Phosphate Mannosyltransferase Subunit 3, Regulatory |
| BACE2 | Beta-Secretase 2 |
| PCDH8 | Protocadherin 8 |
| SDCBP2 | Syndecan Binding Protein 2 |
| ZMYND8 | Zinc Finger MYND-Type Containing 8 |
| SLC30A6 | Solute Carrier Family 30 Member 6 |
| SASS6 | SAS-6 Centriolar Assembly Protein |
| BCL7A | BAF Chromatin Remodeling Complex Subunit BCL7A |
| BCL7C | BAF Chromatin Remodeling Complex Subunit BCL7C |
| TUT1 | Terminal Uridylyl Transferase 1, U6 SnRNA-Specific |
| GPR180 | G Protein-Coupled Receptor 180 |
| FAM53B | Family With Sequence Similarity 53 Member B |
| WDTC1 | WD And Tetratricopeptide Repeats 1 |
| CTAGE1 | Cutaneous T Cell Lymphoma-Associated Antigen 1 |
| MT-ND3 | Mitochondrially Encoded NADH:Ubiquinone Oxidoreductase Core Subunit 3 |
| KIRREL1 | Kirre Like Nephrin Family Adhesion Molecule 1 |
| MT-ND4L | Mitochondrially Encoded NADH:Ubiquinone Oxidoreductase Core Subunit 4L |
| AFG1L | AFG1 Like ATPase |
| TCL6 | T Cell Leukemia/Lymphoma 6 |
| OTULINL | OTU Deubiquitinase With Linear Linkage Specificity Like |
| CFAP47 | Cilia And Flagella Associated Protein 47 |
| RPEL1 | Ribulose-5-Phosphate-3-Epimerase Like 1 |
| MIAT | Myocardial Infarction Associated Transcript |
| MIR373 | MicroRNA 373 |
| MIR154 | MicroRNA 154 |
| MIR582 | MicroRNA 582 |
| MIR103A1 | MicroRNA 103a-1 |
| MIR614 | MicroRNA 614 |
| MIR532 | MicroRNA 532 |
| RNU5A-1 | RNA, U5A Small Nuclear 1 |
| BANCR | BRAF-Activated Non-Protein Coding RNA |
| SLC5A5 | Solute Carrier Family 5 Member 5 |
| ULK2 | Unc-51 Like Autophagy Activating Kinase 2 |
| YKT6 | YKT6 V-SNARE Homolog |
| GIP | Gastric Inhibitory Polypeptide |
| CENPN | Centromere Protein N |
| ALDH16A1 | Aldehyde Dehydrogenase 16 Family Member A1 |
| GCDH | Glutaryl-CoA Dehydrogenase |
| GMFG | Glia Maturation Factor Gamma |
| EXOC2 | Exocyst Complex Component 2 |
| CENPA | Centromere Protein A |
| PTPRB | Protein Tyrosine Phosphatase Receptor Type B |
| ATP8A1 | ATPase Phospholipid Transporting 8A1 |
| ADCY4 | Adenylate Cyclase 4 |
| WWP1 | WW Domain Containing E3 Ubiquitin Protein Ligase 1 |
| CPNE1 | Copine 1 |
| RNF4 | Ring Finger Protein 4 |
| TRIM69 | Tripartite Motif Containing 69 |
| HRNR | Hornerin |
| LEAP2 | Liver Enriched Antimicrobial Peptide 2 |
| BPIFA2 | BPI Fold Containing Family A Member 2 |
| BPIFB6 | BPI Fold Containing Family B Member 6 |
| TWF2 | Twinfilin Actin Binding Protein 2 |
| MAL | Mal, T Cell Differentiation Protein |
| BANF1 | BAF Nuclear Assembly Factor 1 |
| PRSS56 | Serine Protease 56 |
| ERVFRD-1 | Endogenous Retrovirus Group FRD Member 1, Envelope |
| ITGB8 | Integrin Subunit Beta 8 |
| LIMK2 | LIM Domain Kinase 2 |
| TIE1 | Tyrosine Kinase With Immunoglobulin Like And EGF Like Domains 1 |
| PLA2G5 | Phospholipase A2 Group V |
| NAT1 | N-Acetyltransferase 1 |
| SF3B2 | Splicing Factor 3b Subunit 2 |
| RAMP2 | Receptor Activity Modifying Protein 2 |
| SOCS4 | Suppressor Of Cytokine Signaling 4 |
| INSRR | Insulin Receptor Related Receptor |
| CER1 | Cerberus 1, DAN Family BMP Antagonist |
| IFNW1 | Interferon Omega 1 |
| GABARAPL2 | GABA Type A Receptor Associated Protein Like 2 |
| RIPK4 | Receptor Interacting Serine/Threonine Kinase 4 |
| RAD52 | RAD52 Homolog, DNA Repair Protein |
| ABCE1 | ATP Binding Cassette Subfamily E Member 1 |
| DNM1 | Dynamin 1 |
| MLST8 | MTOR Associated Protein, LST8 Homolog |
| TNRC6A | Trinucleotide Repeat Containing Adaptor 6A |
| KPNA5 | Karyopherin Subunit Alpha 5 |
| RHOU | Ras Homolog Family Member U |
| POM121 | POM121 Transmembrane Nucleoporin |
| H3C4 | H3 Clustered Histone 4 |
| H3C3 | H3 Clustered Histone 3 |
| H3C10 | H3 Clustered Histone 10 |
| H3C12 | H3 Clustered Histone 12 |
| H3C11 | H3 Clustered Histone 11 |
| H3C6 | H3 Clustered Histone 6 |
| H3C7 | H3 Clustered Histone 7 |
| H3C8 | H3 Clustered Histone 8 |
| NMNAT1 | Nicotinamide Nucleotide Adenylyltransferase 1 |
| F2RL2 | Coagulation Factor II Thrombin Receptor Like 2 |
| KLF4 | Kruppel Like Factor 4 |
| PDP1 | Pyruvate Dehyrogenase Phosphatase Catalytic Subunit 1 |
| TARS1 | Threonyl-TRNA Synthetase 1 |
| DPP9 | Dipeptidyl Peptidase 9 |
| TNPO1 | Transportin 1 |
| RPS3 | Ribosomal Protein S3 |
| ASPH | Aspartate Beta-Hydroxylase |
| ADCY2 | Adenylate Cyclase 2 |
| CAP1 | Cyclase Associated Actin Cytoskeleton Regulatory Protein 1 |
| HECTD1 | HECT Domain E3 Ubiquitin Protein Ligase 1 |
| TRIM36 | Tripartite Motif Containing 36 |
| LONRF1 | LON Peptidase N-Terminal Domain And Ring Finger 1 |
| FBXO17 | F-Box Protein 17 |
| DEFB136 | Defensin Beta 136 |
| PRPF6 | Pre-MRNA Processing Factor 6 |
| LOX | Lysyl Oxidase |
| VNN2 | Vanin 2 |
| SLFN11 | Schlafen Family Member 11 |
| CERT1 | Ceramide Transporter 1 |
| AASS | Aminoadipate-Semialdehyde Synthase |
| DOCK7 | Dedicator Of Cytokinesis 7 |
| DDX54 | DEAD-Box Helicase 54 |
| EFHD2 | EF-Hand Domain Family Member D2 |
| MARCHF7 | Membrane Associated Ring-CH-Type Finger 7 |
| MC4R | Melanocortin 4 Receptor |
| CA9 | Carbonic Anhydrase 9 |
| EME1 | Essential Meiotic Structure-Specific Endonuclease 1 |
| SCARF1 | Scavenger Receptor Class F Member 1 |
| TUBA8 | Tubulin Alpha 8 |
| HNRNPDL | Heterogeneous Nuclear Ribonucleoprotein D Like |
| PDZD2 | PDZ Domain Containing 2 |
| P2RY6 | Pyrimidinergic Receptor P2Y6 |
| BMP10 | Bone Morphogenetic Protein 10 |
| PYCR1 | Pyrroline-5-Carboxylate Reductase 1 |
| FSHR | Follicle Stimulating Hormone Receptor |
| MIR148A | MicroRNA 148a |
| NLRP8 | NLR Family Pyrin Domain Containing 8 |
| CNTNAP1 | Contactin Associated Protein 1 |
| NKRF | NFKB Repressing Factor |
| OR10J3 | Olfactory Receptor Family 10 Subfamily J Member 3 |
| PLS1 | Plastin 1 |
| NAT10 | N-Acetyltransferase 10 |
| ZMIZ1 | Zinc Finger MIZ-Type Containing 1 |
| ATP1A3 | ATPase Na+/K+ Transporting Subunit Alpha 3 |
| POFUT1 | Protein O-Fucosyltransferase 1 |
| POMT1 | Protein O-Mannosyltransferase 1 |
| PRSS8 | Serine Protease 8 |
| RSPO1 | R-Spondin 1 |
| GLRA2 | Glycine Receptor Alpha 2 |
| CHSY1 | Chondroitin Sulfate Synthase 1 |
| GCSH | Glycine Cleavage System Protein H |
| SLC13A5 | Solute Carrier Family 13 Member 5 |
| MPG | N-Methylpurine DNA Glycosylase |
| SGCE | Sarcoglycan Epsilon |
| CDK5RAP2 | CDK5 Regulatory Subunit Associated Protein 2 |
| MFAP5 | Microfibril Associated Protein 5 |
| SPEG | Striated Muscle Enriched Protein Kinase |
| PARL | Presenilin Associated Rhomboid Like |
| FLCN | Folliculin |
| CAPN10 | Calpain 10 |
| TOP3B | DNA Topoisomerase III Beta |
| SETD1A | SET Domain Containing 1A, Histone Lysine Methyltransferase |
| TNS1 | Tensin 1 |
| SLC39A5 | Solute Carrier Family 39 Member 5 |
| NME5 | NME/NM23 Family Member 5 |
| NRBP1 | Nuclear Receptor Binding Protein 1 |
| SYT7 | Synaptotagmin 7 |
| TRMU | TRNA 5-Methylaminomethyl-2-Thiouridylate Methyltransferase |
| PAPPA2 | Pappalysin 2 |
| SRRT | Serrate, RNA Effector Molecule |
| PLSCR3 | Phospholipid Scramblase 3 |
| TMEM126A | Transmembrane Protein 126A |
| HOXC4 | Homeobox C4 |
| SCYL3 | SCY1 Like Pseudokinase 3 |
| ENC1 | Ectodermal-Neural Cortex 1 |
| KCNH4 | Potassium Voltage-Gated Channel Subfamily H Member 4 |
| MYCL | MYCL Proto-Oncogene, BHLH Transcription Factor |
| OMA1 | OMA1 Zinc Metallopeptidase |
| CNN3 | Calponin 3 |
| SCAPER | S-Phase Cyclin A Associated Protein In The ER |
| LRCH1 | Leucine Rich Repeats And Calponin Homology Domain Containing 1 |
| ANKRD27 | Ankyrin Repeat Domain 27 |
| URI1 | URI1 Prefoldin Like Chaperone |
| TTLL1 | Tubulin Tyrosine Ligase Like 1 |
| ENKUR | Enkurin, TRPC Channel Interacting Protein |
| NHLH1 | Nescient Helix-Loop-Helix 1 |
| FAM163A | Family With Sequence Similarity 163 Member A |
| KIF27 | Kinesin Family Member 27 |
| TXNDC2 | Thioredoxin Domain Containing 2 |
| CFAP53 | Cilia And Flagella Associated Protein 53 |
| DPCD | Deleted In Primary Ciliary Dyskinesia Homolog (Mouse) |
| FRRS1L | Ferric Chelate Reductase 1 Like |
| AGBL1 | ATP/GTP Binding Protein Like 1 |
| AGBL4 | ATP/GTP Binding Protein Like 4 |
| LRRC17 | Leucine Rich Repeat Containing 17 |
| ACER1 | Alkaline Ceramidase 1 |
| TTC30B | Tetratricopeptide Repeat Domain 30B |
| GPR89B | G Protein-Coupled Receptor 89B |
| SMCR8 | SMCR8-C9orf72 Complex Subunit |
| RIPPLY3 | Ripply Transcriptional Repressor 3 |
| ZNF595 | Zinc Finger Protein 595 |
| EIF4E3 | Eukaryotic Translation Initiation Factor 4E Family Member 3 |
| H3C15 | H3 Clustered Histone 15 |
| MIR1248 | MicroRNA 1248 |
| CCAR2 | Cell Cycle And Apoptosis Regulator 2 |
| NAB2 | NGFI-A Binding Protein 2 |
| WARS1 | Tryptophanyl-TRNA Synthetase 1 |
| TIMELESS | Timeless Circadian Regulator |
| DIO2 | Iodothyronine Deiodinase 2 |
| PTN | Pleiotrophin |
| SAGE1 | Sarcoma Antigen 1 |
| CC2D1A | Coiled-Coil And C2 Domain Containing 1A |
| TMEM209 | Transmembrane Protein 209 |
| NTSR1 | Neurotensin Receptor 1 |
| CDA | Cytidine Deaminase |
| ATP6V1B1 | ATPase H+ Transporting V1 Subunit B1 |
| UBE2G1 | Ubiquitin Conjugating Enzyme E2 G1 |
| CDC27 | Cell Division Cycle 27 |
| KLC2 | Kinesin Light Chain 2 |
| SLC44A2 | Solute Carrier Family 44 Member 2 |
| RAB3D | RAB3D, Member RAS Oncogene Family |
| HERC3 | HECT And RLD Domain Containing E3 Ubiquitin Protein Ligase 3 |
| GOLGA7 | Golgin A7 |
| KIF26A | Kinesin Family Member 26A |
| AREL1 | Apoptosis Resistant E3 Ubiquitin Protein Ligase 1 |
| MUCL1 | Mucin Like 1 |
| BTBD6 | BTB Domain Containing 6 |
| TSPAN7 | Tetraspanin 7 |
| SENP3 | SUMO Specific Peptidase 3 |
| GDF9 | Growth Differentiation Factor 9 |
| PPP4C | Protein Phosphatase 4 Catalytic Subunit |
| GPX2 | Glutathione Peroxidase 2 |
| BCLAF1 | BCL2 Associated Transcription Factor 1 |
| PIP4K2B | Phosphatidylinositol-5-Phosphate 4-Kinase Type 2 Beta |
| AGO1 | Argonaute RISC Component 1 |
| NLRP14 | NLR Family Pyrin Domain Containing 14 |
| FGFBP2 | Fibroblast Growth Factor Binding Protein 2 |
| DAPK1 | Death Associated Protein Kinase 1 |
| ABRA | Actin Binding Rho Activating Protein |
| MIR212 | MicroRNA 212 |
| MIR451A | MicroRNA 451a |
| FABP12 | Fatty Acid Binding Protein 12 |
| IDH3B | Isocitrate Dehydrogenase (NAD(+)) 3 Non-Catalytic Subunit Beta |
| SUCLG1 | Succinate-CoA Ligase GDP/ADP-Forming Subunit Alpha |
| MLLT11 | MLLT11 Transcription Factor 7 Cofactor |
| SORT1 | Sortilin 1 |
| RNF128 | Ring Finger Protein 128 |
| TFPI | Tissue Factor Pathway Inhibitor |
| CORO1B | Coronin 1B |
| PWP1 | PWP1 Homolog, Endonuclein |
| MACROH2A1 | MacroH2A.1 Histone |
| NKAP | NFKB Activating Protein |
| ADRA1B | Adrenoceptor Alpha 1B |
| PNPO | Pyridoxamine 5'-Phosphate Oxidase |
| NDUFA1 | NADH:Ubiquinone Oxidoreductase Subunit A1 |
| ETFDH | Electron Transfer Flavoprotein Dehydrogenase |
| SCN4B | Sodium Voltage-Gated Channel Beta Subunit 4 |
| SPG7 | SPG7 Matrix AAA Peptidase Subunit, Paraplegin |
| SLC27A5 | Solute Carrier Family 27 Member 5 |
| SLC35A3 | Solute Carrier Family 35 Member A3 |
| PCDH19 | Protocadherin 19 |
| CRYBA4 | Crystallin Beta A4 |
| DMC1 | DNA Meiotic Recombinase 1 |
| DPP6 | Dipeptidyl Peptidase Like 6 |
| DNMT3L | DNA Methyltransferase 3 Like |
| ZFPM2 | Zinc Finger Protein, FOG Family Member 2 |
| LHX3 | LIM Homeobox 3 |
| TST | Thiosulfate Sulfurtransferase |
| SSTR5 | Somatostatin Receptor 5 |
| COQ9 | Coenzyme Q9 |
| POGZ | Pogo Transposable Element Derived With ZNF Domain |
| TAF13 | TATA-Box Binding Protein Associated Factor 13 |
| CRYBB3 | Crystallin Beta B3 |
| GK2 | Glycerol Kinase 2 |
| ALG11 | ALG11 Alpha-1,2-Mannosyltransferase |
| ALG14 | ALG14 UDP-N-Acetylglucosaminyltransferase Subunit |
| TEF | TEF Transcription Factor, PAR BZIP Family Member |
| TMC1 | Transmembrane Channel Like 1 |
| KLF11 | Kruppel Like Factor 11 |
| KMT2B | Lysine Methyltransferase 2B |
| STRADB | STE20 Related Adaptor Beta |
| COQ4 | Coenzyme Q4 |
| ORC2 | Origin Recognition Complex Subunit 2 |
| TTC3 | Tetratricopeptide Repeat Domain 3 |
| ZFP57 | ZFP57 Zinc Finger Protein |
| ZNF638 | Zinc Finger Protein 638 |
| RCE1 | Ras Converting CAAX Endopeptidase 1 |
| RABIF | RAB Interacting Factor |
| CLVS1 | Clavesin 1 |
| CRYBB1 | Crystallin Beta B1 |
| DCHS1 | Dachsous Cadherin-Related 1 |
| CDH7 | Cadherin 7 |
| ALKBH3 | AlkB Homolog 3, Alpha-Ketoglutaratedependent Dioxygenase |
| WDR11 | WD Repeat Domain 11 |
| CDH19 | Cadherin 19 |
| SOX14 | SRY-Box Transcription Factor 14 |
| IGSF9 | Immunoglobulin Superfamily Member 9 |
| CNTLN | Centlein |
| ELMO3 | Engulfment And Cell Motility 3 |
| RHEBL1 | RHEB Like 1 |
| MEI1 | Meiotic Double-Stranded Break Formation Protein 1 |
| FAM172A | Family With Sequence Similarity 172 Member A |
| ASZ1 | Ankyrin Repeat, SAM And Basic Leucine Zipper Domain Containing 1 |
| CDH20 | Cadherin 20 |
| TMC4 | Transmembrane Channel Like 4 |
| OSGEPL1 | O-Sialoglycoprotein Endopeptidase Like 1 |
| CHCHD4 | Coiled-Coil-Helix-Coiled-Coil-Helix Domain Containing 4 |
| DNAJB14 | DnaJ Heat Shock Protein Family (Hsp40) Member B14 |
| ADAMTS20 | ADAM Metallopeptidase With Thrombospondin Type 1 Motif 20 |
| EPC2 | Enhancer Of Polycomb Homolog 2 |
| ZNF426 | Zinc Finger Protein 426 |
| WWC2 | WW And C2 Domain Containing 2 |
| ZNF2 | Zinc Finger Protein 2 |
| TSSK1B | Testis Specific Serine Kinase 1B |
| ZNF274 | Zinc Finger Protein 274 |
| TMC2 | Transmembrane Channel Like 2 |
| CCDC68 | Coiled-Coil Domain Containing 68 |
| CENPI | Centromere Protein I |
| RNF103 | Ring Finger Protein 103 |
| FAHD2A | Fumarylacetoacetate Hydrolase Domain Containing 2A |
| FAM124B | Family With Sequence Similarity 124 Member B |
| ASPRV1 | Aspartic Peptidase Retroviral Like 1 |
| F8A1 | Coagulation Factor VIII Associated 1 |
| CAPN14 | Calpain 14 |
| TWNK | Twinkle MtDNA Helicase |
| MIEF1 | Mitochondrial Elongation Factor 1 |
| MIEF2 | Mitochondrial Elongation Factor 2 |
| ANKAR | Ankyrin And Armadillo Repeat Containing |
| KRT37 | Keratin 37 |
| YRDC | YrdC N6-Threonylcarbamoyltransferase Domain Containing |
| FAM189A1 | Family With Sequence Similarity 189 Member A1 |
| C3orf67 | Chromosome 3 Open Reading Frame 67 |
| ZNF362 | Zinc Finger Protein 362 |
| TMC3 | Transmembrane Channel Like 3 |
| CENPS | Centromere Protein S |
| ZNF547 | Zinc Finger Protein 547 |
| EVPLL | Envoplakin Like |
| DOP1B | DOP1 Leucine Zipper Like Protein B |
| STUM | Stum, Mechanosensory Transduction Mediator Homolog |
| CTAGE4 | CTAGE Family Member 4 |
| C9orf57 | Chromosome 9 Open Reading Frame 57 |
| HYMAI | Hydatidiform Mole Associated And Imprinted |
| MARCHF11 | Membrane Associated Ring-CH-Type Finger 11 |
| HULC | Hepatocellular Carcinoma Up-Regulated Long Non-Coding RNA |
| MRGPRG-AS1 | MRGPRG Antisense RNA 1 |
| MIR328 | MicroRNA 328 |
| MIR520A | MicroRNA 520a |
| DOCK8-AS1 | DOCK8 Antisense RNA 1 |
| RNU6-1 | RNA, U6 Small Nuclear 1 |
| SNORA1 | Small Nucleolar RNA, H/ACA Box 1 |
| SNORD63 | Small Nucleolar RNA, C/D Box 63 |
| MIR338 | MicroRNA 338 |
| NBEAP1 | Neurobeachin Pseudogene 1 |
| SNORA72 | Small Nucleolar RNA, H/ACA Box 72 |
| MIR3176 | MicroRNA 3176 |
| CTAGE3P | CTAGE Family Member 3, Pseudogene |
| MIR548AA1 | MicroRNA 548aa-1 |
| TSIX | TSIX Transcript, XIST Antisense RNA |
| CACNA1C-AS1 | CACNA1C Antisense RNA 1 |
| ROR1-AS1 | ROR1 Antisense RNA 1 |
| CACNA1C-AS2 | CACNA1C Antisense RNA 2 |
| MT-TG | Mitochondrially Encoded TRNA-Gly (GGN) |
| TP53COR1 | Tumor Protein P53 Pathway Corepressor 1 |
| ENSG00000276861 |  |
| ENSG00000233708 |  |
| GH-LCR | Growth Hormone Locus Control Region |
| ROS1 | ROS Proto-Oncogene 1, Receptor Tyrosine Kinase |
| GAP43 | Growth Associated Protein 43 |
| MFN1 | Mitofusin 1 |
| STK19 | Serine/Threonine Kinase 19 |
| ETV7 | ETS Variant Transcription Factor 7 |
| PDE4C | Phosphodiesterase 4C |
| STXBP1 | Syntaxin Binding Protein 1 |
| P2RY12 | Purinergic Receptor P2Y12 |
| RBMX | RNA Binding Motif Protein X-Linked |
| OGN | Osteoglycin |
| SFN | Stratifin |
| MYH1 | Myosin Heavy Chain 1 |
| MYH4 | Myosin Heavy Chain 4 |
| FUT1 | Fucosyltransferase 1 (H Blood Group) |
| LIME1 | Lck Interacting Transmembrane Adaptor 1 |
| CHST3 | Carbohydrate Sulfotransferase 3 |
| GPR68 | G Protein-Coupled Receptor 68 |
| FAR1 | Fatty Acyl-CoA Reductase 1 |
| ERLIN2 | ER Lipid Raft Associated 2 |
| SH2B2 | SH2B Adaptor Protein 2 |
| ESRRB | Estrogen Related Receptor Beta |
| HMGN2 | High Mobility Group Nucleosomal Binding Domain 2 |
| TRIM15 | Tripartite Motif Containing 15 |
| RTCB | RNA 2',3'-Cyclic Phosphate And 5'-OH Ligase |
| AHCYL1 | Adenosylhomocysteinase Like 1 |
| TNRC6B | Trinucleotide Repeat Containing Adaptor 6B |
| AGO4 | Argonaute RISC Component 4 |
| AGO3 | Argonaute RISC Catalytic Component 3 |
| MGST1 | Microsomal Glutathione S-Transferase 1 |
| ADRB3 | Adrenoceptor Beta 3 |
| BNIP3 | BCL2 Interacting Protein 3 |
| PODXL | Podocalyxin Like |
| SAA2 | Serum Amyloid A2 |
| BMP15 | Bone Morphogenetic Protein 15 |
| YBX3 | Y-Box Binding Protein 3 |
| PER3 | Period Circadian Regulator 3 |
| GAPT | GRB2 Binding Adaptor Protein, Transmembrane |
| ASCC3 | Activating Signal Cointegrator 1 Complex Subunit 3 |
| CHAF1B | Chromatin Assembly Factor 1 Subunit B |
| TNFAIP1 | TNF Alpha Induced Protein 1 |
| KPNA6 | Karyopherin Subunit Alpha 6 |
| PPARD | Peroxisome Proliferator Activated Receptor Delta |
| CYTIP | Cytohesin 1 Interacting Protein |
| RLBP1 | Retinaldehyde Binding Protein 1 |
| MTA2 | Metastasis Associated 1 Family Member 2 |
| GOLPH3 | Golgi Phosphoprotein 3 |
| CH25H | Cholesterol 25-Hydroxylase |
| FOXJ2 | Forkhead Box J2 |
| PAK6 | P21 (RAC1) Activated Kinase 6 |
| NLK | Nemo Like Kinase |
| LCE3A | Late Cornified Envelope 3A |
| FARSA | Phenylalanyl-TRNA Synthetase Subunit Alpha |
| STAU2 | Staufen Double-Stranded RNA Binding Protein 2 |
| LY6G6D | Lymphocyte Antigen 6 Family Member G6D |
| NAP1L1 | Nucleosome Assembly Protein 1 Like 1 |
| CYP2C8 | Cytochrome P450 Family 2 Subfamily C Member 8 |
| NEFM | Neurofilament Medium |
| RPL13 | Ribosomal Protein L13 |
| CALB2 | Calbindin 2 |
| RPL18A | Ribosomal Protein L18a |
| SPAG6 | Sperm Associated Antigen 6 |
| PDF | Peptide Deformylase, Mitochondrial |
| CD163L1 | CD163 Molecule Like 1 |
| SIK2 | Salt Inducible Kinase 2 |
| RPTOR | Regulatory Associated Protein Of MTOR Complex 1 |
| TCF12 | Transcription Factor 12 |
| SLC16A1 | Solute Carrier Family 16 Member 1 |
| HDAC5 | Histone Deacetylase 5 |
| TRPM4 | Transient Receptor Potential Cation Channel Subfamily M Member 4 |
| MAP4K1 | Mitogen-Activated Protein Kinase Kinase Kinase Kinase 1 |
| AP1S2 | Adaptor Related Protein Complex 1 Subunit Sigma 2 |
| CAMKK1 | Calcium/Calmodulin Dependent Protein Kinase Kinase 1 |
| RAPGEF4 | Rap Guanine Nucleotide Exchange Factor 4 |
| EIF4G3 | Eukaryotic Translation Initiation Factor 4 Gamma 3 |
| TRIM17 | Tripartite Motif Containing 17 |
| GBP7 | Guanylate Binding Protein 7 |
| TRIM46 | Tripartite Motif Containing 46 |
| KPNA7 | Karyopherin Subunit Alpha 7 |
| TRIM48 | Tripartite Motif Containing 48 |
| H3C13 | H3 Clustered Histone 13 |
| USP41 | Ubiquitin Specific Peptidase 41 |
| MIR378I | MicroRNA 378i |
| MIR630 | MicroRNA 630 |
| MIR4758 | MicroRNA 4758 |
| MACROH2A2 | MacroH2A.2 Histone |
| BLVRA | Biliverdin Reductase A |
| SLC1A5 | Solute Carrier Family 1 Member 5 |
| EIF2S2 | Eukaryotic Translation Initiation Factor 2 Subunit Beta |
| FCRLA | Fc Receptor Like A |
| ECRG4 | ECRG4 Augurin Precursor |
| GTPBP1 | GTP Binding Protein 1 |
| ADSS2 | Adenylosuccinate Synthase 2 |
| SRGAP1 | SLIT-ROBO Rho GTPase Activating Protein 1 |
| SETDB1 | SET Domain Bifurcated Histone Lysine Methyltransferase 1 |
| ARHGAP4 | Rho GTPase Activating Protein 4 |
| MICAL1 | Microtubule Associated Monooxygenase, Calponin And LIM Domain Containing 1 |
| MS4A6A | Membrane Spanning 4-Domains A6A |
| CDK11B | Cyclin Dependent Kinase 11B |
| TCF20 | Transcription Factor 20 |
| KCTD13 | Potassium Channel Tetramerization Domain Containing 13 |
| GML | Glycosylphosphatidylinositol Anchored Molecule Like |
| CARS1 | Cysteinyl-TRNA Synthetase 1 |
| MIR125B2 | MicroRNA 125b-2 |
| LINC00381 | Long Intergenic Non-Protein Coding RNA 381 |
| THRIL | TNF And HNRNPL Related Immunoregulatory Long Non-Coding RNA |
| A4GALT | Alpha 1,4-Galactosyltransferase (P Blood Group) |
| PER1 | Period Circadian Regulator 1 |
| SRPK2 | SRSF Protein Kinase 2 |
| ATXN3 | Ataxin 3 |
| WDR5 | WD Repeat Domain 5 |
| PAM | Peptidylglycine Alpha-Amidating Monooxygenase |
| DMTN | Dematin Actin Binding Protein |
| PGBD3 | PiggyBac Transposable Element Derived 3 |
| FIRRE | Firre Intergenic Repeating RNA Element |
| KLHL41 | Kelch Like Family Member 41 |
| CAB39 | Calcium Binding Protein 39 |
| ANAPC4 | Anaphase Promoting Complex Subunit 4 |
| ADH5 | Alcohol Dehydrogenase 5 (Class III), Chi Polypeptide |
| S1PR3 | Sphingosine-1-Phosphate Receptor 3 |
| NCK2 | NCK Adaptor Protein 2 |
| ATP6V1G2 | ATPase H+ Transporting V1 Subunit G2 |
| UQCRC2 | Ubiquinol-Cytochrome C Reductase Core Protein 2 |
| PEX14 | Peroxisomal Biogenesis Factor 14 |
| RAB3GAP1 | RAB3 GTPase Activating Protein Catalytic Subunit 1 |
| KLF2 | Kruppel Like Factor 2 |
| AFG3L2 | AFG3 Like Matrix AAA Peptidase Subunit 2 |
| NINJ1 | Ninjurin 1 |
| H1-2 | H1.2 Linker Histone, Cluster Member |
| ATP5PB | ATP Synthase Peripheral Stalk-Membrane Subunit B |
| RTRAF | RNA Transcription, Translation And Transport Factor |
| CLIP1 | CAP-Gly Domain Containing Linker Protein 1 |
| ANKHD1 | Ankyrin Repeat And KH Domain Containing 1 |
| WDHD1 | WD Repeat And HMG-Box DNA Binding Protein 1 |
| HSPH1 | Heat Shock Protein Family H (Hsp110) Member 1 |
| TNFSF12-TNFSF13 | TNFSF12-TNFSF13 Readthrough |
| NF2 | Neurofibromin 2 |
| CYTH1 | Cytohesin 1 |
| MYL6 | Myosin Light Chain 6 |
| QDPR | Quinoid Dihydropteridine Reductase |
| CPE | Carboxypeptidase E |
| DTNA | Dystrobrevin Alpha |
| CDH13 | Cadherin 13 |
| CYTH2 | Cytohesin 2 |
| OPN4 | Opsin 4 |
| PLCH2 | Phospholipase C Eta 2 |
| NECAP1 | NECAP Endocytosis Associated 1 |
| CDCA7L | Cell Division Cycle Associated 7 Like |
| ALDH3B2 | Aldehyde Dehydrogenase 3 Family Member B2 |
| EXOC3 | Exocyst Complex Component 3 |
| CCDC78 | Coiled-Coil Domain Containing 78 |
| CCDC85C | Coiled-Coil Domain Containing 85C |
| SHFL | Shiftless Antiviral Inhibitor Of Ribosomal Frameshifting |
| RASSF5 | Ras Association Domain Family Member 5 |
| CEBPG | CCAAT Enhancer Binding Protein Gamma |
| IFI44 | Interferon Induced Protein 44 |
| SBNO2 | Strawberry Notch Homolog 2 |
| SORBS1 | Sorbin And SH3 Domain Containing 1 |
| LYG2 | Lysozyme G2 |
| LARP1 | La Ribonucleoprotein 1, Translational Regulator |
| USP4 | Ubiquitin Specific Peptidase 4 |
| GADD45B | Growth Arrest And DNA Damage Inducible Beta |
| CCL4L1 | C-C Motif Chemokine Ligand 4 Like 1 |
| DUT | Deoxyuridine Triphosphatase |
| DHX29 | DExH-Box Helicase 29 |
| RBM34 | RNA Binding Motif Protein 34 |
| SSX2 | SSX Family Member 2 |
| GSTZ1 | Glutathione S-Transferase Zeta 1 |
| RBM22 | RNA Binding Motif Protein 22 |
| RPS6KA2 | Ribosomal Protein S6 Kinase A2 |
| SERPINB6 | Serpin Family B Member 6 |
| TRIM9 | Tripartite Motif Containing 9 |
| ANAPC7 | Anaphase Promoting Complex Subunit 7 |
| SPSB1 | SplA/Ryanodine Receptor Domain And SOCS Box Containing 1 |
| UBE3D | Ubiquitin Protein Ligase E3D |
| PEX19 | Peroxisomal Biogenesis Factor 19 |
| IQCB1 | IQ Motif Containing B1 |
| PYCR2 | Pyrroline-5-Carboxylate Reductase 2 |
| ALDH18A1 | Aldehyde Dehydrogenase 18 Family Member A1 |
| SESN1 | Sestrin 1 |
| GABPA | GA Binding Protein Transcription Factor Subunit Alpha |
| MARCHF5 | Membrane Associated Ring-CH-Type Finger 5 |
| GEM | GTP Binding Protein Overexpressed In Skeletal Muscle |
| GALP | Galanin Like Peptide |
| IRAK1BP1 | Interleukin 1 Receptor Associated Kinase 1 Binding Protein 1 |
| MIR136 | MicroRNA 136 |
| TUBB3 | Tubulin Beta 3 Class III |
| UBE2I | Ubiquitin Conjugating Enzyme E2 I |
| SCG5 | Secretogranin V |
| SIT1 | Signaling Threshold Regulating Transmembrane Adaptor 1 |
| DGKA | Diacylglycerol Kinase Alpha |
| VDAC2 | Voltage Dependent Anion Channel 2 |
| HOXA5 | Homeobox A5 |
| IGF2BP1 | Insulin Like Growth Factor 2 MRNA Binding Protein 1 |
| SWAP70 | Switching B Cell Complex Subunit SWAP70 |
| CD2-LCR | CD2 Locus Control Region |
| VAPB | VAMP Associated Protein B And C |
| PDCD6IP | Programmed Cell Death 6 Interacting Protein |
| LCN1 | Lipocalin 1 |
| EYA1 | EYA Transcriptional Coactivator And Phosphatase 1 |
| RPS6KB2 | Ribosomal Protein S6 Kinase B2 |
| PRR5 | Proline Rich 5 |
| SEC61A2 | SEC61 Translocon Subunit Alpha 2 |
| SEC61G | SEC61 Translocon Subunit Gamma |
| LOC102725035 | Leukocyte Immunoglobulin-Like Receptor Subfamily B Member 3 |
| LOC107987462 | Leukocyte Immunoglobulin-Like Receptor Subfamily B Member 3 |
| LOC107987425 | Leukocyte Immunoglobulin-Like Receptor Subfamily B Member 3 |
| KLK1 | Kallikrein 1 |
| ADAM19 | ADAM Metallopeptidase Domain 19 |
| EIF6 | Eukaryotic Translation Initiation Factor 6 |
| ANXA7 | Annexin A7 |
| CELF2 | CUGBP Elav-Like Family Member 2 |
| DNAJC19 | DnaJ Heat Shock Protein Family (Hsp40) Member C19 |
| CDK2AP1 | Cyclin Dependent Kinase 2 Associated Protein 1 |
| ATXN7 | Ataxin 7 |
| FNBP1 | Formin Binding Protein 1 |
| TRMT5 | TRNA Methyltransferase 5 |
| SLC7A4 | Solute Carrier Family 7 Member 4 |
| DRG2 | Developmentally Regulated GTP Binding Protein 2 |
| DHRS9 | Dehydrogenase/Reductase 9 |
| ATPAF2 | ATP Synthase Mitochondrial F1 Complex Assembly Factor 2 |
| SSR3 | Signal Sequence Receptor Subunit 3 |
| MYO15A | Myosin XVA |
| ADGRA2 | Adhesion G Protein-Coupled Receptor A2 |
| C12orf65 | Chromosome 12 Open Reading Frame 65 |
| ZFYVE21 | Zinc Finger FYVE-Type Containing 21 |
| SPIDR | Scaffold Protein Involved In DNA Repair |
| PHF13 | PHD Finger Protein 13 |
| MOK | MOK Protein Kinase |
| GPATCH3 | G-Patch Domain Containing 3 |
| GPR42 | G Protein-Coupled Receptor 42 (Gene/Pseudogene) |
| TAGLN2 | Transgelin 2 |
| ZBTB7B | Zinc Finger And BTB Domain Containing 7B |
| ADCY6 | Adenylate Cyclase 6 |
| SIAH1 | Siah E3 Ubiquitin Protein Ligase 1 |
| SIAH2 | Siah E3 Ubiquitin Protein Ligase 2 |
| KIF5A | Kinesin Family Member 5A |
| RCHY1 | Ring Finger And CHY Zinc Finger Domain Containing 1 |
| PDE1A | Phosphodiesterase 1A |
| AP1M2 | Adaptor Related Protein Complex 1 Subunit Mu 2 |
| RBBP6 | RB Binding Protein 6, Ubiquitin Ligase |
| ASB13 | Ankyrin Repeat And SOCS Box Containing 13 |
| HEBP2 | Heme Binding Protein 2 |
| FBXO22 | F-Box Protein 22 |
| ERV3-1 | Endogenous Retrovirus Group 3 Member 1, Envelope |
| SP3 | Sp3 Transcription Factor |
| OXT | Oxytocin/Neurophysin I Prepropeptide |
| MIR125B1 | MicroRNA 125b-1 |
| GNAZ | G Protein Subunit Alpha Z |
| PIAS2 | Protein Inhibitor Of Activated STAT 2 |
| PFN2 | Profilin 2 |
| NR4A3 | Nuclear Receptor Subfamily 4 Group A Member 3 |
| PDCD4 | Programmed Cell Death 4 |
| EIF2B5 | Eukaryotic Translation Initiation Factor 2B Subunit Epsilon |
| AMFR | Autocrine Motility Factor Receptor |
| SMARCC1 | SWI/SNF Related, Matrix Associated, Actin Dependent Regulator Of Chromatin Subfamily C Member 1 |
| CERK | Ceramide Kinase |
| CPVL | Carboxypeptidase Vitellogenic Like |
| DSTN | Destrin, Actin Depolymerizing Factor |
| TRAPPC9 | Trafficking Protein Particle Complex 9 |
| SLC25A6 | Solute Carrier Family 25 Member 6 |
| CLEC14A | C-Type Lectin Domain Containing 14A |
| REV1 | REV1 DNA Directed Polymerase |
| SETDB2 | SET Domain Bifurcated Histone Lysine Methyltransferase 2 |
| SLC52A2 | Solute Carrier Family 52 Member 2 |
| TUBA1C | Tubulin Alpha 1c |
| CASP12 | Caspase 12 (Gene/Pseudogene) |
| TOMM70 | Translocase Of Outer Mitochondrial Membrane 70 |
| MAP1LC3B | Microtubule Associated Protein 1 Light Chain 3 Beta |
| KRR1 | KRR1 Small Subunit Processome Component Homolog |
| FXR1 | FMR1 Autosomal Homolog 1 |
| USP5 | Ubiquitin Specific Peptidase 5 |
| SULF1 | Sulfatase 1 |
| EBF1 | EBF Transcription Factor 1 |
| CNMD | Chondromodulin |
| ADCY5 | Adenylate Cyclase 5 |
| CTSF | Cathepsin F |
| PYGB | Glycogen Phosphorylase B |
| PDE1B | Phosphodiesterase 1B |
| POLR2F | RNA Polymerase II Subunit F |
| RAB5B | RAB5B, Member RAS Oncogene Family |
| CDC16 | Cell Division Cycle 16 |
| CCNF | Cyclin F |
| ANAPC11 | Anaphase Promoting Complex Subunit 11 |
| KIF4A | Kinesin Family Member 4A |
| RAB4B | RAB4B, Member RAS Oncogene Family |
| KLHL20 | Kelch Like Family Member 20 |
| MGRN1 | Mahogunin Ring Finger 1 |
| MYO10 | Myosin X |
| CDC23 | Cell Division Cycle 23 |
| ANAPC5 | Anaphase Promoting Complex Subunit 5 |
| TXNDC5 | Thioredoxin Domain Containing 5 |
| CREG1 | Cellular Repressor Of E1A Stimulated Genes 1 |
| RBSN | Rabenosyn, RAB Effector |
| KLHL9 | Kelch Like Family Member 9 |
| MUC20 | Mucin 20, Cell Surface Associated |
| FBXO2 | F-Box Protein 2 |
| A1BG | Alpha-1-B Glycoprotein |
| RNF25 | Ring Finger Protein 25 |
| POLR2K | RNA Polymerase II Subunit K |
| HERC4 | HECT And RLD Domain Containing E3 Ubiquitin Protein Ligase 4 |
| RNF144B | Ring Finger Protein 144B |
| RNF111 | Ring Finger Protein 111 |
| BTBD1 | BTB Domain Containing 1 |
| KLHL22 | Kelch Like Family Member 22 |
| ZNRF2 | Zinc And Ring Finger 2 |
| MUC17 | Mucin 17, Cell Surface Associated |
| FBXL18 | F-Box And Leucine Rich Repeat Protein 18 |
| ASB3 | Ankyrin Repeat And SOCS Box Containing 3 |
| ASB6 | Ankyrin Repeat And SOCS Box Containing 6 |
| ASB16 | Ankyrin Repeat And SOCS Box Containing 16 |
| KLHL25 | Kelch Like Family Member 25 |
| UNKL | Unk Like Zinc Finger |
| LRR1 | Leucine Rich Repeat Protein 1 |
| FBXO21 | F-Box Protein 21 |
| ASB4 | Ankyrin Repeat And SOCS Box Containing 4 |
| KLHL11 | Kelch Like Family Member 11 |
| FBXL14 | F-Box And Leucine Rich Repeat Protein 14 |
| C1orf35 | Chromosome 1 Open Reading Frame 35 |
| FBXO30 | F-Box Protein 30 |
| RNF217 | Ring Finger Protein 217 |
| MUC21 | Mucin 21, Cell Surface Associated |
| ASB17 | Ankyrin Repeat And SOCS Box Containing 17 |
| NOTCH4 | Notch Receptor 4 |
| PIKFYVE | Phosphoinositide Kinase, FYVE-Type Zinc Finger Containing |
| PTPN12 | Protein Tyrosine Phosphatase Non-Receptor Type 12 |
| GABRG2 | Gamma-Aminobutyric Acid Type A Receptor Subunit Gamma2 |
| MUTYH | MutY DNA Glycosylase |
| TDP1 | Tyrosyl-DNA Phosphodiesterase 1 |
| POMT2 | Protein O-Mannosyltransferase 2 |
| ITM2B | Integral Membrane Protein 2B |
| KISS1 | KiSS-1 Metastasis Suppressor |
| NKX3-1 | NK3 Homeobox 1 |
| ATP1A4 | ATPase Na+/K+ Transporting Subunit Alpha 4 |
| PLAG1 | PLAG1 Zinc Finger |
| PCSK6 | Proprotein Convertase Subtilisin/Kexin Type 6 |
| CSAD | Cysteine Sulfinic Acid Decarboxylase |
| MRPL44 | Mitochondrial Ribosomal Protein L44 |
| THAP1 | THAP Domain Containing 1 |
| STMN2 | Stathmin 2 |
| MAFA | MAF BZIP Transcription Factor A |
| AP3M2 | Adaptor Related Protein Complex 3 Subunit Mu 2 |
| TCFL5 | Transcription Factor Like 5 |
| ABCF3 | ATP Binding Cassette Subfamily F Member 3 |
| ANO7 | Anoctamin 7 |
| TPPP2 | Tubulin Polymerization Promoting Protein Family Member 2 |
| TTC7B | Tetratricopeptide Repeat Domain 7B |
| LETM2 | Leucine Zipper And EF-Hand Containing Transmembrane Protein 2 |
| POU5F1B | POU Class 5 Homeobox 1B |
| CFAP44 | Cilia And Flagella Associated Protein 44 |
| REXO1L1P | REXO1 Like 1, Pseudogene |
| NAPEPLD | N-Acyl Phosphatidylethanolamine Phospholipase D |
| PTPRH | Protein Tyrosine Phosphatase Receptor Type H |
| DOK4 | Docking Protein 4 |
| TMEM131 | Transmembrane Protein 131 |
| GIMAP3P | GTPase, IMAP Family Member 3 Pseudogene |
| AAVS1 | Adeno-Associated Virus Integration Site 1 |
| CLIC1 | Chloride Intracellular Channel 1 |
| CNOT1 | CCR4-NOT Transcription Complex Subunit 1 |
| BCAS3 | BCAS3 Microtubule Associated Cell Migration Factor |
| CCT4 | Chaperonin Containing TCP1 Subunit 4 |
| MYL1 | Myosin Light Chain 1 |
| CLCA2 | Chloride Channel Accessory 2 |
| SLC2A11 | Solute Carrier Family 2 Member 11 |
| ZNF76 | Zinc Finger Protein 76 |
| DDX27 | DEAD-Box Helicase 27 |
| SCAF4 | SR-Related CTD Associated Factor 4 |
| ATG9B | Autophagy Related 9B |
| TMCO6 | Transmembrane And Coiled-Coil Domains 6 |
| RPS4XP13 | Ribosomal Protein S4X Pseudogene 13 |
| NAB1 | NGFI-A Binding Protein 1 |
| PON3 | Paraoxonase 3 |
| USP21 | Ubiquitin Specific Peptidase 21 |
| ARHGEF5 | Rho Guanine Nucleotide Exchange Factor 5 |
| PDZD8 | PDZ Domain Containing 8 |
| FGF14 | Fibroblast Growth Factor 14 |
| GOT1 | Glutamic-Oxaloacetic Transaminase 1 |
| HIP1 | Huntingtin Interacting Protein 1 |
| GOLGA2 | Golgin A2 |
| MEOX2 | Mesenchyme Homeobox 2 |
| ZC3H14 | Zinc Finger CCCH-Type Containing 14 |
| COMMD8 | COMM Domain Containing 8 |
| PBXIP1 | PBX Homeobox Interacting Protein 1 |
| COMMD10 | COMM Domain Containing 10 |
| ALDH1A1 | Aldehyde Dehydrogenase 1 Family Member A1 |
| RANGAP1 | Ran GTPase Activating Protein 1 |
| BASP1 | Brain Abundant Membrane Attached Signal Protein 1 |
| CTNND2 | Catenin Delta 2 |
| TSPYL1 | TSPY Like 1 |
| POP1 | POP1 Homolog, Ribonuclease P/MRP Subunit |
| FXR2 | FMR1 Autosomal Homolog 2 |
| GTPBP4 | GTP Binding Protein 4 |
| EBNA1BP2 | EBNA1 Binding Protein 2 |
| RRS1 | Ribosome Biogenesis Regulator 1 Homolog |
| PLXNB1 | Plexin B1 |
| BMP8A | Bone Morphogenetic Protein 8a |
| HNRNPUL1 | Heterogeneous Nuclear Ribonucleoprotein U Like 1 |
| PLA2G4B | Phospholipase A2 Group IVB |
| WNT3A | Wnt Family Member 3A |
| LITAF | Lipopolysaccharide Induced TNF Factor |
| PLIN2 | Perilipin 2 |
| AARS2 | Alanyl-TRNA Synthetase 2, Mitochondrial |
| FBLN1 | Fibulin 1 |
| SMG1 | SMG1 Nonsense Mediated MRNA Decay Associated PI3K Related Kinase |
| GPX8 | Glutathione Peroxidase 8 (Putative) |
| NCAPD2 | Non-SMC Condensin I Complex Subunit D2 |
| EEF1E1 | Eukaryotic Translation Elongation Factor 1 Epsilon 1 |
| DDX23 | DEAD-Box Helicase 23 |
| HOXB6 | Homeobox B6 |
| DERL2 | Derlin 2 |
| ESYT2 | Extended Synaptotagmin 2 |
| SMC6 | Structural Maintenance Of Chromosomes 6 |
| RWDD1 | RWD Domain Containing 1 |
| TMEM109 | Transmembrane Protein 109 |
| DUSP14 | Dual Specificity Phosphatase 14 |
| PDE1C | Phosphodiesterase 1C |
| PRKCSH | Protein Kinase C Substrate 80K-H |
| DNAJC5 | DnaJ Heat Shock Protein Family (Hsp40) Member C5 |
| FRK | Fyn Related Src Family Tyrosine Kinase |
| KIF22 | Kinesin Family Member 22 |
| ATP6V0A4 | ATPase H+ Transporting V0 Subunit A4 |
| PGAM1 | Phosphoglycerate Mutase 1 |
| PPIE | Peptidylprolyl Isomerase E |
| ATP6V0A1 | ATPase H+ Transporting V0 Subunit A1 |
| UBE2S | Ubiquitin Conjugating Enzyme E2 S |
| TRPC1 | Transient Receptor Potential Cation Channel Subfamily C Member 1 |
| UBE2Z | Ubiquitin Conjugating Enzyme E2 Z |
| RAB31 | RAB31, Member RAS Oncogene Family |
| FUCA2 | Alpha-L-Fucosidase 2 |
| ATOX1 | Antioxidant 1 Copper Chaperone |
| ANAPC1 | Anaphase Promoting Complex Subunit 1 |
| APEH | Acylaminoacyl-Peptide Hydrolase |
| RAB37 | RAB37, Member RAS Oncogene Family |
| NAPRT | Nicotinate Phosphoribosyltransferase |
| RNF19A | Ring Finger Protein 19A, RBR E3 Ubiquitin Protein Ligase |
| ABCA13 | ATP Binding Cassette Subfamily A Member 13 |
| FBXL3 | F-Box And Leucine Rich Repeat Protein 3 |
| UBA6 | Ubiquitin Like Modifier Activating Enzyme 6 |
| SERPINB10 | Serpin Family B Member 10 |
| KCMF1 | Potassium Channel Modulatory Factor 1 |
| RNF130 | Ring Finger Protein 130 |
| KIF3C | Kinesin Family Member 3C |
| RNF220 | Ring Finger Protein 220 |
| KLC4 | Kinesin Light Chain 4 |
| WIPF2 | WAS/WASL Interacting Protein Family Member 2 |
| ENPP4 | Ectonucleotide Pyrophosphatase/Phosphodiesterase 4 |
| RILP | Rab Interacting Lysosomal Protein |
| CPPED1 | Calcineurin Like Phosphoesterase Domain Containing 1 |
| FBXL13 | F-Box And Leucine Rich Repeat Protein 13 |
| FBXO15 | F-Box Protein 15 |
| FBXW2 | F-Box And WD Repeat Domain Containing 2 |
| FBXO31 | F-Box Protein 31 |
| ASB8 | Ankyrin Repeat And SOCS Box Containing 8 |
| TMBIM1 | Transmembrane BAX Inhibitor Motif Containing 1 |
| MOSPD2 | Motile Sperm Domain Containing 2 |
| HERC6 | HECT And RLD Domain Containing E3 Ubiquitin Protein Ligase Family Member 6 |
| DYNC1LI2 | Dynein Cytoplasmic 1 Light Intermediate Chain 2 |
| FBXO44 | F-Box Protein 44 |
| FBXW8 | F-Box And WD Repeat Domain Containing 8 |
| ARL8A | ADP Ribosylation Factor Like GTPase 8A |
| ASB9 | Ankyrin Repeat And SOCS Box Containing 9 |
| KIF2B | Kinesin Family Member 2B |
| TSPAN14 | Tetraspanin 14 |
| SIKE1 | Suppressor Of IKBKE 1 |
| CDC26 | Cell Division Cycle 26 |
| FBXL12 | F-Box And Leucine Rich Repeat Protein 12 |
| FBXL16 | F-Box And Leucine Rich Repeat Protein 16 |
| FBXO40 | F-Box Protein 40 |
| LRRC41 | Leucine Rich Repeat Containing 41 |
| FBXL15 | F-Box And Leucine Rich Repeat Protein 15 |
| FBXL8 | F-Box And Leucine Rich Repeat Protein 8 |
| FBXL22 | F-Box And Leucine Rich Repeat Protein 22 |
| FBXW10 | F-Box And WD Repeat Domain Containing 10 |
| FBXW12 | F-Box And WD Repeat Domain Containing 12 |
| GHDC | GH3 Domain Containing |
| FBXO41 | F-Box Protein 41 |
| FBXO27 | F-Box Protein 27 |
| ASB12 | Ankyrin Repeat And SOCS Box Containing 12 |
| KBTBD13 | Kelch Repeat And BTB Domain Containing 13 |
| C6orf120 | Chromosome 6 Open Reading Frame 120 |
| RAB44 | RAB44, Member RAS Oncogene Family |
| TRIM39-RPP21 | TRIM39-RPP21 Readthrough |
| MIR5196 | MicroRNA 5196 |
| NOC3L | NOC3 Like DNA Replication Regulator |
| WDR18 | WD Repeat Domain 18 |
| DFFA | DNA Fragmentation Factor Subunit Alpha |
| NOP2 | NOP2 Nucleolar Protein |
| SMAD1 | SMAD Family Member 1 |
| TNKS | Tankyrase |
| DPP10 | Dipeptidyl Peptidase Like 10 |
| H2AJ | H2A.J Histone |
| HBE1 | Hemoglobin Subunit Epsilon 1 |
| HES4 | Hes Family BHLH Transcription Factor 4 |
| MYH13 | Myosin Heavy Chain 13 |
| ESAM | Endothelial Cell Adhesion Molecule |
| SRSF5 | Serine And Arginine Rich Splicing Factor 5 |
| RSL1D1 | Ribosomal L1 Domain Containing 1 |
| MBD2 | Methyl-CpG Binding Domain Protein 2 |
| PELP1 | Proline, Glutamate And Leucine Rich Protein 1 |
| GSPT1 | G1 To S Phase Transition 1 |
| RBM28 | RNA Binding Motif Protein 28 |
| ABO | ABO, Alpha 1-3-N-Acetylgalactosaminyltransferase And Alpha 1-3-Galactosyltransferase |
| NOP16 | NOP16 Nucleolar Protein |
| POLB | DNA Polymerase Beta |
| EGLN3 | Egl-9 Family Hypoxia Inducible Factor 3 |
| PTRH2 | Peptidyl-TRNA Hydrolase 2 |
| MSI2 | Musashi RNA Binding Protein 2 |
| MTF1 | Metal Regulatory Transcription Factor 1 |
| LIPT1 | Lipoyltransferase 1 |
| GPR132 | G Protein-Coupled Receptor 132 |
| PIM3 | Pim-3 Proto-Oncogene, Serine/Threonine Kinase |
| AKTIP | AKT Interacting Protein |
| PGRMC2 | Progesterone Receptor Membrane Component 2 |
| NUBPL | Nucleotide Binding Protein Like |
| SSBP2 | Single Stranded DNA Binding Protein 2 |
| TRAF3IP3 | TRAF3 Interacting Protein 3 |
| MAFK | MAF BZIP Transcription Factor K |
| CXCL17 | C-X-C Motif Chemokine Ligand 17 |
| EPB41L1 | Erythrocyte Membrane Protein Band 4.1 Like 1 |
| CSNK1E | Casein Kinase 1 Epsilon |
| FPR3 | Formyl Peptide Receptor 3 |
| USP10 | Ubiquitin Specific Peptidase 10 |
| DEFB131B | Defensin Beta 131B |
| TRPV6 | Transient Receptor Potential Cation Channel Subfamily V Member 6 |
| SRD5A1 | Steroid 5 Alpha-Reductase 1 |
| GOLGB1 | Golgin B1 |
| PABPC4 | Poly(A) Binding Protein Cytoplasmic 4 |
| NAP1L4 | Nucleosome Assembly Protein 1 Like 4 |
| CCT6A | Chaperonin Containing TCP1 Subunit 6A |
| DHX57 | DExH-Box Helicase 57 |
| NSA2 | NSA2 Ribosome Biogenesis Factor |
| MRTO4 | MRT4 Homolog, Ribosome Maturation Factor |
| UMODL1 | Uromodulin Like 1 |
| LRP6 | LDL Receptor Related Protein 6 |
| EPS15 | Epidermal Growth Factor Receptor Pathway Substrate 15 |
| LAMC3 | Laminin Subunit Gamma 3 |
| RHOC | Ras Homolog Family Member C |
| POLQ | DNA Polymerase Theta |
| SSR2 | Signal Sequence Receptor Subunit 2 |
| MLLT3 | MLLT3 Super Elongation Complex Subunit |
| RGS3 | Regulator Of G Protein Signaling 3 |
| BOK | BCL2 Family Apoptosis Regulator BOK |
| GTPBP2 | GTP Binding Protein 2 |
| GCNT3 | Glucosaminyl (N-Acetyl) Transferase 3, Mucin Type |
| ZC3H12C | Zinc Finger CCCH-Type Containing 12C |
| SLC46A2 | Solute Carrier Family 46 Member 2 |
| TRIM40 | Tripartite Motif Containing 40 |
| USP19 | Ubiquitin Specific Peptidase 19 |
| SMC4 | Structural Maintenance Of Chromosomes 4 |
| LRRFIP2 | LRR Binding FLII Interacting Protein 2 |
| TRIM65 | Tripartite Motif Containing 65 |
| FCRL2 | Fc Receptor Like 2 |
| HSPA14 | Heat Shock Protein Family A (Hsp70) Member 14 |
| BCAR1 | BCAR1 Scaffold Protein, Cas Family Member |
| GTF2B | General Transcription Factor IIB |
| MIR30C1 | MicroRNA 30c-1 |
| MT1A | Metallothionein 1A |
| SSX3 | SSX Family Member 3 |
| LOC109286563 | TBX21 Promoter Region |
| SEMA5A | Semaphorin 5A |
| ARID5A | AT-Rich Interaction Domain 5A |
| PRR7 | Proline Rich 7, Synaptic |
| MRPS26 | Mitochondrial Ribosomal Protein S26 |
| MAP4K2 | Mitogen-Activated Protein Kinase Kinase Kinase Kinase 2 |
| TMEM37 | Transmembrane Protein 37 |
| HAT1 | Histone Acetyltransferase 1 |
| UBR5 | Ubiquitin Protein Ligase E3 Component N-Recognin 5 |
| S1PR2 | Sphingosine-1-Phosphate Receptor 2 |
| ADRA2A | Adrenoceptor Alpha 2A |
| ADH7 | Alcohol Dehydrogenase 7 (Class IV), Mu Or Sigma Polypeptide |
| RNF13 | Ring Finger Protein 13 |
| ORC4 | Origin Recognition Complex Subunit 4 |
| FKBP1B | FKBP Prolyl Isomerase 1B |
| FBXL2 | F-Box And Leucine Rich Repeat Protein 2 |
| GPHA2 | Glycoprotein Hormone Subunit Alpha 2 |
| ARSH | Arylsulfatase Family Member H |
| MIR370 | MicroRNA 370 |
| ARL1 | ADP Ribosylation Factor Like GTPase 1 |
| ARNTL2 | Aryl Hydrocarbon Receptor Nuclear Translocator Like 2 |
| ARC | Activity Regulated Cytoskeleton Associated Protein |
| GRAMD4 | GRAM Domain Containing 4 |
| MCM3 | Minichromosome Maintenance Complex Component 3 |
| MFGE8 | Milk Fat Globule-EGF Factor 8 Protein |
| STK3 | Serine/Threonine Kinase 3 |
| RGS14 | Regulator Of G Protein Signaling 14 |
| DDAH1 | Dimethylarginine Dimethylaminohydrolase 1 |
| MORF4L1 | Mortality Factor 4 Like 1 |
| MYO1D | Myosin ID |
| ATG9A | Autophagy Related 9A |
| APBA3 | Amyloid Beta Precursor Protein Binding Family A Member 3 |
| NCOA5 | Nuclear Receptor Coactivator 5 |
| MPP6 | Membrane Palmitoylated Protein 6 |
| FNDC4 | Fibronectin Type III Domain Containing 4 |
| RGCC | Regulator Of Cell Cycle |
| AKR1B1 | Aldo-Keto Reductase Family 1 Member B |
| NTN1 | Netrin 1 |
| FSHB | Follicle Stimulating Hormone Subunit Beta |
| DDAH2 | Dimethylarginine Dimethylaminohydrolase 2 |
| TRPM8 | Transient Receptor Potential Cation Channel Subfamily M Member 8 |
| LPXN | Leupaxin |
| MIR196A2 | MicroRNA 196a-2 |
| MIR503 | MicroRNA 503 |
| HELZ2 | Helicase With Zinc Finger 2 |
| TUBG1 | Tubulin Gamma 1 |
| TUBB2B | Tubulin Beta 2B Class IIb |
| SEC63 | SEC63 Homolog, Protein Translocation Regulator |
| KRIT1 | KRIT1 Ankyrin Repeat Containing |
| GLIPR2 | GLI Pathogenesis Related 2 |
| HNRNPA0 | Heterogeneous Nuclear Ribonucleoprotein A0 |
| SIGMAR1 | Sigma Non-Opioid Intracellular Receptor 1 |
| SSX1 | SSX Family Member 1 |
| RPS2 | Ribosomal Protein S2 |
| MGAT3 | Beta-1,4-Mannosyl-Glycoprotein 4-Beta-N-Acetylglucosaminyltransferase |
| IRF2BPL | Interferon Regulatory Factor 2 Binding Protein Like |
| FCRL1 | Fc Receptor Like 1 |
| TIAL1 | TIA1 Cytotoxic Granule Associated RNA Binding Protein Like 1 |
| TUBA1A | Tubulin Alpha 1a |
| CSNK2A2 | Casein Kinase 2 Alpha 2 |
| DUSP22 | Dual Specificity Phosphatase 22 |
| SLC6A2 | Solute Carrier Family 6 Member 2 |
| HSD11B2 | Hydroxysteroid 11-Beta Dehydrogenase 2 |
| PITX1 | Paired Like Homeodomain 1 |
| HPN | Hepsin |
| GCKR | Glucokinase Regulator |
| ALDH9A1 | Aldehyde Dehydrogenase 9 Family Member A1 |
| TAOK3 | TAO Kinase 3 |
| RDH10 | Retinol Dehydrogenase 10 |
| MBNL1 | Muscleblind Like Splicing Regulator 1 |
| SLC45A3 | Solute Carrier Family 45 Member 3 |
| SNX18 | Sorting Nexin 18 |
| GPS2 | G Protein Pathway Suppressor 2 |
| NELFE | Negative Elongation Factor Complex Member E |
| SCGB1D2 | Secretoglobin Family 1D Member 2 |
| MIR139 | MicroRNA 139 |
| MIR217 | MicroRNA 217 |
| OGFR | Opioid Growth Factor Receptor |
| MYL7 | Myosin Light Chain 7 |
| MYL5 | Myosin Light Chain 5 |
| PPM1D | Protein Phosphatase, Mg2+/Mn2+ Dependent 1D |
| HSD17B4 | Hydroxysteroid 17-Beta Dehydrogenase 4 |
| HRH3 | Histamine Receptor H3 |
| PCTP | Phosphatidylcholine Transfer Protein |
| RGS5 | Regulator Of G Protein Signaling 5 |
| DUOXA2 | Dual Oxidase Maturation Factor 2 |
| UEVLD | UEV And Lactate/Malate Dehyrogenase Domains |
| ZBTB46 | Zinc Finger And BTB Domain Containing 46 |
| TCAIM | T Cell Activation Inhibitor, Mitochondrial |
| COLCA2 | Colorectal Cancer Associated 2 |
| CPAMD8 | C3 And PZP Like Alpha-2-Macroglobulin Domain Containing 8 |
| LY6G6F | Lymphocyte Antigen 6 Family Member G6F |
| GPANK1 | G-Patch Domain And Ankyrin Repeats 1 |
| GYG1 | Glycogenin 1 |
| HK3 | Hexokinase 3 |
| RAB3A | RAB3A, Member RAS Oncogene Family |
| PAFAH1B2 | Platelet Activating Factor Acetylhydrolase 1b Catalytic Subunit 2 |
| SEC24D | SEC24 Homolog D, COPII Coat Complex Component |
| ATP6V1E1 | ATPase H+ Transporting V1 Subunit E1 |
| UBA5 | Ubiquitin Like Modifier Activating Enzyme 5 |
| UBE2R2 | Ubiquitin Conjugating Enzyme E2 R2 |
| HECW2 | HECT, C2 And WW Domain Containing E3 Ubiquitin Protein Ligase 2 |
| SEC31A | SEC31 Homolog A, COPII Coat Complex Component |
| ARHGAP9 | Rho GTPase Activating Protein 9 |
| KLHL3 | Kelch Like Family Member 3 |
| PGM2 | Phosphoglucomutase 2 |
| SYNGR1 | Synaptogyrin 1 |
| ATP6V1C2 | ATPase H+ Transporting V1 Subunit C2 |
| ANAPC2 | Anaphase Promoting Complex Subunit 2 |
| UBE2D4 | Ubiquitin Conjugating Enzyme E2 D4 (Putative) |
| UBE2Q2 | Ubiquitin Conjugating Enzyme E2 Q2 |
| RNF138 | Ring Finger Protein 138 |
| OSTF1 | Osteoclast Stimulating Factor 1 |
| DEGS1 | Delta 4-Desaturase, Sphingolipid 1 |
| ATP6V1D | ATPase H+ Transporting V1 Subunit D |
| ATP6V1F | ATPase H+ Transporting V1 Subunit F |
| ATP6V1G3 | ATPase H+ Transporting V1 Subunit G3 |
| ALDOC | Aldolase, Fructose-Bisphosphate C |
| ATP6V0C | ATPase H+ Transporting V0 Subunit C |
| ANAPC10 | Anaphase Promoting Complex Subunit 10 |
| ERP44 | Endoplasmic Reticulum Protein 44 |
| RAP2B | RAP2B, Member Of RAS Oncogene Family |
| KLC3 | Kinesin Light Chain 3 |
| KIF20A | Kinesin Family Member 20A |
| NDUFC2 | NADH:Ubiquinone Oxidoreductase Subunit C2 |
| DERA | Deoxyribose-Phosphate Aldolase |
| SCAMP1 | Secretory Carrier Membrane Protein 1 |
| NIT2 | Nitrilase Family Member 2 |
| GAN | Gigaxonin |
| ATP6V1C1 | ATPase H+ Transporting V1 Subunit C1 |
| ATP11B | ATPase Phospholipid Transporting 11B (Putative) |
| LRG1 | Leucine Rich Alpha-2-Glycoprotein 1 |
| SRMS | Src-Related Kinase Lacking C-Terminal Regulatory Tyrosine And N-Terminal Myristylation Sites |
| TMEM63A | Transmembrane Protein 63A |
| KLHL13 | Kelch Like Family Member 13 |
| FOLR3 | Folate Receptor Gamma |
| SEC24A | SEC24 Homolog A, COPII Coat Complex Component |
| ATP6V1E2 | ATPase H+ Transporting V1 Subunit E2 |
| ATP6V1G1 | ATPase H+ Transporting V1 Subunit G1 |
| BRK1 | BRICK1 Subunit Of SCAR/WAVE Actin Nucleating Complex |
| LRRC7 | Leucine Rich Repeat Containing 7 |
| VAT1 | Vesicle Amine Transport 1 |
| UBE2Q1 | Ubiquitin Conjugating Enzyme E2 Q1 |
| TRIM71 | Tripartite Motif Containing 71 |
| RAP2C | RAP2C, Member Of RAS Oncogene Family |
| CPNE3 | Copine 3 |
| ATP6V0E1 | ATPase H+ Transporting V0 Subunit E1 |
| ANAPC13 | Anaphase Promoting Complex Subunit 13 |
| ATP8B4 | ATPase Phospholipid Transporting 8B4 (Putative) |
| SLCO4C1 | Solute Carrier Organic Anion Transporter Family Member 4C1 |
| SPSB2 | SplA/Ryanodine Receptor Domain And SOCS Box Containing 2 |
| RAB24 | RAB24, Member RAS Oncogene Family |
| KBTBD8 | Kelch Repeat And BTB Domain Containing 8 |
| KLHL5 | Kelch Like Family Member 5 |
| KIF18A | Kinesin Family Member 18A |
| STBD1 | Starch Binding Domain 1 |
| CTSO | Cathepsin O |
| DCTN3 | Dynactin Subunit 3 |
| DCTN5 | Dynactin Subunit 5 |
| RNF123 | Ring Finger Protein 123 |
| RNF126 | Ring Finger Protein 126 |
| METTL7A | Methyltransferase Like 7A |
| PDZD11 | PDZ Domain Containing 11 |
| POLR3GL | RNA Polymerase III Subunit G Like |
| RAB9B | RAB9B, Member RAS Oncogene Family |
| DCTN6 | Dynactin Subunit 6 |
| ATP6V0B | ATPase H+ Transporting V0 Subunit B |
| ASB10 | Ankyrin Repeat And SOCS Box Containing 10 |
| ASB7 | Ankyrin Repeat And SOCS Box Containing 7 |
| UBE2U | Ubiquitin Conjugating Enzyme E2 U |
| MUC15 | Mucin 15, Cell Surface Associated |
| ACTR10 | Actin Related Protein 10 |
| ASB11 | Ankyrin Repeat And SOCS Box Containing 11 |
| KCTD6 | Potassium Channel Tetramerization Domain Containing 6 |
| UBOX5 | U-Box Domain Containing 5 |
| SVIP | Small VCP Interacting Protein |
| DET1 | DET1 Partner Of COP1 E3 Ubiquitin Ligase |
| SPSB4 | SplA/Ryanodine Receptor Domain And SOCS Box Containing 4 |
| ASB5 | Ankyrin Repeat And SOCS Box Containing 5 |
| WIPF3 | WAS/WASL Interacting Protein Family Member 3 |
| KBTBD6 | Kelch Repeat And BTB Domain Containing 6 |
| KLHL42 | Kelch Like Family Member 42 |
| CYSTM1 | Cysteine Rich Transmembrane Module Containing 1 |
| NHLRC3 | NHL Repeat Containing 3 |
| ASB15 | Ankyrin Repeat And SOCS Box Containing 15 |
| KIF4B | Kinesin Family Member 4B |
| TMEM179B | Transmembrane Protein 179B |
| ASB18 | Ankyrin Repeat And SOCS Box Containing 18 |
| FRMPD3 | FERM And PDZ Domain Containing 3 |
| PLPP4 | Phospholipid Phosphatase 4 |
| EPPIN-WFDC6 | EPPIN-WFDC6 Readthrough |
| CBLL2 | Cbl Proto-Oncogene Like 2 |
| MUC3B | Mucin 3B, Cell Surface Associated |
| MIR3620 | MicroRNA 3620 |
| MIR5010 | MicroRNA 5010 |
| MIR4687 | MicroRNA 4687 |
| MIR1281 | MicroRNA 1281 |
| MIR4709 | MicroRNA 4709 |
| MIR6513 | MicroRNA 6513 |
| MIR6837 | MicroRNA 6837 |
| MIR6843 | MicroRNA 6843 |
| MIR6848 | MicroRNA 6848 |
| MIR4720 | MicroRNA 4720 |
| MIR6748 | MicroRNA 6748 |
| MIR6742 | MicroRNA 6742 |
| LOC105369632 | Uncharacterized LOC105369632 |
| LOC105372824 | Uncharacterized LOC105372824 |
| SERPINF1 | Serpin Family F Member 1 |
| ERVFC1-1 | Endogenous Retrovirus Group FC1 Member 1, Envelope |
| ERVS71-1 | Endogenous Retrovirus Group S71 Member 1, Envelope |
| ERVPABLB-1 | Endogenous Retrovirus Group PABLB Member 1, Envelope |
| ERVFC1 | Endogenous Retrovirus Group FC1 Env Polyprotein |
| XRN2 | 5'-3' Exoribonuclease 2 |
| CAPZB | Capping Actin Protein Of Muscle Z-Line Subunit Beta |
| DDX24 | DEAD-Box Helicase 24 |
| RALY | RALY Heterogeneous Nuclear Ribonucleoprotein |
| ACTBL2 | Actin Beta Like 2 |
| FASTKD5 | FAST Kinase Domains 5 |
| F7 | Coagulation Factor VII |
| MRPS22 | Mitochondrial Ribosomal Protein S22 |
| DHX15 | DEAH-Box Helicase 15 |
| EIF3K | Eukaryotic Translation Initiation Factor 3 Subunit K |
| ECD | Ecdysoneless Cell Cycle Regulator |
| WWTR1 | WW Domain Containing Transcription Regulator 1 |
| TNFAIP8 | TNF Alpha Induced Protein 8 |
| PPAN | Peter Pan Homolog |
| IGF2BP3 | Insulin Like Growth Factor 2 MRNA Binding Protein 3 |
| COPS2 | COP9 Signalosome Subunit 2 |
| SIX1 | SIX Homeobox 1 |
| PRPH2 | Peripherin 2 |
| BBS5 | Bardet-Biedl Syndrome 5 |
| HDAC7 | Histone Deacetylase 7 |
| ECHS1 | Enoyl-CoA Hydratase, Short Chain 1 |
| MTNR1B | Melatonin Receptor 1B |
| ADAM12 | ADAM Metallopeptidase Domain 12 |
| ZIC1 | Zic Family Member 1 |
| HMBS | Hydroxymethylbilane Synthase |
| FABP4 | Fatty Acid Binding Protein 4 |
| SPINT2 | Serine Peptidase Inhibitor, Kunitz Type 2 |
| CRABP2 | Cellular Retinoic Acid Binding Protein 2 |
| ETV4 | ETS Variant Transcription Factor 4 |
| AMH | Anti-Mullerian Hormone |
| WNT7B | Wnt Family Member 7B |
| TES | Testin LIM Domain Protein |
| SLC17A3 | Solute Carrier Family 17 Member 3 |
| SLC30A5 | Solute Carrier Family 30 Member 5 |
| STXBP3 | Syntaxin Binding Protein 3 |
| DSCAM | DS Cell Adhesion Molecule |
| CRTC3 | CREB Regulated Transcription Coactivator 3 |
| GGA3 | Golgi Associated, Gamma Adaptin Ear Containing, ARF Binding Protein 3 |
| ANKLE2 | Ankyrin Repeat And LEM Domain Containing 2 |
| SLC39A6 | Solute Carrier Family 39 Member 6 |
| CELF1 | CUGBP Elav-Like Family Member 1 |
| CCAR1 | Cell Division Cycle And Apoptosis Regulator 1 |
| TMPRSS13 | Transmembrane Serine Protease 13 |
| DSPP | Dentin Sialophosphoprotein |
| RER1 | Retention In Endoplasmic Reticulum Sorting Receptor 1 |
| RBM24 | RNA Binding Motif Protein 24 |
| B3GLCT | Beta 3-Glucosyltransferase |
| UCMA | Upper Zone Of Growth Plate And Cartilage Matrix Associated |
| C1orf141 | Chromosome 1 Open Reading Frame 141 |
| MIR27B | MicroRNA 27b |
| MIR378A | MicroRNA 378a |
| MYOF | Myoferlin |
| ZFR | Zinc Finger RNA Binding Protein |
| FUT7 | Fucosyltransferase 7 |
| DNASE2 | Deoxyribonuclease 2, Lysosomal |
| SNX27 | Sorting Nexin 27 |
| TRIM23 | Tripartite Motif Containing 23 |
| SUMO2 | Small Ubiquitin Like Modifier 2 |
| HGS | Hepatocyte Growth Factor-Regulated Tyrosine Kinase Substrate |
| PDCD11 | Programmed Cell Death 11 |
| CSMD2 | CUB And Sushi Multiple Domains 2 |
| FGD2 | FYVE, RhoGEF And PH Domain Containing 2 |
| SSX4 | SSX Family Member 4 |
| DCANP1 | Dendritic Cell Associated Nuclear Protein |
| CPQ | Carboxypeptidase Q |
| EPHX2 | Epoxide Hydrolase 2 |
| ILRUN | Inflammation And Lipid Regulator With UBA-Like And NBR1-Like Domains |
| DNAJB1 | DnaJ Heat Shock Protein Family (Hsp40) Member B1 |
| ITFG1 | Integrin Alpha FG-GAP Repeat Containing 1 |
| ERBIN | Erbb2 Interacting Protein |
| CHD2 | Chromodomain Helicase DNA Binding Protein 2 |
| UCHL3 | Ubiquitin C-Terminal Hydrolase L3 |
| PNO1 | Partner Of NOB1 Homolog |
| APEX2 | Apurinic/Apyrimidinic Endodeoxyribonuclease 2 |
| TDRD3 | Tudor Domain Containing 3 |
| AKR1B10 | Aldo-Keto Reductase Family 1 Member B10 |
| MAP1A | Microtubule Associated Protein 1A |
| MYNN | Myoneurin |
| SEL1L | SEL1L Adaptor Subunit Of ERAD E3 Ubiquitin Ligase |
| UNC5B | Unc-5 Netrin Receptor B |
| SAMSN1 | SAM Domain, SH3 Domain And Nuclear Localization Signals 1 |
| COPG2 | COPI Coat Complex Subunit Gamma 2 |
| FKBP1A | FKBP Prolyl Isomerase 1A |
| EEF1D | Eukaryotic Translation Elongation Factor 1 Delta |
| PDE3A | Phosphodiesterase 3A |
| GCGR | Glucagon Receptor |
| TNK2 | Tyrosine Kinase Non Receptor 2 |
| AMD1 | Adenosylmethionine Decarboxylase 1 |
| MAOB | Monoamine Oxidase B |
| DCXR | Dicarbonyl And L-Xylulose Reductase |
| APOA5 | Apolipoprotein A5 |
| SLC5A3 | Solute Carrier Family 5 Member 3 |
| MMP17 | Matrix Metallopeptidase 17 |
| AKR7A2 | Aldo-Keto Reductase Family 7 Member A2 |
| ARX | Aristaless Related Homeobox |
| STIL | STIL Centriolar Assembly Protein |
| ERC1 | ELKS/RAB6-Interacting/CAST Family Member 1 |
| TRPM3 | Transient Receptor Potential Cation Channel Subfamily M Member 3 |
| MMADHC | Metabolism Of Cobalamin Associated D |
| ABCC11 | ATP Binding Cassette Subfamily C Member 11 |
| UGT2B17 | UDP Glucuronosyltransferase Family 2 Member B17 |
| SLC35D1 | Solute Carrier Family 35 Member D1 |
| SLC17A1 | Solute Carrier Family 17 Member 1 |
| TGM4 | Transglutaminase 4 |
| RFX3 | Regulatory Factor X3 |
| HBZ | Hemoglobin Subunit Zeta |
| MRRF | Mitochondrial Ribosome Recycling Factor |
| GPR158 | G Protein-Coupled Receptor 158 |
| ARHGAP24 | Rho GTPase Activating Protein 24 |
| ARHGAP32 | Rho GTPase Activating Protein 32 |
| TMEFF2 | Transmembrane Protein With EGF Like And Two Follistatin Like Domains 2 |
| DENR | Density Regulated Re-Initiation And Release Factor |
| MAML2 | Mastermind Like Transcriptional Coactivator 2 |
| ANKS6 | Ankyrin Repeat And Sterile Alpha Motif Domain Containing 6 |
| SLC8B1 | Solute Carrier Family 8 Member B1 |
| ELF5 | E74 Like ETS Transcription Factor 5 |
| DENND5A | DENN Domain Containing 5A |
| TMPRSS11A | Transmembrane Serine Protease 11A |
| GREB1 | Growth Regulating Estrogen Receptor Binding 1 |
| SACS | Sacsin Molecular Chaperone |
| FNBP1L | Formin Binding Protein 1 Like |
| UST | Uronyl 2-Sulfotransferase |
| ZBTB48 | Zinc Finger And BTB Domain Containing 48 |
| THAP3 | THAP Domain Containing 3 |
| CEP83 | Centrosomal Protein 83 |
| MACROD2 | Mono-ADP Ribosylhydrolase 2 |
| ZPBP2 | Zona Pellucida Binding Protein 2 |
| EFHC2 | EF-Hand Domain Containing 2 |
| RMI2 | RecQ Mediated Genome Instability 2 |
| APOOL | Apolipoprotein O Like |
| TMEM119 | Transmembrane Protein 119 |
| CWC15 | CWC15 Spliceosome Associated Protein Homolog |
| CCDC122 | Coiled-Coil Domain Containing 122 |
| ZSCAN9 | Zinc Finger And SCAN Domain Containing 9 |
| H4C11 | H4 Clustered Histone 11 |
| ANGPTL8 | Angiopoietin Like 8 |
| POTEF | POTE Ankyrin Domain Family Member F |
| CARMIL1 | Capping Protein Regulator And Myosin 1 Linker 1 |
| NBPF14 | NBPF Member 14 |
| CRNDE | Colorectal Neoplasia Differentially Expressed |
| MIR188 | MicroRNA 188 |
| PANDAR | Promoter Of CDKN1A Antisense DNA Damage Activated RNA |
| ENSG00000080572 |  |
| TSBP1-AS1 | TSBP1 And BTNL2 Antisense RNA 1 |
| C1QTNF5 | C1q And TNF Related 5 |
| BBC3 | BCL2 Binding Component 3 |
| DTX1 | Deltex E3 Ubiquitin Ligase 1 |
| PHF20 | PHD Finger Protein 20 |
| POMGNT2 | Protein O-Linked Mannose N-Acetylglucosaminyltransferase 2 (Beta 1,4-) |
| ADAM15 | ADAM Metallopeptidase Domain 15 |
| VPS35 | VPS35 Retromer Complex Component |
| FUT3 | Fucosyltransferase 3 (Lewis Blood Group) |
| FARSB | Phenylalanyl-TRNA Synthetase Subunit Beta |
| MAP1B | Microtubule Associated Protein 1B |
| GNL3 | G Protein Nucleolar 3 |
| BTF3 | Basic Transcription Factor 3 |
| CALU | Calumenin |
| SCAMP3 | Secretory Carrier Membrane Protein 3 |
| NOB1 | NIN1 (RPN12) Binding Protein 1 Homolog |
| ANKRD13A | Ankyrin Repeat Domain 13A |
| HACD3 | 3-Hydroxyacyl-CoA Dehydratase 3 |
| UTP18 | UTP18 Small Subunit Processome Component |
| TNIP3 | TNFAIP3 Interacting Protein 3 |
| EPHA1 | EPH Receptor A1 |
| GNA13 | G Protein Subunit Alpha 13 |
| PMS1 | PMS1 Homolog 1, Mismatch Repair System Component |
| MYH14 | Myosin Heavy Chain 14 |
| CYTL1 | Cytokine Like 1 |
| SENP7 | SUMO Specific Peptidase 7 |
| LOC110806262 | Solute Carrier Family 6 Member 4 Gene Promoter |
| ADGRA1 | Adhesion G Protein-Coupled Receptor A1 |
| CLEC18A | C-Type Lectin Domain Family 18 Member A |
| TAFA2 | TAFA Chemokine Like Family Member 2 |
| NEK2 | NIMA Related Kinase 2 |
| PTCH2 | Patched 2 |
| ALDOB | Aldolase, Fructose-Bisphosphate B |
| ABCA4 | ATP Binding Cassette Subfamily A Member 4 |
| CRB1 | Crumbs Cell Polarity Complex Component 1 |
| FIG4 | FIG4 Phosphoinositide 5-Phosphatase |
| FAM20C | FAM20C Golgi Associated Secretory Pathway Kinase |
| RBM39 | RNA Binding Motif Protein 39 |
| CLGN | Calmegin |
| EHD2 | EH Domain Containing 2 |
| FAM120A | Family With Sequence Similarity 120A |
| APOBEC2 | Apolipoprotein B MRNA Editing Enzyme Catalytic Subunit 2 |
| PDS5A | PDS5 Cohesin Associated Factor A |
| DDX47 | DEAD-Box Helicase 47 |
| TM9SF3 | Transmembrane 9 Superfamily Member 3 |
| IRF2BP1 | Interferon Regulatory Factor 2 Binding Protein 1 |
| SDAD1 | SDA1 Domain Containing 1 |
| PALD1 | Phosphatase Domain Containing Paladin 1 |
| SUCNR1 | Succinate Receptor 1 |
| CHST4 | Carbohydrate Sulfotransferase 4 |
| FCRLB | Fc Receptor Like B |
| SASH3 | SAM And SH3 Domain Containing 3 |
| P2RY2 | Purinergic Receptor P2Y2 |
| H2BC12 | H2B Clustered Histone 12 |
| NCL | Nucleolin |
| PHB2 | Prohibitin 2 |
| SREBF2 | Sterol Regulatory Element Binding Transcription Factor 2 |
| SLC1A3 | Solute Carrier Family 1 Member 3 |
| SCN2A | Sodium Voltage-Gated Channel Alpha Subunit 2 |
| PAX8 | Paired Box 8 |
| SLC18A2 | Solute Carrier Family 18 Member A2 |
| CDH11 | Cadherin 11 |
| KCNQ2 | Potassium Voltage-Gated Channel Subfamily Q Member 2 |
| ALDH1A3 | Aldehyde Dehydrogenase 1 Family Member A3 |
| MVD | Mevalonate Diphosphate Decarboxylase |
| SCN1B | Sodium Voltage-Gated Channel Beta Subunit 1 |
| HTR4 | 5-Hydroxytryptamine Receptor 4 |
| SPRY4 | Sprouty RTK Signaling Antagonist 4 |
| CNDP1 | Carnosine Dipeptidase 1 |
| FZD1 | Frizzled Class Receptor 1 |
| WARS2 | Tryptophanyl TRNA Synthetase 2, Mitochondrial |
| UGT2B7 | UDP Glucuronosyltransferase Family 2 Member B7 |
| WNT11 | Wnt Family Member 11 |
| KCNJ12 | Potassium Inwardly Rectifying Channel Subfamily J Member 12 |
| NFIB | Nuclear Factor I B |
| HIBCH | 3-Hydroxyisobutyryl-CoA Hydrolase |
| CDT1 | Chromatin Licensing And DNA Replication Factor 1 |
| UGT1A9 | UDP Glucuronosyltransferase Family 1 Member A9 |
| LINGO1 | Leucine Rich Repeat And Ig Domain Containing 1 |
| NPY5R | Neuropeptide Y Receptor Y5 |
| SLC6A11 | Solute Carrier Family 6 Member 11 |
| MPC1 | Mitochondrial Pyruvate Carrier 1 |
| MINK1 | Misshapen Like Kinase 1 |
| MTO1 | Mitochondrial TRNA Translation Optimization 1 |
| CDKL5 | Cyclin Dependent Kinase Like 5 |
| MCEE | Methylmalonyl-CoA Epimerase |
| ADH1A | Alcohol Dehydrogenase 1A (Class I), Alpha Polypeptide |
| ADPGK | ADP Dependent Glucokinase |
| ETHE1 | ETHE1 Persulfide Dioxygenase |
| B4GALT7 | Beta-1,4-Galactosyltransferase 7 |
| ASAH2 | N-Acylsphingosine Amidohydrolase 2 |
| SLCO4A1 | Solute Carrier Organic Anion Transporter Family Member 4A1 |
| TFB1M | Transcription Factor B1, Mitochondrial |
| NDUFAF1 | NADH:Ubiquinone Oxidoreductase Complex Assembly Factor 1 |
| KCNA4 | Potassium Voltage-Gated Channel Subfamily A Member 4 |
| NKX2-2 | NK2 Homeobox 2 |
| PKIB | CAMP-Dependent Protein Kinase Inhibitor Beta |
| RARRES1 | Retinoic Acid Receptor Responder 1 |
| SLC22A11 | Solute Carrier Family 22 Member 11 |
| SIM1 | SIM BHLH Transcription Factor 1 |
| TIMM17A | Translocase Of Inner Mitochondrial Membrane 17A |
| KCNAB1 | Potassium Voltage-Gated Channel Subfamily A Member Regulatory Beta Subunit 1 |
| CXXC1 | CXXC Finger Protein 1 |
| RFX6 | Regulatory Factor X6 |
| DEPDC5 | DEP Domain Containing 5, GATOR1 Subcomplex Subunit |
| OLFM1 | Olfactomedin 1 |
| B3GALNT2 | Beta-1,3-N-Acetylgalactosaminyltransferase 2 |
| BRSK2 | BR Serine/Threonine Kinase 2 |
| UBE4B | Ubiquitination Factor E4B |
| PIGH | Phosphatidylinositol Glycan Anchor Biosynthesis Class H |
| GLRA3 | Glycine Receptor Alpha 3 |
| TET3 | Tet Methylcytosine Dioxygenase 3 |
| DSCAML1 | DS Cell Adhesion Molecule Like 1 |
| CEP135 | Centrosomal Protein 135 |
| AGAP1 | ArfGAP With GTPase Domain, Ankyrin Repeat And PH Domain 1 |
| C2CD3 | C2 Domain Containing 3 Centriole Elongation Regulator |
| ARL2 | ADP Ribosylation Factor Like GTPase 2 |
| ASPN | Asporin |
| TXNDC12 | Thioredoxin Domain Containing 12 |
| ZNF23 | Zinc Finger Protein 23 |
| USP6 | Ubiquitin Specific Peptidase 6 |
| ZBTB18 | Zinc Finger And BTB Domain Containing 18 |
| SLC35B2 | Solute Carrier Family 35 Member B2 |
| PMEPA1 | Prostate Transmembrane Protein, Androgen Induced 1 |
| POU4F2 | POU Class 4 Homeobox 2 |
| RASD1 | Ras Related Dexamethasone Induced 1 |
| SLC17A9 | Solute Carrier Family 17 Member 9 |
| STXBP5 | Syntaxin Binding Protein 5 |
| OMD | Osteomodulin |
| CHPF2 | Chondroitin Polymerizing Factor 2 |
| RPRD1B | Regulation Of Nuclear Pre-MRNA Domain Containing 1B |
| AMY2A | Amylase Alpha 2A |
| ARL13B | ADP Ribosylation Factor Like GTPase 13B |
| PMF1 | Polyamine Modulated Factor 1 |
| PRUNE2 | Prune Homolog 2 With BCH Domain |
| NAA50 | N-Alpha-Acetyltransferase 50, NatE Catalytic Subunit |
| FCHSD1 | FCH And Double SH3 Domains 1 |
| SLC39A11 | Solute Carrier Family 39 Member 11 |
| INTS6 | Integrator Complex Subunit 6 |
| SLC22A23 | Solute Carrier Family 22 Member 23 |
| POGK | Pogo Transposable Element Derived With KRAB Domain |
| JAKMIP1 | Janus Kinase And Microtubule Interacting Protein 1 |
| TBCC | Tubulin Folding Cofactor C |
| QSOX2 | Quiescin Sulfhydryl Oxidase 2 |
| SOX21 | SRY-Box Transcription Factor 21 |
| CUTA | CutA Divalent Cation Tolerance Homolog |
| DNAH17 | Dynein Axonemal Heavy Chain 17 |
| SEMA4F | Ssemaphorin 4F |
| ORC5 | Origin Recognition Complex Subunit 5 |
| CENPQ | Centromere Protein Q |
| AHDC1 | AT-Hook DNA Binding Motif Containing 1 |
| FAM107A | Family With Sequence Similarity 107 Member A |
| AMOTL2 | Angiomotin Like 2 |
| WDR81 | WD Repeat Domain 81 |
| WDR66 | WD Repeat Domain 66 |
| ZBTB33 | Zinc Finger And BTB Domain Containing 33 |
| HSPB11 | Heat Shock Protein Family B (Small) Member 11 |
| TCTE1 | T-Complex-Associated-Testis-Expressed 1 |
| TCTE3 | T-Complex-Associated-Testis-Expressed 3 |
| DNAH10 | Dynein Axonemal Heavy Chain 10 |
| MTERF1 | Mitochondrial Transcription Termination Factor 1 |
| MTHFSD | Methenyltetrahydrofolate Synthetase Domain Containing |
| MCU | Mitochondrial Calcium Uniporter |
| LYPD4 | LY6/PLAUR Domain Containing 4 |
| CAGE1 | Cancer Antigen 1 |
| CDH26 | Cadherin 26 |
| PRAF2 | PRA1 Domain Family Member 2 |
| SNRNP48 | Small Nuclear Ribonucleoprotein U11/U12 Subunit 48 |
| SYDE1 | Synapse Defective Rho GTPase Homolog 1 |
| MNS1 | Meiosis Specific Nuclear Structural 1 |
| NBPF3 | NBPF Member 3 |
| DCLRE1B | DNA Cross-Link Repair 1B |
| DNAH12 | Dynein Axonemal Heavy Chain 12 |
| OR51E2 | Olfactory Receptor Family 51 Subfamily E Member 2 |
| CEP131 | Centrosomal Protein 131 |
| CDC42SE1 | CDC42 Small Effector 1 |
| MARVELD3 | MARVEL Domain Containing 3 |
| CABP1 | Calcium Binding Protein 1 |
| APOLD1 | Apolipoprotein L Domain Containing 1 |
| TSPY1 | Testis Specific Protein Y-Linked 1 |
| KLHL17 | Kelch Like Family Member 17 |
| NDNF | Neuron Derived Neurotrophic Factor |
| TTC17 | Tetratricopeptide Repeat Domain 17 |
| SOGA3 | SOGA Family Member 3 |
| DNAH6 | Dynein Axonemal Heavy Chain 6 |
| GPAT3 | Glycerol-3-Phosphate Acyltransferase 3 |
| NWD1 | NACHT And WD Repeat Domain Containing 1 |
| ASCL3 | Achaete-Scute Family BHLH Transcription Factor 3 |
| LGALS7B | Galectin 7B |
| GLRA4 | Glycine Receptor Alpha 4 (Pseudogene) |
| NBPF1 | NBPF Member 1 |
| PAGE4 | PAGE Family Member 4 |
| GDPD4 | Glycerophosphodiester Phosphodiesterase Domain Containing 4 |
| CCDC180 | Coiled-Coil Domain Containing 180 |
| EDDM3B | Epididymal Protein 3B |
| SCGB1D1 | Secretoglobin Family 1D Member 1 |
| CGB7 | Chorionic Gonadotropin Subunit Beta 7 |
| SPANXD | SPANX Family Member D |
| ZNF783 | Zinc Finger Family Member 783 |
| CFAP74 | Cilia And Flagella Associated Protein 74 |
| DOP1A | DOP1 Leucine Zipper Like Protein A |
| USF3 | Upstream Transcription Factor Family Member 3 |
| CLPSL2 | Colipase Like 2 |
| KCNQ1OT1 | KCNQ1 Opposite Strand/Antisense Transcript 1 |
| NEMP2 | Nuclear Envelope Integral Membrane Protein 2 |
| CFAP54 | Cilia And Flagella Associated Protein 54 |
| PCA3 | Prostate Cancer Associated 3 |
| RBM14-RBM4 | RBM14-RBM4 Readthrough |
| DANCR | Differentiation Antagonizing Non-Protein Coding RNA |
| MIR191 | MicroRNA 191 |
| HAND2-AS1 | HAND2 Antisense RNA 1 |
| PCAT18 | Prostate Cancer Associated Transcript 18 |
| MIR33A | MicroRNA 33a |
| MIR628 | MicroRNA 628 |
| FENDRR | FOXF1 Adjacent Non-Coding Developmental Regulatory RNA |
| TRAF3IP2-AS1 | TRAF3IP2 Antisense RNA 1 |
| GUSBP2 | GUSB Pseudogene 2 |
| MICOS10-NBL1 | MICOS10-NBL1 Readthrough |
| MT-RNR1 | Mitochondrially Encoded 12S RRNA |
| NBPF22P | NBPF Member 22, Pseudogene |
| LINC00221 | Long Intergenic Non-Protein Coding RNA 221 |
| NBPF19 | NBPF Member 19 |
| NBPF26 | NBPF Member 26 |
| SUZ12P1 | SUZ12 Pseudogene 1 |
| CTBP1-AS | CTBP1 Antisense RNA |
| NBPF25P | NBPF Member 25, Pseudogene |
| HBA-LCR | Alpha-Globin Locus Control Region |
| LOC107988021 | IKBKG Recombination Region |
| LOC107988022 | IKBKG Downstream Recombination Region |
| KMO | Kynurenine 3-Monooxygenase |
| MTHFD2 | Methylenetetrahydrofolate Dehydrogenase (NADP+ Dependent) 2, Methenyltetrahydrofolate Cyclohydrolase |
| IGFBP5 | Insulin Like Growth Factor Binding Protein 5 |
| CHMP4B | Charged Multivesicular Body Protein 4B |
| FLOT2 | Flotillin 2 |
| TRIOBP | TRIO And F-Actin Binding Protein |
| MRPS9 | Mitochondrial Ribosomal Protein S9 |
| DAP3 | Death Associated Protein 3 |
| GLMN | Glomulin, FKBP Associated Protein |
| PARP12 | Poly(ADP-Ribose) Polymerase Family Member 12 |
| SRRM1 | Serine And Arginine Repetitive Matrix 1 |
| MRPS23 | Mitochondrial Ribosomal Protein S23 |
| YIPF5 | Yip1 Domain Family Member 5 |
| TNKS1BP1 | Tankyrase 1 Binding Protein 1 |
| LARP4B | La Ribonucleoprotein 4B |
| RPL7L1 | Ribosomal Protein L7 Like 1 |
| TMEM259 | Transmembrane Protein 259 |
| NCBP3 | Nuclear Cap Binding Subunit 3 |
| HDAC3 | Histone Deacetylase 3 |
| SLC25A4 | Solute Carrier Family 25 Member 4 |
| MAP2K5 | Mitogen-Activated Protein Kinase Kinase 5 |
| PLA2G12A | Phospholipase A2 Group XIIA |
| TEP1 | Telomerase Associated Protein 1 |
| EEF1G | Eukaryotic Translation Elongation Factor 1 Gamma |
| PVRIG | PVR Related Immunoglobulin Domain Containing |
| SAFB | Scaffold Attachment Factor B |
| FOXM1 | Forkhead Box M1 |
| SLC9A4 | Solute Carrier Family 9 Member A4 |
| PRSS16 | Serine Protease 16 |
| PREX1 | Phosphatidylinositol-3,4,5-Trisphosphate Dependent Rac Exchange Factor 1 |
| FOLR1 | Folate Receptor Alpha |
| RPL3 | Ribosomal Protein L3 |
| DHX30 | DExH-Box Helicase 30 |
| CSTF1 | Cleavage Stimulation Factor Subunit 1 |
| MRPS17 | Mitochondrial Ribosomal Protein S17 |
| MRPS2 | Mitochondrial Ribosomal Protein S2 |
| MRPS25 | Mitochondrial Ribosomal Protein S25 |
| CHP1 | Calcineurin Like EF-Hand Protein 1 |
| EXOC5 | Exocyst Complex Component 5 |
| EHD4 | EH Domain Containing 4 |
| MRPS18B | Mitochondrial Ribosomal Protein S18B |
| SURF6 | Surfeit 6 |
| ANKRD28 | Ankyrin Repeat Domain 28 |
| SELENOF | Selenoprotein F |
| SRP68 | Signal Recognition Particle 68 |
| GSTM3 | Glutathione S-Transferase Mu 3 |
| IFRD1 | Interferon Related Developmental Regulator 1 |
| PSG1 | Pregnancy Specific Beta-1-Glycoprotein 1 |
| GBF1 | Golgi Brefeldin A Resistant Guanine Nucleotide Exchange Factor 1 |
| TRPV3 | Transient Receptor Potential Cation Channel Subfamily V Member 3 |
| TMEM127 | Transmembrane Protein 127 |
| BBS12 | Bardet-Biedl Syndrome 12 |
| SNRPD2 | Small Nuclear Ribonucleoprotein D2 Polypeptide |
| THOC7 | THO Complex 7 |
| H1-5 | H1.5 Linker Histone, Cluster Member |
| PARP14 | Poly(ADP-Ribose) Polymerase Family Member 14 |
| CLNK | Cytokine Dependent Hematopoietic Cell Linker |
| ENDOU | Endonuclease, Poly(U) Specific |
| GPSM3 | G Protein Signaling Modulator 3 |
| GPR31 | G Protein-Coupled Receptor 31 |
| DNAJA1 | DnaJ Heat Shock Protein Family (Hsp40) Member A1 |
| ATP5F1A | ATP Synthase F1 Subunit Alpha |
| ATP5F1B | ATP Synthase F1 Subunit Beta |
| EXOSC3 | Exosome Component 3 |
| OSBPL8 | Oxysterol Binding Protein Like 8 |
| PNN | Pinin, Desmosome Associated Protein |
| SNU13 | Small Nuclear Ribonucleoprotein 13 |
| HES1 | Hes Family BHLH Transcription Factor 1 |
| TSTA3 | Tissue Specific Transplantation Antigen P35B |
| ADPRH | ADP-Ribosylarginine Hydrolase |
| PCID2 | PCI Domain Containing 2 |
| ITGB6 | Integrin Subunit Beta 6 |
| NPR1 | Natriuretic Peptide Receptor 1 |
| KLF10 | Kruppel Like Factor 10 |
| CYP2A6 | Cytochrome P450 Family 2 Subfamily A Member 6 |
| PARP2 | Poly(ADP-Ribose) Polymerase 2 |
| ASCC1 | Activating Signal Cointegrator 1 Complex Subunit 1 |
| UQCRC1 | Ubiquinol-Cytochrome C Reductase Core Protein 1 |
| MRPS7 | Mitochondrial Ribosomal Protein S7 |
| MRPS28 | Mitochondrial Ribosomal Protein S28 |
| TSR1 | TSR1 Ribosome Maturation Factor |
| MRPS34 | Mitochondrial Ribosomal Protein S34 |
| TUBGCP2 | Tubulin Gamma Complex Associated Protein 2 |
| PURB | Purine Rich Element Binding Protein B |
| MRPS35 | Mitochondrial Ribosomal Protein S35 |
| MRPS5 | Mitochondrial Ribosomal Protein S5 |
| MRPS27 | Mitochondrial Ribosomal Protein S27 |
| MRPS31 | Mitochondrial Ribosomal Protein S31 |
| PTCD3 | Pentatricopeptide Repeat Domain 3 |
| MRPS15 | Mitochondrial Ribosomal Protein S15 |
| MAP7D1 | MAP7 Domain Containing 1 |
| LLPH | LLP Homolog, Long-Term Synaptic Facilitation Factor |
| SYNE2 | Spectrin Repeat Containing Nuclear Envelope Protein 2 |
| NMI | N-Myc And STAT Interactor |
| TMPRSS12 | Transmembrane Serine Protease 12 |
| OVCH1 | Ovochymase 1 |
| OVCH2 | Ovochymase 2 (Gene/Pseudogene) |
| HNRNPR | Heterogeneous Nuclear Ribonucleoprotein R |
| DUSP19 | Dual Specificity Phosphatase 19 |
| SERBP1 | SERPINE1 MRNA Binding Protein 1 |
| NCBP1 | Nuclear Cap Binding Protein Subunit 1 |
| MPV17L2 | MPV17 Mitochondrial Inner Membrane Protein Like 2 |
| PRKAB2 | Protein Kinase AMP-Activated Non-Catalytic Subunit Beta 2 |
| MEF2D | Myocyte Enhancer Factor 2D |
| ANGPT4 | Angiopoietin 4 |
| TOB1 | Transducer Of ERBB2, 1 |
| RAMP3 | Receptor Activity Modifying Protein 3 |
| ARL14EP | ADP Ribosylation Factor Like GTPase 14 Effector Protein |
| TRIB1 | Tribbles Pseudokinase 1 |
| SEZ6L2 | Seizure Related 6 Homolog Like 2 |
| EIF3A | Eukaryotic Translation Initiation Factor 3 Subunit A |
| SNRPA1 | Small Nuclear Ribonucleoprotein Polypeptide A' |
| NLRP11 | NLR Family Pyrin Domain Containing 11 |
| SHMT2 | Serine Hydroxymethyltransferase 2 |
| TENM1 | Teneurin Transmembrane Protein 1 |
| MT3 | Metallothionein 3 |
| CHMP5 | Charged Multivesicular Body Protein 5 |
| RAI14 | Retinoic Acid Induced 14 |
| SPATS2L | Spermatogenesis Associated Serine Rich 2 Like |
| SEPTIN2 | Septin 2 |
| RFC3 | Replication Factor C Subunit 3 |
| USP36 | Ubiquitin Specific Peptidase 36 |
| POLDIP3 | DNA Polymerase Delta Interacting Protein 3 |
| CEBPZ | CCAAT Enhancer Binding Protein Zeta |
| DDX56 | DEAD-Box Helicase 56 |
| RPF2 | Ribosome Production Factor 2 Homolog |
| SF3B4 | Splicing Factor 3b Subunit 4 |
| EFS | Embryonal Fyn-Associated Substrate |
| CCN4 | Cellular Communication Network Factor 4 |
| P2RY1 | Purinergic Receptor P2Y1 |
| S100A6 | S100 Calcium Binding Protein A6 |
| MARS1 | Methionyl-TRNA Synthetase 1 |
| PGD | Phosphogluconate Dehydrogenase |
| UNC45A | Unc-45 Myosin Chaperone A |
| SEMA3F | Semaphorin 3F |
| CBFA2T3 | CBFA2/RUNX1 Partner Transcriptional Co-Repressor 3 |
| FASTK | Fas Activated Serine/Threonine Kinase |
| RGS13 | Regulator Of G Protein Signaling 13 |
| SEPTIN7 | Septin 7 |
| MARCHF3 | Membrane Associated Ring-CH-Type Finger 3 |
| MMRN1 | Multimerin 1 |
| HEATR1 | HEAT Repeat Containing 1 |
| SART3 | Spliceosome Associated Factor 3, U4/U6 Recycling Protein |
| DNAJC10 | DnaJ Heat Shock Protein Family (Hsp40) Member C10 |
| RPL36A | Ribosomal Protein L36a |
| RRP12 | Ribosomal RNA Processing 12 Homolog |
| GAB3 | GRB2 Associated Binding Protein 3 |
| RRP1 | Ribosomal RNA Processing 1 |
| MTREX | Mtr4 Exosome RNA Helicase |
| CMAS | Cytidine Monophosphate N-Acetylneuraminic Acid Synthetase |
| IGSF21 | Immunoglobin Superfamily Member 21 |
| INHBE | Inhibin Subunit Beta E |
| PSG4 | Pregnancy Specific Beta-1-Glycoprotein 4 |
| TAFA4 | TAFA Chemokine Like Family Member 4 |
| TAFA1 | TAFA Chemokine Like Family Member 1 |
| SSX2B | SSX Family Member 2B |
| SSX4B | SSX Family Member 4B |
| TAFA3 | TAFA Chemokine Like Family Member 3 |
| TAFA5 | TAFA Chemokine Like Family Member 5 |
| IGSF23 | Immunoglobulin Superfamily Member 23 |
| SSX7 | SSX Family Member 7 |
| SSX6P | SSX Family Member 6, Pseudogene |
| SSX9P | SSX Family Member 9, Pseudogene |
| PTPRS | Protein Tyrosine Phosphatase Receptor Type S |
| GDF6 | Growth Differentiation Factor 6 |
| ABCB6 | ATP Binding Cassette Subfamily B Member 6 (Langereis Blood Group) |
| PARP4 | Poly(ADP-Ribose) Polymerase Family Member 4 |
| SLC15A2 | Solute Carrier Family 15 Member 2 |
| ZNF350 | Zinc Finger Protein 350 |
| CARD10 | Caspase Recruitment Domain Family Member 10 |
| UBAP2L | Ubiquitin Associated Protein 2 Like |
| ST2 | Suppression Of Tumorigenicity 2 |
| TLN2 | Talin 2 |
| ARHGAP35 | Rho GTPase Activating Protein 35 |
| CYP2C9 | Cytochrome P450 Family 2 Subfamily C Member 9 |
| IGHV1OR15-1 | Immunoglobulin Heavy Variable 1/OR15-1 (Non-Functional) |
| ENSG00000250264 |  |
| ZP4 | Zona Pellucida Glycoprotein 4 |
| ARHGAP5 | Rho GTPase Activating Protein 5 |
| AIFM1 | Apoptosis Inducing Factor Mitochondria Associated 1 |
| GANAB | Glucosidase II Alpha Subunit |
| RPS20 | Ribosomal Protein S20 |
| DAAM1 | Dishevelled Associated Activator Of Morphogenesis 1 |
| COA8 | Cytochrome C Oxidase Assembly Factor 8 |
| PLIN3 | Perilipin 3 |
| TRAPPC3 | Trafficking Protein Particle Complex 3 |
| GNL2 | G Protein Nucleolar 2 |
| WDR3 | WD Repeat Domain 3 |
| RRP8 | Ribosomal RNA Processing 8 |
| COMMD4 | COMM Domain Containing 4 |
| NOL6 | Nucleolar Protein 6 |
| NOC4L | Nucleolar Complex Associated 4 Homolog |
| LSM12 | LSM12 Homolog |
| PUM3 | Pumilio RNA Binding Family Member 3 |
| SRSF3 | Serine And Arginine Rich Splicing Factor 3 |
| GYS1 | Glycogen Synthase 1 |
| WNT7A | Wnt Family Member 7A |
| HSD17B3 | Hydroxysteroid 17-Beta Dehydrogenase 3 |
| PCK1 | Phosphoenolpyruvate Carboxykinase 1 |
| GABBR2 | Gamma-Aminobutyric Acid Type B Receptor Subunit 2 |
| ALDH7A1 | Aldehyde Dehydrogenase 7 Family Member A1 |
| AKR1C2 | Aldo-Keto Reductase Family 1 Member C2 |
| SHMT1 | Serine Hydroxymethyltransferase 1 |
| HTR7 | 5-Hydroxytryptamine Receptor 7 |
| SLC26A3 | Solute Carrier Family 26 Member 3 |
| GRIK1 | Glutamate Ionotropic Receptor Kainate Type Subunit 1 |
| GPT2 | Glutamic--Pyruvic Transaminase 2 |
| GABRA2 | Gamma-Aminobutyric Acid Type A Receptor Subunit Alpha2 |
| ETV1 | ETS Variant Transcription Factor 1 |
| ATP2B3 | ATPase Plasma Membrane Ca2+ Transporting 3 |
| XYLT2 | Xylosyltransferase 2 |
| KCNJ3 | Potassium Inwardly Rectifying Channel Subfamily J Member 3 |
| SORD | Sorbitol Dehydrogenase |
| LARS2 | Leucyl-TRNA Synthetase 2, Mitochondrial |
| NTHL1 | Nth Like DNA Glycosylase 1 |
| SCN2B | Sodium Voltage-Gated Channel Beta Subunit 2 |
| PNPLA6 | Patatin Like Phospholipase Domain Containing 6 |
| POMGNT1 | Protein O-Linked Mannose N-Acetylglucosaminyltransferase 1 (Beta 1,2-) |
| MTM1 | Myotubularin 1 |
| GIPR | Gastric Inhibitory Polypeptide Receptor |
| MAPK8IP1 | Mitogen-Activated Protein Kinase 8 Interacting Protein 1 |
| AVPR1B | Arginine Vasopressin Receptor 1B |
| FDXR | Ferredoxin Reductase |
| FLRT3 | Fibronectin Leucine Rich Transmembrane Protein 3 |
| MECR | Mitochondrial Trans-2-Enoyl-CoA Reductase |
| WNT5B | Wnt Family Member 5B |
| WNK4 | WNK Lysine Deficient Protein Kinase 4 |
| SLCO1B3 | Solute Carrier Organic Anion Transporter Family Member 1B3 |
| CLCN1 | Chloride Voltage-Gated Channel 1 |
| NME7 | NME/NM23 Family Member 7 |
| TMLHE | Trimethyllysine Hydroxylase, Epsilon |
| HDAC10 | Histone Deacetylase 10 |
| DHRS3 | Dehydrogenase/Reductase 3 |
| ORC1 | Origin Recognition Complex Subunit 1 |
| CCS | Copper Chaperone For Superoxide Dismutase |
| MASTL | Microtubule Associated Serine/Threonine Kinase Like |
| MCFD2 | Multiple Coagulation Factor Deficiency 2, ER Cargo Receptor Complex Subunit |
| TNNT1 | Troponin T1, Slow Skeletal Type |
| NDUFB3 | NADH:Ubiquinone Oxidoreductase Subunit B3 |
| TCF7L1 | Transcription Factor 7 Like 1 |
| STEAP2 | STEAP2 Metalloreductase |
| CREB3L1 | CAMP Responsive Element Binding Protein 3 Like 1 |
| COQ7 | Coenzyme Q7, Hydroxylase |
| DNAL4 | Dynein Axonemal Light Chain 4 |
| DTYMK | Deoxythymidylate Kinase |
| HOXA11 | Homeobox A11 |
| METTL1 | Methyltransferase Like 1 |
| ADI1 | Acireductone Dioxygenase 1 |
| AFF2 | AF4/FMR2 Family Member 2 |
| ATXN10 | Ataxin 10 |
| FMO5 | Flavin Containing Dimethylaniline Monoxygenase 5 |
| USP16 | Ubiquitin Specific Peptidase 16 |
| YME1L1 | YME1 Like 1 ATPase |
| SLC39A13 | Solute Carrier Family 39 Member 13 |
| SRM | Spermidine Synthase |
| SULT4A1 | Sulfotransferase Family 4A Member 1 |
| BCAM | Basal Cell Adhesion Molecule (Lutheran Blood Group) |
| EPN2 | Epsin 2 |
| WNT9A | Wnt Family Member 9A |
| UGT2B15 | UDP Glucuronosyltransferase Family 2 Member B15 |
| HKDC1 | Hexokinase Domain Containing 1 |
| NBL1 | NBL1, DAN Family BMP Antagonist |
| NDUFAF2 | NADH:Ubiquinone Oxidoreductase Complex Assembly Factor 2 |
| PREPL | Prolyl Endopeptidase Like |
| IFT88 | Intraflagellar Transport 88 |
| PEMT | Phosphatidylethanolamine N-Methyltransferase |
| PMM1 | Phosphomannomutase 1 |
| KATNB1 | Katanin Regulatory Subunit B1 |
| TBCK | TBC1 Domain Containing Kinase |
| KLK10 | Kallikrein Related Peptidase 10 |
| PRSS12 | Serine Protease 12 |
| GTPBP3 | GTP Binding Protein 3, Mitochondrial |
| CYP8B1 | Cytochrome P450 Family 8 Subfamily B Member 1 |
| RFX2 | Regulatory Factor X2 |
| DDX4 | DEAD-Box Helicase 4 |
| CEP63 | Centrosomal Protein 63 |
| GALNT11 | Polypeptide N-Acetylgalactosaminyltransferase 11 |
| ME3 | Malic Enzyme 3 |
| ACP6 | Acid Phosphatase 6, Lysophosphatidic |
| FKBP14 | FKBP Prolyl Isomerase 14 |
| UNC13A | Unc-13 Homolog A |
| TULP3 | TUB Like Protein 3 |
| SLC38A4 | Solute Carrier Family 38 Member 4 |
| PHF6 | PHD Finger Protein 6 |
| SLC47A1 | Solute Carrier Family 47 Member 1 |
| EN2 | Engrailed Homeobox 2 |
| NT5C1A | 5'-Nucleotidase, Cytosolic IA |
| SLC16A9 | Solute Carrier Family 16 Member 9 |
| RECQL5 | RecQ Like Helicase 5 |
| SLC43A1 | Solute Carrier Family 43 Member 1 |
| IFT81 | Intraflagellar Transport 81 |
| PRDM6 | PR/SET Domain 6 |
| POFUT2 | Protein O-Fucosyltransferase 2 |
| SLC7A3 | Solute Carrier Family 7 Member 3 |
| TEX14 | Testis Expressed 14, Intercellular Bridge Forming Factor |
| IFT57 | Intraflagellar Transport 57 |
| NIF3L1 | NGG1 Interacting Factor 3 Like 1 |
| HS3ST3B1 | Heparan Sulfate-Glucosamine 3-Sulfotransferase 3B1 |
| SDSL | Serine Dehydratase Like |
| NKX6-1 | NK6 Homeobox 1 |
| OPA3 | Outer Mitochondrial Membrane Lipid Metabolism Regulator OPA3 |
| RRAD | RRAD, Ras Related Glycolysis Inhibitor And Calcium Channel Regulator |
| MAPRE3 | Microtubule Associated Protein RP/EB Family Member 3 |
| FAM20A | FAM20A Golgi Associated Secretory Pathway Pseudokinase |
| ACOT2 | Acyl-CoA Thioesterase 2 |
| ACY3 | Aminoacylase 3 |
| TUBGCP6 | Tubulin Gamma Complex Associated Protein 6 |
| UFSP2 | UFM1 Specific Peptidase 2 |
| UQCR10 | Ubiquinol-Cytochrome C Reductase, Complex III Subunit X |
| TSEN54 | TRNA Splicing Endonuclease Subunit 54 |
| PGLS | 6-Phosphogluconolactonase |
| LLGL1 | LLGL Scribble Cell Polarity Complex Component 1 |
| SLC25A16 | Solute Carrier Family 25 Member 16 |
| SLC6A18 | Solute Carrier Family 6 Member 18 |
| POLD4 | DNA Polymerase Delta 4, Accessory Subunit |
| IRX5 | Iroquois Homeobox 5 |
| TBC1D20 | TBC1 Domain Family Member 20 |
| KIF17 | Kinesin Family Member 17 |
| TMOD4 | Tropomodulin 4 |
| TMPRSS5 | Transmembrane Serine Protease 5 |
| TMEM126B | Transmembrane Protein 126B |
| KLHL12 | Kelch Like Family Member 12 |
| GSPT2 | G1 To S Phase Transition 2 |
| MMP23B | Matrix Metallopeptidase 23B |
| EAF2 | ELL Associated Factor 2 |
| NAV2 | Neuron Navigator 2 |
| HCN3 | Hyperpolarization Activated Cyclic Nucleotide Gated Potassium Channel 3 |
| COQ5 | Coenzyme Q5, Methyltransferase |
| HOXC6 | Homeobox C6 |
| HPCAL4 | Hippocalcin Like 4 |
| GPR85 | G Protein-Coupled Receptor 85 |
| DNALI1 | Dynein Axonemal Light Intermediate Chain 1 |
| DPY19L2 | Dpy-19 Like 2 |
| MFF | Mitochondrial Fission Factor |
| CCP110 | Centriolar Coiled-Coil Protein 110 |
| AGR3 | Anterior Gradient 3, Protein Disulphide Isomerase Family Member |
| ANKK1 | Ankyrin Repeat And Kinase Domain Containing 1 |
| ARHGEF16 | Rho Guanine Nucleotide Exchange Factor 16 |
| AATK | Apoptosis Associated Tyrosine Kinase |
| ACCS | 1-Aminocyclopropane-1-Carboxylate Synthase Homolog (Inactive) |
| ERC2 | ELKS/RAB6-Interacting/CAST Family Member 2 |
| KRT83 | Keratin 83 |
| TNS4 | Tensin 4 |
| UFC1 | Ubiquitin-Fold Modifier Conjugating Enzyme 1 |
| PIGP | Phosphatidylinositol Glycan Anchor Biosynthesis Class P |
| SLC39A12 | Solute Carrier Family 39 Member 12 |
| PHOSPHO1 | Phosphoethanolamine/Phosphocholine Phosphatase 1 |
| SEC31B | SEC31 Homolog B, COPII Coat Complex Component |
| TBCA | Tubulin Folding Cofactor A |
| SYCE1 | Synaptonemal Complex Central Element Protein 1 |
| KLHL40 | Kelch Like Family Member 40 |
| PRDM9 | PR/SET Domain 9 |
| NHLH2 | Nescient Helix-Loop-Helix 2 |
| CREB3L4 | CAMP Responsive Element Binding Protein 3 Like 4 |
| RMI1 | RecQ Mediated Genome Instability 1 |
| CDK5RAP1 | CDK5 Regulatory Subunit Associated Protein 1 |
| ALKBH2 | AlkB Homolog 2, Alpha-Ketoglutarate Dependent Dioxygenase |
| ADAMTS14 | ADAM Metallopeptidase With Thrombospondin Type 1 Motif 14 |
| FGGY | FGGY Carbohydrate Kinase Domain Containing |
| FEZF1 | FEZ Family Zinc Finger 1 |
| RDH16 | Retinol Dehydrogenase 16 |
| ZIC4 | Zic Family Member 4 |
| LEO1 | LEO1 Homolog, Paf1/RNA Polymerase II Complex Component |
| ZNF354A | Zinc Finger Protein 354A |
| ZNF592 | Zinc Finger Protein 592 |
| CNTROB | Centrobin, Centriole Duplication And Spindle Assembly Protein |
| NOSTRIN | Nitric Oxide Synthase Trafficking |
| REPS2 | RALBP1 Associated Eps Domain Containing 2 |
| RBMS2 | RNA Binding Motif Single Stranded Interacting Protein 2 |
| IFT74 | Intraflagellar Transport 74 |
| PIH1D1 | PIH1 Domain Containing 1 |
| PKD1L1 | Polycystin 1 Like 1, Transient Receptor Potential Channel Interacting |
| HTR3E | 5-Hydroxytryptamine Receptor 3E |
| SPAG16 | Sperm Associated Antigen 16 |
| RASIP1 | Ras Interacting Protein 1 |
| TEKT1 | Tektin 1 |
| TEKT2 | Tektin 2 |
| PAXIP1 | PAX Interacting Protein 1 |
| P3H2 | Prolyl 3-Hydroxylase 2 |
| SUGCT | Succinyl-CoA:Glutarate-CoA Transferase |
| TAF1C | TATA-Box Binding Protein Associated Factor, RNA Polymerase I Subunit C |
| IFT52 | Intraflagellar Transport 52 |
| DAND5 | DAN Domain BMP Antagonist Family Member 5 |
| CRYZL1 | Crystallin Zeta Like 1 |
| DCAF17 | DDB1 And CUL4 Associated Factor 17 |
| DNAH2 | Dynein Axonemal Heavy Chain 2 |
| NT5M | 5',3'-Nucleotidase, Mitochondrial |
| ROPN1L | Rhophilin Associated Tail Protein 1 Like |
| GABRR3 | Gamma-Aminobutyric Acid Type A Receptor Subunit Rho3 (Gene/Pseudogene) |
| FTMT | Ferritin Mitochondrial |
| CCDC8 | Coiled-Coil Domain Containing 8 |
| C19orf12 | Chromosome 19 Open Reading Frame 12 |
| LYPD1 | LY6/PLAUR Domain Containing 1 |
| ACER2 | Alkaline Ceramidase 2 |
| ARV1 | ARV1 Homolog, Fatty Acid Homeostasis Modulator |
| CCDC141 | Coiled-Coil Domain Containing 141 |
| TRAPPC12 | Trafficking Protein Particle Complex 12 |
| ZBTB38 | Zinc Finger And BTB Domain Containing 38 |
| TTC14 | Tetratricopeptide Repeat Domain 14 |
| SLC25A40 | Solute Carrier Family 25 Member 40 |
| SRCIN1 | SRC Kinase Signaling Inhibitor 1 |
| SETD1B | SET Domain Containing 1B, Histone Lysine Methyltransferase |
| SPATA16 | Spermatogenesis Associated 16 |
| TEKT4 | Tektin 4 |
| TEKT5 | Tektin 5 |
| ISLR2 | Immunoglobulin Superfamily Containing Leucine Rich Repeat 2 |
| ITIH5 | Inter-Alpha-Trypsin Inhibitor Heavy Chain 5 |
| PATL1 | PAT1 Homolog 1, Processing Body MRNA Decay Factor |
| SYCP1 | Synaptonemal Complex Protein 1 |
| INSL6 | Insulin Like 6 |
| HES5 | Hes Family BHLH Transcription Factor 5 |
| NAALADL2 | N-Acetylated Alpha-Linked Acidic Dipeptidase Like 2 |
| HBQ1 | Hemoglobin Subunit Theta 1 |
| GPR22 | G Protein-Coupled Receptor 22 |
| CEP89 | Centrosomal Protein 89 |
| GLYATL1 | Glycine-N-Acyltransferase Like 1 |
| FOXR1 | Forkhead Box R1 |
| DUSP26 | Dual Specificity Phosphatase 26 |
| CEND1 | Cell Cycle Exit And Neuronal Differentiation 1 |
| AKAP3 | A-Kinase Anchoring Protein 3 |
| ACTRT2 | Actin Related Protein T2 |
| LRTM1 | Leucine Rich Repeats And Transmembrane Domains 1 |
| MAP1S | Microtubule Associated Protein 1S |
| CACHD1 | Cache Domain Containing 1 |
| ARMC10 | Armadillo Repeat Containing 10 |
| MED9 | Mediator Complex Subunit 9 |
| ZFP90 | ZFP90 Zinc Finger Protein |
| ZMYM3 | Zinc Finger MYM-Type Containing 3 |
| TTLL3 | Tubulin Tyrosine Ligase Like 3 |
| TTLL6 | Tubulin Tyrosine Ligase Like 6 |
| SLC35E3 | Solute Carrier Family 35 Member E3 |
| ZNF563 | Zinc Finger Protein 563 |
| CDH24 | Cadherin 24 |
| HENMT1 | HEN Methyltransferase 1 |
| EFR3B | EFR3 Homolog B |
| NRK | Nik Related Kinase |
| PLGRKT | Plasminogen Receptor With A C-Terminal Lysine |
| TEKT3 | Tektin 3 |
| TCHP | Trichoplein Keratin Filament Binding |
| PCIF1 | PDX1 C-Terminal Inhibiting Factor 1 |
| IQCA1 | IQ Motif Containing With AAA Domain 1 |
| TANC1 | Tetratricopeptide Repeat, Ankyrin Repeat And Coiled-Coil Containing 1 |
| TANGO2 | Transport And Golgi Organization 2 Homolog |
| TEX12 | Testis Expressed 12 |
| TMEM41A | Transmembrane Protein 41A |
| MAP9 | Microtubule Associated Protein 9 |
| FNDC5 | Fibronectin Type III Domain Containing 5 |
| RFX7 | Regulatory Factor X7 |
| SCLT1 | Sodium Channel And Clathrin Linker 1 |
| MIEN1 | Migration And Invasion Enhancer 1 |
| GOLIM4 | Golgi Integral Membrane Protein 4 |
| GMCL1 | Germ Cell-Less 1, Spermatogenesis Associated |
| DPH7 | Diphthamide Biosynthesis 7 |
| RNF112 | Ring Finger Protein 112 |
| MFSD6 | Major Facilitator Superfamily Domain Containing 6 |
| MDM1 | Mdm1 Nuclear Protein |
| MCF2L2 | MCF.2 Cell Line Derived Transforming Sequence-Like 2 |
| AJAP1 | Adherens Junctions Associated Protein 1 |
| C21orf91 | Chromosome 21 Open Reading Frame 91 |
| MAATS1 | MYCBP Associated And Testis Expressed 1 |
| ALX3 | ALX Homeobox 3 |
| ARSJ | Arylsulfatase Family Member J |
| USP50 | Ubiquitin Specific Peptidase 50 |
| ZNF17 | Zinc Finger Protein 17 |
| ZBTB4 | Zinc Finger And BTB Domain Containing 4 |
| TTLL9 | Tubulin Tyrosine Ligase Like 9 |
| TSC22D2 | TSC22 Domain Family Member 2 |
| ZNF771 | Zinc Finger Protein 771 |
| ZNF496 | Zinc Finger Protein 496 |
| INIP | INTS3 And NABP Interacting Protein |
| SCRN3 | Secernin 3 |
| RPAP3 | RNA Polymerase II Associated Protein 3 |
| FUOM | Fucose Mutarotase |
| CCDC63 | Coiled-Coil Domain Containing 63 |
| AMN1 | Antagonist Of Mitotic Exit Network 1 Homolog |
| FBF1 | Fas Binding Factor 1 |
| UNC80 | Unc-80 Homolog, NALCN Channel Complex Subunit |
| PCDHGA5 | Protocadherin Gamma Subfamily A, 5 |
| ZNF721 | Zinc Finger Protein 721 |
| SPEF1 | Sperm Flagellar 1 |
| SPEM1 | Spermatid Maturation 1 |
| PWWP2B | PWWP Domain Containing 2B |
| TAMM41 | TAM41 Mitochondrial Translocator Assembly And Maintenance Homolog |
| IGSF5 | Immunoglobulin Superfamily Member 5 |
| IGLON5 | IgLON Family Member 5 |
| CYP27C1 | Cytochrome P450 Family 27 Subfamily C Member 1 |
| RIBC2 | RIB43A Domain With Coiled-Coils 2 |
| OR5H6 | Olfactory Receptor Family 5 Subfamily H Member 6 (Gene/Pseudogene) |
| CFAP52 | Cilia And Flagella Associated Protein 52 |
| DNHD1 | Dynein Heavy Chain Domain 1 |
| MMGT1 | Membrane Magnesium Transporter 1 |
| BEND7 | BEN Domain Containing 7 |
| ARHGAP23 | Rho GTPase Activating Protein 23 |
| C5orf15 | Chromosome 5 Open Reading Frame 15 |
| AP5M1 | Adaptor Related Protein Complex 5 Subunit Mu 1 |
| CALML6 | Calmodulin Like 6 |
| RDM1 | RAD52 Motif Containing 1 |
| TMEM186 | Transmembrane Protein 186 |
| TM2D1 | TM2 Domain Containing 1 |
| ZCWPW1 | Zinc Finger CW-Type And PWWP Domain Containing 1 |
| ZNF141 | Zinc Finger Protein 141 |
| PCDHB1 | Protocadherin Beta 1 |
| CAMTA2 | Calmodulin Binding Transcription Activator 2 |
| IFT22 | Intraflagellar Transport 22 |
| TAAR9 | Trace Amine Associated Receptor 9 (Gene/Pseudogene) |
| IQCD | IQ Motif Containing D |
| TMEM59L | Transmembrane Protein 59 Like |
| MRM2 | Mitochondrial RRNA Methyltransferase 2 |
| DPPA3 | Developmental Pluripotency Associated 3 |
| RPL39L | Ribosomal Protein L39 Like |
| FAM193A | Family With Sequence Similarity 193 Member A |
| ARL13A | ADP Ribosylation Factor Like GTPase 13A |
| LDLRAD2 | Low Density Lipoprotein Receptor Class A Domain Containing 2 |
| SLC45A1 | Solute Carrier Family 45 Member 1 |
| ZNF672 | Zinc Finger Protein 672 |
| ZNF772 | Zinc Finger Protein 772 |
| MAP10 | Microtubule Associated Protein 10 |
| DAW1 | Dynein Assembly Factor With WD Repeats 1 |
| NBPF15 | NBPF Member 15 |
| DLEU7 | Deleted In Lymphocytic Leukemia 7 |
| CFAP43 | Cilia And Flagella Associated Protein 43 |
| GPR148 | G Protein-Coupled Receptor 148 |
| MTURN | Maturin, Neural Progenitor Differentiation Regulator Homolog |
| RSPH6A | Radial Spoke Head 6 Homolog A |
| ESX1 | ESX Homeobox 1 |
| PGBD2 | PiggyBac Transposable Element Derived 2 |
| ZNF69 | Zinc Finger Protein 69 |
| ZNF718 | Zinc Finger Protein 718 |
| ZSWIM2 | Zinc Finger SWIM-Type Containing 2 |
| TBC1D3 | TBC1 Domain Family Member 3 |
| TMSB15A | Thymosin Beta 15a |
| DRC7 | Dynein Regulatory Complex Subunit 7 |
| ANKRD20A1 | Ankyrin Repeat Domain 20 Family Member A1 |
| DSCR4 | Down Syndrome Critical Region 4 |
| SPDYE4 | Speedy/RINGO Cell Cycle Regulator Family Member E4 |
| CLTRN | Collectrin, Amino Acid Transport Regulator |
| NBPF6 | NBPF Member 6 |
| MSMP | Microseminoprotein, Prostate Associated |
| CFAP61 | Cilia And Flagella Associated Protein 61 |
| FAM153B | Family With Sequence Similarity 153 Member B |
| C4orf48 | Chromosome 4 Open Reading Frame 48 |
| CCDC144A | Coiled-Coil Domain Containing 144A |
| PIFO | Primary Cilia Formation |
| SMIM1 | Small Integral Membrane Protein 1 (Vel Blood Group) |
| PRB4 | Proline Rich Protein BstNI Subfamily 4 |
| TMEM52 | Transmembrane Protein 52 |
| NBPF10 | NBPF Member 10 |
| NBPF11 | NBPF Member 11 |
| NBPF12 | NBPF Member 12 |
| NBPF4 | NBPF Member 4 |
| MTARC1 | Mitochondrial Amidoxime Reducing Component 1 |
| FOXI3 | Forkhead Box I3 |
| FAM170B | Family With Sequence Similarity 170 Member B |
| AADACL3 | Arylacetamide Deacetylase Like 3 |
| KRTAP4-5 | Keratin Associated Protein 4-5 |
| TMEM238 | Transmembrane Protein 238 |
| DSCR8 | Down Syndrome Critical Region 8 |
| DSCR9 | Down Syndrome Critical Region 9 |
| CLLU1 | Chronic Lymphocytic Leukemia Up-Regulated 1 |
| GMNC | Geminin Coiled-Coil Domain Containing |
| NKX1-1 | NK1 Homeobox 1 |
| ATXN8OS | ATXN8 Opposite Strand LncRNA |
| C22orf42 | Chromosome 22 Open Reading Frame 42 |
| WT1-AS | WT1 Antisense RNA |
| OST4 | Oligosaccharyltransferase Complex Subunit 4, Non-Catalytic |
| PRAC1 | PRAC1 Small Nuclear Protein |
| NBPF9 | NBPF Member 9 |
| GON7 | GON7 Subunit Of KEOPS Complex |
| CASC2 | Cancer Susceptibility 2 |
| MYCNOS | MYCN Opposite Strand |
| NBPF7 | NBPF Member 7 |
| SPEM2 | SPEM Family Member 2 |
| PCOTH | Pro-X-Gly Collagen Triple Helix Like Repeat Containing |
| NBPF20 | NBPF Member 20 |
| NBPF8 | NBPF Member 8 |
| HOTTIP | HOXA Distal Transcript Antisense RNA |
| FRG2C | FSHD Region Gene 2 Family Member C |
| FAM187A | Family With Sequence Similarity 187 Member A |
| C17orf107 | Chromosome 17 Open Reading Frame 107 |
| BCLAF3 | BCLAF1 And THRAP3 Family Member 3 |
| SMKR1 | Small Lysine Rich Protein 1 |
| LINC01587 | Long Intergenic Non-Protein Coding RNA 1587 |
| PCAT4 | Prostate Cancer Associated Transcript 4 |
| PTENP1 | Phosphatase And Tensin Homolog Pseudogene 1 |
| CFAP161 | Cilia And Flagella Associated Protein 161 |
| DSCR10 | Down Syndrome Critical Region 10 |
| PRAC2 | PRAC2 Small Nuclear Protein |
| EGOT | Eosinophil Granule Ontogeny Transcript |
| LINC00467 | Long Intergenic Non-Protein Coding RNA 467 |
| SNHG8 | Small Nucleolar RNA Host Gene 8 |
| PMS2P2 | PMS1 Homolog 2, Mismatch Repair System Component Pseudogene 2 |
| MIR190A | MicroRNA 190a |
| HMGN2P46 | High Mobility Group Nucleosomal Binding Domain 2 Pseudogene 46 |
| DBH-AS1 | DBH Antisense RNA 1 |
| BDNF-AS | BDNF Antisense RNA |
| CASC15 | Cancer Susceptibility 15 |
| SMCR5 | Smith-Magenis Syndrome Chromosome Region, Candidate 5 |
| MIR16-2 | MicroRNA 16-2 |
| LRRC74B | Leucine Rich Repeat Containing 74B |
| HLA-F-AS1 | HLA-F Antisense RNA 1 |
| SNHG16 | Small Nucleolar RNA Host Gene 16 |
| MIR202 | MicroRNA 202 |
| MIR519D | MicroRNA 519d |
| MIR497 | MicroRNA 497 |
| HCG11 | HLA Complex Group 11 |
| MIR100HG | Mir-100-Let-7a-2-Mir-125b-1 Cluster Host Gene |
| MIR371A | MicroRNA 371a |
| SNORA59A | Small Nucleolar RNA, H/ACA Box 59A |
| TBC1D3D | TBC1 Domain Family Member 3D |
| MIR802 | MicroRNA 802 |
| MIR92A2 | MicroRNA 92a-2 |
| MIR95 | MicroRNA 95 |
| HAGLR | HOXD Antisense Growth-Associated Long Non-Coding RNA |
| FMR1-AS1 | FMR1 Antisense RNA 1 |
| LINC00599 | Long Intergenic Non-Protein Coding RNA 599 |
| LINC01098 | Long Intergenic Non-Protein Coding RNA 1098 |
| MIR498 | MicroRNA 498 |
| RNU11 | RNA, U11 Small Nuclear |
| SNORD35A | Small Nucleolar RNA, C/D Box 35A |
| SOCS2-AS1 | SOCS2 Antisense RNA 1 |
| PCAT2 | Prostate Cancer Associated Transcript 2 |
| PCAT6 | Prostate Cancer Associated Transcript 6 |
| PAUPAR | PAX6 Upstream Antisense RNA |
| GUSBP4 | GUSB Pseudogene 4 |
| NBPF5P | NBPF Member 5, Pseudogene |
| NBAT1 | Neuroblastoma Associated Transcript 1 |
| RNY1 | RNA, Ro60-Associated Y1 |
| SACS-AS1 | SACS Antisense RNA 1 |
| GATA6-AS1 | GATA6 Antisense RNA 1 (Head To Head) |
| FOXD2-AS1 | FOXD2 Adjacent Opposite Strand RNA 1 |
| FALEC | Focally Amplified Long Non-Coding RNA In Epithelial Cancer |
| TTTY7 | Testis-Specific Transcript, Y-Linked 7 |
| LINC01013 | Long Intergenic Non-Protein Coding RNA 1013 |
| ZNF667-AS1 | ZNF667 Antisense RNA 1 (Head To Head) |
| PCAT19 | Prostate Cancer Associated Transcript 19 |
| PCAT29 | Prostate Cancer Associated Transcript 29 |
| MAP3K14-AS1 | MAP3K14 Antisense RNA 1 |
| NHEG1 | Neuroblastoma Highly Expressed 1 |
| MIR4707 | MicroRNA 4707 |
| RNY3 | RNA, Ro60-Associated Y3 |
| CD81-AS1 | CD81 Antisense RNA 1 |
| FGD5-AS1 | FGD5 Antisense RNA 1 |
| LINC00536 | Long Intergenic Non-Protein Coding RNA 536 |
| LINC01405 | Long Intergenic Non-Protein Coding RNA 1405 |
| LINC01138 | Long Intergenic Non-Protein Coding RNA 1138 |
| LINC01191 | Long Intergenic Non-Protein Coding RNA 1191 |
| KCNK15-AS1 | KCNK15 And WISP2 Antisense RNA 1 |
| TMPO-AS1 | TMPO Antisense RNA 1 |
| MIR1537 | MicroRNA 1537 |
| HLX-AS1 | HLX Antisense RNA 1 |
| MIR676 | MicroRNA 676 |
| DRAIC | Downregulated RNA In Cancer, Inhibitor Of Cell Invasion And Migration |
| MYCNUT | MYCN Upstream Transcript |
| SCHLAP1 | SWI/SNF Complex Antagonist Associated With Prostate Cancer 1 |
| CSMD2-AS1 | CSMD2 Antisense RNA 1 |
| CCAT2 | Colon Cancer Associated Transcript 2 |
| BUB1B-PAK6 | BUB1B-PAK6 Readthrough |
| CASC6 | Cancer Susceptibility 6 |
| TRERNA1 | Translation Regulatory Long Non-Coding RNA 1 |
| TRPM2-AS | TRPM2 Antisense RNA |
| SMCR2 | Smith-Magenis Syndrome Chromosome Region, Candidate 2 |
| LINC00963 | Long Intergenic Non-Protein Coding RNA 963 |
| LINC01564 | Long Intergenic Non-Protein Coding RNA 1564 |
| LOC101927322 | Uncharacterized LOC101927322 |
| PACERR | PTGS2 Antisense NFKB1 Complex-Mediated Expression Regulator RNA |
| PCAT5 | Prostate Cancer Associated Transcript 5 |
| MIR3945HG | MIR3945 Host Gene |
| MME-AS1 | MME Antisense RNA 1 |
| GATA2-AS1 | GATA2 Antisense RNA 1 |
| FAM225A | Family With Sequence Similarity 225 Member A |
| CASC23 | Cancer Susceptibility 23 |
| LINC00708 | Long Intergenic Non-Protein Coding RNA 708 |
| TGFB2-AS1 | TGFB2 Antisense RNA 1 (Head To Head) |
| WARS2-IT1 | WARS2 Intronic Transcript 1 |
| PCAT14 | Prostate Cancer Associated Transcript 14 |
| PCAT7 | Prostate Cancer Associated Transcript 7 |
| MHRT | Myosin Heavy Chain Associated RNA Transcript |
| LOC283177 | Uncharacterized LOC283177 |
| ETS1-AS1 | ETS1 Antisense RNA 1 |
| LUNAR1 | Leukemia-Associated Non-Coding IGF1R Activator RNA 1 |
| NBPF18P | NBPF Member 18, Pseudogene |
| NBPF2P | NBPF Member 2, Pseudogene |
| TGFB2-OT1 | TGFB2 Overlapping Transcript 1 |
| MIR3668 | MicroRNA 3668 |
| SPRY4-IT1 | SPRY4 Intronic Transcript 1 |
| NBPF13P | NBPF Member 13, Pseudogene |
| NBPF17P | NBPF Member 17, Pseudogene |
| RNU12-2P | RNA, U12 Small Nuclear 2, Pseudogene |
| DNAAF4-CCPG1 | DNAAF4-CCPG1 Readthrough (NMD Candidate) |
| CFAP298-TCP10L | CFAP298-TCP10L Readthrough |
| TRL-AAG2-3 | TRNA-Leu (Anticodon AAG) 2-3 |
| DUXAP9 | Double Homeobox A Pseudogene 9 |
| GUSBP15 | GUSB Pseudogene 15 |
| NBPF21P | NBPF Member 21, Pseudogene |
| LOC101448202 | Uncharacterized LOC101448202 |
| XLOC_008559 | Uncharacterized LOC105378427 |
| WHSC1L2P | Wolf-Hirschhorn Syndrome Candidate 1-Like 2, Pseudogene |
| ENSG00000259342 |  |
| PCSEAT | Prostate Cancer Expressed EZH2 Associated Transcript |
| SRP68P1 | Signal Recognition Particle 68 Pseudogene 1 |
| ENSG00000196893 |  |
| ENSG00000261777 |  |
| DNAH8-AS1 | DNAH8 Antisense RNA 1 |
| DALIR | DNMT1-Associated Long Intergenic Non-Coding RNA |
| ENSG00000286027 |  |
| ENSG00000259039 |  |
| H19-ICR | H19/IGF2 Imprinting Control Region |
| SNORD144 | Small Nucleolar RNA, C/D Box 144 |
| LOC105372310 | Uncharacterized LOC105372310 |
| POTEF-AS1 | POTEF Antisense RNA 1 |
| CNC2 | Carney Complex Type 2, Multiple Neoplasia And Lentiginosis |
| ENSG00000202198 |  |
| ENSG00000277524 |  |
| SMCR6 | Smith-Magenis Syndrome Chromosome Region, Candidate 6 |
| IDDM11 | Insulin Dependent Diabetes Mellitus 11 |
| IDDM3 | Insulin Dependent Diabetes Mellitus 3 |
| IDDM4 | Insulin Dependent Diabetes Mellitus 4 |
| IDDM8 | Insulin Dependent Diabetes Mellitus 8 |
| PRD | Primary Retinal Dysplasia |
| SNORD134 | Small Nucleolar RNA, C/D Box 134 |
| SNORD135 | Small Nucleolar RNA, C/D Box 135 |
| IDDM13 | Insulin Dependent Diabetes Mellitus 13 |
| IDDM7 | Insulin Dependent Diabetes Mellitus 7 |
| ENSG00000255174 |  |
| LOC100421446 | Nuclear Receptor Binding SET Domain Protein 3 Pseudogene |
| ENSG00000224410 |  |
| XLOC_007697 | Uncharacterized LOC105500239 |
| XLOC_009911 | Uncharacterized LOC105500240 |
| GRDX | Graves Disease, Susceptibility To, X-Linked |
| CMS1A1 | Myasthenia Gravis, Familial Infantile, 1 |
| GCCD2 | Glucocorticoid Deficiency 2 |
| TNDM | Diabetes Mellitus, Transient Neonatal |
| HBHR | Alpha-Thalassemia/Mental Retardation Syndrome, Type 1 |
| LOC107988024 | IKBKGP1 Recombination Region |
| LOC107988025 | IKBKGP1 Upstream Recombination Region |
| LOC114803478 | TRAF3IP2 EExon Liver Enhancer |
| LOC113939944 | Sharpr-MPRA Regulatory Region 9539 |
| LOC108745275 | Distal SMS-REP Block A Recombination Region |
| LOC108745276 | Proximal SMS-REP Block A Recombination Region |
| LOC109461476 | GDF5 Promoter Region |
| LOC110006319 | Beta-Globin Gene 3' Regulatory Region |
| LOC110011216 | Paired Like Homeobox 2b Polyalanine Repeat Instability Region |
| LOC112533672 | Sharpr-MPRA Regulatory Region 1193 |
| LOC106020709 | Distal SMS-REP Block C Recombination Region |
| LOC106020710 | Proximal SMS-REP Block C Recombination Region |
| LOC106020711 | LCR17pA Recombination Region |
| LOC106020712 | LCR17pD Recombination Region |
| LOC106099062 | HBB Recombination Region |
| LOC106694316 | Enhancer Region In Introns 7-9 Of MPO |
| LOC106804612 | Hemoglobin Subunit Alpha 2 Recombination Region |
| LOC106804613 | Hemoglobin Subunit Alpha 1 Recombination Region |
| LOC107133509 | NUP98-NSD3 Recombination Region |
| LOC107133510 | Origin Of Replication At HBB |
| LOC107372315 | OSGEP/APEX1 Bi-Directional Promoter Region |
| LOC107457585 | Meiotic Recombination Hotspot J |
| PCSK2 | Proprotein Convertase Subtilisin/Kexin Type 2 |
| RPS16 | Ribosomal Protein S16 |
| RPS18 | Ribosomal Protein S18 |
| SF3A1 | Splicing Factor 3a Subunit 1 |
| DEFT1P | Defensin Theta 1, Pseudogene |
| TREH | Trehalase |
| CLCN2 | Chloride Voltage-Gated Channel 2 |
| CLPP | Caseinolytic Mitochondrial Matrix Peptidase Proteolytic Subunit |
| UPF2 | UPF2 Regulator Of Nonsense Mediated MRNA Decay |
| NLE1 | Notchless Homolog 1 |
| TYMS | Thymidylate Synthetase |
| H4C1 | H4 Clustered Histone 1 |
| VCAN | Versican |
| CHRM1 | Cholinergic Receptor Muscarinic 1 |
| UIMC1 | Ubiquitin Interaction Motif Containing 1 |
| LRCH2 | Leucine Rich Repeats And Calponin Homology Domain Containing 2 |
| CDKN1C | Cyclin Dependent Kinase Inhibitor 1C |
| FABP6 | Fatty Acid Binding Protein 6 |
| KAT7 | Lysine Acetyltransferase 7 |
| TMEM43 | Transmembrane Protein 43 |
| EXOSC2 | Exosome Component 2 |
| PPP1R9B | Protein Phosphatase 1 Regulatory Subunit 9B |
| MID1IP1 | MID1 Interacting Protein 1 |
| SRSF11 | Serine And Arginine Rich Splicing Factor 11 |
| DDX46 | DEAD-Box Helicase 46 |
| LAS1L | LAS1 Like Ribosome Biogenesis Factor |
| HSPA4L | Heat Shock Protein Family A (Hsp70) Member 4 Like |
| SMU1 | SMU1 DNA Replication Regulator And Spliceosomal Factor |
| RRP1B | Ribosomal RNA Processing 1B |
| CCDC137 | Coiled-Coil Domain Containing 137 |
| URB1 | URB1 Ribosome Biogenesis Homolog |
| ITPRID2 | ITPR Interacting Domain Containing 2 |
| RPL27A | Ribosomal Protein L27a |
| CYC1 | Cytochrome C1 |
| PFKFB3 | 6-Phosphofructo-2-Kinase/Fructose-2,6-Biphosphatase 3 |
| SLC25A5 | Solute Carrier Family 25 Member 5 |
| RPLP1 | Ribosomal Protein Lateral Stalk Subunit P1 |
| ATP1B1 | ATPase Na+/K+ Transporting Subunit Beta 1 |
| TUBB6 | Tubulin Beta 6 Class V |
| THRAP3 | Thyroid Hormone Receptor Associated Protein 3 |
| SNRPF | Small Nuclear Ribonucleoprotein Polypeptide F |
| UCN3 | Urocortin 3 |
| PPM1A | Protein Phosphatase, Mg2+/Mn2+ Dependent 1A |
| ID3 | Inhibitor Of DNA Binding 3, HLH Protein |
| SLC12A1 | Solute Carrier Family 12 Member 1 |
| SCO1 | Synthesis Of Cytochrome C Oxidase 1 |
| SLC19A3 | Solute Carrier Family 19 Member 3 |
| CNGA3 | Cyclic Nucleotide Gated Channel Subunit Alpha 3 |
| SOX17 | SRY-Box Transcription Factor 17 |
| MYO7A | Myosin VIIA |
| PEX26 | Peroxisomal Biogenesis Factor 26 |
| LYL1 | LYL1 Basic Helix-Loop-Helix Family Member |
| FAN1 | FANCD2 And FANCI Associated Nuclease 1 |
| FAM161A | FAM161 Centrosomal Protein A |
| WHRN | Whirlin |
| PCARE | Photoreceptor Cilium Actin Regulator |
| USF1 | Upstream Transcription Factor 1 |
| SYNCRIP | Synaptotagmin Binding Cytoplasmic RNA Interacting Protein |
| CAPRIN1 | Cell Cycle Associated Protein 1 |
| SPAG8 | Sperm Associated Antigen 8 |
| EIF3J | Eukaryotic Translation Initiation Factor 3 Subunit J |
| RPP30 | Ribonuclease P/MRP Subunit P30 |
| RFLNB | Refilin B |
| BAG5 | BAG Cochaperone 5 |
| RBM17 | RNA Binding Motif Protein 17 |
| HCN2 | Hyperpolarization Activated Cyclic Nucleotide Gated Potassium And Sodium Channel 2 |
| STAM | Signal Transducing Adaptor Molecule |
| MPZL2 | Myelin Protein Zero Like 2 |
| SVEP1 | Sushi, Von Willebrand Factor Type A, EGF And Pentraxin Domain Containing 1 |
| SMAP1 | Small ArfGAP 1 |
| DDX52 | DExD-Box Helicase 52 |
| CNGB3 | Cyclic Nucleotide Gated Channel Subunit Beta 3 |
| NAGA | Alpha-N-Acetylgalactosaminidase |
| LTB4R2 | Leukotriene B4 Receptor 2 |
| KRT6A | Keratin 6A |
| SUSD4 | Sushi Domain Containing 4 |
| ABLIM1 | Actin Binding LIM Protein 1 |
| ABCF2 | ATP Binding Cassette Subfamily F Member 2 |
| HNRNPAB | Heterogeneous Nuclear Ribonucleoprotein A/B |
| ADGRE2 | Adhesion G Protein-Coupled Receptor E2 |
| FUBP3 | Far Upstream Element Binding Protein 3 |
| PRRC2C | Proline Rich Coiled-Coil 2C |
| SLC36A1 | Solute Carrier Family 36 Member 1 |
| LBH | LBH Regulator Of WNT Signaling Pathway |
| CCDC116 | Coiled-Coil Domain Containing 116 |
| IFITM3P5 | IFITM3 Pseudogene 5 |
| MYLK2 | Myosin Light Chain Kinase 2 |
| SLC16A2 | Solute Carrier Family 16 Member 2 |
| MYL4 | Myosin Light Chain 4 |
| SLCO2B1 | Solute Carrier Organic Anion Transporter Family Member 2B1 |
| PNPLA8 | Patatin Like Phospholipase Domain Containing 8 |
| ANGPTL1 | Angiopoietin Like 1 |
| CDKN2D | Cyclin Dependent Kinase Inhibitor 2D |
| MYH8 | Myosin Heavy Chain 8 |
| MYH15 | Myosin Heavy Chain 15 |
| MYH16 | Myosin Heavy Chain 16 Pseudogene |
| RGS6 | Regulator Of G Protein Signaling 6 |
| FOXC1 | Forkhead Box C1 |
| ACOT8 | Acyl-CoA Thioesterase 8 |
| EIF3G | Eukaryotic Translation Initiation Factor 3 Subunit G |
| DDX50 | DExD-Box Helicase 50 |
| MAT2A | Methionine Adenosyltransferase 2A |
| GMDS | GDP-Mannose 4,6-Dehydratase |
| TM9SF2 | Transmembrane 9 Superfamily Member 2 |
| RBM3 | RNA Binding Motif Protein 3 |
| ZNF384 | Zinc Finger Protein 384 |
| OLIG3 | Oligodendrocyte Transcription Factor 3 |
| GRWD1 | Glutamate Rich WD Repeat Containing 1 |
| RUVBL1 | RuvB Like AAA ATPase 1 |
| CDK8 | Cyclin Dependent Kinase 8 |
| RBL2 | RB Transcriptional Corepressor Like 2 |
| MTHFD1L | Methylenetetrahydrofolate Dehydrogenase (NADP+ Dependent) 1 Like |
| SND1 | Staphylococcal Nuclease And Tudor Domain Containing 1 |
| RPL23 | Ribosomal Protein L23 |
| EIF3C | Eukaryotic Translation Initiation Factor 3 Subunit C |
| ATAD3A | ATPase Family AAA Domain Containing 3A |
| AKAP8L | A-Kinase Anchoring Protein 8 Like |
| ATP1A2 | ATPase Na+/K+ Transporting Subunit Alpha 2 |
| PPIB | Peptidylprolyl Isomerase B |
| BDKRB1 | Bradykinin Receptor B1 |
| H2BC11 | H2B Clustered Histone 11 |
| H2BC6 | H2B Clustered Histone 6 |
| MMP16 | Matrix Metallopeptidase 16 |
| RPA3 | Replication Protein A3 |
| BRCC3 | BRCA1/BRCA2-Containing Complex Subunit 3 |
| CIAO3 | Cytosolic Iron-Sulfur Assembly Component 3 |
| PPP2R2A | Protein Phosphatase 2 Regulatory Subunit Balpha |
| EIF2S3 | Eukaryotic Translation Initiation Factor 2 Subunit Gamma |
| TNKS2 | Tankyrase 2 |
| EIF4B | Eukaryotic Translation Initiation Factor 4B |
| DNAJB6 | DnaJ Heat Shock Protein Family (Hsp40) Member B6 |
| ATP1B3 | ATPase Na+/K+ Transporting Subunit Beta 3 |
| CCT7 | Chaperonin Containing TCP1 Subunit 7 |
| CYGB | Cytoglobin |
| THOC1 | THO Complex 1 |
| EIF3B | Eukaryotic Translation Initiation Factor 3 Subunit B |
| RPL17 | Ribosomal Protein L17 |
| TMEM33 | Transmembrane Protein 33 |
| AHNAK2 | AHNAK Nucleoprotein 2 |
| LOC107980440 | ABL Breakpoint Recombination Region |
| ST3GAL6 | ST3 Beta-Galactoside Alpha-2,3-Sialyltransferase 6 |
| ZNF365 | Zinc Finger Protein 365 |
| FEM1A | Fem-1 Homolog A |
| SCYL2 | SCY1 Like Pseudokinase 2 |
| PRAM1 | PML-RARA Regulated Adaptor Molecule 1 |
| HELB | DNA Helicase B |
| GPRIN1 | G Protein Regulated Inducer Of Neurite Outgrowth 1 |
| AKNA | AT-Hook Transcription Factor |
| DDIT4 | DNA Damage Inducible Transcript 4 |
| CCKBR | Cholecystokinin B Receptor |
| RPL21 | Ribosomal Protein L21 |
| PTPRF | Protein Tyrosine Phosphatase Receptor Type F |
| PPP1R13B | Protein Phosphatase 1 Regulatory Subunit 13B |
| RNASE10 | Ribonuclease A Family Member 10 (Inactive) |
| CAD | Carbamoyl-Phosphate Synthetase 2, Aspartate Transcarbamylase, And Dihydroorotase |
| TUBB2A | Tubulin Beta 2A Class IIa |
| TMED10 | Transmembrane P24 Trafficking Protein 10 |
| EEF1B2 | Eukaryotic Translation Elongation Factor 1 Beta 2 |
| ANP32A | Acidic Nuclear Phosphoprotein 32 Family Member A |
| RPL34 | Ribosomal Protein L34 |
| COPE | COPI Coat Complex Subunit Epsilon |
| DNAJB11 | DnaJ Heat Shock Protein Family (Hsp40) Member B11 |
| RPL36 | Ribosomal Protein L36 |
| SRSF9 | Serine And Arginine Rich Splicing Factor 9 |
| EIF3D | Eukaryotic Translation Initiation Factor 3 Subunit D |
| EIF3I | Eukaryotic Translation Initiation Factor 3 Subunit I |
| SF3B3 | Splicing Factor 3b Subunit 3 |
| NPLOC4 | NPL4 Homolog, Ubiquitin Recognition Factor |
| MYO1B | Myosin IB |
| SIPA1L1 | Signal Induced Proliferation Associated 1 Like 1 |
| EIF3E | Eukaryotic Translation Initiation Factor 3 Subunit E |
| GPS1 | G Protein Pathway Suppressor 1 |
| METTL9 | Methyltransferase Like 9 |
| SLIRP | SRA Stem-Loop Interacting RNA Binding Protein |
| SH2D4B | SH2 Domain Containing 4B |
| CNOT9 | CCR4-NOT Transcription Complex Subunit 9 |
| H2BC3 | H2B Clustered Histone 3 |
| IGHV3-69-1 | Immunoglobulin Heavy Variable 3-69-1 (Pseudogene) |
| CLPX | Caseinolytic Mitochondrial Matrix Peptidase Chaperone Subunit X |
| RAD51B | RAD51 Paralog B |
| TBRG1 | Transforming Growth Factor Beta Regulator 1 |
| NLRP7 | NLR Family Pyrin Domain Containing 7 |
| SPECC1L | Sperm Antigen With Calponin Homology And Coiled-Coil Domains 1 Like |
| ENOPH1 | Enolase-Phosphatase 1 |
| WDR61 | WD Repeat Domain 61 |
| TPPP3 | Tubulin Polymerization Promoting Protein Family Member 3 |
| ARID5B | AT-Rich Interaction Domain 5B |
| SET | SET Nuclear Proto-Oncogene |
| HADHB | Hydroxyacyl-CoA Dehydrogenase Trifunctional Multienzyme Complex Subunit Beta |
| WEE1 | WEE1 G2 Checkpoint Kinase |
| HSD17B10 | Hydroxysteroid 17-Beta Dehydrogenase 10 |
| PTGES3 | Prostaglandin E Synthase 3 |
| PLOD3 | Procollagen-Lysine,2-Oxoglutarate 5-Dioxygenase 3 |
| EIF2B2 | Eukaryotic Translation Initiation Factor 2B Subunit Beta |
| EIF3F | Eukaryotic Translation Initiation Factor 3 Subunit F |
| CDX2 | Caudal Type Homeobox 2 |
| GSTA4 | Glutathione S-Transferase Alpha 4 |
| BAG2 | BAG Cochaperone 2 |
| RCN2 | Reticulocalbin 2 |
| RPL14 | Ribosomal Protein L14 |
| EIF3L | Eukaryotic Translation Initiation Factor 3 Subunit L |
| LUC7L3 | LUC7 Like 3 Pre-MRNA Splicing Factor |
| SNX19 | Sorting Nexin 19 |
| CLINT1 | Clathrin Interactor 1 |
| MRPL18 | Mitochondrial Ribosomal Protein L18 |
| AHRR | Aryl-Hydrocarbon Receptor Repressor |
| SH3BGRL2 | SH3 Domain Binding Glutamate Rich Protein Like 2 |
| GRAMD1B | GRAM Domain Containing 1B |
| ANP32E | Acidic Nuclear Phosphoprotein 32 Family Member E |
| FAM98B | Family With Sequence Similarity 98 Member B |
| LIPE | Lipase E, Hormone Sensitive Type |
| TOPORS | TOP1 Binding Arginine/Serine Rich Protein |
| KRT2 | Keratin 2 |
| PCDH15 | Protocadherin Related 15 |
| EGLN1 | Egl-9 Family Hypoxia Inducible Factor 1 |
| NPR3 | Natriuretic Peptide Receptor 3 |
| STATH | Statherin |
| MAGED4B | MAGE Family Member D4B |
| SPAG11B | Sperm Associated Antigen 11B |
| VPS28 | VPS28 Subunit Of ESCRT-I |
| C7orf50 | Chromosome 7 Open Reading Frame 50 |
| ZNF366 | Zinc Finger Protein 366 |
| NR6A1 | Nuclear Receptor Subfamily 6 Group A Member 1 |
| PDLIM5 | PDZ And LIM Domain 5 |
| H2BS1 | H2B.S Histone 1 |
| RNGTT | RNA Guanylyltransferase And 5'-Phosphatase |
| ZYX | Zyxin |
| SERPINA5 | Serpin Family A Member 5 |
| ATP2B1 | ATPase Plasma Membrane Ca2+ Transporting 1 |
| CLTB | Clathrin Light Chain B |
| VDAC3 | Voltage Dependent Anion Channel 3 |
| MRPL3 | Mitochondrial Ribosomal Protein L3 |
| CSE1L | Chromosome Segregation 1 Like |
| HNRNPH3 | Heterogeneous Nuclear Ribonucleoprotein H3 |
| RPL37A | Ribosomal Protein L37a |
| ACIN1 | Apoptotic Chromatin Condensation Inducer 1 |
| EIF5B | Eukaryotic Translation Initiation Factor 5B |
| LMAN2 | Lectin, Mannose Binding 2 |
| SCFD1 | Sec1 Family Domain Containing 1 |
| GEMIN5 | Gem Nuclear Organelle Associated Protein 5 |
| RANBP10 | RAN Binding Protein 10 |
| IPO8 | Importin 8 |
| TMED2 | Transmembrane P24 Trafficking Protein 2 |
| DIMT1 | DIMT1 RRNA Methyltransferase And Ribosome Maturation Factor |
| SAMD4A | Sterile Alpha Motif Domain Containing 4A |
| ARL8B | ADP Ribosylation Factor Like GTPase 8B |
| TTI2 | TELO2 Interacting Protein 2 |
| LRCH3 | Leucine Rich Repeats And Calponin Homology Domain Containing 3 |
| LY6K | Lymphocyte Antigen 6 Family Member K |
| TTC27 | Tetratricopeptide Repeat Domain 27 |
| BOLA2B | BolA Family Member 2B |
| H2AC12 | H2A Clustered Histone 12 |
| EEF1A1P5 | Eukaryotic Translation Elongation Factor 1 Alpha 1 Pseudogene 5 |
| CR1L | Complement C3b/C4b Receptor 1 Like |
| PYHIN1 | Pyrin And HIN Domain Family Member 1 |
| PKHD1L1 | PKHD1 Like 1 |
| BPIFB3 | BPI Fold Containing Family B Member 3 |
| IGKV1OR2-108 | Immunoglobulin Kappa Variable 1/OR2-108 (Non-Functional) |
| POGLUT1 | Protein O-Glucosyltransferase 1 |
| TOR4A | Torsin Family 4 Member A |
| ABCA3 | ATP Binding Cassette Subfamily A Member 3 |
| VEGFB | Vascular Endothelial Growth Factor B |
| MMP11 | Matrix Metallopeptidase 11 |
| DRD4 | Dopamine Receptor D4 |
| GLRX3 | Glutaredoxin 3 |
| EPHA4 | EPH Receptor A4 |
| NSFL1C | NSFL1 Cofactor |
| PHF19 | PHD Finger Protein 19 |
| C2CD2 | C2 Calcium Dependent Domain Containing 2 |
| ZBTB21 | Zinc Finger And BTB Domain Containing 21 |
| CCDC69 | Coiled-Coil Domain Containing 69 |
| PHETA1 | PH Domain Containing Endocytic Trafficking Adaptor 1 |
| PI4KAP2 | Phosphatidylinositol 4-Kinase Alpha Pseudogene 2 |
| LINC01122 | Long Intergenic Non-Protein Coding RNA 1122 |
| ADAM1A | ADAM Metallopeptidase Domain 1A (Pseudogene) |
| CUTALP | CutA Divalent Cation Tolerance Like, Pseudogene |
| LINC02352 | Long Intergenic Non-Protein Coding RNA 2352 |
| ENSG00000260302 |  |
| RNU6-1149P | RNA, U6 Small Nuclear 1149, Pseudogene |
| DUTP6 | Deoxyuridine Triphosphatase Pseudogene 6 |
| ENSG00000231128 |  |
| RNA5SP185 | RNA, 5S Ribosomal Pseudogene 185 |
| RPS10P14 | Ribosomal Protein S10 Pseudogene 14 |
| RNU6-565P | RNA, U6 Small Nuclear 565, Pseudogene |
| ENSG00000200113 |  |
| RNU6-959P | RNA, U6 Small Nuclear 959, Pseudogene |
| RNU6-1133P | RNA, U6 Small Nuclear 1133, Pseudogene |
| RN7SKP226 | RN7SK Pseudogene 226 |
| LOC105369519 | Uncharacterized LOC105369519 |
| RPL13AP23 | Ribosomal Protein L13a Pseudogene 23 |
| ENSG00000201566 |  |
| PSMC1P4 | Proteasome 26S Subunit, ATPase 1 Pseudogene 4 |
| lnc-RCSD1-5 |  |
| lnc-ANXA6-3 |  |
| lnc-IRGM-2 |  |
| lnc-YDJC-1 |  |
| piR-43107-023 |  |
| RF00026-601 |  |
| lnc-PSMD5-5 |  |
| RF00017-5007 |  |
| lnc-SFRP4-2 |  |
| lnc-CD247-1 |  |
| RF00017-1272 |  |
| RF00026-664 |  |
| piR-43105-423 |  |
| ENSG00000231712 |  |
| HSALNG0013961 |  |
| HSALNG0013962 |  |
| piR-61240-151 |  |
| ENSG00000286034 |  |
| lnc-CEP76-2 |  |
| RF00994-258 |  |
| piR-56480-009 |  |
| piR-41245-052 |  |
| piR-40398 |  |
| DLST | Dihydrolipoamide S-Succinyltransferase |
| RFC1 | Replication Factor C Subunit 1 |
| CTBP2 | C-Terminal Binding Protein 2 |
| ANXA11 | Annexin A11 |
| NRIP1 | Nuclear Receptor Interacting Protein 1 |
| SLC25A11 | Solute Carrier Family 25 Member 11 |
| HCRTR2 | Hypocretin Receptor 2 |
| CALD1 | Caldesmon 1 |
| KDM4A | Lysine Demethylase 4A |
| DDX20 | DEAD-Box Helicase 20 |
| RPN2 | Ribophorin II |
| ART3 | ADP-Ribosyltransferase 3 |
| USF2 | Upstream Transcription Factor 2, C-Fos Interacting |
| NCAPG | Non-SMC Condensin I Complex Subunit G |
| MRPL1 | Mitochondrial Ribosomal Protein L1 |
| MRPL15 | Mitochondrial Ribosomal Protein L15 |
| VIL1 | Villin 1 |
| TIMM44 | Translocase Of Inner Mitochondrial Membrane 44 |
| SSBP3 | Single Stranded DNA Binding Protein 3 |
| STRAP | Serine/Threonine Kinase Receptor Associated Protein |
| MOB1B | MOB Kinase Activator 1B |
| MRPL11 | Mitochondrial Ribosomal Protein L11 |
| BCAS2 | BCAS2 Pre-MRNA Processing Factor |
| SDF4 | Stromal Cell Derived Factor 4 |
| IWS1 | Interacts With SUPT6H, CTD Assembly Factor 1 |
| MMS19 | MMS19 Homolog, Cytosolic Iron-Sulfur Assembly Component |
| MRPL49 | Mitochondrial Ribosomal Protein L49 |
| MRPL4 | Mitochondrial Ribosomal Protein L4 |
| MRPL43 | Mitochondrial Ribosomal Protein L43 |
| IPO4 | Importin 4 |
| ERH | ERH MRNA Splicing And Mitosis Factor |
| KCTD12 | Potassium Channel Tetramerization Domain Containing 12 |
| GID8 | GID Complex Subunit 8 Homolog |
| ENY2 | ENY2 Transcription And Export Complex 2 Subunit |
| MRPL52 | Mitochondrial Ribosomal Protein L52 |
| PLEKHG3 | Pleckstrin Homology And RhoGEF Domain Containing G3 |
| LTV1 | LTV1 Ribosome Biogenesis Factor |
| NOP9 | NOP9 Nucleolar Protein |
| QTRT2 | Queuine TRNA-Ribosyltransferase Accessory Subunit 2 |
| MACO1 | Macoilin 1 |
| QARS1 | Glutaminyl-TRNA Synthetase 1 |
| HDHD5 | Haloacid Dehalogenase Like Hydrolase Domain Containing 5 |
| CALHM6 | Calcium Homeostasis Modulator Family Member 6 |
| PRKAG1 | Protein Kinase AMP-Activated Non-Catalytic Subunit Gamma 1 |
| TRIB2 | Tribbles Pseudokinase 2 |
| NPY1R | Neuropeptide Y Receptor Y1 |
| LOXL2 | Lysyl Oxidase Like 2 |
| DAB1 | DAB Adaptor Protein 1 |
| ASIP | Agouti Signaling Protein |
| SIRT2 | Sirtuin 2 |
| H2BC4 | H2B Clustered Histone 4 |
| H2BC10 | H2B Clustered Histone 10 |
| H2BC7 | H2B Clustered Histone 7 |
| H2BC8 | H2B Clustered Histone 8 |
| SLC9A1 | Solute Carrier Family 9 Member A1 |
| FKBP4 | FKBP Prolyl Isomerase 4 |
| EIF2B4 | Eukaryotic Translation Initiation Factor 2B Subunit Delta |
| HNRNPA3 | Heterogeneous Nuclear Ribonucleoprotein A3 |
| GSDMA | Gasdermin A |
| NPM3 | Nucleophosmin/Nucleoplasmin 3 |
| MGA | MAX Dimerization Protein MGA |
| LDLRAP1 | Low Density Lipoprotein Receptor Adaptor Protein 1 |
| RIMS1 | Regulating Synaptic Membrane Exocytosis 1 |
| MKKS | McKusick-Kaufman Syndrome |
| LZTFL1 | Leucine Zipper Transcription Factor Like 1 |
| RAX2 | Retina And Anterior Neural Fold Homeobox 2 |
| UQCC3 | Ubiquinol-Cytochrome C Reductase Complex Assembly Factor 3 |
| CRIP1 | Cysteine Rich Protein 1 |
| PAK4 | P21 (RAC1) Activated Kinase 4 |
| PPP1R13L | Protein Phosphatase 1 Regulatory Subunit 13 Like |
| SAE1 | SUMO1 Activating Enzyme Subunit 1 |
| CCNH | Cyclin H |
| SSTR3 | Somatostatin Receptor 3 |
| NCAPD3 | Non-SMC Condensin II Complex Subunit D3 |
| SCG2 | Secretogranin II |
| ZW10 | Zw10 Kinetochore Protein |
| SNX6 | Sorting Nexin 6 |
| STRBP | Spermatid Perinuclear RNA Binding Protein |
| NT5DC2 | 5'-Nucleotidase Domain Containing 2 |
| FOXK1 | Forkhead Box K1 |
| XRN1 | 5'-3' Exoribonuclease 1 |
| SLAIN2 | SLAIN Motif Family Member 2 |
| N4BP1 | NEDD4 Binding Protein 1 |
| LARP4 | La Ribonucleoprotein 4 |
| EMC2 | ER Membrane Protein Complex Subunit 2 |
| WFDC8 | WAP Four-Disulfide Core Domain 8 |
| H2AC6 | H2A Clustered Histone 6 |
| LOC107963955 | BCR-ABL Major-Breakpoint Cluster Region |
| FZD5 | Frizzled Class Receptor 5 |
| ADD1 | Adducin 1 |
| NR0B2 | Nuclear Receptor Subfamily 0 Group B Member 2 |
| SYCP3 | Synaptonemal Complex Protein 3 |
| ZFYVE9 | Zinc Finger FYVE-Type Containing 9 |
| ST13 | ST13 Hsp70 Interacting Protein |
| CERS2 | Ceramide Synthase 2 |
| SMC2 | Structural Maintenance Of Chromosomes 2 |
| EXOSC1 | Exosome Component 1 |
| CHGB | Chromogranin B |
| ST13P4 | ST13, Hsp70 Interacting Protein Pseudogene 4 |
| MRPS17P5 | Mitochondrial Ribosomal Protein S17 Pseudogene 5 |
| RPS6KA4 | Ribosomal Protein S6 Kinase A4 |
| CYP26B1 | Cytochrome P450 Family 26 Subfamily B Member 1 |
| FLVCR2 | FLVCR Heme Transporter 2 |
| GKN2 | Gastrokine 2 |
| AKAP5 | A-Kinase Anchoring Protein 5 |
| DAP | Death Associated Protein |
| AVP | Arginine Vasopressin |
| NFE2L1 | Nuclear Factor, Erythroid 2 Like 1 |
| IGBP1 | Immunoglobulin Binding Protein 1 |
| PFKP | Phosphofructokinase, Platelet |
| DGKB | Diacylglycerol Kinase Beta |
| PAICS | Phosphoribosylaminoimidazole Carboxylase And Phosphoribosylaminoimidazolesuccinocarboxamide Synthase |
| STX6 | Syntaxin 6 |
| THOC2 | THO Complex 2 |
| EDF1 | Endothelial Differentiation Related Factor 1 |
| HERPUD1 | Homocysteine Inducible ER Protein With Ubiquitin Like Domain 1 |
| AHSP | Alpha Hemoglobin Stabilizing Protein |
| LRRC59 | Leucine Rich Repeat Containing 59 |
| ATP5PD | ATP Synthase Peripheral Stalk Subunit D |
| ACVR2A | Activin A Receptor Type 2A |
| CPM | Carboxypeptidase M |
| MSH3 | MutS Homolog 3 |
| PUM1 | Pumilio RNA Binding Family Member 1 |
| MBOAT7 | Membrane Bound O-Acyltransferase Domain Containing 7 |
| CADM3 | Cell Adhesion Molecule 3 |
| KCTD17 | Potassium Channel Tetramerization Domain Containing 17 |
| MTF2 | Metal Response Element Binding Transcription Factor 2 |
| MAGEA12 | MAGE Family Member A12 |
| DUX4 | Double Homeobox 4 |
| HEXA | Hexosaminidase Subunit Alpha |
| KCNJ13 | Potassium Inwardly Rectifying Channel Subfamily J Member 13 |
| UQCRB | Ubiquinol-Cytochrome C Reductase Binding Protein |
| TTLL5 | Tubulin Tyrosine Ligase Like 5 |
| TTC8 | Tetratricopeptide Repeat Domain 8 |
| SBSPON | Somatomedin B And Thrombospondin Type 1 Domain Containing |
| PYDC5 | Pyrin Domain Containing 5 |
| SERINC3 | Serine Incorporator 3 |
| SSC5D | Scavenger Receptor Cysteine Rich Family Member With 5 Domains |
| PRAME | Preferentially Expressed Antigen In Melanoma |
| LAMB1 | Laminin Subunit Beta 1 |
| GPX4 | Glutathione Peroxidase 4 |
| MIB1 | Mindbomb E3 Ubiquitin Protein Ligase 1 |
| LRPPRC | Leucine Rich Pentatricopeptide Repeat Containing |
| RPN1 | Ribophorin I |
| AIMP2 | Aminoacyl TRNA Synthetase Complex Interacting Multifunctional Protein 2 |
| RCC1 | Regulator Of Chromosome Condensation 1 |
| SART1 | Spliceosome Associated Factor 1, Recruiter Of U4/U6.U5 Tri-SnRNP |
| RABEP1 | Rabaptin, RAB GTPase Binding Effector Protein 1 |
| SSBP1 | Single Stranded DNA Binding Protein 1 |
| PI4K2B | Phosphatidylinositol 4-Kinase Type 2 Beta |
| SRGN | Serglycin |
| LGALS7 | Galectin 7 |
| SRPRB | SRP Receptor Subunit Beta |
| PLP2 | Proteolipid Protein 2 |
| SF3A3 | Splicing Factor 3a Subunit 3 |
| IMP3 | IMP U3 Small Nucleolar Ribonucleoprotein 3 |
| COMMD7 | COMM Domain Containing 7 |
| CORO2A | Coronin 2A |
| EXOSC4 | Exosome Component 4 |
| LHX2 | LIM Homeobox 2 |
| MICAL3 | Microtubule Associated Monooxygenase, Calponin And LIM Domain Containing 3 |
| ARPC5L | Actin Related Protein 2/3 Complex Subunit 5 Like |
| WDR6 | WD Repeat Domain 6 |
| RRP15 | Ribosomal RNA Processing 15 Homolog |
| PPP1R18 | Protein Phosphatase 1 Regulatory Subunit 18 |
| CHTOP | Chromatin Target Of PRMT1 |
| OTUD1 | OTU Deubiquitinase 1 |
| CSAG3 | CSAG Family Member 3 |
| CSAG2 | CSAG Family Member 2 |
| PKN1 | Protein Kinase N1 |
| THBS2 | Thrombospondin 2 |
| PTGFR | Prostaglandin F Receptor |
| RABGEF1 | RAB Guanine Nucleotide Exchange Factor 1 |
| RNF2 | Ring Finger Protein 2 |
| CAMLG | Calcium Modulating Ligand |
| GPA33 | Glycoprotein A33 |
| SUGP1 | SURP And G-Patch Domain Containing 1 |
| TIPARP | TCDD Inducible Poly(ADP-Ribose) Polymerase |
| ZNF318 | Zinc Finger Protein 318 |
| SCFV | Single-Chain Fv Fragment |
| PRND | Prion Like Protein Doppel |
| NID2 | Nidogen 2 |
| PPA1 | Inorganic Pyrophosphatase 1 |
| PPIC | Peptidylprolyl Isomerase C |
| PRELP | Proline And Arginine Rich End Leucine Rich Repeat Protein |
| RNF20 | Ring Finger Protein 20 |
| CBX1 | Chromobox 1 |
| PLXDC2 | Plexin Domain Containing 2 |
| RNF185 | Ring Finger Protein 185 |
| KRT23 | Keratin 23 |
| RAB15 | RAB15, Member RAS Oncogene Family |
| RBM25 | RNA Binding Motif Protein 25 |
| ANKRD30A | Ankyrin Repeat Domain 30A |
| SFTA3 | Surfactant Associated 3 |
| LOC102723878 | Uncharacterized LOC102723878 |
| AGPS | Alkylglycerone Phosphate Synthase |
| VLDLR | Very Low Density Lipoprotein Receptor |
| RRM1 | Ribonucleotide Reductase Catalytic Subunit M1 |
| SUCLA2 | Succinate-CoA Ligase ADP-Forming Subunit Beta |
| BCKDK | Branched Chain Keto Acid Dehydrogenase Kinase |
| STAG2 | Stromal Antigen 2 |
| ETFA | Electron Transfer Flavoprotein Subunit Alpha |
| GAK | Cyclin G Associated Kinase |
| MDH1 | Malate Dehydrogenase 1 |
| EIF2B1 | Eukaryotic Translation Initiation Factor 2B Subunit Alpha |
| GOSR2 | Golgi SNAP Receptor Complex Member 2 |
| ELOVL5 | ELOVL Fatty Acid Elongase 5 |
| NUDC | Nuclear Distribution C, Dynein Complex Regulator |
| PIGK | Phosphatidylinositol Glycan Anchor Biosynthesis Class K |
| RFC5 | Replication Factor C Subunit 5 |
| EDN2 | Endothelin 2 |
| OS9 | OS9 Endoplasmic Reticulum Lectin |
| MARCKS | Myristoylated Alanine Rich Protein Kinase C Substrate |
| AP3S1 | Adaptor Related Protein Complex 3 Subunit Sigma 1 |
| VPS53 | VPS53 Subunit Of GARP Complex |
| SMPD2 | Sphingomyelin Phosphodiesterase 2 |
| NOX3 | NADPH Oxidase 3 |
| RCC2 | Regulator Of Chromosome Condensation 2 |
| PDCD6 | Programmed Cell Death 6 |
| EIF2A | Eukaryotic Translation Initiation Factor 2A |
| SF1 | Splicing Factor 1 |
| UQCRH | Ubiquinol-Cytochrome C Reductase Hinge Protein |
| VTI1A | Vesicle Transport Through Interaction With T-SNAREs 1A |
| LMO4 | LIM Domain Only 4 |
| SUB1 | SUB1 Regulator Of Transcription |
| DCP1A | Decapping MRNA 1A |
| MRGPRX2 | MAS Related GPR Family Member X2 |
| BCL2L14 | BCL2 Like 14 |
| USP34 | Ubiquitin Specific Peptidase 34 |
| THADA | THADA Armadillo Repeat Containing |
| HOOK3 | Hook Microtubule Tethering Protein 3 |
| MPHOSPH8 | M-Phase Phosphoprotein 8 |
| TRMT10C | TRNA Methyltransferase 10C, Mitochondrial RNase P Subunit |
| RANBP6 | RAN Binding Protein 6 |
| SRP9 | Signal Recognition Particle 9 |
| PNRC1 | Proline Rich Nuclear Receptor Coactivator 1 |
| PPP6R1 | Protein Phosphatase 6 Regulatory Subunit 1 |
| MEAF6 | MYST/Esa1 Associated Factor 6 |
| BET1L | Bet1 Golgi Vesicular Membrane Trafficking Protein Like |
| ERP29 | Endoplasmic Reticulum Protein 29 |
| ZCCHC8 | Zinc Finger CCHC-Type Containing 8 |
| SPECC1 | Sperm Antigen With Calponin Homology And Coiled-Coil Domains 1 |
| YIF1B | Yip1 Interacting Factor Homolog B, Membrane Trafficking Protein |
| ZNF326 | Zinc Finger Protein 326 |
| SAAL1 | Serum Amyloid A Like 1 |
| LRRC25 | Leucine Rich Repeat Containing 25 |
| YLPM1 | YLP Motif Containing 1 |
| JADE2 | Jade Family PHD Finger 2 |
| COMMD2 | COMM Domain Containing 2 |
| ATP5F1C | ATP Synthase F1 Subunit Gamma |
| H2AC11 | H2A Clustered Histone 11 |
| ATP5MF | ATP Synthase Membrane Subunit F |
| DEFA1A3 | Defensin Alpha 1 And Alpha 3, Variable Copy Number Locus |
| LOC100420831 | Chromosome 1 Open Reading Frame 106 Pseudogene |
| ORAI3 | ORAI Calcium Release-Activated Calcium Modulator 3 |
| PRKAG2 | Protein Kinase AMP-Activated Non-Catalytic Subunit Gamma 2 |
| SIRT6 | Sirtuin 6 |
| CHRNA3 | Cholinergic Receptor Nicotinic Alpha 3 Subunit |
| MAP3K10 | Mitogen-Activated Protein Kinase Kinase Kinase 10 |
| JAG2 | Jagged Canonical Notch Ligand 2 |
| TAGLN | Transgelin |
| GRAP | GRB2 Related Adaptor Protein |
| USP25 | Ubiquitin Specific Peptidase 25 |
| DDX42 | DEAD-Box Helicase 42 |
| TRIP6 | Thyroid Hormone Receptor Interactor 6 |
| SELENOW | Selenoprotein W |
| NRL | Neural Retina Leucine Zipper |
| COL14A1 | Collagen Type XIV Alpha 1 Chain |
| AMN | Amnion Associated Transmembrane Protein |
| RP1 | RP1 Axonemal Microtubule Associated |
| SPATA7 | Spermatogenesis Associated 7 |
| C8orf37 | Chromosome 8 Open Reading Frame 37 |
| GDF10 | Growth Differentiation Factor 10 |
| CNIH1 | Cornichon Family AMPA Receptor Auxiliary Protein 1 |
| SPAG11A | Sperm Associated Antigen 11A |
| CTH | Cystathionine Gamma-Lyase |
| PARK7 | Parkinsonism Associated Deglycase |
| NDUFS1 | NADH:Ubiquinone Oxidoreductase Core Subunit S1 |
| IMPA2 | Inositol Monophosphatase 2 |
| GALNT2 | Polypeptide N-Acetylgalactosaminyltransferase 2 |
| UBQLN1 | Ubiquilin 1 |
| NDUFAF4 | NADH:Ubiquinone Oxidoreductase Complex Assembly Factor 4 |
| RARS2 | Arginyl-TRNA Synthetase 2, Mitochondrial |
| MC5R | Melanocortin 5 Receptor |
| ERLIN1 | ER Lipid Raft Associated 1 |
| RFC4 | Replication Factor C Subunit 4 |
| BTAF1 | B-TFIID TATA-Box Binding Protein Associated Factor 1 |
| PPFIBP1 | PPFIA Binding Protein 1 |
| TELO2 | Telomere Maintenance 2 |
| NCAPH | Non-SMC Condensin I Complex Subunit H |
| CDK3 | Cyclin Dependent Kinase 3 |
| REPS1 | RALBP1 Associated Eps Domain Containing 1 |
| REEP5 | Receptor Accessory Protein 5 |
| SLC30A9 | Solute Carrier Family 30 Member 9 |
| KIF14 | Kinesin Family Member 14 |
| STX12 | Syntaxin 12 |
| CNNM4 | Cyclin And CBS Domain Divalent Metal Cation Transport Mediator 4 |
| DIP2A | Disco Interacting Protein 2 Homolog A |
| SEC14L2 | SEC14 Like Lipid Binding 2 |
| RHOT2 | Ras Homolog Family Member T2 |
| RRBP1 | Ribosome Binding Protein 1 |
| SACM1L | SAC1 Like Phosphatidylinositide Phosphatase |
| AGPAT4 | 1-Acylglycerol-3-Phosphate O-Acyltransferase 4 |
| ATG10 | Autophagy Related 10 |
| ERAL1 | Era Like 12S Mitochondrial RRNA Chaperone 1 |
| WTAP | WT1 Associated Protein |
| LDB1 | LIM Domain Binding 1 |
| ING5 | Inhibitor Of Growth Family Member 5 |
| IPO11 | Importin 11 |
| TIMM50 | Translocase Of Inner Mitochondrial Membrane 50 |
| TMX3 | Thioredoxin Related Transmembrane Protein 3 |
| NCAPG2 | Non-SMC Condensin II Complex Subunit G2 |
| CSDE1 | Cold Shock Domain Containing E1 |
| HOOK2 | Hook Microtubule Tethering Protein 2 |
| CHCHD2 | Coiled-Coil-Helix-Coiled-Coil-Helix Domain Containing 2 |
| NOP58 | NOP58 Ribonucleoprotein |
| NOLC1 | Nucleolar And Coiled-Body Phosphoprotein 1 |
| ATP13A3 | ATPase 13A3 |
| ESYT1 | Extended Synaptotagmin 1 |
| USP39 | Ubiquitin Specific Peptidase 39 |
| ZNF175 | Zinc Finger Protein 175 |
| XPO4 | Exportin 4 |
| SLC25A28 | Solute Carrier Family 25 Member 28 |
| PACSIN3 | Protein Kinase C And Casein Kinase Substrate In Neurons 3 |
| MRPS6 | Mitochondrial Ribosomal Protein S6 |
| SAMM50 | SAMM50 Sorting And Assembly Machinery Component |
| FAM20B | FAM20B Glycosaminoglycan Xylosylkinase |
| ADGRL2 | Adhesion G Protein-Coupled Receptor L2 |
| FSTL3 | Follistatin Like 3 |
| TUBA3C | Tubulin Alpha 3c |
| LIMCH1 | LIM And Calponin Homology Domains 1 |
| PTGFRN | Prostaglandin F2 Receptor Inhibitor |
| SDF2L1 | Stromal Cell Derived Factor 2 Like 1 |
| PRPSAP1 | Phosphoribosyl Pyrophosphate Synthetase Associated Protein 1 |
| COLGALT1 | Collagen Beta(1-O)Galactosyltransferase 1 |
| MRPS11 | Mitochondrial Ribosomal Protein S11 |
| LUC7L2 | LUC7 Like 2, Pre-MRNA Splicing Factor |
| ARHGAP11A | Rho GTPase Activating Protein 11A |
| ANP32B | Acidic Nuclear Phosphoprotein 32 Family Member B |
| TBRG4 | Transforming Growth Factor Beta Regulator 4 |
| SSBP4 | Single Stranded DNA Binding Protein 4 |
| STX10 | Syntaxin 10 |
| MRPS10 | Mitochondrial Ribosomal Protein S10 |
| GNL3L | G Protein Nucleolar 3 Like |
| TMED9 | Transmembrane P24 Trafficking Protein 9 |
| SLC35E1 | Solute Carrier Family 35 Member E1 |
| CNOT6L | CCR4-NOT Transcription Complex Subunit 6 Like |
| SNAP47 | Synaptosome Associated Protein 47 |
| HNRNPUL2 | Heterogeneous Nuclear Ribonucleoprotein U Like 2 |
| CCDC85B | Coiled-Coil Domain Containing 85B |
| PSMG3 | Proteasome Assembly Chaperone 3 |
| MYO19 | Myosin XIX |
| UBN2 | Ubinuclein 2 |
| GYPE | Glycophorin E (MNS Blood Group) |
| H2AC20 | H2A Clustered Histone 20 |
| ARFGEF3 | ARFGEF Family Member 3 |
| WFDC3 | WAP Four-Disulfide Core Domain 3 |
| PHLDB3 | Pleckstrin Homology Like Domain Family B Member 3 |
| H2AC4 | H2A Clustered Histone 4 |
| KIAA1671 | KIAA1671 |
| H2AC7 | H2A Clustered Histone 7 |
| H2AC14 | H2A Clustered Histone 14 |
| ECPAS | Ecm29 Proteasome Adaptor And Scaffold |
| PALM2AKAP2 | PALM2 And AKAP2 Fusion |
| BMS1P20 | BMS1 Pseudogene 20 |
| RRM2B | Ribonucleotide Reductase Regulatory TP53 Inducible Subunit M2B |
| TBXA2R | Thromboxane A2 Receptor |
| PTGIR | Prostaglandin I2 Receptor |
| NPR2 | Natriuretic Peptide Receptor 2 |
| F2RL3 | F2R Like Thrombin Or Trypsin Receptor 3 |
| COL4A2 | Collagen Type IV Alpha 2 Chain |
| SATB2 | SATB Homeobox 2 |
| MAD2L1 | Mitotic Arrest Deficient 2 Like 1 |
| SQLE | Squalene Epoxidase |
| HYAL2 | Hyaluronidase 2 |
| CBX4 | Chromobox 4 |
| UVRAG | UV Radiation Resistance Associated |
| ST3GAL1 | ST3 Beta-Galactoside Alpha-2,3-Sialyltransferase 1 |
| CDH17 | Cadherin 17 |
| GSTM4 | Glutathione S-Transferase Mu 4 |
| RPL13A | Ribosomal Protein L13a |
| CLCN3 | Chloride Voltage-Gated Channel 3 |
| NSMAF | Neutral Sphingomyelinase Activation Associated Factor |
| SMOC1 | SPARC Related Modular Calcium Binding 1 |
| RPL24 | Ribosomal Protein L24 |
| USP3 | Ubiquitin Specific Peptidase 3 |
| GIGYF2 | GRB10 Interacting GYF Protein 2 |
| BCO2 | Beta-Carotene Oxygenase 2 |
| PLA2G15 | Phospholipase A2 Group XV |
| DDHD1 | DDHD Domain Containing 1 |
| MDGA2 | MAM Domain Containing Glycosylphosphatidylinositol Anchor 2 |
| ANKS1A | Ankyrin Repeat And Sterile Alpha Motif Domain Containing 1A |
| MAPRE1 | Microtubule Associated Protein RP/EB Family Member 1 |
| PDE4DIP | Phosphodiesterase 4D Interacting Protein |
| TMX1 | Thioredoxin Related Transmembrane Protein 1 |
| MRPL24 | Mitochondrial Ribosomal Protein L24 |
| AP3M1 | Adaptor Related Protein Complex 3 Subunit Mu 1 |
| TPD52L2 | TPD52 Like 2 |
| TPTE | Transmembrane Phosphatase With Tensin Homology |
| DHDH | Dihydrodiol Dehydrogenase |
| NUCKS1 | Nuclear Casein Kinase And Cyclin Dependent Kinase Substrate 1 |
| ANKFY1 | Ankyrin Repeat And FYVE Domain Containing 1 |
| MRPL47 | Mitochondrial Ribosomal Protein L47 |
| RAET1M | Retinoic Acid Early Transcript 1M Pseudogene |
| KRT18P39 | Keratin 18 Pseudogene 39 |
| ENPEP | Glutamyl Aminopeptidase |
| KAT6A | Lysine Acetyltransferase 6A |
| POLI | DNA Polymerase Iota |
| CDC25B | Cell Division Cycle 25B |
| AKR1C4 | Aldo-Keto Reductase Family 1 Member C4 |
| OXCT1 | 3-Oxoacid CoA-Transferase 1 |
| NDUFS8 | NADH:Ubiquinone Oxidoreductase Core Subunit S8 |
| ALDH1B1 | Aldehyde Dehydrogenase 1 Family Member B1 |
| ACAA2 | Acetyl-CoA Acyltransferase 2 |
| EFNB3 | Ephrin B3 |
| MRPS16 | Mitochondrial Ribosomal Protein S16 |
| NDUFA5 | NADH:Ubiquinone Oxidoreductase Subunit A5 |
| NDUFB10 | NADH:Ubiquinone Oxidoreductase Subunit B10 |
| POLRMT | RNA Polymerase Mitochondrial |
| CYB5B | Cytochrome B5 Type B |
| EHD1 | EH Domain Containing 1 |
| ARF4 | ADP Ribosylation Factor 4 |
| SMYD3 | SET And MYND Domain Containing 3 |
| FTSJ1 | FtsJ RNA 2'-O-Methyltransferase 1 |
| SIRT4 | Sirtuin 4 |
| SUCLG2 | Succinate-CoA Ligase GDP-Forming Subunit Beta |
| CTHRC1 | Collagen Triple Helix Repeat Containing 1 |
| MOB1A | MOB Kinase Activator 1A |
| CIAO1 | Cytosolic Iron-Sulfur Assembly Component 1 |
| GFM2 | G Elongation Factor Mitochondrial 2 |
| ATF7 | Activating Transcription Factor 7 |
| PELO | Pelota MRNA Surveillance And Ribosome Rescue Factor |
| TMEM59 | Transmembrane Protein 59 |
| GTSE1 | G2 And S-Phase Expressed 1 |
| DENND1A | DENN Domain Containing 1A |
| GNPTG | N-Acetylglucosamine-1-Phosphate Transferase Subunit Gamma |
| FIGNL1 | Fidgetin Like 1 |
| MAB21L2 | Mab-21 Like 2 |
| BMP2K | BMP2 Inducible Kinase |
| ART4 | ADP-Ribosyltransferase 4 (Dombrock Blood Group) |
| TRAPPC6B | Trafficking Protein Particle Complex 6B |
| TUBGCP4 | Tubulin Gamma Complex Associated Protein 4 |
| USP33 | Ubiquitin Specific Peptidase 33 |
| UBAP1 | Ubiquitin Associated Protein 1 |
| STX8 | Syntaxin 8 |
| TBC1D15 | TBC1 Domain Family Member 15 |
| SPAG5 | Sperm Associated Antigen 5 |
| IPO13 | Importin 13 |
| TIMM8B | Translocase Of Inner Mitochondrial Membrane 8 Homolog B |
| TMOD1 | Tropomodulin 1 |
| HIGD1A | HIG1 Hypoxia Inducible Domain Family Member 1A |
| DYM | Dymeclin |
| DYNLRB1 | Dynein Light Chain Roadblock-Type 1 |
| NAPG | NSF Attachment Protein Gamma |
| DHX32 | DEAH-Box Helicase 32 (Putative) |
| SDF2 | Stromal Cell Derived Factor 2 |
| MTPAP | Mitochondrial Poly(A) Polymerase |
| NOM1 | Nucleolar Protein With MIF4G Domain 1 |
| NUDT21 | Nudix Hydrolase 21 |
| RSL24D1 | Ribosomal L24 Domain Containing 1 |
| GEMIN4 | Gem Nuclear Organelle Associated Protein 4 |
| GEMIN6 | Gem Nuclear Organelle Associated Protein 6 |
| ESRP1 | Epithelial Splicing Regulatory Protein 1 |
| RDH13 | Retinol Dehydrogenase 13 |
| TRAPPC4 | Trafficking Protein Particle Complex 4 |
| VMP1 | Vacuole Membrane Protein 1 |
| TUBA3E | Tubulin Alpha 3e |
| PIGS | Phosphatidylinositol Glycan Anchor Biosynthesis Class S |
| STX18 | Syntaxin 18 |
| HIVEP1 | HIVEP Zinc Finger 1 |
| EIF1 | Eukaryotic Translation Initiation Factor 1 |
| MRPS18C | Mitochondrial Ribosomal Protein S18C |
| MRPS21 | Mitochondrial Ribosomal Protein S21 |
| MRPS33 | Mitochondrial Ribosomal Protein S33 |
| DNAJC1 | DnaJ Heat Shock Protein Family (Hsp40) Member C1 |
| DNAJC7 | DnaJ Heat Shock Protein Family (Hsp40) Member C7 |
| NIP7 | Nucleolar Pre-RRNA Processing Protein NIP7 |
| BET1 | Bet1 Golgi Vesicular Membrane Trafficking Protein |
| AUP1 | AUP1 Lipid Droplet Regulating VLDL Assembly Factor |
| VAMP5 | Vesicle Associated Membrane Protein 5 |
| SNRNP40 | Small Nuclear Ribonucleoprotein U5 Subunit 40 |
| ISL2 | ISL LIM Homeobox 2 |
| JMY | Junction Mediating And Regulatory Protein, P53 Cofactor |
| ST7 | Suppression Of Tumorigenicity 7 |
| CLPTM1 | CLPTM1 Regulator Of GABA Type A Receptor Forward Trafficking |
| GYPB | Glycophorin B (MNS Blood Group) |
| GTF3C1 | General Transcription Factor IIIC Subunit 1 |
| RINT1 | RAD50 Interactor 1 |
| NCLN | Nicalin |
| NEMF | Nuclear Export Mediator Factor |
| RMND5A | Required For Meiotic Nuclear Division 5 Homolog A |
| DCAKD | Dephospho-CoA Kinase Domain Containing |
| SEC62 | SEC62 Homolog, Preprotein Translocation Factor |
| MTX2 | Metaxin 2 |
| DNAJB12 | DnaJ Heat Shock Protein Family (Hsp40) Member B12 |
| ALDH8A1 | Aldehyde Dehydrogenase 8 Family Member A1 |
| ALG5 | ALG5 Dolichyl-Phosphate Beta-Glucosyltransferase |
| BNIP1 | BCL2 Interacting Protein 1 |
| BZW2 | Basic Leucine Zipper And W2 Domains 2 |
| LUC7L | LUC7 Like |
| ERLEC1 | Endoplasmic Reticulum Lectin 1 |
| TNPO2 | Transportin 2 |
| TRAPPC2L | Trafficking Protein Particle Complex 2 Like |
| TUBB8 | Tubulin Beta 8 Class VIII |
| USE1 | Unconventional SNARE In The ER 1 |
| VPS8 | VPS8 Subunit Of CORVET Complex |
| TOM1L1 | Target Of Myb1 Like 1 Membrane Trafficking Protein |
| CLCC1 | Chloride Channel CLIC Like 1 |
| GPKOW | G-Patch Domain And KOW Motifs |
| IPO9 | Importin 9 |
| KNSTRN | Kinetochore Localized Astrin (SPAG5) Binding Protein |
| MON2 | MON2 Homolog, Regulator Of Endosome-To-Golgi Trafficking |
| EI24 | EI24 Autophagy Associated Transmembrane Protein |
| HAUS6 | HAUS Augmin Like Complex Subunit 6 |
| CPNE8 | Copine 8 |
| COPG1 | COPI Coat Complex Subunit Gamma 1 |
| MRPL50 | Mitochondrial Ribosomal Protein L50 |
| MRPL9 | Mitochondrial Ribosomal Protein L9 |
| MRPL30 | Mitochondrial Ribosomal Protein L30 |
| SCCPDH | Saccharopine Dehydrogenase (Putative) |
| NUDT19 | Nudix Hydrolase 19 |
| FAM160B1 | Family With Sequence Similarity 160 Member B1 |
| BZW1 | Basic Leucine Zipper And W2 Domains 1 |
| FCHO2 | FCH And Mu Domain Containing Endocytic Adaptor 2 |
| TOR3A | Torsin Family 3 Member A |
| TRAPPC5 | Trafficking Protein Particle Complex 5 |
| TXLNB | Taxilin Beta |
| UBL4A | Ubiquitin Like 4A |
| YIPF3 | Yip1 Domain Family Member 3 |
| LANCL2 | LanC Like 2 |
| CAND2 | Cullin Associated And Neddylation Dissociated 2 (Putative) |
| PSMG2 | Proteasome Assembly Chaperone 2 |
| COPS4 | COP9 Signalosome Subunit 4 |
| MRPL51 | Mitochondrial Ribosomal Protein L51 |
| SCFD2 | Sec1 Family Domain Containing 2 |
| SCGB2A1 | Secretoglobin Family 2A Member 1 |
| EMC1 | ER Membrane Protein Complex Subunit 1 |
| CDKN2AIP | CDKN2A Interacting Protein |
| GET3 | Guided Entry Of Tail-Anchored Proteins Factor 3, ATPase |
| CCDC93 | Coiled-Coil Domain Containing 93 |
| ATP5F1D | ATP Synthase F1 Subunit Delta |
| EVI5L | Ecotropic Viral Integration Site 5 Like |
| RABL3 | RAB, Member Of RAS Oncogene Family Like 3 |
| TBC1D9B | TBC1 Domain Family Member 9B |
| PCMTD2 | Protein-L-Isoaspartate (D-Aspartate) O-Methyltransferase Domain Containing 2 |
| GRAMD1A | GRAM Domain Containing 1A |
| GTPBP10 | GTP Binding Protein 10 |
| CEP44 | Centrosomal Protein 44 |
| C1orf112 | Chromosome 1 Open Reading Frame 112 |
| ZNF740 | Zinc Finger Protein 740 |
| LMBRD2 | LMBR1 Domain Containing 2 |
| KIAA0355 | KIAA0355 |
| TMEM183A | Transmembrane Protein 183A |
| TMEM255A | Transmembrane Protein 255A |
| HEATR3 | HEAT Repeat Containing 3 |
| HNRNPA1L2 | Heterogeneous Nuclear Ribonucleoprotein A1 Like 2 |
| EMC3 | ER Membrane Protein Complex Subunit 3 |
| BHLHE23 | Basic Helix-Loop-Helix Family Member E23 |
| ATP5F1E | ATP Synthase F1 Subunit Epsilon |
| ATP5PF | ATP Synthase Peripheral Stalk Subunit F6 |
| C19orf25 | Chromosome 19 Open Reading Frame 25 |
| VWA8 | Von Willebrand Factor A Domain Containing 8 |
| WFDC12 | WAP Four-Disulfide Core Domain 12 |
| TRMT1L | TRNA Methyltransferase 1 Like |
| LRRC47 | Leucine Rich Repeat Containing 47 |
| FLYWCH2 | FLYWCH Family Member 2 |
| THEM6 | Thioesterase Superfamily Member 6 |
| MAP11 | Microtubule Associated Protein 11 |
| CIAO2B | Cytosolic Iron-Sulfur Assembly Component 2B |
| ATP5ME | ATP Synthase Membrane Subunit E |
| TRIM52 | Tripartite Motif Containing 52 |
| MAGED4 | MAGE Family Member D4 |
| ANXA2R | Annexin A2 Receptor |
| TUT7 | Terminal Uridylyl Transferase 7 |
| LNPK | Lunapark, ER Junction Formation Factor |
| AK6 | Adenylate Kinase 6 |
| MAIP1 | Matrix AAA Peptidase Interacting Protein 1 |
| RETREG3 | Reticulophagy Regulator Family Member 3 |
| DMAC2 | Distal Membrane Arm Assembly Complex 2 |
| MCRIP1 | MAPK Regulated Corepressor Interacting Protein 1 |
| CRACD | Capping Protein Inhibiting Regulator Of Actin Dynamics |
| PGAP4 | Post-GPI Attachment To Proteins GalNAc Transferase 4 |
| FAM241B | Family With Sequence Similarity 241 Member B |
| SMIM11A | Small Integral Membrane Protein 11A |
| C11orf98 | Chromosome 11 Open Reading Frame 98 |
| HSP90AB2P | Heat Shock Protein 90 Alpha Family Class B Member 2, Pseudogene |
| NFIA | Nuclear Factor I A |
| LTBP2 | Latent Transforming Growth Factor Beta Binding Protein 2 |
| HDGF | Heparin Binding Growth Factor |
| SRR | Serine Racemase |
| P2RX2 | Purinergic Receptor P2X 2 |
| PRMT3 | Protein Arginine Methyltransferase 3 |
| SLC14A1 | Solute Carrier Family 14 Member 1 (Kidd Blood Group) |
| DAD1 | Defender Against Cell Death 1 |
| DAGLA | Diacylglycerol Lipase Alpha |
| RAB6B | RAB6B, Member RAS Oncogene Family |
| MFNG | MFNG O-Fucosylpeptide 3-Beta-N-Acetylglucosaminyltransferase |
| SLA | Src Like Adaptor |
| NCEH1 | Neutral Cholesterol Ester Hydrolase 1 |
| NDRG2 | NDRG Family Member 2 |
| ATP10A | ATPase Phospholipid Transporting 10A (Putative) |
| SUPV3L1 | Suv3 Like RNA Helicase |
| ERRFI1 | ERBB Receptor Feedback Inhibitor 1 |
| TSPAN2 | Tetraspanin 2 |
| GABPB2 | GA Binding Protein Transcription Factor Subunit Beta 2 |
| AIF1L | Allograft Inflammatory Factor 1 Like |
| ADAM7 | ADAM Metallopeptidase Domain 7 |
| L3MBTL2 | L3MBTL Histone Methyl-Lysine Binding Protein 2 |
| PPP1R16B | Protein Phosphatase 1 Regulatory Subunit 16B |
| ATP5MD | ATP Synthase Membrane Subunit DAPIT |
| UCN2 | Urocortin 2 |
| TMIGD2 | Transmembrane And Immunoglobulin Domain Containing 2 |
| NR1D1 | Nuclear Receptor Subfamily 1 Group D Member 1 |
| CHRNA2 | Cholinergic Receptor Nicotinic Alpha 2 Subunit |
| CDK7 | Cyclin Dependent Kinase 7 |
| CYP3A7 | Cytochrome P450 Family 3 Subfamily A Member 7 |
| PLCD4 | Phospholipase C Delta 4 |
| DGAT2 | Diacylglycerol O-Acyltransferase 2 |
| DNAJB2 | DnaJ Heat Shock Protein Family (Hsp40) Member B2 |
| ETF1 | Eukaryotic Translation Termination Factor 1 |
| TRHR | Thyrotropin Releasing Hormone Receptor |
| PLCD3 | Phospholipase C Delta 3 |
| RHOT1 | Ras Homolog Family Member T1 |
| CLK1 | CDC Like Kinase 1 |
| IQSEC1 | IQ Motif And Sec7 Domain ArfGEF 1 |
| LIN28A | Lin-28 Homolog A |
| NR2F6 | Nuclear Receptor Subfamily 2 Group F Member 6 |
| P4HA1 | Prolyl 4-Hydroxylase Subunit Alpha 1 |
| KIF21A | Kinesin Family Member 21A |
| STRN | Striatin |
| CDK19 | Cyclin Dependent Kinase 19 |
| MATN2 | Matrilin 2 |
| DUSP13 | Dual Specificity Phosphatase 13 |
| MYOZ2 | Myozenin 2 |
| CDC42BPG | CDC42 Binding Protein Kinase Gamma |
| LPGAT1 | Lysophosphatidylglycerol Acyltransferase 1 |
| LRCH4 | Leucine Rich Repeats And Calponin Homology Domain Containing 4 |
| CNNM3 | Cyclin And CBS Domain Divalent Metal Cation Transport Mediator 3 |
| HCAR1 | Hydroxycarboxylic Acid Receptor 1 |
| DNAJC2 | DnaJ Heat Shock Protein Family (Hsp40) Member C2 |
| IFRD2 | Interferon Related Developmental Regulator 2 |
| ITPRIP | Inositol 1,4,5-Trisphosphate Receptor Interacting Protein |
| DDX28 | DEAD-Box Helicase 28 |
| ATF5 | Activating Transcription Factor 5 |
| TRIM72 | Tripartite Motif Containing 72 |
| IKBIP | IKBKB Interacting Protein |
| MTPN | Myotrophin |
| FOXK2 | Forkhead Box K2 |
| BOP1 | BOP1 Ribosomal Biogenesis Factor |
| UXT | Ubiquitously Expressed Prefoldin Like Chaperone |
| SESTD1 | SEC14 And Spectrin Domain Containing 1 |
| MRPL41 | Mitochondrial Ribosomal Protein L41 |
| DCUN1D5 | Defective In Cullin Neddylation 1 Domain Containing 5 |
| FCF1 | FCF1 RRNA-Processing Protein |
| TXLNG | Taxilin Gamma |
| DCAF4 | DDB1 And CUL4 Associated Factor 4 |
| KYAT3 | Kynurenine Aminotransferase 3 |
| ZNF395 | Zinc Finger Protein 395 |
| WFDC6 | WAP Four-Disulfide Core Domain 6 |
| CARD19 | Caspase Recruitment Domain Family Member 19 |
| WFDC10B | WAP Four-Disulfide Core Domain 10B |
| WFDC11 | WAP Four-Disulfide Core Domain 11 |
| WFDC9 | WAP Four-Disulfide Core Domain 9 |
| WFDC13 | WAP Four-Disulfide Core Domain 13 |
| WFDC10A | WAP Four-Disulfide Core Domain 10A |
| GVINP1 | GTPase, Very Large Interferon Inducible Pseudogene 1 |
| P2RY11 | Purinergic Receptor P2Y11 |
| BMP3 | Bone Morphogenetic Protein 3 |
| FAM3A | FAM3 Metabolism Regulating Signaling Molecule A |
| HMGB4 | High Mobility Group Box 4 |
| CD8B2 | CD8b2 Molecule |
| ENSG00000173366 |  |
| CPT1B | Carnitine Palmitoyltransferase 1B |
| PLPP1 | Phospholipid Phosphatase 1 |
| KIR2DP1 | Killer Cell Immunoglobulin Like Receptor, Two Ig Domains Pseudogene 1 |
| CARS2 | Cysteinyl-TRNA Synthetase 2, Mitochondrial |
| PDGFC | Platelet Derived Growth Factor C |
| TIMP4 | TIMP Metallopeptidase Inhibitor 4 |
| STAMBP | STAM Binding Protein |
| MAP4K5 | Mitogen-Activated Protein Kinase Kinase Kinase Kinase 5 |
| PBK | PDZ Binding Kinase |
| ING1 | Inhibitor Of Growth Family Member 1 |
| NMT1 | N-Myristoyltransferase 1 |
| RPL22 | Ribosomal Protein L22 |
| FOXG1 | Forkhead Box G1 |
| MBTPS1 | Membrane Bound Transcription Factor Peptidase, Site 1 |
| ESPL1 | Extra Spindle Pole Bodies Like 1, Separase |
| MBD3 | Methyl-CpG Binding Domain Protein 3 |
| ENTPD5 | Ectonucleoside Triphosphate Diphosphohydrolase 5 (Inactive) |
| SLC25A21 | Solute Carrier Family 25 Member 21 |
| ZBTB17 | Zinc Finger And BTB Domain Containing 17 |
| PACSIN1 | Protein Kinase C And Casein Kinase Substrate In Neurons 1 |
| CLP1 | Cleavage Factor Polyribonucleotide Kinase Subunit 1 |
| LSM3 | LSM3 Homolog, U6 Small Nuclear RNA And MRNA Degradation Associated |
| SASH1 | SAM And SH3 Domain Containing 1 |
| GGT5 | Gamma-Glutamyltransferase 5 |
| BAG4 | BAG Cochaperone 4 |
| IMMP2L | Inner Mitochondrial Membrane Peptidase Subunit 2 |
| ARID4A | AT-Rich Interaction Domain 4A |
| ESRP2 | Epithelial Splicing Regulatory Protein 2 |
| HAPLN4 | Hyaluronan And Proteoglycan Link Protein 4 |
| PTPRCAP | Protein Tyrosine Phosphatase Receptor Type C Associated Protein |
| WFDC1 | WAP Four-Disulfide Core Domain 1 |
| LONRF3 | LON Peptidase N-Terminal Domain And Ring Finger 3 |
| YPEL4 | Yippee Like 4 |
| TMEM219 | Transmembrane Protein 219 |
| GDPD3 | Glycerophosphodiester Phosphodiesterase Domain Containing 3 |
| CCDC88B | Coiled-Coil Domain Containing 88B |
| TEDC1 | Tubulin Epsilon And Delta Complex 1 |
| HPF1 | Histone PARylation Factor 1 |
| PHF14 | PHD Finger Protein 14 |
| TACSTD2 | Tumor Associated Calcium Signal Transducer 2 |
| ESRRA | Estrogen Related Receptor Alpha |
| ETFB | Electron Transfer Flavoprotein Subunit Beta |
| SLC6A6 | Solute Carrier Family 6 Member 6 |
| HSD17B2 | Hydroxysteroid 17-Beta Dehydrogenase 2 |
| FZD7 | Frizzled Class Receptor 7 |
| CBR3 | Carbonyl Reductase 3 |
| SLC23A1 | Solute Carrier Family 23 Member 1 |
| INPP5K | Inositol Polyphosphate-5-Phosphatase K |
| TFPI2 | Tissue Factor Pathway Inhibitor 2 |
| PRIM1 | DNA Primase Subunit 1 |
| SLC23A2 | Solute Carrier Family 23 Member 2 |
| TP53I3 | Tumor Protein P53 Inducible Protein 3 |
| CDIPT | CDP-Diacylglycerol--Inositol 3-Phosphatidyltransferase |
| ATP1B2 | ATPase Na+/K+ Transporting Subunit Beta 2 |
| UGT1A4 | UDP Glucuronosyltransferase Family 1 Member A4 |
| PLA1A | Phospholipase A1 Member A |
| EIF5A2 | Eukaryotic Translation Initiation Factor 5A2 |
| FOXA3 | Forkhead Box A3 |
| MED17 | Mediator Complex Subunit 17 |
| B3GNT3 | UDP-GlcNAc:BetaGal Beta-1,3-N-Acetylglucosaminyltransferase 3 |
| NUBP2 | Nucleotide Binding Protein 2 |
| RGS17 | Regulator Of G Protein Signaling 17 |
| DIO1 | Iodothyronine Deiodinase 1 |
| OLIG1 | Oligodendrocyte Transcription Factor 1 |
| RAB34 | RAB34, Member RAS Oncogene Family |
| ABI3 | ABI Family Member 3 |
| TRMT2A | TRNA Methyltransferase 2 Homolog A |
| MORF4L2 | Mortality Factor 4 Like 2 |
| NFXL1 | Nuclear Transcription Factor, X-Box Binding Like 1 |
| S100A16 | S100 Calcium Binding Protein A16 |
| DUOXA1 | Dual Oxidase Maturation Factor 1 |
| AP5Z1 | Adaptor Related Protein Complex 5 Subunit Zeta 1 |
| GDE1 | Glycerophosphodiester Phosphodiesterase 1 |
| PDE12 | Phosphodiesterase 12 |
| PLAC1 | Placenta Enriched 1 |
| PLEKHB2 | Pleckstrin Homology Domain Containing B2 |
| SPNS1 | Sphingolipid Transporter 1 (Putative) |
| MLF2 | Myeloid Leukemia Factor 2 |
| FAM184A | Family With Sequence Similarity 184 Member A |
| WDFY1 | WD Repeat And FYVE Domain Containing 1 |
| PRM2 | Protamine 2 |
| ZCCHC17 | Zinc Finger CCHC-Type Containing 17 |
| SPINK4 | Serine Peptidase Inhibitor Kazal Type 4 |
| GOLT1B | Golgi Transport 1B |
| MAP1LC3C | Microtubule Associated Protein 1 Light Chain 3 Gamma |
| TCAF1 | TRPM8 Channel Associated Factor 1 |
| C8orf33 | Chromosome 8 Open Reading Frame 33 |
| HNRNPCL1 | Heterogeneous Nuclear Ribonucleoprotein C Like 1 |
| EFCAB11 | EF-Hand Calcium Binding Domain 11 |
| S100A7L2 | S100 Calcium Binding Protein A7 Like 2 |
| TDRP | Testis Development Related Protein |
| H2BU1 | H2B.U Histone 1 |
| CCDC191 | Coiled-Coil Domain Containing 191 |
| MIR301A | MicroRNA 301a |
| NACA4P | NACA Family Member 4, Pseudogene |
| CST9LP1 | Cystatin 9-Like Pseudogene 1 |
| SIRPB3P | Signal Regulatory Protein Beta 3, Pseudogene |
| SIRPAP1 | Signal Regulatory Protein Alpha Pseudogene 1 |
| LOC100289473 | Cytoskeleton Associated Protein 2-Like Pseudogene |
| EGID-100124696 | Transient Erythroblastopenia Of Childhood |
| DSE | Dermatan Sulfate Epimerase |
| LGALS2 | Galectin 2 |
| MGAT5 | Alpha-1,6-Mannosylglycoprotein 6-Beta-N-Acetylglucosaminyltransferase |
| USH1G | USH1 Protein Network Component Sans |
| RFX4 | Regulatory Factor X4 |
| STYX | Serine/Threonine/Tyrosine Interacting Protein |
| NUMB | NUMB Endocytic Adaptor Protein |
| TEAD1 | TEA Domain Transcription Factor 1 |
| AMT | Aminomethyltransferase |
| HTR1D | 5-Hydroxytryptamine Receptor 1D |
| HIF1AN | Hypoxia Inducible Factor 1 Subunit Alpha Inhibitor |
| IGFALS | Insulin Like Growth Factor Binding Protein Acid Labile Subunit |
| SOAT2 | Sterol O-Acyltransferase 2 |
| CRYM | Crystallin Mu |
| MED12 | Mediator Complex Subunit 12 |
| ABCA5 | ATP Binding Cassette Subfamily A Member 5 |
| FMO1 | Flavin Containing Dimethylaniline Monoxygenase 1 |
| SLC28A2 | Solute Carrier Family 28 Member 2 |
| BHMT | Betaine--Homocysteine S-Methyltransferase |
| APLP2 | Amyloid Beta Precursor Like Protein 2 |
| HUS1 | HUS1 Checkpoint Clamp Component |
| BBOX1 | Gamma-Butyrobetaine Hydroxylase 1 |
| NUBP1 | Nucleotide Binding Protein 1 |
| PKP4 | Plakophilin 4 |
| PKIA | CAMP-Dependent Protein Kinase Inhibitor Alpha |
| ALDH1L1 | Aldehyde Dehydrogenase 1 Family Member L1 |
| SCUBE2 | Signal Peptide, CUB Domain And EGF Like Domain Containing 2 |
| SAT2 | Spermidine/Spermine N1-Acetyltransferase Family Member 2 |
| MEST | Mesoderm Specific Transcript |
| ABCA6 | ATP Binding Cassette Subfamily A Member 6 |
| RERG | RAS Like Estrogen Regulated Growth Inhibitor |
| MYCBP2 | MYC Binding Protein 2 |
| APOL6 | Apolipoprotein L6 |
| VTA1 | Vesicle Trafficking 1 |
| PPP1R11 | Protein Phosphatase 1 Regulatory Inhibitor Subunit 11 |
| TAS2R14 | Taste 2 Receptor Member 14 |
| MPHOSPH10 | M-Phase Phosphoprotein 10 |
| RCSD1 | RCSD Domain Containing 1 |
| JRK | Jrk Helix-Turn-Helix Protein |
| DEPDC7 | DEP Domain Containing 7 |
| RPA4 | Replication Protein A4 |
| BSN | Bassoon Presynaptic Cytomatrix Protein |
| TROAP | Trophinin Associated Protein |
| H2AZ1 | H2A.Z Variant Histone 1 |
| DNAJC18 | DnaJ Heat Shock Protein Family (Hsp40) Member C18 |
| ZNF230 | Zinc Finger Protein 230 |
| HILPDA | Hypoxia Inducible Lipid Droplet Associated |
| CRISPLD1 | Cysteine Rich Secretory Protein LCCL Domain Containing 1 |
| SYCP2L | Synaptonemal Complex Protein 2 Like |
| RMDN2 | Regulator Of Microtubule Dynamics 2 |
| CCDC110 | Coiled-Coil Domain Containing 110 |
| BROX | BRO1 Domain And CAAX Motif Containing |
| WFDC5 | WAP Four-Disulfide Core Domain 5 |
| SEPTIN4 | Septin 4 |
| PYM1 | PYM Homolog 1, Exon Junction Complex Associated Factor |
| CCDC168 | Coiled-Coil Domain Containing 168 |
| GPR33 | G Protein-Coupled Receptor 33 (Gene/Pseudogene) |
| ZNF879 | Zinc Finger Protein 879 |
| SNORA31 | Small Nucleolar RNA, H/ACA Box 31 |
| CFAP58-DT | CFAP58 Divergent Transcript |
| BNIP3P6 | BCL2 Interacting Protein 3 Pseudogene 6 |
| PDYN | Prodynorphin |
| BCAT1 | Branched Chain Amino Acid Transaminase 1 |
| FNTB | Farnesyltransferase, CAAX Box, Beta |
| DHRS4 | Dehydrogenase/Reductase 4 |
| ATP2B4 | ATPase Plasma Membrane Ca2+ Transporting 4 |
| SLC24A4 | Solute Carrier Family 24 Member 4 |
| SENP1 | SUMO Specific Peptidase 1 |
| INHBB | Inhibin Subunit Beta B |
| PTPRE | Protein Tyrosine Phosphatase Receptor Type E |
| SOCS6 | Suppressor Of Cytokine Signaling 6 |
| TSN | Translin |
| SLC6A14 | Solute Carrier Family 6 Member 14 |
| MYBL1 | MYB Proto-Oncogene Like 1 |
| STX2 | Syntaxin 2 |
| NEIL2 | Nei Like DNA Glycosylase 2 |
| ELP3 | Elongator Acetyltransferase Complex Subunit 3 |
| SLC28A3 | Solute Carrier Family 28 Member 3 |
| CREB3L3 | CAMP Responsive Element Binding Protein 3 Like 3 |
| GPRC5C | G Protein-Coupled Receptor Class C Group 5 Member C |
| MTSS1 | MTSS I-BAR Domain Containing 1 |
| DOCK10 | Dedicator Of Cytokinesis 10 |
| ELK4 | ETS Transcription Factor ELK4 |
| CUZD1 | CUB And Zona Pellucida Like Domains 1 |
| B4GAT1 | Beta-1,4-Glucuronyltransferase 1 |
| UNK | Unk Zinc Finger |
| EMX1 | Empty Spiracles Homeobox 1 |
| TMEM260 | Transmembrane Protein 260 |
| DHRS7B | Dehydrogenase/Reductase 7B |
| CHCHD7 | Coiled-Coil-Helix-Coiled-Coil-Helix Domain Containing 7 |
| SNX31 | Sorting Nexin 31 |
| LRRC19 | Leucine Rich Repeat Containing 19 |
| VCPKMT | Valosin Containing Protein Lysine Methyltransferase |
| CSRNP3 | Cysteine And Serine Rich Nuclear Protein 3 |
| TTC9 | Tetratricopeptide Repeat Domain 9 |
| LRMDA | Leucine Rich Melanocyte Differentiation Associated |
| TMEM64 | Transmembrane Protein 64 |
| FAM92A | Family With Sequence Similarity 92 Member A |
| NUGGC | Nuclear GTPase, Germinal Center Associated |
| RIOX1 | Ribosomal Oxygenase 1 |
| GNRH2 | Gonadotropin Releasing Hormone 2 |
| CCPG1 | Cell Cycle Progression 1 |
| COL4A6 | Collagen Type IV Alpha 6 Chain |
| TINAG | Tubulointerstitial Nephritis Antigen |
| GPR39 | G Protein-Coupled Receptor 39 |
| SNURF | SNRPN Upstream Reading Frame |
| RGS21 | Regulator Of G Protein Signaling 21 |
| RPS2P19 | Ribosomal Protein S2 Pseudogene 19 |
| SUMO3 | Small Ubiquitin Like Modifier 3 |
| LRPAP1 | LDL Receptor Related Protein Associated Protein 1 |
| THRA | Thyroid Hormone Receptor Alpha |
| FADS2 | Fatty Acid Desaturase 2 |
| SLC34A2 | Solute Carrier Family 34 Member 2 |
| CHMP2B | Charged Multivesicular Body Protein 2B |
| TLK1 | Tousled Like Kinase 1 |
| HAAO | 3-Hydroxyanthranilate 3,4-Dioxygenase |
| HSD17B6 | Hydroxysteroid 17-Beta Dehydrogenase 6 |
| AGAP2 | ArfGAP With GTPase Domain, Ankyrin Repeat And PH Domain 2 |
| AAK1 | AP2 Associated Kinase 1 |
| EPB41L3 | Erythrocyte Membrane Protein Band 4.1 Like 3 |
| VPS4B | Vacuolar Protein Sorting 4 Homolog B |
| MYT1 | Myelin Transcription Factor 1 |
| HOMER1 | Homer Scaffold Protein 1 |
| CHMP1A | Charged Multivesicular Body Protein 1A |
| MCM8 | Minichromosome Maintenance 8 Homologous Recombination Repair Factor |
| CAPN7 | Calpain 7 |
| SESN3 | Sestrin 3 |
| CHST12 | Carbohydrate Sulfotransferase 12 |
| CHMP2A | Charged Multivesicular Body Protein 2A |
| CHMP3 | Charged Multivesicular Body Protein 3 |
| MAGOH | Mago Homolog, Exon Junction Complex Subunit |
| APPBP2 | Amyloid Beta Precursor Protein Binding Protein 2 |
| SEC16A | SEC16 Homolog A, Endoplasmic Reticulum Export Factor |
| RHBDL2 | Rhomboid Like 2 |
| DKKL1 | Dickkopf Like Acrosomal Protein 1 |
| CLUAP1 | Clusterin Associated Protein 1 |
| HELQ | Helicase, POLQ Like |
| CEP68 | Centrosomal Protein 68 |
| DUSP12 | Dual Specificity Phosphatase 12 |
| RAB3B | RAB3B, Member RAS Oncogene Family |
| HMBOX1 | Homeobox Containing 1 |
| DNASE2B | Deoxyribonuclease 2 Beta |
| RPL22L1 | Ribosomal Protein L22 Like 1 |
| ERMN | Ermin |
| TPSG1 | Tryptase Gamma 1 |
| MRPL38 | Mitochondrial Ribosomal Protein L38 |
| LSG1 | Large 60S Subunit Nuclear Export GTPase 1 |
| ZBTB11 | Zinc Finger And BTB Domain Containing 11 |
| ZNF93 | Zinc Finger Protein 93 |
| ZNF385A | Zinc Finger Protein 385A |
| RNF44 | Ring Finger Protein 44 |
| TTC9C | Tetratricopeptide Repeat Domain 9C |
| RBMXL1 | RBMX Like 1 |
| PIP4P1 | Phosphatidylinositol-4,5-Bisphosphate 4-Phosphatase 1 |
| PAGE5 | PAGE Family Member 5 |
| TMEM150B | Transmembrane Protein 150B |
| SLX4IP | SLX4 Interacting Protein |
| PIP4P2 | Phosphatidylinositol-4,5-Bisphosphate 4-Phosphatase 2 |
| NAA80 | N-Alpha-Acetyltransferase 80, NatH Catalytic Subunit |
| KLRF2 | Killer Cell Lectin Like Receptor F2 |
| MIR3142HG | MIR3142 Host Gene |
| COX6CP1 | Cytochrome C Oxidase Subunit 6C Pseudogene 1 |
| RNA5SP141 | RNA, 5S Ribosomal Pseudogene 141 |
| GSTT1-AS1 | GSTT1 Antisense RNA 1 |
| LOC102723403 | Uncharacterized LOC102723403 |
| RAC3 | Rac Family Small GTPase 3 |
| GRIP1 | Glutamate Receptor Interacting Protein 1 |
| COL12A1 | Collagen Type XII Alpha 1 Chain |
| UQCRQ | Ubiquinol-Cytochrome C Reductase Complex III Subunit VII |
| APCDD1 | APC Down-Regulated 1 |
| KIF7 | Kinesin Family Member 7 |
| OPN1LW | Opsin 1, Long Wave Sensitive |
| SUSD2 | Sushi Domain Containing 2 |
| CHID1 | Chitinase Domain Containing 1 |
| CHRNA4 | Cholinergic Receptor Nicotinic Alpha 4 Subunit |
| DKK3 | Dickkopf WNT Signaling Pathway Inhibitor 3 |
| ENTPD7 | Ectonucleoside Triphosphate Diphosphohydrolase 7 |
| ANGPTL2 | Angiopoietin Like 2 |
| KLF7 | Kruppel Like Factor 7 |
| RNASE1 | Ribonuclease A Family Member 1, Pancreatic |
| SETD7 | SET Domain Containing 7, Histone Lysine Methyltransferase |
| P2RX3 | Purinergic Receptor P2X 3 |
| MGAT5B | Alpha-1,6-Mannosylglycoprotein 6-Beta-N-Acetylglucosaminyltransferase B |
| LGALS13 | Galectin 13 |
| VENTX | VENT Homeobox |
| LPIN1 | Lipin 1 |
| WNK1 | WNK Lysine Deficient Protein Kinase 1 |
| LRAT | Lecithin Retinol Acyltransferase |
| GUCY2D | Guanylate Cyclase 2D, Retinal |
| PRPF4 | Pre-MRNA Processing Factor 4 |
| NR2E3 | Nuclear Receptor Subfamily 2 Group E Member 3 |
| BBS9 | Bardet-Biedl Syndrome 9 |
| DRAM2 | DNA Damage Regulated Autophagy Modulator 2 |
| RP9 | RP9 Pre-MRNA Splicing Factor |
| TMEM237 | Transmembrane Protein 237 |
| UQCC2 | Ubiquinol-Cytochrome C Reductase Complex Assembly Factor 2 |
| MUCL3 | Mucin Like 3 |
| SERINC5 | Serine Incorporator 5 |
| GPR151 | G Protein-Coupled Receptor 151 |
| XCL2 | X-C Motif Chemokine Ligand 2 |
| MIR181B2 | MicroRNA 181b-2 |
| RORB | RAR Related Orphan Receptor B |
| LASP1 | LIM And SH3 Protein 1 |
| DGKQ | Diacylglycerol Kinase Theta |
| SMPD3 | Sphingomyelin Phosphodiesterase 3 |
| AATF | Apoptosis Antagonizing Transcription Factor |
| IPMK | Inositol Polyphosphate Multikinase |
| EGFL7 | EGF Like Domain Multiple 7 |
| MGAT4B | Alpha-1,3-Mannosyl-Glycoprotein 4-Beta-N-Acetylglucosaminyltransferase B |
| CARD6 | Caspase Recruitment Domain Family Member 6 |
| RETNLB | Resistin Like Beta |
| A3GALT2 | Alpha 1,3-Galactosyltransferase 2 |
| MAPK6 | Mitogen-Activated Protein Kinase 6 |
| PTPRZ1 | Protein Tyrosine Phosphatase Receptor Type Z1 |
| MYO1F | Myosin IF |
| PPP1R8 | Protein Phosphatase 1 Regulatory Subunit 8 |
| RAD1 | RAD1 Checkpoint DNA Exonuclease |
| SCIN | Scinderin |
| NOTUM | Notum, Palmitoleoyl-Protein Carboxylesterase |
| NMUR1 | Neuromedin U Receptor 1 |
| NOVA1 | NOVA Alternative Splicing Regulator 1 |
| GPRC6A | G Protein-Coupled Receptor Class C Group 6 Member A |
| SIRPG | Signal Regulatory Protein Gamma |
| API5 | Apoptosis Inhibitor 5 |
| KRBOX4 | KRAB Box Domain Containing 4 |
| MIR625 | MicroRNA 625 |
| UMLILO | Upstream Master LncRNA Of The Inflammatory Chemokine Locus |
| VRK1 | VRK Serine/Threonine Kinase 1 |
| ITGA9 | Integrin Subunit Alpha 9 |
| CLIC5 | Chloride Intracellular Channel 5 |
| RGS10 | Regulator Of G Protein Signaling 10 |
| SH3BP4 | SH3 Domain Binding Protein 4 |
| GPR83 | G Protein-Coupled Receptor 83 |
| HIC1 | HIC ZBTB Transcriptional Repressor 1 |
| NTN4 | Netrin 4 |
| FKBP3 | FKBP Prolyl Isomerase 3 |
| WLS | Wnt Ligand Secretion Mediator |
| ELL2 | Elongation Factor For RNA Polymerase II 2 |
| GLG1 | Golgi Glycoprotein 1 |
| TCF21 | Transcription Factor 21 |
| TIFAB | TIFA Inhibitor |
| GRB7 | Growth Factor Receptor Bound Protein 7 |
| PABPC1 | Poly(A) Binding Protein Cytoplasmic 1 |
| FBP1 | Fructose-Bisphosphatase 1 |
| PDE8B | Phosphodiesterase 8B |
| CRX | Cone-Rod Homeobox |
| PEX6 | Peroxisomal Biogenesis Factor 6 |
| CNGB1 | Cyclic Nucleotide Gated Channel Subunit Beta 1 |
| PEX3 | Peroxisomal Biogenesis Factor 3 |
| GUCA1A | Guanylate Cyclase Activator 1A |
| COX6B1 | Cytochrome C Oxidase Subunit 6B1 |
| SDHAF2 | Succinate Dehydrogenase Complex Assembly Factor 2 |
| ROBO2 | Roundabout Guidance Receptor 2 |
| AIPL1 | Aryl Hydrocarbon Receptor Interacting Protein Like 1 |
| ARL6 | ADP Ribosylation Factor Like GTPase 6 |
| LHX1 | LIM Homeobox 1 |
| TBX18 | T-Box Transcription Factor 18 |
| PEX13 | Peroxisomal Biogenesis Factor 13 |
| RAB28 | RAB28, Member RAS Oncogene Family |
| KLHL7 | Kelch Like Family Member 7 |
| GUCA1B | Guanylate Cyclase Activator 1B |
| CRTAP | Cartilage Associated Protein |
| COL8A2 | Collagen Type VIII Alpha 2 Chain |
| BBS10 | Bardet-Biedl Syndrome 10 |
| SIX5 | SIX Homeobox 5 |
| FREM2 | FRAS1 Related Extracellular Matrix 2 |
| TTC19 | Tetratricopeptide Repeat Domain 19 |
| WDR60 | WD Repeat Domain 60 |
| BBS7 | Bardet-Biedl Syndrome 7 |
| LCA5 | Lebercilin LCA5 |
| PDZD7 | PDZ Domain Containing 7 |
| BCO1 | Beta-Carotene Oxygenase 1 |
| TMEM138 | Transmembrane Protein 138 |
| ZNF513 | Zinc Finger Protein 513 |
| KIZ | Kizuna Centrosomal Protein |
| NPDC1 | Neural Proliferation, Differentiation And Control 1 |
| PRELID1 | PRELI Domain Containing 1 |
| GNL1 | G Protein Nucleolar 1 (Putative) |
| VPREB3 | V-Set Pre-B Cell Surrogate Light Chain 3 |
| KAAG1 | Kidney Associated Antigen 1 |
| SDHAF4 | Succinate Dehydrogenase Complex Assembly Factor 4 |
| BTN2A3P | Butyrophilin Subfamily 2 Member A3, Pseudogene |
| IGHV1OR21-1 | Immunoglobulin Heavy Variable 1/OR21-1 (Non-Functional) |
| ROBO1 | Roundabout Guidance Receptor 1 |
| GRIN3B | Glutamate Ionotropic Receptor NMDA Type Subunit 3B |
| PRPF40A | Pre-MRNA Processing Factor 40 Homolog A |
| RCL1 | RNA Terminal Phosphate Cyclase Like 1 |
| MDFIC | MyoD Family Inhibitor Domain Containing |
| LAYN | Layilin |
| MEA1 | Male-Enhanced Antigen 1 |
| TASOR | Transcription Activation Suppressor |
| WFDC21P | WAP Four-Disulfide Core Domain 21, Pseudogene |
| TRPC3 | Transient Receptor Potential Cation Channel Subfamily C Member 3 |
| GJB1 | Gap Junction Protein Beta 1 |
| NQO2 | N-Ribosyldihydronicotinamide:Quinone Reductase 2 |
| GRK6 | G Protein-Coupled Receptor Kinase 6 |
| DVL3 | Dishevelled Segment Polarity Protein 3 |
| CPB2 | Carboxypeptidase B2 |
| SFRP4 | Secreted Frizzled Related Protein 4 |
| SLC20A1 | Solute Carrier Family 20 Member 1 |
| RGS2 | Regulator Of G Protein Signaling 2 |
| MAP4K3 | Mitogen-Activated Protein Kinase Kinase Kinase Kinase 3 |
| AQP9 | Aquaporin 9 |
| PLXNA2 | Plexin A2 |
| TKTL1 | Transketolase Like 1 |
| MEP1A | Meprin A Subunit Alpha |
| CYB561 | Cytochrome B561 |
| EDIL3 | EGF Like Repeats And Discoidin Domains 3 |
| HOXA9 | Homeobox A9 |
| ADRM1 | Adhesion Regulating Molecule 1 |
| ACSL6 | Acyl-CoA Synthetase Long Chain Family Member 6 |
| FMOD | Fibromodulin |
| TOX | Thymocyte Selection Associated High Mobility Group Box |
| CWC27 | CWC27 Spliceosome Associated Cyclophilin |
| KLF13 | Kruppel Like Factor 13 |
| SLC39A3 | Solute Carrier Family 39 Member 3 |
| LAPTM5 | Lysosomal Protein Transmembrane 5 |
| MRGPRX1 | MAS Related GPR Family Member X1 |
| ARID4B | AT-Rich Interaction Domain 4B |
| KLHL6 | Kelch Like Family Member 6 |
| LANCL1 | LanC Like 1 |
| CMIP | C-Maf Inducing Protein |
| H1-3 | H1.3 Linker Histone, Cluster Member |
| TNFAIP2 | TNF Alpha Induced Protein 2 |
| GLCE | Glucuronic Acid Epimerase |
| EXTL2 | Exostosin Like Glycosyltransferase 2 |
| ZNF10 | Zinc Finger Protein 10 |
| ZNF746 | Zinc Finger Protein 746 |
| TMEM39A | Transmembrane Protein 39A |
| ALPK2 | Alpha Kinase 2 |
| MAMSTR | MEF2 Activating Motif And SAP Domain Containing Transcriptional Regulator |
| ZNF438 | Zinc Finger Protein 438 |
| ODF3B | Outer Dense Fiber Of Sperm Tails 3B |
| NAT16 | N-Acetyltransferase 16 (Putative) |
| BECN2 | Beclin 2 |
| ZNF767P | Zinc Finger Family Member 767, Pseudogene |
| LOC285626 | Uncharacterized LOC285626 |
| LINC01147 | Long Intergenic Non-Protein Coding RNA 1147 |
| TLR12P | Toll Like Receptor 12, Pseudogene |
| EGILA | EGFR Interacting LncRNA |
| MASCRNA | MALAT1-Associated Small Cytoplasmic RNA |
| ELF2 | E74 Like ETS Transcription Factor 2 |
| PNMT | Phenylethanolamine N-Methyltransferase |
| FABP1 | Fatty Acid Binding Protein 1 |
| SPRY1 | Sprouty RTK Signaling Antagonist 1 |
| BRD1 | Bromodomain Containing 1 |
| HOMER3 | Homer Scaffold Protein 3 |
| CHFR | Checkpoint With Forkhead And Ring Finger Domains |
| BYSL | Bystin Like |
| UTP6 | UTP6 Small Subunit Processome Component |
| PPHLN1 | Periphilin 1 |
| GRIN3A | Glutamate Ionotropic Receptor NMDA Type Subunit 3A |
| RRP9 | Ribosomal RNA Processing 9, U3 Small Nucleolar RNA Binding Protein |
| UBFD1 | Ubiquitin Family Domain Containing 1 |
| SPACA3 | Sperm Acrosome Associated 3 |
| ISX | Intestine Specific Homeobox |
| LGALS14 | Galectin 14 |
| SPANXA1 | Sperm Protein Associated With The Nucleus, X-Linked, Family Member A1 |
| LGALS17A | Galectin 14 Pseudogene |
| USP17L9P | Ubiquitin Specific Peptidase 17 Like Family Member 9, Pseudogene |
| RPL3P2 | Ribosomal Protein L3 Pseudogene 2 |
| GRIA1 | Glutamate Ionotropic Receptor AMPA Type Subunit 1 |
| CSNK1D | Casein Kinase 1 Delta |
| GNAL | G Protein Subunit Alpha L |
| PLPP3 | Phospholipid Phosphatase 3 |
| ZP2 | Zona Pellucida Glycoprotein 2 |
| NISCH | Nischarin |
| SLC29A1 | Solute Carrier Family 29 Member 1 (Augustine Blood Group) |
| NR5A2 | Nuclear Receptor Subfamily 5 Group A Member 2 |
| ANGPTL4 | Angiopoietin Like 4 |
| CITED2 | Cbp/P300 Interacting Transactivator With Glu/Asp Rich Carboxy-Terminal Domain 2 |
| ARHGEF12 | Rho Guanine Nucleotide Exchange Factor 12 |
| SNX3 | Sorting Nexin 3 |
| PTPN14 | Protein Tyrosine Phosphatase Non-Receptor Type 14 |
| KLF5 | Kruppel Like Factor 5 |
| COL11A1 | Collagen Type XI Alpha 1 Chain |
| CPN1 | Carboxypeptidase N Subunit 1 |
| CCNA1 | Cyclin A1 |
| MARK2 | Microtubule Affinity Regulating Kinase 2 |
| MDM4 | MDM4 Regulator Of P53 |
| WWP2 | WW Domain Containing E3 Ubiquitin Protein Ligase 2 |
| PERP | P53 Apoptosis Effector Related To PMP22 |
| OXSR1 | Oxidative Stress Responsive Kinase 1 |
| DLG3 | Discs Large MAGUK Scaffold Protein 3 |
| TPX2 | TPX2 Microtubule Nucleation Factor |
| XPNPEP1 | X-Prolyl Aminopeptidase 1 |
| PDIA4 | Protein Disulfide Isomerase Family A Member 4 |
| JARID2 | Jumonji And AT-Rich Interaction Domain Containing 2 |
| RGS16 | Regulator Of G Protein Signaling 16 |
| DTNBP1 | Dystrobrevin Binding Protein 1 |
| GIT2 | GIT ArfGAP 2 |
| ADD2 | Adducin 2 |
| PDLIM7 | PDZ And LIM Domain 7 |
| DIP2B | Disco Interacting Protein 2 Homolog B |
| SGCB | Sarcoglycan Beta |
| CD320 | CD320 Molecule |
| GALNT1 | Polypeptide N-Acetylgalactosaminyltransferase 1 |
| FKRP | Fukutin Related Protein |
| ST6GALNAC2 | ST6 N-Acetylgalactosaminide Alpha-2,6-Sialyltransferase 2 |
| HEPH | Hephaestin |
| SCARA5 | Scavenger Receptor Class A Member 5 |
| MEP1B | Meprin A Subunit Beta |
| AFF1 | AF4/FMR2 Family Member 1 |
| RAPGEF6 | Rap Guanine Nucleotide Exchange Factor 6 |
| PATZ1 | POZ/BTB And AT Hook Containing Zinc Finger 1 |
| MKRN2 | Makorin Ring Finger Protein 2 |
| ARID3A | AT-Rich Interaction Domain 3A |
| MED20 | Mediator Complex Subunit 20 |
| ZBTB32 | Zinc Finger And BTB Domain Containing 32 |
| TAF7 | TATA-Box Binding Protein Associated Factor 7 |
| ADGRF5 | Adhesion G Protein-Coupled Receptor F5 |
| WDR37 | WD Repeat Domain 37 |
| SNX20 | Sorting Nexin 20 |
| BBX | BBX High Mobility Group Box Domain Containing |
| LRRC8D | Leucine Rich Repeat Containing 8 VRAC Subunit D |
| ATG16L2 | Autophagy Related 16 Like 2 |
| ARHGAP30 | Rho GTPase Activating Protein 30 |
| TUSC2 | Tumor Suppressor 2, Mitochondrial Calcium Regulator |
| PRDM13 | PR/SET Domain 13 |
| CA12 | Carbonic Anhydrase 12 |
| LPAR1 | Lysophosphatidic Acid Receptor 1 |
| HSF2 | Heat Shock Transcription Factor 2 |
| EIF5 | Eukaryotic Translation Initiation Factor 5 |
| COIL | Coilin |
| HS2ST1 | Heparan Sulfate 2-O-Sulfotransferase 1 |
| DHX16 | DEAH-Box Helicase 16 |
| EMG1 | EMG1 N1-Specific Pseudouridine Methyltransferase |
| EPS15L1 | Epidermal Growth Factor Receptor Pathway Substrate 15 Like 1 |
| NR2C1 | Nuclear Receptor Subfamily 2 Group C Member 1 |
| DDX10 | DEAD-Box Helicase 10 |
| SYNJ2BP | Synaptojanin 2 Binding Protein |
| DBP | D-Box Binding PAR BZIP Transcription Factor |
| ASMT | Acetylserotonin O-Methyltransferase |
| NPY4R | Neuropeptide Y Receptor Y4 |
| FUT5 | Fucosyltransferase 5 |
| EXOSC7 | Exosome Component 7 |
| WDR46 | WD Repeat Domain 46 |
| VGLL4 | Vestigial Like Family Member 4 |
| WDR74 | WD Repeat Domain 74 |
| UTP14A | UTP14A Small Subunit Processome Component |
| TOR1AIP2 | Torsin 1A Interacting Protein 2 |
| RRP36 | Ribosomal RNA Processing 36 |
| TNFAIP8L1 | TNF Alpha Induced Protein 8 Like 1 |
| SEPTIN11 | Septin 11 |
| MARCHF2 | Membrane Associated Ring-CH-Type Finger 2 |
| MEAK7 | MTOR Associated Protein, Eak-7 Homolog |
| LGALS16 | Galectin 16 |
| MIR506 | MicroRNA 506 |
| SAA3P | Serum Amyloid A3, Pseudogene |
| ERVK-18 | Endogenous Retrovirus Group K Member 18 |
| CLCP2 | Charcot-Leyden Crystal Protein Pseudogene 2 |
| ERVE-4 | Endogenous Retrovirus Group E Member 4 |
| LOC100290036 | Immunglobulin Heavy Chain Variable Region |
| EGR2 | Early Growth Response 2 |
| PGAM2 | Phosphoglycerate Mutase 2 |
| CLDN14 | Claudin 14 |
| PLA2G4D | Phospholipase A2 Group IVD |
| CLDN25 | Claudin 25 |
| NASP | Nuclear Autoantigenic Sperm Protein |
| NLRP13 | NLR Family Pyrin Domain Containing 13 |
| EPHA7 | EPH Receptor A7 |
| EPHA5 | EPH Receptor A5 |
| MAPK4 | Mitogen-Activated Protein Kinase 4 |
| PTK7 | Protein Tyrosine Kinase 7 (Inactive) |
| CSNK1G1 | Casein Kinase 1 Gamma 1 |
| RYK | Receptor Like Tyrosine Kinase |
| FGF13 | Fibroblast Growth Factor 13 |
| CSNK1G3 | Casein Kinase 1 Gamma 3 |
| SEMA4G | Semaphorin 4G |
| LMTK2 | Lemur Tyrosine Kinase 2 |
| ACO1 | Aconitase 1 |
| HAL | Histidine Ammonia-Lyase |
| GSTA1 | Glutathione S-Transferase Alpha 1 |
| MNAT1 | MNAT1 Component Of CDK Activating Kinase |
| GSTA2 | Glutathione S-Transferase Alpha 2 |
| EFNB1 | Ephrin B1 |
| DCC | DCC Netrin 1 Receptor |
| RRAS2 | RAS Related 2 |
| DOT1L | DOT1 Like Histone Lysine Methyltransferase |
| LSS | Lanosterol Synthase |
| TRPV5 | Transient Receptor Potential Cation Channel Subfamily V Member 5 |
| EFEMP1 | EGF Containing Fibulin Extracellular Matrix Protein 1 |
| ADCYAP1R1 | ADCYAP Receptor Type I |
| USP1 | Ubiquitin Specific Peptidase 1 |
| NPY2R | Neuropeptide Y Receptor Y2 |
| ITPKB | Inositol-Trisphosphate 3-Kinase B |
| TLL1 | Tolloid Like 1 |
| RGS4 | Regulator Of G Protein Signaling 4 |
| ARHGAP1 | Rho GTPase Activating Protein 1 |
| TP53BP2 | Tumor Protein P53 Binding Protein 2 |
| PTTG1 | PTTG1 Regulator Of Sister Chromatid Separation, Securin |
| COX15 | Cytochrome C Oxidase Assembly Homolog COX15 |
| PTP4A2 | Protein Tyrosine Phosphatase 4A2 |
| NDUFS4 | NADH:Ubiquinone Oxidoreductase Subunit S4 |
| MPZL1 | Myelin Protein Zero Like 1 |
| SGCA | Sarcoglycan Alpha |
| BIRC6 | Baculoviral IAP Repeat Containing 6 |
| ASPSCR1 | ASPSCR1 Tether For SLC2A4, UBX Domain Containing |
| USP22 | Ubiquitin Specific Peptidase 22 |
| MCAT | Malonyl-CoA-Acyl Carrier Protein Transacylase |
| USP11 | Ubiquitin Specific Peptidase 11 |
| SUZ12 | SUZ12 Polycomb Repressive Complex 2 Subunit |
| KDM5A | Lysine Demethylase 5A |
| PITX3 | Paired Like Homeodomain 3 |
| HOXA7 | Homeobox A7 |
| MTMR4 | Myotubularin Related Protein 4 |
| GLIS2 | GLIS Family Zinc Finger 2 |
| ARHGAP15 | Rho GTPase Activating Protein 15 |
| ACAP1 | ArfGAP With Coiled-Coil, Ankyrin Repeat And PH Domains 1 |
| ZNF148 | Zinc Finger Protein 148 |
| POLM | DNA Polymerase Mu |
| PARG | Poly(ADP-Ribose) Glycohydrolase |
| CTNNBL1 | Catenin Beta Like 1 |
| HPS3 | HPS3 Biogenesis Of Lysosomal Organelles Complex 2 Subunit 1 |
| FAIM | Fas Apoptotic Inhibitory Molecule |
| C1QTNF3 | C1q And TNF Related 3 |
| TP53INP1 | Tumor Protein P53 Inducible Nuclear Protein 1 |
| RAB17 | RAB17, Member RAS Oncogene Family |
| PPT2 | Palmitoyl-Protein Thioesterase 2 |
| SOX13 | SRY-Box Transcription Factor 13 |
| PBX2 | PBX Homeobox 2 |
| CERS6 | Ceramide Synthase 6 |
| PNLIPRP2 | Pancreatic Lipase Related Protein 2 (Gene/Pseudogene) |
| PLEK2 | Pleckstrin 2 |
| SH3PXD2A | SH3 And PX Domains 2A |
| KLHDC2 | Kelch Domain Containing 2 |
| RHOBTB3 | Rho Related BTB Domain Containing 3 |
| COPS8 | COP9 Signalosome Subunit 8 |
| GPR34 | G Protein-Coupled Receptor 34 |
| MED28 | Mediator Complex Subunit 28 |
| USP29 | Ubiquitin Specific Peptidase 29 |
| PGGT1B | Protein Geranylgeranyltransferase Type I Subunit Beta |
| SRXN1 | Sulfiredoxin 1 |
| TEX101 | Testis Expressed 101 |
| CROCC | Ciliary Rootlet Coiled-Coil, Rootletin |
| DEDD | Death Effector Domain Containing |
| CHORDC1 | Cysteine And Histidine Rich Domain Containing 1 |
| PODXL2 | Podocalyxin Like 2 |
| TMEM181 | Transmembrane Protein 181 |
| GUCY1B1 | Guanylate Cyclase 1 Soluble Subunit Beta 1 |
| TECPR1 | Tectonin Beta-Propeller Repeat Containing 1 |
| CCN3 | Cellular Communication Network Factor 3 |
| LMBR1L | Limb Development Membrane Protein 1 Like |
| PLAAT3 | Phospholipase A And Acyltransferase 3 |
| RIOX2 | Ribosomal Oxygenase 2 |
| ABTB2 | Ankyrin Repeat And BTB Domain Containing 2 |
| PIK3R6 | Phosphoinositide-3-Kinase Regulatory Subunit 6 |
| TMEM258 | Transmembrane Protein 258 |
| PCED1A | PC-Esterase Domain Containing 1A |
| H2AZ2 | H2A.Z Variant Histone 2 |
| GIMAP1-GIMAP5 | GIMAP1-GIMAP5 Readthrough |
| SLC6A12 | Solute Carrier Family 6 Member 12 |
| SECISBP2 | SECIS Binding Protein 2 |
| CLCN4 | Chloride Voltage-Gated Channel 4 |
| SMUG1 | Single-Strand-Selective Monofunctional Uracil-DNA Glycosylase 1 |
| NMB | Neuromedin B |
| CDK20 | Cyclin Dependent Kinase 20 |
| SMCHD1 | Structural Maintenance Of Chromosomes Flexible Hinge Domain Containing 1 |
| CDK12 | Cyclin Dependent Kinase 12 |
| NPTN | Neuroplastin |
| SAP18 | Sin3A Associated Protein 18 |
| GAPVD1 | GTPase Activating Protein And VPS9 Domains 1 |
| LYPLA2 | Lysophospholipase 2 |
| ANGPTL7 | Angiopoietin Like 7 |
| EPB41L2 | Erythrocyte Membrane Protein Band 4.1 Like 2 |
| ATAD2 | ATPase Family AAA Domain Containing 2 |
| TOR1AIP1 | Torsin 1A Interacting Protein 1 |
| PHIP | Pleckstrin Homology Domain Interacting Protein |
| SUN1 | Sad1 And UNC84 Domain Containing 1 |
| DTL | Denticleless E3 Ubiquitin Protein Ligase Homolog |
| KHDRBS3 | KH RNA Binding Domain Containing, Signal Transduction Associated 3 |
| PPP1R14C | Protein Phosphatase 1 Regulatory Inhibitor Subunit 14C |
| HMG20A | High Mobility Group 20A |
| MPRIP | Myosin Phosphatase Rho Interacting Protein |
| DEDD2 | Death Effector Domain Containing 2 |
| FHOD1 | Formin Homology 2 Domain Containing 1 |
| ERGIC3 | ERGIC And Golgi 3 |
| YARS1 | Tyrosyl-TRNA Synthetase 1 |
| USP54 | Ubiquitin Specific Peptidase 54 |
| PAK1IP1 | PAK1 Interacting Protein 1 |
| RBM19 | RNA Binding Motif Protein 19 |
| DCAF13 | DDB1 And CUL4 Associated Factor 13 |
| BRIX1 | Biogenesis Of Ribosomes BRX1 |
| SPATS2 | Spermatogenesis Associated Serine Rich 2 |
| RABL6 | RAB, Member RAS Oncogene Family Like 6 |
| PCNP | PEST Proteolytic Signal Containing Nuclear Protein |
| NIFK | Nucleolar Protein Interacting With The FHA Domain Of MKI67 |
| DNTTIP2 | Deoxynucleotidyltransferase Terminal Interacting Protein 2 |
| NOL10 | Nucleolar Protein 10 |
| MAGEA10 | MAGE Family Member A10 |
| WDR43 | WD Repeat Domain 43 |
| NOL9 | Nucleolar Protein 9 |
| ZBTB12 | Zinc Finger And BTB Domain Containing 12 |
| KLF14 | Kruppel Like Factor 14 |
| UTP20 | UTP20 Small Subunit Processome Component |
| SIRPB2 | Signal Regulatory Protein Beta 2 |
| SPRR2F | Small Proline Rich Protein 2F |
| DCAF15 | DDB1 And CUL4 Associated Factor 15 |
| UTP11 | UTP11 Small Subunit Processome Component |
| MIR204 | MicroRNA 204 |
| MIR448 | MicroRNA 448 |
| GVINP2 | GTPase, Very Large Interferon Inducible Pseudogene 2 |
| SLC12A6 | Solute Carrier Family 12 Member 6 |
| GRM6 | Glutamate Metabotropic Receptor 6 |
| GNAT1 | G Protein Subunit Alpha Transducin 1 |
| PDE6A | Phosphodiesterase 6A |
| CNGA1 | Cyclic Nucleotide Gated Channel Subunit Alpha 1 |
| UROD | Uroporphyrinogen Decarboxylase |
| PEX1 | Peroxisomal Biogenesis Factor 1 |
| EHHADH | Enoyl-CoA Hydratase And 3-Hydroxyacyl CoA Dehydrogenase |
| DLX5 | Distal-Less Homeobox 5 |
| ACSL4 | Acyl-CoA Synthetase Long Chain Family Member 4 |
| CACNG2 | Calcium Voltage-Gated Channel Auxiliary Subunit Gamma 2 |
| XPNPEP2 | X-Prolyl Aminopeptidase 2 |
| PDE6D | Phosphodiesterase 6D |
| PDE6G | Phosphodiesterase 6G |
| SLC24A1 | Solute Carrier Family 24 Member 1 |
| GRK1 | G Protein-Coupled Receptor Kinase 1 |
| DHDDS | Dehydrodolichyl Diphosphate Synthase Subunit |
| BEST1 | Bestrophin 1 |
| MAP3K6 | Mitogen-Activated Protein Kinase Kinase Kinase 6 |
| CACNA2D4 | Calcium Voltage-Gated Channel Auxiliary Subunit Alpha2delta 4 |
| PPM1K | Protein Phosphatase, Mg2+/Mn2+ Dependent 1K |
| ITGA8 | Integrin Subunit Alpha 8 |
| RGR | Retinal G Protein Coupled Receptor |
| CYP4V2 | Cytochrome P450 Family 4 Subfamily V Member 2 |
| SLC7A14 | Solute Carrier Family 7 Member 14 |
| PUS1 | Pseudouridine Synthase 1 |
| AGGF1 | Angiogenic Factor With G-Patch And FHA Domains 1 |
| PEX11B | Peroxisomal Biogenesis Factor 11 Beta |
| PORCN | Porcupine O-Acyltransferase |
| IMPAD1 | Inositol Monophosphatase Domain Containing 1 |
| CIB2 | Calcium And Integrin Binding Family Member 2 |
| FGD1 | FYVE, RhoGEF And PH Domain Containing 1 |
| ADAMTSL2 | ADAMTS Like 2 |
| P3H1 | Prolyl 3-Hydroxylase 1 |
| PEX12 | Peroxisomal Biogenesis Factor 12 |
| PITPNM3 | PITPNM Family Member 3 |
| CERKL | Ceramide Kinase Like |
| CDHR1 | Cadherin Related Family Member 1 |
| FSCN2 | Fascin Actin-Bundling Protein 2, Retinal |
| AMMECR1 | AMMECR Nuclear Protein 1 |
| AAGAB | Alpha And Gamma Adaptin Binding Protein |
| SLC25A38 | Solute Carrier Family 25 Member 38 |
| LMF1 | Lipase Maturation Factor 1 |
| RD3 | Retinal Degeneration 3, GUCY2D Regulator |
| PEX16 | Peroxisomal Biogenesis Factor 16 |
| IMPG2 | Interphotoreceptor Matrix Proteoglycan 2 |
| COX20 | Cytochrome C Oxidase Assembly Factor COX20 |
| LRIT3 | Leucine Rich Repeat, Ig-Like And Transmembrane Domains 3 |
| CABP4 | Calcium Binding Protein 4 |
| TRAPPC2 | Trafficking Protein Particle Complex 2 |
| VSX1 | Visual System Homeobox 1 |
| SLITRK6 | SLIT And NTRK Like Family Member 6 |
| NYX | Nyctalopin |
| LYRM7 | LYR Motif Containing 7 |
| COX14 | Cytochrome C Oxidase Assembly Factor COX14 |
| CLRN1 | Clarin 1 |
| HMX1 | H6 Family Homeobox 1 |
| GPR179 | G Protein-Coupled Receptor 179 |
| ADGRV1 | Adhesion G Protein-Coupled Receptor V1 |
| CEP19 | Centrosomal Protein 19 |
| MFRP | Membrane Frizzled-Related Protein |
| STN1 | STN1 Subunit Of CST Complex |
| OPN1MW | Opsin 1, Medium Wave Sensitive |
| BBIP1 | BBSome Interacting Protein 1 |
| KCNE5 | Potassium Voltage-Gated Channel Subfamily E Regulatory Subunit 5 |
| PET100 | PET100 Cytochrome C Oxidase Chaperone |
| PRCD | Photoreceptor Disc Component |
| MAGEA4 | MAGE Family Member A4 |
| REG1B | Regenerating Family Member 1 Beta |
| YTHDF1 | YTH N6-Methyladenosine RNA Binding Protein 1 |
| PPP1R14B | Protein Phosphatase 1 Regulatory Inhibitor Subunit 14B |
| ADSS1 | Adenylosuccinate Synthase 1 |
| BTNL3 | Butyrophilin Like 3 |
| TMEM106A | Transmembrane Protein 106A |
| ROMO1 | Reactive Oxygen Species Modulator 1 |
| BTNL10 | Butyrophilin Like 10 |
| IGHV4OR15-8 | Immunoglobulin Heavy Variable 4/OR15-8 (Non-Functional) |
| IGHV3OR16-10 | Immunoglobulin Heavy Variable 3/OR16-10 (Non-Functional) |
| CCL15-CCL14 | CCL15-CCL14 Readthrough (NMD Candidate) |
| IGHV1OR15-9 | Immunoglobulin Heavy Variable 1/OR15-9 (Non-Functional) |
| IGHV2OR16-5 | Immunoglobulin Heavy Variable 2/OR16-5 (Non-Functional) |
| IGKV3OR2-268 | Immunoglobulin Kappa Variable 3/OR2-268 (Non-Functional) |
| IGHV3OR16-8 | Immunoglobulin Heavy Variable 3/OR16-8 (Non-Functional) |
| TRGJ2 | T Cell Receptor Gamma Joining 2 |
| ENSG00000239920 |  |
| IGHV3OR15-7 | Immunoglobulin Heavy Variable 3/OR15-7 (Pseudogene) |
| IGHV3OR16-12 | Immunoglobulin Heavy Variable 3/OR16-12 (Non-Functional) |
| IGHV3OR16-13 | Immunoglobulin Heavy Variable 3/OR16-13 (Non-Functional) |
| IGHV3OR16-9 | Immunoglobulin Heavy Variable 3/OR16-9 (Non-Functional) |
| ENSG00000259680 |  |
| ENSG00000281179 |  |
| ENSG00000278263 |  |
| EEF2K | Eukaryotic Elongation Factor 2 Kinase |
| SNCG | Synuclein Gamma |
| PNPT1 | Polyribonucleotide Nucleotidyltransferase 1 |
| EIF2AK1 | Eukaryotic Translation Initiation Factor 2 Alpha Kinase 1 |
| KCNK2 | Potassium Two Pore Domain Channel Subfamily K Member 2 |
| PSD | Pleckstrin And Sec7 Domain Containing |
| ECH1 | Enoyl-CoA Hydratase 1 |
| DDX18 | DEAD-Box Helicase 18 |
| AADAT | Aminoadipate Aminotransferase |
| TBL3 | Transducin Beta Like 3 |
| SVIL | Supervillin |
| CLIC6 | Chloride Intracellular Channel 6 |
| GPR4 | G Protein-Coupled Receptor 4 |
| EXOSC8 | Exosome Component 8 |
| ZNF24 | Zinc Finger Protein 24 |
| POP4 | POP4 Homolog, Ribonuclease P/MRP Subunit |
| QTRT1 | Queuine TRNA-Ribosyltransferase Catalytic Subunit 1 |
| HBP1 | HMG-Box Transcription Factor 1 |
| NVL | Nuclear VCP Like |
| ADAM2 | ADAM Metallopeptidase Domain 2 |
| XAB2 | XPA Binding Protein 2 |
| OSGIN1 | Oxidative Stress Induced Growth Inhibitor 1 |
| CERS5 | Ceramide Synthase 5 |
| WDR12 | WD Repeat Domain 12 |
| SNX15 | Sorting Nexin 15 |
| PPIL4 | Peptidylprolyl Isomerase Like 4 |
| REXO4 | REX4 Homolog, 3'-5' Exonuclease |
| CRNKL1 | Crooked Neck Pre-MRNA Splicing Factor 1 |
| PPP4R2 | Protein Phosphatase 4 Regulatory Subunit 2 |
| PWP2 | PWP2 Small Subunit Processome Component |
| PRPF38A | Pre-MRNA Processing Factor 38A |
| GLYR1 | Glyoxylate Reductase 1 Homolog |
| TXNDC9 | Thioredoxin Domain Containing 9 |
| UTP15 | UTP15 Small Subunit Processome Component |
| SNRNP27 | Small Nuclear Ribonucleoprotein U4/U6.U5 Subunit 27 |
| DDX51 | DEAD-Box Helicase 51 |
| ELP5 | Elongator Acetyltransferase Complex Subunit 5 |
| EXOSC6 | Exosome Component 6 |
| TIPRL | TOR Signaling Pathway Regulator |
| TNFAIP8L3 | TNF Alpha Induced Protein 8 Like 3 |
| TM4SF5 | Transmembrane 4 L Six Family Member 5 |
| WDR75 | WD Repeat Domain 75 |
| UTP3 | UTP3 Small Subunit Processome Component |
| IMP4 | IMP U3 Small Nucleolar Ribonucleoprotein 4 |
| MAK16 | MAK16 Homolog |
| CEP85L | Centrosomal Protein 85 Like |
| RPF1 | Ribosome Production Factor 1 Homolog |
| NOL11 | Nucleolar Protein 11 |
| KIAA1586 | KIAA1586 |
| URB2 | URB2 Ribosome Biogenesis Homolog |
| NLGN4Y | Neuroligin 4 Y-Linked |
| TEX10 | Testis Expressed 10 |
| EHBP1L1 | EH Domain Binding Protein 1 Like 1 |
| CENPV | Centromere Protein V |
| ESF1 | ESF1 Nucleolar Pre-RRNA Processing Protein Homolog |
| ZNF574 | Zinc Finger Protein 574 |
| PNMA3 | PNMA Family Member 3 |
| RGPD8 | RANBP2 Like And GRIP Domain Containing 8 |
| CEP162 | Centrosomal Protein 162 |
| SLFN12L | Schlafen Family Member 12 Like |
| SEPTIN1 | Septin 1 |
| MARCHF6 | Membrane Associated Ring-CH-Type Finger 6 |
| RGPD3 | RANBP2 Like And GRIP Domain Containing 3 |
| MARCHF4 | Membrane Associated Ring-CH-Type Finger 4 |
| PPAN-P2RY11 | PPAN-P2RY11 Readthrough |
| AHSA2P | Activator Of HSP90 ATPase Homolog 2, Pseudogene |
| LUZP6 | Leucine Zipper Protein 6 |
| MIR409 | MicroRNA 409 |
| CDB2 | Corneal Dystrophy Of Bowman Layer Type II (Thiel-Behnke) |
| RPS13 | Ribosomal Protein S13 |
| ITIH3 | Inter-Alpha-Trypsin Inhibitor Heavy Chain 3 |
| APOC1 | Apolipoprotein C1 |
| SIRT3 | Sirtuin 3 |
| MSX2 | Msh Homeobox 2 |
| MAT1A | Methionine Adenosyltransferase 1A |
| NDST1 | N-Deacetylase And N-Sulfotransferase 1 |
| E2F4 | E2F Transcription Factor 4 |
| GNMT | Glycine N-Methyltransferase |
| GATM | Glycine Amidinotransferase |
| AVPR1A | Arginine Vasopressin Receptor 1A |
| SLC9A6 | Solute Carrier Family 9 Member A6 |
| SLC4A4 | Solute Carrier Family 4 Member 4 |
| SLC6A8 | Solute Carrier Family 6 Member 8 |
| FHIT | Fragile Histidine Triad Diadenosine Triphosphatase |
| ACOX1 | Acyl-CoA Oxidase 1 |
| PAX7 | Paired Box 7 |
| STK24 | Serine/Threonine Kinase 24 |
| SLC16A3 | Solute Carrier Family 16 Member 3 |
| SKIL | SKI Like Proto-Oncogene |
| DKK2 | Dickkopf WNT Signaling Pathway Inhibitor 2 |
| CDC7 | Cell Division Cycle 7 |
| LPAR2 | Lysophosphatidic Acid Receptor 2 |
| PRMT7 | Protein Arginine Methyltransferase 7 |
| SLC31A1 | Solute Carrier Family 31 Member 1 |
| GPX7 | Glutathione Peroxidase 7 |
| DFFB | DNA Fragmentation Factor Subunit Beta |
| SGK3 | Serum/Glucocorticoid Regulated Kinase Family Member 3 |
| ADAMTS18 | ADAM Metallopeptidase With Thrombospondin Type 1 Motif 18 |
| CALCRL | Calcitonin Receptor Like Receptor |
| ACP2 | Acid Phosphatase 2, Lysosomal |
| XPR1 | Xenotropic And Polytropic Retrovirus Receptor 1 |
| PANK1 | Pantothenate Kinase 1 |
| KIF1C | Kinesin Family Member 1C |
| IDH3G | Isocitrate Dehydrogenase (NAD(+)) 3 Non-Catalytic Subunit Gamma |
| HHIP | Hedgehog Interacting Protein |
| NEK7 | NIMA Related Kinase 7 |
| GATAD2B | GATA Zinc Finger Domain Containing 2B |
| CELSR1 | Cadherin EGF LAG Seven-Pass G-Type Receptor 1 |
| CCM2 | CCM2 Scaffold Protein |
| LUM | Lumican |
| CA6 | Carbonic Anhydrase 6 |
| CACNA2D2 | Calcium Voltage-Gated Channel Auxiliary Subunit Alpha2delta 2 |
| TWIST2 | Twist Family BHLH Transcription Factor 2 |
| UGT8 | UDP Glycosyltransferase 8 |
| EDC3 | Enhancer Of MRNA Decapping 3 |
| GPRC5A | G Protein-Coupled Receptor Class C Group 5 Member A |
| CDYL | Chromodomain Y Like |
| MFAP2 | Microfibril Associated Protein 2 |
| SLC9A2 | Solute Carrier Family 9 Member A2 |
| RASSF2 | Ras Association Domain Family Member 2 |
| TAF4 | TATA-Box Binding Protein Associated Factor 4 |
| PTPN9 | Protein Tyrosine Phosphatase Non-Receptor Type 9 |
| GSTK1 | Glutathione S-Transferase Kappa 1 |
| CLIC4 | Chloride Intracellular Channel 4 |
| CNOT4 | CCR4-NOT Transcription Complex Subunit 4 |
| SGMS1 | Sphingomyelin Synthase 1 |
| CHST11 | Carbohydrate Sulfotransferase 11 |
| GPR17 | G Protein-Coupled Receptor 17 |
| MAST2 | Microtubule Associated Serine/Threonine Kinase 2 |
| BOC | BOC Cell Adhesion Associated, Oncogene Regulated |
| LRRC4C | Leucine Rich Repeat Containing 4C |
| MAFG | MAF BZIP Transcription Factor G |
| ARHGAP17 | Rho GTPase Activating Protein 17 |
| ARHGEF4 | Rho Guanine Nucleotide Exchange Factor 4 |
| KLF3 | Kruppel Like Factor 3 |
| ULK3 | Unc-51 Like Kinase 3 |
| ULK4 | Unc-51 Like Kinase 4 |
| USP28 | Ubiquitin Specific Peptidase 28 |
| PPP1R15A | Protein Phosphatase 1 Regulatory Subunit 15A |
| RNF139 | Ring Finger Protein 139 |
| NPAS3 | Neuronal PAS Domain Protein 3 |
| SLC43A2 | Solute Carrier Family 43 Member 2 |
| SRPX | Sushi Repeat Containing Protein X-Linked |
| SERPINI2 | Serpin Family I Member 2 |
| SHPRH | SNF2 Histone Linker PHD RING Helicase |
| RFK | Riboflavin Kinase |
| CUEDC2 | CUE Domain Containing 2 |
| ELK3 | ETS Transcription Factor ELK3 |
| CRIM1 | Cysteine Rich Transmembrane BMP Regulator 1 |
| DTX2 | Deltex E3 Ubiquitin Ligase 2 |
| MPP5 | Membrane Palmitoylated Protein 5 |
| NUS1 | NUS1 Dehydrodolichyl Diphosphate Synthase Subunit |
| GATA5 | GATA Binding Protein 5 |
| ABCC10 | ATP Binding Cassette Subfamily C Member 10 |
| BIK | BCL2 Interacting Killer |
| TNS3 | Tensin 3 |
| USP45 | Ubiquitin Specific Peptidase 45 |
| SLC9A8 | Solute Carrier Family 9 Member A8 |
| TFAP4 | Transcription Factor AP-4 |
| TIGAR | TP53 Induced Glycolysis Regulatory Phosphatase |
| PITPNM1 | Phosphatidylinositol Transfer Protein Membrane Associated 1 |
| PLEKHA7 | Pleckstrin Homology Domain Containing A7 |
| PPFIBP2 | PPFIA Binding Protein 2 |
| ST3GAL2 | ST3 Beta-Galactoside Alpha-2,3-Sialyltransferase 2 |
| RHBDF1 | Rhomboid 5 Homolog 1 |
| SCMH1 | Scm Polycomb Group Protein Homolog 1 |
| MXD1 | MAX Dimerization Protein 1 |
| CDKL2 | Cyclin Dependent Kinase Like 2 |
| GJA10 | Gap Junction Protein Alpha 10 |
| AIG1 | Androgen Induced 1 |
| CAPN11 | Calpain 11 |
| ADAMTS9 | ADAM Metallopeptidase With Thrombospondin Type 1 Motif 9 |
| KIAA1549 | KIAA1549 |
| TPCN2 | Two Pore Segment Channel 2 |
| TOB2 | Transducer Of ERBB2, 2 |
| ZFX | Zinc Finger Protein X-Linked |
| USP47 | Ubiquitin Specific Peptidase 47 |
| PTCRA | Pre T Cell Antigen Receptor Alpha |
| RAB21 | RAB21, Member RAS Oncogene Family |
| MMP28 | Matrix Metallopeptidase 28 |
| DIDO1 | Death Inducer-Obliterator 1 |
| RPL38 | Ribosomal Protein L38 |
| CBX6 | Chromobox 6 |
| AKAP11 | A-Kinase Anchoring Protein 11 |
| LRFN2 | Leucine Rich Repeat And Fibronectin Type III Domain Containing 2 |
| RNF146 | Ring Finger Protein 146 |
| PRAP1 | Proline Rich Acidic Protein 1 |
| PSTPIP2 | Proline-Serine-Threonine Phosphatase Interacting Protein 2 |
| KLHL14 | Kelch Like Family Member 14 |
| FNIP1 | Folliculin Interacting Protein 1 |
| CIDEB | Cell Death Inducing DFFA Like Effector B |
| CMTM7 | CKLF Like MARVEL Transmembrane Domain Containing 7 |
| NEIL3 | Nei Like DNA Glycosylase 3 |
| CRLF3 | Cytokine Receptor Like Factor 3 |
| HOXD3 | Homeobox D3 |
| DMTF1 | Cyclin D Binding Myb Like Transcription Factor 1 |
| DMXL2 | Dmx Like 2 |
| CD99L2 | CD99 Molecule Like 2 |
| MFSD1 | Major Facilitator Superfamily Domain Containing 1 |
| ADAMTS19 | ADAM Metallopeptidase With Thrombospondin Type 1 Motif 19 |
| AFAP1L2 | Actin Filament Associated Protein 1 Like 2 |
| ARHGAP18 | Rho GTPase Activating Protein 18 |
| ARHGAP21 | Rho GTPase Activating Protein 21 |
| ANKRD2 | Ankyrin Repeat Domain 2 |
| TLX3 | T Cell Leukemia Homeobox 3 |
| TSPAN33 | Tetraspanin 33 |
| RAPH1 | Ras Association (RalGDS/AF-6) And Pleckstrin Homology Domains 1 |
| PRDM4 | PR/SET Domain 4 |
| NFATC2IP | Nuclear Factor Of Activated T Cells 2 Interacting Protein |
| CPNE5 | Copine 5 |
| DNAJC11 | DnaJ Heat Shock Protein Family (Hsp40) Member C11 |
| OCIAD1 | OCIA Domain Containing 1 |
| CASC4 | Cancer Susceptibility 4 |
| KRT76 | Keratin 76 |
| TRAM2 | Translocation Associated Membrane Protein 2 |
| TRIM58 | Tripartite Motif Containing 58 |
| POPDC3 | Popeye Domain Containing 3 |
| RAB20 | RAB20, Member RAS Oncogene Family |
| ITFG2 | Integrin Alpha FG-GAP Repeat Containing 2 |
| PCGF3 | Polycomb Group Ring Finger 3 |
| PCOLCE2 | Procollagen C-Endopeptidase Enhancer 2 |
| SHCBP1 | SHC Binding And Spindle Associated 1 |
| TESPA1 | Thymocyte Expressed, Positive Selection Associated 1 |
| DACT2 | Dishevelled Binding Antagonist Of Beta Catenin 2 |
| MRFAP1 | Morf4 Family Associated Protein 1 |
| DENND5B | DENN Domain Containing 5B |
| GON4L | Gon-4 Like |
| NIPAL3 | NIPA Like Domain Containing 3 |
| FOXJ3 | Forkhead Box J3 |
| AKIP1 | A-Kinase Interacting Protein 1 |
| BLOC1S5 | Biogenesis Of Lysosomal Organelles Complex 1 Subunit 5 |
| APOC4 | Apolipoprotein C4 |
| KDM7A | Lysine Demethylase 7A |
| CGRRF1 | Cell Growth Regulator With Ring Finger Domain 1 |
| ELOVL3 | ELOVL Fatty Acid Elongase 3 |
| GALNT18 | Polypeptide N-Acetylgalactosaminyltransferase 18 |
| LRRC3 | Leucine Rich Repeat Containing 3 |
| TMEM14A | Transmembrane Protein 14A |
| TCERG1L | Transcription Elongation Regulator 1 Like |
| TMEM163 | Transmembrane Protein 163 |
| KMT5B | Lysine Methyltransferase 5B |
| KNL1 | Kinetochore Scaffold 1 |
| MMS22L | MMS22 Like, DNA Repair Protein |
| FOXQ1 | Forkhead Box Q1 |
| ZG16 | Zymogen Granule Protein 16 |
| SERTAD2 | SERTA Domain Containing 2 |
| RADIL | Rap Associating With DIL Domain |
| IFIT1B | Interferon Induced Protein With Tetratricopeptide Repeats 1B |
| CRIP3 | Cysteine Rich Protein 3 |
| ANXA8L1 | Annexin A8 Like 1 |
| TBC1D32 | TBC1 Domain Family Member 32 |
| RIC1 | RIC1 Homolog, RAB6A GEF Complex Partner 1 |
| FAM47E | Family With Sequence Similarity 47 Member E |
| OCSTAMP | Osteoclast Stimulatory Transmembrane Protein |
| ZSWIM1 | Zinc Finger SWIM-Type Containing 1 |
| SPINK14 | Serine Peptidase Inhibitor Kazal Type 14 (Putative) |
| CPLANE2 | Ciliogenesis And Planar Polarity Effector 2 |
| ANKHD1-EIF4EBP3 | ANKHD1-EIF4EBP3 Readthrough |
| IRX2 | Iroquois Homeobox 2 |
| BAGE | B Melanoma Antigen |
| TTF2 | Transcription Termination Factor 2 |
| MCRS1 | Microspherule Protein 1 |
| CSMD1 | CUB And Sushi Multiple Domains 1 |
| FIBCD1 | Fibrinogen C Domain Containing 1 |
| RPL19 | Ribosomal Protein L19 |
| NPPC | Natriuretic Peptide C |
| LRRC23 | Leucine Rich Repeat Containing 23 |
| CARD16 | Caspase Recruitment Domain Family Member 16 |
| DDX5 | DEAD-Box Helicase 5 |
| CPA6 | Carboxypeptidase A6 |
| HLX | H2.0 Like Homeobox |
| RPL23A | Ribosomal Protein L23a |
| MIR499A | MicroRNA 499a |
| FUS | FUS RNA Binding Protein |
| CEACAM4 | CEA Cell Adhesion Molecule 4 |
| EXT2 | Exostosin Glycosyltransferase 2 |
| ACACB | Acetyl-CoA Carboxylase Beta |
| STRADA | STE20 Related Adaptor Alpha |
| KHK | Ketohexokinase |
| SLK | STE20 Like Kinase |
| NARS2 | Asparaginyl-TRNA Synthetase 2, Mitochondrial |
| GALR3 | Galanin Receptor 3 |
| RYR3 | Ryanodine Receptor 3 |
| GMPPA | GDP-Mannose Pyrophosphorylase A |
| HAS3 | Hyaluronan Synthase 3 |
| PPP1R2 | Protein Phosphatase 1 Regulatory Inhibitor Subunit 2 |
| TACC1 | Transforming Acidic Coiled-Coil Containing Protein 1 |
| OBSCN | Obscurin, Cytoskeletal Calmodulin And Titin-Interacting RhoGEF |
| ADH6 | Alcohol Dehydrogenase 6 (Class V) |
| FDX1 | Ferredoxin 1 |
| ELP4 | Elongator Acetyltransferase Complex Subunit 4 |
| ZFYVE16 | Zinc Finger FYVE-Type Containing 16 |
| UHMK1 | U2AF Homology Motif Kinase 1 |
| RANBP3 | RAN Binding Protein 3 |
| RAPGEF5 | Rap Guanine Nucleotide Exchange Factor 5 |
| ARMC9 | Armadillo Repeat Containing 9 |
| PLEKHA8 | Pleckstrin Homology Domain Containing A8 |
| WDR44 | WD Repeat Domain 44 |
| PI15 | Peptidase Inhibitor 15 |
| HEMGN | Hemogen |
| MIIP | Migration And Invasion Inhibitory Protein |
| SYAP1 | Synapse Associated Protein 1 |
| FAM114A2 | Family With Sequence Similarity 114 Member A2 |
| CALML4 | Calmodulin Like 4 |
| ZFC3H1 | Zinc Finger C3H1-Type Containing |
| SGO1 | Shugoshin 1 |
| ZNHIT6 | Zinc Finger HIT-Type Containing 6 |
| RAB29 | RAB29, Member RAS Oncogene Family |
| DBF4B | DBF4 Zinc Finger B |
| FRMD4B | FERM Domain Containing 4B |
| SS18L2 | SS18 Like 2 |
| PRADC1 | Protease Associated Domain Containing 1 |
| SPATC1L | Spermatogenesis And Centriole Associated 1 Like |
| PRM1 | Protamine 1 |
| PPP1R37 | Protein Phosphatase 1 Regulatory Subunit 37 |
| LRRC42 | Leucine Rich Repeat Containing 42 |
| ANKRD42 | Ankyrin Repeat Domain 42 |
| TENT2 | Terminal Nucleotidyltransferase 2 |
| DCAF4L2 | DDB1 And CUL4 Associated Factor 4 Like 2 |
| JPT1 | Jupiter Microtubule Associated Homolog 1 |
| CRAMP1 | Cramped Chromatin Regulator Homolog 1 |
| TCEAL9 | Transcription Elongation Factor A Like 9 |
| REXO5 | RNA Exonuclease 5 |
| RTL8C | Retrotransposon Gag Like 8C |
| ABHD14A-ACY1 | ABHD14A-ACY1 Readthrough |
| MIR570 | MicroRNA 570 |
| DUX4L1 | Double Homeobox 4 Like 1 (Pseudogene) |
| MROH3P | Maestro Heat Like Repeat Family Member 3, Pseudogene |
| NKILA | NF-KappaB Interacting LncRNA |
| MIR4422HG | MIR4422 Host Gene |
| MEF2C-AS2 | MEF2C Antisense RNA 2 |
| LINC01934 | Long Intergenic Non-Protein Coding RNA 1934 |
| CD44-AS1 | CD44 Antisense RNA 1 |
| SPON1-AS1 | SPON1 Antisense RNA 1 |
| FLT1P1 | FLT1 Pseudogene 1 |
| LINC02238 | Long Intergenic Non-Protein Coding RNA 2238 |
| LILRB1-AS1 | LILRB1 Antisense RNA 1 |
| SLC25A38P1 | Solute Carrier Family 25 Member 38 Pseudogene 1 |
| UQCRC2P1 | Ubiquinol-Cytochrome C Reductase Core Protein 2 Pseudogene 1 |
| BTF3L4P3 | Basic Transcription Factor 3 Like 4 Pseudogene 3 |
| LOC101927840 | Uncharacterized LOC101927840 |
| AFD1 | Acrofacial Dysostosis 1, Nager Type |
| LOC111674463 | CFTR Promoter Region |
| LOC111674476 | CFTR -35 Kb Upstream Enhancer |
| LOC111674478 | CFTR -44 Kb Upstream Enhancer |
| EGR3 | Early Growth Response 3 |
| AVPR2 | Arginine Vasopressin Receptor 2 |
| PTGIS | Prostaglandin I2 Synthase |
| TXNRD1 | Thioredoxin Reductase 1 |
| GRM3 | Glutamate Metabotropic Receptor 3 |
| RPL31 | Ribosomal Protein L31 |
| RPS11 | Ribosomal Protein S11 |
| DRG1 | Developmentally Regulated GTP Binding Protein 1 |
| ESM1 | Endothelial Cell Specific Molecule 1 |
| RNASE9 | Ribonuclease A Family Member 9 (Inactive) |
| RS1 | Retinoschisin 1 |
| PLA2G4E | Phospholipase A2 Group IVE |
| PLA2G4F | Phospholipase A2 Group IVF |
| SHC4 | SHC Adaptor Protein 4 |
| PAK5 | P21 (RAC1) Activated Kinase 5 |
| MYL10 | Myosin Light Chain 10 |
| JMJD7-PLA2G4B | JMJD7-PLA2G4B Readthrough |
| CLDN24 | Claudin 24 |
| MIR3917 | MicroRNA 3917 |
| EPHB1 | EPH Receptor B1 |
| CSNK1G2 | Casein Kinase 1 Gamma 2 |
| EPHB3 | EPH Receptor B3 |
| EPHA8 | EPH Receptor A8 |
| GNA14 | G Protein Subunit Alpha 14 |
| GPC6 | Glypican 6 |
| FGF12 | Fibroblast Growth Factor 12 |
| GPR119 | G Protein-Coupled Receptor 119 |
| FGF11 | Fibroblast Growth Factor 11 |
| BMP8B | Bone Morphogenetic Protein 8b |
| LMTK3 | Lemur Tyrosine Kinase 3 |
| PPBPP2 | Pro-Platelet Basic Protein Pseudogene 2 |
| SGK1 | Serum/Glucocorticoid Regulated Kinase 1 |
| CHRM2 | Cholinergic Receptor Muscarinic 2 |
| CHRNA5 | Cholinergic Receptor Nicotinic Alpha 5 Subunit |
| CYP4F3 | Cytochrome P450 Family 4 Subfamily F Member 3 |
| SCGB2A2 | Secretoglobin Family 2A Member 2 |
| RHOBTB2 | Rho Related BTB Domain Containing 2 |
| RPS4X | Ribosomal Protein S4 X-Linked |
| TAF5 | TATA-Box Binding Protein Associated Factor 5 |
| GPSM1 | G Protein Signaling Modulator 1 |
| NECTIN3 | Nectin Cell Adhesion Molecule 3 |
| CD200R1L | CD200 Receptor 1 Like |
| MIRLET7F1 | MicroRNA Let-7f-1 |
| FUT9 | Fucosyltransferase 9 |
| DDT | D-Dopachrome Tautomerase |
| TLE3 | TLE Family Member 3, Transcriptional Corepressor |
| RPS9 | Ribosomal Protein S9 |
| RPL9 | Ribosomal Protein L9 |
| RUNX1T1 | RUNX1 Partner Transcriptional Co-Repressor 1 |
| RPL6 | Ribosomal Protein L6 |
| RPS8 | Ribosomal Protein S8 |
| FYCO1 | FYVE And Coiled-Coil Domain Autophagy Adaptor 1 |
| MAGED2 | MAGE Family Member D2 |
| OTUB2 | OTU Deubiquitinase, Ubiquitin Aldehyde Binding 2 |
| LY6H | Lymphocyte Antigen 6 Family Member H |
| COL20A1 | Collagen Type XX Alpha 1 Chain |
| DNAJB4 | DnaJ Heat Shock Protein Family (Hsp40) Member B4 |
| PCGF6 | Polycomb Group Ring Finger 6 |
| PXDNL | Peroxidasin Like |
| FCGBP | Fc Fragment Of IgG Binding Protein |
| MPHOSPH9 | M-Phase Phosphoprotein 9 |
| H1-4 | H1.4 Linker Histone, Cluster Member |
| TRMT61A | TRNA Methyltransferase 61A |
| SLC35G2 | Solute Carrier Family 35 Member G2 |
| STIMATE | STIM Activating Enhancer |
| STIMATE-MUSTN1 | STIMATE-MUSTN1 Readthrough |
| LINC02551 | Long Intergenic Non-Protein Coding RNA 2551 |
| DRD5 | Dopamine Receptor D5 |
| SNW1 | SNW Domain Containing 1 |
| MAN2A2 | Mannosidase Alpha Class 2A Member 2 |
| ACRV1 | Acrosomal Vesicle Protein 1 |
| AMPD2 | Adenosine Monophosphate Deaminase 2 |
| KCNA1 | Potassium Voltage-Gated Channel Subfamily A Member 1 |
| CACNB4 | Calcium Voltage-Gated Channel Auxiliary Subunit Beta 4 |
| PDK4 | Pyruvate Dehydrogenase Kinase 4 |
| SMAD9 | SMAD Family Member 9 |
| MMP15 | Matrix Metallopeptidase 15 |
| DYRK1B | Dual Specificity Tyrosine Phosphorylation Regulated Kinase 1B |
| SCN3B | Sodium Voltage-Gated Channel Beta Subunit 3 |
| KCNE3 | Potassium Voltage-Gated Channel Subfamily E Regulatory Subunit 3 |
| CHRNB2 | Cholinergic Receptor Nicotinic Beta 2 Subunit |
| FZD9 | Frizzled Class Receptor 9 |
| CACNA1I | Calcium Voltage-Gated Channel Subunit Alpha1 I |
| ALS2 | Alsin Rho Guanine Nucleotide Exchange Factor ALS2 |
| TNIK | TRAF2 And NCK Interacting Kinase |
| NME4 | NME/NM23 Nucleoside Diphosphate Kinase 4 |
| RAMP1 | Receptor Activity Modifying Protein 1 |
| SLC33A1 | Solute Carrier Family 33 Member 1 |
| TLE4 | TLE Family Member 4, Transcriptional Corepressor |
| STK17B | Serine/Threonine Kinase 17b |
| HOXB1 | Homeobox B1 |
| RXFP2 | Relaxin Family Peptide Receptor 2 |
| CBX2 | Chromobox 2 |
| EZH1 | Enhancer Of Zeste 1 Polycomb Repressive Complex 2 Subunit |
| ARHGEF18 | Rho/Rac Guanine Nucleotide Exchange Factor 18 |
| ARSG | Arylsulfatase G |
| GDA | Guanine Deaminase |
| KERA | Keratocan |
| SIRT7 | Sirtuin 7 |
| INPP1 | Inositol Polyphosphate-1-Phosphatase |
| CPT1C | Carnitine Palmitoyltransferase 1C |
| NUDT1 | Nudix Hydrolase 1 |
| LRIG1 | Leucine Rich Repeats And Immunoglobulin Like Domains 1 |
| ANO3 | Anoctamin 3 |
| ASH1L | ASH1 Like Histone Lysine Methyltransferase |
| EPHA6 | EPH Receptor A6 |
| PIGV | Phosphatidylinositol Glycan Anchor Biosynthesis Class V |
| SP4 | Sp4 Transcription Factor |
| KLF15 | Kruppel Like Factor 15 |
| CHRD | Chordin |
| DOCK3 | Dedicator Of Cytokinesis 3 |
| TM7SF2 | Transmembrane 7 Superfamily Member 2 |
| WBP2 | WW Domain Binding Protein 2 |
| NPFFR2 | Neuropeptide FF Receptor 2 |
| KDELR1 | KDEL Endoplasmic Reticulum Protein Retention Receptor 1 |
| RABGGTA | Rab Geranylgeranyltransferase Subunit Alpha |
| SLC26A1 | Solute Carrier Family 26 Member 1 |
| ST8SIA1 | ST8 Alpha-N-Acetyl-Neuraminide Alpha-2,8-Sialyltransferase 1 |
| E2F6 | E2F Transcription Factor 6 |
| HOXB4 | Homeobox B4 |
| HOXB7 | Homeobox B7 |
| ONECUT1 | One Cut Homeobox 1 |
| CHST15 | Carbohydrate Sulfotransferase 15 |
| CDS2 | CDP-Diacylglycerol Synthase 2 |
| GALR2 | Galanin Receptor 2 |
| MFSD2A | Major Facilitator Superfamily Domain Containing 2A |
| AGTPBP1 | ATP/GTP Binding Protein 1 |
| ABCD2 | ATP Binding Cassette Subfamily D Member 2 |
| ASIC4 | Acid Sensing Ion Channel Subunit Family Member 4 |
| B3GNT5 | UDP-GlcNAc:BetaGal Beta-1,3-N-Acetylglucosaminyltransferase 5 |
| CAPG | Capping Actin Protein, Gelsolin Like |
| UXS1 | UDP-Glucuronate Decarboxylase 1 |
| UPP1 | Uridine Phosphorylase 1 |
| LIMD1 | LIM Domains Containing 1 |
| UPF3B | UPF3B Regulator Of Nonsense Mediated MRNA Decay |
| PHC1 | Polyhomeotic Homolog 1 |
| KCNK13 | Potassium Two Pore Domain Channel Subfamily K Member 13 |
| SNRK | SNF Related Kinase |
| SOSTDC1 | Sclerostin Domain Containing 1 |
| SH2D3C | SH2 Domain Containing 3C |
| PARVB | Parvin Beta |
| SIN3B | SIN3 Transcription Regulator Family Member B |
| INPP4B | Inositol Polyphosphate-4-Phosphatase Type II B |
| STARD13 | StAR Related Lipid Transfer Domain Containing 13 |
| MORC2 | MORC Family CW-Type Zinc Finger 2 |
| RIF1 | Replication Timing Regulatory Factor 1 |
| NEGR1 | Neuronal Growth Regulator 1 |
| NDST2 | N-Deacetylase And N-Sulfotransferase 2 |
| SEMA5B | Semaphorin 5B |
| SEZ6L | Seizure Related 6 Homolog Like |
| GPRC5B | G Protein-Coupled Receptor Class C Group 5 Member B |
| CHCHD10 | Coiled-Coil-Helix-Coiled-Coil-Helix Domain Containing 10 |
| MYO3B | Myosin IIIB |
| RNF10 | Ring Finger Protein 10 |
| RGS12 | Regulator Of G Protein Signaling 12 |
| ODF2 | Outer Dense Fiber Of Sperm Tails 2 |
| MAST4 | Microtubule Associated Serine/Threonine Kinase Family Member 4 |
| ADAMTS6 | ADAM Metallopeptidase With Thrombospondin Type 1 Motif 6 |
| ETNK1 | Ethanolamine Kinase 1 |
| ATP2C2 | ATPase Secretory Pathway Ca2+ Transporting 2 |
| BAZ2B | Bromodomain Adjacent To Zinc Finger Domain 2B |
| LPAR4 | Lysophosphatidic Acid Receptor 4 |
| BMF | Bcl2 Modifying Factor |
| ASF1B | Anti-Silencing Function 1B Histone Chaperone |
| ZFP36 | ZFP36 Ring Finger Protein |
| TUFT1 | Tuftelin 1 |
| WAC | WW Domain Containing Adaptor With Coiled-Coil |
| PHC2 | Polyhomeotic Homolog 2 |
| KCNH8 | Potassium Voltage-Gated Channel Subfamily H Member 8 |
| KCTD15 | Potassium Channel Tetramerization Domain Containing 15 |
| SPEN | Spen Family Transcriptional Repressor |
| PKN3 | Protein Kinase N3 |
| HSD17B14 | Hydroxysteroid 17-Beta Dehydrogenase 14 |
| POLE4 | DNA Polymerase Epsilon 4, Accessory Subunit |
| TDRD7 | Tudor Domain Containing 7 |
| ITM2C | Integral Membrane Protein 2C |
| OXGR1 | Oxoglutarate Receptor 1 |
| STAM2 | Signal Transducing Adaptor Molecule 2 |
| DCUN1D1 | Defective In Cullin Neddylation 1 Domain Containing 1 |
| OSBPL3 | Oxysterol Binding Protein Like 3 |
| SEMA6C | Semaphorin 6C |
| CHST2 | Carbohydrate Sulfotransferase 2 |
| GPM6B | Glycoprotein M6B |
| BCAS1 | Breast Carcinoma Amplified Sequence 1 |
| DPH2 | Diphthamide Biosynthesis 2 |
| DNAJC15 | DnaJ Heat Shock Protein Family (Hsp40) Member C15 |
| RRAGA | Ras Related GTP Binding A |
| GJC3 | Gap Junction Protein Gamma 3 |
| CELF4 | CUGBP Elav-Like Family Member 4 |
| MARCKSL1 | MARCKS Like 1 |
| LPIN3 | Lipin 3 |
| ESPN | Espin |
| CADPS2 | Calcium Dependent Secretion Activator 2 |
| AP4E1 | Adaptor Related Protein Complex 4 Subunit Epsilon 1 |
| ERN2 | Endoplasmic Reticulum To Nucleus Signaling 2 |
| TWSG1 | Twisted Gastrulation BMP Signaling Modulator 1 |
| ZC3HC1 | Zinc Finger C3HC-Type Containing 1 |
| ZDHHC13 | Zinc Finger DHHC-Type Palmitoyltransferase 13 |
| TRPM5 | Transient Receptor Potential Cation Channel Subfamily M Member 5 |
| L3MBTL1 | L3MBTL Histone Methyl-Lysine Binding Protein 1 |
| PGAP3 | Post-GPI Attachment To Proteins Phospholipase 3 |
| PFDN1 | Prefoldin Subunit 1 |
| SLCO1A2 | Solute Carrier Organic Anion Transporter Family Member 1A2 |
| SLC25A37 | Solute Carrier Family 25 Member 37 |
| SLC25A46 | Solute Carrier Family 25 Member 46 |
| ZNF644 | Zinc Finger Protein 644 |
| ZNF521 | Zinc Finger Protein 521 |
| SPO11 | SPO11 Initiator Of Meiotic Double Stranded Breaks |
| KPTN | Kaptin, Actin Binding Protein |
| CIZ1 | CDKN1A Interacting Zinc Finger Protein 1 |
| CTBS | Chitobiase |
| NHS | NHS Actin Remodeling Regulator |
| NDOR1 | NADPH Dependent Diflavin Oxidoreductase 1 |
| COX7A1 | Cytochrome C Oxidase Subunit 7A1 |
| CHST1 | Carbohydrate Sulfotransferase 1 |
| NMRK1 | Nicotinamide Riboside Kinase 1 |
| GK5 | Glycerol Kinase 5 |
| MFSD8 | Major Facilitator Superfamily Domain Containing 8 |
| GABPB1 | GA Binding Protein Transcription Factor Subunit Beta 1 |
| MEX3B | Mex-3 RNA Binding Family Member B |
| CCNB1IP1 | Cyclin B1 Interacting Protein 1 |
| ADAMTS16 | ADAM Metallopeptidase With Thrombospondin Type 1 Motif 16 |
| B3GNT7 | UDP-GlcNAc:BetaGal Beta-1,3-N-Acetylglucosaminyltransferase 7 |
| LYPD3 | LY6/PLAUR Domain Containing 3 |
| ARL6IP5 | ADP Ribosylation Factor Like GTPase 6 Interacting Protein 5 |
| ACOT11 | Acyl-CoA Thioesterase 11 |
| LIN9 | Lin-9 DREAM MuvB Core Complex Component |
| EN1 | Engrailed Homeobox 1 |
| THOC5 | THO Complex 5 |
| PIK3IP1 | Phosphoinositide-3-Kinase Interacting Protein 1 |
| HSPBAP1 | HSPB1 Associated Protein 1 |
| PPM1J | Protein Phosphatase, Mg2+/Mn2+ Dependent 1J |
| PPM1M | Protein Phosphatase, Mg2+/Mn2+ Dependent 1M |
| SYTL1 | Synaptotagmin Like 1 |
| ING2 | Inhibitor Of Growth Family Member 2 |
| KIAA1324 | KIAA1324 |
| STARD7 | StAR Related Lipid Transfer Domain Containing 7 |
| STK32A | Serine/Threonine Kinase 32A |
| EIF4EBP3 | Eukaryotic Translation Initiation Factor 4E Binding Protein 3 |
| NCAPH2 | Non-SMC Condensin II Complex Subunit H2 |
| HOXB3 | Homeobox B3 |
| HOXB8 | Homeobox B8 |
| SEMA3G | Semaphorin 3G |
| RTBDN | Retbindin |
| CEP76 | Centrosomal Protein 76 |
| NINJ2 | Ninjurin 2 |
| NUDT7 | Nudix Hydrolase 7 |
| SAMD8 | Sterile Alpha Motif Domain Containing 8 |
| RSAD1 | Radical S-Adenosyl Methionine Domain Containing 1 |
| GDPD5 | Glycerophosphodiester Phosphodiesterase Domain Containing 5 |
| MESP1 | Mesoderm Posterior BHLH Transcription Factor 1 |
| BIN3 | Bridging Integrator 3 |
| ARHGAP22 | Rho GTPase Activating Protein 22 |
| B3GNT6 | UDP-GlcNAc:BetaGal Beta-1,3-N-Acetylglucosaminyltransferase 6 |
| C12orf4 | Chromosome 12 Open Reading Frame 4 |
| C1QTNF7 | C1q And TNF Related 7 |
| ATL2 | Atlastin GTPase 2 |
| ATMIN | ATM Interactor |
| LYPLAL1 | Lysophospholipase Like 1 |
| AMER1 | APC Membrane Recruitment Protein 1 |
| AMZ2 | Archaelysin Family Metallopeptidase 2 |
| APLF | Aprataxin And PNKP Like Factor |
| ARAP2 | ArfGAP With RhoGAP Domain, Ankyrin Repeat And PH Domain 2 |
| A1CF | APOBEC1 Complementation Factor |
| A4GNT | Alpha-1,4-N-Acetylglucosaminyltransferase |
| EPYC | Epiphycan |
| TPCN1 | Two Pore Segment Channel 1 |
| UPK1B | Uroplakin 1B |
| USP12 | Ubiquitin Specific Peptidase 12 |
| WDR41 | WD Repeat Domain 41 |
| USP42 | Ubiquitin Specific Peptidase 42 |
| VPS54 | VPS54 Subunit Of GARP Complex |
| SLU7 | SLU7 Homolog, Splicing Factor |
| PHLDA1 | Pleckstrin Homology Like Domain Family A Member 1 |
| SGMS2 | Sphingomyelin Synthase 2 |
| SGSM1 | Small G Protein Signaling Modulator 1 |
| GPR21 | G Protein-Coupled Receptor 21 |
| SPIC | Spi-C Transcription Factor |
| PLEKHA5 | Pleckstrin Homology Domain Containing A5 |
| SNAI3 | Snail Family Transcriptional Repressor 3 |
| TAS2R1 | Taste 2 Receptor Member 1 |
| TASP1 | Taspase 1 |
| SIDT2 | SID1 Transmembrane Family Member 2 |
| QRICH1 | Glutamine Rich 1 |
| SSPN | Sarcospan |
| GSTCD | Glutathione S-Transferase C-Terminal Domain Containing |
| GSX1 | GS Homeobox 1 |
| RFTN1 | Raftlin, Lipid Raft Linker 1 |
| HNRNPLL | Heterogeneous Nuclear Ribonucleoprotein L Like |
| ELMOD1 | ELMO Domain Containing 1 |
| NDST3 | N-Deacetylase And N-Sulfotransferase 3 |
| MZF1 | Myeloid Zinc Finger 1 |
| RNF169 | Ring Finger Protein 169 |
| DCLRE1A | DNA Cross-Link Repair 1A |
| DHX35 | DEAH-Box Helicase 35 |
| GLT8D2 | Glycosyltransferase 8 Domain Containing 2 |
| MXRA8 | Matrix Remodeling Associated 8 |
| NKX3-2 | NK3 Homeobox 2 |
| NXNL1 | Nucleoredoxin Like 1 |
| GJD4 | Gap Junction Protein Delta 4 |
| CECR2 | CECR2 Histone Acetyl-Lysine Reader |
| MFSD10 | Major Facilitator Superfamily Domain Containing 10 |
| GLB1L2 | Galactosidase Beta 1 Like 2 |
| GCNT4 | Glucosaminyl (N-Acetyl) Transferase 4 |
| MED18 | Mediator Complex Subunit 18 |
| ATP11C | ATPase Phospholipid Transporting 11C |
| B4GALNT3 | Beta-1,4-N-Acetyl-Galactosaminyltransferase 3 |
| FITM2 | Fat Storage Inducing Transmembrane Protein 2 |
| FKBP11 | FKBP Prolyl Isomerase 11 |
| AQP11 | Aquaporin 11 |
| ARHGAP25 | Rho GTPase Activating Protein 25 |
| AVIL | Advillin |
| CCDC92 | Coiled-Coil Domain Containing 92 |
| TNS2 | Tensin 2 |
| VEZF1 | Vascular Endothelial Zinc Finger 1 |
| ZNF142 | Zinc Finger Protein 142 |
| YTHDC2 | YTH Domain Containing 2 |
| SLC38A10 | Solute Carrier Family 38 Member 10 |
| SLC46A3 | Solute Carrier Family 46 Member 3 |
| ZRANB1 | Zinc Finger RANBP2-Type Containing 1 |
| NABP2 | Nucleic Acid Binding Protein 2 |
| KDM8 | Lysine Demethylase 8 |
| THEMIS2 | Thymocyte Selection Associated Family Member 2 |
| PLD5 | Phospholipase D Family Member 5 |
| PLEKHF1 | Pleckstrin Homology And FYVE Domain Containing 1 |
| SNX8 | Sorting Nexin 8 |
| HSF2BP | Heat Shock Transcription Factor 2 Binding Protein |
| SOX12 | SRY-Box Transcription Factor 12 |
| RAB43 | RAB43, Member RAS Oncogene Family |
| TMUB1 | Transmembrane And Ubiquitin Like Domain Containing 1 |
| DBR1 | Debranching RNA Lariats 1 |
| NBEAL1 | Neurobeachin Like 1 |
| HDDC3 | HD Domain Containing 3 |
| CSRNP2 | Cysteine And Serine Rich Nuclear Protein 2 |
| CHCHD1 | Coiled-Coil-Helix-Coiled-Coil-Helix Domain Containing 1 |
| GPR155 | G Protein-Coupled Receptor 155 |
| C1orf127 | Chromosome 1 Open Reading Frame 127 |
| DOHH | Deoxyhypusine Hydroxylase |
| DPH6 | Diphthamine Biosynthesis 6 |
| RPS19BP1 | Ribosomal Protein S19 Binding Protein 1 |
| ETV2 | ETS Variant Transcription Factor 2 |
| FAM110B | Family With Sequence Similarity 110 Member B |
| FIGN | Fidgetin, Microtubule Severing Factor |
| C1orf43 | Chromosome 1 Open Reading Frame 43 |
| APOO | Apolipoprotein O |
| TMEM100 | Transmembrane Protein 100 |
| ZNF106 | Zinc Finger Protein 106 |
| YIPF6 | Yip1 Domain Family Member 6 |
| PDIK1L | PDLIM1 Interacting Kinase 1 Like |
| SLITRK4 | SLIT And NTRK Like Family Member 4 |
| ZNF420 | Zinc Finger Protein 420 |
| COL26A1 | Collagen Type XXVI Alpha 1 Chain |
| PRIMPOL | Primase And DNA Directed Polymerase |
| TMEM204 | Transmembrane Protein 204 |
| TMEM248 | Transmembrane Protein 248 |
| PRSS53 | Serine Protease 53 |
| CLEC4F | C-Type Lectin Domain Family 4 Member F |
| CYB561D2 | Cytochrome B561 Family Member D2 |
| RNFT2 | Ring Finger Protein, Transmembrane 2 |
| SCAI | Suppressor Of Cancer Cell Invasion |
| MICALCL | MICAL C-Terminal Like |
| HMGXB3 | HMG-Box Containing 3 |
| MFSD2B | Major Facilitator Superfamily Domain Containing 2B |
| CCDC106 | Coiled-Coil Domain Containing 106 |
| FRYL | FRY Like Transcription Coactivator |
| ATP13A5 | ATPase 13A5 |
| FAM83G | Family With Sequence Similarity 83 Member G |
| LDLRAD4 | Low Density Lipoprotein Receptor Class A Domain Containing 4 |
| SMG9 | SMG9 Nonsense Mediated MRNA Decay Factor |
| EFCAB3 | EF-Hand Calcium Binding Domain 3 |
| RAB19 | RAB19, Member RAS Oncogene Family |
| PXYLP1 | 2-Phosphoxylose Phosphatase 1 |
| SYCN | Syncollin |
| INCA1 | Inhibitor Of CDK, Cyclin A1 Interacting Protein 1 |
| KIAA1324L | KIAA1324 Like |
| TMEM178A | Transmembrane Protein 178A |
| PRSS50 | Serine Protease 50 |
| RHBDD3 | Rhomboid Domain Containing 3 |
| RIC8B | RIC8 Guanine Nucleotide Exchange Factor B |
| RNF186 | Ring Finger Protein 186 |
| HEG1 | Heart Development Protein With EGF Like Domains 1 |
| GPR146 | G Protein-Coupled Receptor 146 |
| GPATCH1 | G-Patch Domain Containing 1 |
| OAF | Out At First Homolog |
| METTL4 | Methyltransferase Like 4 |
| FOXO6 | Forkhead Box O6 |
| CCDC57 | Coiled-Coil Domain Containing 57 |
| ABHD17A | Abhydrolase Domain Containing 17A |
| FBXO36 | F-Box Protein 36 |
| C9orf116 | Chromosome 9 Open Reading Frame 116 |
| CAMSAP3 | Calmodulin Regulated Spectrin Associated Protein Family Member 3 |
| TMEM102 | Transmembrane Protein 102 |
| LGALS9B | Galectin 9B |
| WDR89 | WD Repeat Domain 89 |
| VSIG10 | V-Set And Immunoglobulin Domain Containing 10 |
| LARGE1 | LARGE Xylosyl- And Glucuronyltransferase 1 |
| ZSCAN10 | Zinc Finger And SCAN Domain Containing 10 |
| TAS2R39 | Taste 2 Receptor Member 39 |
| TMEM211 | Transmembrane Protein 211 |
| TMEM74 | Transmembrane Protein 74 |
| NFKBID | NFKB Inhibitor Delta |
| CSRNP1 | Cysteine And Serine Rich Nuclear Protein 1 |
| EMC10 | ER Membrane Protein Complex Subunit 10 |
| SAC3D1 | SAC3 Domain Containing 1 |
| BEX1 | Brain Expressed X-Linked 1 |
| FAM160A1 | Family With Sequence Similarity 160 Member A1 |
| BTBD16 | BTB Domain Containing 16 |
| C1orf198 | Chromosome 1 Open Reading Frame 198 |
| ASTE1 | Asteroid Homolog 1 |
| ZFP14 | ZFP14 Zinc Finger Protein |
| TMEM234 | Transmembrane Protein 234 |
| ZNF565 | Zinc Finger Protein 565 |
| LIX1L | Limb And CNS Expressed 1 Like |
| PANX3 | Pannexin 3 |
| KIAA1211L | KIAA1211 Like |
| C5orf51 | Chromosome 5 Open Reading Frame 51 |
| SERF1B | Small EDRK-Rich Factor 1B |
| GLMP | Glycosylated Lysosomal Membrane Protein |
| EXO5 | Exonuclease 5 |
| FAM210A | Family With Sequence Similarity 210 Member A |
| C1orf159 | Chromosome 1 Open Reading Frame 159 |
| C2CD4A | C2 Calcium Dependent Domain Containing 4A |
| LYG1 | Lysozyme G1 |
| KAT14 | Lysine Acetyltransferase 14 |
| KMT5C | Lysine Methyltransferase 5C |
| CCDC171 | Coiled-Coil Domain Containing 171 |
| AQP12B | Aquaporin 12B |
| ZNF488 | Zinc Finger Protein 488 |
| SQOR | Sulfide Quinone Oxidoreductase |
| MYBPHL | Myosin Binding Protein H Like |
| FAM209B | Family With Sequence Similarity 209 Member B |
| MEDAG | Mesenteric Estrogen Dependent Adipogenesis |
| XKRX | XK Related X-Linked |
| PRXL2A | Peroxiredoxin Like 2A |
| CIAO2A | Cytosolic Iron-Sulfur Assembly Component 2A |
| CFC1B | Cripto, FRL-1, Cryptic Family 1B |
| MINDY1 | MINDY Lysine 48 Deubiquitinase 1 |
| ABRAXAS2 | Abraxas 2, BRISC Complex Subunit |
| ACTL10 | Actin Like 10 |
| FAM234B | Family With Sequence Similarity 234 Member B |
| C4orf45 | Chromosome 4 Open Reading Frame 45 |
| LRRC30 | Leucine Rich Repeat Containing 30 |
| SKIDA1 | SKI/DACH Domain Containing 1 |
| TMEM35A | Transmembrane Protein 35A |
| COMMD3-BMI1 | COMMD3-BMI1 Readthrough |
| VSTM5 | V-Set And Transmembrane Domain Containing 5 |
| RCC1L | RCC1 Like |
| TERB1 | Telomere Repeat Binding Bouquet Formation Protein 1 |
| MINAR1 | Membrane Integral NOTCH2 Associated Receptor 1 |
| MGAT4D | MGAT4 Family Member D |
| RTL3 | Retrotransposon Gag Like 3 |
| FAM166C | Family With Sequence Similarity 166 Member C |
| C19orf67 | Chromosome 19 Open Reading Frame 67 |
| SPDYE18 | Speedy/RINGO Cell Cycle Regulator Family Member E18 |
| MTTP | Microsomal Triglyceride Transfer Protein |
| PABPN1 | Poly(A) Binding Protein Nuclear 1 |
| PTPRD | Protein Tyrosine Phosphatase Receptor Type D |
| SLC8A1 | Solute Carrier Family 8 Member A1 |
| LDHC | Lactate Dehydrogenase C |
| RPS23 | Ribosomal Protein S23 |
| TNFRSF19 | TNF Receptor Superfamily Member 19 |
| IK | IK Cytokine |
| CASP8AP2 | Caspase 8 Associated Protein 2 |
| DPYSL2 | Dihydropyrimidinase Like 2 |
| GCLM | Glutamate-Cysteine Ligase Modifier Subunit |
| BNIP3L | BCL2 Interacting Protein 3 Like |
| RAB9A | RAB9A, Member RAS Oncogene Family |
| RPS5 | Ribosomal Protein S5 |
| ASAP1 | ArfGAP With SH3 Domain, Ankyrin Repeat And PH Domain 1 |
| NRGN | Neurogranin |
| CLNS1A | Chloride Nucleotide-Sensitive Channel 1A |
| SF3B6 | Splicing Factor 3b Subunit 6 |
| TLE1 | TLE Family Member 1, Transcriptional Corepressor |
| EPS8 | Epidermal Growth Factor Receptor Pathway Substrate 8 |
| GRK5 | G Protein-Coupled Receptor Kinase 5 |
| PTP4A3 | Protein Tyrosine Phosphatase 4A3 |
| NFRKB | Nuclear Factor Related To KappaB Binding Protein |
| CD302 | CD302 Molecule |
| VARS1 | Valyl-TRNA Synthetase 1 |
| BCL2L15 | BCL2 Like 15 |
| STX1B | Syntaxin 1B |
| NUDT2 | Nudix Hydrolase 2 |
| ZNF282 | Zinc Finger Protein 282 |
| SNORD105 | Small Nucleolar RNA, C/D Box 105 |
| IGLV1-41 | Immunoglobulin Lambda Variable 1-41 (Pseudogene) |
| IGLV2-34 | Immunoglobulin Lambda Variable 2-34 (Pseudogene) |
| LOC111674464 | CFTR Intron 1 Enhancer |
| LOC111674466 | DNase I Hypersensitive Site -20.9 Kb Upstream Of CFTR |
| LOC111674470 | DNase I Hypersensitive Region 15.6 Kb Downstream Of CFTR |
| LOC111674473 | DNase I Hypersensitive Sites In Intron 10ab Of CFTR |
| LOC111674475 | CFTR Intron 11 Enhancer |
| LOC111674477 | CFTR Intron 23 Enhancer |
| LOC111674479 | CFTR +36.6 Kb Downstream Enhancer |
| LOC113633875 | CFTR Intron 18a DNase I Hypersensitive Site |
| LOC113633877 | CFTR Intron 19 DNase I Hypersensitive Site |
| LOC113523647 | CFTR +21.5 Kb DNase I Hypersensitive Site |
| STK39 | Serine/Threonine Kinase 39 |
| KDM3B | Lysine Demethylase 3B |
| TAF2 | TATA-Box Binding Protein Associated Factor 2 |
| STK16 | Serine/Threonine Kinase 16 |
| S100A14 | S100 Calcium Binding Protein A14 |
| PPP2R3A | Protein Phosphatase 2 Regulatory Subunit B''Alpha |
| USP49 | Ubiquitin Specific Peptidase 49 |
| ADAMTSL3 | ADAMTS Like 3 |
| LSM14A | LSM14A MRNA Processing Body Assembly Factor |
| EAPP | E2F Associated Phosphoprotein |
| IARS1 | Isoleucyl-TRNA Synthetase 1 |
| PNMA1 | PNMA Family Member 1 |
| TSNARE1 | T-SNARE Domain Containing 1 |
| ECSCR | Endothelial Cell Surface Expressed Chemotaxis And Apoptosis Regulator |
| MIP | Major Intrinsic Protein Of Lens Fiber |
| ADAM18 | ADAM Metallopeptidase Domain 18 |
| KAT2A | Lysine Acetyltransferase 2A |
| CAPN2 | Calpain 2 |
| PAFAH1B1 | Platelet Activating Factor Acetylhydrolase 1b Regulatory Subunit 1 |
| PIK3C2B | Phosphatidylinositol-4-Phosphate 3-Kinase Catalytic Subunit Type 2 Beta |
| ANK3 | Ankyrin 3 |
| RHOB | Ras Homolog Family Member B |
| USP2 | Ubiquitin Specific Peptidase 2 |
| PCBP1 | Poly(RC) Binding Protein 1 |
| LGR6 | Leucine Rich Repeat Containing G Protein-Coupled Receptor 6 |
| GOLGA1 | Golgin A1 |
| FMNL1 | Formin Like 1 |
| GOLGA4 | Golgin A4 |
| IMPACT | Impact RWD Domain Protein |
| SH2D3A | SH2 Domain Containing 3A |
| ZBTB10 | Zinc Finger And BTB Domain Containing 10 |
| MELTF | Melanotransferrin |
| CRADD | CASP2 And RIPK1 Domain Containing Adaptor With Death Domain |
| EFNA1 | Ephrin A1 |
| AGRP | Agouti Related Neuropeptide |
| RPL10A | Ribosomal Protein L10a |
| RPL4 | Ribosomal Protein L4 |
| CALB1 | Calbindin 1 |
| VRK2 | VRK Serine/Threonine Kinase 2 |
| MYBBP1A | MYB Binding Protein 1a |
| LSM4 | LSM4 Homolog, U6 Small Nuclear RNA And MRNA Degradation Associated |
| NEO1 | Neogenin 1 |
| ABCB9 | ATP Binding Cassette Subfamily B Member 9 |
| BPHL | Biphenyl Hydrolase Like |
| APOM | Apolipoprotein M |
| TOMM20 | Translocase Of Outer Mitochondrial Membrane 20 |
| SPAM1 | Sperm Adhesion Molecule 1 |
| NEU3 | Neuraminidase 3 |
| SFXN1 | Sideroflexin 1 |
| DNER | Delta/Notch Like EGF Repeat Containing |
| SLC4A3 | Solute Carrier Family 4 Member 3 |
| NKTR | Natural Killer Cell Triggering Receptor |
| MED6 | Mediator Complex Subunit 6 |
| HCAR3 | Hydroxycarboxylic Acid Receptor 3 |
| SFMBT1 | Scm Like With Four Mbt Domains 1 |
| TSPAN4 | Tetraspanin 4 |
| KIAA0100 | KIAA0100 |
| SRRM2 | Serine/Arginine Repetitive Matrix 2 |
| PLEKHO1 | Pleckstrin Homology Domain Containing O1 |
| LACRT | Lacritin |
| HSP90AA2P | Heat Shock Protein 90 Alpha Family Class A Member 2, Pseudogene |
| KRT4 | Keratin 4 |
| IGFBP4 | Insulin Like Growth Factor Binding Protein 4 |
| CRYZ | Crystallin Zeta |
| MYO1A | Myosin IA |
| PPY | Pancreatic Polypeptide |
| RPS21 | Ribosomal Protein S21 |
| SRSF4 | Serine And Arginine Rich Splicing Factor 4 |
| ITIH1 | Inter-Alpha-Trypsin Inhibitor Heavy Chain 1 |
| AHSA1 | Activator Of HSP90 ATPase Activity 1 |
| CYTH3 | Cytohesin 3 |
| GEMIN2 | Gem Nuclear Organelle Associated Protein 2 |
| AFM | Afamin |
| IGSF10 | Immunoglobulin Superfamily Member 10 |
| PYCR3 | Pyrroline-5-Carboxylate Reductase 3 |
| MICU3 | Mitochondrial Calcium Uptake Family Member 3 |
| HGH1 | HGH1 Homolog |
| DEFB130B | Defensin Beta 130B |
| MAP4K4 | Mitogen-Activated Protein Kinase Kinase Kinase Kinase 4 |
| CDK5R1 | Cyclin Dependent Kinase 5 Regulatory Subunit 1 |
| NT5C2 | 5'-Nucleotidase, Cytosolic II |
| TUBA4A | Tubulin Alpha 4a |
| TDG | Thymine DNA Glycosylase |
| QKI | QKI, KH Domain Containing RNA Binding |
| GSTO1 | Glutathione S-Transferase Omega 1 |
| APOD | Apolipoprotein D |
| RAB2A | RAB2A, Member RAS Oncogene Family |
| CSRP1 | Cysteine And Glycine Rich Protein 1 |
| RPL7A | Ribosomal Protein L7a |
| CCNK | Cyclin K |
| ARHGEF7 | Rho Guanine Nucleotide Exchange Factor 7 |
| TAOK2 | TAO Kinase 2 |
| GPLD1 | Glycosylphosphatidylinositol Specific Phospholipase D1 |
| HNRNPF | Heterogeneous Nuclear Ribonucleoprotein F |
| HNRNPM | Heterogeneous Nuclear Ribonucleoprotein M |
| RPL28 | Ribosomal Protein L28 |
| TMOD3 | Tropomodulin 3 |
| NFYA | Nuclear Transcription Factor Y Subunit Alpha |
| GATAD2A | GATA Zinc Finger Domain Containing 2A |
| ANXA13 | Annexin A13 |
| SMOX | Spermine Oxidase |
| RPS25 | Ribosomal Protein S25 |
| PIWIL4 | Piwi Like RNA-Mediated Gene Silencing 4 |
| STAB2 | Stabilin 2 |
| SNAPIN | SNAP Associated Protein |
| PPP1R3B | Protein Phosphatase 1 Regulatory Subunit 3B |
| MORC3 | MORC Family CW-Type Zinc Finger 3 |
| EFHD1 | EF-Hand Domain Family Member D1 |
| STK35 | Serine/Threonine Kinase 35 |
| CNTNAP4 | Contactin Associated Protein Family Member 4 |
| DOCK5 | Dedicator Of Cytokinesis 5 |
| MED30 | Mediator Complex Subunit 30 |
| MON1B | MON1 Homolog B, Secretory Trafficking Associated |
| CAB39L | Calcium Binding Protein 39 Like |
| AAMP | Angio Associated Migratory Cell Protein |
| APOL3 | Apolipoprotein L3 |
| RLN1 | Relaxin 1 |
| PRR16 | Proline Rich 16 |
| MMD2 | Monocyte To Macrophage Differentiation Associated 2 |
| PRR4 | Proline Rich 4 |
| GSDMC | Gasdermin C |
| RBM33 | RNA Binding Motif Protein 33 |
| PAGE1 | PAGE Family Member 1 |
| SELENOH | Selenoprotein H |
| TARP | TCR Gamma Alternate Reading Frame Protein |
| RANP1 | RAN Pseudogene 1 |
| DEFB108C | Defensin Beta 108C |
| GLUD1 | Glutamate Dehydrogenase 1 |
| ENO3 | Enolase 3 |
| MYO6 | Myosin VI |
| CHN1 | Chimerin 1 |
| ABAT | 4-Aminobutyrate Aminotransferase |
| GSTA3 | Glutathione S-Transferase Alpha 3 |
| HIPK2 | Homeodomain Interacting Protein Kinase 2 |
| UBA2 | Ubiquitin Like Modifier Activating Enzyme 2 |
| NEK1 | NIMA Related Kinase 1 |
| DLGAP1 | DLG Associated Protein 1 |
| SLC8A3 | Solute Carrier Family 8 Member A3 |
| RAP1GDS1 | Rap1 GTPase-GDP Dissociation Stimulator 1 |
| CNNM2 | Cyclin And CBS Domain Divalent Metal Cation Transport Mediator 2 |
| HAGH | Hydroxyacylglutathione Hydrolase |
| CHN2 | Chimerin 2 |
| NRXN3 | Neurexin 3 |
| SLC6A20 | Solute Carrier Family 6 Member 20 |
| INA | Internexin Neuronal Intermediate Filament Protein Alpha |
| RBM10 | RNA Binding Motif Protein 10 |
| EHF | ETS Homologous Factor |
| HAS2 | Hyaluronan Synthase 2 |
| DIO3 | Iodothyronine Deiodinase 3 |
| SENP2 | SUMO Specific Peptidase 2 |
| AMOT | Angiomotin |
| VPS26A | VPS26, Retromer Complex Component A |
| TNK1 | Tyrosine Kinase Non Receptor 1 |
| TGS1 | Trimethylguanosine Synthase 1 |
| PLCL1 | Phospholipase C Like 1 (Inactive) |
| PSD3 | Pleckstrin And Sec7 Domain Containing 3 |
| MRPL12 | Mitochondrial Ribosomal Protein L12 |
| NECTIN4 | Nectin Cell Adhesion Molecule 4 |
| FOXP4 | Forkhead Box P4 |
| RPL26L1 | Ribosomal Protein L26 Like 1 |
| RPL29 | Ribosomal Protein L29 |
| TRO | Trophinin |
| NOSIP | Nitric Oxide Synthase Interacting Protein |
| GTF2A1 | General Transcription Factor IIA Subunit 1 |
| SAMD12 | Sterile Alpha Motif Domain Containing 12 |
| NUTF2 | Nuclear Transport Factor 2 |
| SNAPC4 | Small Nuclear RNA Activating Complex Polypeptide 4 |
| STXBP6 | Syntaxin Binding Protein 6 |
| GPR162 | G Protein-Coupled Receptor 162 |
| RNF11 | Ring Finger Protein 11 |
| GSTA5 | Glutathione S-Transferase Alpha 5 |
| LRFN5 | Leucine Rich Repeat And Fibronectin Type III Domain Containing 5 |
| ZDHHC5 | Zinc Finger DHHC-Type Palmitoyltransferase 5 |
| C1QL1 | Complement C1q Like 1 |
| OR2H1 | Olfactory Receptor Family 2 Subfamily H Member 1 |
| MYO7B | Myosin VIIB |
| ACTR5 | Actin Related Protein 5 |
| CABYR | Calcium Binding Tyrosine Phosphorylation Regulated |
| MAMLD1 | Mastermind Like Domain Containing 1 |
| ANKRD44 | Ankyrin Repeat Domain 44 |
| THSD7A | Thrombospondin Type 1 Domain Containing 7A |
| ZNF827 | Zinc Finger Protein 827 |
| RAVER2 | Ribonucleoprotein, PTB Binding 2 |
| MS4A14 | Membrane Spanning 4-Domains A14 |
| MICALL1 | MICAL Like 1 |
| RNF26 | Ring Finger Protein 26 |
| ARL6IP4 | ADP Ribosylation Factor Like GTPase 6 Interacting Protein 4 |
| SWT1 | SWT1 RNA Endoribonuclease Homolog |
| XKR4 | XK Related 4 |
| FRMD1 | FERM Domain Containing 1 |
| AKIRIN1 | Akirin 1 |
| C1orf68 | Chromosome 1 Open Reading Frame 68 |
| IFI27L1 | Interferon Alpha Inducible Protein 27 Like 1 |
| POTEE | POTE Ankyrin Domain Family Member E |
| KIR3DX1 | Killer Cell Immunoglobulin Like Receptor, Three Ig Domains X1 (Pseudogene) |
| SOX2-OT | SOX2 Overlapping Transcript |
| MIR148B | MicroRNA 148b |
| LINC00271 | Long Intergenic Non-Protein Coding RNA 271 |
| MIR576 | MicroRNA 576 |
| SIGLEC17P | Sialic Acid Binding Ig Like Lectin 17, Pseudogene |
| DEFB109B | Defensin Beta 109B |
| MBL1P | Mannose Binding Lectin 1, Pseudogene |
| DEFT1P2 | Defensin Theta 1, Pseudogene 2 |
| LOC109623489 | Insulin Repeat Instability Region |
| POLG2 | DNA Polymerase Gamma 2, Accessory Subunit |
| SRI | Sorcin |
| TCEA1 | Transcription Elongation Factor A1 |
| CDCA5 | Cell Division Cycle Associated 5 |
| NAA20 | N-Alpha-Acetyltransferase 20, NatB Catalytic Subunit |
| SOX7 | SRY-Box Transcription Factor 7 |
| TNP1 | Transition Protein 1 |
| SPACA1 | Sperm Acrosome Associated 1 |
| AQP2 | Aquaporin 2 |
| ATP2A3 | ATPase Sarcoplasmic/Endoplasmic Reticulum Ca2+ Transporting 3 |
| TNNI2 | Troponin I2, Fast Skeletal Type |
| CRAT | Carnitine O-Acetyltransferase |
| GPR37 | G Protein-Coupled Receptor 37 |
| ALAS1 | 5'-Aminolevulinate Synthase 1 |
| MTA1 | Metastasis Associated 1 |
| RXFP1 | Relaxin Family Peptide Receptor 1 |
| CES2 | Carboxylesterase 2 |
| PTH2R | Parathyroid Hormone 2 Receptor |
| EMX2 | Empty Spiracles Homeobox 2 |
| SLC25A10 | Solute Carrier Family 25 Member 10 |
| MSRA | Methionine Sulfoxide Reductase A |
| RPL12 | Ribosomal Protein L12 |
| ARHGEF10 | Rho Guanine Nucleotide Exchange Factor 10 |
| SLC4A5 | Solute Carrier Family 4 Member 5 |
| GSTM5 | Glutathione S-Transferase Mu 5 |
| GPR1 | G Protein-Coupled Receptor 1 |
| GDF3 | Growth Differentiation Factor 3 |
| MADD | MAP Kinase Activating Death Domain |
| BRD7 | Bromodomain Containing 7 |
| HEPACAM | Hepatic And Glial Cell Adhesion Molecule |
| SRGAP3 | SLIT-ROBO Rho GTPase Activating Protein 3 |
| PRIM2 | DNA Primase Subunit 2 |
| PUS7 | Pseudouridine Synthase 7 |
| NEK4 | NIMA Related Kinase 4 |
| SFXN4 | Sideroflexin 4 |
| GPR37L1 | G Protein-Coupled Receptor 37 Like 1 |
| GPM6A | Glycoprotein M6A |
| CHDH | Choline Dehydrogenase |
| BCAR3 | BCAR3 Adaptor Protein, NSP Family Member |
| NDUFB6 | NADH:Ubiquinone Oxidoreductase Subunit B6 |
| GALNT10 | Polypeptide N-Acetylgalactosaminyltransferase 10 |
| MACF1 | Microtubule Actin Crosslinking Factor 1 |
| AS3MT | Arsenite Methyltransferase |
| LIMS2 | LIM Zinc Finger Domain Containing 2 |
| SMG6 | SMG6 Nonsense Mediated MRNA Decay Factor |
| SLC4A10 | Solute Carrier Family 4 Member 10 |
| MYH7B | Myosin Heavy Chain 7B |
| RCOR1 | REST Corepressor 1 |
| TESK1 | Testis Associated Actin Remodelling Kinase 1 |
| RGL2 | Ral Guanine Nucleotide Dissociation Stimulator Like 2 |
| NUCB1 | Nucleobindin 1 |
| GDF7 | Growth Differentiation Factor 7 |
| BCL2L13 | BCL2 Like 13 |
| FKBP6 | FKBP Prolyl Isomerase 6 |
| TSNAX | Translin Associated Factor X |
| MTHFD2L | Methylenetetrahydrofolate Dehydrogenase (NADP+ Dependent) 2 Like |
| NOXA1 | NADPH Oxidase Activator 1 |
| RLN2 | Relaxin 2 |
| CENPK | Centromere Protein K |
| DOK5 | Docking Protein 5 |
| FAHD1 | Fumarylacetoacetate Hydrolase Domain Containing 1 |
| EYA3 | EYA Transcriptional Coactivator And Phosphatase 3 |
| FAM83H | Family With Sequence Similarity 83 Member H |
| TSPAN31 | Tetraspanin 31 |
| PSRC1 | Proline And Serine Rich Coiled-Coil 1 |
| SPARCL1 | SPARC Like 1 |
| TBC1D5 | TBC1 Domain Family Member 5 |
| NFE2L3 | Nuclear Factor, Erythroid 2 Like 3 |
| MRPS18A | Mitochondrial Ribosomal Protein S18A |
| ASCL2 | Achaete-Scute Family BHLH Transcription Factor 2 |
| WDR33 | WD Repeat Domain 33 |
| SLITRK2 | SLIT And NTRK Like Family Member 2 |
| CNTRL | Centriolin |
| SUN5 | Sad1 And UNC84 Domain Containing 5 |
| RASSF3 | Ras Association Domain Family Member 3 |
| TEX15 | Testis Expressed 15, Meiosis And Synapsis Associated |
| TMTC1 | Transmembrane O-Mannosyltransferase Targeting Cadherins 1 |
| TMX2 | Thioredoxin Related Transmembrane Protein 2 |
| STARD5 | StAR Related Lipid Transfer Domain Containing 5 |
| FNDC3B | Fibronectin Type III Domain Containing 3B |
| CNTN5 | Contactin 5 |
| MOB3B | MOB Kinase Activator 3B |
| CRIP2 | Cysteine Rich Protein 2 |
| DERL1 | Derlin 1 |
| MRPL22 | Mitochondrial Ribosomal Protein L22 |
| CENPT | Centromere Protein T |
| DUS4L | Dihydrouridine Synthase 4 Like |
| CDX1 | Caudal Type Homeobox 1 |
| BCAP29 | B Cell Receptor Associated Protein 29 |
| CAMKMT | Calmodulin-Lysine N-Methyltransferase |
| ARHGAP10 | Rho GTPase Activating Protein 10 |
| THEM5 | Thioesterase Superfamily Member 5 |
| VSIG2 | V-Set And Immunoglobulin Domain Containing 2 |
| SLITRK3 | SLIT And NTRK Like Family Member 3 |
| ZSWIM6 | Zinc Finger SWIM-Type Containing 6 |
| COL28A1 | Collagen Type XXVIII Alpha 1 Chain |
| PCYOX1L | Prenylcysteine Oxidase 1 Like |
| KIF12 | Kinesin Family Member 12 |
| CMTR1 | Cap Methyltransferase 1 |
| CHADL | Chondroadherin Like |
| CENPM | Centromere Protein M |
| RPAIN | RPA Interacting Protein |
| GFOD2 | Glucose-Fructose Oxidoreductase Domain Containing 2 |
| EPHX4 | Epoxide Hydrolase 4 |
| VEPH1 | Ventricular Zone Expressed PH Domain Containing 1 |
| LMAN1L | Lectin, Mannose Binding 1 Like |
| EFR3A | EFR3 Homolog A |
| NPFF | Neuropeptide FF-Amide Peptide Precursor |
| PUS7L | Pseudouridine Synthase 7 Like |
| PSG9 | Pregnancy Specific Beta-1-Glycoprotein 9 |
| RFTN2 | Raftlin Family Member 2 |
| ELAC1 | ElaC Ribonuclease Z 1 |
| COL6A5 | Collagen Type VI Alpha 5 Chain |
| MSL2 | MSL Complex Subunit 2 |
| CHERP | Calcium Homeostasis Endoplasmic Reticulum Protein |
| LRIT1 | Leucine Rich Repeat, Ig-Like And Transmembrane Domains 1 |
| ATAT1 | Alpha Tubulin Acetyltransferase 1 |
| USP35 | Ubiquitin Specific Peptidase 35 |
| WDR92 | WD Repeat Domain 92 |
| ZNF839 | Zinc Finger Protein 839 |
| C1QL2 | Complement C1q Like 2 |
| HAGHL | Hydroxyacylglutathione Hydrolase Like |
| RMDN3 | Regulator Of Microtubule Dynamics 3 |
| DNAH3 | Dynein Axonemal Heavy Chain 3 |
| KRT78 | Keratin 78 |
| TENM2 | Teneurin Transmembrane Protein 2 |
| PDCD7 | Programmed Cell Death 7 |
| RBM15B | RNA Binding Motif Protein 15B |
| LYSMD3 | LysM Domain Containing 3 |
| NECAB3 | N-Terminal EF-Hand Calcium Binding Protein 3 |
| DCHS2 | Dachsous Cadherin-Related 2 |
| NUDT22 | Nudix Hydrolase 22 |
| CCM2L | CCM2 Like Scaffold Protein |
| MEPCE | Methylphosphate Capping Enzyme |
| LRIT2 | Leucine Rich Repeat, Ig-Like And Transmembrane Domains 2 |
| TSNAXIP1 | Translin Associated Factor X Interacting Protein 1 |
| ZPLD1 | Zona Pellucida Like Domain Containing 1 |
| GSKIP | GSK3B Interacting Protein |
| CMTM1 | CKLF Like MARVEL Transmembrane Domain Containing 1 |
| MS4A5 | Membrane Spanning 4-Domains A5 |
| DNAJC4 | DnaJ Heat Shock Protein Family (Hsp40) Member C4 |
| RNF122 | Ring Finger Protein 122 |
| ENTHD1 | ENTH Domain Containing 1 |
| ZC3H15 | Zinc Finger CCCH-Type Containing 15 |
| SPATA2L | Spermatogenesis Associated 2 Like |
| IGSF9B | Immunoglobulin Superfamily Member 9B |
| SLC7A6OS | Solute Carrier Family 7 Member 6 Opposite Strand |
| HEATR9 | HEAT Repeat Containing 9 |
| HASPIN | Histone H3 Associated Protein Kinase |
| BTBD18 | BTB Domain Containing 18 |
| DRICH1 | Aspartate Rich 1 |
| DMAC2L | Distal Membrane Arm Assembly Complex 2 Like |
| CEMIP2 | Cell Migration Inducing Hyaluronidase 2 |
| C2orf83 | Chromosome 2 Open Reading Frame 83 |
| C11orf40 | Chromosome 11 Open Reading Frame 40 (Putative) |
| SPINDOC | Spindlin Interactor And Repressor Of Chromatin Binding |
| TTC6 | Tetratricopeptide Repeat Domain 6 |
| SNORC | Secondary Ossification Center Associated Regulator Of Chondrocyte Maturation |
| TMEM121B | Transmembrane Protein 121B |
| KIRREL3-AS3 | KIRREL3 Antisense RNA 3 |
| MIR590 | MicroRNA 590 |
| MIR137 | MicroRNA 137 |
| LINC00486 | Long Intergenic Non-Protein Coding RNA 486 |
| TMX2-CTNND1 | TMX2-CTNND1 Readthrough (NMD Candidate) |
| CYMP | Chymosin, Pseudogene |
| SMIM27 | Small Integral Membrane Protein 27 |
| ENO1P1 | Enolase 1 Pseudogene 1 |
| CYMP-AS1 | CYMP Antisense RNA 1 |
| TRAJ2 | T Cell Receptor Alpha Joining 2 (Non-Functional) |
| TRAJ1 | T Cell Receptor Alpha Joining 1 (Non-Functional) |
| TRAJ12 | T Cell Receptor Alpha Joining 12 |
| TRAJ22 | T Cell Receptor Alpha Joining 22 |
| TRAJ24 | T Cell Receptor Alpha Joining 24 |
| TRAJ25 | T Cell Receptor Alpha Joining 25 (Non-Functional) |
| TRAJ29 | T Cell Receptor Alpha Joining 29 |
| PCBP3-AS1 | PCBP3 Antisense RNA 1 |
| LOC729968 | Spore Coat Protein SP96 |
| ATP5MGP5 | ATP Synthase Membrane Subunit G Pseudogene 5 |
| ATP5MC2P2 | ATP Synthase Membrane Subunit C Locus 2 Pseudogene 2 |
| LOC441996 | Aconitase 2, Mitochondrial Pseudogene |
| LOC100129725 | PERP, TP53 Apoptosis Effector Pseudogene |
| MIR302A | MicroRNA 302a |
| LOC110283621 | SPP1 5' Regulatory Region |
| ADRA1D | Adrenoceptor Alpha 1D |
| SLC18A1 | Solute Carrier Family 18 Member A1 |
| OXTR | Oxytocin Receptor |
| HCN1 | Hyperpolarization Activated Cyclic Nucleotide Gated Potassium Channel 1 |
| TPK1 | Thiamin Pyrophosphokinase 1 |
| NDUFA6 | NADH:Ubiquinone Oxidoreductase Subunit A6 |
| PTDSS1 | Phosphatidylserine Synthase 1 |
| PRPH | Peripherin |
| FARS2 | Phenylalanyl-TRNA Synthetase 2, Mitochondrial |
| BCAT2 | Branched Chain Amino Acid Transaminase 2 |
| CA14 | Carbonic Anhydrase 14 |
| KDM5B | Lysine Demethylase 5B |
| TNNT3 | Troponin T3, Fast Skeletal Type |
| CKMT2 | Creatine Kinase, Mitochondrial 2 |
| SFRP2 | Secreted Frizzled Related Protein 2 |
| PARD6A | Par-6 Family Cell Polarity Regulator Alpha |
| STAG1 | Stromal Antigen 1 |
| STK38L | Serine/Threonine Kinase 38 Like |
| RHOQ | Ras Homolog Family Member Q |
| RSPO2 | R-Spondin 2 |
| APH1A | Aph-1 Homolog A, Gamma-Secretase Subunit |
| KCNMB1 | Potassium Calcium-Activated Channel Subfamily M Regulatory Beta Subunit 1 |
| REEP2 | Receptor Accessory Protein 2 |
| POLE2 | DNA Polymerase Epsilon 2, Accessory Subunit |
| SLC32A1 | Solute Carrier Family 32 Member 1 |
| FNTA | Farnesyltransferase, CAAX Box, Alpha |
| MLH3 | MutL Homolog 3 |
| HNF4G | Hepatocyte Nuclear Factor 4 Gamma |
| NCAN | Neurocan |
| MARS2 | Methionyl-TRNA Synthetase 2, Mitochondrial |
| KDM2A | Lysine Demethylase 2A |
| DGKI | Diacylglycerol Kinase Iota |
| RPL8 | Ribosomal Protein L8 |
| MAPRE2 | Microtubule Associated Protein RP/EB Family Member 2 |
| ADAM23 | ADAM Metallopeptidase Domain 23 |
| BRF1 | BRF1 RNA Polymerase III Transcription Initiation Factor Subunit |
| NLGN4X | Neuroligin 4 X-Linked |
| TDRD9 | Tudor Domain Containing 9 |
| ISYNA1 | Inositol-3-Phosphate Synthase 1 |
| PSKH1 | Protein Serine Kinase H1 |
| CNTN4 | Contactin 4 |
| E2F5 | E2F Transcription Factor 5 |
| MYT1L | Myelin Transcription Factor 1 Like |
| MSI1 | Musashi RNA Binding Protein 1 |
| USP20 | Ubiquitin Specific Peptidase 20 |
| TFCP2 | Transcription Factor CP2 |
| TARBP1 | TAR (HIV-1) RNA Binding Protein 1 |
| JPH1 | Junctophilin 1 |
| CTCFL | CCCTC-Binding Factor Like |
| NGEF | Neuronal Guanine Nucleotide Exchange Factor |
| HAP1 | Huntingtin Associated Protein 1 |
| SCGN | Secretagogin, EF-Hand Calcium Binding Protein |
| CEPT1 | Choline/Ethanolamine Phosphotransferase 1 |
| DPEP2 | Dipeptidase 2 |
| DOC2A | Double C2 Domain Alpha |
| CCNL1 | Cyclin L1 |
| ADAM28 | ADAM Metallopeptidase Domain 28 |
| AZIN1 | Antizyme Inhibitor 1 |
| AMBN | Ameloblastin |
| ANO10 | Anoctamin 10 |
| TRA2B | Transformer 2 Beta Homolog |
| SGPP1 | Sphingosine-1-Phosphate Phosphatase 1 |
| ZNF331 | Zinc Finger Protein 331 |
| PPP1R10 | Protein Phosphatase 1 Regulatory Subunit 10 |
| KIF20B | Kinesin Family Member 20B |
| STAC | SH3 And Cysteine Rich Domain |
| STRN3 | Striatin 3 |
| CLASP2 | Cytoplasmic Linker Associated Protein 2 |
| NGB | Neuroglobin |
| RNF170 | Ring Finger Protein 170 |
| SFXN2 | Sideroflexin 2 |
| SEC23IP | SEC23 Interacting Protein |
| GLT8D1 | Glycosyltransferase 8 Domain Containing 1 |
| RPL32 | Ribosomal Protein L32 |
| RTN1 | Reticulon 1 |
| GALNT7 | Polypeptide N-Acetylgalactosaminyltransferase 7 |
| AGTRAP | Angiotensin II Receptor Associated Protein |
| MAF1 | MAF1 Homolog, Negative Regulator Of RNA Polymerase III |
| SGTA | Small Glutamine Rich Tetratricopeptide Repeat Containing Alpha |
| NDUFA4L2 | NDUFA4 Mitochondrial Complex Associated Like 2 |
| TGOLN2 | Trans-Golgi Network Protein 2 |
| NAV1 | Neuron Navigator 1 |
| OSBPL9 | Oxysterol Binding Protein Like 9 |
| GCM1 | Glial Cells Missing Transcription Factor 1 |
| ZC4H2 | Zinc Finger C4H2-Type Containing |
| ZNF281 | Zinc Finger Protein 281 |
| GRSF1 | G-Rich RNA Sequence Binding Factor 1 |
| THAP11 | THAP Domain Containing 11 |
| SETD3 | SET Domain Containing 3, Actin Histidine Methyltransferase |
| SH2D4A | SH2 Domain Containing 4A |
| PTDSS2 | Phosphatidylserine Synthase 2 |
| KMT5A | Lysine Methyltransferase 5A |
| TMTC3 | Transmembrane O-Mannosyltransferase Targeting Cadherins 3 |
| STAC3 | SH3 And Cysteine Rich Domain 3 |
| COL22A1 | Collagen Type XXII Alpha 1 Chain |
| COQ10B | Coenzyme Q10B |
| MRPL46 | Mitochondrial Ribosomal Protein L46 |
| MRPL2 | Mitochondrial Ribosomal Protein L2 |
| MRPL20 | Mitochondrial Ribosomal Protein L20 |
| MRPL23 | Mitochondrial Ribosomal Protein L23 |
| CHCHD3 | Coiled-Coil-Helix-Coiled-Coil-Helix Domain Containing 3 |
| CHMP4A | Charged Multivesicular Body Protein 4A |
| DPEP3 | Dipeptidase 3 |
| NXPH4 | Neurexophilin 4 |
| FAAH2 | Fatty Acid Amide Hydrolase 2 |
| C11orf58 | Chromosome 11 Open Reading Frame 58 |
| BOLL | Boule Homolog, RNA Binding Protein |
| TSPYL2 | TSPY Like 2 |
| TSSK6 | Testis Specific Serine Kinase 6 |
| SGSM2 | Small G Protein Signaling Modulator 2 |
| KCNV1 | Potassium Voltage-Gated Channel Modifier Subfamily V Member 1 |
| PITPNM2 | Phosphatidylinositol Transfer Protein Membrane Associated 2 |
| SERPINA9 | Serpin Family A Member 9 |
| RABEP2 | Rabaptin, RAB GTPase Binding Effector Protein 2 |
| PBX4 | PBX Homeobox 4 |
| TMEM50A | Transmembrane Protein 50A |
| DARS1 | Aspartyl-TRNA Synthetase 1 |
| MRPL37 | Mitochondrial Ribosomal Protein L37 |
| HOMEZ | Homeobox And Leucine Zipper Encoding |
| SBNO1 | Strawberry Notch Homolog 1 |
| EMP1 | Epithelial Membrane Protein 1 |
| ARHGAP39 | Rho GTPase Activating Protein 39 |
| ASPG | Asparaginase |
| MAGEB2 | MAGE Family Member B2 |
| MED8 | Mediator Complex Subunit 8 |
| KRT80 | Keratin 80 |
| YPEL3 | Yippee Like 3 |
| SLC38A7 | Solute Carrier Family 38 Member 7 |
| SYNE3 | Spectrin Repeat Containing Nuclear Envelope Family Member 3 |
| REM2 | RRAD And GEM Like GTPase 2 |
| RCN3 | Reticulocalbin 3 |
| PLEKHA2 | Pleckstrin Homology Domain Containing A2 |
| R3HDM2 | R3H Domain Containing 2 |
| STK32B | Serine/Threonine Kinase 32B |
| CILP2 | Cartilage Intermediate Layer Protein 2 |
| ABT1 | Activator Of Basal Transcription 1 |
| LUZP2 | Leucine Zipper Protein 2 |
| LRRCC1 | Leucine Rich Repeat And Coiled-Coil Centrosomal Protein 1 |
| ANKRD9 | Ankyrin Repeat Domain 9 |
| KRT72 | Keratin 72 |
| YTHDF3 | YTH N6-Methyladenosine RNA Binding Protein 3 |
| ZNF804A | Zinc Finger Protein 804A |
| ZNF410 | Zinc Finger Protein 410 |
| PABPC1L | Poly(A) Binding Protein Cytoplasmic 1 Like |
| INSM2 | INSM Transcriptional Repressor 2 |
| NAA35 | N-Alpha-Acetyltransferase 35, NatC Auxiliary Subunit |
| SCAF11 | SR-Related CTD Associated Factor 11 |
| MUSTN1 | Musculoskeletal, Embryonic Nuclear Protein 1 |
| OGFOD2 | 2-Oxoglutarate And Iron Dependent Oxygenase Domain Containing 2 |
| MED19 | Mediator Complex Subunit 19 |
| MAU2 | MAU2 Sister Chromatid Cohesion Factor |
| ACRBP | Acrosin Binding Protein |
| C15orf48 | Chromosome 15 Open Reading Frame 48 |
| WBP2NL | WBP2 N-Terminal Like |
| SMDT1 | Single-Pass Membrane Protein With Aspartate Rich Tail 1 |
| ENKD1 | Enkurin Domain Containing 1 |
| INO80E | INO80 Complex Subunit E |
| TMEM167A | Transmembrane Protein 167A |
| PRR12 | Proline Rich 12 |
| HDX | Highly Divergent Homeobox |
| H3-4 | H3.4 Histone |
| HEATR5A | HEAT Repeat Containing 5A |
| CHURC1 | Churchill Domain Containing 1 |
| DNAJB8 | DnaJ Heat Shock Protein Family (Hsp40) Member B8 |
| OGA | O-GlcNAcase |
| CCDC170 | Coiled-Coil Domain Containing 170 |
| FAM53C | Family With Sequence Similarity 53 Member C |
| EXOC3L4 | Exocyst Complex Component 3 Like 4 |
| ANKFN1 | Ankyrin Repeat And Fibronectin Type III Domain Containing 1 |
| TM9SF1 | Transmembrane 9 Superfamily Member 1 |
| KHNYN | KH And NYN Domain Containing |
| SLCO5A1 | Solute Carrier Organic Anion Transporter Family Member 5A1 |
| ZSCAN2 | Zinc Finger And SCAN Domain Containing 2 |
| PLPP2 | Phospholipid Phosphatase 2 |
| PRRG2 | Proline Rich And Gla Domain 2 |
| MIS18BP1 | MIS18 Binding Protein 1 |
| NCCRP1 | NCCRP1, F-Box Associated Domain Containing |
| SCAF1 | SR-Related CTD Associated Factor 1 |
| RTL1 | Retrotransposon Gag Like 1 |
| ARL16 | ADP Ribosylation Factor Like GTPase 16 |
| ASPHD1 | Aspartate Beta-Hydroxylase Domain Containing 1 |
| TRANK1 | Tetratricopeptide Repeat And Ankyrin Repeat Containing 1 |
| LINS1 | Lines Homolog 1 |
| NRN1L | Neuritin 1 Like |
| TBC1D31 | TBC1 Domain Family Member 31 |
| SYT8 | Synaptotagmin 8 |
| MROH1 | Maestro Heat Like Repeat Family Member 1 |
| SEPTIN3 | Septin 3 |
| FSBP | Fibrinogen Silencer Binding Protein |
| LRRIQ3 | Leucine Rich Repeats And IQ Motif Containing 3 |
| CCT8L2 | Chaperonin Containing TCP1 Subunit 8 Like 2 |
| ZDHHC22 | Zinc Finger DHHC-Type Palmitoyltransferase 22 |
| WBP1L | WW Domain Binding Protein 1 Like |
| MSANTD2 | Myb/SANT DNA Binding Domain Containing 2 |
| C4orf17 | Chromosome 4 Open Reading Frame 17 |
| TM6SF2 | Transmembrane 6 Superfamily Member 2 |
| C16orf86 | Chromosome 16 Open Reading Frame 86 |
| FBXO48 | F-Box Protein 48 |
| C11orf87 | Chromosome 11 Open Reading Frame 87 |
| C1orf54 | Chromosome 1 Open Reading Frame 54 |
| ZNRF4 | Zinc And Ring Finger 4 |
| SHISA8 | Shisa Family Member 8 |
| H1-10 | H1.10 Linker Histone |
| BCL2L2-PABPN1 | BCL2L2-PABPN1 Readthrough |
| C12orf42 | Chromosome 12 Open Reading Frame 42 |
| LRRC69 | Leucine Rich Repeat Containing 69 |
| CT83 | Cancer/Testis Antigen 83 |
| OCM2 | Oncomodulin 2 |
| CCDC26 | CCDC26 Long Non-Coding RNA |
| C16orf92 | Chromosome 16 Open Reading Frame 92 |
| ANKRD63 | Ankyrin Repeat Domain 63 |
| SMIM4 | Small Integral Membrane Protein 4 |
| GOLGA6L4 | Golgin A6 Family Like 4 |
| ODR4 | Odr-4 GPCR Localization Factor Homolog |
| BORCS7 | BLOC-1 Related Complex Subunit 7 |
| TLCD3B | TLC Domain Containing 3B |
| POTEM | POTE Ankyrin Domain Family Member M |
| PHETA2 | PH Domain Containing Endocytic Trafficking Adaptor 2 |
| TDH | L-Threonine Dehydrogenase (Pseudogene) |
| C3orf49 | Chromosome 3 Open Reading Frame 49 |
| RNF212B | Ring Finger Protein 212B |
| BAGE2 | BAGE Family Member 2 |
| USP17L8 | Ubiquitin Specific Peptidase 17 Like Family Member 8 |
| HCG22 | HLA Complex Group 22 (Gene/Pseudogene) |
| USP17L1 | Ubiquitin Specific Peptidase 17 Like Family Member 1 |
| USP17L7 | Ubiquitin Specific Peptidase 17 Like Family Member 7 |
| USP17L3 | Ubiquitin Specific Peptidase 17 Like Family Member 3 |
| USP17L4 | Ubiquitin Specific Peptidase 17 Like Family Member 4 |
| FAM30A | Family With Sequence Similarity 30 Member A |
| TXLNGY | Taxilin Gamma Pseudogene, Y-Linked |
| MIR137HG | MIR137 Host Gene |
| LINC00862 | Long Intergenic Non-Protein Coding RNA 862 |
| FRMD6-AS1 | FRMD6 Antisense RNA 1 |
| TUNAR | TCL1 Upstream Neural Differentiation-Associated RNA |
| LINC01619 | Long Intergenic Non-Protein Coding RNA 1619 |
| CYP21A1P | Cytochrome P450 Family 21 Subfamily A Member 1, Pseudogene |
| FAM83H-AS1 | FAM83H Antisense RNA 1 (Head To Head) |
| KLKP1 | Kallikrein Pseudogene 1 |
| NRAV | Negative Regulator Of Antiviral Response |
| MIR29B2CHG | MIR29B2 And MIR29C Host Gene |
| BAIAP2-DT | BAIAP2 Divergent Transcript |
| GULOP | Gulonolactone (L-) Oxidase, Pseudogene |
| MIR650 | MicroRNA 650 |
| DEFB117 | Defensin Beta 117 (Pseudogene) |
| EP300-AS1 | EP300 Antisense RNA 1 |
| TNRC6C-AS1 | TNRC6C Antisense RNA 1 |
| WAKMAR2 | Wound And Keratinocyte Migration Associated LncRNA 2 |
| RB1-DT | RB1 Divergent Transcript |
| MROCKI | MARCKS Cis Regulating LncRNA Promoter Of Cytokines And Inflammation |
| BORCS7-ASMT | BORCS7-ASMT Readthrough (NMD Candidate) |
| LINC01470 | Long Intergenic Non-Protein Coding RNA 1470 |
| MIR6891 | MicroRNA 6891 |
| MIR548AJ2 | MicroRNA 548aj-2 |
| LYNX1-SLURP2 | LYNX1-SLURP2 Readthrough |
| TRAJ34 | T Cell Receptor Alpha Joining 34 |
| LINC02560 | Long Intergenic Non-Protein Coding RNA 2560 |
| LINC02561 | Long Intergenic Non-Protein Coding RNA 2561 |
| LINC02562 | Long Intergenic Non-Protein Coding RNA 2562 |
| TRAJ14 | T Cell Receptor Alpha Joining 14 |
| TRAJ9 | T Cell Receptor Alpha Joining 9 |
| DDIT4-AS1 | DDIT4 Antisense RNA 1 |
| MTCO2P12 | MT-CO2 Pseudogene 12 |
| BPIFB5P | BPI Fold Containing Family B Member 5, Pseudogene |
| ABCB5 | ATP Binding Cassette Subfamily B Member 5 |
| PTPDC1 | Protein Tyrosine Phosphatase Domain Containing 1 |
| TH2-LCR | Th2 Cytokine Locus Control Region |
| AURKB | Aurora Kinase B |
| KCNK3 | Potassium Two Pore Domain Channel Subfamily K Member 3 |
| ATP2A1 | ATPase Sarcoplasmic/Endoplasmic Reticulum Ca2+ Transporting 1 |
| KCNQ3 | Potassium Voltage-Gated Channel Subfamily Q Member 3 |
| CIT | Citron Rho-Interacting Serine/Threonine Kinase |
| DBI | Diazepam Binding Inhibitor, Acyl-CoA Binding Protein |
| ALDH6A1 | Aldehyde Dehydrogenase 6 Family Member A1 |
| DLC1 | DLC1 Rho GTPase Activating Protein |
| TFDP1 | Transcription Factor Dp-1 |
| CYP2C18 | Cytochrome P450 Family 2 Subfamily C Member 18 |
| ENDOG | Endonuclease G |
| KCNH5 | Potassium Voltage-Gated Channel Subfamily H Member 5 |
| GRB10 | Growth Factor Receptor Bound Protein 10 |
| CYP2S1 | Cytochrome P450 Family 2 Subfamily S Member 1 |
| DHX38 | DEAH-Box Helicase 38 |
| MTMR14 | Myotubularin Related Protein 14 |
| FBLN2 | Fibulin 2 |
| PLCZ1 | Phospholipase C Zeta 1 |
| SULT1A2 | Sulfotransferase Family 1A Member 2 |
| HEY1 | Hes Related Family BHLH Transcription Factor With YRPW Motif 1 |
| HCCS | Holocytochrome C Synthase |
| CHRNA6 | Cholinergic Receptor Nicotinic Alpha 6 Subunit |
| SALL2 | Spalt Like Transcription Factor 2 |
| GAPDHS | Glyceraldehyde-3-Phosphate Dehydrogenase, Spermatogenic |
| CA3 | Carbonic Anhydrase 3 |
| TOP1MT | DNA Topoisomerase I Mitochondrial |
| PDE6H | Phosphodiesterase 6H |
| GSTM2 | Glutathione S-Transferase Mu 2 |
| CYP46A1 | Cytochrome P450 Family 46 Subfamily A Member 1 |
| COX5B | Cytochrome C Oxidase Subunit 5B |
| KRT6B | Keratin 6B |
| WIPI2 | WD Repeat Domain, Phosphoinositide Interacting 2 |
| POLD3 | DNA Polymerase Delta 3, Accessory Subunit |
| SORBS3 | Sorbin And SH3 Domain Containing 3 |
| KIF13B | Kinesin Family Member 13B |
| STC2 | Stanniocalcin 2 |
| MYOCD | Myocardin |
| DDHD2 | DDHD Domain Containing 2 |
| CDKL1 | Cyclin Dependent Kinase Like 1 |
| CCDC88C | Coiled-Coil Domain Containing 88C |
| AKAP6 | A-Kinase Anchoring Protein 6 |
| ADAM11 | ADAM Metallopeptidase Domain 11 |
| BICD2 | BICD Cargo Adaptor 2 |
| FKBP2 | FKBP Prolyl Isomerase 2 |
| VSX2 | Visual System Homeobox 2 |
| OTUD6B | OTU Deubiquitinase 6B |
| SIX4 | SIX Homeobox 4 |
| KCNK10 | Potassium Two Pore Domain Channel Subfamily K Member 10 |
| RALGAPA1 | Ral GTPase Activating Protein Catalytic Subunit Alpha 1 |
| OXR1 | Oxidation Resistance 1 |
| SLC16A4 | Solute Carrier Family 16 Member 4 |
| PTPN21 | Protein Tyrosine Phosphatase Non-Receptor Type 21 |
| DACT1 | Dishevelled Binding Antagonist Of Beta Catenin 1 |
| MRPL13 | Mitochondrial Ribosomal Protein L13 |
| SFXN3 | Sideroflexin 3 |
| GMPR2 | Guanosine Monophosphate Reductase 2 |
| FRMD6 | FERM Domain Containing 6 |
| LSM1 | LSM1 Homolog, MRNA Degradation Associated |
| LRP12 | LDL Receptor Related Protein 12 |
| EPS8L2 | EPS8 Like 2 |
| ACYP1 | Acylphosphatase 1 |
| TPD52L1 | TPD52 Like 1 |
| KRT12 | Keratin 12 |
| VGF | VGF Nerve Growth Factor Inducible |
| ZFYVE26 | Zinc Finger FYVE-Type Containing 26 |
| SLC25A29 | Solute Carrier Family 25 Member 29 |
| ISCA2 | Iron-Sulfur Cluster Assembly 2 |
| NDUFB1 | NADH:Ubiquinone Oxidoreductase Subunit B1 |
| OSR2 | Odd-Skipped Related Transciption Factor 2 |
| REC8 | REC8 Meiotic Recombination Protein |
| PREX2 | Phosphatidylinositol-3,4,5-Trisphosphate Dependent Rac Exchange Factor 2 |
| SNF8 | SNF8 Subunit Of ESCRT-II |
| ITM2A | Integral Membrane Protein 2A |
| PAPLN | Papilin, Proteoglycan Like Sulfated Glycoprotein |
| TMEM106B | Transmembrane Protein 106B |
| HYAL3 | Hyaluronidase 3 |
| GPX5 | Glutathione Peroxidase 5 |
| RIN3 | Ras And Rab Interactor 3 |
| NCAM2 | Neural Cell Adhesion Molecule 2 |
| COX7A2 | Cytochrome C Oxidase Subunit 7A2 |
| CPNE6 | Copine 6 |
| DHRS2 | Dehydrogenase/Reductase 2 |
| S100A13 | S100 Calcium Binding Protein A13 |
| GOLGA5 | Golgin A5 |
| DLGAP5 | DLG Associated Protein 5 |
| MTMR7 | Myotubularin Related Protein 7 |
| MTMR9 | Myotubularin Related Protein 9 |
| RIMS2 | Regulating Synaptic Membrane Exocytosis 2 |
| CDCA2 | Cell Division Cycle Associated 2 |
| AJUBA | Ajuba LIM Protein |
| ABHD4 | Abhydrolase Domain Containing 4 |
| BRD8 | Bromodomain Containing 8 |
| ESCO1 | Establishment Of Sister Chromatid Cohesion N-Acetyltransferase 1 |
| AP4S1 | Adaptor Related Protein Complex 4 Subunit Sigma 1 |
| VASH1 | Vasohibin 1 |
| SLC25A27 | Solute Carrier Family 25 Member 27 |
| CREBZF | CREB/ATF BZIP Transcription Factor |
| ENOX2 | Ecto-NOX Disulfide-Thiol Exchanger 2 |
| SPIN1 | Spindlin 1 |
| E2F7 | E2F Transcription Factor 7 |
| DEGS2 | Delta 4-Desaturase, Sphingolipid 2 |
| DENND3 | DENN Domain Containing 3 |
| DLGAP2 | DLG Associated Protein 2 |
| CHMP7 | Charged Multivesicular Body Protein 7 |
| NPM2 | Nucleophosmin/Nucleoplasmin 2 |
| CD2BP2 | CD2 Cytoplasmic Tail Binding Protein 2 |
| ATXN2L | Ataxin 2 Like |
| CAPS | Calcyphosine |
| ACAP2 | ArfGAP With Coiled-Coil, Ankyrin Repeat And PH Domains 2 |
| MBIP | MAP3K12 Binding Inhibitory Protein 1 |
| ZFHX4 | Zinc Finger Homeobox 4 |
| U2AF2 | U2 Small Nuclear RNA Auxiliary Factor 2 |
| LGI3 | Leucine Rich Repeat LGI Family Member 3 |
| TSPAN1 | Tetraspanin 1 |
| COX16 | Cytochrome C Oxidase Assembly Factor COX16 |
| PARP10 | Poly(ADP-Ribose) Polymerase Family Member 10 |
| THTPA | Thiamine Triphosphatase |
| REEP4 | Receptor Accessory Protein 4 |
| THSD4 | Thrombospondin Type 1 Domain Containing 4 |
| KCNU1 | Potassium Calcium-Activated Channel Subfamily U Member 1 |
| PITPNC1 | Phosphatidylinositol Transfer Protein Cytoplasmic 1 |
| SNX16 | Sorting Nexin 16 |
| POMK | Protein O-Mannose Kinase |
| SLC22A17 | Solute Carrier Family 22 Member 17 |
| SSU72 | SSU72 Homolog, RNA Polymerase II CTD Phosphatase |
| STMN4 | Stathmin 4 |
| LSM6 | LSM6 Homolog, U6 Small Nuclear RNA And MRNA Degradation Associated |
| LSM7 | LSM7 Homolog, U6 Small Nuclear RNA And MRNA Degradation Associated |
| GSTT2 | Glutathione S-Transferase Theta 2 (Gene/Pseudogene) |
| CMTM5 | CKLF Like MARVEL Transmembrane Domain Containing 5 |
| RLF | Rearranged L-Myc Fusion |
| EBF2 | EBF Transcription Factor 2 |
| DHRS7 | Dehydrogenase/Reductase 7 |
| MRPL17 | Mitochondrial Ribosomal Protein L17 |
| MRPL39 | Mitochondrial Ribosomal Protein L39 |
| MTFR1 | Mitochondrial Fission Regulator 1 |
| SF3A2 | Splicing Factor 3a Subunit 2 |
| GPR135 | G Protein-Coupled Receptor 135 |
| RP1L1 | RP1 Like 1 |
| RPRD2 | Regulation Of Nuclear Pre-MRNA Domain Containing 2 |
| CDCA4 | Cell Division Cycle Associated 4 |
| FOXN3 | Forkhead Box N3 |
| AZIN2 | Antizyme Inhibitor 2 |
| CCDC25 | Coiled-Coil Domain Containing 25 |
| KRT77 | Keratin 77 |
| ZFYVE1 | Zinc Finger FYVE-Type Containing 1 |
| VPS37C | VPS37C Subunit Of ESCRT-I |
| U2SURP | U2 SnRNP Associated SURP Domain Containing |
| ZNF652 | Zinc Finger Protein 652 |
| ZNF397 | Zinc Finger Protein 397 |
| NDUFAF6 | NADH:Ubiquinone Oxidoreductase Complex Assembly Factor 6 |
| INTS9 | Integrator Complex Subunit 9 |
| POF1B | POF1B Actin Binding Protein |
| PLBD1 | Phospholipase B Domain Containing 1 |
| RALYL | RALY RNA Binding Protein Like |
| PCMTD1 | Protein-L-Isoaspartate (D-Aspartate) O-Methyltransferase Domain Containing 1 |
| RBM23 | RNA Binding Motif Protein 23 |
| SSNA1 | SS Nuclear Autoantigen 1 |
| PRPF39 | Pre-MRNA Processing Factor 39 |
| CLMN | Calmin |
| CINP | Cyclin Dependent Kinase 2 Interacting Protein |
| CNTNAP3 | Contactin Associated Protein Family Member 3 |
| RGS22 | Regulator Of G Protein Signaling 22 |
| HHIPL1 | HHIP Like 1 |
| HAUS1 | HAUS Augmin Like Complex Subunit 1 |
| MRPL16 | Mitochondrial Ribosomal Protein L16 |
| MRPL21 | Mitochondrial Ribosomal Protein L21 |
| DDX19A | DEAD-Box Helicase 19A |
| MPHOSPH6 | M-Phase Phosphoprotein 6 |
| CEP97 | Centrosomal Protein 97 |
| DPF3 | Double PHD Fingers 3 |
| DNAJA4 | DnaJ Heat Shock Protein Family (Hsp40) Member A4 |
| MRPS30 | Mitochondrial Ribosomal Protein S30 |
| EMCN | Endomucin |
| RPS6KL1 | Ribosomal Protein S6 Kinase Like 1 |
| ADAM20 | ADAM Metallopeptidase Domain 20 |
| ADAM21 | ADAM Metallopeptidase Domain 21 |
| ADAM32 | ADAM Metallopeptidase Domain 32 |
| ADCK5 | AarF Domain Containing Kinase 5 |
| FAM120C | Family With Sequence Similarity 120C |
| FAM135B | Family With Sequence Similarity 135 Member B |
| FBXO43 | F-Box Protein 43 |
| ATG2B | Autophagy Related 2B |
| AAR2 | AAR2 Splicing Factor |
| KRT25 | Keratin 25 |
| KRT34 | Keratin 34 |
| TOX4 | TOX High Mobility Group Box Family Member 4 |
| KCTD5 | Potassium Channel Tetramerization Domain Containing 5 |
| KIFC2 | Kinesin Family Member C2 |
| LEPROTL1 | Leptin Receptor Overlapping Transcript Like 1 |
| WDR20 | WD Repeat Domain 20 |
| TTC5 | Tetratricopeptide Repeat Domain 5 |
| SLC38A6 | Solute Carrier Family 38 Member 6 |
| SLC39A9 | Solute Carrier Family 39 Member 9 |
| PDS5B | PDS5 Cohesin Associated Factor B |
| ZNF250 | Zinc Finger Protein 250 |
| TC2N | Tandem C2 Domains, Nuclear |
| KCTD2 | Potassium Channel Tetramerization Domain Containing 2 |
| HTRA4 | HtrA Serine Peptidase 4 |
| INTS1 | Integrator Complex Subunit 1 |
| TMEM63C | Transmembrane Protein 63C |
| STON2 | Stonin 2 |
| MAPK1IP1L | Mitogen-Activated Protein Kinase 1 Interacting Protein 1 Like |
| DHRS4L2 | Dehydrogenase/Reductase 4 Like 2 |
| SDR39U1 | Short Chain Dehydrogenase/Reductase Family 39U Member 1 |
| RPS4Y1 | Ribosomal Protein S4 Y-Linked 1 |
| ADM2 | Adrenomedullin 2 |
| FAM91A1 | Family With Sequence Similarity 91 Member A1 |
| ASIC5 | Acid Sensing Ion Channel Subunit Family Member 5 |
| FAM161B | FAM161 Centrosomal Protein B |
| FAM168A | Family With Sequence Similarity 168 Member A |
| BRMS1L | BRMS1 Like Transcriptional Repressor |
| ARMC1 | Armadillo Repeat Containing 1 |
| ARHGEF40 | Rho Guanine Nucleotide Exchange Factor 40 |
| ASXL3 | ASXL Transcriptional Regulator 3 |
| ATAD5 | ATPase Family AAA Domain Containing 5 |
| KRT27 | Keratin 27 |
| UTP23 | UTP23 Small Subunit Processome Component |
| UNC79 | Unc-79 Homolog, NALCN Channel Complex Subunit |
| ZC3H3 | Zinc Finger CCCH-Type Containing 3 |
| SLC45A4 | Solute Carrier Family 45 Member 4 |
| ZNF251 | Zinc Finger Protein 251 |
| NRBP2 | Nuclear Receptor Binding Protein 2 |
| RBM12B | RNA Binding Motif Protein 12B |
| KLHL38 | Kelch Like Family Member 38 |
| TMEM65 | Transmembrane Protein 65 |
| CASS4 | Cas Scaffold Protein Family Member 4 |
| C9orf78 | Chromosome 9 Open Reading Frame 78 |
| MTBP | MDM2 Binding Protein |
| MTERF3 | Mitochondrial Transcription Termination Factor 3 |
| NIPAL2 | NIPA Like Domain Containing 2 |
| EML5 | EMAP Like 5 |
| NYNRIN | NYN Domain And Retroviral Integrase Containing |
| FER1L6 | Fer-1 Like Family Member 6 |
| KRT84 | Keratin 84 |
| WDYHV1 | WDYHV Motif Containing 1 |
| TPRN | Taperin |
| SLFN14 | Schlafen Family Member 14 |
| PHF20L1 | PHD Finger Protein 20 Like 1 |
| ZNF517 | Zinc Finger Protein 517 |
| PGAM4 | Phosphoglycerate Mutase Family Member 4 |
| EFCAB1 | EF-Hand Calcium Binding Domain 1 |
| RBMXL2 | RBMX Like 2 |
| PLBD2 | Phospholipase B Domain Containing 2 |
| SPATC1 | Spermatogenesis And Centriole Associated 1 |
| ISM2 | Isthmin 2 |
| SYT16 | Synaptotagmin 16 |
| TMEM71 | Transmembrane Protein 71 |
| DRAM1 | DNA Damage Regulated Autophagy Modulator 1 |
| HAUS4 | HAUS Augmin Like Complex Subunit 4 |
| SBSN | Suprabasin |
| OR4D2 | Olfactory Receptor Family 4 Subfamily D Member 2 |
| GOT1L1 | Glutamic-Oxaloacetic Transaminase 1 Like 1 |
| GPATCH2L | G-Patch Domain Containing 2 Like |
| CEP170B | Centrosomal Protein 170B |
| DCAF11 | DDB1 And CUL4 Associated Factor 11 |
| NUDT14 | Nudix Hydrolase 14 |
| NUDT18 | Nudix Hydrolase 18 |
| SARAF | Store-Operated Calcium Entry Associated Regulatory Factor |
| ABHD12B | Abhydrolase Domain Containing 12B |
| EXD2 | Exonuclease 3'-5' Domain Containing 2 |
| FAM160B2 | Family With Sequence Similarity 160 Member B2 |
| C14orf39 | Chromosome 14 Open Reading Frame 39 |
| FBXO16 | F-Box Protein 16 |
| ANGEL1 | Angel Homolog 1 |
| ZFHX2 | Zinc Finger Homeobox 2 |
| ZC3H18 | Zinc Finger CCCH-Type Containing 18 |
| R3HCC1 | R3H Domain And Coiled-Coil Containing 1 |
| SPIN3 | Spindlin Family Member 3 |
| SPDL1 | Spindle Apparatus Coiled-Coil Protein 1 |
| PPP4R3A | Protein Phosphatase 4 Regulatory Subunit 3A |
| PPP1R42 | Protein Phosphatase 1 Regulatory Subunit 42 |
| TIMM21 | Translocase Of Inner Mitochondrial Membrane 21 |
| PRSS55 | Serine Protease 55 |
| MITD1 | Microtubule Interacting And Trafficking Domain Containing 1 |
| GPHB5 | Glycoprotein Hormone Subunit Beta 5 |
| MRPL58 | Mitochondrial Ribosomal Protein L58 |
| RMDN1 | Regulator Of Microtubule Dynamics 1 |
| OSGIN2 | Oxidative Stress Induced Growth Inhibitor Family Member 2 |
| SEMG2 | Semenogelin 2 |
| DTD2 | D-Aminoacyl-TRNA Deacylase 2 |
| CCN5 | Cellular Communication Network Factor 5 |
| FSCB | Fibrous Sheath CABYR Binding Protein |
| MCMBP | Minichromosome Maintenance Complex Binding Protein |
| BTBD7 | BTB Domain Containing 7 |
| C14orf93 | Chromosome 14 Open Reading Frame 93 |
| C8orf34 | Chromosome 8 Open Reading Frame 34 |
| LURAP1 | Leucine Rich Adaptor Protein 1 |
| WDR25 | WD Repeat Domain 25 |
| ZC2HC1C | Zinc Finger C2HC-Type Containing 1C |
| L3HYPDH | Trans-L-3-Hydroxyproline Dehydratase |
| SMIM19 | Small Integral Membrane Protein 19 |
| ZSCAN26 | Zinc Finger And SCAN Domain Containing 26 |
| ZNF596 | Zinc Finger Protein 596 |
| OR4K15 | Olfactory Receptor Family 4 Subfamily K Member 15 |
| SPINT4 | Serine Peptidase Inhibitor, Kunitz Type 4 |
| KPRP | Keratinocyte Proline Rich Protein |
| PROX2 | Prospero Homeobox 2 |
| CIPC | CLOCK Interacting Pacemaker |
| RFPL4A | Ret Finger Protein Like 4A |
| HHLA1 | HERV-H LTR-Associating 1 |
| SEPTIN6 | Septin 6 |
| CCDC87 | Coiled-Coil Domain Containing 87 |
| C8orf48 | Chromosome 8 Open Reading Frame 48 |
| C8orf74 | Chromosome 8 Open Reading Frame 74 |
| LRRC24 | Leucine Rich Repeat Containing 24 |
| TMED8 | Transmembrane P24 Trafficking Protein Family Member 8 |
| ZC2HC1A | Zinc Finger C2HC-Type Containing 1A |
| TIGD5 | Tigger Transposable Element Derived 5 |
| ZNF623 | Zinc Finger Protein 623 |
| NOXRED1 | NADP Dependent Oxidoreductase Domain Containing 1 |
| SHTN1 | Shootin 1 |
| KLHL33 | Kelch Like Family Member 33 |
| TMEM251 | Transmembrane Protein 251 |
| CMC4 | C-X9-C Motif Containing 4 |
| RIPOR2 | RHO Family Interacting Cell Polarization Regulator 2 |
| OR5AU1 | Olfactory Receptor Family 5 Subfamily AU Member 1 |
| NKX6-3 | NK6 Homeobox 3 |
| CCSER1 | Coiled-Coil Serine Rich Protein 1 |
| FAM86B1 | Family With Sequence Similarity 86 Member B1 |
| KRBA2 | KRAB-A Domain Containing 2 |
| PPP4R3B | Protein Phosphatase 4 Regulatory Subunit 3B |
| POTEG | POTE Ankyrin Domain Family Member G |
| SYNDIG1L | Synapse Differentiation Inducing 1 Like |
| TMEM179 | Transmembrane Protein 179 |
| IGIP | IgA Inducing Protein |
| HEATR4 | HEAT Repeat Containing 4 |
| OR11G2 | Olfactory Receptor Family 11 Subfamily G Member 2 |
| GPR137C | G Protein-Coupled Receptor 137C |
| OC90 | Otoconin 90 |
| SAMD15 | Sterile Alpha Motif Domain Containing 15 |
| MBOAT4 | Membrane Bound O-Acyltransferase Domain Containing 4 |
| MCMDC2 | Minichromosome Maintenance Domain Containing 2 |
| ETFBKMT | Electron Transfer Flavoprotein Subunit Beta Lysine Methyltransferase |
| FAM181A | Family With Sequence Similarity 181 Member A |
| C8orf76 | Chromosome 8 Open Reading Frame 76 |
| FAM86B2 | Family With Sequence Similarity 86 Member B2 |
| TRIM51 | Tripartite Motif-Containing 51 |
| SLC35F4 | Solute Carrier Family 35 Member F4 |
| OR4K17 | Olfactory Receptor Family 4 Subfamily K Member 17 |
| PRAG1 | PEAK1 Related, Kinase-Activating Pseudokinase 1 |
| PCNX1 | Pecanex 1 |
| MROH6 | Maestro Heat Like Repeat Family Member 6 |
| SCX | Scleraxis BHLH Transcription Factor |
| RSRP1 | Arginine And Serine Rich Protein 1 |
| ATP5MPL | ATP Synthase Membrane Subunit 6.8PL |
| C14orf178 | Chromosome 14 Open Reading Frame 178 |
| C8orf44 | Chromosome 8 Open Reading Frame 44 |
| LRRC74A | Leucine Rich Repeat Containing 74A |
| ERG28 | Ergosterol Biosynthesis 28 Homolog |
| ERICH5 | Glutamate Rich 5 |
| PRORP | Protein Only RNase P Catalytic Subunit |
| TCF24 | Transcription Factor 24 |
| PLEKHD1 | Pleckstrin Homology And Coiled-Coil Domain Containing D1 |
| POTEA | POTE Ankyrin Domain Family Member A |
| TMEM253 | Transmembrane Protein 253 |
| H2BC17 | H2B Clustered Histone 17 |
| DHRS4L1 | Dehydrogenase/Reductase 4 Like 1 |
| C8orf44-SGK3 | C8orf44-SGK3 Readthrough |
| TRIM49 | Tripartite Motif Containing 49 |
| TRMT9B | TRNA Methyltransferase 9B (Putative) |
| CCDC198 | Coiled-Coil Domain Containing 198 |
| RD3L | Retinal Degeneration 3 Like |
| PCNX4 | Pecanex 4 |
| TIMM23B | Translocase Of Inner Mitochondrial Membrane 23 Homolog B |
| RBIS | Ribosomal Biogenesis Factor |
| TEX22 | Testis Expressed 22 |
| MIDEAS | Mitotic Deacetylase Associated SANT Domain Protein |
| BBOF1 | Basal Body Orientation Factor 1 |
| CARMIL3 | Capping Protein Regulator And Myosin 1 Linker 3 |
| TMEM75 | Transmembrane Protein 75 |
| OR4F21 | Olfactory Receptor Family 4 Subfamily F Member 21 |
| CCDC166 | Coiled-Coil Domain Containing 166 |
| CCDC175 | Coiled-Coil Domain Containing 175 |
| ALKAL1 | ALK And LTK Ligand 1 |
| C14orf132 | Chromosome 14 Open Reading Frame 132 |
| LRRC9 | Leucine Rich Repeat Containing 9 |
| XKR5 | XK Related 5 |
| VXN | Vexin |
| TOGARAM1 | TOG Array Regulator Of Axonemal Microtubules 1 |
| TRIM49B | Tripartite Motif Containing 49B |
| PPDPFL | Pancreatic Progenitor Cell Differentiation And Proliferation Factor Like |
| CLBA1 | Clathrin Binding Box Of Aftiphilin Containing 1 |
| CCDC177 | Coiled-Coil Domain Containing 177 |
| TRIM49C | Tripartite Motif Containing 49C |
| TRIM64B | Tripartite Motif Containing 64B |
| SMIM18 | Small Integral Membrane Protein 18 |
| ZNF705G | Zinc Finger Protein 705G |
| TMEM249 | Transmembrane Protein 249 |
| CHURC1-FNTB | CHURC1-FNTB Readthrough |
| ADAM5 | ADAM Metallopeptidase Domain 5 (Pseudogene) |
| ANXA2P2 | Annexin A2 Pseudogene 2 |
| WDR97 | WD Repeat Domain 97 |
| TRIM64 | Tripartite Motif Containing 64 |
| TRIM77 | Tripartite Motif Containing 77 |
| MIR34C | MicroRNA 34c |
| MROH5 | Maestro Heat Like Repeat Family Member 5 (Gene/Pseudogene) |
| TRIM49D1 | Tripartite Motif Containing 49D1 |
| RHPN1-AS1 | RHPN1 Antisense RNA 1 (Head To Head) |
| NEDD8-MDP1 | NEDD8-MDP1 Readthrough |
| DHRS4-AS1 | DHRS4 Antisense RNA 1 |
| FAM167A-AS1 | FAM167A Antisense RNA 1 |
| C8orf87 | Chromosome 8 Open Reading Frame 87 |
| RBM12B-AS1 | RBM12B Antisense RNA 1 |
| C8orf88 | Chromosome 8 Open Reading Frame 88 |
| FER1L6-AS2 | FER1L6 Antisense RNA 2 |
| TPT1P8 | Tumor Protein, Translationally-Controlled 1 Pseudogene 8 |
| SYNJ2BP-COX16 | SYNJ2BP-COX16 Readthrough |
| PRR23D1 | Proline Rich 23 Domain Containing 1 |
| PRR23D2 | Proline Rich 23 Domain Containing 2 |
| OR11H7 | Olfactory Receptor Family 11 Subfamily H Member 7 (Gene/Pseudogene) |
| FAM87A | Family With Sequence Similarity 87 Member A |
| BAALC-AS2 | BAALC Antisense RNA 2 |
| LINC00588 | Long Intergenic Non-Protein Coding RNA 588 |
| ZNF252P-AS1 | ZNF252P Antisense RNA 1 |
| LINC01551 | Long Intergenic Non-Protein Coding RNA 1551 |
| LINC01599 | Long Intergenic Non-Protein Coding RNA 1599 |
| CCDC196 | Coiled-Coil Domain Containing 196 |
| CCDC197 | Coiled-Coil Domain Containing 197 |
| EIF2S3B | Eukaryotic Translation Initiation Factor 2 Subunit Gamma B |
| OR4K3 | Olfactory Receptor Family 4 Subfamily K Member 3 (Gene/Pseudogene) |
| OR4Q2 | Olfactory Receptor Family 4 Subfamily Q Member 2 (Gene/Pseudogene) |
| FAM90A20P | Family With Sequence Similarity 90 Member A20, Pseudogene |
| FAM90A2P | Family With Sequence Similarity 90 Member A2, Pseudogene |
| FAM90A7P | Family With Sequence Similarity 90 Member A7, Pseudogene |
| TRIM49D2 | Tripartite Motif Containing 49D2 |
| LINC00596 | Long Intergenic Non-Protein Coding RNA 596 |
| FAM90A10P | Family With Sequence Similarity 90 Member A10, Pseudogene |
| SEC11B | SEC11 Homolog B, Signal Peptidase Complex Subunit (Pseudogene) |
| OR4E1 | Olfactory Receptor Family 4 Subfamily E Member 1 (Gene/Pseudogene) |
| FAM90A5P | Family With Sequence Similarity 90 Member A5, Pseudogene |
| PRSS51 | Serine Protease 51 |
| CHMP4BP1 | Charged Multivesicular Body Protein 4B Pseudogene 1 |
| FOXN3-AS2 | FOXN3 Antisense RNA 2 |
| FAM90A13P | Family With Sequence Similarity 90 Member A13, Pseudogene |
| C8orf89 | Chromosome 8 Open Reading Frame 89 |
| ZHX1-C8orf76 | ZHX1-C8orf76 Readthrough |
| HSD3BP4 | Hydroxy-Delta-5-Steroid Dehydrogenase, 3 Beta, Pseudogene 4 |
| REXO1L2P | REXO1 Like 2, Pseudogene |
| IGHJ2 | Immunoglobulin Heavy Joining 2 |
| NAMPTP1 | Nicotinamide Phosphoribosyltransferase Pseudogene 1 |
| HRES1 | HTLV-1 Related Endogenous Sequence |
| SDR16C6P | Short Chain Dehydrogenase/Reductase Family 16C Member 6, Pseudogene |
| FAM90A12P | Family With Sequence Similarity 90 Member A12, Pseudogene |
| FAM90A15P | Family With Sequence Similarity 90 Member A15, Pseudogene |
| FAM90A9P | Family With Sequence Similarity 90 Member A9, Pseudogene |
| TRAJ17 | T Cell Receptor Alpha Joining 17 |
| TRIM53AP | Tripartite Motif Containing 53A, Pseudogene |
| LY6L | Lymphocyte Antigen 6 Family Member L |
| ENSG00000248235 |  |
| IQANK1 | IQ Motif And Ankyrin Repeat Containing 1 |
| IGHJ3 | Immunoglobulin Heavy Joining 3 |
| IGHJ4 | Immunoglobulin Heavy Joining 4 |
| FAM90A14P | Family With Sequence Similarity 90 Member A14, Pseudogene |
| FAM90A22P | Family With Sequence Similarity 90 Member A22, Pseudogene |
| FAM90A23P | Family With Sequence Similarity 90 Member A23, Pseudogene |
| FAM90A8P | Family With Sequence Similarity 90 Member A8, Pseudogene |
| ENSG00000256500 |  |
| TRAV8-5 | T Cell Receptor Alpha Variable 8-5 (Pseudogene) |
| TRBV21-1 | T Cell Receptor Beta Variable 21-1 (Pseudogene) |
| UBBP4 | Ubiquitin B Pseudogene 4 |
| IGHJ5 | Immunoglobulin Heavy Joining 5 |
| IGHJ6 | Immunoglobulin Heavy Joining 6 |
| SCAT1 | S-Phase Cancer Associated Transcript 1 |
| FAM90A16P | Family With Sequence Similarity 90 Member A16, Pseudogene |
| FAM90A18P | Family With Sequence Similarity 90 Member A18, Pseudogene |
| FAM90A24P | Family With Sequence Similarity 90 Member A24, Pseudogene |
| TRAJ23 | T Cell Receptor Alpha Joining 23 |
| TRAJ27 | T Cell Receptor Alpha Joining 27 |
| TRAJ32 | T Cell Receptor Alpha Joining 32 |
| TRAJ37 | T Cell Receptor Alpha Joining 37 |
| TRAJ39 | T Cell Receptor Alpha Joining 39 |
| TRAJ4 | T Cell Receptor Alpha Joining 4 |
| TRAJ44 | T Cell Receptor Alpha Joining 44 |
| TRAJ49 | T Cell Receptor Alpha Joining 49 |
| TRAJ6 | T Cell Receptor Alpha Joining 6 |
| ENSG00000258466 |  |
| UGT1A | UDP Glucuronosyltransferase Family 1 Member A Complex Locus |
| LBHD2 | LBH Domain Containing 2 |
| LMLN2 | Leishmanolysin Like Peptidase 2 |
| IGHD3-3 | Immunoglobulin Heavy Diversity 3-3 |
| ENSG00000254692 |  |
| ENSG00000203546 |  |
| FAM90A17P | Family With Sequence Similarity 90 Member A17, Pseudogene |
| FAM90A19P | Family With Sequence Similarity 90 Member A19, Pseudogene |
| TRAJ10 | T Cell Receptor Alpha Joining 10 |
| TRAJ11 | T Cell Receptor Alpha Joining 11 |
| TRAJ13 | T Cell Receptor Alpha Joining 13 |
| TRAJ16 | T Cell Receptor Alpha Joining 16 |
| TRAJ19 | T Cell Receptor Alpha Joining 19 (Non-Functional) |
| TRAJ20 | T Cell Receptor Alpha Joining 20 |
| TRAJ21 | T Cell Receptor Alpha Joining 21 |
| TRAJ26 | T Cell Receptor Alpha Joining 26 |
| TRAJ28 | T Cell Receptor Alpha Joining 28 |
| TRAJ30 | T Cell Receptor Alpha Joining 30 |
| TRAJ31 | T Cell Receptor Alpha Joining 31 |
| TRAJ33 | T Cell Receptor Alpha Joining 33 |
| TRAJ35 | T Cell Receptor Alpha Joining 35 (Non-Functional) |
| TRAJ36 | T Cell Receptor Alpha Joining 36 |
| TRAJ38 | T Cell Receptor Alpha Joining 38 |
| TRAJ40 | T Cell Receptor Alpha Joining 40 |
| TRAJ41 | T Cell Receptor Alpha Joining 41 |
| TRAJ43 | T Cell Receptor Alpha Joining 43 |
| TRAJ45 | T Cell Receptor Alpha Joining 45 |
| TRAJ46 | T Cell Receptor Alpha Joining 46 |
| TRAJ47 | T Cell Receptor Alpha Joining 47 |
| TRAJ48 | T Cell Receptor Alpha Joining 48 |
| TRAJ5 | T Cell Receptor Alpha Joining 5 |
| TRAJ50 | T Cell Receptor Alpha Joining 50 |
| TRAJ52 | T Cell Receptor Alpha Joining 52 |
| TRAJ53 | T Cell Receptor Alpha Joining 53 |
| TRAJ54 | T Cell Receptor Alpha Joining 54 |
| TRAJ56 | T Cell Receptor Alpha Joining 56 |
| TRAJ57 | T Cell Receptor Alpha Joining 57 |
| TRAJ59 | T Cell Receptor Alpha Joining 59 (Non-Functional) |
| TRAJ61 | T Cell Receptor Alpha Joining 61 (Non-Functional) |
| TRAJ7 | T Cell Receptor Alpha Joining 7 |
| ENSG00000258989 |  |
| ENSG00000259132 |  |
| TRAV8-7 | T Cell Receptor Alpha Variable 8-7 (Non-Functional) |
| TRDJ3 | T Cell Receptor Delta Joining 3 |
| ENSG00000258677 |  |
| ENSG00000257341 |  |
| ENSG00000276418 |  |
| LINC02224 | Long Intergenic Non-Protein Coding RNA 2224 |
| ENSG00000259164 |  |
| TRIM51GP | Tripartite Motif-Containing 51G, Pseudogene |
| ZNF705F | Zinc Finger Protein 705F |
| NF1P6 | Neurofibromin 1 Pseudogene 6 |
| ENSG00000259060 |  |
| ENSG00000259066 |  |
| ENSG00000258417 |  |
| TRDJ2 | T Cell Receptor Delta Joining 2 |
| TRDJ4 | T Cell Receptor Delta Joining 4 |
| ENSG00000258653 |  |
| ENSG00000259171 |  |
| TRIM51JP | Tripartite Motif-Containing 51J, Pseudogene |
| TRIM53CP | Tripartite Motif Containing 53C, Pseudogene |
| TRIM64DP | Tripartite Motif Containing 64D, Pseudogene |
| TRIM64EP | Tripartite Motif Containing 64E, Pseudogene |
| ENSG00000259371 |  |
| ENSG00000259522 |  |
| ENSG00000259529 |  |
| ENSG00000264668 |  |
| ENSG00000226490 |  |
| IGHD5-12 | Immunoglobulin Heavy Diversity 5-12 |
| IGHD6-13 | Immunoglobulin Heavy Diversity 6-13 |
| IGHD6-19 | Immunoglobulin Heavy Diversity 6-19 |
| NF1P4 | Neurofibromin 1 Pseudogene 4 |
| MAGOH3P | Mago Homolog 3, Pseudogene |
| ENSG00000283239 |  |
| ENSG00000258691 |  |
| TRIM51CP | Tripartite Motif-Containing 51C, Pseudogene |
| TRIM64FP | Tripartite Motif Containing 64F, Pseudogene |
| IGHD3-10 | Immunoglobulin Heavy Diversity 3-10 |
| IGHD3-9 | Immunoglobulin Heavy Diversity 3-9 |
| ENSG00000254673 |  |
| DEFB109C | Defensin Beta 109C |
| RRAGAP1 | Ras Related GTP Binding A Pseudogene 1 |
| ENSG00000285655 |  |
| ENSG00000285982 |  |
| ENSG00000286131 |  |
| ENSG00000285880 |  |
| ENSG00000273259 |  |
| LOC100132229 | Nuclear Pore Associated Protein 1 Pseudogene |
| ENSG00000285620 |  |
| ENSG00000284956 |  |
| LOC642414 | Tripartite Motif Containing 64B Pseudogene |
| LOC642579 | Tripartite Motif Containing 64B Pseudogene |
| ENSG00000285720 |  |
| LOC112268391 | FAM90A Pseudogene |
| LOC112268392 | FAM90A Pseudogene |
| LOC112268393 | FAM90A Pseudogene |
| ENSG00000271698 |  |
| ENSG00000285657 |  |
| ENSG00000285687 |  |
| ENSG00000285975 |  |
| ENSG00000285765 |  |
| ENSG00000285814 |  |
| ENSG00000285913 |  |
| LOC646506 | Lysosomal Associated Membrane Protein 1 Pseudogene |
| ENSG00000285937 |  |
| ENSG00000285950 |  |
| LOC102725023 | Killer Cell Immunoglobulin-Like Receptor 2DS3-Like |
| LOC112637023 | ABO Upstream Enhancer |
| INPP5A | Inositol Polyphosphate-5-Phosphatase A |
| SLC2A6 | Solute Carrier Family 2 Member 6 |
| CAPSL | Calcyphosine Like |
| LINC01100 | Long Intergenic Non-Protein Coding RNA 1100 |
| RPL13P6 | Ribosomal Protein L13 Pseudogene 6 |
| RPL24P5 | Ribosomal Protein L24 Pseudogene 5 |
| SDC2 | Syndecan 2 |
| MAN1A1 | Mannosidase Alpha Class 1A Member 1 |
| NPTX1 | Neuronal Pentraxin 1 |
| CPEB4 | Cytoplasmic Polyadenylation Element Binding Protein 4 |
| TMEM176A | Transmembrane Protein 176A |
| SLIT2 | Slit Guidance Ligand 2 |
| BLVRB | Biliverdin Reductase B |
| DDX3Y | DEAD-Box Helicase 3 Y-Linked |
| CRISP1 | Cysteine Rich Secretory Protein 1 |
| TSBP1 | Testis Expressed Basic Protein 1 |
| MIR101-1 | MicroRNA 101-1 |
| GLRX | Glutaredoxin |
| GTF2H1 | General Transcription Factor IIH Subunit 1 |
| CREB5 | CAMP Responsive Element Binding Protein 5 |
| AKAP1 | A-Kinase Anchoring Protein 1 |
| MIA | MIA SH3 Domain Containing |
| GANC | Glucosidase Alpha, Neutral C |
| TFPT | TCF3 Fusion Partner |
| C22orf39 | Chromosome 22 Open Reading Frame 39 |
| TTK | TTK Protein Kinase |
| PKN2 | Protein Kinase N2 |
| PLAA | Phospholipase A2 Activating Protein |
| MAN1A2 | Mannosidase Alpha Class 1A Member 2 |
| GBA2 | Glucosylceramidase Beta 2 |
| TNR | Tenascin R |
| PLXNA3 | Plexin A3 |
| MAN1C1 | Mannosidase Alpha Class 1C Member 1 |
| HRK | Harakiri, BCL2 Interacting Protein |
| DDX39A | DExD-Box Helicase 39A |
| LNX2 | Ligand Of Numb-Protein X 2 |
| GBA3 | Glucosylceramidase Beta 3 (Gene/Pseudogene) |
| CCDC134 | Coiled-Coil Domain Containing 134 |
| GOLGA3 | Golgin A3 |
| MAGEC2 | MAGE Family Member C2 |
| L1TD1 | LINE1 Type Transposase Domain Containing 1 |
| OTOL1 | Otolin 1 |
| KIAA0040 | KIAA0040 |
| LY6G6E | Lymphocyte Antigen 6 Family Member G6E |
| LOC102724971 | Putative V-Set And Immunoglobulin Domain-Containing-Like Protein IGHV4OR15-8 |
| GRM1 | Glutamate Metabotropic Receptor 1 |
| HTR2B | 5-Hydroxytryptamine Receptor 2B |
| SLC22A1 | Solute Carrier Family 22 Member 1 |
| NCS1 | Neuronal Calcium Sensor 1 |
| SDC3 | Syndecan 3 |
| FIBP | FGF1 Intracellular Binding Protein |
| GTF2F2 | General Transcription Factor IIF Subunit 2 |
| AGPAT1 | 1-Acylglycerol-3-Phosphate O-Acyltransferase 1 |
| RGN | Regucalcin |
| CPSF4 | Cleavage And Polyadenylation Specific Factor 4 |
| MED1 | Mediator Complex Subunit 1 |
| GTF2F1 | General Transcription Factor IIF Subunit 1 |
| CSTF3 | Cleavage Stimulation Factor Subunit 3 |
| ZNRD1 | Zinc Ribbon Domain Containing 1 |
| KCNIP3 | Potassium Voltage-Gated Channel Interacting Protein 3 |
| STAP1 | Signal Transducing Adaptor Family Member 1 |
| MT1E | Metallothionein 1E |
| MGAT4A | Alpha-1,3-Mannosyl-Glycoprotein 4-Beta-N-Acetylglucosaminyltransferase A |
| MGAT4C | MGAT4 Family Member C |
| NSMCE1 | NSE1 Homolog, SMC5-SMC6 Complex Component |
| ZG16B | Zymogen Granule Protein 16B |
| RBM27 | RNA Binding Motif Protein 27 |
| FDCSP | Follicular Dendritic Cell Secreted Protein |
| ENTR1 | Endosome Associated Trafficking Regulator 1 |
| FAM215A | Family With Sequence Similarity 215 Member A |
| MSBP1 | Minisatellite Binding Protein 1 |
| RDH5 | Retinol Dehydrogenase 5 |
| GNPAT | Glyceronephosphate O-Acyltransferase |
| ATP2B2 | ATPase Plasma Membrane Ca2+ Transporting 2 |
| SULT1E1 | Sulfotransferase Family 1E Member 1 |
| AOX1 | Aldehyde Oxidase 1 |
| SV2A | Synaptic Vesicle Glycoprotein 2A |
| CYP4B1 | Cytochrome P450 Family 4 Subfamily B Member 1 |
| CAPN9 | Calpain 9 |
| SLC16A7 | Solute Carrier Family 16 Member 7 |
| HSD17B7 | Hydroxysteroid 17-Beta Dehydrogenase 7 |
| FXYD2 | FXYD Domain Containing Ion Transport Regulator 2 |
| SULT1B1 | Sulfotransferase Family 1B Member 1 |
| FREM1 | FRAS1 Related Extracellular Matrix 1 |
| HOXB2 | Homeobox B2 |
| SDS | Serine Dehydratase |
| SCG3 | Secretogranin III |
| GLUD2 | Glutamate Dehydrogenase 2 |
| RPH3A | Rabphilin 3A |
| SPOCK1 | SPARC (Osteonectin), Cwcv And Kazal Like Domains Proteoglycan 1 |
| ITIH2 | Inter-Alpha-Trypsin Inhibitor Heavy Chain 2 |
| OVGP1 | Oviductal Glycoprotein 1 |
| CRYGS | Crystallin Gamma S |
| DTNB | Dystrobrevin Beta |
| HTRA3 | HtrA Serine Peptidase 3 |
| SNRPG | Small Nuclear Ribonucleoprotein Polypeptide G |
| LSM5 | LSM5 Homolog, U6 Small Nuclear RNA And MRNA Degradation Associated |
| CTR9 | CTR9 Homolog, Paf1/RNA Polymerase II Complex Component |
| TTF1 | Transcription Termination Factor 1 |
| AKAP4 | A-Kinase Anchoring Protein 4 |
| TMBIM6 | Transmembrane BAX Inhibitor Motif Containing 6 |
| CDH22 | Cadherin 22 |
| SNTB1 | Syntrophin Beta 1 |
| IGSF11 | Immunoglobulin Superfamily Member 11 |
| DNAJC17 | DnaJ Heat Shock Protein Family (Hsp40) Member C17 |
| CDC42EP1 | CDC42 Effector Protein 1 |
| CCNY | Cyclin Y |
| BHLHA15 | Basic Helix-Loop-Helix Family Member A15 |
| ATF7IP | Activating Transcription Factor 7 Interacting Protein |
| FBN3 | Fibrillin 3 |
| CARHSP1 | Calcium Regulated Heat Stable Protein 1 |
| TRHDE | Thyrotropin Releasing Hormone Degrading Enzyme |
| ZMYM4 | Zinc Finger MYM-Type Containing 4 |
| THUMPD1 | THUMP Domain Containing 1 |
| TBC1D10A | TBC1 Domain Family Member 10A |
| RALGPS2 | Ral GEF With PH Domain And SH3 Binding Motif 2 |
| SH3BP1 | SH3 Domain Binding Protein 1 |
| TESC | Tescalcin |
| MFAP1 | Microfibril Associated Protein 1 |
| ZDHHC20 | Zinc Finger DHHC-Type Palmitoyltransferase 20 |
| LBX1 | Ladybird Homeobox 1 |
| IER2 | Immediate Early Response 2 |
| KIF18B | Kinesin Family Member 18B |
| RTKN2 | Rhotekin 2 |
| MAL2 | Mal, T Cell Differentiation Protein 2 (Gene/Pseudogene) |
| STRIP1 | Striatin Interacting Protein 1 |
| ZFYVE19 | Zinc Finger FYVE-Type Containing 19 |
| ICE2 | Interactor Of Little Elongation Complex ELL Subunit 2 |
| QRICH2 | Glutamine Rich 2 |
| TMEM158 | Transmembrane Protein 158 (Gene/Pseudogene) |
| DPH3 | Diphthamide Biosynthesis 3 |
| SPATA9 | Spermatogenesis Associated 9 |
| TMEM40 | Transmembrane Protein 40 |
| OR1E2 | Olfactory Receptor Family 1 Subfamily E Member 2 |
| PRLH | Prolactin Releasing Hormone |
| MIR744 | MicroRNA 744 |
| MIR579 | MicroRNA 579 |
| MIR1291 | MicroRNA 1291 |
| ENSG00000263264 |  |
| ENSG00000280987 |  |
| ENSG00000198211 |  |
| RPL36P16 | Ribosomal Protein L36 Pseudogene 16 |
| IFIT1P1 | Interferon Induced Protein With Tetratricopeptide Repeats 1 Pseudogene 1 |
| HNP1 | Hypertensive Nephropathy |
| EGID-109951028 | A-Gamma-Globin 3' Regulatory Region |
| ACVR1B | Activin A Receptor Type 1B |
| ACO2 | Aconitase 2 |
| SYT1 | Synaptotagmin 1 |
| S100A10 | S100 Calcium Binding Protein A10 |
| PKP2 | Plakophilin 2 |
| MGLL | Monoglyceride Lipase |
| UGDH | UDP-Glucose 6-Dehydrogenase |
| NRF1 | Nuclear Respiratory Factor 1 |
| NDUFA2 | NADH:Ubiquinone Oxidoreductase Subunit A2 |
| DCLK1 | Doublecortin Like Kinase 1 |
| HSPB2 | Heat Shock Protein Family B (Small) Member 2 |
| HLF | HLF Transcription Factor, PAR BZIP Family Member |
| MT1X | Metallothionein 1X |
| SENP8 | SUMO Peptidase Family Member, NEDD8 Specific |
| ROBO4 | Roundabout Guidance Receptor 4 |
| ABR | ABR Activator Of RhoGEF And GTPase |
| MANF | Mesencephalic Astrocyte Derived Neurotrophic Factor |
| SLC44A4 | Solute Carrier Family 44 Member 4 |
| RBM5 | RNA Binding Motif Protein 5 |
| PEG3 | Paternally Expressed 3 |
| ITSN2 | Intersectin 2 |
| PDCD5 | Programmed Cell Death 5 |
| GUCA2B | Guanylate Cyclase Activator 2B |
| HDLBP | High Density Lipoprotein Binding Protein |
| RTKN | Rhotekin |
| PROKR1 | Prokineticin Receptor 1 |
| PIWIL2 | Piwi Like RNA-Mediated Gene Silencing 2 |
| OXER1 | Oxoeicosanoid Receptor 1 |
| HCAR2 | Hydroxycarboxylic Acid Receptor 2 |
| GCHFR | GTP Cyclohydrolase I Feedback Regulator |
| BRD9 | Bromodomain Containing 9 |
| FBXO3 | F-Box Protein 3 |
| ASF1A | Anti-Silencing Function 1A Histone Chaperone |
| WDR48 | WD Repeat Domain 48 |
| SHROOM3 | Shroom Family Member 3 |
| CCDC50 | Coiled-Coil Domain Containing 50 |
| AP1G2 | Adaptor Related Protein Complex 1 Subunit Gamma 2 |
| HSPBP1 | HSPA (Hsp70) Binding Protein 1 |
| CALCOCO1 | Calcium Binding And Coiled-Coil Domain 1 |
| AMY1A | Amylase Alpha 1A |
| TM9SF4 | Transmembrane 9 Superfamily Member 4 |
| RSF1 | Remodeling And Spacing Factor 1 |
| MAGEA6 | MAGE Family Member A6 |
| QPCTL | Glutaminyl-Peptide Cyclotransferase Like |
| ASTN1 | Astrotactin 1 |
| KCTD3 | Potassium Channel Tetramerization Domain Containing 3 |
| ELOF1 | Elongation Factor 1 Homolog |
| SLTM | SAFB Like Transcription Modulator |
| CSH2 | Chorionic Somatomammotropin Hormone 2 |
| MRTFA | Myocardin Related Transcription Factor A |
| LYZL6 | Lysozyme Like 6 |
| H4C14 | H4 Clustered Histone 14 |
| POGLUT2 | Protein O-Glucosyltransferase 2 |
| HCG4 | HLA Complex Group 4 |
| ABL2 | ABL Proto-Oncogene 2, Non-Receptor Tyrosine Kinase |
| SLC25A12 | Solute Carrier Family 25 Member 12 |
| PDE10A | Phosphodiesterase 10A |
| SYN1 | Synapsin I |
| DYRK2 | Dual Specificity Tyrosine Phosphorylation Regulated Kinase 2 |
| SURF1 | SURF1 Cytochrome C Oxidase Assembly Factor |
| RAD23A | RAD23 Homolog A, Nucleotide Excision Repair Protein |
| CAV2 | Caveolin 2 |
| METAP2 | Methionyl Aminopeptidase 2 |
| POLR2C | RNA Polymerase II Subunit C |
| OMG | Oligodendrocyte Myelin Glycoprotein |
| MTMR3 | Myotubularin Related Protein 3 |
| UGCG | UDP-Glucose Ceramide Glucosyltransferase |
| PTP4A1 | Protein Tyrosine Phosphatase 4A1 |
| CPD | Carboxypeptidase D |
| SHB | SH2 Domain Containing Adaptor Protein B |
| STK17A | Serine/Threonine Kinase 17a |
| HIP1R | Huntingtin Interacting Protein 1 Related |
| NFYB | Nuclear Transcription Factor Y Subunit Beta |
| DNAJA3 | DnaJ Heat Shock Protein Family (Hsp40) Member A3 |
| GALNT6 | Polypeptide N-Acetylgalactosaminyltransferase 6 |
| CCNC | Cyclin C |
| BVES | Blood Vessel Epicardial Substance |
| ARAP3 | ArfGAP With RhoGAP Domain, Ankyrin Repeat And PH Domain 3 |
| LIMS1 | LIM Zinc Finger Domain Containing 1 |
| TGIF2 | TGFB Induced Factor Homeobox 2 |
| KCNN1 | Potassium Calcium-Activated Channel Subfamily N Member 1 |
| PDLIM4 | PDZ And LIM Domain 4 |
| PLXNB2 | Plexin B2 |
| SIPA1L3 | Signal Induced Proliferation Associated 1 Like 3 |
| TAF5L | TATA-Box Binding Protein Associated Factor 5 Like |
| SYN2 | Synapsin II |
| DAZL | Deleted In Azoospermia Like |
| CSTF2 | Cleavage Stimulation Factor Subunit 2 |
| NFIC | Nuclear Factor I C |
| RNPEP | Arginyl Aminopeptidase |
| CST1 | Cystatin SN |
| MRVI1 | Murine Retrovirus Integration Site 1 Homolog |
| GABRP | Gamma-Aminobutyric Acid Type A Receptor Subunit Pi |
| MED4 | Mediator Complex Subunit 4 |
| USP32 | Ubiquitin Specific Peptidase 32 |
| PDIA2 | Protein Disulfide Isomerase Family A Member 2 |
| RBFOX2 | RNA Binding Fox-1 Homolog 2 |
| NPBWR1 | Neuropeptides B And W Receptor 1 |
| PPIL3 | Peptidylprolyl Isomerase Like 3 |
| MCM10 | Minichromosome Maintenance 10 Replication Initiation Factor |
| BTG2 | BTG Anti-Proliferation Factor 2 |
| PPP2R3B | Protein Phosphatase 2 Regulatory Subunit B''Beta |
| GTF2H2 | General Transcription Factor IIH Subunit 2 |
| ZGPAT | Zinc Finger CCCH-Type And G-Patch Domain Containing |
| PHACTR1 | Phosphatase And Actin Regulator 1 |
| NSMCE4A | NSE4 Homolog A, SMC5-SMC6 Complex Component |
| PHF3 | PHD Finger Protein 3 |
| TMSB10 | Thymosin Beta 10 |
| GUCA2A | Guanylate Cyclase Activator 2A |
| MT1H | Metallothionein 1H |
| SARNP | SAP Domain Containing Ribonucleoprotein |
| NUPR1 | Nuclear Protein 1, Transcriptional Regulator |
| CD3EAP | CD3e Molecule Associated Protein |
| BTBD10 | BTB Domain Containing 10 |
| MED7 | Mediator Complex Subunit 7 |
| KRT75 | Keratin 75 |
| LDOC1 | LDOC1 Regulator Of NFKB Signaling |
| ZNF85 | Zinc Finger Protein 85 |
| TCF19 | Transcription Factor 19 |
| SPPL3 | Signal Peptide Peptidase Like 3 |
| LY6G6C | Lymphocyte Antigen 6 Family Member G6C |
| NELFB | Negative Elongation Factor Complex Member B |
| GFOD1 | Glucose-Fructose Oxidoreductase Domain Containing 1 |
| PHTF1 | Putative Homeodomain Transcription Factor 1 |
| PAAF1 | Proteasomal ATPase Associated Factor 1 |
| CLEC3A | C-Type Lectin Domain Family 3 Member A |
| EIF2D | Eukaryotic Translation Initiation Factor 2D |
| MT1G | Metallothionein 1G |
| MRPS14 | Mitochondrial Ribosomal Protein S14 |
| NOL7 | Nucleolar Protein 7 |
| NUSAP1 | Nucleolar And Spindle Associated Protein 1 |
| PHACTR2 | Phosphatase And Actin Regulator 2 |
| TMEM176B | Transmembrane Protein 176B |
| ZBTB9 | Zinc Finger And BTB Domain Containing 9 |
| ELFN2 | Extracellular Leucine Rich Repeat And Fibronectin Type III Domain Containing 2 |
| SLFN5 | Schlafen Family Member 5 |
| GSDME | Gasdermin E |
| MROH7 | Maestro Heat Like Repeat Family Member 7 |
| NRDC | Nardilysin Convertase |
| LY6G5B | Lymphocyte Antigen 6 Family Member G5B |
| SFTA2 | Surfactant Associated 2 |
| H2BC1 | H2B Clustered Histone 1 |
| H2BC13 | H2B Clustered Histone 13 |
| SERPINA2 | Serpin Family A Member 2 (Gene/Pseudogene) |
| TNXA | Tenascin XA (Pseudogene) |
| HCG27 | HLA Complex Group 27 |
| HCG9 | HLA Complex Group 9 |
| MIR199A2 | MicroRNA 199a-2 |
| MIR492 | MicroRNA 492 |
| MIR135B | MicroRNA 135b |
| AFAP1-AS1 | AFAP1 Antisense RNA 1 |
| VTRNA2-1 | Vault RNA 2-1 |
| MIR525 | MicroRNA 525 |
| DEFB108D | Defensin Beta 108D (Pseudogene) |
| DEFB108E | Defensin Beta 108E (Pseudogene) |
| HEAT2 | HEart Disease Associated Transcript 2 |
| LOC107963951 | BCR-ABL Minor-Breakpoint Cluster Region |
| SPR | Sepiapterin Reductase |
| RRM2 | Ribonucleotide Reductase Regulatory Subunit M2 |
| SMO | Smoothened, Frizzled Class Receptor |
| GRIK2 | Glutamate Ionotropic Receptor Kainate Type Subunit 2 |
| MKNK1 | MAPK Interacting Serine/Threonine Kinase 1 |
| ALDH5A1 | Aldehyde Dehydrogenase 5 Family Member A1 |
| SERPIND1 | Serpin Family D Member 1 |
| P4HA2 | Prolyl 4-Hydroxylase Subunit Alpha 2 |
| SPRY2 | Sprouty RTK Signaling Antagonist 2 |
| CEL | Carboxyl Ester Lipase |
| TFAP2B | Transcription Factor AP-2 Beta |
| CASQ1 | Calsequestrin 1 |
| GFM1 | G Elongation Factor Mitochondrial 1 |
| SALL1 | Spalt Like Transcription Factor 1 |
| GGPS1 | Geranylgeranyl Diphosphate Synthase 1 |
| LPAR3 | Lysophosphatidic Acid Receptor 3 |
| AQP7 | Aquaporin 7 |
| WNT2 | Wnt Family Member 2 |
| RNASEH1 | Ribonuclease H1 |
| NME3 | NME/NM23 Nucleoside Diphosphate Kinase 3 |
| SLC22A3 | Solute Carrier Family 22 Member 3 |
| POLR1A | RNA Polymerase I Subunit A |
| SORL1 | Sortilin Related Receptor 1 |
| SULT1A1 | Sulfotransferase Family 1A Member 1 |
| PPP1R1B | Protein Phosphatase 1 Regulatory Inhibitor Subunit 1B |
| HMGCS1 | 3-Hydroxy-3-Methylglutaryl-CoA Synthase 1 |
| CRTC2 | CREB Regulated Transcription Coactivator 2 |
| HOXA10 | Homeobox A10 |
| AKR1C1 | Aldo-Keto Reductase Family 1 Member C1 |
| FABP7 | Fatty Acid Binding Protein 7 |
| ASRGL1 | Asparaginase And Isoaspartyl Peptidase 1 |
| TXNL4A | Thioredoxin Like 4A |
| NDUFA4 | NDUFA4 Mitochondrial Complex Associated |
| PIK3C2G | Phosphatidylinositol-4-Phosphate 3-Kinase Catalytic Subunit Type 2 Gamma |
| POLR2D | RNA Polymerase II Subunit D |
| POU3F2 | POU Class 3 Homeobox 2 |
| ITPKA | Inositol-Trisphosphate 3-Kinase A |
| PTPRT | Protein Tyrosine Phosphatase Receptor Type T |
| ADD3 | Adducin 3 |
| FGD4 | FYVE, RhoGEF And PH Domain Containing 4 |
| SLC4A7 | Solute Carrier Family 4 Member 7 |
| SMAD5 | SMAD Family Member 5 |
| CNN1 | Calponin 1 |
| CHRNA9 | Cholinergic Receptor Nicotinic Alpha 9 Subunit |
| BCAP31 | B Cell Receptor Associated Protein 31 |
| ENTPD3 | Ectonucleoside Triphosphate Diphosphohydrolase 3 |
| PTGR1 | Prostaglandin Reductase 1 |
| KDM2B | Lysine Demethylase 2B |
| POLD2 | DNA Polymerase Delta 2, Accessory Subunit |
| TBPL1 | TATA-Box Binding Protein Like 1 |
| PCMT1 | Protein-L-Isoaspartate (D-Aspartate) O-Methyltransferase |
| KIDINS220 | Kinase D Interacting Substrate 220 |
| KIF13A | Kinesin Family Member 13A |
| RGMA | Repulsive Guidance Molecule BMP Co-Receptor A |
| CRISP2 | Cysteine Rich Secretory Protein 2 |
| SCD5 | Stearoyl-CoA Desaturase 5 |
| FUT6 | Fucosyltransferase 6 |
| APLP1 | Amyloid Beta Precursor Like Protein 1 |
| ARF3 | ADP Ribosylation Factor 3 |
| TPST1 | Tyrosylprotein Sulfotransferase 1 |
| TPST2 | Tyrosylprotein Sulfotransferase 2 |
| TRA2A | Transformer 2 Alpha Homolog |
| UBQLN4 | Ubiquilin 4 |
| TSHZ1 | Teashirt Zinc Finger Homeobox 1 |
| PGK2 | Phosphoglycerate Kinase 2 |
| NLN | Neurolysin |
| PRC1 | Protein Regulator Of Cytokinesis 1 |
| RAB13 | RAB13, Member RAS Oncogene Family |
| TMEM98 | Transmembrane Protein 98 |
| GTF2E1 | General Transcription Factor IIE Subunit 1 |
| NACC1 | Nucleus Accumbens Associated 1 |
| COL8A1 | Collagen Type VIII Alpha 1 Chain |
| MICU1 | Mitochondrial Calcium Uptake 1 |
| GORASP1 | Golgi Reassembly Stacking Protein 1 |
| RPL37 | Ribosomal Protein L37 |
| NUCB2 | Nucleobindin 2 |
| OPN3 | Opsin 3 |
| RSRC1 | Arginine And Serine Rich Coiled-Coil 1 |
| CDC42EP3 | CDC42 Effector Protein 3 |
| METAP1 | Methionyl Aminopeptidase 1 |
| FOXL2 | Forkhead Box L2 |
| CABLES1 | Cdk5 And Abl Enzyme Substrate 1 |
| AANAT | Aralkylamine N-Acetyltransferase |
| USP48 | Ubiquitin Specific Peptidase 48 |
| WNK2 | WNK Lysine Deficient Protein Kinase 2 |
| PDIA5 | Protein Disulfide Isomerase Family A Member 5 |
| STK26 | Serine/Threonine Kinase 26 |
| TEAD3 | TEA Domain Transcription Factor 3 |
| CHML | CHM Like Rab Escort Protein |
| NUB1 | Negative Regulator Of Ubiquitin Like Proteins 1 |
| PITRM1 | Pitrilysin Metallopeptidase 1 |
| PTBP2 | Polypyrimidine Tract Binding Protein 2 |
| KLHL1 | Kelch Like Family Member 1 |
| MOAP1 | Modulator Of Apoptosis 1 |
| DAGLB | Diacylglycerol Lipase Beta |
| RNF40 | Ring Finger Protein 40 |
| RNPS1 | RNA Binding Protein With Serine Rich Domain 1 |
| MPP3 | Membrane Palmitoylated Protein 3 |
| ELOVL2 | ELOVL Fatty Acid Elongase 2 |
| ADGRG6 | Adhesion G Protein-Coupled Receptor G6 |
| BAIAP2L1 | BAR/IMD Domain Containing Adaptor Protein 2 Like 1 |
| ACBD3 | Acyl-CoA Binding Domain Containing 3 |
| ZNF202 | Zinc Finger Protein 202 |
| UBXN11 | UBX Domain Protein 11 |
| ZC3H11A | Zinc Finger CCCH-Type Containing 11A |
| TOE1 | Target Of EGR1, Exonuclease |
| PIGF | Phosphatidylinositol Glycan Anchor Biosynthesis Class F |
| SLC9A9 | Solute Carrier Family 9 Member A9 |
| PMFBP1 | Polyamine Modulated Factor 1 Binding Protein 1 |
| SNX17 | Sorting Nexin 17 |
| HTATSF1 | HIV-1 Tat Specific Factor 1 |
| GLTP | Glycolipid Transfer Protein |
| SAP30BP | SAP30 Binding Protein |
| RSPO3 | R-Spondin 3 |
| KIF6 | Kinesin Family Member 6 |
| ZNF184 | Zinc Finger Protein 184 |
| YOD1 | YOD1 Deubiquitinase |
| SHKBP1 | SH3KBP1 Binding Protein 1 |
| INTS7 | Integrator Complex Subunit 7 |
| THOC3 | THO Complex 3 |
| PPP1R12C | Protein Phosphatase 1 Regulatory Subunit 12C |
| PCGF1 | Polycomb Group Ring Finger 1 |
| TMTC2 | Transmembrane O-Mannosyltransferase Targeting Cadherins 2 |
| MYEF2 | Myelin Expression Factor 2 |
| CRTAC1 | Cartilage Acidic Protein 1 |
| DDX55 | DEAD-Box Helicase 55 |
| MRPL14 | Mitochondrial Ribosomal Protein L14 |
| RUSC2 | RUN And SH3 Domain Containing 2 |
| GCC2 | GRIP And Coiled-Coil Domain Containing 2 |
| MED21 | Mediator Complex Subunit 21 |
| CCNG2 | Cyclin G2 |
| FHL5 | Four And A Half LIM Domains 5 |
| ANO9 | Anoctamin 9 |
| ERGIC2 | ERGIC And Golgi 2 |
| UBLCP1 | Ubiquitin Like Domain Containing CTD Phosphatase 1 |
| USP26 | Ubiquitin Specific Peptidase 26 |
| SMPDL3A | Sphingomyelin Phosphodiesterase Acid Like 3A |
| PHF12 | PHD Finger Protein 12 |
| ZNF451 | Zinc Finger Protein 451 |
| RBMS3 | RNA Binding Motif Single Stranded Interacting Protein 3 |
| SPIRE1 | Spire Type Actin Nucleation Factor 1 |
| PLEKHA3 | Pleckstrin Homology Domain Containing A3 |
| PTBP3 | Polypyrimidine Tract Binding Protein 3 |
| PSD4 | Pleckstrin And Sec7 Domain Containing 4 |
| MDFI | MyoD Family Inhibitor |
| SF3B5 | Splicing Factor 3b Subunit 5 |
| GPATCH8 | G-Patch Domain Containing 8 |
| MED16 | Mediator Complex Subunit 16 |
| ACTL7A | Actin Like 7A |
| ADGRA3 | Adhesion G Protein-Coupled Receptor A3 |
| BEGAIN | Brain Enriched Guanylate Kinase Associated |
| FNBP4 | Formin Binding Protein 4 |
| ZNF131 | Zinc Finger Protein 131 |
| ZNRF3 | Zinc And Ring Finger 3 |
| SLCO6A1 | Solute Carrier Organic Anion Transporter Family Member 6A1 |
| EGFL8 | EGF Like Domain Multiple 8 |
| NRIP3 | Nuclear Receptor Interacting Protein 3 |
| KLHL18 | Kelch Like Family Member 18 |
| CNPY4 | Canopy FGF Signaling Regulator 4 |
| H3-3A | H3.3 Histone A |
| COMMD6 | COMM Domain Containing 6 |
| CORO7 | Coronin 7 |
| DMXL1 | Dmx Like 1 |
| OTUD3 | OTU Deubiquitinase 3 |
| LONRF2 | LON Peptidase N-Terminal Domain And Ring Finger 2 |
| BRI3BP | BRI3 Binding Protein |
| FBXL6 | F-Box And Leucine Rich Repeat Protein 6 |
| ANGPTL5 | Angiopoietin Like 5 |
| KLHL26 | Kelch Like Family Member 26 |
| TMEM147 | Transmembrane Protein 147 |
| UNC5CL | Unc-5 Family C-Terminal Like |
| WDFY4 | WDFY Family Member 4 |
| ZBTB2 | Zinc Finger And BTB Domain Containing 2 |
| DNAJC14 | DnaJ Heat Shock Protein Family (Hsp40) Member C14 |
| ACOXL | Acyl-CoA Oxidase Like |
| KLHL23 | Kelch Like Family Member 23 |
| ZFP91 | ZFP91 Zinc Finger Protein |
| ZC3H13 | Zinc Finger CCCH-Type Containing 13 |
| SUPT7L | SPT7 Like, STAGA Complex Subunit Gamma |
| PRKRIP1 | PRKR Interacting Protein 1 |
| TMED7 | Transmembrane P24 Trafficking Protein 7 |
| NUFIP2 | Nuclear FMR1 Interacting Protein 2 |
| CACUL1 | CDK2 Associated Cullin Domain 1 |
| ZNFX1 | Zinc Finger NFX1-Type Containing 1 |
| TIAF1 | TGFB1-Induced Anti-Apoptotic Factor 1 |
| SELENOS | Selenoprotein S |
| BABAM2 | BRISC And BRCA1 A Complex Member 2 |
| C1QTNF9B | C1q And TNF Related 9B |
| ELOA | Elongin A |
| H4C3 | H4 Clustered Histone 3 |
| C6orf118 | Chromosome 6 Open Reading Frame 118 |
| TMEM242 | Transmembrane Protein 242 |
| C10orf67 | Chromosome 10 Open Reading Frame 67 |
| CT55 | Cancer/Testis Antigen 55 |
| TUT4 | Terminal Uridylyl Transferase 4 |
| ZNF732 | Zinc Finger Protein 732 |
| LY6G5C | Lymphocyte Antigen 6 Family Member G5C |
| C10orf99 | Chromosome 10 Open Reading Frame 99 |
| POM121L2 | POM121 Transmembrane Nucleoporin Like 2 |
| SPANXB1 | SPANX Family Member B1 |
| GAGE2A | G Antigen 2A |
| ADGRF2 | Adhesion G Protein-Coupled Receptor F2 |
| TINCR | TINCR Ubiquitin Domain Containing |
| MIR181C | MicroRNA 181c |
| GAGE12I | G Antigen 12I |
| GAGE2C | G Antigen 2C |
| ADGRE4P | Adhesion G Protein-Coupled Receptor E4, Pseudogene |
| MIR9-2 | MicroRNA 9-2 |
| SCARNA5 | Small Cajal Body-Specific RNA 5 |
| ATP6V1G2-DDX39B | ATP6V1G2-DDX39B Readthrough (NMD Candidate) |
| MIR218-1 | MicroRNA 218-1 |
| IRF1-AS1 | IRF1 Antisense RNA 1 |
| PPT2-EGFL8 | PPT2-EGFL8 Readthrough (NMD Candidate) |
| MIR363 | MicroRNA 363 |
| MIR940 | MicroRNA 940 |
| ZNRD1ASP | Zinc Ribbon Domain Containing 1 Antisense, Pseudogene |
| LINC01139 | Long Intergenic Non-Protein Coding RNA 1139 |
| HCG26 | HLA Complex Group 26 |
| HLA-DRB9 | Major Histocompatibility Complex, Class II, DR Beta 9 (Pseudogene) |
| STAG3L5P-PVRIG2P-PILRB | STAG3L5P-PVRIG2P-PILRB Readthrough |
| LOC100506023 | Uncharacterized LOC100506023 |
| IGHV4-55 | Immunoglobulin Heavy Variable 4-55 (Pseudogene) |
| KAZN-AS1 | KAZN Antisense RNA 1 |
| NM | Neutrophil Migration |
| LINC02605 | Long Intergenic Non-Protein Coding RNA 2605 |
| IFNNP1 | Interferon Nu 1, Pseudogene |
| LOC110384692 | Complement C4A (Rodgers Blood Group)-Like |
| AD10 | Alzheimer Disease-10 |
| PNLIP | Pancreatic Lipase |
| NR2F1 | Nuclear Receptor Subfamily 2 Group F Member 1 |
| SLC12A2 | Solute Carrier Family 12 Member 2 |
| GRID2 | Glutamate Ionotropic Receptor Delta Type Subunit 2 |
| NDUFV1 | NADH:Ubiquinone Oxidoreductase Core Subunit V1 |
| DGKD | Diacylglycerol Kinase Delta |
| PDHB | Pyruvate Dehydrogenase E1 Subunit Beta |
| DAO | D-Amino Acid Oxidase |
| ACAD8 | Acyl-CoA Dehydrogenase Family Member 8 |
| NDUFA10 | NADH:Ubiquinone Oxidoreductase Subunit A10 |
| KCNJ4 | Potassium Inwardly Rectifying Channel Subfamily J Member 4 |
| SLC7A1 | Solute Carrier Family 7 Member 1 |
| MKNK2 | MAPK Interacting Serine/Threonine Kinase 2 |
| CYP4F2 | Cytochrome P450 Family 4 Subfamily F Member 2 |
| MYBPC1 | Myosin Binding Protein C1 |
| CHRM5 | Cholinergic Receptor Muscarinic 5 |
| GIT1 | GIT ArfGAP 1 |
| ATXN1 | Ataxin 1 |
| ARNT2 | Aryl Hydrocarbon Receptor Nuclear Translocator 2 |
| ASPA | Aspartoacylase |
| FABP3 | Fatty Acid Binding Protein 3 |
| TRIM24 | Tripartite Motif Containing 24 |
| PI4KB | Phosphatidylinositol 4-Kinase Beta |
| PMPCA | Peptidase, Mitochondrial Processing Subunit Alpha |
| SNCB | Synuclein Beta |
| TAF15 | TATA-Box Binding Protein Associated Factor 15 |
| QPRT | Quinolinate Phosphoribosyltransferase |
| TAOK1 | TAO Kinase 1 |
| IDI1 | Isopentenyl-Diphosphate Delta Isomerase 1 |
| MAP3K12 | Mitogen-Activated Protein Kinase Kinase Kinase 12 |
| CMPK1 | Cytidine/Uridine Monophosphate Kinase 1 |
| GRIK4 | Glutamate Ionotropic Receptor Kainate Type Subunit 4 |
| EED | Embryonic Ectoderm Development |
| HCRTR1 | Hypocretin Receptor 1 |
| MGAT1 | Alpha-1,3-Mannosyl-Glycoprotein 2-Beta-N-Acetylglucosaminyltransferase |
| GJC1 | Gap Junction Protein Gamma 1 |
| MEIS1 | Meis Homeobox 1 |
| ESD | Esterase D |
| PDE9A | Phosphodiesterase 9A |
| NR1D2 | Nuclear Receptor Subfamily 1 Group D Member 2 |
| P2RY4 | Pyrimidinergic Receptor P2Y4 |
| PMPCB | Peptidase, Mitochondrial Processing Subunit Beta |
| SNX5 | Sorting Nexin 5 |
| SERPINB8 | Serpin Family B Member 8 |
| KALRN | Kalirin RhoGEF Kinase |
| PARD3 | Par-3 Family Cell Polarity Regulator |
| RAB11B | RAB11B, Member RAS Oncogene Family |
| MAP3K13 | Mitogen-Activated Protein Kinase Kinase Kinase 13 |
| CNOT2 | CCR4-NOT Transcription Complex Subunit 2 |
| ELAVL2 | ELAV Like RNA Binding Protein 2 |
| NAPSA | Napsin A Aspartic Peptidase |
| COL9A3 | Collagen Type IX Alpha 3 Chain |
| SARDH | Sarcosine Dehydrogenase |
| MIPEP | Mitochondrial Intermediate Peptidase |
| DSTYK | Dual Serine/Threonine And Tyrosine Protein Kinase |
| RTN3 | Reticulon 3 |
| MED23 | Mediator Complex Subunit 23 |
| ME1 | Malic Enzyme 1 |
| BRS3 | Bombesin Receptor Subtype 3 |
| USP24 | Ubiquitin Specific Peptidase 24 |
| SLC38A1 | Solute Carrier Family 38 Member 1 |
| PGC | Progastricsin |
| SLIT1 | Slit Guidance Ligand 1 |
| PFKFB4 | 6-Phosphofructo-2-Kinase/Fructose-2,6-Biphosphatase 4 |
| KCNJ15 | Potassium Inwardly Rectifying Channel Subfamily J Member 15 |
| KCNH7 | Potassium Voltage-Gated Channel Subfamily H Member 7 |
| THOP1 | Thimet Oligopeptidase 1 |
| THBS3 | Thrombospondin 3 |
| POLR2J | RNA Polymerase II Subunit J |
| PUF60 | Poly(U) Binding Splicing Factor 60 |
| TLE2 | TLE Family Member 2, Transcriptional Corepressor |
| CLK2 | CDC Like Kinase 2 |
| NDUFV3 | NADH:Ubiquinone Oxidoreductase Subunit V3 |
| CRABP1 | Cellular Retinoic Acid Binding Protein 1 |
| MTA3 | Metastasis Associated 1 Family Member 3 |
| KIFC1 | Kinesin Family Member C1 |
| UAP1 | UDP-N-Acetylglucosamine Pyrophosphorylase 1 |
| CHM | CHM Rab Escort Protein |
| PRDM2 | PR/SET Domain 2 |
| SNAP91 | Synaptosome Associated Protein 91 |
| POLR1B | RNA Polymerase I Subunit B |
| SH3GLB1 | SH3 Domain Containing GRB2 Like, Endophilin B1 |
| PASK | PAS Domain Containing Serine/Threonine Kinase |
| SULF2 | Sulfatase 2 |
| TAF6 | TATA-Box Binding Protein Associated Factor 6 |
| ST8SIA4 | ST8 Alpha-N-Acetyl-Neuraminide Alpha-2,8-Sialyltransferase 4 |
| FMO2 | Flavin Containing Dimethylaniline Monoxygenase 2 |
| ELL | Elongation Factor For RNA Polymerase II |
| NDC80 | NDC80 Kinetochore Complex Component |
| RNF5 | Ring Finger Protein 5 |
| DMRT1 | Doublesex And Mab-3 Related Transcription Factor 1 |
| OSBPL5 | Oxysterol Binding Protein Like 5 |
| GPSM2 | G Protein Signaling Modulator 2 |
| CEP250 | Centrosomal Protein 250 |
| GIPC1 | GIPC PDZ Domain Containing Family Member 1 |
| ABCG4 | ATP Binding Cassette Subfamily G Member 4 |
| ADAMTS10 | ADAM Metallopeptidase With Thrombospondin Type 1 Motif 10 |
| BHLHE41 | Basic Helix-Loop-Helix Family Member E41 |
| CACNA2D3 | Calcium Voltage-Gated Channel Auxiliary Subunit Alpha2delta 3 |
| MAGI1 | Membrane Associated Guanylate Kinase, WW And PDZ Domain Containing 1 |
| FKBP10 | FKBP Prolyl Isomerase 10 |
| UGT1A6 | UDP Glucuronosyltransferase Family 1 Member A6 |
| SLCO3A1 | Solute Carrier Organic Anion Transporter Family Member 3A1 |
| KCND1 | Potassium Voltage-Gated Channel Subfamily D Member 1 |
| CWF19L1 | CWF19 Like Cell Cycle Control Factor 1 |
| DPYSL3 | Dihydropyrimidinase Like 3 |
| NDUFB4 | NADH:Ubiquinone Oxidoreductase Subunit B4 |
| SUPT3H | SPT3 Homolog, SAGA And STAGA Complex Component |
| KDELR2 | KDEL Endoplasmic Reticulum Protein Retention Receptor 2 |
| POLR2I | RNA Polymerase II Subunit I |
| SLC16A10 | Solute Carrier Family 16 Member 10 |
| ILKAP | ILK Associated Serine/Threonine Phosphatase |
| IP6K1 | Inositol Hexakisphosphate Kinase 1 |
| SSH2 | Slingshot Protein Phosphatase 2 |
| GTF2H3 | General Transcription Factor IIH Subunit 3 |
| CSTF2T | Cleavage Stimulation Factor Subunit 2 Tau Variant |
| RGS19 | Regulator Of G Protein Signaling 19 |
| ELF3 | E74 Like ETS Transcription Factor 3 |
| NCBP2 | Nuclear Cap Binding Protein Subunit 2 |
| COX17 | Cytochrome C Oxidase Copper Chaperone COX17 |
| CPSF3 | Cleavage And Polyadenylation Specific Factor 3 |
| MSTO1 | Misato Mitochondrial Distribution And Morphology Regulator 1 |
| MSC | Musculin |
| SEMA3B | Semaphorin 3B |
| SEMA4C | Semaphorin 4C |
| CES3 | Carboxylesterase 3 |
| CHMP1B | Charged Multivesicular Body Protein 1B |
| CHI3L2 | Chitinase 3 Like 2 |
| NLGN2 | Neuroligin 2 |
| CDK5RAP3 | CDK5 Regulatory Subunit Associated Protein 3 |
| CCT6B | Chaperonin Containing TCP1 Subunit 6B |
| MED14 | Mediator Complex Subunit 14 |
| CBLN1 | Cerebellin 1 Precursor |
| MAS1 | MAS1 Proto-Oncogene, G Protein-Coupled Receptor |
| MAST3 | Microtubule Associated Serine/Threonine Kinase 3 |
| ADGRG1 | Adhesion G Protein-Coupled Receptor G1 |
| FAM3C | FAM3 Metabolism Regulating Signaling Molecule C |
| FFAR4 | Free Fatty Acid Receptor 4 |
| ERGIC1 | Endoplasmic Reticulum-Golgi Intermediate Compartment 1 |
| AEBP1 | AE Binding Protein 1 |
| LIMA1 | LIM Domain And Actin Binding 1 |
| LDB2 | LIM Domain Binding 2 |
| LAPTM4B | Lysosomal Protein Transmembrane 4 Beta |
| EGFL6 | EGF Like Domain Multiple 6 |
| TAF10 | TATA-Box Binding Protein Associated Factor 10 |
| PRDM14 | PR/SET Domain 14 |
| PLXNA4 | Plexin A4 |
| PPM1L | Protein Phosphatase, Mg2+/Mn2+ Dependent 1L |
| IRX1 | Iroquois Homeobox 1 |
| TAX1BP3 | Tax1 Binding Protein 3 |
| ITGB1BP1 | Integrin Subunit Beta 1 Binding Protein 1 |
| SLC26A11 | Solute Carrier Family 26 Member 11 |
| TMPRSS11D | Transmembrane Serine Protease 11D |
| STARD10 | StAR Related Lipid Transfer Domain Containing 10 |
| MDGA1 | MAM Domain Containing Glycosylphosphatidylinositol Anchor 1 |
| CIR1 | Corepressor Interacting With RBPJ, 1 |
| HMCN1 | Hemicentin 1 |
| RGMB | Repulsive Guidance Molecule BMP Co-Receptor B |
| CUL9 | Cullin 9 |
| NAALAD2 | N-Acetylated Alpha-Linked Acidic Dipeptidase 2 |
| MSH4 | MutS Homolog 4 |
| MT1F | Metallothionein 1F |
| MOXD1 | Monooxygenase DBH Like 1 |
| MIA3 | MIA SH3 Domain ER Export Factor 3 |
| CHPF | Chondroitin Polymerizing Factor |
| GMIP | GEM Interacting Protein |
| NIPA1 | NIPA Magnesium Transporter 1 |
| SAP30 | Sin3A Associated Protein 30 |
| FRMD4A | FERM Domain Containing 4A |
| AKAP7 | A-Kinase Anchoring Protein 7 |
| ATF6B | Activating Transcription Factor 6 Beta |
| BRMS1 | BRMS1 Transcriptional Repressor And Anoikis Regulator |
| APBB3 | Amyloid Beta Precursor Protein Binding Family B Member 3 |
| ACOT13 | Acyl-CoA Thioesterase 13 |
| ASXL2 | ASXL Transcriptional Regulator 2 |
| TM4SF1 | Transmembrane 4 L Six Family Member 1 |
| ZKSCAN5 | Zinc Finger With KRAB And SCAN Domains 5 |
| TRPC4AP | Transient Receptor Potential Cation Channel Subfamily C Member 4 Associated Protein |
| SMNDC1 | Survival Motor Neuron Domain Containing 1 |
| ZNF436 | Zinc Finger Protein 436 |
| SUPT4H1 | SPT4 Homolog, DSIF Elongation Factor Subunit |
| GRPEL1 | GrpE Like 1, Mitochondrial |
| SYMPK | Symplekin |
| KCNK5 | Potassium Two Pore Domain Channel Subfamily K Member 5 |
| NRBF2 | Nuclear Receptor Binding Factor 2 |
| TAS1R1 | Taste 1 Receptor Member 1 |
| SLC22A16 | Solute Carrier Family 22 Member 16 |
| PCGF5 | Polycomb Group Ring Finger 5 |
| PCDH9 | Protocadherin 9 |
| C1QTNF1 | C1q And TNF Related 1 |
| CLN6 | CLN6 Transmembrane ER Protein |
| MKLN1 | Muskelin 1 |
| MOB4 | MOB Family Member 4, Phocein |
| SENP6 | SUMO Specific Peptidase 6 |
| DOK6 | Docking Protein 6 |
| NXPH1 | Neurexophilin 1 |
| MED22 | Mediator Complex Subunit 22 |
| MED24 | Mediator Complex Subunit 24 |
| FBXO5 | F-Box Protein 5 |
| ANKRD6 | Ankyrin Repeat Domain 6 |
| APOF | Apolipoprotein F |
| ZHX2 | Zinc Fingers And Homeoboxes 2 |
| SLC35C2 | Solute Carrier Family 35 Member C2 |
| SLC25A25 | Solute Carrier Family 25 Member 25 |
| SLC43A3 | Solute Carrier Family 43 Member 3 |
| SPRR1B | Small Proline Rich Protein 1B |
| PPFIA1 | PTPRF Interacting Protein Alpha 1 |
| HSPA13 | Heat Shock Protein Family A (Hsp70) Member 13 |
| SOX15 | SRY-Box Transcription Factor 15 |
| RAB33A | RAB33A, Member RAS Oncogene Family |
| RABAC1 | Rab Acceptor 1 |
| TBL1X | Transducin Beta Like 1 X-Linked |
| PDCD2 | Programmed Cell Death 2 |
| SIVA1 | SIVA1 Apoptosis Inducing Factor |
| TAF8 | TATA-Box Binding Protein Associated Factor 8 |
| RAB11FIP3 | RAB11 Family Interacting Protein 3 |
| SPCS3 | Signal Peptidase Complex Subunit 3 |
| STARD3NL | STARD3 N-Terminal Like |
| GTF2A2 | General Transcription Factor IIA Subunit 2 |
| GTF3C5 | General Transcription Factor IIIC Subunit 5 |
| CUTC | CutC Copper Transporter |
| EEFSEC | Eukaryotic Elongation Factor, Selenocysteine-TRNA Specific |
| NEURL1 | Neuralized E3 Ubiquitin Protein Ligase 1 |
| NAT9 | N-Acetyltransferase 9 (Putative) |
| CPSF2 | Cleavage And Polyadenylation Specific Factor 2 |
| COPS7B | COP9 Signalosome Subunit 7B |
| HS3ST4 | Heparan Sulfate-Glucosamine 3-Sulfotransferase 4 |
| SCAMP5 | Secretory Carrier Membrane Protein 5 |
| EIF1AY | Eukaryotic Translation Initiation Factor 1A Y-Linked |
| EIF1B | Eukaryotic Translation Initiation Factor 1B |
| MAPK8IP2 | Mitogen-Activated Protein Kinase 8 Interacting Protein 2 |
| CCNB3 | Cyclin B3 |
| MED27 | Mediator Complex Subunit 27 |
| FADS3 | Fatty Acid Desaturase 3 |
| FGD6 | FYVE, RhoGEF And PH Domain Containing 6 |
| ARHGAP27 | Rho GTPase Activating Protein 27 |
| EPB41L4B | Erythrocyte Membrane Protein Band 4.1 Like 4B |
| KIF26B | Kinesin Family Member 26B |
| ZDHHC14 | Zinc Finger DHHC-Type Palmitoyltransferase 14 |
| ZFAND3 | Zinc Finger AN1-Type Containing 3 |
| UBL3 | Ubiquitin Like 3 |
| WBP11 | WW Domain Binding Protein 11 |
| VPS18 | VPS18 Core Subunit Of CORVET And HOPS Complexes |
| L3MBTL3 | L3MBTL Histone Methyl-Lysine Binding Protein 3 |
| LAMTOR5 | Late Endosomal/Lysosomal Adaptor, MAPK And MTOR Activator 5 |
| TRMT112 | TRNA Methyltransferase Subunit 11-2 |
| ZNF300 | Zinc Finger Protein 300 |
| NOVA2 | NOVA Alternative Splicing Regulator 2 |
| TADA3 | Transcriptional Adaptor 3 |
| KCNE4 | Potassium Voltage-Gated Channel Subfamily E Regulatory Subunit 4 |
| SYNPO2 | Synaptopodin 2 |
| KIAA1217 | KIAA1217 |
| RBFOX3 | RNA Binding Fox-1 Homolog 3 |
| TMEM47 | Transmembrane Protein 47 |
| TMEM11 | Transmembrane Protein 11 |
| FRMPD2 | FERM And PDZ Domain Containing 2 |
| CILK1 | Ciliogenesis Associated Kinase 1 |
| NAA25 | N-Alpha-Acetyltransferase 25, NatB Auxiliary Subunit |
| COQ10A | Coenzyme Q10A |
| DCP1B | Decapping MRNA 1B |
| HOXD1 | Homeobox D1 |
| MT1M | Metallothionein 1M |
| MRPL48 | Mitochondrial Ribosomal Protein L48 |
| RTCA | RNA 3'-Terminal Phosphate Cyclase |
| OR2H2 | Olfactory Receptor Family 2 Subfamily H Member 2 |
| DNAJC9 | DnaJ Heat Shock Protein Family (Hsp40) Member C9 |
| DUSP15 | Dual Specificity Phosphatase 15 |
| RTN4RL2 | Reticulon 4 Receptor Like 2 |
| RPUSD4 | RNA Pseudouridine Synthase D4 |
| FAM83B | Family With Sequence Similarity 83 Member B |
| FAM107B | Family With Sequence Similarity 107 Member B |
| FEM1C | Fem-1 Homolog C |
| LZTS2 | Leucine Zipper Tumor Suppressor 2 |
| FEV | FEV Transcription Factor, ETS Family Member |
| CCDC14 | Coiled-Coil Domain Containing 14 |
| TJAP1 | Tight Junction Associated Protein 1 |
| ZDHHC19 | Zinc Finger DHHC-Type Palmitoyltransferase 19 |
| ZNF219 | Zinc Finger Protein 219 |
| UNC13C | Unc-13 Homolog C |
| VASH2 | Vasohibin 2 |
| ZBTB8A | Zinc Finger And BTB Domain Containing 8A |
| TRIM54 | Tripartite Motif Containing 54 |
| SLC37A2 | Solute Carrier Family 37 Member 2 |
| PDZRN4 | PDZ Domain Containing Ring Finger 4 |
| SLC35D2 | Solute Carrier Family 35 Member D2 |
| ZSCAN21 | Zinc Finger And SCAN Domain Containing 21 |
| ZRSR2 | Zinc Finger CCCH-Type, RNA Binding Motif And Serine/Arginine Rich 2 |
| ZXDC | ZXD Family Zinc Finger C |
| SYF2 | SYF2 Pre-MRNA Splicing Factor |
| RBM26 | RNA Binding Motif Protein 26 |
| ZRANB2 | Zinc Finger RANBP2-Type Containing 2 |
| ZBTB22 | Zinc Finger And BTB Domain Containing 22 |
| SFSWAP | Splicing Factor SWAP |
| PRICKLE3 | Prickle Planar Cell Polarity Protein 3 |
| SNN | Stannin |
| TBC1D8B | TBC1 Domain Family Member 8B |
| SUGP2 | SURP And G-Patch Domain Containing 2 |
| KLHL4 | Kelch Like Family Member 4 |
| KLHDC3 | Kelch Domain Containing 3 |
| TMEM123 | Transmembrane Protein 123 |
| GSTT2B | Glutathione S-Transferase Theta 2B (Gene/Pseudogene) |
| MMP26 | Matrix Metallopeptidase 26 |
| NGRN | Neugrin, Neurite Outgrowth Associated |
| NAP1L2 | Nucleosome Assembly Protein 1 Like 2 |
| HAUS8 | HAUS Augmin Like Complex Subunit 8 |
| HECW1 | HECT, C2 And WW Domain Containing E3 Ubiquitin Protein Ligase 1 |
| OSBPL10 | Oxysterol Binding Protein Like 10 |
| SERF2 | Small EDRK-Rich Factor 2 |
| RNASEK | Ribonuclease K |
| NUDCD3 | NudC Domain Containing 3 |
| CDC42SE2 | CDC42 Small Effector 2 |
| GLIPR1L1 | GLIPR1 Like 1 |
| MED26 | Mediator Complex Subunit 26 |
| FYTTD1 | Forty-Two-Three Domain Containing 1 |
| CBLN4 | Cerebellin 4 Precursor |
| FAM32A | Family With Sequence Similarity 32 Member A |
| CAPN15 | Calpain 15 |
| FKBP15 | FKBP Prolyl Isomerase 15 |
| FBXO33 | F-Box Protein 33 |
| MED10 | Mediator Complex Subunit 10 |
| KLHL29 | Kelch Like Family Member 29 |
| KIAA1841 | KIAA1841 |
| LIMD2 | LIM Domain Containing 2 |
| TWISTNB | TWIST Neighbor |
| YIPF4 | Yip1 Domain Family Member 4 |
| LGSN | Lengsin, Lens Protein With Glutamine Synthetase Domain |
| TPRA1 | Transmembrane Protein Adipocyte Associated 1 |
| TSSC4 | Tumor Suppressing Subtransferable Candidate 4 |
| PDZRN3 | PDZ Domain Containing Ring Finger 3 |
| CWC22 | CWC22 Spliceosome Associated Protein Homolog |
| THAP4 | THAP Domain Containing 4 |
| KCTD20 | Potassium Channel Tetramerization Domain Containing 20 |
| RBFA | Ribosome Binding Factor A |
| RAB40C | RAB40C, Member RAS Oncogene Family |
| ISM1 | Isthmin 1 |
| CNOT10 | CCR4-NOT Transcription Complex Subunit 10 |
| HMGN4 | High Mobility Group Nucleosomal Binding Domain 4 |
| MLIP | Muscular LMNA Interacting Protein |
| NAF1 | Nuclear Assembly Factor 1 Ribonucleoprotein |
| EBPL | EBP Like |
| H3-3B | H3.3 Histone B |
| CEP85 | Centrosomal Protein 85 |
| RNF121 | Ring Finger Protein 121 |
| COP1 | COP1 E3 Ubiquitin Ligase |
| AIDA | Axin Interactor, Dorsalization Associated |
| ACTR6 | Actin Related Protein 6 |
| ADAT2 | Adenosine Deaminase TRNA Specific 2 |
| ADGRF4 | Adhesion G Protein-Coupled Receptor F4 |
| C6orf89 | Chromosome 6 Open Reading Frame 89 |
| LRRC3B | Leucine Rich Repeat Containing 3B |
| FNDC1 | Fibronectin Type III Domain Containing 1 |
| TULP4 | TUB Like Protein 4 |
| WDR70 | WD Repeat Domain 70 |
| ZNF804B | Zinc Finger Protein 804B |
| POLR2M | RNA Polymerase II Subunit M |
| SERPINB11 | Serpin Family B Member 11 (Gene/Pseudogene) |
| TCEANC2 | Transcription Elongation Factor A N-Terminal And Central Domain Containing 2 |
| KLHL35 | Kelch Like Family Member 35 |
| SPESP1 | Sperm Equatorial Segment Protein 1 |
| PRPF38B | Pre-MRNA Processing Factor 38B |
| PRRT1 | Proline Rich Transmembrane Protein 1 |
| CLASRP | CLK4 Associating Serine/Arginine Rich Protein |
| HEPACAM2 | HEPACAM Family Member 2 |
| CTXN1 | Cortexin 1 |
| ELFN1 | Extracellular Leucine Rich Repeat And Fibronectin Type III Domain Containing 1 |
| NCKAP5 | NCK Associated Protein 5 |
| DXO | Decapping Exoribonuclease |
| COMMD5 | COMM Domain Containing 5 |
| CFAP20 | Cilia And Flagella Associated Protein 20 |
| NKAPL | NFKB Activating Protein Like |
| RTP3 | Receptor Transporter Protein 3 |
| G0S2 | G0/G1 Switch 2 |
| CCDC12 | Coiled-Coil Domain Containing 12 |
| CCDC43 | Coiled-Coil Domain Containing 43 |
| ADGRF1 | Adhesion G Protein-Coupled Receptor F1 |
| C6orf58 | Chromosome 6 Open Reading Frame 58 |
| LURAP1L | Leucine Rich Adaptor Protein 1 Like |
| TNRC18 | Trinucleotide Repeat Containing 18 |
| VNN3 | Vanin 3 |
| SMCP | Sperm Mitochondria Associated Cysteine Rich Protein |
| ZNF831 | Zinc Finger Protein 831 |
| ZNF713 | Zinc Finger Protein 713 |
| ZNF502 | Zinc Finger Protein 502 |
| PTCHD4 | Patched Domain Containing 4 |
| SPATA19 | Spermatogenesis Associated 19 |
| CLEC12B | C-Type Lectin Domain Family 12 Member B |
| SFT2D1 | SFT2 Domain Containing 1 |
| SPPL2C | Signal Peptide Peptidase Like 2C |
| ISY1 | ISY1 Splicing Factor Homolog |
| PATJ | PATJ Crumbs Cell Polarity Complex Component |
| H1-1 | H1.1 Linker Histone, Cluster Member |
| CPTP | Ceramide-1-Phosphate Transfer Protein |
| H4C2 | H4 Clustered Histone 2 |
| GPRIN3 | GPRIN Family Member 3 |
| CWF19L2 | CWF19 Like Cell Cycle Control Factor 2 |
| SAPCD2 | Suppressor APC Domain Containing 2 |
| MESD | Mesoderm Development LRP Chaperone |
| CCDC73 | Coiled-Coil Domain Containing 73 |
| CCDC34 | Coiled-Coil Domain Containing 34 |
| ADGRF3 | Adhesion G Protein-Coupled Receptor F3 |
| C16orf54 | Chromosome 16 Open Reading Frame 54 |
| ERMARD | ER Membrane Associated RNA Degradation |
| TRIM43 | Tripartite Motif Containing 43 |
| ZSCAN31 | Zinc Finger And SCAN Domain Containing 31 |
| RBMX2 | RNA Binding Motif Protein X-Linked 2 |
| SHISA3 | Shisa Family Member 3 |
| QRFP | Pyroglutamylated RFamide Peptide |
| KNOP1 | Lysine Rich Nucleolar Protein 1 |
| TMEM129 | Transmembrane Protein 129 |
| RGPD5 | RANBP2 Like And GRIP Domain Containing 5 |
| NHSL1 | NHS Like 1 |
| H4C8 | H4 Clustered Histone 8 |
| H4C9 | H4 Clustered Histone 9 |
| MPND | MPN Domain Containing |
| SEPTIN8 | Septin 8 |
| CCDC18 | Coiled-Coil Domain Containing 18 |
| ABHD17C | Abhydrolase Domain Containing 17C |
| FAM133B | Family With Sequence Similarity 133 Member B |
| C6orf223 | Chromosome 6 Open Reading Frame 223 |
| CAAP1 | Caspase Activity And Apoptosis Inhibitor 1 |
| CCDC9 | Coiled-Coil Domain Containing 9 |
| LCE3D | Late Cornified Envelope 3D |
| ZNF84 | Zinc Finger Protein 84 |
| H2BC9 | H2B Clustered Histone 9 |
| H4C6 | H4 Clustered Histone 6 |
| MRGPRE | MAS Related GPR Family Member E |
| GAGE1 | G Antigen 1 |
| TMEM14B | Transmembrane Protein 14B |
| ZBED9 | Zinc Finger BED-Type Containing 9 |
| TSPO2 | Translocator Protein 2 |
| RAB40A | RAB40A, Member RAS Oncogene Family |
| INTS11 | Integrator Complex Subunit 11 |
| INTS13 | Integrator Complex Subunit 13 |
| H2AC13 | H2A Clustered Histone 13 |
| H4C12 | H4 Clustered Histone 12 |
| H4C13 | H4 Clustered Histone 13 |
| GLYATL3 | Glycine-N-Acyltransferase Like 3 |
| ARPIN | Actin Related Protein 2/3 Complex Inhibitor |
| TMEM189-UBE2V1 | TMEM189-UBE2V1 Readthrough |
| SMIM13 | Small Integral Membrane Protein 13 |
| ZNF98 | Zinc Finger Protein 98 |
| CT45A1 | Cancer/Testis Antigen Family 45 Member A1 |
| H2BC15 | H2B Clustered Histone 15 |
| H4C4 | H4 Clustered Histone 4 |
| H4C5 | H4 Clustered Histone 5 |
| DCAF4L1 | DDB1 And CUL4 Associated Factor 4 Like 1 |
| SELENOM | Selenoprotein M |
| C2orf74 | Chromosome 2 Open Reading Frame 74 |
| FMR1NB | FMR1 Neighbor |
| LCE3E | Late Cornified Envelope 3E |
| PAGE2 | PAGE Family Member 2 |
| H4C15 | H4 Clustered Histone 15 |
| RTF2 | Replication Termination Factor 2 |
| RAB40AL | RAB40A Like |
| LRRC3C | Leucine Rich Repeat Containing 3C |
| LCE4A | Late Cornified Envelope 4A |
| CTAGE9 | CTAGE Family Member 9 |
| FAM153A | Family With Sequence Similarity 153 Member A |
| RPS10-NUDT3 | RPS10-NUDT3 Readthrough |
| RTL10 | Retrotransposon Gag Like 10 |
| GAS8-AS1 | GAS8 Antisense RNA 1 |
| ARMH4 | Armadillo Like Helical Domain Containing 4 |
| SMIM29 | Small Integral Membrane Protein 29 |
| C5orf66 | Chromosome 5 Open Reading Frame 66 |
| GAGE12F | G Antigen 12F |
| GAGE12J | G Antigen 12J |
| GAGE2D | G Antigen 2D |
| GAGE2E | G Antigen 2E |
| MIR206 | MicroRNA 206 |
| GAGE10 | G Antigen 10 |
| GAGE12C | G Antigen 12C |
| GAGE12D | G Antigen 12D |
| GAGE12G | G Antigen 12G |
| GAGE12H | G Antigen 12H |
| GAGE5 | G Antigen 5 |
| BLOC1S5-TXNDC5 | BLOC1S5-TXNDC5 Readthrough (NMD Candidate) |
| LINC00261 | Long Intergenic Non-Protein Coding RNA 261 |
| LINC00324 | Long Intergenic Non-Protein Coding RNA 324 |
| KIF25-AS1 | KIF25 Antisense RNA 1 |
| RNASEK-C17orf49 | RNASEK-C17orf49 Readthrough |
| GAGE12B | G Antigen 12B |
| GAGE13 | G Antigen 13 |
| GUSBP1 | GUSB Pseudogene 1 |
| CYTOR | Cytoskeleton Regulator RNA |
| GAGE12E | G Antigen 12E |
| GAGE2B | G Antigen 2B |
| GAGE6 | G Antigen 6 |
| GAGE7 | G Antigen 7 |
| CCDC162P | Coiled-Coil Domain Containing 162, Pseudogene |
| FKBP1C | FKBP Prolyl Isomerase 1C |
| SNORA55 | Small Nucleolar RNA, H/ACA Box 55 |
| MIR302B | MicroRNA 302b |
| EEF1E1-BLOC1S5 | EEF1E1-BLOC1S5 Readthrough (NMD Candidate) |
| MIR505 | MicroRNA 505 |
| MIR432 | MicroRNA 432 |
| MSH5-SAPCD1 | MSH5-SAPCD1 Readthrough (NMD Candidate) |
| MIR101-2 | MicroRNA 101-2 |
| GAGE8 | G Antigen 8 |
| RNU1-1 | RNA, U1 Small Nuclear 1 |
| RAB4B-EGLN2 | RAB4B-EGLN2 Readthrough (NMD Candidate) |
| RNF216P1 | Ring Finger Protein 216 Pseudogene 1 |
| MIR517A | MicroRNA 517a |
| MIR452 | MicroRNA 452 |
| HCG4B | HLA Complex Group 4B |
| MIR1296 | MicroRNA 1296 |
| MUC8 | Mucin 8 |
| LINC00608 | Long Intergenic Non-Protein Coding RNA 608 |
| LINC00665 | Long Intergenic Non-Protein Coding RNA 665 |
| DEFB122 | Defensin Beta 122 (Pseudogene) |
| MOXD2P | Monooxygenase DBH Like 2, Pseudogene |
| GAGE4 | G Antigen 4 |
| NRIR | Negative Regulator Of Interferon Response |
| PPIEL | Peptidylprolyl Isomerase E Like Pseudogene |
| MIR378D2 | MicroRNA 378d-2 |
| MIR3940 | MicroRNA 3940 |
| MIF-AS1 | MIF Antisense RNA 1 |
| MIR1231 | MicroRNA 1231 |
| ADD3-AS1 | ADD3 Antisense RNA 1 |
| TTTY1 | Testis-Specific Transcript, Y-Linked 1 |
| LINC01185 | Long Intergenic Non-Protein Coding RNA 1185 |
| GOLGA6L5P | Golgin A6 Family Like 5, Pseudogene |
| IGKJ3 | Immunoglobulin Kappa Joining 3 |
| MMP24OS | MMP24 Opposite Strand |
| MIR548AC | MicroRNA 548ac |
| HORMAD2-AS1 | HORMAD2 And MTMR3 Antisense RNA 1 |
| MIR1178 | MicroRNA 1178 |
| RPSAP47 | Ribosomal Protein SA Pseudogene 47 |
| CEACAM22P | CEA Cell Adhesion Molecule 22, Pseudogene |
| TRBV20OR9-2 | T Cell Receptor Beta Variable 20/OR9-2 (Non-Functional) |
| SKINT1L | Skint1 Like (Pseudogene) |
| MIR4281 | MicroRNA 4281 |
| MICD | MHC Class I Polypeptide-Related Sequence D (Pseudogene) |
| MIR7705 | MicroRNA 7705 |
| HLA-V | Major Histocompatibility Complex, Class I, V (Pseudogene) |
| LINC00297 | Long Intergenic Non-Protein Coding RNA 297 |
| SMAD1-AS1 | SMAD1 Antisense RNA 1 |
| LINC02801 | Long Intergenic Non-Protein Coding RNA 2801 |
| DEFA9P | Defensin Alpha 9, Pseudogene |
| LOC400867 | Uncharacterized LOC400867 |
| STAG3L5P | Stromal Antigen 3-Like 5 Pseudogene |
| IGKJ4 | Immunoglobulin Kappa Joining 4 |
| IGKJ5 | Immunoglobulin Kappa Joining 5 |
| RNU4-5P | RNA, U4 Small Nuclear 5, Pseudogene |
| HCP5B | HLA Complex P5B |
| HLA-T | Major Histocompatibility Complex, Class I, T (Pseudogene) |
| HLA-W | Major Histocompatibility Complex, Class I, W (Pseudogene) |
| RPL13P2 | Ribosomal Protein L13 Pseudogene 2 |
| ABHD15-AS1 | ABHD15 Antisense RNA 1 |
| LOC157273 | Uncharacterized LOC157273 |
| LINC02520 | Long Intergenic Non-Protein Coding RNA 2520 |
| PTMAP6 | Prothymosin Alpha Pseudogene 6 |
| IGKJ2 | Immunoglobulin Kappa Joining 2 |
| CRPP1 | C-Reactive Protein Pseudogene 1 |
| HCG4P11 | HLA Complex Group 4 Pseudogene 11 |
| DNAJB1P1 | DnaJ Heat Shock Protein Family (Hsp40) Member B1 Pseudogene 1 |
| HLA-U | Major Histocompatibility Complex, Class I, U (Pseudogene) |
| LINC02312 | Long Intergenic Non-Protein Coding RNA 2312 |
| HCG4P8 | HLA Complex Group 4 Pseudogene 8 |
| MSNP1 | Moesin Pseudogene 1 |
| HLA-P | Major Histocompatibility Complex, Class I, P (Pseudogene) |
| LOC100131395 | Uncharacterized LOC100131395 |
| RPL21P33 | Ribosomal Protein L21 Pseudogene 33 |
| YTHDF2P1 | YTH Domain Family Member 2 Pseudogene 1 |
| HNRNPCP2 | Heterogeneous Nuclear Ribonucleoprotein C Pseudogene 2 |
| HCG4P5 | HLA Complex Group 4 Pseudogene 5 |
| MICG | MHC Class I Polypeptide-Related Sequence G (Pseudogene) |
| RPL7AP7 | Ribosomal Protein L7a Pseudogene 7 |
| TRIM26BP | Tripartite Motif Containing 26B, Pseudogene |
| RPS26P3 | Ribosomal Protein S26 Pseudogene 3 |
| HCG4P7 | HLA Complex Group 4 Pseudogene 7 |
| HCG9P5 | HLA Complex Group 9 Pseudogene 5 |
| MICF | MHC Class I Polypeptide-Related Sequence F (Pseudogene) |
| VENTXP5 | VENT Homeobox Pseudogene 5 |
| PAEPP1 | Progestagen Associated Endometrial Protein Pseudogene 1 |
| CRIP1P2 | Cysteine Rich Protein 1 Pseudogene 2 |
| CRIP1P4 | Cysteine Rich Protein 1 Pseudogene 4 |
| HCG4P3 | HLA Complex Group 4 Pseudogene 3 |
| HCG4P4 | HLA Complex Group 4 Pseudogene 4 |
| HCG4P9 | HLA Complex Group 4 Pseudogene 9 |
| HCGVIII-2 | HCGVIII-2 Pseudogene |
| HPLH1 | Hemophagocytic Lymphohistiocytosis 1 |
| OTSC2 | Otosclerosis 2 |
| LOC647132 | DExH-Box Helicase 29 Pseudogene |
| CDR3 | Cerebellar Degeneration-Related Autoantigen-3 |
| ADIPQTL3 | Circulating Adiponectin QTL On Chromosome 14 |
| RNA5-8SP | RNA, 5.8S Ribosomal Pseudogene |
| LOC111188163 | MSLN Promoter Region |
| LOC353008 | HLA Complex Group 26 (Non-Protein Coding) Pseudogene |
| LOC353009 | HLA Complex Group 26 (Non-Protein Coding) Pseudogene |
| LOC353010 | HLA Complex Group 26 (Non-Protein Coding) Pseudogene |
| LOC353007 | HLA Complex Group 26 (Non-Protein Coding) Pseudogene |
| LOC106783509 | Nonconserved Acetylation Island Sequence 60 Enhancer |
| LOC107832851 | SIRT1 Promoter Region |
| PDHA1 | Pyruvate Dehydrogenase E1 Subunit Alpha 1 |
| SLC1A1 | Solute Carrier Family 1 Member 1 |
| SCN3A | Sodium Voltage-Gated Channel Alpha Subunit 3 |
| IHH | Indian Hedgehog Signaling Molecule |
| ST3GAL5 | ST3 Beta-Galactoside Alpha-2,3-Sialyltransferase 5 |
| PDK1 | Pyruvate Dehydrogenase Kinase 1 |
| KCNK9 | Potassium Two Pore Domain Channel Subfamily K Member 9 |
| PRKG2 | Protein Kinase CGMP-Dependent 2 |
| PYGM | Glycogen Phosphorylase, Muscle Associated |
| ST3GAL3 | ST3 Beta-Galactoside Alpha-2,3-Sialyltransferase 3 |
| NDUFS7 | NADH:Ubiquinone Oxidoreductase Core Subunit S7 |
| ACY1 | Aminoacylase 1 |
| COASY | Coenzyme A Synthase |
| KCNQ4 | Potassium Voltage-Gated Channel Subfamily Q Member 4 |
| SIRT5 | Sirtuin 5 |
| GRM4 | Glutamate Metabotropic Receptor 4 |
| DCX | Doublecortin |
| CHRM4 | Cholinergic Receptor Muscarinic 4 |
| MELK | Maternal Embryonic Leucine Zipper Kinase |
| ACSL1 | Acyl-CoA Synthetase Long Chain Family Member 1 |
| ACAT2 | Acetyl-CoA Acetyltransferase 2 |
| WIF1 | WNT Inhibitory Factor 1 |
| PDE2A | Phosphodiesterase 2A |
| NANS | N-Acetylneuraminate Synthase |
| KCNQ5 | Potassium Voltage-Gated Channel Subfamily Q Member 5 |
| SIX6 | SIX Homeobox 6 |
| HTR3B | 5-Hydroxytryptamine Receptor 3B |
| SLC12A7 | Solute Carrier Family 12 Member 7 |
| PARVA | Parvin Alpha |
| GRM7 | Glutamate Metabotropic Receptor 7 |
| HMGCS2 | 3-Hydroxy-3-Methylglutaryl-CoA Synthase 2 |
| CYB5A | Cytochrome B5 Type A |
| CYP2J2 | Cytochrome P450 Family 2 Subfamily J Member 2 |
| NDUFB9 | NADH:Ubiquinone Oxidoreductase Subunit B9 |
| COX6A1 | Cytochrome C Oxidase Subunit 6A1 |
| GDI1 | GDP Dissociation Inhibitor 1 |
| GABRA3 | Gamma-Aminobutyric Acid Type A Receptor Subunit Alpha3 |
| GLP2R | Glucagon Like Peptide 2 Receptor |
| MAPKAPK5 | MAPK Activated Protein Kinase 5 |
| ADAMTS1 | ADAM Metallopeptidase With Thrombospondin Type 1 Motif 1 |
| LATS1 | Large Tumor Suppressor Kinase 1 |
| LATS2 | Large Tumor Suppressor Kinase 2 |
| KCNN3 | Potassium Calcium-Activated Channel Subfamily N Member 3 |
| KDM4B | Lysine Demethylase 4B |
| SLC44A1 | Solute Carrier Family 44 Member 1 |
| PRKAG3 | Protein Kinase AMP-Activated Non-Catalytic Subunit Gamma 3 |
| PKMYT1 | Protein Kinase, Membrane Associated Tyrosine/Threonine 1 |
| TARS2 | Threonyl-TRNA Synthetase 2, Mitochondrial |
| GSTO2 | Glutathione S-Transferase Omega 2 |
| HIPK3 | Homeodomain Interacting Protein Kinase 3 |
| HAO1 | Hydroxyacid Oxidase 1 |
| MPST | Mercaptopyruvate Sulfurtransferase |
| MGST3 | Microsomal Glutathione S-Transferase 3 |
| GMPR | Guanosine Monophosphate Reductase |
| MTNR1A | Melatonin Receptor 1A |
| NLGN1 | Neuroligin 1 |
| RPS6KA6 | Ribosomal Protein S6 Kinase A6 |
| FZD10 | Frizzled Class Receptor 10 |
| MARK4 | Microtubule Affinity Regulating Kinase 4 |
| MAP3K9 | Mitogen-Activated Protein Kinase Kinase Kinase 9 |
| APBB1 | Amyloid Beta Precursor Protein Binding Family B Member 1 |
| VARS2 | Valyl-TRNA Synthetase 2, Mitochondrial |
| USP13 | Ubiquitin Specific Peptidase 13 |
| PDK2 | Pyruvate Dehydrogenase Kinase 2 |
| EFNA4 | Ephrin A4 |
| NR2E1 | Nuclear Receptor Subfamily 2 Group E Member 1 |
| NMBR | Neuromedin B Receptor |
| NMNAT2 | Nicotinamide Nucleotide Adenylyltransferase 2 |
| NTSR2 | Neurotensin Receptor 2 |
| TFAP2C | Transcription Factor AP-2 Gamma |
| PPP2R2C | Protein Phosphatase 2 Regulatory Subunit Bgamma |
| IYD | Iodotyrosine Deiodinase |
| RAB4A | RAB4A, Member RAS Oncogene Family |
| ITGA11 | Integrin Subunit Alpha 11 |
| RALGDS | Ral Guanine Nucleotide Dissociation Stimulator |
| TBC1D7 | TBC1 Domain Family Member 7 |
| ITPK1 | Inositol-Tetrakisphosphate 1-Kinase |
| PBX3 | PBX Homeobox 3 |
| SHANK2 | SH3 And Multiple Ankyrin Repeat Domains 2 |
| PADI3 | Peptidyl Arginine Deiminase 3 |
| SLC22A7 | Solute Carrier Family 22 Member 7 |
| TAF4B | TATA-Box Binding Protein Associated Factor 4b |
| PTPN7 | Protein Tyrosine Phosphatase Non-Receptor Type 7 |
| PTPRM | Protein Tyrosine Phosphatase Receptor Type M |
| TMEM38B | Transmembrane Protein 38B |
| CLK3 | CDC Like Kinase 3 |
| GRIK3 | Glutamate Ionotropic Receptor Kainate Type Subunit 3 |
| D2HGDH | D-2-Hydroxyglutarate Dehydrogenase |
| RXRG | Retinoid X Receptor Gamma |
| SEPHS1 | Selenophosphate Synthetase 1 |
| GOPC | Golgi Associated PDZ And Coiled-Coil Motif Containing |
| ELOVL1 | ELOVL Fatty Acid Elongase 1 |
| OPHN1 | Oligophrenin 1 |
| DAPK2 | Death Associated Protein Kinase 2 |
| GABRR1 | Gamma-Aminobutyric Acid Type A Receptor Subunit Rho1 |
| CDH4 | Cadherin 4 |
| FPGS | Folylpolyglutamate Synthase |
| ACSF3 | Acyl-CoA Synthetase Family Member 3 |
| LOXL4 | Lysyl Oxidase Like 4 |
| FHL3 | Four And A Half LIM Domains 3 |
| CACNG1 | Calcium Voltage-Gated Channel Auxiliary Subunit Gamma 1 |
| ARFGEF2 | ADP Ribosylation Factor Guanine Nucleotide Exchange Factor 2 |
| ARHGEF11 | Rho Guanine Nucleotide Exchange Factor 11 |
| ARHGEF3 | Rho Guanine Nucleotide Exchange Factor 3 |
| CALML3 | Calmodulin Like 3 |
| UCK2 | Uridine-Cytidine Kinase 2 |
| TSFM | Ts Translation Elongation Factor, Mitochondrial |
| TTBK2 | Tau Tubulin Kinase 2 |
| SMOC2 | SPARC Related Modular Calcium Binding 2 |
| PIGQ | Phosphatidylinositol Glycan Anchor Biosynthesis Class Q |
| SLC38A2 | Solute Carrier Family 38 Member 2 |
| PTPRG | Protein Tyrosine Phosphatase Receptor Type G |
| CDO1 | Cysteine Dioxygenase Type 1 |
| KCNC4 | Potassium Voltage-Gated Channel Subfamily C Member 4 |
| LIPF | Lipase F, Gastric Type |
| NDUFA8 | NADH:Ubiquinone Oxidoreductase Subunit A8 |
| EFNA2 | Ephrin A2 |
| KCNH6 | Potassium Voltage-Gated Channel Subfamily H Member 6 |
| PRKX | Protein Kinase X-Linked |
| PPIH | Peptidylprolyl Isomerase H |
| KANK1 | KN Motif And Ankyrin Repeat Domains 1 |
| PCYT1B | Phosphate Cytidylyltransferase 1, Choline, Beta |
| STAG3 | Stromal Antigen 3 |
| SSH1 | Slingshot Protein Phosphatase 1 |
| CLN5 | CLN5 Intracellular Trafficking Protein |
| GUCY1A2 | Guanylate Cyclase 1 Soluble Subunit Alpha 2 |
| CKS2 | CDC28 Protein Kinase Regulatory Subunit 2 |
| MLC1 | Modulator Of VRAC Current 1 |
| CYP39A1 | Cytochrome P450 Family 39 Subfamily A Member 1 |
| CTDP1 | CTD Phosphatase Subunit 1 |
| OSBPL2 | Oxysterol Binding Protein Like 2 |
| GPC5 | Glypican 5 |
| RND3 | Rho Family GTPase 3 |
| SAR1A | Secretion Associated Ras Related GTPase 1A |
| CDH6 | Cadherin 6 |
| CCDC88A | Coiled-Coil Domain Containing 88A |
| ADAM22 | ADAM Metallopeptidase Domain 22 |
| BACH1 | BTB Domain And CNC Homolog 1 |
| CAPNS1 | Calpain Small Subunit 1 |
| WWC1 | WW And C2 Domain Containing 1 |
| PICK1 | Protein Interacting With PRKCA 1 |
| EFHC1 | EF-Hand Domain Containing 1 |
| NDUFAB1 | NADH:Ubiquinone Oxidoreductase Subunit AB1 |
| NTM | Neurotrimin |
| POLA2 | DNA Polymerase Alpha 2, Accessory Subunit |
| PPIL1 | Peptidylprolyl Isomerase Like 1 |
| PPA2 | Inorganic Pyrophosphatase 2 |
| SPAST | Spastin |
| HTATIP2 | HIV-1 Tat Interactive Protein 2 |
| RAD9A | RAD9 Checkpoint Clamp Component A |
| RAB33B | RAB33B, Member RAS Oncogene Family |
| SLC26A8 | Solute Carrier Family 26 Member 8 |
| SLC26A9 | Solute Carrier Family 26 Member 9 |
| P2RX6 | Purinergic Receptor P2X 6 |
| IQGAP3 | IQ Motif Containing GTPase Activating Protein 3 |
| INPP5J | Inositol Polyphosphate-5-Phosphatase J |
| SYNJ2 | Synaptojanin 2 |
| PTPRR | Protein Tyrosine Phosphatase Receptor Type R |
| KCNB2 | Potassium Voltage-Gated Channel Subfamily B Member 2 |
| PROZ | Protein Z, Vitamin K Dependent Plasma Glycoprotein |
| SSR1 | Signal Sequence Receptor Subunit 1 |
| PRPF4B | Pre-MRNA Processing Factor 4B |
| PRSS21 | Serine Protease 21 |
| CLEC3B | C-Type Lectin Domain Family 3 Member B |
| CNOT8 | CCR4-NOT Transcription Complex Subunit 8 |
| CYBRD1 | Cytochrome B Reductase 1 |
| RIOK1 | RIO Kinase 1 |
| DBF4 | DBF4 Zinc Finger |
| CTDSP1 | CTD Small Phosphatase 1 |
| HIPK1 | Homeodomain Interacting Protein Kinase 1 |
| EIF4H | Eukaryotic Translation Initiation Factor 4H |
| ECI1 | Enoyl-CoA Delta Isomerase 1 |
| NALCN | Sodium Leak Channel, Non-Selective |
| NAAA | N-Acylethanolamine Acid Amidase |
| COX7A2L | Cytochrome C Oxidase Subunit 7A2 Like |
| COPZ1 | COPI Coat Complex Subunit Zeta 1 |
| HOXD10 | Homeobox D10 |
| HOXD9 | Homeobox D9 |
| DDO | D-Aspartate Oxidase |
| HPCAL1 | Hippocalcin Like 1 |
| SCN7A | Sodium Voltage-Gated Channel Alpha Subunit 7 |
| OSBP | Oxysterol Binding Protein |
| SERINC1 | Serine Incorporator 1 |
| CHRNB3 | Cholinergic Receptor Nicotinic Beta 3 Subunit |
| GMFB | Glia Maturation Factor Beta |
| ELOVL6 | ELOVL Fatty Acid Elongase 6 |
| RTN2 | Reticulon 2 |
| RTN4IP1 | Reticulon 4 Interacting Protein 1 |
| MOCS1 | Molybdenum Cofactor Synthesis 1 |
| GGA1 | Golgi Associated, Gamma Adaptin Ear Containing, ARF Binding Protein 1 |
| GABRR2 | Gamma-Aminobutyric Acid Type A Receptor Subunit Rho2 |
| MED13 | Mediator Complex Subunit 13 |
| CCDC6 | Coiled-Coil Domain Containing 6 |
| AGXT2 | Alanine--Glyoxylate Aminotransferase 2 |
| AKAP10 | A-Kinase Anchoring Protein 10 |
| B4GALT3 | Beta-1,4-Galactosyltransferase 3 |
| B4GALT5 | Beta-1,4-Galactosyltransferase 5 |
| BTG1 | BTG Anti-Proliferation Factor 1 |
| LPAR5 | Lysophosphatidic Acid Receptor 5 |
| BRPF1 | Bromodomain And PHD Finger Containing 1 |
| BNIP2 | BCL2 Interacting Protein 2 |
| LYPLA1 | Lysophospholipase 1 |
| MAFF | MAF BZIP Transcription Factor F |
| CAPN6 | Calpain 6 |
| ARAP1 | ArfGAP With RhoGAP Domain, Ankyrin Repeat And PH Domain 1 |
| ARL6IP1 | ADP Ribosylation Factor Like GTPase 6 Interacting Protein 1 |
| KRT3 | Keratin 3 |
| KIF5C | Kinesin Family Member 5C |
| TRDN | Triadin |
| ZNF41 | Zinc Finger Protein 41 |
| UGT1A10 | UDP Glucuronosyltransferase Family 1 Member A10 |
| USP53 | Ubiquitin Specific Peptidase 53 |
| SMARCD1 | SWI/SNF Related, Matrix Associated, Actin Dependent Regulator Of Chromatin, Subfamily D, Member 1 |
| SLC8A2 | Solute Carrier Family 8 Member A2 |
| HTR1E | 5-Hydroxytryptamine Receptor 1E |
| COX11 | Cytochrome C Oxidase Copper Chaperone COX11 |
| EMP3 | Epithelial Membrane Protein 3 |
| NRN1 | Neuritin 1 |
| NUAK2 | NUAK Family Kinase 2 |
| SIX2 | SIX Homeobox 2 |
| SERPINA10 | Serpin Family A Member 10 |
| SV2B | Synaptic Vesicle Glycoprotein 2B |
| TFDP2 | Transcription Factor Dp-2 |
| TFEC | Transcription Factor EC |
| KCNK6 | Potassium Two Pore Domain Channel Subfamily K Member 6 |
| RBMS1 | RNA Binding Motif Single Stranded Interacting Protein 1 |
| PDE7B | Phosphodiesterase 7B |
| SLC6A7 | Solute Carrier Family 6 Member 7 |
| SLC25A17 | Solute Carrier Family 25 Member 17 |
| SORBS2 | Sorbin And SH3 Domain Containing 2 |
| PPP1R9A | Protein Phosphatase 1 Regulatory Subunit 9A |
| KATNA1 | Katanin Catalytic Subunit A1 |
| RAB25 | RAB25, Member RAS Oncogene Family |
| RABEPK | Rab9 Effector Protein With Kelch Motifs |
| SLC2A12 | Solute Carrier Family 2 Member 12 |
| SLC2A13 | Solute Carrier Family 2 Member 13 |
| PCOLCE | Procollagen C-Endopeptidase Enhancer |
| P4HA3 | Prolyl 4-Hydroxylase Subunit Alpha 3 |
| SH3GLB2 | SH3 Domain Containing GRB2 Like, Endophilin B2 |
| PCDH10 | Protocadherin 10 |
| PZP | PZP Alpha-2-Macroglobulin Like |
| RAB11FIP5 | RAB11 Family Interacting Protein 5 |
| PUM2 | Pumilio RNA Binding Family Member 2 |
| INSIG1 | Insulin Induced Gene 1 |
| KIAA0319 | KIAA0319 |
| TJP3 | Tight Junction Protein 3 |
| PPM1F | Protein Phosphatase, Mg2+/Mn2+ Dependent 1F |
| STARD3 | StAR Related Lipid Transfer Domain Containing 3 |
| STEAP1 | STEAP Family Member 1 |
| CNOT6 | CCR4-NOT Transcription Complex Subunit 6 |
| CLASP1 | Cytoplasmic Linker Associated Protein 1 |
| CYB5R4 | Cytochrome B5 Reductase 4 |
| RIN1 | Ras And Rab Interactor 1 |
| RIOK2 | RIO Kinase 2 |
| RIT2 | Ras Like Without CAAX 2 |
| MOS | MOS Proto-Oncogene, Serine/Threonine Kinase |
| HEYL | Hes Related Family BHLH Transcription Factor With YRPW Motif Like |
| HFM1 | Helicase For Meiosis 1 |
| HIF3A | Hypoxia Inducible Factor 3 Subunit Alpha |
| CTNNAL1 | Catenin Alpha Like 1 |
| DACH1 | Dachshund Family Transcription Factor 1 |
| NDRG4 | NDRG Family Member 4 |
| DYNLRB2 | Dynein Light Chain Roadblock-Type 2 |
| NEK6 | NIMA Related Kinase 6 |
| NELL1 | Neural EGFL Like 1 |
| EBF3 | EBF Transcription Factor 3 |
| CREB3L2 | CAMP Responsive Element Binding Protein 3 Like 2 |
| CPA5 | Carboxypeptidase A5 |
| COQ3 | Coenzyme Q3, Methyltransferase |
| HS3ST2 | Heparan Sulfate-Glucosamine 3-Sulfotransferase 2 |
| S100A3 | S100 Calcium Binding Protein A3 |
| SAV1 | Salvador Family WW Domain Containing Protein 1 |
| CHAD | Chondroadherin |
| CETN3 | Centrin 3 |
| GPR161 | G Protein-Coupled Receptor 161 |
| GLYAT | Glycine-N-Acyltransferase |
| DPP3 | Dipeptidyl Peptidase 3 |
| DOCK4 | Dedicator Of Cytokinesis 4 |
| DUSP23 | Dual Specificity Phosphatase 23 |
| NDUFB7 | NADH:Ubiquinone Oxidoreductase Subunit B7 |
| NEUROG1 | Neurogenin 1 |
| NPRL2 | NPR2 Like, GATOR1 Complex Subunit |
| OAZ1 | Ornithine Decarboxylase Antizyme 1 |
| NUDT3 | Nudix Hydrolase 3 |
| CDK17 | Cyclin Dependent Kinase 17 |
| CDC40 | Cell Division Cycle 40 |
| CDC42BPA | CDC42 Binding Protein Kinase Alpha |
| MCM9 | Minichromosome Maintenance 9 Homologous Recombination Repair Factor |
| FRMD7 | FERM Domain Containing 7 |
| ALDH1L2 | Aldehyde Dehydrogenase 1 Family Member L2 |
| ADAT1 | Adenosine Deaminase TRNA Specific 1 |
| BICD1 | BICD Cargo Adaptor 1 |
| BCL2L10 | BCL2 Like 10 |
| ATP13A1 | ATPase 13A1 |
| B3GALT4 | Beta-1,3-Galactosyltransferase 4 |
| B4GALT6 | Beta-1,4-Galactosyltransferase 6 |
| LRP1B | LDL Receptor Related Protein 1B |
| FLRT1 | Fibronectin Leucine Rich Transmembrane Protein 1 |
| CA7 | Carbonic Anhydrase 7 |
| MAML3 | Mastermind Like Transcriptional Coactivator 3 |
| CACNG6 | Calcium Voltage-Gated Channel Auxiliary Subunit Gamma 6 |
| ALX4 | ALX Homeobox 4 |
| APBB2 | Amyloid Beta Precursor Protein Binding Family B Member 2 |
| ARFIP2 | ADP Ribosylation Factor Interacting Protein 2 |
| APOL2 | Apolipoprotein L2 |
| ASIC2 | Acid Sensing Ion Channel Subunit 2 |
| ADAMTS8 | ADAM Metallopeptidase With Thrombospondin Type 1 Motif 8 |
| FEZ1 | Fasciculation And Elongation Protein Zeta 1 |
| TXNRD3 | Thioredoxin Reductase 3 |
| ZFAND5 | Zinc Finger AN1-Type Containing 5 |
| LIN7A | Lin-7 Homolog A, Crumbs Cell Polarity Complex Component |
| TSEN2 | TRNA Splicing Endonuclease Subunit 2 |
| TSHZ2 | Teashirt Zinc Finger Homeobox 2 |
| TSPAN8 | Tetraspanin 8 |
| PDHA2 | Pyruvate Dehydrogenase E1 Subunit Alpha 2 |
| PHF1 | PHD Finger Protein 1 |
| SLC25A36 | Solute Carrier Family 25 Member 36 |
| SGSM3 | Small G Protein Signaling Modulator 3 |
| PSMC3IP | PSMC3 Interacting Protein |
| IMPG1 | Interphotoreceptor Matrix Proteoglycan 1 |
| LLGL2 | LLGL Scribble Cell Polarity Complex Component 2 |
| CHRNA10 | Cholinergic Receptor Nicotinic Alpha 10 Subunit |
| ENAM | Enamelin |
| SUPT6H | SPT6 Homolog, Histone Chaperone And Transcription Elongation Factor |
| KCNG3 | Potassium Voltage-Gated Channel Modifier Subfamily G Member 3 |
| KDM1B | Lysine Demethylase 1B |
| SLC5A10 | Solute Carrier Family 5 Member 10 |
| SS18L1 | SS18L1 Subunit Of BAF Chromatin Remodeling Complex |
| PPP4R1 | Protein Phosphatase 4 Regulatory Subunit 1 |
| PM20D1 | Peptidase M20 Domain Containing 1 |
| PLCH1 | Phospholipase C Eta 1 |
| SESN2 | Sestrin 2 |
| SOBP | Sine Oculis Binding Protein Homolog |
| PPME1 | Protein Phosphatase Methylesterase 1 |
| SLC2A8 | Solute Carrier Family 2 Member 8 |
| SLC30A3 | Solute Carrier Family 30 Member 3 |
| PABPC3 | Poly(A) Binding Protein Cytoplasmic 3 |
| PANX2 | Pannexin 2 |
| SHOX2 | Short Stature Homeobox 2 |
| PAIP2 | Poly(A) Binding Protein Interacting Protein 2 |
| PARD6G | Par-6 Family Cell Polarity Regulator Gamma |
| TACC2 | Transforming Acidic Coiled-Coil Containing Protein 2 |
| INTS3 | Integrator Complex Subunit 3 |
| IPPK | Inositol-Pentakisphosphate 2-Kinase |
| QRSL1 | Glutaminyl-TRNA Amidotransferase Subunit QRSL1 |
| RAB11FIP4 | RAB11 Family Interacting Protein 4 |
| SYT5 | Synaptotagmin 5 |
| KLF12 | Kruppel Like Factor 12 |
| STAP2 | Signal Transducing Adaptor Family Member 2 |
| SSX2IP | SSX Family Member 2 Interacting Protein |
| LY6D | Lymphocyte Antigen 6 Family Member D |
| CLSTN1 | Calsyntenin 1 |
| CIRBP | Cold Inducible RNA Binding Protein |
| CISD1 | CDGSH Iron Sulfur Domain 1 |
| GTF3C2 | General Transcription Factor IIIC Subunit 2 |
| RFFL | Ring Finger And FYVE Like Domain Containing E3 Ubiquitin Protein Ligase |
| DAZAP1 | DAZ Associated Protein 1 |
| HEY2 | Hes Related Family BHLH Transcription Factor With YRPW Motif 2 |
| RHPN2 | Rhophilin Rho GTPase Binding Protein 2 |
| MYCBP | MYC Binding Protein |
| NDRG3 | NDRG Family Member 3 |
| COL19A1 | Collagen Type XIX Alpha 1 Chain |
| DCP2 | Decapping MRNA 2 |
| MTCH1 | Mitochondrial Carrier 1 |
| MPC2 | Mitochondrial Pyruvate Carrier 2 |
| DKK4 | Dickkopf WNT Signaling Pathway Inhibitor 4 |
| SCUBE3 | Signal Peptide, CUB Domain And EGF Like Domain Containing 3 |
| SFXN5 | Sideroflexin 5 |
| OSBP2 | Oxysterol Binding Protein 2 |
| SCAND1 | SCAN Domain Containing 1 |
| CEP41 | Centrosomal Protein 41 |
| CBX8 | Chromobox 8 |
| MYADM | Myeloid Associated Differentiation Marker |
| EMILIN1 | Elastin Microfibril Interfacer 1 |
| RPL36AL | Ribosomal Protein L36a Like |
| NXT1 | Nuclear Transport Factor 2 Like Export Factor 1 |
| RTN4RL1 | Reticulon 4 Receptor Like 1 |
| RRAGB | Ras Related GTP Binding B |
| DLGAP4 | DLG Associated Protein 4 |
| GIPC2 | GIPC PDZ Domain Containing Family Member 2 |
| CBLC | Cbl Proto-Oncogene C |
| CBR4 | Carbonyl Reductase 4 |
| MCHR2 | Melanin Concentrating Hormone Receptor 2 |
| ADGRL4 | Adhesion G Protein-Coupled Receptor L4 |
| AIFM2 | Apoptosis Inducing Factor Mitochondria Associated 2 |
| AFF3 | AF4/FMR2 Family Member 3 |
| FARP1 | FERM, ARH/RhoGEF And Pleckstrin Domain Protein 1 |
| BANP | BTG3 Associated Nuclear Protein |
| BDH2 | 3-Hydroxybutyrate Dehydrogenase 2 |
| BABAM1 | BRISC And BRCA1 A Complex Member 1 |
| LYRM4 | LYR Motif Containing 4 |
| CALCB | Calcitonin Related Polypeptide Beta |
| LZTS1 | Leucine Zipper Tumor Suppressor 1 |
| AMDHD1 | Amidohydrolase Domain Containing 1 |
| AMOTL1 | Angiomotin Like 1 |
| ARHGEF19 | Rho Guanine Nucleotide Exchange Factor 19 |
| ART5 | ADP-Ribosyltransferase 5 |
| EPHA10 | EPH Receptor A10 |
| ATE1 | Arginyltransferase 1 |
| GABRE | Gamma-Aminobutyric Acid Type A Receptor Subunit Epsilon |
| KRT86 | Keratin 86 |
| TRIM44 | Tripartite Motif Containing 44 |
| TRERF1 | Transcriptional Regulating Factor 1 |
| VKORC1L1 | Vitamin K Epoxide Reductase Complex Subunit 1 Like 1 |
| YY1AP1 | YY1 Associated Protein 1 |
| UBXN6 | UBX Domain Protein 6 |
| USP37 | Ubiquitin Specific Peptidase 37 |
| UGT1A3 | UDP Glucuronosyltransferase Family 1 Member A3 |
| UGT1A7 | UDP Glucuronosyltransferase Family 1 Member A7 |
| WDR59 | WD Repeat Domain 59 |
| WEE2 | WEE2 Oocyte Meiosis Inhibiting Kinase |
| TSPAN5 | Tetraspanin 5 |
| PFDN4 | Prefoldin Subunit 4 |
| PFDN5 | Prefoldin Subunit 5 |
| SLC9A7 | Solute Carrier Family 9 Member A7 |
| PDPR | Pyruvate Dehydrogenase Phosphatase Regulatory Subunit |
| PDXDC1 | Pyridoxal Dependent Decarboxylase Domain Containing 1 |
| SLC47A2 | Solute Carrier Family 47 Member 2 |
| SLC52A1 | Solute Carrier Family 52 Member 1 |
| ZNF79 | Zinc Finger Protein 79 |
| ZPBP | Zona Pellucida Binding Protein |
| ZNF212 | Zinc Finger Protein 212 |
| SYNGR2 | Synaptogyrin 2 |
| RBP2 | Retinol Binding Protein 2 |
| HAPLN3 | Hyaluronan And Proteoglycan Link Protein 3 |
| ENPP5 | Ectonucleotide Pyrophosphatase/Phosphodiesterase Family Member 5 |
| NTPCR | Nucleoside-Triphosphatase, Cancer-Related |
| SLC16A6 | Solute Carrier Family 16 Member 6 |
| KHDRBS2 | KH RNA Binding Domain Containing, Signal Transduction Associated 2 |
| KDM4D | Lysine Demethylase 4D |
| SPOCK2 | SPARC (Osteonectin), Cwcv And Kazal Like Domains Proteoglycan 2 |
| PRCC | Proline Rich Mitotic Checkpoint Control Factor |
| RALGAPA2 | Ral GTPase Activating Protein Catalytic Subunit Alpha 2 |
| PCBD2 | Pterin-4 Alpha-Carbinolamine Dehydratase 2 |
| SUMF2 | Sulfatase Modifying Factor 2 |
| SYNM | Synemin |
| TMEM9 | Transmembrane Protein 9 |
| KLHL15 | Kelch Like Family Member 15 |
| PRTG | Protogenin |
| CKMT1A | Creatine Kinase, Mitochondrial 1A |
| HMGCLL1 | 3-Hydroxymethyl-3-Methylglutaryl-CoA Lyase Like 1 |
| CTRB1 | Chymotrypsinogen B1 |
| EHD3 | EH Domain Containing 3 |
| ECHDC1 | Ethylmalonyl-CoA Decarboxylase 1 |
| NETO2 | Neuropilin And Tolloid Like 2 |
| NEU4 | Neuraminidase 4 |
| COL21A1 | Collagen Type XXI Alpha 1 Chain |
| HOXD11 | Homeobox D11 |
| MRS2 | Magnesium Transporter MRS2 |
| MRI1 | Methylthioribose-1-Phosphate Isomerase 1 |
| MRPL19 | Mitochondrial Ribosomal Protein L19 |
| RMND1 | Required For Meiotic Nuclear Division 1 Homolog |
| SCPEP1 | Serine Carboxypeptidase 1 |
| SEC16B | SEC16 Homolog B, Endoplasmic Reticulum Export Factor |
| SENP5 | SUMO Specific Peptidase 5 |
| GPR61 | G Protein-Coupled Receptor 61 |
| MIOS | Meiosis Regulator For Oocyte Development |
| C12orf57 | Chromosome 12 Open Reading Frame 57 |
| NIT1 | Nitrilase 1 |
| RPTN | Repetin |
| NUF2 | NUF2 Component Of NDC80 Kinetochore Complex |
| CDH18 | Cadherin 18 |
| FOXF2 | Forkhead Box F2 |
| FRMD5 | FERM Domain Containing 5 |
| ADO | 2-Aminoethanethiol Dioxygenase |
| ERI1 | Exoribonuclease 1 |
| LONP2 | Lon Peptidase 2, Peroxisomal |
| LRRTM3 | Leucine Rich Repeat Transmembrane Neuronal 3 |
| ACOT9 | Acyl-CoA Thioesterase 9 |
| ALKBH8 | AlkB Homolog 8, TRNA Methyltransferase |
| TOMM34 | Translocase Of Outer Mitochondrial Membrane 34 |
| TUBE1 | Tubulin Epsilon 1 |
| TUBG2 | Tubulin Gamma 2 |
| ZC3H7B | Zinc Finger CCCH-Type Containing 7B |
| TRIM55 | Tripartite Motif Containing 55 |
| TRMT11 | TRNA Methyltransferase 11 Homolog |
| PDLIM3 | PDZ And LIM Domain 3 |
| PHF10 | PHD Finger Protein 10 |
| PHLDB1 | Pleckstrin Homology Like Domain Family B Member 1 |
| SLC35F2 | Solute Carrier Family 35 Member F2 |
| SYPL1 | Synaptophysin Like 1 |
| STAC2 | SH3 And Cysteine Rich Domain 2 |
| CST4 | Cystatin S |
| KCNK16 | Potassium Two Pore Domain Channel Subfamily K Member 16 |
| KCNK17 | Potassium Two Pore Domain Channel Subfamily K Member 17 |
| RBM6 | RNA Binding Motif Protein 6 |
| SRSF10 | Serine And Arginine Rich Splicing Factor 10 |
| SPINK7 | Serine Peptidase Inhibitor Kazal Type 7 |
| SNAPC5 | Small Nuclear RNA Activating Complex Polypeptide 5 |
| PKDCC | Protein Kinase Domain Containing, Cytoplasmic |
| PLLP | Plasmolipin |
| PLEKHB1 | Pleckstrin Homology Domain Containing B1 |
| SNPH | Syntaphilin |
| PLB1 | Phospholipase B1 |
| PIGY | Phosphatidylinositol Glycan Anchor Biosynthesis Class Y |
| HSD17B13 | Hydroxysteroid 17-Beta Dehydrogenase 13 |
| SNX13 | Sorting Nexin 13 |
| PPFIA3 | PTPRF Interacting Protein Alpha 3 |
| SORCS2 | Sortilin Related VPS10 Domain Containing Receptor 2 |
| PLCXD2 | Phosphatidylinositol Specific Phospholipase C X Domain Containing 2 |
| TCTN1 | Tectonic Family Member 1 |
| RASGEF1B | RasGEF Domain Family Member 1B |
| TENM3 | Teneurin Transmembrane Protein 3 |
| RAB22A | RAB22A, Member RAS Oncogene Family |
| TCEAL1 | Transcription Elongation Factor A Like 1 |
| TCP11 | T-Complex 11 |
| SLC2A4RG | SLC2A4 Regulator |
| OVOL1 | Ovo Like Transcriptional Repressor 1 |
| OVOL2 | Ovo Like Zinc Finger 2 |
| SLC13A1 | Solute Carrier Family 13 Member 1 |
| PALM | Paralemmin |
| SKA1 | Spindle And Kinetochore Associated Complex Subunit 1 |
| PAQR8 | Progestin And AdipoQ Receptor Family Member 8 |
| SLC23A3 | Solute Carrier Family 23 Member 3 |
| INSM1 | INSM Transcriptional Repressor 1 |
| TAF9B | TATA-Box Binding Protein Associated Factor 9b |
| SZT2 | SZT2 Subunit Of KICSTOR Complex |
| TAAR2 | Trace Amine Associated Receptor 2 (Gene/Pseudogene) |
| RBM12 | RNA Binding Motif Protein 12 |
| TMEM91 | Transmembrane Protein 91 |
| TMX4 | Thioredoxin Related Transmembrane Protein 4 |
| KLF9 | Kruppel Like Factor 9 |
| LRRC1 | Leucine Rich Repeat Containing 1 |
| GTF2A1L | General Transcription Factor IIA Subunit 1 Like |
| GXYLT2 | Glucoside Xylosyltransferase 2 |
| CKAP2 | Cytoskeleton Associated Protein 2 |
| CMTM8 | CKLF Like MARVEL Transmembrane Domain Containing 8 |
| MIXL1 | Mix Paired-Like Homeobox |
| HEXIM2 | HEXIM P-TEFb Complex Subunit 2 |
| MND1 | Meiotic Nuclear Divisions 1 |
| DACH2 | Dachshund Family Transcription Factor 2 |
| MNT | MAX Network Transcriptional Repressor |
| EIF4ENIF1 | Eukaryotic Translation Initiation Factor 4E Nuclear Import Factor 1 |
| NCOA7 | Nuclear Receptor Coactivator 7 |
| RNPEPL1 | Arginyl Aminopeptidase Like 1 |
| NAT8 | N-Acetyltransferase 8 (Putative) |
| RNF181 | Ring Finger Protein 181 |
| CSNK1A1L | Casein Kinase 1 Alpha 1 Like |
| HOXC9 | Homeobox C9 |
| MRGPRD | MAS Related GPR Family Member D |
| HOXD4 | Homeobox D4 |
| DHX34 | DExH-Box Helicase 34 |
| DDX19B | DEAD-Box Helicase 19B |
| HOPX | HOP Homeobox |
| SETD6 | SET Domain Containing 6, Protein Lysine Methyltransferase |
| ORMDL2 | ORMDL Sphingolipid Biosynthesis Regulator 2 |
| CHST9 | Carbohydrate Sulfotransferase 9 |
| GPR142 | G Protein-Coupled Receptor 142 |
| CEP72 | Centrosomal Protein 72 |
| CEP78 | Centrosomal Protein 78 |
| DPPA2 | Developmental Pluripotency Associated 2 |
| MYLK4 | Myosin Light Chain Kinase Family Member 4 |
| DUS2 | Dihydrouridine Synthase 2 |
| DUSP18 | Dual Specificity Phosphatase 18 |
| COMTD1 | Catechol-O-Methyltransferase Domain Containing 1 |
| DDX43 | DEAD-Box Helicase 43 |
| OAZ2 | Ornithine Decarboxylase Antizyme 2 |
| GBGT1 | Globoside Alpha-1,3-N-Acetylgalactosaminyltransferase 1 (FORS Blood Group) |
| CATSPER3 | Cation Channel Sperm Associated 3 |
| MED12L | Mediator Complex Subunit 12L |
| MATN4 | Matrilin 4 |
| ABHD2 | Abhydrolase Domain Containing 2 |
| FAIM2 | Fas Apoptotic Inhibitory Molecule 2 |
| B3GAT2 | Beta-1,3-Glucuronyltransferase 2 |
| FAM120B | Family With Sequence Similarity 120B |
| BTBD3 | BTB Domain Containing 3 |
| BTBD9 | BTB Domain Containing 9 |
| BTF3L4 | Basic Transcription Factor 3 Like 4 |
| LPCAT3 | Lysophosphatidylcholine Acyltransferase 3 |
| BRPF3 | Bromodomain And PHD Finger Containing 3 |
| LRFN1 | Leucine Rich Repeat And Fibronectin Type III Domain Containing 1 |
| LRIG3 | Leucine Rich Repeats And Immunoglobulin Like Domains 3 |
| LRRC15 | Leucine Rich Repeat Containing 15 |
| ATG2A | Autophagy Related 2A |
| FEM1B | Fem-1 Homolog B |
| FNDC3A | Fibronectin Type III Domain Containing 3A |
| AP3S2 | Adaptor Related Protein Complex 3 Subunit Sigma 2 |
| ARPP19 | CAMP Regulated Phosphoprotein 19 |
| ERP27 | Endoplasmic Reticulum Protein 27 |
| ASTN2 | Astrotactin 2 |
| ADAP2 | ArfGAP With Dual PH Domains 2 |
| FEZ2 | Fasciculation And Elongation Protein Zeta 2 |
| KRT24 | Keratin 24 |
| TOR2A | Torsin Family 2 Member A |
| TLX2 | T Cell Leukemia Homeobox 2 |
| TUBAL3 | Tubulin Alpha Like 3 |
| WDR45B | WD Repeat Domain 45B |
| UNC119B | Unc-119 Lipid Binding Chaperone B |
| TUBD1 | Tubulin Delta 1 |
| LETMD1 | LETM1 Domain Containing 1 |
| ZCCHC7 | Zinc Finger CCHC-Type Containing 7 |
| ZDHHC3 | Zinc Finger DHHC-Type Palmitoyltransferase 3 |
| ZFP64 | ZFP64 Zinc Finger Protein |
| ZFAND6 | Zinc Finger AN1-Type Containing 6 |
| ZNF12 | Zinc Finger Protein 12 |
| ZNF182 | Zinc Finger Protein 182 |
| YEATS2 | YEATS Domain Containing 2 |
| VPS72 | Vacuolar Protein Sorting 72 Homolog |
| VPS52 | VPS52 Subunit Of GARP Complex |
| TOMM22 | Translocase Of Outer Mitochondrial Membrane 22 |
| TMEM14C | Transmembrane Protein 14C |
| TP53AIP1 | Tumor Protein P53 Regulated Apoptosis Inducing Protein 1 |
| TSPAN3 | Tetraspanin 3 |
| PIEZO2 | Piezo Type Mechanosensitive Ion Channel Component 2 |
| SMYD4 | SET And MYND Domain Containing 4 |
| SLC37A3 | Solute Carrier Family 37 Member 3 |
| SLC38A8 | Solute Carrier Family 38 Member 8 |
| PFDN6 | Prefoldin Subunit 6 |
| SLC9A5 | Solute Carrier Family 9 Member A5 |
| SLC25A43 | Solute Carrier Family 25 Member 43 |
| ZNF264 | Zinc Finger Protein 264 |
| ZNF268 | Zinc Finger Protein 268 |
| SRP19 | Signal Recognition Particle 19 |
| FKBPL | FKBP Prolyl Isomerase Like |
| GDAP2 | Ganglioside Induced Differentiation Associated Protein 2 |
| ZNF519 | Zinc Finger Protein 519 |
| ZNF668 | Zinc Finger Protein 668 |
| GRTP1 | Growth Hormone Regulated TBC Protein 1 |
| NANOS2 | Nanos C2HC-Type Zinc Finger 2 |
| ENOSF1 | Enolase Superfamily Member 1 |
| ENOX1 | Ecto-NOX Disulfide-Thiol Exchanger 1 |
| SLC10A4 | Solute Carrier Family 10 Member 4 |
| PARP15 | Poly(ADP-Ribose) Polymerase Family Member 15 |
| PARP16 | Poly(ADP-Ribose) Polymerase Family Member 16 |
| SUSD1 | Sushi Domain Containing 1 |
| TBC1D17 | TBC1 Domain Family Member 17 |
| TGFBRAP1 | Transforming Growth Factor Beta Receptor Associated Protein 1 |
| KCNMB2 | Potassium Calcium-Activated Channel Subfamily M Regulatory Beta Subunit 2 |
| PEAR1 | Platelet Endothelial Aggregation Receptor 1 |
| PCBP3 | Poly(RC) Binding Protein 3 |
| PCDHA12 | Protocadherin Alpha 12 |
| SRGAP2 | SLIT-ROBO Rho GTPase Activating Protein 2 |
| PRDM7 | PR/SET Domain 7 |
| SPDYA | Speedy/RINGO Cell Cycle Regulator Family Member A |
| SNAPC1 | Small Nuclear RNA Activating Complex Polypeptide 1 |
| POLDIP2 | DNA Polymerase Delta Interacting Protein 2 |
| SNED1 | Sushi, Nidogen And EGF Like Domains 1 |
| PHF21B | PHD Finger Protein 21B |
| SLC17A2 | Solute Carrier Family 17 Member 2 |
| SERTAD1 | SERTA Domain Containing 1 |
| SNX7 | Sorting Nexin 7 |
| SNX32 | Sorting Nexin 32 |
| SNX12 | Sorting Nexin 12 |
| JSRP1 | Junctional Sarcoplasmic Reticulum Protein 1 |
| RASGEF1C | RasGEF Domain Family Member 1C |
| TECRL | Trans-2,3-Enoyl-CoA Reductase Like |
| JADE1 | Jade Family PHD Finger 1 |
| TBC1D22B | TBC1 Domain Family Member 22B |
| TBCEL | Tubulin Folding Cofactor E Like |
| SLC17A4 | Solute Carrier Family 17 Member 4 |
| PARD3B | Par-3 Family Cell Polarity Regulator Beta |
| PCDH11X | Protocadherin 11 X-Linked |
| SYBU | Syntabulin |
| PWWP2A | PWWP Domain Containing 2A |
| IPCEF1 | Interaction Protein For Cytohesin Exchange Factors 1 |
| PTTG1IP | PTTG1 Interacting Protein |
| TMCC2 | Transmembrane And Coiled-Coil Domain Family 2 |
| TMEM38A | Transmembrane Protein 38A |
| PRSS33 | Serine Protease 33 |
| PRSS35 | Serine Protease 35 |
| SPIRE2 | Spire Type Actin Nucleation Factor 2 |
| PRPF18 | Pre-MRNA Processing Factor 18 |
| STRN4 | Striatin 4 |
| C1QL3 | Complement C1q Like 3 |
| GSG1 | Germ Cell Associated 1 |
| CNKSR3 | CNKSR Family Member 3 |
| CNNM1 | Cyclin And CBS Domain Divalent Metal Cation Transport Mediator 1 |
| CNPY2 | Canopy FGF Signaling Regulator 2 |
| GPX6 | Glutathione Peroxidase 6 |
| CLVS2 | Clavesin 2 |
| GTF3C3 | General Transcription Factor IIIC Subunit 3 |
| CMTM3 | CKLF Like MARVEL Transmembrane Domain Containing 3 |
| HMG20B | High Mobility Group 20B |
| CTDNEP1 | CTD Nuclear Envelope Phosphatase 1 |
| CUEDC1 | CUE Domain Containing 1 |
| HHIPL2 | HHIP Like 2 |
| RIC8A | RIC8 Guanine Nucleotide Exchange Factor A |
| ECHDC2 | Enoyl-CoA Hydratase Domain Containing 2 |
| EDEM3 | ER Degradation Enhancing Alpha-Mannosidase Like Protein 3 |
| RNF38 | Ring Finger Protein 38 |
| DZIP1 | DAZ Interacting Zinc Finger Protein 1 |
| E2F8 | E2F Transcription Factor 8 |
| N4BP2L2 | NEDD4 Binding Protein 2 Like 2 |
| HEBP1 | Heme Binding Protein 1 |
| HOXC10 | Homeobox C10 |
| MREG | Melanoregulin |
| MRGPRF | MAS Related GPR Family Member F |
| DENND4A | DENN Domain Containing 4A |
| HORMAD2 | HORMA Domain Containing 2 |
| DCBLD1 | Discoidin, CUB And LCCL Domain Containing 1 |
| DMWD | DM1 Locus, WD Repeat Containing |
| OR11A1 | Olfactory Receptor Family 11 Subfamily A Member 1 |
| S100G | S100 Calcium Binding Protein G |
| SARS1 | Seryl-TRNA Synthetase 1 |
| SDHAF1 | Succinate Dehydrogenase Complex Assembly Factor 1 |
| SDK2 | Sidekick Cell Adhesion Molecule 2 |
| SBK1 | SH3 Domain Binding Kinase 1 |
| SCAMP4 | Secretory Carrier Membrane Protein 4 |
| SCEL | Sciellin |
| SCML4 | Scm Polycomb Group Protein Like 4 |
| SERINC2 | Serine Incorporator 2 |
| GPR6 | G Protein-Coupled Receptor 6 |
| GPR63 | G Protein-Coupled Receptor 63 |
| GPATCH4 | G-Patch Domain Containing 4 |
| CENPO | Centromere Protein O |
| CEP350 | Centrosomal Protein 350 |
| MTRF1L | Mitochondrial Translational Release Factor 1 Like |
| MYO1H | Myosin IH |
| NKD1 | NKD Inhibitor Of WNT Signaling Pathway 1 |
| EMILIN2 | Elastin Microfibril Interfacer 2 |
| NRSN1 | Neurensin 1 |
| OGFOD1 | 2-Oxoglutarate And Iron Dependent Oxygenase Domain Containing 1 |
| RUSC1 | RUN And SH3 Domain Containing 1 |
| CDC123 | Cell Division Cycle 123 |
| GADD45GIP1 | GADD45G Interacting Protein 1 |
| CDIP1 | Cell Death Inducing P53 Target 1 |
| GEMIN7 | Gem Nuclear Organelle Associated Protein 7 |
| MFSD11 | Major Facilitator Superfamily Domain Containing 11 |
| FOXD1 | Forkhead Box D1 |
| MBOAT1 | Membrane Bound O-Acyltransferase Domain Containing 1 |
| MCUR1 | Mitochondrial Calcium Uniporter Regulator 1 |
| ABI3BP | ABI Family Member 3 Binding Protein |
| ADAM30 | ADAM Metallopeptidase Domain 30 |
| AFAP1L1 | Actin Filament Associated Protein 1 Like 1 |
| AGAP3 | ArfGAP With GTPase Domain, Ankyrin Repeat And PH Domain 3 |
| ADGRB3 | Adhesion G Protein-Coupled Receptor B3 |
| ADGRD1 | Adhesion G Protein-Coupled Receptor D1 |
| ADGRG2 | Adhesion G Protein-Coupled Receptor G2 |
| FAM76B | Family With Sequence Similarity 76 Member B |
| BCORL1 | BCL6 Corepressor Like 1 |
| ATRAID | All-Trans Retinoic Acid Induced Differentiation Factor |
| BUD13 | BUD13 Homolog |
| C12orf10 | Chromosome 12 Open Reading Frame 10 |
| LRRC8B | Leucine Rich Repeat Containing 8 VRAC Subunit B |
| LRRC8E | Leucine Rich Repeat Containing 8 VRAC Subunit E |
| LRFN4 | Leucine Rich Repeat And Fibronectin Type III Domain Containing 4 |
| MAD2L1BP | MAD2L1 Binding Protein |
| MAGI3 | Membrane Associated Guanylate Kinase, WW And PDZ Domain Containing 3 |
| AMELX | Amelogenin X-Linked |
| ARGLU1 | Arginine And Glutamate Rich 1 |
| ARL5A | ADP Ribosylation Factor Like GTPase 5A |
| ARMC2 | Armadillo Repeat Containing 2 |
| ARHGAP42 | Rho GTPase Activating Protein 42 |
| ERMP1 | Endoplasmic Reticulum Metallopeptidase 1 |
| ANKZF1 | Ankyrin Repeat And Zinc Finger Peptidyl TRNA Hydrolase 1 |
| BCL9L | BCL9 Like |
| LGALSL | Galectin Like |
| ZDHHC21 | Zinc Finger DHHC-Type Palmitoyltransferase 21 |
| ZFAND2B | Zinc Finger AN1-Type Containing 2B |
| ZNF165 | Zinc Finger Protein 165 |
| ZNF174 | Zinc Finger Protein 174 |
| ZNF195 | Zinc Finger Protein 195 |
| ZNF185 | Zinc Finger Protein 185 With LIM Domain |
| LHX9 | LIM Homeobox 9 |
| UBP1 | Upstream Binding Protein 1 |
| UBTD2 | Ubiquitin Domain Containing 2 |
| URM1 | Ubiquitin Related Modifier 1 |
| USHBP1 | USH1 Protein Network Component Harmonin Binding Protein 1 |
| WBP1 | WW Domain Binding Protein 1 |
| USP40 | Ubiquitin Specific Peptidase 40 |
| ZBTB25 | Zinc Finger And BTB Domain Containing 25 |
| U2AF1L4 | U2 Small Nuclear RNA Auxiliary Factor 1 Like 4 |
| KRT79 | Keratin 79 |
| TTBK1 | Tau Tubulin Kinase 1 |
| TRIM7 | Tripartite Motif Containing 7 |
| PGBD1 | PiggyBac Transposable Element Derived 1 |
| PHACTR3 | Phosphatase And Actin Regulator 3 |
| SMG5 | SMG5 Nonsense Mediated MRNA Decay Factor |
| PDZK1IP1 | PDZK1 Interacting Protein 1 |
| SLC35B3 | Solute Carrier Family 35 Member B3 |
| SLC35F1 | Solute Carrier Family 35 Member F1 |
| PCDHA3 | Protocadherin Alpha 3 |
| ZNF778 | Zinc Finger Protein 778 |
| ZSWIM5 | Zinc Finger SWIM-Type Containing 5 |
| ZNF292 | Zinc Finger Protein 292 |
| ZNF263 | Zinc Finger Protein 263 |
| CLDND1 | Claudin Domain Containing 1 |
| TAAR6 | Trace Amine Associated Receptor 6 |
| TFAP2D | Transcription Factor AP-2 Delta |
| XIRP1 | Xin Actin Binding Repeat Containing 1 |
| RNF145 | Ring Finger Protein 145 |
| NDUFAF7 | NADH:Ubiquinone Oxidoreductase Complex Assembly Factor 7 |
| NPEPL1 | Aminopeptidase Like 1 |
| NRM | Nurim |
| RBM47 | RNA Binding Motif Protein 47 |
| THAP7 | THAP Domain Containing 7 |
| SPSB3 | SplA/Ryanodine Receptor Domain And SOCS Box Containing 3 |
| SLC35A5 | Solute Carrier Family 35 Member A5 |
| PCDHA1 | Protocadherin Alpha 1 |
| PCDHA10 | Protocadherin Alpha 10 |
| SRL | Sarcalumenin |
| SRRM4 | Serine/Arginine Repetitive Matrix 4 |
| PKD1L2 | Polycystin 1 Like 2 (Gene/Pseudogene) |
| PLEKHA6 | Pleckstrin Homology Domain Containing A6 |
| SOCS7 | Suppressor Of Cytokine Signaling 7 |
| HSBP1 | Heat Shock Factor Binding Protein 1 |
| PIP5KL1 | Phosphatidylinositol-4-Phosphate 5-Kinase Like 1 |
| IZUMO1 | Izumo Sperm-Egg Fusion 1 |
| KANSL2 | KAT8 Regulatory NSL Complex Subunit 2 |
| KANSL3 | KAT8 Regulatory NSL Complex Subunit 3 |
| ITLN2 | Intelectin 2 |
| JKAMP | JNK1/MAPK8 Associated Membrane Protein |
| JMJD7 | Jumonji Domain Containing 7 |
| SH3RF3 | SH3 Domain Containing Ring Finger 3 |
| PAQR4 | Progestin And AdipoQ Receptor Family Member 4 |
| INTS2 | Integrator Complex Subunit 2 |
| R3HDM1 | R3H Domain Containing 1 |
| PURG | Purine Rich Element Binding Protein G |
| KIAA1191 | KIAA1191 |
| TMC5 | Transmembrane Channel Like 5 |
| TMCO3 | Transmembrane And Coiled-Coil Domains 3 |
| TMEM161A | Transmembrane Protein 161A |
| TMEM214 | Transmembrane Protein 214 |
| TMEM87B | Transmembrane Protein 87B |
| PPP6R2 | Protein Phosphatase 6 Regulatory Subunit 2 |
| PRTFDC1 | Phosphoribosyl Transferase Domain Containing 1 |
| PRPSAP2 | Phosphoribosyl Pyrophosphate Synthetase Associated Protein 2 |
| GSE1 | Gse1 Coiled-Coil Protein |
| RGP1 | RGP1 Homolog, RAB6A GEF Complex Partner 1 |
| CTTNBP2NL | CTTNBP2 N-Terminal Like |
| DALRD3 | DALR Anticodon Binding Domain Containing 3 |
| MOB2 | MOB Kinase Activator 2 |
| NDFIP2 | Nedd4 Family Interacting Protein 2 |
| EBF4 | EBF Family Member 4 |
| N4BP2 | NEDD4 Binding Protein 2 |
| NAA40 | N-Alpha-Acetyltransferase 40, NatD Catalytic Subunit |
| HOXC11 | Homeobox C11 |
| DCUN1D4 | Defective In Cullin Neddylation 1 Domain Containing 4 |
| DEF8 | Differentially Expressed In FDCP 8 Homolog |
| DENND2D | DENN Domain Containing 2D |
| MTCL1 | Microtubule Crosslinking Factor 1 |
| DCAF6 | DDB1 And CUL4 Associated Factor 6 |
| RMND5B | Required For Meiotic Nuclear Division 5 Homolog B |
| OR2B6 | Olfactory Receptor Family 2 Subfamily B Member 6 |
| SCAF8 | SR-Related CTD Associated Factor 8 |
| SERHL2 | Serine Hydrolase Like 2 |
| GPR52 | G Protein-Coupled Receptor 52 |
| CHP2 | Calcineurin Like EF-Hand Protein 2 |
| GLYATL2 | Glycine-N-Acyltransferase Like 2 |
| MXD3 | MAX Dimerization Protein 3 |
| DNPH1 | 2'-Deoxynucleoside 5'-Phosphate N-Hydrolase 1 |
| DUS3L | Dihydrouridine Synthase 3 Like |
| DUSP11 | Dual Specificity Phosphatase 11 |
| RIMS4 | Regulating Synaptic Membrane Exocytosis 4 |
| NIPSNAP3B | Nipsnap Homolog 3B |
| ROPN1 | Rhophilin Associated Tail Protein 1 |
| NOBOX | NOBOX Oogenesis Homeobox |
| NT5DC1 | 5'-Nucleotidase Domain Containing 1 |
| OPN5 | Opsin 5 |
| OARD1 | O-Acyl-ADP-Ribose Deacylase 1 |
| SAMD11 | Sterile Alpha Motif Domain Containing 11 |
| OIT3 | Oncoprotein Induced Transcript 3 |
| GAL3ST2 | Galactose-3-O-Sulfotransferase 2 |
| GET4 | Guided Entry Of Tail-Anchored Proteins Factor 4 |
| FOXD4 | Forkhead Box D4 |
| FUT11 | Fucosyltransferase 11 |
| MED31 | Mediator Complex Subunit 31 |
| FOXS1 | Forkhead Box S1 |
| ADGRL3 | Adhesion G Protein-Coupled Receptor L3 |
| BARHL1 | BarH Like Homeobox 1 |
| ATXN1L | Ataxin 1 Like |
| EXOC6B | Exocyst Complex Component 6B |
| C18orf25 | Chromosome 18 Open Reading Frame 25 |
| FBXO45 | F-Box Protein 45 |
| FILIP1 | Filamin A Interacting Protein 1 |
| LVRN | Laeverin |
| LRRC8C | Leucine Rich Repeat Containing 8 VRAC Subunit C |
| LRP11 | LDL Receptor Related Protein 11 |
| LRRC14 | Leucine Rich Repeat Containing 14 |
| C1QTNF9 | C1q And TNF Related 9 |
| C20orf194 | Chromosome 20 Open Reading Frame 194 |
| MALSU1 | Mitochondrial Assembly Of Ribosomal Large Subunit 1 |
| ANKRD52 | Ankyrin Repeat Domain 52 |
| ANKS3 | Ankyrin Repeat And Sterile Alpha Motif Domain Containing 3 |
| EPB41L4A | Erythrocyte Membrane Protein Band 4.1 Like 4A |
| AGFG2 | ArfGAP With FG Repeats 2 |
| FAM114A1 | Family With Sequence Similarity 114 Member A1 |
| KRT36 | Keratin 36 |
| TMEM132E | Transmembrane Protein 132E |
| TM7SF3 | Transmembrane 7 Superfamily Member 3 |
| TRAPPC8 | Trafficking Protein Particle Complex 8 |
| LHFPL5 | LHFPL Tetraspan Subfamily Member 5 |
| TXNDC11 | Thioredoxin Domain Containing 11 |
| ZDHHC23 | Zinc Finger DHHC-Type Palmitoyltransferase 23 |
| ZKSCAN3 | Zinc Finger With KRAB And SCAN Domains 3 |
| ZKSCAN4 | Zinc Finger With KRAB And SCAN Domains 4 |
| ZMIZ2 | Zinc Finger MIZ-Type Containing 2 |
| ZNF124 | Zinc Finger Protein 124 |
| ZNF200 | Zinc Finger Protein 200 |
| UNC93A | Unc-93 Homolog A |
| WIZ | WIZ Zinc Finger |
| VPS36 | Vacuolar Protein Sorting 36 Homolog |
| TTYH3 | Tweety Family Member 3 |
| TRPT1 | TRNA Phosphotransferase 1 |
| TTC28 | Tetratricopeptide Repeat Domain 28 |
| TRIM66 | Tripartite Motif Containing 66 |
| TRMT6 | TRNA Methyltransferase 6 |
| PID1 | Phosphotyrosine Interaction Domain Containing 1 |
| SMYD5 | SMYD Family Member 5 |
| PHTF2 | Putative Homeodomain Transcription Factor 2 |
| SLC35D3 | Solute Carrier Family 35 Member D3 |
| PCDHA6 | Protocadherin Alpha 6 |
| PCDHA9 | Protocadherin Alpha 9 |
| PCDHAC2 | Protocadherin Alpha Subfamily C, 2 |
| ZNF791 | Zinc Finger Protein 791 |
| ZNF674 | Zinc Finger Protein 674 |
| ZNF785 | Zinc Finger Protein 785 |
| ZNF266 | Zinc Finger Protein 266 |
| ZNF273 | Zinc Finger Protein 273 |
| ZNF407 | Zinc Finger Protein 407 |
| ZNF567 | Zinc Finger Protein 567 |
| TAAR5 | Trace Amine Associated Receptor 5 |
| LIPT2 | Lipoyl(Octanoyl) Transferase 2 |
| ZSCAN18 | Zinc Finger And SCAN Domain Containing 18 |
| SERPINA11 | Serpin Family A Member 11 |
| TBC1D16 | TBC1 Domain Family Member 16 |
| RBM41 | RNA Binding Motif Protein 41 |
| SPZ1 | Spermatogenic Leucine Zipper 1 |
| PRH1 | Proline Rich Protein HaeIII Subfamily 1 |
| PM20D2 | Peptidase M20 Domain Containing 2 |
| PNLDC1 | PARN Like, Ribonuclease Domain Containing 1 |
| PPIL6 | Peptidylprolyl Isomerase Like 6 |
| SPATA17 | Spermatogenesis Associated 17 |
| KAZN | Kazrin, Periplakin Interacting Protein |
| RASL10B | RAS Like Family 10 Member B |
| TBC1D2B | TBC1 Domain Family Member 2B |
| SLC26A10 | Solute Carrier Family 26 Member 10 |
| SH3BP5L | SH3 Binding Domain Protein 5 Like |
| TAF1L | TATA-Box Binding Protein Associated Factor 1 Like |
| KIAA0825 | KIAA0825 |
| TMCO5A | Transmembrane And Coiled-Coil Domains 5A |
| KLF16 | Kruppel Like Factor 16 |
| TMEM184A | Transmembrane Protein 184A |
| TMEM41B | Transmembrane Protein 41B |
| KLHDC10 | Kelch Domain Containing 10 |
| TMEM120B | Transmembrane Protein 120B |
| GTF3C6 | General Transcription Factor IIIC Subunit 6 |
| MYCT1 | MYC Target 1 |
| NACC2 | NACC Family Member 2 |
| RNF39 | Ring Finger Protein 39 |
| HECA | Hdc Homolog, Cell Cycle Regulator |
| DDX53 | DEAD-Box Helicase 53 |
| DCAF5 | DDB1 And CUL4 Associated Factor 5 |
| OR2W1 | Olfactory Receptor Family 2 Subfamily W Member 1 |
| SCRT1 | Scratch Family Transcriptional Repressor 1 |
| OR2J3 | Olfactory Receptor Family 2 Subfamily J Member 3 |
| CHTF8 | Chromosome Transmission Fidelity Factor 8 |
| CENPW | Centromere Protein W |
| C1orf109 | Chromosome 1 Open Reading Frame 109 |
| DPY19L1 | Dpy-19 Like C-Mannosyltransferase 1 |
| NKX2-4 | NK2 Homeobox 4 |
| SAMD3 | Sterile Alpha Motif Domain Containing 3 |
| DLK2 | Delta Like Non-Canonical Notch Ligand 2 |
| GINS3 | GINS Complex Subunit 3 |
| GLI4 | GLI Family Zinc Finger 4 |
| CCN6 | Cellular Communication Network Factor 6 |
| METRN | Meteorin, Glial Cell Differentiation Regulator |
| CCDC77 | Coiled-Coil Domain Containing 77 |
| ABRACL | ABRA C-Terminal Like |
| FANK1 | Fibronectin Type III And Ankyrin Repeat Domains 1 |
| ATP1B4 | ATPase Na+/K+ Transporting Family Member Beta 4 |
| EVX2 | Even-Skipped Homeobox 2 |
| FAM102A | Family With Sequence Similarity 102 Member A |
| C12orf43 | Chromosome 12 Open Reading Frame 43 |
| CAPS2 | Calcyphosine 2 |
| ANKRD53 | Ankyrin Repeat Domain 53 |
| ADGRG7 | Adhesion G Protein-Coupled Receptor G7 |
| TMEM101 | Transmembrane Protein 101 |
| TRIM16 | Tripartite Motif Containing 16 |
| TTC30A | Tetratricopeptide Repeat Domain 30A |
| WDR76 | WD Repeat Domain 76 |
| UQCC1 | Ubiquinol-Cytochrome C Reductase Complex Assembly Factor 1 |
| ZFP2 | ZFP2 Zinc Finger Protein |
| ZKSCAN8 | Zinc Finger With KRAB And SCAN Domains 8 |
| UBXN2A | UBX Domain Protein 2A |
| XKR6 | XK Related 6 |
| WDR91 | WD Repeat Domain 91 |
| LENG8 | Leukocyte Receptor Cluster Member 8 |
| TSPAN17 | Tetraspanin 17 |
| PGA3 | Pepsinogen A3 |
| PGA5 | Pepsinogen A5 |
| ZNF92 | Zinc Finger Protein 92 |
| ZSCAN25 | Zinc Finger And SCAN Domain Containing 25 |
| ZZZ3 | Zinc Finger ZZ-Type Containing 3 |
| ZNF467 | Zinc Finger Protein 467 |
| ZNF484 | Zinc Finger Protein 484 |
| SFT2D2 | SFT2 Domain Containing 2 |
| THUMPD2 | THUMP Domain Containing 2 |
| KHDC1 | KH Domain Containing 1 |
| THNSL2 | Threonine Synthase Like 2 |
| SPTSSA | Serine Palmitoyltransferase Small Subunit A |
| PCDHA11 | Protocadherin Alpha 11 |
| PCDHA13 | Protocadherin Alpha 13 |
| SRFBP1 | Serum Response Factor Binding Protein 1 |
| PIWIL3 | Piwi Like RNA-Mediated Gene Silencing 3 |
| PLD6 | Phospholipase D Family Member 6 |
| PLEKHH3 | Pleckstrin Homology, MyTH4 And FERM Domain Containing H3 |
| HUS1B | HUS1 Checkpoint Clamp Component B |
| TCTA | T Cell Leukemia Translocation Altered |
| JRKL | JRK Like |
| TAS2R40 | Taste 2 Receptor Member 40 |
| RAB6C | RAB6C, Member RAS Oncogene Family |
| TBC1D30 | TBC1 Domain Family Member 30 |
| P3H3 | Prolyl 3-Hydroxylase 3 |
| PCDH20 | Protocadherin 20 |
| PUSL1 | Pseudouridine Synthase Like 1 |
| TEX2 | Testis Expressed 2 |
| KLHL32 | Kelch Like Family Member 32 |
| TMEM200A | Transmembrane Protein 200A |
| TMEM245 | Transmembrane Protein 245 |
| TMEM25 | Transmembrane Protein 25 |
| TMEM53 | Transmembrane Protein 53 |
| TMEM63B | Transmembrane Protein 63B |
| TMEM132C | Transmembrane Protein 132C |
| PRR3 | Proline Rich 3 |
| GSX2 | GS Homeobox 2 |
| GTF2H2C | GTF2H2 Family Member C |
| CMC2 | C-X9-C Motif Containing 2 |
| CNEP1R1 | CTD Nuclear Envelope Phosphatase 1 Regulatory Subunit 1 |
| HMGN3 | High Mobility Group Nucleosomal Binding Domain 3 |
| MORC4 | MORC Family CW-Type Zinc Finger 4 |
| CST9 | Cystatin 9 |
| HINT3 | Histidine Triad Nucleotide Binding Protein 3 |
| MOB3C | MOB Kinase Activator 3C |
| MYCBPAP | MYCBP Associated Protein |
| ECT2L | Epithelial Cell Transforming 2 Like |
| HDDC2 | HD Domain Containing 2 |
| DMRTC2 | DMRT Like Family C2 |
| MTFR2 | Mitochondrial Fission Regulator 2 |
| RWDD2A | RWD Domain Containing 2A |
| OR5V1 | Olfactory Receptor Family 5 Subfamily V Member 1 |
| OR2B2 | Olfactory Receptor Family 2 Subfamily B Member 2 |
| OR2B3 | Olfactory Receptor Family 2 Subfamily B Member 3 |
| OR2J2 | Olfactory Receptor Family 2 Subfamily J Member 2 |
| GNRHR2 | Gonadotropin Releasing Hormone Receptor 2 (Pseudogene) |
| GOLGA7B | Golgin A7 Family Member B |
| CHRFAM7A | CHRNA7 (Exons 5-10) And FAM7A (Exons A-E) Fusion |
| GPAT4 | Glycerol-3-Phosphate Acyltransferase 4 |
| CEP57L1 | Centrosomal Protein 57 Like 1 |
| EMC8 | ER Membrane Protein Complex Subunit 8 |
| OR2A4 | Olfactory Receptor Family 2 Subfamily A Member 4 |
| RSBN1 | Round Spermatid Basic Protein 1 |
| RSBN1L | Round Spermatid Basic Protein 1 Like |
| OGFRL1 | Opioid Growth Factor Receptor Like 1 |
| CEACAM16 | CEA Cell Adhesion Molecule 16, Tectorial Membrane Component |
| GJB7 | Gap Junction Protein Beta 7 |
| GAS2L3 | Growth Arrest Specific 2 Like 3 |
| CCDC174 | Coiled-Coil Domain Containing 174 |
| CCDC62 | Coiled-Coil Domain Containing 62 |
| FAM8A1 | Family With Sequence Similarity 8 Member A1 |
| BEND3 | BEN Domain Containing 3 |
| ERI2 | ERI1 Exoribonuclease Family Member 2 |
| ARMT1 | Acidic Residue Methyltransferase 1 |
| FAM174B | Family With Sequence Similarity 174 Member B |
| FAM131A | Family With Sequence Similarity 131 Member A |
| FAM168B | Family With Sequence Similarity 168 Member B |
| C16orf58 | Chromosome 16 Open Reading Frame 58 |
| C17orf49 | Chromosome 17 Open Reading Frame 49 |
| C19orf44 | Chromosome 19 Open Reading Frame 44 |
| C19orf48 | Chromosome 19 Open Reading Frame 48 |
| C6orf62 | Chromosome 6 Open Reading Frame 62 |
| C4orf19 | Chromosome 4 Open Reading Frame 19 |
| C1QTNF8 | C1q And TNF Related 8 |
| CARD18 | Caspase Recruitment Domain Family Member 18 |
| MAP3K7CL | MAP3K7 C-Terminal Like |
| AMY1B | Amylase Alpha 1B |
| AMY1C | Amylase Alpha 1C |
| ANGEL2 | Angel Homolog 2 |
| ANKEF1 | Ankyrin Repeat And EF-Hand Domain Containing 1 |
| ANKRD13C | Ankyrin Repeat Domain 13C |
| ERO1B | Endoplasmic Reticulum Oxidoreductase 1 Beta |
| FATE1 | Fetal And Adult Testis Expressed 1 |
| BLCAP | BLCAP Apoptosis Inducing Factor |
| KLHL31 | Kelch Like Family Member 31 |
| KIAA1958 | KIAA1958 |
| VGLL2 | Vestigial Like Family Member 2 |
| TSTD1 | Thiosulfate Sulfurtransferase Like Domain Containing 1 |
| ZKSCAN7 | Zinc Finger With KRAB And SCAN Domains 7 |
| ZNF385D | Zinc Finger Protein 385D |
| UNCX | UNC Homeobox |
| ZMAT2 | Zinc Finger Matrin-Type 2 |
| ZNF232 | Zinc Finger Protein 232 |
| UGT1A5 | UDP Glucuronosyltransferase Family 1 Member A5 |
| WDR13 | WD Repeat Domain 13 |
| WDR24 | WD Repeat Domain 24 |
| USPL1 | Ubiquitin Specific Peptidase Like 1 |
| VOPP1 | VOPP1 WW Domain Binding Protein |
| VPS37D | VPS37D Subunit Of ESCRT-I |
| ZCCHC24 | Zinc Finger CCHC-Type Containing 24 |
| UBAP2 | Ubiquitin Associated Protein 2 |
| TMEM240 | Transmembrane Protein 240 |
| TSPYL4 | TSPY Like 4 |
| TIGD4 | Tigger Transposable Element Derived 4 |
| SMAGP | Small Cell Adhesion Glycoprotein |
| PHACTR4 | Phosphatase And Actin Regulator 4 |
| PCDHA5 | Protocadherin Alpha 5 |
| PCDHA7 | Protocadherin Alpha 7 |
| PCDHAC1 | Protocadherin Alpha Subfamily C, 1 |
| PCDHGB1 | Protocadherin Gamma Subfamily B, 1 |
| ZNF90 | Zinc Finger Protein 90 |
| ZSCAN32 | Zinc Finger And SCAN Domain Containing 32 |
| ZSCAN12 | Zinc Finger And SCAN Domain Containing 12 |
| ZSCAN4 | Zinc Finger And SCAN Domain Containing 4 |
| ZSCAN16 | Zinc Finger And SCAN Domain Containing 16 |
| ZNF747 | Zinc Finger Protein 747 |
| ZNF768 | Zinc Finger Protein 768 |
| ZNF226 | Zinc Finger Protein 226 |
| ZNF311 | Zinc Finger Protein 311 |
| ZNF354B | Zinc Finger Protein 354B |
| ZNF605 | Zinc Finger Protein 605 |
| ZACN | Zinc Activated Ion Channel |
| NME1-NME2 | NME1-NME2 Readthrough |
| SUSD6 | Sushi Domain Containing 6 |
| RELL1 | RELT Like 1 |
| SPRYD3 | SPRY Domain Containing 3 |
| PRH2 | Proline Rich Protein HaeIII Subfamily 2 |
| SLC35A4 | Solute Carrier Family 35 Member A4 |
| SRSF12 | Serine And Arginine Rich Splicing Factor 12 |
| SPIN4 | Spindlin Family Member 4 |
| PLEKHG1 | Pleckstrin Homology And RhoGEF Domain Containing G1 |
| JMJD8 | Jumonji Domain Containing 8 |
| TCP10 | T-Complex 10 |
| SLC18B1 | Solute Carrier Family 18 Member B1 |
| TAAR8 | Trace Amine Associated Receptor 8 |
| IQUB | IQ Motif And Ubiquitin Domain Containing |
| TMEM17 | Transmembrane Protein 17 |
| STARD9 | StAR Related Lipid Transfer Domain Containing 9 |
| PRR5L | Proline Rich 5 Like |
| SOGA1 | Suppressor Of Glucose, Autophagy Associated 1 |
| CCDC7 | Coiled-Coil Domain Containing 7 |
| CMSS1 | Cms1 Ribosomal Small Subunit Homolog |
| MISP | Mitotic Spindle Positioning |
| CYLC1 | Cylicin 1 |
| RGSL1 | Regulator Of G Protein Signaling Like 1 |
| RHOXF1 | Rhox Homeobox Family Member 1 |
| NCKAP5L | NCK Associated Protein 5 Like |
| MZT2B | Mitotic Spindle Organizing Protein 2B |
| CRACR2B | Calcium Release Activated Channel Regulator 2B |
| COX8C | Cytochrome C Oxidase Subunit 8C |
| DENND4B | DENN Domain Containing 4B |
| MS4A6E | Membrane Spanning 4-Domains A6E |
| DCDC1 | Doublecortin Domain Containing 1 |
| OR12D2 | Olfactory Receptor Family 12 Subfamily D Member 2 (Gene/Pseudogene) |
| OR12D3 | Olfactory Receptor Family 12 Subfamily D Member 3 |
| OR10C1 | Olfactory Receptor Family 10 Subfamily C Member 1 (Gene/Pseudogene) |
| OTOP1 | Otopetrin 1 |
| SEC22C | SEC22 Homolog C, Vesicle Trafficking Protein |
| SERF1A | Small EDRK-Rich Factor 1A |
| GPR75 | G Protein-Coupled Receptor 75 |
| CEP95 | Centrosomal Protein 95 |
| GPBP1L1 | GC-Rich Promoter Binding Protein 1 Like 1 |
| DTWD2 | DTW Domain Containing 2 |
| EMC6 | ER Membrane Protein Complex Subunit 6 |
| OCM | Oncomodulin |
| GGTLC2 | Gamma-Glutamyltransferase Light Chain 2 |
| CDV3 | CDV3 Homolog |
| CDADC1 | Cytidine And DCMP Deaminase Domain Containing 1 |
| METTL8 | Methyltransferase Like 8 |
| CCDC112 | Coiled-Coil Domain Containing 112 |
| CCDC121 | Coiled-Coil Domain Containing 121 |
| CCDC125 | Coiled-Coil Domain Containing 125 |
| CCDC83 | Coiled-Coil Domain Containing 83 |
| MCCD1 | Mitochondrial Coiled-Coil Domain 1 |
| FREM3 | FRAS1 Related Extracellular Matrix 3 |
| CCDC28A | Coiled-Coil Domain Containing 28A |
| CCDC38 | Coiled-Coil Domain Containing 38 |
| ADTRP | Androgen Dependent TFPI Regulating Protein |
| FAM50B | Family With Sequence Similarity 50 Member B |
| BEND6 | BEN Domain Containing 6 |
| AWAT2 | Acyl-CoA Wax Alcohol Acyltransferase 2 |
| FBRSL1 | Fibrosin Like 1 |
| C4orf33 | Chromosome 4 Open Reading Frame 33 |
| LRIF1 | Ligand Dependent Nuclear Receptor Interacting Factor 1 |
| C1orf87 | Chromosome 1 Open Reading Frame 87 |
| LRRC61 | Leucine Rich Repeat Containing 61 |
| ARL14 | ADP Ribosylation Factor Like GTPase 14 |
| ANKRD22 | Ankyrin Repeat Domain 22 |
| ANKRD34B | Ankyrin Repeat Domain 34B |
| ATAD3C | ATPase Family AAA Domain Containing 3C |
| FAXC | Failed Axon Connections Homolog, Metaxin Like GST Domain Containing |
| BOLA2 | BolA Family Member 2 |
| MAGEA2 | MAGE Family Member A2 |
| C2orf88 | Chromosome 2 Open Reading Frame 88 |
| TMEM141 | Transmembrane Protein 141 |
| WDR27 | WD Repeat Domain 27 |
| ZFY | Zinc Finger Protein Y-Linked |
| LGALS9C | Galectin 9C |
| TXNDC16 | Thioredoxin Domain Containing 16 |
| VWA7 | Von Willebrand Factor A Domain Containing 7 |
| ZBTB3 | Zinc Finger And BTB Domain Containing 3 |
| ZBTB41 | Zinc Finger And BTB Domain Containing 41 |
| ZBTB6 | Zinc Finger And BTB Domain Containing 6 |
| ZC3H12B | Zinc Finger CCCH-Type Containing 12B |
| LEMD1 | LEM Domain Containing 1 |
| PCDHA4 | Protocadherin Alpha 4 |
| ZNF787 | Zinc Finger Protein 787 |
| ZSWIM3 | Zinc Finger SWIM-Type Containing 3 |
| ZNF383 | Zinc Finger Protein 383 |
| ZNF584 | Zinc Finger Protein 584 |
| ZNF440 | Zinc Finger Protein 440 |
| TCP11L1 | T-Complex 11 Like 1 |
| LINGO3 | Leucine Rich Repeat And Ig Domain Containing 3 |
| CREG2 | Cellular Repressor Of E1A Stimulated Genes 2 |
| PABPC4L | Poly(A) Binding Protein Cytoplasmic 4 Like |
| SRRM3 | Serine/Arginine Repetitive Matrix 3 |
| IFI27L2 | Interferon Alpha Inducible Protein 27 Like 2 |
| PLEKHG7 | Pleckstrin Homology And RhoGEF Domain Containing G7 |
| PITHD1 | PITH Domain Containing 1 |
| SERPINE3 | Serpin Family E Member 3 |
| SPANXC | SPANX Family Member C |
| TENT4A | Terminal Nucleotidyltransferase 4A |
| SHISA7 | Shisa Family Member 7 |
| PATE1 | Prostate And Testis Expressed 1 |
| PXDC1 | PX Domain Containing 1 |
| QSER1 | Glutamine And Serine Rich 1 |
| SZRD1 | SUZ RNA Binding Domain Containing 1 |
| KIAA1522 | KIAA1522 |
| TMEM151B | Transmembrane Protein 151B |
| TMEM185A | Transmembrane Protein 185A |
| TMEM217 | Transmembrane Protein 217 |
| PSMG4 | Proteasome Assembly Chaperone 4 |
| CLEC2L | C-Type Lectin Domain Family 2 Member L |
| H1-6 | H1.6 Linker Histone, Cluster Member |
| CITED4 | Cbp/P300 Interacting Transactivator With Glu/Asp Rich Carboxy-Terminal Domain 4 |
| CXorf38 | Chromosome X Open Reading Frame 38 |
| HMGN5 | High Mobility Group Nucleosome Binding Domain 5 |
| RITA1 | RBPJ Interacting And Tubulin Associated 1 |
| DAOA | D-Amino Acid Oxidase Activator |
| NIBAN2 | Niban Apoptosis Regulator 2 |
| NAT8B | N-Acetyltransferase 8B (Putative, Gene/Pseudogene) |
| CRPPA | CDP-L-Ribitol Pyrophosphorylase A |
| MS4A15 | Membrane Spanning 4-Domains A15 |
| MSGN1 | Mesogenin 1 |
| MROH2B | Maestro Heat Like Repeat Family Member 2B |
| DDTL | D-Dopachrome Tautomerase Like |
| SELENOI | Selenoprotein I |
| SAYSD1 | SAYSVFN Motif Domain Containing 1 |
| DPPA5 | Developmental Pluripotency Associated 5 |
| RNASE11 | Ribonuclease A Family Member 11 (Inactive) |
| RNASE13 | Ribonuclease A Family Member 13 (Inactive) |
| DUSP28 | Dual Specificity Phosphatase 28 |
| DCAF10 | DDB1 And CUL4 Associated Factor 10 |
| NIPSNAP2 | Nipsnap Homolog 2 |
| EMC7 | ER Membrane Protein Complex Subunit 7 |
| RUFY4 | RUN And FYVE Domain Containing 4 |
| ODF3L2 | Outer Dense Fiber Of Sperm Tails 3 Like 2 |
| OR14J1 | Olfactory Receptor Family 14 Subfamily J Member 1 |
| NXPE4 | Neurexophilin And PC-Esterase Domain Family Member 4 |
| GINM1 | Glycoprotein Integral Membrane 1 |
| CEACAM19 | CEA Cell Adhesion Molecule 19 |
| MFSD4B | Major Facilitator Superfamily Domain Containing 4B |
| FAAP24 | FA Core Complex Associated Protein 24 |
| CCDC138 | Coiled-Coil Domain Containing 138 |
| ADPRS | ADP-Ribosylserine Hydrolase |
| ACTL9 | Actin Like 9 |
| FAM98A | Family With Sequence Similarity 98 Member A |
| ATXN7L3B | Ataxin 7 Like 3B |
| FAM102B | Family With Sequence Similarity 102 Member B |
| FAM207A | Family With Sequence Similarity 207 Member A |
| FAM219A | Family With Sequence Similarity 219 Member A |
| FAM162B | Family With Sequence Similarity 162 Member B |
| FBXO46 | F-Box Protein 46 |
| C6orf136 | Chromosome 6 Open Reading Frame 136 |
| C6orf201 | Chromosome 6 Open Reading Frame 201 |
| C6orf47 | Chromosome 6 Open Reading Frame 47 |
| C1orf226 | Chromosome 1 Open Reading Frame 226 |
| BPIFA3 | BPI Fold Containing Family A Member 3 |
| ARMC12 | Armadillo Repeat Containing 12 |
| TOMM6 | Translocase Of Outer Mitochondrial Membrane 6 |
| KIAA1549L | KIAA1549 Like |
| KIAA1614 | KIAA1614 |
| TRIM16L | Tripartite Motif Containing 16 Like |
| KRTAP2-1 | Keratin Associated Protein 2-1 |
| VWC2L | Von Willebrand Factor C Domain Containing 2 Like |
| TTLL2 | Tubulin Tyrosine Ligase Like 2 |
| TMEM241 | Transmembrane Protein 241 |
| PIRT | Phosphoinositide Interacting Regulator Of Transient Receptor Potential Channels |
| PCDHA8 | Protocadherin Alpha 8 |
| ZNF774 | Zinc Finger Protein 774 |
| ZSCAN1 | Zinc Finger And SCAN Domain Containing 1 |
| ZNF391 | Zinc Finger Protein 391 |
| SPATA21 | Spermatogenesis Associated 21 |
| NSG2 | Neuronal Vesicle Trafficking Associated 2 |
| SMIM8 | Small Integral Membrane Protein 8 |
| TENT5A | Terminal Nucleotidyltransferase 5A |
| RBBP8NL | RBBP8 N-Terminal Like |
| PTX4 | Pentraxin 4 |
| KIAA0408 | KIAA0408 |
| KLHL34 | Kelch Like Family Member 34 |
| TMEM215 | Transmembrane Protein 215 |
| PRRT3 | Proline Rich Transmembrane Protein 3 |
| GTPBP6 | GTP Binding Protein 6 (Putative) |
| RFX8 | RFX Family Member 8, Lacking RFX DNA Binding Domain |
| MORN2 | MORN Repeat Containing 2 |
| RHOXF2 | Rhox Homeobox Family Member 2 |
| HPDL | 4-Hydroxyphenylpyruvate Dioxygenase Like |
| SCGB1D4 | Secretoglobin Family 1D Member 4 |
| NPIPA1 | Nuclear Pore Complex Interacting Protein Family Member A1 |
| NOL4L | Nucleolar Protein 4 Like |
| NXPE1 | Neurexophilin And PC-Esterase Domain Family Member 1 |
| NXPE3 | Neurexophilin And PC-Esterase Domain Family Member 3 |
| CCZ1B | CCZ1 Homolog B, Vacuolar Protein Trafficking And Biogenesis Associated |
| CCDC167 | Coiled-Coil Domain Containing 167 |
| ADGB | Androglobin |
| ADGRG4 | Adhesion G Protein-Coupled Receptor G4 |
| C6orf163 | Chromosome 6 Open Reading Frame 163 |
| FSIP2 | Fibrous Sheath Interacting Protein 2 |
| KRTAP2-4 | Keratin Associated Protein 2-4 |
| VWA3A | Von Willebrand Factor A Domain Containing 3A |
| TTC13 | Tetratricopeptide Repeat Domain 13 |
| PGA4 | Pepsinogen A4 |
| ZNF720 | Zinc Finger Protein 720 |
| ZNF594 | Zinc Finger Protein 594 |
| ZNF501 | Zinc Finger Protein 501 |
| TFDP3 | Transcription Factor Dp Family Member 3 |
| PXT1 | Peroxisomal Testis Enriched Protein 1 |
| PNMA6A | PNMA Family Member 6A |
| PLET1 | Placenta Expressed Transcript 1 |
| SOWAHA | Sosondowah Ankyrin Repeat Domain Family Member A |
| PPP1R3G | Protein Phosphatase 1 Regulatory Subunit 3G |
| TENT4B | Terminal Nucleotidyltransferase 4B |
| TCEANC | Transcription Elongation Factor A N-Terminal And Central Domain Containing |
| SH2D7 | SH2 Domain Containing 7 |
| KIF25 | Kinesin Family Member 25 |
| PRR18 | Proline Rich 18 |
| PRRT4 | Proline Rich Transmembrane Protein 4 |
| CLEC18B | C-Type Lectin Domain Family 18 Member B |
| CLEC18C | C-Type Lectin Domain Family 18 Member C |
| H2AC1 | H2A Clustered Histone 1 |
| H2AC15 | H2A Clustered Histone 15 |
| H2AC16 | H2A Clustered Histone 16 |
| CYSRT1 | Cysteine Rich Tail 1 |
| H2BC5 | H2B Clustered Histone 5 |
| DEXI | Dexi Homolog |
| OR10G7 | Olfactory Receptor Family 10 Subfamily G Member 7 |
| ONECUT3 | One Cut Homeobox 3 |
| CGB2 | Chorionic Gonadotropin Subunit Beta 2 |
| CEP295 | Centrosomal Protein 295 |
| C1orf122 | Chromosome 1 Open Reading Frame 122 |
| NEURL3 | Neuralized E3 Ubiquitin Protein Ligase 3 |
| RPL41 | Ribosomal Protein L41 |
| SAMD5 | Sterile Alpha Motif Domain Containing 5 |
| GFRAL | GDNF Family Receptor Alpha Like |
| METTL24 | Methyltransferase Like 24 |
| ERVMER34-1 | Endogenous Retrovirus Group MER34 Member 1, Envelope |
| FAM183A | Family With Sequence Similarity 183 Member A |
| FAM217A | Family With Sequence Similarity 217 Member A |
| BAHCC1 | BAH Domain And Coiled-Coil Containing 1 |
| FAM120AOS | Family With Sequence Similarity 120A Opposite Strand |
| BRINP2 | BMP/Retinoic Acid Inducible Neural Specific 2 |
| C10orf55 | Chromosome 10 Open Reading Frame 55 (Putative) |
| C12orf45 | Chromosome 12 Open Reading Frame 45 |
| C6orf141 | Chromosome 6 Open Reading Frame 141 |
| LTO1 | LTO1 Maturation Factor Of ABCE1 |
| C9orf131 | Chromosome 9 Open Reading Frame 131 |
| C1orf53 | Chromosome 1 Open Reading Frame 53 |
| FDXACB1 | Ferredoxin-Fold Anticodon Binding Domain Containing 1 |
| ARMCX4 | Armadillo Repeat Containing X-Linked 4 |
| TUSC1 | Tumor Suppressor Candidate 1 |
| ZCWPW2 | Zinc Finger CW-Type And PWWP Domain Containing 2 |
| ZFP41 | ZFP41 Zinc Finger Protein |
| ZC2HC1B | Zinc Finger C2HC-Type Containing 1B |
| TRMO | TRNA Methyltransferase O |
| SMIM5 | Small Integral Membrane Protein 5 |
| PRR14L | Proline Rich 14 Like |
| MILR1 | Mast Cell Immunoglobulin Like Receptor 1 |
| H2AC8 | H2A Clustered Histone 8 |
| MYADML2 | Myeloid Associated Differentiation Marker Like 2 |
| SHLD2 | Shieldin Complex Subunit 2 |
| PLPPR5 | Phospholipid Phosphatase Related 5 |
| HSFX1 | Heat Shock Transcription Factor Family, X-Linked 1 |
| IZUMO1R | IZUMO1 Receptor, JUNO |
| TCP10L2 | T-Complex 10 Like 2 |
| PBOV1 | Prostate And Breast Cancer Overexpressed 1 |
| TMEM170B | Transmembrane Protein 170B |
| TMEM114 | Transmembrane Protein 114 |
| PRSS48 | Serine Protease 48 |
| H2AC17 | H2A Clustered Histone 17 |
| RIPOR1 | RHO Family Interacting Cell Polarization Regulator 1 |
| HIGD1C | HIG1 Hypoxia Inducible Domain Family Member 1C |
| RIMBP3 | RIMS Binding Protein 3 |
| EIPR1 | EARP Complex And GARP Complex Interacting Protein 1 |
| CRYBG1 | Crystallin Beta-Gamma Domain Containing 1 |
| HDGFL1 | HDGF Like 1 |
| H2BC14 | H2B Clustered Histone 14 |
| CGB1 | Chorionic Gonadotropin Subunit Beta 1 |
| CEACAM18 | CEA Cell Adhesion Molecule 18 |
| FAAP20 | FA Core Complex Associated Protein 20 |
| MARF1 | Meiosis Regulator And MRNA Stability Factor 1 |
| FAM229B | Family With Sequence Similarity 229 Member B |
| ATPSCKMT | ATP Synthase C Subunit Lysine N-Methyltransferase |
| FAM200B | Family With Sequence Similarity 200 Member B |
| C12orf71 | Chromosome 12 Open Reading Frame 71 |
| C2orf16 | Chromosome 2 Open Reading Frame 16 |
| ARL17B | ADP Ribosylation Factor Like GTPase 17B |
| ARL5C | ADP Ribosylation Factor Like GTPase 5C |
| ANKRD33B | Ankyrin Repeat Domain 33B |
| ANKRD65 | Ankyrin Repeat Domain 65 |
| FBH1 | F-Box DNA Helicase 1 |
| LRRC70 | Leucine Rich Repeat Containing 70 |
| LRRC73 | Leucine Rich Repeat Containing 73 |
| KRTAP4-11 | Keratin Associated Protein 4-11 |
| KRTAP4-8 | Keratin Associated Protein 4-8 |
| VIRMA | Vir Like M6A Methyltransferase Associated |
| XAGE2 | X Antigen Family Member 2 |
| TMEM236 | Transmembrane Protein 236 |
| TTC24 | Tetratricopeptide Repeat Domain 24 |
| ZNF891 | Zinc Finger Protein 891 |
| ZNF737 | Zinc Finger Protein 737 |
| ZUP1 | Zinc Finger Containing Ubiquitin Peptidase 1 |
| ZSCAN23 | Zinc Finger And SCAN Domain Containing 23 |
| ZNF587B | Zinc Finger Protein 587B |
| EFCAB9 | EF-Hand Calcium Binding Domain 9 |
| NOTCH2NLA | Notch 2 N-Terminal Like A |
| PTGES3L | Prostaglandin E Synthase 3 Like |
| PTGES3L-AARSD1 | PTGES3L-AARSD1 Readthrough |
| TGIF2LY | TGFB Induced Factor Homeobox 2 Like Y-Linked |
| SLC66A2 | Solute Carrier Family 66 Member 2 |
| POTEI | POTE Ankyrin Domain Family Member I |
| SOWAHC | Sosondowah Ankyrin Repeat Domain Family Member C |
| SPANXA2 | SPANX Family Member A2 |
| SPATA1 | Spermatogenesis Associated 1 |
| PCNX3 | Pecanex 3 |
| TMEM131L | Transmembrane 131 Like |
| STEAP1B | STEAP Family Member 1B |
| STMND1 | Stathmin Domain Containing 1 |
| PRR35 | Proline Rich 35 |
| MIR22HG | MIR22 Host Gene |
| NEMP1 | Nuclear Envelope Integral Membrane Protein 1 |
| DEUP1 | Deuterosome Assembly Protein 1 |
| DIPK2A | Divergent Protein Kinase Domain 2A |
| DISP3 | Dispatched RND Transporter Family Member 3 |
| CFAP46 | Cilia And Flagella Associated Protein 46 |
| RNASE12 | Ribonuclease A Family Member 12 (Inactive) |
| SAPCD1 | Suppressor APC Domain Containing 1 |
| MFSD14B | Major Facilitator Superfamily Domain Containing 14B |
| CCDC169 | Coiled-Coil Domain Containing 169 |
| C16orf95 | Chromosome 16 Open Reading Frame 95 |
| C6orf132 | Chromosome 6 Open Reading Frame 132 |
| BORCS5 | BLOC-1 Related Complex Subunit 5 |
| MAP3K21 | Mitogen-Activated Protein Kinase Kinase Kinase 21 |
| TRAPPC3L | Trafficking Protein Particle Complex 3 Like |
| KRTAP2-2 | Keratin Associated Protein 2-2 |
| LEUTX | Leucine Twenty Homeobox |
| KRTAP16-1 | Keratin Associated Protein 16-1 |
| PGAP6 | Post-Glycosylphosphatidylinositol Attachment To Proteins 6 |
| SLF2 | SMC5-SMC6 Complex Localization Factor 2 |
| ZSCAN5B | Zinc Finger And SCAN Domain Containing 5B |
| KHDC4 | KH Domain Containing 4, Pre-MRNA Splicing Factor |
| PLAAT1 | Phospholipase A And Acyltransferase 1 |
| TMEM178B | Transmembrane Protein 178B |
| CYRIA | CYFIP Related Rac1 Interactor A |
| CSNK2A3 | Casein Kinase 2 Alpha 3 |
| DENND11 | DENN Domain Containing 11 |
| DIPK2B | Divergent Protein Kinase Domain 2B |
| MICOS10 | Mitochondrial Contact Site And Cristae Organizing System Subunit 10 |
| MINDY3 | MINDY Lysine 48 Deubiquitinase 3 |
| CCDC159 | Coiled-Coil Domain Containing 159 |
| BMERB1 | BMERB Domain Containing 1 |
| C6orf52 | Chromosome 6 Open Reading Frame 52 |
| C3orf80 | Chromosome 3 Open Reading Frame 80 |
| FER1L5 | Fer-1 Like Family Member 5 |
| EOLA1 | Endothelium And Lymphocyte Associated ASCH Domain 1 |
| KRBOX1 | KRAB Box Domain Containing 1 |
| ZNF726 | Zinc Finger Protein 726 |
| JPT2 | Jupiter Microtubule Associated Homolog 2 |
| NSUN5P2 | NSUN5 Pseudogene 2 |
| POTEJ | POTE Ankyrin Domain Family Member J |
| RAB5IF | RAB5 Interacting Factor |
| INAFM1 | InaF Motif Containing 1 |
| TMED7-TICAM2 | TMED7-TICAM2 Readthrough |
| CLPSL1 | Colipase Like 1 |
| GRAMD2B | GRAM Domain Containing 2B |
| NBR2 | Neighbor Of BRCA1 LncRNA 2 |
| RTL6 | Retrotransposon Gag Like 6 |
| CASTOR1 | Cytosolic Arginine Sensor For MTORC1 Subunit 1 |
| MCRIP2 | MAPK Regulated Corepressor Interacting Protein 2 |
| FAM228B | Family With Sequence Similarity 228 Member B |
| FAM25A | Family With Sequence Similarity 25 Member A |
| C11orf94 | Chromosome 11 Open Reading Frame 94 |
| C6orf99 | Chromosome 6 Open Reading Frame 99 |
| C20orf173 | Chromosome 20 Open Reading Frame 173 |
| ANHX | Anomalous Homeobox |
| ANKUB1 | Ankyrin Repeat And Ubiquitin Domain Containing 1 |
| KIAA0087 | KIAA0087 LncRNA |
| KRTAP2-3 | Keratin Associated Protein 2-3 |
| KRTAP4-7 | Keratin Associated Protein 4-7 |
| LINC02693 | Long Intergenic Non-Protein Coding RNA 2693 |
| SMLR1 | Small Leucine Rich Protein 1 |
| SHISAL1 | Shisa Like 1 |
| KHDC1L | KH Domain Containing 1 Like |
| PRAMEF18 | PRAME Family Member 18 |
| PMF1-BGLAP | PMF1-BGLAP Readthrough |
| SNHG12 | Small Nucleolar RNA Host Gene 12 |
| PATE3 | Prostate And Testis Expressed 3 |
| RETREG2 | Reticulophagy Regulator Family Member 2 |
| EEF1AKMT3 | EEF1A Lysine Methyltransferase 3 |
| H4C7 | H4 Clustered Histone 7 |
| DCAF8L2 | DDB1 And CUL4 Associated Factor 8 Like 2 |
| SERHL | Serine Hydrolase Like (Pseudogene) |
| CFAP206 | Cilia And Flagella Associated Protein 206 |
| CEP295NL | CEP295 N-Terminal Like |
| MTRES1 | Mitochondrial Transcription Rescue Factor 1 |
| MUC22 | Mucin 22 |
| OPRPN | Opiorphin Prepropeptide |
| BICRAL | BRD4 Interacting Chromatin Remodeling Complex Associated Protein Like |
| ARMH3 | Armadillo Like Helical Domain Containing 3 |
| C17orf100 | Chromosome 17 Open Reading Frame 100 |
| C11orf91 | Chromosome 11 Open Reading Frame 91 |
| BORCS8-MEF2B | BORCS8-MEF2B Readthrough |
| CALHM5 | Calcium Homeostasis Modulator Family Member 5 |
| ARPC4-TTLL3 | ARPC4-TTLL3 Readthrough |
| LINC00472 | Long Intergenic Non-Protein Coding RNA 472 |
| STPG4 | Sperm-Tail PG-Rich Repeat Containing 4 |
| SNHG11 | Small Nucleolar RNA Host Gene 11 |
| SMIM9 | Small Integral Membrane Protein 9 |
| DNAJC25-GNG10 | DNAJC25-GNG10 Readthrough |
| MEI4 | Meiotic Double-Stranded Break Formation Protein 4 |
| C16orf97 | Chromosome 16 Open Reading Frame 97 |
| TMEM244 | Transmembrane Protein 244 |
| LINC01600 | Long Intergenic Non-Protein Coding RNA 1600 |
| JPX | JPX Transcript, XIST Activator |
| TMEM35B | Transmembrane Protein 35B |
| MIR34B | MicroRNA 34b |
| CYP2D7 | Cytochrome P450 Family 2 Subfamily D Member 7 (Gene/Pseudogene) |
| MKRN2OS | MKRN2 Opposite Strand |
| DELEC1 | Deleted In Esophageal Cancer 1 |
| CENPS-CORT | CENPS-CORT Readthrough |
| OCLM | Oculomedin |
| GGNBP1 | Gametogenetin Binding Protein 1 (Pseudogene) |
| ALKAL2 | ALK And LTK Ligand 2 |
| BNIP5 | BCL2 Interacting Protein 5 |
| C10orf126 | Chromosome 10 Open Reading Frame 126 |
| ANKRD26P1 | Ankyrin Repeat Domain 26 Pseudogene 1 |
| ANKRD30BL | Ankyrin Repeat Domain 30B Like |
| UQCRHL | Ubiquinol-Cytochrome C Reductase Hinge Protein Like |
| USP17L5 | Ubiquitin Specific Peptidase 17 Like Family Member 5 |
| SMIM6 | Small Integral Membrane Protein 6 |
| TEX47 | Testis Expressed 47 |
| PRAMEF19 | PRAME Family Member 19 |
| SPDYE6 | Speedy/RINGO Cell Cycle Regulator Family Member E6 |
| GGTA1P | Glycoprotein Alpha-Galactosyltransferase 1, Pseudogene |
| GJE1 | Gap Junction Protein Epsilon 1 |
| ADARB2-AS1 | ADARB2 Antisense RNA 1 |
| CALHM4 | Calcium Homeostasis Modulator Family Member 4 |
| ARMH1 | Armadillo Like Helical Domain Containing 1 |
| ANKRD66 | Ankyrin Repeat Domain 66 |
| LINC00242 | Long Intergenic Non-Protein Coding RNA 242 |
| LINC00574 | Long Intergenic Non-Protein Coding RNA 574 |
| LINC00299 | Long Intergenic Non-Protein Coding RNA 299 |
| LINC00312 | Long Intergenic Non-Protein Coding RNA 312 |
| LINC01006 | Long Intergenic Non-Protein Coding RNA 1006 |
| LINC01588 | Long Intergenic Non-Protein Coding RNA 1588 |
| MIR31HG | MIR31 Host Gene |
| MIRLET7BHG | MIRLET7B Host Gene |
| MIR9-3 | MicroRNA 9-3 |
| HNF1A-AS1 | HNF1A Antisense RNA 1 |
| MIR431 | MicroRNA 431 |
| MIR589 | MicroRNA 589 |
| HOXB-AS3 | HOXB Cluster Antisense RNA 3 |
| HOTAIRM1 | HOXA Transcript Antisense RNA, Myeloid-Specific 1 |
| FAM47E-STBD1 | FAM47E-STBD1 Readthrough |
| B3GALT5-AS1 | B3GALT5 Antisense RNA 1 |
| LRRC53 | Leucine Rich Repeat Containing 53 |
| BPIFA4P | BPI Fold Containing Family A Member 4, Pseudogene |
| ARPIN-AP3S2 | ARPIN-AP3S2 Readthrough |
| TRAPPC2B | Trafficking Protein Particle Complex 2B |
| ZNF137P | Zinc Finger Protein 137, Pseudogene |
| USP17L24 | Ubiquitin Specific Peptidase 17 Like Family Member 24 |
| USP17L25 | Ubiquitin Specific Peptidase 17 Like Family Member 25 |
| USP17L29 | Ubiquitin Specific Peptidase 17 Like Family Member 29 |
| TSNAX-DISC1 | TSNAX-DISC1 Readthrough (NMD Candidate) |
| LINC01558 | Long Intergenic Non-Protein Coding RNA 1558 |
| RBAK-RBAKDN | RBAK-RBAKDN Readthrough |
| SLC7A5P2 | Solute Carrier Family 7 Member 5 Pseudogene 2 |
| SNHG3 | Small Nucleolar RNA Host Gene 3 |
| IQCA1L | IQ Motif Containing With AAA Domain 1 Like |
| MIR30C2 | MicroRNA 30c-2 |
| MIR24-2 | MicroRNA 24-2 |
| MIR340 | MicroRNA 340 |
| NAPSB | Napsin B Aspartic Peptidase, Pseudogene |
| OR2J1 | Olfactory Receptor Family 2 Subfamily J Member 1 (Gene/Pseudogene) |
| MIR1224 | MicroRNA 1224 |
| MIR124-2 | MicroRNA 124-2 |
| BVES-AS1 | BVES Antisense RNA 1 |
| C8orf17 | Chromosome 8 Open Reading Frame 17 |
| ANKRD19P | Ankyrin Repeat Domain 19, Pseudogene |
| UMAD1 | UBAP1-MVB12-Associated (UMA) Domain Containing 1 |
| USP17L20 | Ubiquitin Specific Peptidase 17 Like Family Member 20 |
| USP17L22 | Ubiquitin Specific Peptidase 17 Like Family Member 22 |
| USP17L26 | Ubiquitin Specific Peptidase 17 Like Family Member 26 |
| USP17L27 | Ubiquitin Specific Peptidase 17 Like Family Member 27 |
| USP17L13 | Ubiquitin Specific Peptidase 17 Like Family Member 13 |
| USP17L30 | Ubiquitin Specific Peptidase 17 Like Family Member 30 |
| USP17L17 | Ubiquitin Specific Peptidase 17 Like Family Member 17 |
| USP17L18 | Ubiquitin Specific Peptidase 17 Like Family Member 18 |
| TSTD3 | Thiosulfate Sulfurtransferase Like Domain Containing 3 |
| LINC01590 | Long Intergenic Non-Protein Coding RNA 1590 |
| SYS1-DBNDD2 | SYS1-DBNDD2 Readthrough (NMD Candidate) |
| HLA-J | Major Histocompatibility Complex, Class I, J (Pseudogene) |
| SNORD118 | Small Nucleolar RNA, C/D Box 118 |
| SNORA73B | Small Nucleolar RNA, H/ACA Box 73B |
| TDGF1P3 | Teratocarcinoma-Derived Growth Factor 1 Pseudogene 3 |
| SLC22A20P | Solute Carrier Family 22 Member 20, Pseudogene |
| MIR365A | MicroRNA 365a |
| MIR181D | MicroRNA 181d |
| MIR187 | MicroRNA 187 |
| GRIFIN | Galectin-Related Inter-Fiber Protein |
| MIR519B | MicroRNA 519b |
| RTEL1-TNFRSF6B | RTEL1-TNFRSF6B Readthrough (NMD Candidate) |
| MIR133A1 | MicroRNA 133a-1 |
| MIR124-3 | MicroRNA 124-3 |
| BCAR4 | Breast Cancer Anti-Estrogen Resistance 4 |
| EPB41L4A-AS1 | EPB41L4A Antisense RNA 1 |
| LINC00269 | Long Intergenic Non-Protein Coding RNA 269 |
| USP17L19 | Ubiquitin Specific Peptidase 17 Like Family Member 19 |
| USP17L10 | Ubiquitin Specific Peptidase 17 Like Family Member 10 |
| USP17L11 | Ubiquitin Specific Peptidase 17 Like Family Member 11 |
| USP17L12 | Ubiquitin Specific Peptidase 17 Like Family Member 12 |
| USP17L28 | Ubiquitin Specific Peptidase 17 Like Family Member 28 |
| USP17L15 | Ubiquitin Specific Peptidase 17 Like Family Member 15 |
| LINC00452 | Long Intergenic Non-Protein Coding RNA 452 |
| TRPC5OS | TRPC5 Opposite Strand |
| LBX2-AS1 | LBX2 Antisense RNA 1 |
| LINC01548 | Long Intergenic Non-Protein Coding RNA 1548 |
| LINC01556 | Long Intergenic Non-Protein Coding RNA 1556 |
| EGFEM1P | EGF Like And EMI Domain Containing 1, Pseudogene |
| NR2F1-AS1 | NR2F1 Antisense RNA 1 |
| SERPINA13P | Serpin Family A Member 13, Pseudogene |
| TGIF2-RAB5IF | TGIF2-RAB5IF Readthrough |
| SNORD17 | Small Nucleolar RNA, C/D Box 17 |
| SNORD20 | Small Nucleolar RNA, C/D Box 20 |
| SNORA73A | Small Nucleolar RNA, H/ACA Box 73A |
| SNORD83A | Small Nucleolar RNA, C/D Box 83A |
| SNORD83B | Small Nucleolar RNA, C/D Box 83B |
| SNORA52 | Small Nucleolar RNA, H/ACA Box 52 |
| HSP90AB4P | Heat Shock Protein 90 Alpha Family Class B Member 4, Pseudogene |
| PPP1R2P1 | Protein Phosphatase 1 Regulatory Inhibitor Subunit 2 Pseudogene 1 |
| TMEM191A | Transmembrane Protein 191A (Pseudogene) |
| PRORSD1P | Prolyl-TRNA Synthetase Associated Domain Containing 1, Pseudogene |
| SSR4P1 | Signal Sequence Receptor Subunit 4 Pseudogene 1 |
| CLRN1-AS1 | CLRN1 Antisense RNA 1 |
| MIR147B | MicroRNA 147b |
| MIR19B2 | MicroRNA 19b-2 |
| MIR885 | MicroRNA 885 |
| MIR616 | MicroRNA 616 |
| RNU12 | RNA, U12 Small Nuclear |
| MIR520D | MicroRNA 520d |
| MIR490 | MicroRNA 490 |
| MIR495 | MicroRNA 495 |
| MIR4435-2HG | MIR4435-2 Host Gene |
| HNRNPUL2-BSCL2 | HNRNPUL2-BSCL2 Readthrough (NMD Candidate) |
| HOXA10-AS | HOXA10 Antisense RNA |
| OR2B8P | Olfactory Receptor Family 2 Subfamily B Member 8 Pseudogene |
| MTRNR2L9 | MT-RNR2 Like 9 |
| MIR501 | MicroRNA 501 |
| NKX2-1-AS1 | NKX2-1 Antisense RNA 1 |
| HNRNPCL2 | Heterogeneous Nuclear Ribonucleoprotein C Like 2 |
| AFDN-DT | AFDN Divergent Transcript |
| C11orf72 | Chromosome 11 Putative Open Reading Frame 72 |
| FMC1-LUC7L2 | FMC1-LUC7L2 Readthrough |
| ZFP91-CNTF | ZFP91-CNTF Readthrough (NMD Candidate) |
| USP17L21 | Ubiquitin Specific Peptidase 17 Like Family Member 21 |
| LINC00029 | Long Intergenic Non-Protein Coding RNA 29 |
| LINC00310 | Long Intergenic Non-Protein Coding RNA 310 |
| TRIM75P | Tripartite Motif Containing 75, Pseudogene |
| LINC01549 | Long Intergenic Non-Protein Coding RNA 1549 |
| LINC-PINT | Long Intergenic Non-Protein Coding RNA, P53 Induced Transcript |
| NTM-AS1 | NTM Antisense RNA 1 |
| SNORA13 | Small Nucleolar RNA, H/ACA Box 13 |
| SNORA23 | Small Nucleolar RNA, H/ACA Box 23 |
| SNORD12B | Small Nucleolar RNA, C/D Box 12B |
| SNORD13 | Small Nucleolar RNA, C/D Box 13 |
| SNORD14C | Small Nucleolar RNA, C/D Box 14C |
| SNORD15B | Small Nucleolar RNA, C/D Box 15B |
| SNORD3A | Small Nucleolar RNA, C/D Box 3A |
| SNORD3B-1 | Small Nucleolar RNA, C/D Box 3B-1 |
| SNORD61 | Small Nucleolar RNA, C/D Box 61 |
| SNORA53 | Small Nucleolar RNA, H/ACA Box 53 |
| TMEM265 | Transmembrane Protein 265 |
| STX16-NPEPL1 | STX16-NPEPL1 Readthrough (NMD Candidate) |
| CLUHP3 | Clustered Mitochondria Homolog Pseudogene 3 |
| MIR1908 | MicroRNA 1908 |
| MKRN4P | Makorin Ring Finger Protein 4, Pseudogene |
| MIR618 | MicroRNA 618 |
| MIR663B | MicroRNA 663b |
| MIR526A1 | MicroRNA 526a-1 |
| HCG14 | HLA Complex Group 14 |
| RN7SL2 | RNA Component Of Signal Recognition Particle 7SL2 |
| MROH7-TTC4 | MROH7-TTC4 Readthrough (NMD Candidate) |
| MIR105-2 | MicroRNA 105-2 |
| RPL13AP20 | Ribosomal Protein L13a Pseudogene 20 |
| GACAT2 | Gastric Cancer Associated Transcript 2 |
| CCDC28A-AS1 | CCDC28A Antisense RNA 1 |
| AGPAT4-IT1 | AGPAT4 Intronic Transcript 1 |
| LOC155060 | AI894139 Pseudogene |
| FAM66A | Family With Sequence Similarity 66 Member A |
| FAM226B | Family With Sequence Similarity 226 Member B |
| LOC339803 | Uncharacterized LOC339803 |
| C1GALT1C1L | C1GALT1 Specific Chaperone 1 Like |
| FGF10-AS1 | FGF10 Antisense RNA 1 |
| CASC8 | Cancer Susceptibility 8 |
| TIPARP-AS1 | TIPARP Antisense RNA 1 |
| TPI1P2 | Triosephosphate Isomerase 1 Pseudogene 2 |
| LINC00239 | Long Intergenic Non-Protein Coding RNA 239 |
| LINC00545 | Long Intergenic Non-Protein Coding RNA 545 |
| UBE2F-SCLY | UBE2F-SCLY Readthrough (NMD Candidate) |
| LINC02210 | Long Intergenic Non-Protein Coding RNA 2210 |
| TTTY14 | Testis-Specific Transcript, Y-Linked 14 |
| LCN1P1 | Lipocalin 1 Pseudogene 1 |
| ZNF663P | Zinc Finger Protein 663, Pseudogene |
| LINC01387 | Long Intergenic Non-Protein Coding RNA 1387 |
| ZNF300P1 | Zinc Finger Protein 300 Pseudogene 1 |
| PTPRVP | Protein Tyrosine Phosphatase Receptor Type V, Pseudogene |
| SNORD15A | Small Nucleolar RNA, C/D Box 15A |
| SNORD16 | Small Nucleolar RNA, C/D Box 16 |
| SNORD3B-2 | Small Nucleolar RNA, C/D Box 3B-2 |
| SNORD3C | Small Nucleolar RNA, C/D Box 3C |
| PISRT1 | PISRT1 LncRNA |
| MIR216B | MicroRNA 216b |
| MIR362 | MicroRNA 362 |
| RNU4-1 | RNA, U4 Small Nuclear 1 |
| MIR4500HG | MIR4500 Host Gene |
| DISC1FP1 | DISC1 Fusion Partner 1 |
| SCGB1C2 | Secretoglobin Family 1C Member 2 |
| MGC16025 | Uncharacterized LOC85009 |
| ADAM3A | ADAM Metallopeptidase Domain 3A (Pseudogene) |
| LRRC37BP1 | Leucine Rich Repeat Containing 37B Pseudogene 1 |
| ARMH2 | Armadillo Like Helical Domain Containing 2 |
| MBNL1-AS1 | MBNL1 Antisense RNA 1 |
| USP17L6P | Ubiquitin Specific Peptidase 17 Like Family Member 6, Pseudogene |
| TMEM238L | Transmembrane Protein 238 Like |
| LINC01433 | Long Intergenic Non-Protein Coding RNA 1433 |
| LINC00941 | Long Intergenic Non-Protein Coding RNA 941 |
| LINC00977 | Long Intergenic Non-Protein Coding RNA 977 |
| LINC00598 | Long Intergenic Non-Protein Coding RNA 598 |
| LINC01104 | Long Intergenic Non-Protein Coding RNA 1104 |
| MIR758 | MicroRNA 758 |
| HLA-L | Major Histocompatibility Complex, Class I, L (Pseudogene) |
| IL21R-AS1 | IL21R Antisense RNA 1 |
| PXN-AS1 | PXN Antisense RNA 1 |
| INTS6-AS1 | INTS6 Antisense RNA 1 |
| SNORD18B | Small Nucleolar RNA, C/D Box 18B |
| SNORD18C | Small Nucleolar RNA, C/D Box 18C |
| SNORD25 | Small Nucleolar RNA, C/D Box 25 |
| SNORD26 | Small Nucleolar RNA, C/D Box 26 |
| SNORD35B | Small Nucleolar RNA, C/D Box 35B |
| SNORA80E | Small Nucleolar RNA, H/ACA Box 80E |
| SNORD10 | Small Nucleolar RNA, C/D Box 10 |
| SNORD101 | Small Nucleolar RNA, C/D Box 101 |
| SNORD104 | Small Nucleolar RNA, C/D Box 104 |
| SNORA3B | Small Nucleolar RNA, H/ACA Box 3B |
| SOX9-AS1 | SOX9 Antisense RNA 1 |
| SLC25A5-AS1 | SLC25A5 Antisense RNA 1 |
| IQCM | IQ Motif Containing M |
| GTF3C2-AS1 | GTF3C2 Antisense RNA 1 |
| HM13-AS1 | HM13 Antisense RNA 1 |
| RNU4-2 | RNA, U4 Small Nuclear 2 |
| RNF5P1 | Ring Finger Protein 5 Pseudogene 1 |
| RNU1-4 | RNA, U1 Small Nuclear 4 |
| MIR526A2 | MicroRNA 526a-2 |
| MIR548C | MicroRNA 548c |
| MIR487A | MicroRNA 487a |
| MIR376B | MicroRNA 376b |
| MSTO2P | Misato Family Member 2, Pseudogene |
| HPN-AS1 | HPN Antisense RNA 1 |
| MRPL23-AS1 | MRPL23 Antisense RNA 1 |
| OR12D1 | Olfactory Receptor Family 12 Subfamily D Member 1 (Gene/Pseudogene) |
| SDHAP2 | Succinate Dehydrogenase Complex Flavoprotein Subunit A Pseudogene 2 |
| MIR1299 | MicroRNA 1299 |
| MIR105-1 | MicroRNA 105-1 |
| LOC100130691 | Uncharacterized LOC100130691 |
| OR1E3 | Olfactory Receptor Family 1 Subfamily E Member 3 (Gene/Pseudogene) |
| SAP30L-AS1 | SAP30L Antisense RNA 1 (Head To Head) |
| DLGAP4-AS1 | DLGAP4 Antisense RNA 1 |
| GCC2-AS1 | GCC2 Antisense RNA 1 |
| CCNYL3 | Cyclin Y Like 3 (Pseudogene) |
| MCF2L-AS1 | MCF2L Antisense RNA 1 |
| B4GALT1-AS1 | B4GALT1 Antisense RNA 1 |
| BRD3OS | BRD3 Opposite Strand |
| CCDC144NL-AS1 | CCDC144NL Antisense RNA 1 |
| TPTEP2-CSNK1E | TPTEP2-CSNK1E Readthrough |
| LINC00583 | Long Intergenic Non-Protein Coding RNA 583 |
| LINC00587 | Long Intergenic Non-Protein Coding RNA 587 |
| VN1R10P | Vomeronasal 1 Receptor 10 Pseudogene |
| LINC00290 | Long Intergenic Non-Protein Coding RNA 290 |
| LINC02210-CRHR1 | LINC02210-CRHR1 Readthrough |
| WWTR1-AS1 | WWTR1 Antisense RNA 1 |
| SLIT2-IT1 | SLIT2 Intronic Transcript 1 |
| LINC01090 | Long Intergenic Non-Protein Coding RNA 1090 |
| LINC01014 | Long Intergenic Non-Protein Coding RNA 1014 |
| DUSP5P1 | Dual Specificity Phosphatase 5 Pseudogene 1 |
| RNU5F-1 | RNA, U5F Small Nuclear 1 |
| REELD1 | Reeler Domain Containing 1 |
| IFITM4P | Interferon Induced Transmembrane Protein 4 Pseudogene |
| SRP9P1 | Signal Recognition Particle 9 Pseudogene 1 |
| PRC1-AS1 | PRC1 Antisense RNA 1 |
| PPP3CB-AS1 | PPP3CB Antisense RNA 1 (Head To Head) |
| SNORD14E | Small Nucleolar RNA, C/D Box 14E |
| SNHG19 | Small Nucleolar RNA Host Gene 19 |
| PKN2-AS1 | PKN2 Antisense RNA 1 |
| SERPINB9P1 | Serpin Family B Member 9 Pseudogene 1 |
| PPP1R26-AS1 | PPP1R26 Antisense RNA 1 |
| P2RX5-TAX1BP3 | P2RX5-TAX1BP3 Readthrough (NMD Candidate) |
| CCDC192 | Coiled-Coil Domain Containing 192 |
| FLNB-AS1 | FLNB Antisense RNA 1 |
| MIR3130-2 | MicroRNA 3130-2 |
| MKNK1-AS1 | MKNK1 Antisense RNA 1 |
| MIR663AHG | MIR663A Host Gene |
| RNU1-2 | RNA, U1 Small Nuclear 2 |
| RNU1-3 | RNA, U1 Small Nuclear 3 |
| CRTC3-AS1 | CRTC3 Antisense RNA 1 |
| MIR548K | MicroRNA 548k |
| MIR4449 | MicroRNA 4449 |
| SDHAP1 | Succinate Dehydrogenase Complex Flavoprotein Subunit A Pseudogene 1 |
| SDIM1 | Stress Responsive DNAJB4 Interacting Membrane Protein 1 |
| SENP3-EIF4A1 | SENP3-EIF4A1 Readthrough (NMD Candidate) |
| GOLGA2P7 | GOLGA2 Pseudogene 7 |
| MIA-RAB4B | MIA-RAB4B Readthrough (NMD Candidate) |
| MIR1246 | MicroRNA 1246 |
| MTUS2-AS1 | MTUS2 Antisense RNA 1 |
| RNA5S1 | RNA, 5S Ribosomal 1 |
| RNA5S10 | RNA, 5S Ribosomal 10 |
| RNA5S11 | RNA, 5S Ribosomal 11 |
| RNA5S12 | RNA, 5S Ribosomal 12 |
| RNA5S13 | RNA, 5S Ribosomal 13 |
| RNA5S15 | RNA, 5S Ribosomal 15 |
| RNA5S16 | RNA, 5S Ribosomal 16 |
| RNA5S17 | RNA, 5S Ribosomal 17 |
| RNA5S2 | RNA, 5S Ribosomal 2 |
| RNA5S3 | RNA, 5S Ribosomal 3 |
| RNA5S4 | RNA, 5S Ribosomal 4 |
| RNA5S5 | RNA, 5S Ribosomal 5 |
| RNA5S6 | RNA, 5S Ribosomal 6 |
| RNA5S7 | RNA, 5S Ribosomal 7 |
| RNA5S8 | RNA, 5S Ribosomal 8 |
| RNA5S9 | RNA, 5S Ribosomal 9 |
| DNM1P35 | Dynamin 1 Pseudogene 35 |
| RNVU1-18 | RNA, Variant U1 Small Nuclear 18 |
| LOC100287896 | Uncharacterized LOC100287896 |
| LOC100505549 | Uncharacterized LOC100505549 |
| RNU6-8 | RNA, U6 Small Nuclear 8 |
| RNU7-1 | RNA, U7 Small Nuclear 1 |
| CD99P1 | CD99 Molecule Pseudogene 1 |
| CCDC183-AS1 | CCDC183 Antisense RNA 1 |
| ADPGK-AS1 | ADPGK Antisense RNA 1 |
| ADAM6 | ADAM Metallopeptidase Domain 6 (Pseudogene) |
| AGAP2-AS1 | AGAP2 Antisense RNA 1 |
| FAM230I | Family With Sequence Similarity 230 Member I |
| FAM237A | Family With Sequence Similarity 237 Member A |
| LOC730098 | Uncharacterized LOC730098 |
| CARD8-AS1 | CARD8 Antisense RNA 1 |
| MAFTRR | MAF Transcriptional Regulator RNA |
| FLJ31356 | Uncharacterized Protein FLJ31356 |
| RCAN3AS | RCAN3 Antisense RNA |
| UNC5B-AS1 | UNC5B Antisense RNA 1 |
| VTRNA1-1 | Vault RNA 1-1 |
| UNQ6494 | Uncharacterized LOC100129066 |
| LINC00410 | Long Intergenic Non-Protein Coding RNA 410 |
| LINC00327 | Long Intergenic Non-Protein Coding RNA 327 |
| SLC8A1-AS1 | SLC8A1 Antisense RNA 1 |
| SMG1P3 | SMG1 Pseudogene 3 |
| ZNF840P | Zinc Finger Protein 840, Pseudogene |
| LINC00865 | Long Intergenic Non-Protein Coding RNA 865 |
| LINC00899 | Long Intergenic Non-Protein Coding RNA 899 |
| ZNF37BP | Zinc Finger Protein 37B, Pseudogene |
| SLCO4A1-AS1 | SLCO4A1 Antisense RNA 1 |
| LINC02869 | Long Intergenic Non-Protein Coding RNA 2869 |
| MIR3130-1 | MicroRNA 3130-1 |
| NOS2P1 | Nitric Oxide Synthase 2 Pseudogene 1 |
| SH3TC2-DT | SH3TC2 Divergent Transcript |
| PRG1 | P53-Responsive Gene 1 |
| PCDHA@ | Protocadherin Alpha Cluster, Complex Locus |
| ENSG00000241489 |  |
| PIN4P1 | Peptidylprolyl Cis/Trans Isomerase, NIMA-Interacting 4 Pseudogene 1 |
| ENSG00000244255 |  |
| RALY-AS1 | RALY Antisense RNA 1 |
| SSU72P8 | SSU72 Pseudogene 8 |
| CCDC200 | Coiled-Coil Domain Containing 200 |
| EEF1A1P6 | Eukaryotic Translation Elongation Factor 1 Alpha 1 Pseudogene 6 |
| MIR568 | MicroRNA 568 |
| DEFA7P | Defensin Alpha 7, Pseudogene |
| DEFA8P | Defensin Alpha 8, Pseudogene |
| DHFRP2 | Dihydrofolate Reductase Pseudogene 2 |
| SEPT5-GP1BB | SEPT5-GP1BB Readthrough |
| MIR1245A | MicroRNA 1245a |
| MYMX | Myomixer, Myoblast Fusion Factor |
| MIR7706 | MicroRNA 7706 |
| RNVU1-7 | RNA, Variant U1 Small Nuclear 7 |
| RN7SL3 | RNA Component Of Signal Recognition Particle 7SL3 |
| RPL37AP8 | Ribosomal Protein L37a Pseudogene 8 |
| OR2A1-AS1 | OR2A1 Antisense RNA 1 |
| MCPH1-AS1 | MCPH1 Antisense RNA 1 |
| LOC284865 | Uncharacterized LOC284865 |
| EXOC1L | Exocyst Complex Component 1 Like |
| LOC441204 | Uncharacterized LOC441204 |
| LOC442028 | Uncharacterized LOC442028 |
| FLJ37453 | Uncharacterized LOC729614 |
| ARAP1-AS2 | ARAP1 Antisense RNA 2 |
| LINC00294 | Long Intergenic Non-Protein Coding RNA 294 |
| UBE2Q2P2 | Ubiquitin Conjugating Enzyme E2 Q2 Pseudogene 2 |
| LANCL1-AS1 | LANCL1 Antisense RNA 1 |
| SMG1P5 | SMG1 Pseudogene 5 |
| ZNF890P | Zinc Finger Protein 890, Pseudogene |
| ZNF723 | Zinc Finger Protein 723 |
| LINC01346 | Long Intergenic Non-Protein Coding RNA 1346 |
| LINC01475 | Long Intergenic Non-Protein Coding RNA 1475 |
| LINC00907 | Long Intergenic Non-Protein Coding RNA 907 |
| LINC01518 | Long Intergenic Non-Protein Coding RNA 1518 |
| LINC01625 | Long Intergenic Non-Protein Coding RNA 1625 |
| PGAM1P5 | Phosphoglycerate Mutase 1 Pseudogene 5 |
| CERS6-AS1 | CERS6 Antisense RNA 1 |
| LINC02874 | Long Intergenic Non-Protein Coding RNA 2874 |
| RPS25P6 | Ribosomal Protein S25 Pseudogene 6 |
| PPBPP1 | Pro-Platelet Basic Protein Pseudogene 1 |
| LY6E-DT | LY6E Divergent Transcript |
| MIR3648-1 | MicroRNA 3648-1 |
| HNRNPA1P33 | Heterogeneous Nuclear Ribonucleoprotein A1 Pseudogene 33 |
| EEF1DP1 | Eukaryotic Translation Elongation Factor 1 Delta Pseudogene 1 |
| MIR5571 | MicroRNA 5571 |
| MIR570HG | MIR570 Host Gene |
| MMADHC-DT | MMADHC Divergent Transcript |
| RNVU1-6 | RNA, Variant U1 Small Nuclear 6 |
| LOC100289580 | Uncharacterized LOC100289580 |
| FAM86B3P | Family With Sequence Similarity 86 Member B3, Pseudogene |
| LOC286359 | Uncharacterized LOC286359 |
| LOC339862 | Uncharacterized LOC339862 |
| FAM111A-DT | FAM111A Divergent Transcript |
| FLJ22447 | Uncharacterized LOC400221 |
| FLJ40194 | Uncharacterized FLJ40194 |
| KRT18P42 | Keratin 18 Pseudogene 42 |
| KRTAP4-16 | Keratin Associated Protein 4-16 |
| LINC01704 | Long Intergenic Non-Protein Coding RNA 1704 |
| LINC01963 | Long Intergenic Non-Protein Coding RNA 1963 |
| LINC00492 | Long Intergenic Non-Protein Coding RNA 492 |
| LINC00513 | Long Intergenic Non-Protein Coding RNA 513 |
| LINC01144 | Long Intergenic Non-Protein Coding RNA 1144 |
| IGLV2-5 | Immunoglobulin Lambda Variable 2-5 (Pseudogene) |
| TBC1D3P1-DHX40P1 | TBC1D3P1-DHX40P1 Readthrough, Transcribed Pseudogene |
| LINC02871 | Long Intergenic Non-Protein Coding RNA 2871 |
| LINC02347 | Long Intergenic Non-Protein Coding RNA 2347 |
| MIR7702 | MicroRNA 7702 |
| NOS2P2 | Nitric Oxide Synthase 2 Pseudogene 2 |
| RPS2P5 | Ribosomal Protein S2 Pseudogene 5 |
| PTMAP2 | Prothymosin Alpha Pseudogene 2 |
| PTGES3P1 | Prostaglandin E Synthase 3 Pseudogene 1 |
| IGBP1P2 | Immunoglobulin (CD79A) Binding Protein 1 Pseudogene 2 |
| ENSG00000249141 |  |
| IATPR | ITGB1 Adjacent Tumor Promoting LncRNA |
| SNORA50C | Small Nucleolar RNA, H/ACA Box 50C |
| TDGF1P2 | Teratocarcinoma-Derived Growth Factor 1 Pseudogene 2 |
| RAMACL | RNA Guanine-7 Methyltransferase Activating Subunit Like (Pseudogene) |
| KIAA2012-AS1 | KIAA2012 Antisense RNA 1 |
| RNU1-11P | RNA, U1 Small Nuclear 11, Pseudogene |
| DEFA11P | Defensin Alpha 11, Pseudogene |
| OR5BS1P | Olfactory Receptor Family 5 Subfamily BS Member 1 Pseudogene |
| OR51C1P | Olfactory Receptor Family 51 Subfamily C Member 1 Pseudogene |
| GNG14 | G Protein Subunit Gamma 14 |
| HOXA10-HOXA9 | HOXA10-HOXA9 Readthrough |
| MIR7704 | MicroRNA 7704 |
| MSH2-OT1 | MSH2 Overlapping Transcript 1 |
| RNVU1-17 | RNA, Variant U1 Small Nuclear 17 |
| RPL7P23 | Ribosomal Protein L7 Pseudogene 23 |
| LOC100192426 | Uncharacterized LOC100192426 |
| LOC100240728 | Uncharacterized LOC100240728 |
| RPL17P39 | Ribosomal Protein L17 Pseudogene 39 |
| RPL18AP3 | Ribosomal Protein L18a Pseudogene 3 |
| RPL23AP1 | Ribosomal Protein L23a Pseudogene 1 |
| RNVU1-1 | RNA, Variant U1 Small Nuclear 1 |
| ALG9-IT1 | ALG9 Intronic Transcript 1 |
| LOC730100 | Uncharacterized LOC730100 |
| CARS1-AS1 | CARS1 Antisense RNA 1 |
| LY6G6F-LY6G6D | LY6G6F-LY6G6D Readthrough |
| MAN1B1-DT | MAN1B1 Divergent Transcript |
| ARHGEF34P | Rho Guanine Nucleotide Exchange Factor 34, Pseudogene |
| ANKRD26P3 | Ankyrin Repeat Domain 26 Pseudogene 3 |
| KIF26B-AS1 | KIF26B Antisense RNA 1 |
| ENSG00000254536 |  |
| KRT18P13 | Keratin 18 Pseudogene 13 |
| TUBAP2 | Tubulin Alpha Pseudogene 2 |
| ZNF236-DT | ZNF236 Divergent Transcript |
| LINC02499 | Long Intergenic Non-Protein Coding RNA 2499 |
| LINC02574 | Long Intergenic Non-Protein Coding RNA 2574 |
| LINC02575 | Long Intergenic Non-Protein Coding RNA 2575 |
| LINC02577 | Long Intergenic Non-Protein Coding RNA 2577 |
| LINC02579 | Long Intergenic Non-Protein Coding RNA 2579 |
| LINC02580 | Long Intergenic Non-Protein Coding RNA 2580 |
| WASIR1 | WASH And IL9R Antisense RNA 1 |
| SMG1P2 | SMG1 Pseudogene 2 |
| SMIM40 | Small Integral Membrane Protein 40 |
| ENSG00000263020 |  |
| LINC01230 | Long Intergenic Non-Protein Coding RNA 1230 |
| ZBTB11-AS1 | ZBTB11 Antisense RNA 1 |
| LINC02218 | Long Intergenic Non-Protein Coding RNA 2218 |
| MIR6875 | MicroRNA 6875 |
| RPS2P46 | Ribosomal Protein S2 Pseudogene 46 |
| ENSG00000248993 |  |
| SPINT5P | Serine Peptidase Inhibitor, Kunitz Type 5, Pseudogene |
| SERPINH1P1 | Serpin Family H Member 1 Pseudogene 1 |
| HSPD1P11 | Heat Shock Protein Family D (Hsp60) Member 1 Pseudogene 11 |
| PPP1R14BP3 | Protein Phosphatase 1 Regulatory Inhibitor Subunit 14B Pseudogene 3 |
| RAB42P1 | RAB42, Member RAS Oncogene Family, Pseudogene 1 |
| PTBP1P | Polypyrimidine Tract Binding Protein 1 Pseudogene |
| SUB1P1 | SUB1 Pseudogene 1 |
| IGKV2-26 | Immunoglobulin Kappa Variable 2-26 (Pseudogene) |
| GTF2IRD1P1 | GTF2I Repeat Domain Containing 1 Pseudogene 1 |
| MIR6727 | MicroRNA 6727 |
| MIR6833 | MicroRNA 6833 |
| MIR6775 | MicroRNA 6775 |
| HNRNPA1P8 | Heterogeneous Nuclear Ribonucleoprotein A1 Pseudogene 8 |
| RNU1-27P | RNA, U1 Small Nuclear 27, Pseudogene |
| RNU1-28P | RNA, U1 Small Nuclear 28, Pseudogene |
| MIR4717 | MicroRNA 4717 |
| OR6K4P | Olfactory Receptor Family 6 Subfamily K Member 4 Pseudogene |
| RNA5SP145 | RNA, 5S Ribosomal Pseudogene 145 |
| HNRNPA1P21 | Heterogeneous Nuclear Ribonucleoprotein A1 Pseudogene 21 |
| RPS20P14 | Ribosomal Protein S20 Pseudogene 14 |
| RNVU1-19 | RNA, Variant U1 Small Nuclear 19 |
| RPL7AP47 | Ribosomal Protein L7a Pseudogene 47 |
| RPLP1P6 | Ribosomal Protein Lateral Stalk Subunit P1 Pseudogene 6 |
| RPL12P33 | Ribosomal Protein L12 Pseudogene 33 |
| RPL13AP2 | Ribosomal Protein L13a Pseudogene 2 |
| LOC100132356 | Uncharacterized LOC100132356 |
| RPL6P27 | Ribosomal Protein L6 Pseudogene 27 |
| RPL21P126 | Ribosomal Protein L21 Pseudogene 126 |
| LOC100289495 | Uncharacterized LOC100289495 |
| RPL21P7 | Ribosomal Protein L21 Pseudogene 7 |
| RPL22P3 | Ribosomal Protein L22 Pseudogene 3 |
| RPL23AP42 | Ribosomal Protein L23a Pseudogene 42 |
| RPL26P30 | Ribosomal Protein L26 Pseudogene 30 |
| RPL31P57 | Ribosomal Protein L31 Pseudogene 57 |
| RNVU1-14 | RNA, Variant U1 Small Nuclear 14 |
| RPS26P28 | Ribosomal Protein S26 Pseudogene 28 |
| RPS3P7 | Ribosomal Protein S3 Pseudogene 7 |
| BTF3L4P2 | Basic Transcription Factor 3 Like 4 Pseudogene 2 |
| BRDTP1 | Bromodomain Testis Associated Pseudogene 1 |
| LOC728158 | Uncharacterized LOC728158 |
| LOC729254 | HCG2045843 |
| LOC642484 | Uncharacterized LOC642484 |
| CALM2P1 | Calmodulin 2 Pseudogene 1 |
| ERVK-9 | Endogenous Retrovirus Group K Member 9 |
| CCT7P2 | Chaperonin Containing TCP1 Subunit 7 Pseudogene 2 |
| KRT8P3 | Keratin 8 Pseudogene 3 |
| KRT18P16 | Keratin 18 Pseudogene 16 |
| LOC107984208 | Uncharacterized LOC107984208 |
| LINC01952 | Long Intergenic Non-Protein Coding RNA 1952 |
| LINC02431 | Long Intergenic Non-Protein Coding RNA 2431 |
| LINC02656 | Long Intergenic Non-Protein Coding RNA 2656 |
| LINC02702 | Long Intergenic Non-Protein Coding RNA 2702 |
| XBP1P1 | X-Box Binding Protein 1 Pseudogene 1 |
| PHKA1P1 | Phosphorylase Kinase Regulatory Subunit Alpha 1 Pseudogene |
| SMIM11P1 | Small Integral Membrane Protein 11 Pseudogene 1 |
| IMMTP1 | Inner Membrane Mitochondrial Protein Pseudogene 1 |
| FKSG29 | FKSG29 |
| LINC02667 | Long Intergenic Non-Protein Coding RNA 2667 |
| RNU5E-6P | RNA, U5E Small Nuclear 6, Pseudogene |
| RPS2P40 | Ribosomal Protein S2 Pseudogene 40 |
| RPS27P3 | Ribosomal Protein S27 Pseudogene 3 |
| PTMAP3 | Prothymosin Alpha Pseudogene 3 |
| PTMAP4 | Prothymosin Alpha Pseudogene 4 |
| PTGES3P3 | Prostaglandin E Synthase 3 Pseudogene 3 |
| IFITM8P | Interferon Induced Transmembrane Protein 8 Pseudogene |
| PHF2P2 | PHD Finger Protein 2 Pseudogene 2 |
| SERPINB8P1 | Serpin Family B Member 8 Pseudogene 1 |
| HSPD1P16 | Heat Shock Protein Family D (Hsp60) Member 1 Pseudogene 16 |
| PP2672 | Uncharacterized LOC100130249 |
| ENSG00000243501 |  |
| RAP1BL | RAP1B, Member Of RAS Oncogene Family Pseudogene |
| PCED1CP | PC-Esterase Domain Containing 1C, Pseudogene |
| PABPC1P1 | Poly(A) Binding Protein Cytoplasmic 1 Pseudogene 1 |
| SVIL2P | Supervillin Family Member 2, Pseudogene |
| ENSG00000249773 |  |
| MIR3648-2 | MicroRNA 3648-2 |
| HMGN1P37 | High Mobility Group Nucleosome Binding Domain 1 Pseudogene 37 |
| HMGA1P3 | High Mobility Group AT-Hook 1 Pseudogene 3 |
| DBF4P1 | DBF4 Zinc Finger Pseudogene 1 |
| MIR6826 | MicroRNA 6826 |
| MIR6832 | MicroRNA 6832 |
| EEF1A1P3 | Eukaryotic Translation Elongation Factor 1 Alpha 1 Pseudogene 3 |
| EEF1B2P3 | Eukaryotic Translation Elongation Factor 1 Beta 2 Pseudogene 3 |
| EIF3FP3 | Eukaryotic Translation Initiation Factor 3 Subunit F Pseudogene 3 |
| RNU1-19P | RNA, U1 Small Nuclear 19, Pseudogene |
| E2F6P4 | E2F Transcription Factor 6 Pseudogene 4 |
| RN7SL4P | RNA, 7SL, Cytoplasmic 4, Pseudogene |
| RN7SL5P | RNA, 7SL, Cytoplasmic 5, Pseudogene |
| SETP4 | SET Pseudogene 4 |
| LOC102723566 | Uncharacterized LOC102723566 |
| SERF2-C15ORF63 | SERF2-C15orf63 Readthrough |
| GNL3LP1 | G Protein Nucleolar 3 Like Pseudogene 1 |
| RNA5SP74 | RNA, 5S Ribosomal Pseudogene 74 |
| ELOBP2 | Elongin B Pseudogene 2 |
| RPL7AP66 | Ribosomal Protein L7a Pseudogene 66 |
| ENSG00000225528 |  |
| RPL7P9 | Ribosomal Protein L7 Pseudogene 9 |
| RPL15P15 | Ribosomal Protein L15 Pseudogene 15 |
| RPL18AP4 | Ribosomal Protein L18a Pseudogene 4 |
| ENSG00000173867 |  |
| RPL35P5 | Ribosomal Protein L35 Pseudogene 5 |
| RPL36AP8 | Ribosomal Protein L36a Pseudogene 8 |
| RPL36P11 | Ribosomal Protein L36 Pseudogene 11 |
| RPL39P3 | Ribosomal Protein L39 Pseudogene 3 |
| RPL4P5 | Ribosomal Protein L4 Pseudogene 5 |
| RPL23AP14 | Ribosomal Protein L23a Pseudogene 14 |
| RPL23AP74 | Ribosomal Protein L23a Pseudogene 74 |
| RPL26P32 | Ribosomal Protein L26 Pseudogene 32 |
| ENSG00000204422 |  |
| LOC101928697 | Uncharacterized LOC101928697 |
| RPS3P6 | Ribosomal Protein S3 Pseudogene 6 |
| RPS4XP17 | Ribosomal Protein S4X Pseudogene 17 |
| FSCN1P1 | Fascin Actin-Bundling Protein 1 Pseudogene 1 |
| AKR1B1P3 | Aldo-Keto Reductase Family 1 Member B1 Pseudogene 3 |
| LOC114841035 | Uncharacterized Protein LOC114841035 |
| LOC114841037 | Uncharacterized Protein 114841037 |
| AZF1 | Azoospermia Factor 1 |
| BMS1P21 | BMS1 Pseudogene 21 |
| LOC441488 | Transcription Factor Dp-1-Like Pseudogene |
| LYPLA2P2 | LYPLA2 Pseudogene 2 |
| ENSG00000287363 |  |
| ERV9-1 | Endogenous Retrovirus Group 9 Member 1 |
| KRT8P34 | Keratin 8 Pseudogene 34 |
| KRT8P39 | Keratin 8 Pseudogene 39 |
| KRT18P15 | Keratin 18 Pseudogene 15 |
| ENSG00000260537 |  |
| ENSG00000277726 |  |
| LINC02078 | Long Intergenic Non-Protein Coding RNA 2078 |
| LINC02576 | Long Intergenic Non-Protein Coding RNA 2576 |
| LINC02578 | Long Intergenic Non-Protein Coding RNA 2578 |
| UQCRFS1P2 | Ubiquinol-Cytochrome C Reductase, Rieske Iron-Sulfur Polypeptide 1 Pseudogene 2 |
| WRBP1 | Tryptophan Rich Basic Protein Pseudogene 1 |
| WDR82P1 | WD Repeat Domain 82 Pseudogene 1 |
| LARP4P | LARP4 Pseudogene |
| PCDHA14 | Protocadherin Alpha 14 Pseudogene |
| PTPN2P1 | PTPN2 Pseudogene 1 |
| H2BW4P | H2B.W Histone 4, Pseudogene |
| RPS26P15 | Ribosomal Protein S26 Pseudogene 15 |
| TBC1D7-LOC100130357 | TBC1D7-LOC100130357 Readthrough |
| TAF11L6 | TATA-Box Binding Protein Associated Factor 11 Like 6 |
| ENSG00000255330 |  |
| IGHV4-80 | Immunoglobulin Heavy Variable 4-80 (Pseudogene) |
| LSM12P1 | LSM12 Pseudogene 1 |
| HMGN1P31 | High Mobility Group Nucleosome Binding Domain 1 Pseudogene 31 |
| HIGD1AP1 | HIG1 Hypoxia Inducible Domain Family Member 1A Pseudogene 1 |
| CYP4F23P | Cytochrome P450 Family 4 Subfamily F Member 23, Pseudogene |
| EEF1B2P6 | Eukaryotic Translation Elongation Factor 1 Beta 2 Pseudogene 6 |
| CROCCP4 | CROCC Pseudogene 4 |
| HOTS | H19 Opposite Tumor Suppressor |
| RNA5SP259 | RNA, 5S Ribosomal Pseudogene 259 |
| RNA5SP352 | RNA, 5S Ribosomal Pseudogene 352 |
| RPS15AP34 | Ribosomal Protein S15a Pseudogene 34 |
| RPS15AP9 | Ribosomal Protein S15a Pseudogene 9 |
| RPL17P7 | Ribosomal Protein L17 Pseudogene 7 |
| RPL18P11 | Ribosomal Protein L18 Pseudogene 11 |
| ENSG00000124593 |  |
| RPL35AP35 | Ribosomal Protein L35a Pseudogene 35 |
| RPL36AP7 | Ribosomal Protein L36a Pseudogene 7 |
| RPL27AP6 | Ribosomal Protein L27a Pseudogene 6 |
| RPL31P50 | Ribosomal Protein L31 Pseudogene 50 |
| RPSAP14 | Ribosomal Protein SA Pseudogene 14 |
| GCRG224 | Gastric Cancer-Related Gene GCRG224 |
| FTH1P23 | Ferritin Heavy Chain 1 Pseudogene 23 |
| ENSG00000285991 |  |
| ENSG00000286105 |  |
| ENSG00000282218 |  |
| ENSG00000282804 |  |
| ENSG00000283706 |  |
| ENSG00000283977 |  |
| ENSG00000284762 |  |
| ENSG00000285791 |  |
| ATP5F1CP1 | ATP Synthase F1 Subunit Gamma Pseudogene 1 |
| LOC392196 | Ubiquitin Specific Peptidase 17-Like Family Member 2 Pseudogene |
| LOC728024 | Chromosome X Open Reading Frame 56 Pseudogene |
| LOC642502 | Succinate Dehydrogenase Complex Subunit C Pseudogene |
| ENSG00000288520 |  |
| KRT8P48 | Keratin 8 Pseudogene 48 |
| LINC02631 | Long Intergenic Non-Protein Coding RNA 2631 |
| WBP2P1 | WW Domain Binding Protein 2 Pseudogene 1 |
| TOMM22P3 | TOMM22 Pseudogene 3 |
| SMIM28 | Small Integral Membrane Protein 28 |
| ENSG00000272162 |  |
| ENSG00000272442 |  |
| NMBR-AS1 | NMBR Antisense RNA 1 |
| LOC101927960 | Uncharacterized LOC101927960 |
| SERPINA15P | Serpin Family A Member 15, Pseudogene |
| TAF11L10 | TATA-Box Binding Protein Associated Factor 11 Like 10 |
| SERPINA7P1 | Serpin Family A Member 7 Pseudogene 1 |
| TAF11L5 | TATA-Box Binding Protein Associated Factor 11 Like 5 |
| TAF11L7 | TATA-Box Binding Protein Associated Factor 11 Like 7 |
| TAF11L8 | TATA-Box Binding Protein Associated Factor 11 Like 8 |
| TMEM161BP1 | Transmembrane Protein 161B Pseudogene 1 |
| HMGB1P40 | High Mobility Group Box 1 Pseudogene 40 |
| EIF2S2P7 | Eukaryotic Translation Initiation Factor 2 Subunit 2 Beta Pseudogene 7 |
| RNU1-18P | RNA, U1 Small Nuclear 18, Pseudogene |
| CORD1 | Cone Rod Dystrophy 1 (Autosomal Dominant) |
| MSRB1P1 | Methionine Sulfoxide Reductase B1 Pseudogene 1 |
| MRPS35P1 | Mitochondrial Ribosomal Protein S35 Pseudogene 1 |
| HPT | Hypoparathyroidism |
| DDX3P3 | DEAD-Box Helicase 3 Pseudogene 3 |
| OR10Q2P | Olfactory Receptor Family 10 Subfamily Q Member 2 Pseudogene |
| SETDB2-PHF11 | SETDB2-PHF11 Readthrough |
| CHCHD2P8 | Coiled-Coil-Helix-Coiled-Coil-Helix Domain Containing 2 Pseudogene 8 |
| RNA5SP191 | RNA, 5S Ribosomal Pseudogene 191 |
| RNA5SP202 | RNA, 5S Ribosomal Pseudogene 202 |
| CYP4F9P | Cytochrome P450 Family 4 Subfamily F Member 9, Pseudogene |
| RPS15P4 | Ribosomal Protein S15 Pseudogene 4 |
| RPS18P5 | Ribosomal Protein S18 Pseudogene 5 |
| RPLP1P7 | Ribosomal Protein Lateral Stalk Subunit P1 Pseudogene 7 |
| RPL17P6 | Ribosomal Protein L17 Pseudogene 6 |
| ENSG00000214265 |  |
| RPL18AP16 | Ribosomal Protein L18a Pseudogene 16 |
| RPL32P36 | Ribosomal Protein L32 Pseudogene 36 |
| RPL36AP10 | Ribosomal Protein L36a Pseudogene 10 |
| RPL28P1 | Ribosomal Protein L28 Pseudogene 1 |
| RPL31P45 | Ribosomal Protein L31 Pseudogene 45 |
| RPL32P18 | Ribosomal Protein L32 Pseudogene 18 |
| RPL32P19 | Ribosomal Protein L32 Pseudogene 19 |
| RPL32P28 | Ribosomal Protein L32 Pseudogene 28 |
| RPS2P14 | Ribosomal Protein S2 Pseudogene 14 |
| RPS5P5 | Ribosomal Protein S5 Pseudogene 5 |
| RPSAP26 | Ribosomal Protein SA Pseudogene 26 |
| CDKN2AIPNLP1 | CDKN2A Interacting Protein N-Terminal Like Pseudogene 1 |
| ENSG00000285976 |  |
| ABCD1P5 | ATP Binding Cassette Subfamily D Member 1 Pseudogene 5 |
| ENSG00000281613 |  |
| ENSG00000282988 |  |
| ENSG00000284024 |  |
| ACTG1P14 | Actin Gamma 1 Pseudogene 14 |
| ENSG00000284554 |  |
| ENSG00000285446 |  |
| LOC339902 | HCG1813818 |
| ENSG00000285733 |  |
| LOC649352 | Ubiquitin Carboxyl-Terminal Hydrolase 17-Like Protein 2-Like |
| LOC642204 | Ankyrin Repeat Domain 20 Family Member A1 Pseudogene |
| LOC642696 | Uncharacterized LOC642696 |
| AAA1 | Aortic Aneurysm, Familial Abdominal 1 |
| ENSG00000286920 |  |
| ENSG00000280148 |  |
| ACF | Asymmetric Crying Facies (Cayler Cardiofacial Syndrome) |
| ARPP19P1 | ARPP19 Pseudogene 1 |
| ARPP19P2 | ARPP19 Pseudogene 2 |
| ENSG00000285723 |  |
| ENSG00000283189 |  |
| ENSG00000257065 |  |
| UBTFL7 | UBTF Like 7 (Pseudogene) |
| PCDHACT | Protocadherin Alpha Constant |
| ENSG00000271793 |  |
| ENSG00000266826 |  |
| SERPINE4P | Serpin Family E Member 4, Pseudogene |
| CYAT1 | Immunoglobulin Lambda Light Chain-Like |
| RNA5SP506 | RNA, 5S Ribosomal Pseudogene 506 |
| H3P44 | H3 Histone Pseudogene 44 |
| RNA28SN5 | RNA, 28S Ribosomal N5 |
| MGAT4FP | MGAT4 Family Member F, Pseudogene |
| MYL6P5 | Myosin Light Chain 6 Pseudogene 5 |
| RPL27AP2 | Ribosomal Protein L27a Pseudogene 2 |
| LOC101928354 | Uncharacterized LOC101928354 |
| ENSG00000284341 |  |
| ENSG00000285085 |  |
| ATP5MGP4 | ATP Synthase Membrane Subunit G Pseudogene 4 |
| LOC642969 | Phosphoglycerate Mutase 1 (Brain) Pseudogene |
| KRT18P11 | Keratin 18 Pseudogene 11 |
| TRAPPC13P1 | Trafficking Protein Particle Complex 13 Pseudogene 1 |
| VN1R106P | Vomeronasal 1 Receptor 106 Pseudogene |
| ENSG00000276302 |  |
| USP17L14P | Ubiquitin Specific Peptidase 17 Like Family Member 14, Pseudogene |
| USP17L16P | Ubiquitin Specific Peptidase 17 Like Family Member 16, Pseudogene |
| WBSCR23 | Williams-Beuren Syndrome Chromosome Region 23 |
| XS | X-Linked Suppressor Of LU Antigens |
| ENSG00000268655 |  |
| H2ACP1 | H2AC Histone Family Pseudogene 1 |
| NDUFAF2P1 | NDUFAF2 Pseudogene 1 |
| THM | Thymoma |
| PRO2268 | Uncharacterized Protein PRO2268 |
| LOC105378085 | Uncharacterized LOC105378085 |
| IGHV1-38-4 | Immunoglobulin Heavy Variable 1-38-4 (Non-Functional) |
| IGHV3-38-3 | Immunoglobulin Heavy Variable 3-38-3 (Non-Functional) |
| C5-OT1 | C5 3' UTR Overlapping Transcript 1 |
| RIEG2 | Rieger Syndrome 2 |
| OTSC4 | Otosclerosis 4 |
| RNA5SP162 | RNA, 5S Ribosomal Pseudogene 162 |
| LOC100129100 | Uncharacterized Protein C12orf29 Homolog |
| LOC100421094 | Ubiquitin Specific Peptidase 17-Like Family Member 2 Pseudogene |
| LOC100420746 | NADH:Ubiquinone Oxidoreductase Complex Assembly Factor 7 Pseudogene |
| OCTN3 | Organic Cation Transporter 3 |
| AIR | Acute Insulin Response (2) |
| LOC644667 | Golgin A7 Pseudogene |
| LOC392187 | Ubiquitin Carboxyl-Terminal Hydrolase 17-Like Protein 2-Like |
| LOC728688 | Ubiquitin Like With PHD And Ring Finger Domains 1 Pseudogene |
| LOC643576 | Phosphoglycerate Mutase 1 (Brain) Pseudogene |
| LOC442293 | Glycine Cleavage System Protein H (Aminomethyl Carrier) Pseudogene |
| RCHTS | Roifman-Chitayat Syndrome |
| NAIPP1 | NAIP Pseudogene 1 |
| DEL22Q11.2 | Chromosome 22q11.2 Deletion Syndrome, Distal |
| LOC100129274 | NGG1 Interacting Factor 3 Like 1 Pseudogene |
| LOC647481 | Family With Sequence Similarity 91 Member A1 Pseudogene |
| LOC402329 | Ubiquitin Carboxyl-Terminal Hydrolase 17-Like Protein 2-Like |
| IGSF6-DREV1 | Region Containing Immunoglobulin Superfamily, Member 6 And DREV1 |
| LOC110386947 | CYP19A1 Promoter I.1 |
| LOC110599571 | CYP2C8 Promoter |
| LOC110631417 | CYP21A2 5' Regulatory Region |
| LOC110806263 | TERT 5' Regulatory Region |
| LOC111089946 | NAIP Promoter Region |
| LOC111099027 | Transmembrane Protease Serine 2 Breakpoint Cluster Recombination Region |
| LOC111099028 | ERG, ETS Transcription Factor Breakpoint Cluster Recombination Region |
| LOC111162621 | DeltaNp63 Promoter Of Tumor Protein P63 |
| LOC111258525 | NOS1 1f And 1g Alternate Promoter Region |
| LOC108167315 | POMC Promoter Region |
| LOC440084 | Uncharacterized LOC440084 |
| LOC108511947 | ALAS2 Intronic Erythroid Enhancer |
| LOC108863620 | STAR 5' Regulatory Region |
| LOC109611593 | RUNX2 P1 Promoter Region |
| LOC110013312 | BGLAP Promoter Region |
| LOC390846 | Golgi SNAP Receptor Complex Member 2 Pseudogene |
| LOC106128905 | KLK3 Upstream Enhancer/Promoter Region |
| LOC106146150 | Int22h-1 Recombination Region |
| LOC106146151 | Int22h-2 Recombination Region |
| LOC106146152 | Int22h-3 Recombination Region |
| LOC106728418 | LEP 5' Regulatory Region |
| LOC107105350 | NUP98-HOXC11 Recombination Region |
| LOC107126281 | NUP98-HOXA11 Recombination Region |
| LOC107197952 | NUP98-HOXA9 Recombination Region |
| LOC107963948 | BCR-ABL Micro-Breakpoint Cluster Region |
| LOC107985820 | Uncharacterized LOC107985820 |
