# Supplementary material for: Integrated Analyses Identify Immune-Related Signature Associated with Qingyihuaji Formula for Treatment of Pancreatic Ductal Adenocarcinoma Using Network Pharmacology and Weighted Gene Co-Expression Network
Source: J Immunol Res. 2020 May 20;2020:7503605. doi: 10.1155/2020/7503605 (PMC7256764; doi:10.1155/2020/7503605)
Supplement: Supplementary Materials — Table 1S. The PDAC genes in the GeneCards database Supplementary file 2: Table 2S. The immune-related targets in the GeneCards database Supplementary file 3: Table 3S. The detailed ADME parameters and the SMILES information of the aforementioned 67 selected compounds. Supplementary file 4: Figure S1. The potential active ingredients-target network of QYHJ. Supplementary file 5: Table 4S. The association between active ingredients and up-regulated overlapping genes. Supplementary file 6: Figure S2. Association of Methylation sites with the expression of 12 overlapped DEGs. [file 7503605.f1.zip › Supplementary file Table 3S.doc]

**Supplementary Table Ⅱ** The detailed ADME parameters and the SMILES information of 67 selected compounds.

Tiannanxing (Rhizoma Arisaematis erubescentis, RAE)

| SMILES | Mumbers | [**Mol ID**](http://lsp.nwu.edu.cn/tcmspsearch.php?qr=Arisaematis Rhizoma&qsr=herb_en_name&token=625d16b741fb2795959ada57190a7cd4) | [**Molecule Name**](http://lsp.nwu.edu.cn/tcmspsearch.php?qr=Arisaematis Rhizoma&qsr=herb_en_name&token=625d16b741fb2795959ada57190a7cd4) | [**OB (%)**](http://lsp.nwu.edu.cn/tcmspsearch.php?qr=Arisaematis Rhizoma&qsr=herb_en_name&token=625d16b741fb2795959ada57190a7cd4) | [**DL**](http://lsp.nwu.edu.cn/tcmspsearch.php?qr=Arisaematis Rhizoma&qsr=herb_en_name&token=625d16b741fb2795959ada57190a7cd4) | [**HL**](http://lsp.nwu.edu.cn/tcmspsearch.php?qr=Arisaematis Rhizoma&qsr=herb_en_name&token=625d16b741fb2795959ada57190a7cd4) |
| --- | --- | --- | --- | --- | --- | --- |
| O(C(=O)CCCCCC/C=C/C/C=C/C/C=C/CCCCCCC)C | RAE-1 | MOL013146 | [8,11,14-Docosatrienoic acid, methyl ester](http://lsp.nwu.edu.cn/molecule.php?qn=13146) | 43.23 | 0.3 | 5.29 |
| O(C[C@@H](CNC(=O)[C@H](NC(=O)c1ccccc1)Cc1ccccc1)Cc1ccccc1)C(=O)C | RAE-2 | MOL013156 | [[(2R)-2-[[[(2R)-2-(benzoylamino)-3-phenylpropanoyl]amino]methyl]-3-phenylpropyl] acetate](http://lsp.nwu.edu.cn/molecule.php?qn=13156) | 38.88 | 0.56 | 6.46 |
| O[C@@H]1CC2=CC[C@H]3[C@H]4[C@@]([C@H](CC4)[C@@H](CC[C@@H](C(C)C)C)C)(CC[C@@H]3[C@]2(CC1)C)C | RAE-3 | MOL001510 | [24-epicampesterol](http://lsp.nwu.edu.cn/molecule.php?qn=1510) | 37.58 | 0.71 | 4.5 |
| O[C@@H]1CC2=CC[C@H]3[C@H]4[C@@]([C@H](CC4)[C@@H](CC[C@H](C(C)C)CC)C)(CC[C@@H]3[C@]2(CC1)C)C | RAE-4 | MOL000358 | [beta-sitosterol](http://lsp.nwu.edu.cn/molecule.php?qn=358) | 36.91 | 0.75 | 5.36 |
| O[C@@H]1CC2=CC[C@H]3[C@H]4[C@@]([C@H](CC4)[C@H](C)/C=C/[C@H](C(C)C)CC)(CC[C@@H]3[C@]2(CC1)C)C | RAE-5 | MOL000449 | [Stigmasterol](http://lsp.nwu.edu.cn/molecule.php?qn=449) | 43.83 | 0.76 | 5.57 |
| O[C@@H]1CC2=CC[C@H]3[C@H]4[C@@]([C@H](CC4)[C@@H](CCCC(C)C)C)(CC[C@@H]3[C@]2(CC1)C)C | RAE-6 | MOL000953 | [CLR](http://lsp.nwu.edu.cn/molecule.php?qn=953) | 37.87 | 0.68 | 4.52 |

Banzhilian (*Herba Scutellariae Barbatae,* HSB)

| SMILES | Mumbers | [**Mol ID**](http://lsp.nwu.edu.cn/tcmspsearch.php?qr=Scutellariae Barbatae Herba&qsr=herb_en_name&token=625d16b741fb2795959ada57190a7cd4) | [**Molecule Name**](http://lsp.nwu.edu.cn/tcmspsearch.php?qr=Scutellariae Barbatae Herba&qsr=herb_en_name&token=625d16b741fb2795959ada57190a7cd4) | [**OB (%)**](http://lsp.nwu.edu.cn/tcmspsearch.php?qr=Scutellariae Barbatae Herba&qsr=herb_en_name&token=625d16b741fb2795959ada57190a7cd4) | [**DL**](http://lsp.nwu.edu.cn/tcmspsearch.php?qr=Scutellariae Barbatae Herba&qsr=herb_en_name&token=625d16b741fb2795959ada57190a7cd4) | [**HL**](http://lsp.nwu.edu.cn/tcmspsearch.php?qr=Scutellariae Barbatae Herba&qsr=herb_en_name&token=625d16b741fb2795959ada57190a7cd4) |
| --- | --- | --- | --- | --- | --- | --- |
| O1[C@H](CC(=O)c2c1cc(O)cc2O)c1ccc(O)cc1 | HSB-1 | MOL001040 | [(2R)-5,7-dihydroxy-2-(4-hydroxyphenyl)chroman-4-one](http://lsp.nwu.edu.cn/molecule.php?qn=1040) | 42.36 | 0.21 | 16.83 |
| O1[C@H](CC(=O)c2c1cc(O)c(OC)c2O)c1ccc(O)cc1 | HSB-2 | MOL012245 | [5,7,4'-trihydroxy-6-methoxyflavanone](http://lsp.nwu.edu.cn/molecule.php?qn=12245) | 36.63 | 0.27 | 16.12 |
| c1(c2c(c(c(c1)O)OC)oc(cc2=O)c1ccc(cc1)O)O | HSB-3 | MOL012246 | [5,7,4'-trihydroxy-8-methoxyflavanone](http://lsp.nwu.edu.cn/molecule.php?qn=12246) | 74.24 | 0.26 | 16.85 |
| o1c2c(c(O)cc(OC)c2OC)c(=O)cc1c1ccc(OC)cc1 | HSB-4 | MOL012248 | [5-hydroxy-7,8-dimethoxy-2-(4-methoxyphenyl)chromone](http://lsp.nwu.edu.cn/molecule.php?qn=12248) | 65.82 | 0.33 | 16.41 |
| o1c2c(c(OC)cc(O)c2OC)c(=O)cc1c1ccccc1 | HSB-5 | MOL012250 | [7-hydroxy-5,8-dimethoxy-2-phenyl-chromone](http://lsp.nwu.edu.cn/molecule.php?qn=12250) | 43.72 | 0.25 | 16.77 |
| o1c2c(c(OC)cc(O)c2)c(=O)cc1c1ccccc1 | HSB-6 | MOL012251 | [Chrysin-5-methylether](http://lsp.nwu.edu.cn/molecule.php?qn=12251) | 37.27 | 0.2 | 17.24 |
| [C@H]1(C([C@H]2[C@]3(CC1)[C@@]1([C@@H](CC2)[C@]2([C@](CC1)([C@H](CC2)C(CCC=C(C)C)C)C)C)C3)(C)C)OC(=O)C | HSB-7 | MOL012252 | [9,19-cyclolanost-24-en-3-ol](http://lsp.nwu.edu.cn/molecule.php?qn=12252) | 38.69 | 0.78 | 5.41 |
| O1[C@@H](Oc2cc3oc(cc(=O)c3c(O)c2O)c2ccccc2)[C@H](O)[C@@H](O)[C@H](O)[C@H]1C(=O)O | HSB-8 | MOL002776 | [Baicalin](http://lsp.nwu.edu.cn/molecule.php?qn=2776) | 40.12 | 0.75 | 17.36 |
| C1=C(CC2[C@](C1)([C@@H]1[C@@H](CC2)[C@H]2[C@](CC1)([C@H](CC2)[C@@H](CC[C@H](C(C)C)C)C)C)C)O | HSB-9 | MOL012254 | [campesterol](http://lsp.nwu.edu.cn/molecule.php?qn=12254) | 37.58 | 0.71 | 4.63 |
| O[C@@H]1CC2=CC[C@H]3[C@H]4[C@@]([C@H](CC4)[C@@H](CCCC(C)C)C)(CC[C@@H]3[C@]2(CC1)C)C | HSB-10 | MOL000953 | [CLR](http://lsp.nwu.edu.cn/molecule.php?qn=953) | 37.87 | 0.68 | 4.52 |
| O[C@@H]1CC2=CC[C@H]3[C@H]4[C@@]([C@H](CC4)[C@@H](CC[C@H](C(C)C)CC)C)(CC[C@@H]3[C@]2(CC1)C)C | HSB-11 | MOL000358 | [beta-sitosterol](http://lsp.nwu.edu.cn/molecule.php?qn=358) | 36.91 | 0.75 | 5.36 |
| o1c2c(c(=O)cc1c1c(OC)cccc1O)c(O)cc(OC)c2OC | HSB-12 | MOL012266 | [rivularin](http://lsp.nwu.edu.cn/molecule.php?qn=12266) | 37.94 | 0.37 | 16.25 |
| O([C@@H]1CC2=CC[C@H]3[C@H]4[C@@]([C@H](CC4)[C@@H](CC[C@H](C(C)C)CC)C)(CC[C@@H]3[C@]2(CC1)C)C)C(=O)C | HSB-13 | MOL001973 | [Sitosteryl acetate](http://lsp.nwu.edu.cn/molecule.php?qn=1973) | 40.39 | 0.85 | 6.34 |
| C1(CC2=CCC3C(C2(CC1)C)CCC1(C3CCC1C(C)/C=C\C(CC)C(C)C)C)OC(=O)C | HSB-14 | MOL012269 | [Stigmasta-5,22-dien-3-ol-acetate](http://lsp.nwu.edu.cn/molecule.php?qn=12269) | 46.44 | 0.86 | 6.77 |
| C12=CC[C@@H]3C([C@]2(CCC=C1)C)CC[C@]1(C3CCC1[C@@H](C)/C=C/[C@H](C(C)C)CC)C | HSB-15 | MOL012270 | [Stigmastan-3,5,22-triene](http://lsp.nwu.edu.cn/molecule.php?qn=12270) | 45.03 | 0.71 | 6.21 |
| O[C@@H]1CC2=CC[C@H]3[C@H]4[C@@]([C@H](CC4)[C@H](C)/C=C/[C@H](C(C)C)CC)(CC[C@@H]3[C@]2(CC1)C)C | HSB-16 | MOL000449 | [Stigmasterol](http://lsp.nwu.edu.cn/molecule.php?qn=449) | 43.83 | 0.76 | 5.57 |
| o1c2c(c(=O)cc1c1ccccc1)c(O)cc(O)c2OC | HSB-17 | MOL000173 | [wogonin](http://lsp.nwu.edu.cn/molecule.php?qn=173) | 30.68 | 0.23 | 17.75 |
| o1c2c(c(O)c(OC)c(O)c2)c(=O)cc1c1ccc(O)cc1 | HSB-18 | MOL001735 | [Dinatin](http://lsp.nwu.edu.cn/molecule.php?qn=1735) | 30.97 | 0.27 | 16.44 |
| O=C1CC[C@@]2([C@@H]3[C@H]([C@H]4[C@@]([C@H](CC4)[C@@H](CC[C@H](C(C)C)CC)C)(CC3)C)CCC2=C1)C | HSB-19 | MOL001755 | [24-Ethylcholest-4-en-3-one](http://lsp.nwu.edu.cn/molecule.php?qn=1755) | 36.08 | 0.76 | 5.49 |
| o1c2c(c(O)c(O)c(O)c2)c(=O)cc1c1ccccc1 | HSB-20 | MOL002714 | [baicalein](http://lsp.nwu.edu.cn/molecule.php?qn=2714) | 33.52 | 0.21 | 16.25 |
| O1[C@@H](CC(=O)c2c1cc(O)c(O)c2O)c1ccc(O)cc1 | HSB-21 | MOL002719 | [6-Hydroxynaringenin](http://lsp.nwu.edu.cn/molecule.php?qn=2719) | 33.23 | 0.24 | 15.67 |
| o1c2c(c(O)c(OC)c(OC)c2)c(=O)cc1c1ccc(OC)cc1 | HSB-22 | MOL002915 | [Salvigenin](http://lsp.nwu.edu.cn/molecule.php?qn=2915) | 49.07 | 0.33 | 15.87 |
| o1c2c(c(=O)c(O)c1c1cc(OC)c(O)cc1)c(O)cc(OC)c2 | HSB-23 | MOL000351 | [Rhamnazin](http://lsp.nwu.edu.cn/molecule.php?qn=351) | 47.14 | 0.34 | 13.54 |
| O[C@H]1CC2=CC[C@H]3[C@H]4[C@@]([C@H](CC4)[C@@H](CC[C@H](C(C)C)CC)C)(CC[C@@H]3[C@]2(CC1)C)C | HSB-24 | MOL000359 | [sitosterol](http://lsp.nwu.edu.cn/molecule.php?qn=359) | 36.91 | 0.75 | 5.37 |
| O1[C@@H](CC(=O)c2c1cc(O)cc2O)c1cc(O)c(O)cc1 | HSB-25 | MOL005190 | [eriodictyol](http://lsp.nwu.edu.cn/molecule.php?qn=5190) | 71.79 | 0.24 | 15.81 |
| [C@H]1([C@H]([C@@H]([C@H]([C@@H](O1)O[C@@H]1CCC2=CC[C@@H]3[C@@H]([C@]2(C1)C)CC[C@]1([C@H]3CC[C@@H]1[C@@H](CC[C@H](C(C)C)CC)C)C)O)O)O)CO | HSB-26 | MOL005869 | [daucostero_qt](http://lsp.nwu.edu.cn/molecule.php?qn=5869) | 36.91 | 0.75 | 5.08 |
| o1c2c(c(=O)cc1c1cc(O)c(O)cc1)c(O)cc(O)c2 | HSB-27 | MOL000006 | [luteolin](http://lsp.nwu.edu.cn/molecule.php?qn=6) | 36.16 | 0.25 | 15.94 |
| o1c2c(c(O)cc(OC)c2OC)c(=O)cc1c1ccccc1 | HSB-28 | MOL008206 | [Moslosooflavone](http://lsp.nwu.edu.cn/molecule.php?qn=8206) | 44.09 | 0.25 | 17.02 |
| o1c2c(c(=O)c(O)c1c1cc(O)c(O)cc1)c(O)cc(O)c2 | HSB-29 | MOL000098 | [quercetin](http://lsp.nwu.edu.cn/molecule.php?qn=98) | 46.43 | 0.28 | 14.4 |

Baihuasheshecao (*HerbaHedyotdis*, HBHY)

| SMILES | Mumbers | [**Mol ID**](http://lsp.nwu.edu.cn/tcmspsearch.php?qr=Hedyotis Diffusae Herba&qsr=herb_en_name&token=625d16b741fb2795959ada57190a7cd4) | [**Molecule Name**](http://lsp.nwu.edu.cn/tcmspsearch.php?qr=Hedyotis Diffusae Herba&qsr=herb_en_name&token=625d16b741fb2795959ada57190a7cd4) | [**OB (%)**](http://lsp.nwu.edu.cn/tcmspsearch.php?qr=Hedyotis Diffusae Herba&qsr=herb_en_name&token=625d16b741fb2795959ada57190a7cd4) | [**DL**](http://lsp.nwu.edu.cn/tcmspsearch.php?qr=Hedyotis Diffusae Herba&qsr=herb_en_name&token=625d16b741fb2795959ada57190a7cd4) | [**HL**](http://lsp.nwu.edu.cn/tcmspsearch.php?qr=Hedyotis Diffusae Herba&qsr=herb_en_name&token=625d16b741fb2795959ada57190a7cd4) |
| --- | --- | --- | --- | --- | --- | --- |
| c1(cc2c(cc1OC)C(=O)c1c(C2=O)c(ccc1)C)OC | HBHY-1 | MOL001646 | [2,3-dimethoxy-6-methyanthraquinone](http://lsp.nwu.edu.cn/molecule.php?qn=1646) | 34.86 | 0.26 | 28.98 |
| O[C@@H]1CC2=CC[C@H]3[C@H]4[C@@]([C@H](CC4)[C@H](C)/C=C/[C@@H](C(C)C)CC)(CC[C@@H]3[C@]2(CC1)C)C | HBHY-2 | MOL001659 | [Poriferasterol](http://lsp.nwu.edu.cn/molecule.php?qn=1659) | 43.83 | 0.76 | 5.34 |
| O[C@H]1C([C@H]2[C@@]([C@@H]3[C@]([C@]4(C(=CC3)[C@H]3[C@@](CC4)(CCC(C3)(C)C)C(=O)O)C)(CC2)C)(CC1)C)(C)C | HBHY-3 | MOL001663 | [(4aS,6aR,6aS,6bR,8aR,10R,12aR,14bS)-10-hydroxy-2,2,6a,6b,9,9,12a-heptamethyl-1,3,4,5,6,6a,7,8,8a,10,11,12,13,14b-tetradecahydropicene-4a-carboxylic acid](http://lsp.nwu.edu.cn/molecule.php?qn=1663) | 32.03 | 0.76 | 4.34 |
| O(c1c(cc2c(C(=O)c3c(C2=O)cccc3)c1)C)C | HBHY-4 | MOL001670 | [2-methoxy-3-methyl-9,10-anthraquinone](http://lsp.nwu.edu.cn/molecule.php?qn=1670) | 37.83 | 0.21 | 28.82 |
| O[C@@H]1CC2=CC[C@H]3[C@H]4[C@@]([C@H](CC4)[C@H](C)/C=C/[C@H](C(C)C)CC)(CC[C@@H]3[C@]2(CC1)C)C | HBHY-5 | MOL000449 | [Stigmasterol](http://lsp.nwu.edu.cn/molecule.php?qn=449) | 43.83 | 0.76 | 5.57 |
| O[C@@H]1CC2=CC[C@H]3[C@H]4[C@@]([C@H](CC4)[C@@H](CC[C@H](C(C)C)CC)C)(CC[C@@H]3[C@]2(CC1)C)C | HBHY-6 | MOL000358 | [beta-sitosterol](http://lsp.nwu.edu.cn/molecule.php?qn=358) | 36.91 | 0.75 | 5.36 |
| o1c2c(c(=O)c(O)c1c1cc(O)c(O)cc1)c(O)cc(O)c2 | HBHY-7 | MOL000098 | [quercetin](http://lsp.nwu.edu.cn/molecule.php?qn=98) | 46.43 | 0.28 | 14.4 |

Jiaogulan (*Herba seu Radix Gynostemmatis pentaphylli,* HSRGP)

| SMILES | Mumbers | [**Mol ID**](http://lsp.nwu.edu.cn/tcmspsearch.php?qr=Gynostemmae Pentaphylli Herba&qsr=herb_en_name&token=625d16b741fb2795959ada57190a7cd4) | [**Molecule Name**](http://lsp.nwu.edu.cn/tcmspsearch.php?qr=Gynostemmae Pentaphylli Herba&qsr=herb_en_name&token=625d16b741fb2795959ada57190a7cd4) | [**OB (%)**](http://lsp.nwu.edu.cn/tcmspsearch.php?qr=Gynostemmae Pentaphylli Herba&qsr=herb_en_name&token=625d16b741fb2795959ada57190a7cd4) | [**DL**](http://lsp.nwu.edu.cn/tcmspsearch.php?qr=Gynostemmae Pentaphylli Herba&qsr=herb_en_name&token=625d16b741fb2795959ada57190a7cd4) | [**HL**](http://lsp.nwu.edu.cn/tcmspsearch.php?qr=Gynostemmae Pentaphylli Herba&qsr=herb_en_name&token=625d16b741fb2795959ada57190a7cd4) |
| --- | --- | --- | --- | --- | --- | --- |
| O1[C@H](CC(=O)c2c1cc(O)cc2O)c1cc(OC)c(O)cc1 | HSRGP-1 | MOL000338 | [3'-methyleriodictyol](http://lsp.nwu.edu.cn/molecule.php?qn=338) | 51.61 | 0.27 | 16.05 |
| o1c2c(c(=O)c(O)c1c1cc(OC)c(O)cc1)c(O)cc(OC)c2 | HSRGP-2 | MOL000351 | [Rhamnazin](http://lsp.nwu.edu.cn/molecule.php?qn=351) | 47.14 | 0.34 | 13.54 |
| O[C@H]1CC2=CC[C@H]3[C@H]4[C@@]([C@H](CC4)[C@@H](CC[C@H](C(C)C)CC)C)(CC[C@@H]3[C@]2(CC1)C)C | HSRGP-3 | MOL000359 | [sitosterol](http://lsp.nwu.edu.cn/molecule.php?qn=359) | 36.91 | 0.75 | 5.37 |
| C1=C(COC1=O)C1C2(C(CC1)(C1C(CC2)C2(C(CC1)CC(CC2)OC1OC(C(C(C1O)OC)O)C)CO)O)C | HSRGP-4 | MOL004350 | [Ruvoside_qt](http://lsp.nwu.edu.cn/molecule.php?qn=4350) | 36.12 | 0.76 | 7.21 |
| O[C@@H]1C[C@H]2[C@@]([C@@H]3C(=CC2)[C@H]2[C@@]([C@H](CC2)[C@H](C)/C=C/[C@H](C(C)C)CC)(CC3)C)(CC1)C | HSRGP-5 | MOL004355 | [Spinasterol](http://lsp.nwu.edu.cn/molecule.php?qn=4355) | 42.98 | 0.76 | 5.32 |
| C1=C(CC2[C@](C1)([C@@H]1[C@@H](CC2)[C@H]2[C@](CC1)([C@H](CC2)[C@@H](CC[C@H](C(C)C)C)C)C)C)O | HSRGP-6 | MOL005438 | [campesterol](http://lsp.nwu.edu.cn/molecule.php?qn=5438) | 37.58 | 0.71 | 4.83 |
| O[C@@H]1CC2=CC[C@H]3[C@H]4[C@@]([C@H](CC4)[C@@H](CC/C(=C/C)/C(C)C)C)(CC[C@@H]3[C@]2(CC1)C)C | HSRGP-7 | MOL005440 | [Isofucosterol](http://lsp.nwu.edu.cn/molecule.php?qn=5440) | 43.78 | 0.76 | 5.18 |
| O[C@@H]1CC2=CC[C@H]3[C@H]4[C@@]([C@H](CC4)[C@@H](CCCC(C)C)C)(CC[C@@H]3[C@]2(CC1)C)C | HSRGP-8 | MOL000953 | [CLR](http://lsp.nwu.edu.cn/molecule.php?qn=953) | 37.87 | 0.68 | 4.52 |
| o1c2c(c(=O)c(O)c1c1cc(O)c(O)cc1)c(O)cc(O)c2 | HSRGP-9 | MOL000098 | [quercetin](http://lsp.nwu.edu.cn/molecule.php?qn=98) | 46.43 | 0.28 | 14.4 |
| C12=CCC3[C@H]([C@@]1(CC[C@H](C2)O)C)CC[C@@]1(C3CC[C@@H]1[C@H](/C=C/[C@@H](C(=C)C)CC)C)C | HSRGP-10 | MOL009855 | [(24S)-Ethylcholesta-5,22,25-trans-3beta-ol](http://lsp.nwu.edu.cn/molecule.php?qn=9855) | 46.91 | 0.76 | 5.53 |
| C12[C@@](CCC([C@H]1C)O)(C1=CC[C@@]3([C@](C1=CC2)(CC[C@@H]3[C@@H](C)CCC(=C)C(C)C)C)C)C | HSRGP-11 | MOL009867 | [4α,14α-dimethyl-5α-ergosta-7,9(11),24(28)-trien-3β-ol](http://lsp.nwu.edu.cn/molecule.php?qn=9867) | 46.29 | 0.76 | 6.23 |
| C1[C@H](C(C2=CCC3[C@@](C2C1)(CC[C@@]1([C@]3(CC[C@@H]1[C@@H](C)CCC=C(C)C)C)C)C)(C)C)O | HSRGP-12 | MOL009877 | [cucurbita-5,24-dienol](http://lsp.nwu.edu.cn/molecule.php?qn=9877) | 44.02 | 0.74 | 6.05 |
| [C@]123C(C(=C)C(CC1)NC)CC[C@@H]1[C@]2(CC[C@]2([C@@]1(C[C@@H](C2[C@H](NC)C)O)C)C)C3 | HSRGP-13 | MOL009878 | [Cyclobuxine](http://lsp.nwu.edu.cn/molecule.php?qn=9878) | 84.48 | 0.7 | 5.08 |
| C1C(C(C2[C@@](C1)([C@@H]1[C@](CC2)([C@]2(C(CC1)[C@@H](CC2)C(C)(O)CCC=C(C)C)C)C)C=O)(C)C)O | HSRGP-14 | MOL009888 | [Gypenoside XXXVI_qt](http://lsp.nwu.edu.cn/molecule.php?qn=9888) | 37.85 | 0.78 | 8.16 |
| C1[C@H](C(C2[C@@](C1)([C@H]1[C@](CC2)([C@]2(C(C(=O)C1)[C@@H](CC2)C(CC(=O)C=C(C)C)(C)O)C)C)C)(C)C)O | HSRGP-15 | MOL009986 | [Gypentonoside A_qt](http://lsp.nwu.edu.cn/molecule.php?qn=9986) | 36.13 | 0.8 | 6.95 |

Doukou (*Fructus Amomi Rotundus,* FAR)

| SMILES | Mumbers | [**Mol ID**](http://lsp.nwu.edu.cn/tcmspsearch.php?qr=Gynostemmae Pentaphylli Herba&qsr=herb_en_name&token=625d16b741fb2795959ada57190a7cd4) | [**Molecule Name**](http://lsp.nwu.edu.cn/tcmspsearch.php?qr=Gynostemmae Pentaphylli Herba&qsr=herb_en_name&token=625d16b741fb2795959ada57190a7cd4) | [**OB (%)**](http://lsp.nwu.edu.cn/tcmspsearch.php?qr=Gynostemmae Pentaphylli Herba&qsr=herb_en_name&token=625d16b741fb2795959ada57190a7cd4) | [**DL**](http://lsp.nwu.edu.cn/tcmspsearch.php?qr=Gynostemmae Pentaphylli Herba&qsr=herb_en_name&token=625d16b741fb2795959ada57190a7cd4) | [**HL**](http://lsp.nwu.edu.cn/tcmspsearch.php?qr=Gynostemmae Pentaphylli Herba&qsr=herb_en_name&token=625d16b741fb2795959ada57190a7cd4) |
| --- | --- | --- | --- | --- | --- | --- |
| O1[C@H](CC(=O)c2c1cc(O)cc2OC)c1ccccc1 | FAR-1 | MOL000228 | [(2R)-7-hydroxy-5-methoxy-2-phenylchroman-4-one](http://lsp.nwu.edu.cn/molecule.php?qn=228) | 55.23 | 0.2 | 17.02 |
| c1(cc(c2c(c1)O[C@@H](CC2=O)c1ccccc1)O)O | FAR-2 | MOL000230 | [Pinocembrin](http://lsp.nwu.edu.cn/molecule.php?qn=230) | 57.56 | 0.2 | 16.85 |
| c1ccc(cc1)/C=C/[C@@H](C[C@@H](CCc1ccccc1)C)O | FAR-3 | MOL000235 | [1,7-diphenyl-3,5-dihydroxy-1-heptene](http://lsp.nwu.edu.cn/molecule.php?qn=235) | 49.01 | 0.18 | 7.33 |
| O[C@@H](CC(=O)CCc1ccccc1)/C=C/c1ccccc1 | FAR-4 | MOL000238 | [1,7-diphenyl-5-hydroxy-6-hepten-3-one](http://lsp.nwu.edu.cn/molecule.php?qn=238) | 32.65 | 0.18 | 4.32 |
| o1c2c(c(=O)c(OC)c1c1ccc(O)cc1)c(O)cc(OC)c2 | FAR-5 | MOL000239 | [Jaranol](http://lsp.nwu.edu.cn/molecule.php?qn=239) | 50.83 | 0.29 | 15.5 |
| O1[C@@H](CC(=O)c2c1cc(OC)cc2O)c1cc(O)c(O)cc1 | FAR-6 | MOL000242 | [7-O-Methyleriodictyol](http://lsp.nwu.edu.cn/molecule.php?qn=242) | 56.56 | 0.27 | 14.99 |
| c1(cc(ccc1O)[C@H]1Cc2c(O1)c(cc(c2)/C=C/C)OC)OC | FAR-7 | MOL000258 | [dehydrodiisoeugenol](http://lsp.nwu.edu.cn/molecule.php?qn=258) | 56.84 | 0.29 | 8.36 |
| O1[C@H]([C@@H](c2c1c(OC)cc(c2)/C=C/C)C)c1cc2OCOc2cc1 | FAR-8 | MOL000260 | [5-[(2R,3R)-7-methoxy-3-methyl-5-[(E)-prop-1-enyl]-2,3-dihydrobenzofuran-2-yl]-1,3-benzodioxole](http://lsp.nwu.edu.cn/molecule.php?qn=260) | 65.55 | 0.4 | 11.93 |
| o1c2c(c(=O)cc1c1cc(O)c(O)cc1)c(O)cc(O)c2 | FAR-9 | MOL000006 | [luteolin](http://lsp.nwu.edu.cn/molecule.php?qn=6) | 36.16 | 0.25 | 15.94 |
| o1c2c(c(=O)c(O)c1c1cc(O)c(O)cc1)c(O)cc(O)c2 | FAR-10 | MOL000098 | [quercetin](http://lsp.nwu.edu.cn/molecule.php?qn=98) | 46.43 | 0.28 | 14.4 |
