# Supplementary material for: Integrated Analyses Identify Immune-Related Signature Associated with Qingyihuaji Formula for Treatment of Pancreatic Ductal Adenocarcinoma Using Network Pharmacology and Weighted Gene Co-Expression Network
Source: J Immunol Res. 2020 May 20;2020:7503605. doi: 10.1155/2020/7503605 (PMC7256764; doi:10.1155/2020/7503605)
Supplement: Supplementary Materials — Table 1S. The PDAC genes in the GeneCards database Supplementary file 2: Table 2S. The immune-related targets in the GeneCards database Supplementary file 3: Table 3S. The detailed ADME parameters and the SMILES information of the aforementioned 67 selected compounds. Supplementary file 4: Figure S1. The potential active ingredients-target network of QYHJ. Supplementary file 5: Table 4S. The association between active ingredients and up-regulated overlapping genes. Supplementary file 6: Figure S2. Association of Methylation sites with the expression of 12 overlapped DEGs. [file 7503605.f1.zip › Supplementary file Table 4S.doc]

**Supplementary Table Ⅲ** The association between active ingredients and up-regulated overlapping genes.

| Targets | Active ingredients | Count | Total |
| --- | --- | --- | --- |
| CDK1 | RAE-1, -2 | 2 | 26 |
|  | HSB-3, -4, -5, -6, -17, -18,-20,-22, -23, -27, -28, -29 | 12 |  |
|  | HBHY-4, -7 | 2 |  |
|  | HSRGP-2, -9 | 2 |  |
|  | FAR-1, -4, -5, -6, -7, -8, -9, -10 | 8 |  |
| PLD1 | RAE-2 | 1 | 1 |
| MET | RAE-2 | 1 | 26 |
|  | HSB-2, -3, -4, -12, -17, -18, -20, -21, -22, -23, -25, -26, -27, -28, -29 | 15 |  |
|  | HBHY-7 | 1 |  |
|  | HSRGP-1, -2, -9 | 3 |  |
|  | FAR-1, -2, -5, -6, -9, -10 | 6 |  |
| F2RL1 | RAE-2 | 1 | 2 |
|  | HSRGP-4 | 1 |  |
| XDH | HSB-3, -4, -5, -6, -8, -12, -17, -18, -20, -22,-23, -27, -28, -29 | 14 | 20 |
|  | HBHY-7 | 1 |  |
|  | HSRGP-2, -9 | 2 |  |
|  | FAR-5, -9, -10 | 3 |  |
| NEK2 | HSB-3, -5, -12, -18, -23, -27, -29 | 7 | 12 |
|  | HBHY-7 | 1 |  |
|  | HSRGP-2, -9 | 2 |  |
|  | FAR-9, -10 | 2 |  |
| TOP2A | HSB-5, -6, -12, -17, -20, -23, -29 | 7 | 13 |
|  | HBHY-3, -7 | 2 |  |
|  | HSRGP-2, -5, -9, -11 | 4 |  |
|  | FAR-5, -10 | 2 |  |
| NQO1 | HSB-12 | 1 | 3 |
|  | HBHY-7 | 1 |  |
|  | FAR-7 | 1 |  |
| CCND1 | HBHY-4 | 1 | 3 |
|  | FAR-3, -4 | 2 |  |
| PTK6 | HSRGP-15 | 1 | 1 |
| CTSE | HSRGP-15 | 1 | 1 |
| ERBB2 | HSRGP-15 | 1 | 1 |
